# Cesium Carbonate Promoted Direct Amidation of Unactivated Esters with Amino Alcohol Derivatives

**DOI:** 10.1021/acs.joc.4c00162

**Published:** 2024-03-25

**Authors:** Chih-Hung Kuo, Wen-Tsai Hsieh, Ya-Hsu Yang, Teng-Li Hwang, Yu-Shan Cheng, Yuya A. Lin

**Affiliations:** † Department of Chemistry, National Sun Yat-sen University, Kaohsiung 804, Taiwan; ‡ Department of Medicinal and Applied Chemistry, Kaohsiung Medical University, Kaohsiung 807, Taiwan

## Abstract

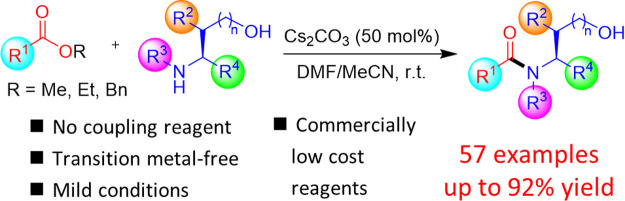

Cesium carbonate promoted direct amidation of unactivated esters with amino alcohols was developed without the use of transition-metal catalysts and coupling reagents. This method enabled the synthesis of several serine-containing oligopeptides and benzamide derivatives with yields up to 90%. The methodology proceeds under mild reaction conditions and exhibits no racemization for most naturally occurring amino acid substrates. The reaction demonstrates good compatibility with primary alkyl and benzyl esters and broad tolerance for a range of amino acid substrates with nonpolar and protected side chains. The hydroxy group on the amine nucleophile was found to be critical for the reaction to be successful. A likely mechanism involving cesium coordination to the substrates enabling the subsequent proximity-driven acyl transfer was proposed. The practicality of this approach was demonstrated in the preparation of a biologically active nicotinamide derivative in a reasonable yield.

## Introduction

Peptides are important structural moieties present in many natural products and pharmaceuticals.^1^ Despite being one of the industry’s most frequently performed chemical transformations,^2^ peptide synthesis still depends heavily on the use of aminium- or phosphonium-based coupling reagents and additives such as HOBt or Oxyma to minimize racemization making the process poor regarding atom economy.^3^ Furthermore, the protecting group strategy for either the amine or the carboxylic acid is also critical to achieving reaction specificity in the key amide-formation step. For peptide synthesis involving residues containing nucleophilic side chains, additional protecting groups orthogonal to the ones at the N- and C-terminal are required, further decreasing the atom economy and the cost-effectiveness of the synthesis. These are the ongoing challenges that remain in peptide synthesis. Serine (Ser)- and threonine (Thr)-containing oligopeptides have high pharmaceutical potential since these residues are very frequently found in nonribosomal peptides (NRPs),^4^ a class of compounds produced by microbes that have a broad spectrum of biological activities. The presented hydroxyl groups in these amino acids allow the formation of branched primary structures in NRPs, for instance, in syringomycins^5^ and actinomycine D.^6^ Thus, developing a synthetic methodology for the synthesis of serine- and threonine-containing oligopeptides would aid research in these fields. Native chemical ligation is one of the most widely applied and developed chemoselective amidation method that utilizes the nucleophilic nature of cysteine at the N-terminus.^7^ There are also other reported amidation methods based on the acyl transfer reactions of the nucleophilic side chains in serine/threonine,^8^ tryptophan,^9^ and histidine residues.^10^ While some of these examples could be accomplished under mild conditions and are protecting-group-free, the reactions were found to work only with reactive esters such as salicylaldehyde esters, OBt esters, or thioesters, which are often prepared by conventional methods using coupling reagents.

**Scheme 1:**
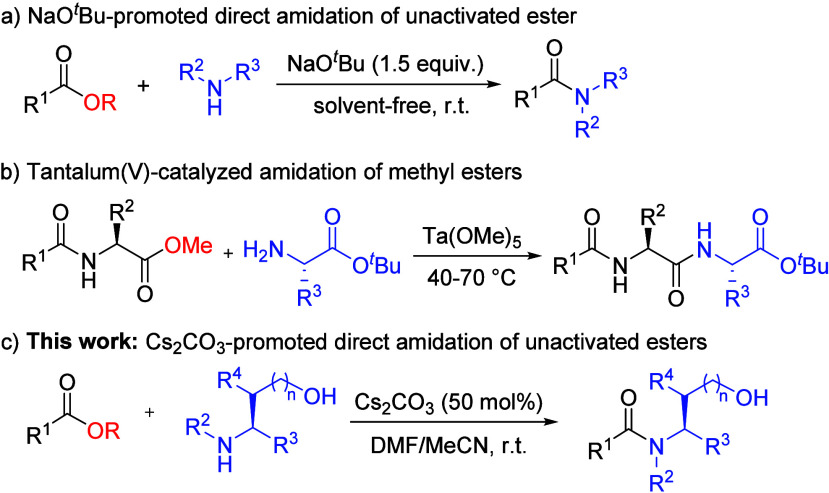
Methods for Direct Amidation of Unactivated Esters

Direct amidation of unactivated esters such as methyl esters is an attractive and greener synthetic alternative to conventional methods due to the improved atom economy since the only major byproduct would simply be methanol. However, direct amidation of alkyl esters often requires a base-promoter, transition metal catalyst, or organocatalyst due to their poor reactivity.^11^ The base-promoted amidation methods have been shown to involve bases such as KO^*t*^Bu,^12^ BEMP,^13^ LiHMDS,^14^ and NaO^*t*^Bu,^15^ which can achieve good to excellent yields with low-cost, commonly available reagents (Scheme 1a) but the substrate scopes are mainly limited to simple amides and not applicable for peptide synthesis due to the increased risk of racemization under strongly basic conditions. The reported transition metal catalyzed direct amidations of esters have involved the use of La(III),^16^ Mn(I),^17^ Ni(0)^18^ or Pd(0)^19^ complexes often at high reaction temperatures (50–140 °C) for a long time making these incompatible for peptide synthesis. While some reported cases of direct amidation of methyl esters using transition metal catalysts such as Ta(OMe)_5_ can be applied to short peptide synthesis without racemization (Scheme 1b),^20^ the downside of such methods is the high cost of the metal catalysts. In this work, a method utilizing a commonly available base, cesium carbonate, as a promotor for the direct amidation of unactivated esters (alkyl esters) with unprotected serine, threonine, and other amino alcohol derivatives has been disclosed (Scheme 1c). This method enables the synthesis of various serine-containing oligopeptides and benzamides without the use of coupling reagents and transition metal catalysts.

## Results and Discussion

### Reaction Optimization

With an aim to develop a methodology compatible with peptide synthesis, the reaction condition optimization was conducted with glycine methyl ester (**3a**) and serine derivative **2a** as substrates in the presence of a catalytic amount of Cs_2_CO_3_. A *N*-hexyl amide derivative of serine was used due to the conveniently available reagents in the laboratory and the better solubility in organic solvents. The base Cs_2_CO_3_ was selected at first because cesium salts have been demonstrated to promote various reactions in organic synthesis.^21^ We were further inspired by the work of Meng and Fürstner on the total synthesis of (−)-Sinulariadiolide,^22^ in which a unique reactivity of cesium ion was proposed; we hypothesized that the oxophilic cesium ion could coordinate the carbonyl groups of the methyl ester and serine derive and facilitate the amidation in a similar manner. The reaction solvent and amount of the amine and base promoter were optimized (entries 1–8, Table 1). The results showed that the direct amidation at methyl ester can indeed proceed to give the dipeptide product **6aa** in the best yield of 78% using 50 mol % of Cs_2_CO_3_ and DMF as the solvent (entry 8). During the reaction, the starting materials appeared to have poor solublility in DMF, and if the reaction was conducted in acetonitrile, the reaction intermediates would precipitate out from the solution. Thus, the reaction conditions were further optimized using a mixed solvent system of DMF and acetonitrile. With a 1:3 ratio of DMF and acetonitrile, the product could be obtained with an improved yield of 87% and is pure without chromatography purification (Table 1, entry 10). When the amount of Cs_2_CO_3_ in reaction is reduced to 20 mol % under the mixed solvent system, the yield decreased to 64% (Table 1, entry 11) suggesting that 50 mol % should be the optimal loading of the base promotor. The optimized reaction conditions allowed the reaction to be conducted at 5 times the original scale (2.64 mmol) without any decrease in yield (Table 1, entry 12). With the previous reports on base-promoted amidation, a range of alternative bases including ^*t*^BuOK, K_2_CO_3_, KHCO_3_, LiHMDS, KHMDS, DIEA, and DBU were also investigated. When these bases were used instead of Cs_2_CO_3_, a significant decrease in the product yields was observed (Table 1, entries 13–19). The presence of Cs_2_CO_3_ was further demonstrated to be critical for the reaction to work since only 5% of the amidation product was isolated if the base was not added (Table 1, entry 20). Next, the reactivity of various esters under the optimized reaction conditions was investigated. Ethyl and benzyl esters reacted similarly to the methyl ester resulting in 80% and 96% of the amidation product, respectively (Table 1, entries 21–22). The amidation with *tert*-butyl ester **3d** obtained only a 29% yield which could be attributed to the steric effects of the bulky group that hinders nucleophilic attack from serine (Table 1, entry 23). The poor yield of 35% obtained from phenolic ester **3e** was initially unexpected since phenolate should be a better leaving group than alkoxides (Table 1, entry 24). This result suggested that the leaving group likely acted as a base deprotonating another molecule of amino-alcohol substrate, allowing the next reaction cycle to continue. In the case of the phenolic ester, the phenolate leaving group would not be basic enough to deprotonate the amino alcohol. A similar trend in the reactivity of various esters was also observed in the work by Qin on NaO^*t*^Bu-promoted amidation of unactivated esters.^15^ These studies showed that the direct amidation of alkyl esters is affected by the choice of base, solvent system, the steric bulk of the ester, and the basicity of the leaving group.

**Table 1:**
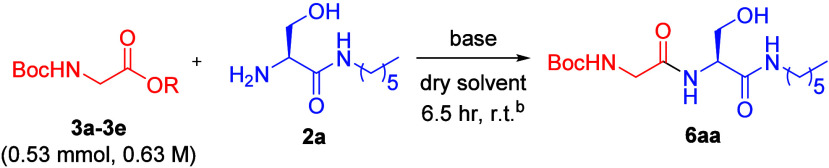
Reaction Optimization

Entry	R	Ester:**2a**	Solvent	Base (mol %)	Yield (%)^f^
1^a^	Me (**3a**)	1.1:1	THF	Cs_2_CO_3_ (10)	55
2^a^	Me (**3a**)	1.1:1	DCE	Cs_2_CO_3_ (10)	51
3^a^	Me (**3a**)	1.1:1	MeCN	Cs_2_CO_3_ (10)	63
4^a^	Me (**3a**)	1.1:1	MeCN	Cs_2_CO_3_ (50)	71
5^a^	Me (**3a**)	1.1:1	MeCN	Cs_2_CO_3_ (100)	72
6^a^	Me (**3a**)	1.1:1	MeCN	Cs_2_CO_3_ (120)	71
7^c^	Me (**3a**)	1:2	MeCN	Cs_2_CO_3_ (50)	74
8^c^	Me (**3a**)	1:2	DMF	Cs_2_CO_3_ (50)	78
9^c^	Me (**3a**)	1:2	DMF:MeCN (1:1.1)	Cs_2_CO_3_ (50)	77
**10**^c^^,^^d^	Me (**3a**)	1:2	**DMF:MeCN** (1:3)	**Cs**_**2**_**CO**_**3**_**(50)**	**87**
11	Me (**3a**)	1:2	DMF:MeCN (1:3)	Cs_2_CO_3_ (20)	64
12^a^^,^^e^	Me (**3a**)	1:2	DMF:MeCN (1:3)	Cs_2_CO_3_ (50)	85
13	Me (**3a**)	1:2	DMF:MeCN (1:3)	^*t*^BuOK (50)	5
14	Me (**3a**)	1:2	DMF:MeCN (1:3)	K_2_CO_3_ (50)	19
15	Me (**3a**)	1:2	DMF:MeCN (1:3)	KHCO_3_ (50)	8
16	Me (**3a**)	1:2	DMF:MeCN (1:3)	KHMDS (50)	42
17^c^	Me (**3a**)	1:2	DMF:MeCN (1:3)	LiHMDS (50)	52
18	Me (**3a**)	1:2	DMF:MeCN (1:3)	DIEA (50)	5
19	Me (**3a**)	1:2	DMF:MeCN (1:3)	DBU (50)	66
20	Me (**3a**)	1:2	DMF:MeCN (1:3)	None	5
21^a^	Et (**3b**)	1:2	DMF:MeCN (1:3)	Cs_2_CO_3_ (50)	80
**22**^a^	**Bn (3c)**	1:2	**DMF:MeCN** (1:3)	**Cs**_**2**_**CO**_**3**_**(50)**	**96**
23^a^	^*t*^Bu (**3d**)	1:2	DMF:MeCN (1:3)	Cs_2_CO_3_ (50)	29
24^a^	Ph (**3e**)	1:2	DMF:MeCN (1:3)	Cs_2_CO_3_ (50)	35

a 24 h.

b r.t. stands for room temperature (25 °C - 30 °C).

c Column chromatography not required.

d Optimized reaction condition: **2a** (0.2 g, 1.06 mmol), **3a** (0.1 g, 0.53 mmol) and Cs_2_CO_3_ (0.87 g, 0.27 mmol) in 1:3 DMF/MeCN (0.85 mL) was reacted for 6.5 h at room temperature. After reaction work up by aqueous extraction, the pure product **6aa** was obtained (0.16 g, 87%).

e Yield 0.78 g (85%) of **6aa** from 0.5 g (2.64 mmol) of **3a**, 1.0 g (5.31 mmol) of **2a**, and 0.435 g (1.32 mmol) of Cs_2_CO_3_.

f Isolated yield.

### Substrate Scope Investigation

**Scheme 2:**
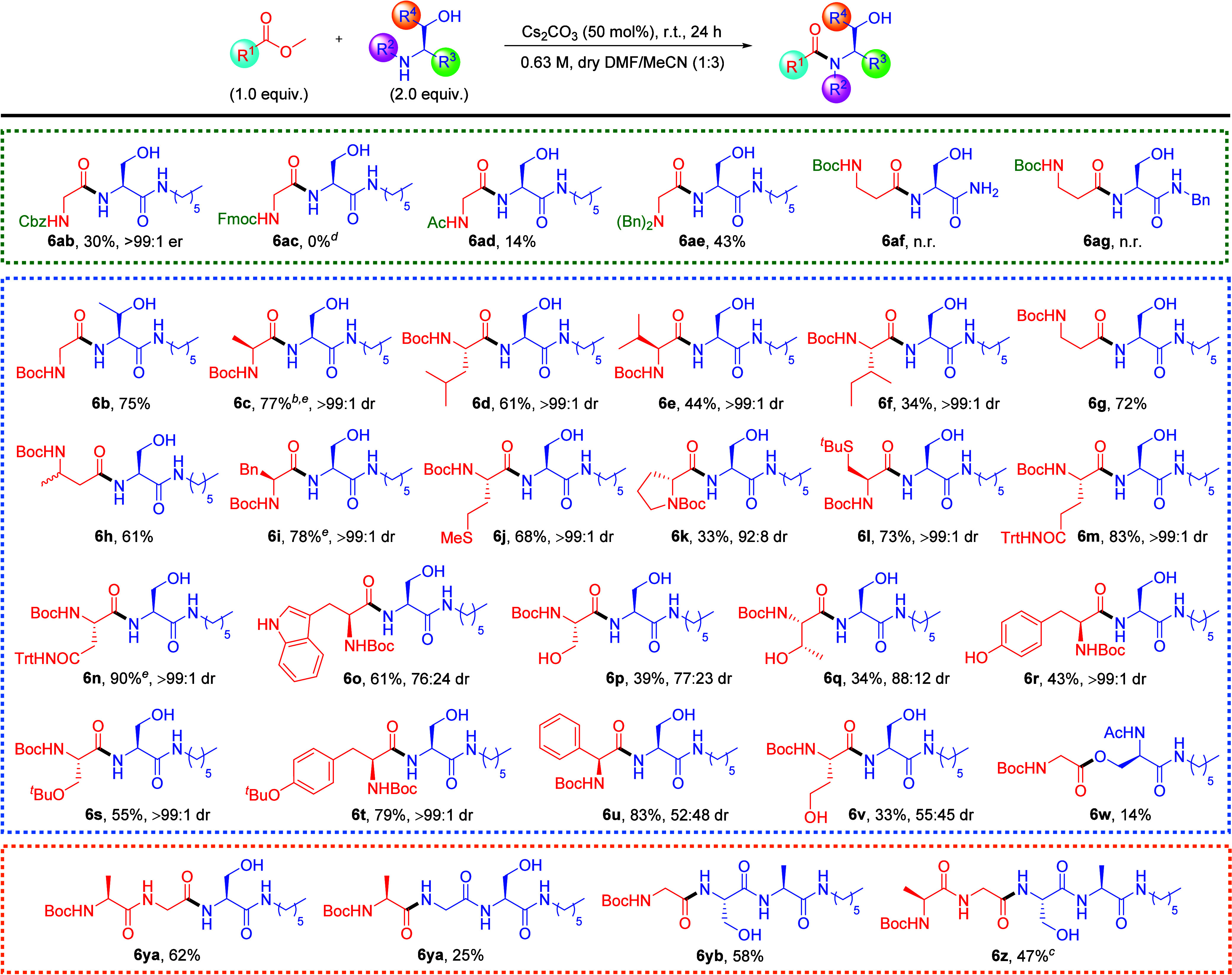
Evaluation of Amino Acid and Dipeptide Substrate Scope of Cs_2_CO_3_-Promoted Direct Amidation of Methyl Esters with Serine/Threonine Derivatives % yields refer to isolated yields. n.r. = no reaction. The er of **6ab** and dr’s of **6i** and **6u** were determined by HPLC. The dr’s of **6c**–**6f**, **6i**–**6v** were determined by ^1^H NMR. The reaction time was 6.5 h. The reaction time was 48 h. Complex mixture. Column chromatography purification was not required.

Although benzyl ester **3c** provided the best yield of 96% in the model reaction study, the substrate scope of the reaction will be examined using various methyl esters because these would generate a smaller molecular weight byproduct (methanol). With the optimized reaction conditions in hand, the substrate scope was first investigated with glycine containing different *N*-terminal protecting groups, various amino acids methyl esters, and dipeptides (Scheme 2). Among the different *N*-protecting groups tested, Boc-protected substrates performed the best (87%) while Cbz-, Ac-, and dibenzyl-protected derivatives (**4a**, **4c**, **4d**) resulted in low yields ranging from 14% to 43% (**6ab**, **6ad**, **6ae**) due to limited solubility in DMF/MeCN. Unreacted ester starting materials were observed at the end of the reaction. Despite the lower yields, chiral HPLC analysis of dipeptide **6ab** showed >99% e.e. meaning that the reaction condition does not result in the racemization of the α-hydrogen. The Fmoc protecting group is base-labile thus the reaction between Fmoc-Gly-OMe (**4b**) and serine **2a** resulted in complex mixtures caused by side reactions such as Fmoc deprotection even under these mildly basic reaction conditions. Other amide functional group on serine was also screened under optimized reaction conditions with Boc-βAla-OMe (**5e**). Serine amide substrates such as H-Ser-NH_2_ (**2f**) and H-Ser-NHBn (**2g**) were found to be not soluble in the solvent system used and ester starting material remained unreacted due to poor homogeneity during the reaction. When threonine was used instead of serine, the Cs_2_CO_3_-promoted reaction conditions could yield 75% of dipeptide **6b**. This slightly decreased yield is likely due to the steric effect from the β-methyl group. Next, a range of amino acid derivatives containing nonpolar side chains were investigated. The yields of the dipeptide products of alanine (**6c**), leucine (**6d**), valine (**6e**), isoleucine (**6f**), β-alanine (**6g**), homoalanine (**6h**), phenylalanine (**6i**), and proline (**6k**) ranged from 77% to 33%. The yields decrease as the steric hindrance of the methyl ester substrates at β-position (e.g., valine, isoleucine) increases or has a rigid structure (e.g., proline). The reaction can tolerate a range of functional groups including thioether, amide, and indole. Good to excellent yields ranging from 60% to 90% were observed for dipeptides **6j**, **6l**, **6m**, **6n**, and **6o**. The reaction, however, does not tolerate the presence of the hydroxy group on the methyl ester substrate, which is thought to perturb the reaction enhancement by the base promotor. Poor yields of 33% to 43% were observed for products **6p**, **6q**, **6r**, **6v**. Under the basic reaction conditions, methyl esters containing an acidic proton in the side chain (Trp, Ser, Thr, Tyr, Hse) will be transformed to the corresponding conjugate bases that seemed to promote some racemization, evident from the lower d.r. values observed for product **6o**, **6p**, **6q** and **6v**. Notably, a γ-lactone product was also isolated from the direct amidation with homoserine methyl ester (**5t**) indicating that an intramolecular lactonization may have occurred first under such reaction conditions. Racemization on the γ-lactone occurs more easily due to the more acidic α-hydrogen, and a poor d.r. of 55:45 was observed for **6v**. The tyrosine-containing product **6r**, however, experienced no racemization (>99:1 d.r.) during the amidation owing to the less basic phenolate side chain. When the hydroxy groups on serine and tyrosine esters were protected, the amidation yields for these substrates were improved significantly and 55% and 79% yields were obtained for **6s** and **6t**, respectively. The amidation product **6s**, containing *O*-protected serine, also has an improved d.r. of >99:1. Phenylglycine is another amino acid particularly prone to racemization, and a 52:48 diastereomeric mixture of **6u** was obtained despite the good yield of 83%. Nevertheless, the reaction conditions for Cs_2_CO_3_-promoted amidation were mild and no racemization was observed for most amino acid methyl esters, evident from the high d.r. values obtained. It should be noted that the amidation does not work when the hydroxy group is protected (e.g., H-Ser(^*t*^Bu)-NHHex) or absent (e.g., H-Ala-NHHex) in the amine nucleophiles (Table S1, Supporting Information), and only starting materials were isolated. In the case of *N*-acetyl protected amino alcohol substrate, Ac-Ser-NHHex (**2c**), only the ester product **6w** was isolated, revealing some mechanistic insight into the reaction. These results demonstrated the necessity of the amino alcohol moiety in these reactions. The methodology can further be applied to tri- and tetrapeptide synthesis, and moderate yields between 47% to 62% could be obtained for products **6ya**, **6yb**, and **6z**. Notably, when the tripeptide Boc-AlaGlySer-NHHex (**6ya**) was synthesized from Boc-Ala-OMe and H-GlySerNHHex the product yield was only 25%, which is significantly lower compared to the synthesis from Boc-AlaGly-OMe and H-Ser-NHHex (62%). This result indicated the importance of having the amino-alcohol moiety at the *N*-terminus and that a hydroxyl group further away from the amidation site could not promote the reaction. The 25% yield of **6ya** from H-GlySerNHHex is likely a result of direct amidation by the N-terminal glycine, which is not very efficient.

**Scheme 3:**
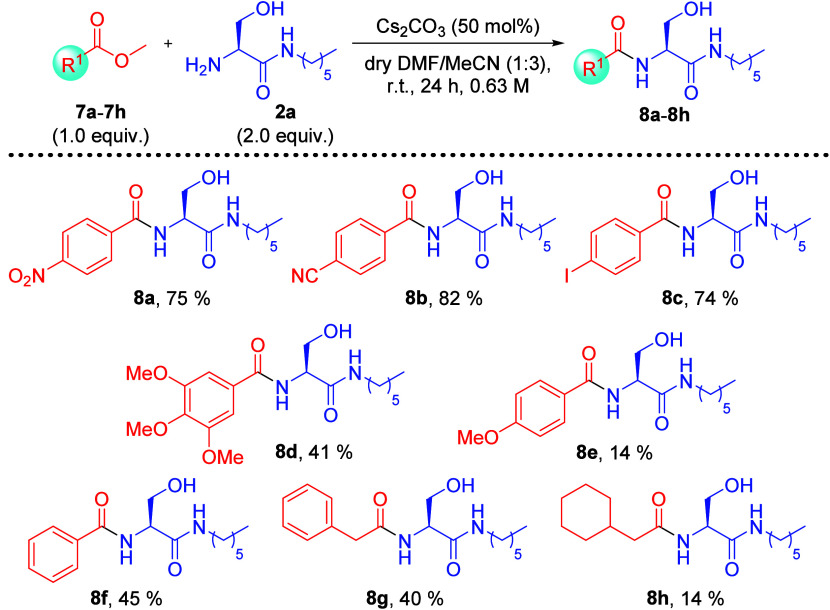
Substrate Scope Study of Cs_2_CO_3_-Promoted Direct Amidation of Methyl Esters **7a**–**7h** with Serine Derivative **2a** % Yields refer to isolated yields.

**Scheme 4:**
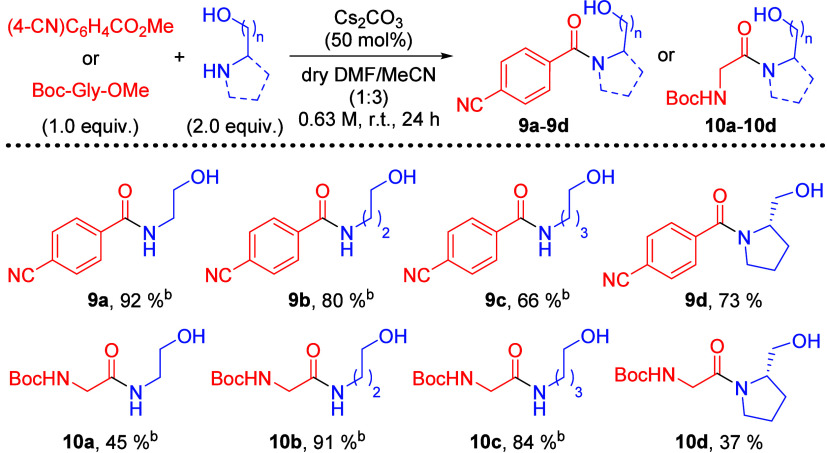
Comparing the Effect of Amino Alcohols of Various Carbon Chain Lengths in Cs_2_CO_3_-Promoted Direct Amidation of Methyl Ester **7b** and **3a** % Yields refer to isolated yields. Aqueous extraction omitted during reaction work up.

When the substrate scope was investigated with methyl benzoate derivatives **7a** to **7f**, methyl phenylacetate (**7g**), and methyl cyclohexylacetate (**7h**) (Scheme 3), it was clear that the electronic properties of the aromatic substituents affect the efficiency of amidation with serine derivative **2a**. Yields from 74% to 82% of amidation product (**8a**–**8c**) were obtained with electron-withdrawing groups para to the methyl esters (**7a**–**7c**) while poor to moderate yields ranging from 14% to 45% (**8d**–**8h**) were obtained from methyl esters containing electron-donating substituents (**7d**–**7h**). Similar trends have been observed for cesium carbonate promoted esterification of *N*-benzyl-*N*-Boc amides.^21a^ Further amidation studies using simple amino alcohols of various carbon chain lengths were conducted with methyl 4-cyanobenzoate (**7b**) and Boc-Gly-OMe (**3a**) (Scheme 4). In the case of methyl 4-cyanobenzoate, the best yield of 92% was obtained with ethanolamine and the yields decreased as the carbon tether became longer. On the other hand, 3-amino-1-propanol performed the best among other aliphatic amino alcohols in the amidation with Boc-Gly-OMe and 91% yield of the product **10b** was obtained. From the result of **6ya** (Scheme 2), one should expect the yield to decrease more significantly as the hydroxyl group becomes further away. However, 66% of **9c** and 84% of **10c** can be obtained. Two explanations were proposed to rationalize these results. First, for an electron-deficient substrate like **7b**, it is possible that the simple amino alcohols can efficiently react via the amine without the enhancement from the hydroxyl group. Second, the reaction enhancement from the hydroxyl group of amino alcohols could also be operating intermolecularly due to the small size of these substrates and thus not be limited by the distance away from amidation site. In addition, it was interesting to find that the reaction between serine derivative **2a** and simple benzoate derivatives under the optimized conditions resulted in lower yields compared to the more complex dipeptide examples. This observation suggests that the carbamate protecting group of the amino acid substrates could likely act as a ligand to coordinate the Cs^+^ ion, similar to the substrate-directed catalytic model proposed by Yamamoto et al.^20^

**Scheme 5:**
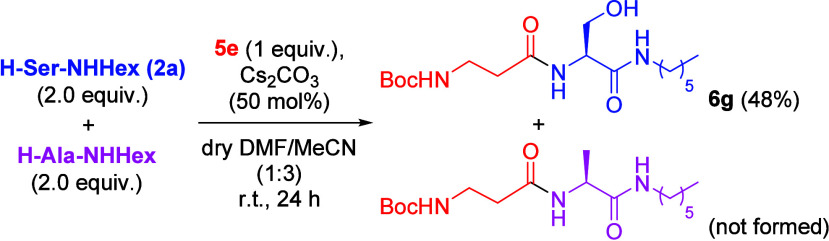
Competition Experiment between **2a** and **H-Ala-NHHex** with **5e** under Cs_2_CO_3_-Promoted Amidation Conditions % Yields refer to isolated yields.

To examine the specificity of Cs_2_CO_3_-promoted amidation with amino acids containing hydroxyl groups like serine and threonine, serine derivative **2a** was subjected to amidation conditions with Boc-βAla-OMe (**5e**) in the presence of another amino acid nucleophile, H-Ala-NHHex (Scheme 5). The additional nucleophilic competitor can interfere with the reaction environment causing a complex reaction mixture, but we were pleased to know that a single major product **6g** could be obtained in 48% yield. Boc-βAlaAla-NHHex, the product resulted from the amidation of H-Ala-NHHex, was not formed.

**Scheme 6:**
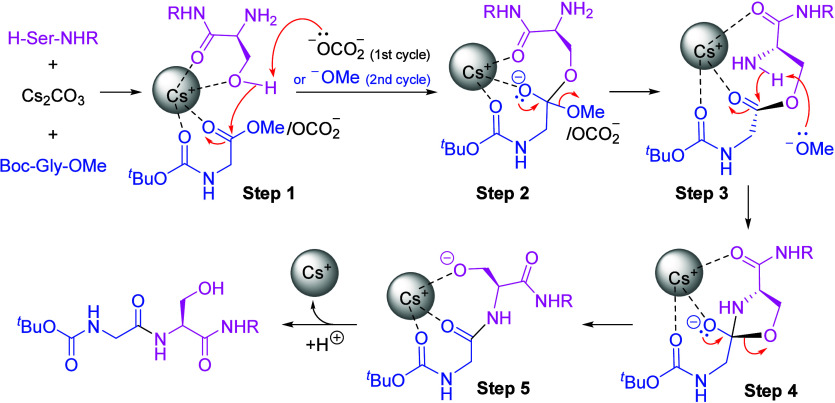
Proposed Mechanism of Cs_2_CO_3_-Promoted Direct Amidation of Amino Acid Methyl Ester

After compiling the experimental evidence and precedent literature, a plausible mechanism was developed to explain the Cs_2_CO_3_-promoted amidation of unactivated ester by amino alcohols (Scheme 6). First, there are several examples of reaction enhancement by cesium ions in organic synthesis based on their ability to coordinate oxygen atoms.^22,23^ The fact that amino acid substrates react better than methyl benzoates in our studies (Scheme 2, 3) led us to propose the cesium ion to act as a mild Lewis acid. The cesium ion can coordinate the oxygen-containing groups in the reactants; such preorganization brings the substrates to proximity allowing the reaction to occur readily. Second, the carbonate ester has been proposed as a possible intermediate in Cs_2_CO_3_-promoted esterification.^21a^ Similarly, in Cs_2_CO_3_-promoted amidation, the carbonate anion may undergo an ester exchange with methyl ester resulting in a proposed structure shown in **Step 1**. From the result of the study using various ester substrates (Table 1, entries 21–24), the efficiency of the amidation reaction seemed to be affected by the basicity of the ester leaving group. This led us to propose that the deprotonation of the acidic proton on serine is likely by the carbonate anion in during the first cycle, and by the methoxide in the subsequent cycles. Finally, the unsuccessful amidation with *O*-protected serine (Table S1, entry 1) and the formation of ester **6w** from Ac-Ser-NHHex (**2c**) in the substrate scope study suggested likely mechanistic steps involving an initial transesterification between the ester and amino alcohol (**Step 1–2**), followed by an intramolecular *O*-to-*N* acyl transfer (**Step 3–5**) and then aqueous workup to yield the amidation product.

**Scheme 7:**
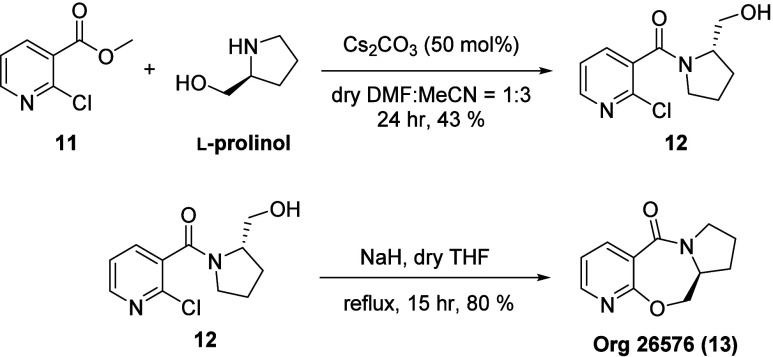
**Org 26576** Synthesis via Cs_2_CO_3_-Promoted Amidation at Unactivated Ester % Yields refer to isolated yields.

Finally, the developed cesium carbonate promoted amidation of amino alcohols was applied in the synthesis of a medicinally relevant molecule **Org 26576** (**13**), which is a potentiator for α-amino-3-hydroxy-5-methylisoxazole-4-propionic acid (AMPA) receptor (Scheme 7).^24^ The amidation was carried out with the commercially available methyl nicotinate derivative **11** and l-prolinol to yield the intermediate amide **12** in 43% yield. The yield obtained was comparable to the case with methyl benzoate derivatives in the substrate scope study. The subsequent S_N_Ar chemistry under basic conditions was conducted according to previously reported procedures^13^ to furnish the target compound **Org 26576** in 80% yield.

## Conclusions

In summary, a method of performing amidation at unactivated esters has been developed. This methodology requires no coupling reagents nor expensive metal catalysts and is without racemization for naturally occurring amino acid substrates that do not have acidic protons in the side chains. The substrate scope is thoroughly examined with 57 examples, in which the reaction demonstrated broad compatibility toward various amino acids, dipeptides, and methyl benzoate derivatives with moderate to good yields. The hydroxy group on the amino alcohol was essential for effective amidation to occur. In addition, amidation with serine can occur selectively in the presence of another amino acid nucleophile without the hydroxyl group. Together with prior literature, a plausible mechanism based on cesium ion coordination to the substrates enabling a proximity-driven nucleophilic attack followed by an intramolecular transacylation was proposed. Finally, a biologically active molecule was synthesized from commercially available starting materials in two steps utilizing the methodology developed.

## Experimental Section

### General Considerations

Melting points were recorded by Fargo Instrument MP-ID. The temperature range is between 25 to 300 °C. Optical rotations were measured on a polarimeter (ATAGO, POLAX-2L) with a 200 mm (10 mL) observation tube (OT-200(P)). Nuclear magnetic resonance (NMR) spectra were recorded on a JEOL JNM-ECS 400 (^1^H: 400 MHz; ^13^C{^1^H}: 100 MHz), or a Bruker Avance 300 MHz-NMR (^1^H: 300 MHz; ^13^C{^1^H}: 75 MHz) spectrometer, as indicated. All chemical shifts are quoted on the δ scale in ppm using residual solvent as the internal standard (^1^H NMR: CDCl_3_ = 7.26, CD_3_OD = 3.31; DMSO-*d*_6_ = 2.50 and ^13^C{^1^H} NMR: CDCl_3_ = 77.1; CD_3_OD = 49.0; DMSO-*d*_6_ = 39.5) and CFCl_3_ as the external standard. Coupling constants (*J*) are reported in Hz with the following splitting abbreviations: s = singlet, d = doublet, t = triplet, q = quartet, quin = quintet, m = multiplet, and app = apparent. Raw fid files were processed by Bruker TopSpin 4.0.7 or JEOL Delta 5.2.1 software. Infrared (IR) spectra were recorded on a PerkinElmer Spectrum Two FT-IR Spectrometer with UATR Diamond/ZnSe ATR (L160000A). Absorption maxima (ν_max_) are reported in wavenumbers (cm^–1^). High-resolution mass spectra (HRMS) were recorded on the Orbitrap Exploris 120 mass spectrometer (Thermo Fisher) (ESI) or AccuTOF GCx-plus (JEOL) (EI). Normal and exact *m*/*z* values are reported in daltons. Thin-layer chromatography (TLC) was carried out using Merck aluminum backed sheets coated with 60F_254_ silica gel. Visualization of the silica plates was achieved using a UV lamp (λ_max_ = 254 nm), and/or potassium permanganate (5% KMnO_4_ in 1 M NaOH with 5% potassium carbonate). Flash column chromatography was carried out using Silica Flash P60 (40–60 μm) purchased from SiliCycle. Mobile phases are reported in the ratio of solvents for binary systems (e.g., ethyl acetate:*n*-hexane = 1:4). Chiral HPLC was performed using Agilent 1260 Infinity HPLC equipped with the Agilent 1260 Infinity II Diode Array Detector and Daicel CHIRALPAK IA-3 Column (4.6 mm × 25 cm). Anhydrous dichloromethane, *N,N*-dimethylformamide, and methanol were distilled over calcium hydride. Anhydrous acetonitrile was first distilled over P_2_O_5_ and then distilled for the second time over calcium hydride. Anhydrous tetrahydrofuran was distilled over sodium metal. All other solvents were used as supplied (Analytical or HPLC grade), without prior purification. Unless noted, all materials were purchased from commercial suppliers (Sigma-Aldrich, Alfa Aeasar or Nova-Matls) and used as received. Distilled water was used for chemical reactions. All reactions using anhydrous conditions were performed using a flame-dried apparatus under an atmosphere of nitrogen. Workups were carried out in the air. Brine refers to a saturated solution of sodium chloride. Anhydrous magnesium sulfate (MgSO_4_) was used as the drying agent after reaction workup, as indicated. Standard three-letter abbreviations are used to represent amino acids.

### General Procedure for Cesium-Promoted Amidation of Unactivated Esters with Amino Alcohol

To a 10 mL flamed-dried, two-necked flask containing a magnetic stir bar were added cesium carbonate (87 mg, 0.27 mmol) and the selected methyl ester derivatives (0.53 mmol), dissolved with anhydrous acetonitrile (0.1 mL) and anhydrous *N,N*-dimethylformamide (0.2 mL). The mixture was stirred at room temperature for 10 min before adding the selected amino alcohol derivatives (1.06 mmol) and anhydrous acetonitrile (0.55 mL) sequentially. The reaction was stirred at room temperature for 24 h. After this time, the reaction was transferred to a separatory funnel with ethyl acetate (50 mL) and 1 M HCl_(aq.)_ (50 mL). The reaction was extracted, and the organic layer was collected. The aqueous layer was further extracted with ethyl acetate (50 mL × 2). The combined organic layer was washed sequentially with deionized water (100 mL) and saturated sodium bicarbonate (100 mL). The organic layer was dried over MgSO_4_, filtered, and concentrated under reduced pressure to obtain a residue which was purified by flash column chromatography to yield **6aa**–**6z**, **8a**–**8h**, **9a**–**9d**, **10a**–**10d**. *Column chromatography was not required for compound **6aa**, **6c**, **6i**, and **6n**. *Aqueous extraction was omitted for compound **9a**–**9c** and **10a**–**10c**.

#### Boc-GlySer-NHHex (**6aa**)

Yellow oil (0.16 g, 87% from 0.53 mmol of **3a** following the general procedure). Reaction procedure with 2.64 mmol of **3a**: To a 10 mL flamed-dried, two-necked flask containing a magnetic stir bar were added cesium carbonate (0.435 g, 1.32 mmol)) and **3a** (0.5 g, 2.64 mmol) which was dissolved with anhydrous acetonitrile (0.5 mL) and anhydrous *N,N*-dimethylformamide (1 mL). The mixture was stirred at room temperature for 10 min before adding the **2a** (1.0 g, 5.31 mmol) and anhydrous acetonitrile (2.75 mL) sequentially. The reaction was stirred at room temperature for 24 h. After this time, the reaction was transferred to a separatory funnel with ethyl acetate and 1 M HCl_(aq.)_. The reaction was extracted, and the organic layer was collected. The aqueous layer was further extracted with ethyl acetate twice. The combined organic layer was washed sequentially with deionized water and saturated sodium bicarbonate. The organic layer was dried over MgSO_4_, filtered, and concentrated under reduced pressure to obtain a residue which was purified by flash column chromatography to yield **6aa** as a yellow oil (0.78 g, 85%). R_*f*_ = 0.4 (MeOH:DCM = 1:19); ^1^H NMR (300 MHz, CDCl_3_): δ 7.23 (br.s, 1H), 6.93 (br.s, 1H), 5.36 (br.s, 1H), 4.44–4.46 (m, 1H), 4.09 (dd, *J* = 11.1, 3.2 Hz, 1H), 3.82 (d, *J* = 5.4 Hz, 2H), 3.65 (dd, *J* = 11.3, 4.7 Hz, 1H), 1.48–1.56 (m, 2H), 1.45 (s, 9H), 1.28 (br.s, 6H), 0.87 (t, *J* = 6.7 Hz, 3H);^13^C{^1^H} NMR (100 MHz, CDCl_3_): δ 170.5, 170.4, 156.5, 80.6, 62.7, 54.5, 44.5, 39.9, 31.5, 29.4, 28.4, 26.6, 22.6, 14.1; IR (ν_max_, film, cm^–1^): 3302, 2961, 2930, 2863, 1646, 1532, 1365, 1297, 1248, 1166, 1051, 944, 646; HRMS (ESI-FT) *m*/*z*: [M + H]^+^ Calcd for C_16_H_32_N_3_O_5_ 346.2337; Found 346.2337.

#### Cbz-GlySer-NHHex (**6ab**)

White solid (0.06 g, 30%); R_*f*_ = 0.3 (MeOH:DCM = 1:19); mp = 142–144 °C; ^1^H NMR (300 MHz, CD_3_OD): δ 7.26–7.36 (m, 5H); 5.12 (s, 2H), 4.39 (t, *J* = 5.0 Hz, 1H), 3.83 (d, *J* = 5.4 Hz, 2H), 3.73–3.80 (m, 2H), 3.17 (t, *J* = 7.0 Hz, 2H), 1.46–1.52 (m, 2H), 1.30 (br.s, 6H), 0.89 (t, *J* = 6.7 Hz, 3H); ^13^C{^1^H} NMR (75 MHz, DMSO-*d*_6_): δ 169.5, 169.0, 156.1, 137.0, 128.3, 127.7, 127.6, 65.4, 61.6, 55.1, 43.6, 38.5, 30.9, 28.9, 25.9, 22.0, 13.9; HRMS (ESI-FT) *m*/*z*: [M + Na]^+^ Calcd for C_19_H_29_N_3_O_5_ 402.1999; Found 402.1997; IR (ν_max_, solid, cm^–1^): 3388, 3319, 3276, 2953, 2925, 2856, 1698, 1659, 1644, 1535, 1497, 1468, 1454, 1416, 1395, 1374, 1333, 1286, 1265, 1224, 1165, 1138, 1092, 1050, 1014, 995, 963, 929, 900, 869, 812, 788, 724, 693, 660, 607, 577, 543, 471; HPLC (IA-3, 2-propanol/*n*-hexane = 10/90, flow rate = 0.7 mL/min, l = 230.4 nm) t_R_ = 39.121 min (area% = 100), e.r. > 99:1.

#### Ac-GlySer-NHHex (**6ad**)

White solid (0.02 g, 14%); R_*f*_ = 0.2 (MeOH:DCM = 1:19); mp = 166–168 °C; ^1^H NMR (300 MHz, DMSO-*d*_6_): δ 8.14 (t, *J* = 5.4 Hz, 1H), 7.87 (d, *J* = 7.5 Hz, 1H), 7.74 (t, *J* = 5.6 Hz, 1H), 4.85 (t, *J* = 5.6 Hz, 1H), 4.17–4.23 (m, 1H), 3.71 (dd, *J* = 8.3, 5.8 Hz, 2H), 3.53–3.57 (m, 2H), 2.99–3.06 (m, 2H), 1.85 (s, 3H), 1.23 (br.s, 8H), 0.85 (t, *J* = 6.8 Hz, 3H); ^13^C{^1^H} NMR (100 MHz, DMSO-*d*_6_): δ 169.3, 169.0, 168.5, 61.1, 54.6, 41.8, 38.1, 30.4, 28.3, 25.4, 21.9, 21.5, 13.4; HRMS (EI) *m*/*z*: Calcd for C_13_H_25_N_3_O_4_ 287.1839; Found 287.1840; IR (ν_max_, solid, cm^–1^): 3280, 2928, 2855, 1626, 1558, 1453, 1372, 1287, 1248, 1151, 1055, 683, 597, 543, 418.

#### (Bn)_2_-GlySer-NHHex (**6ae**)

White solid (0.10 g, 43%); R_*f*_ = 0.7 (MeOH:DCM = 1:19); mp = 74–76 °C; ^1^H NMR (300 MHz, CDCl_3_): δ 8.28 (d, *J* = 7.3 Hz, 1H), 7.26–7.60 (m, 10H), 6.62 (br.s, 1H), 4.21–4.23 (m, 1H), 4.10 (dd, *J* = 10.6, 2.9 Hz, 1H), 3.64 (s, 4H), 3.51 (dd, *J* = 11.2, 4.1 Hz, 1H), 3.13–3.20 (m, 4H), 1.69 (br.s, 1H), 1.38–1.42 (m, 2H), 1.23 (br.s, 6H), 0.86 (t, *J* = 6.6 Hz, 3H); ^13^C{^1^H} NMR (75 MHz, CDCl_3_): δ 172.4, 170.7, 137.8, 129.2, 128.7, 127.7, 62.9, 59.7, 57.3, 53.6, 44.5, 39.6, 31.5, 29.4, 26.6, 22.6, 14.1; HRMS (ESI-FT) *m*/*z*: [M + H]^+^ Calcd for C_25_H_36_N_3_O_3_ 426.2751; Found 426.2751; IR (ν_max_, solid, cm^–1^): 3336, 3279, 3028, 2956, 2925, 2856, 2816, 1677, 1629, 1524, 1494, 1454, 1422, 1373, 1342, 1265, 1249, 1222, 1205, 1153, 1131, 1115, 1078, 1029, 982, 970, 956, 907, 880, 820, 748, 735, 695, 630, 608, 584, 518, 502, 462, 452, 441.

#### Boc-GlyThr-NHHex (**6b**)

Colorless oil (0.14 g, 75%); R_*f*_ = 0.4 (MeOH:DCM = 1:19); ^1^H NMR (300 MHz, CDCl_3_): δ 7.02 (d, *J* = 7.0 Hz, 1H), 6.77 (br.s, 1H), 5.17 (br.s, 1H), 4.37–4.49 (m, 1H), 4.29 (dd, *J* = 7.6, 1.2 Hz, 1H), 3.84 (d, *J* = 5.9 Hz, 2H), 3.15–3.29 (m, 2H), 1.64 (br.s, 1H), 1.40–1.52 (m, 2H), 1.46 (s, 9H), 1.28 (br.s, 6H), 1.14 (d, *J* = 6.5 Hz, 3H), 0.88 (t, *J* = 6.8 Hz, 3H); ^13^C{^1^H} NMR (100 MHz, CDCl_3_): δ 170.7, 170.6, 156.4, 80.6, 66.7, 57.4, 44.2, 39.8, 31.5, 29.4, 28.4, 26.6, 22.6, 18.5, 14.1; HRMS (ESI-FT) *m*/*z*: [M + Na]^+^ Calcd for C_17_H_33_N_3_O_5_Na 382.2312; Found 382.2313; IR (ν_max_, solid, cm^–1^): 3353, 3255, 2976, 2953, 2927, 2857, 1698, 1670, 1640, 1526, 1466, 1425, 1369, 1345, 1288, 1248, 1225, 1169, 1119, 1094, 1067, 1046, 1029, 976, 946, 920, 881, 861, 805, 752, 719, 666, 646, 615, 574.

#### Boc-AlaSer-NHHex (**6c**)

White solid (0.15 g, 77%, with >99:1 dr); R_*f*_ = 0.5 (MeOH:DCM = 1:19); mp = 104–106 °C; ^1^H NMR (300 MHz, CDCl_3_): δ 7.19–7.21 (m, 1H), 6.92 (br.s, 1H), 5.08 (br.s, 1H), 4.42–4.44 (m, 1H,), 4.09–4.18 (m, 2H), 3.64 (dd, *J* = 11.3, 4.3 Hz, 1H), 3.12–3.31 (m, 2H), 1.46–1.51 (m, 2H), 1.44 (s, 9H), 1.38 (d, *J* = 7.1 Hz, 3H), 1.27 (br.s, 6H), 0.87(t, *J* = 6.8 Hz, 3H); ^13^C{^1^H} NMR (100 MHz, CDCl_3_): δ 173.7, 170.4, 156.0, 80.6, 62.6, 54.7, 51.0, 39.8, 31.5, 29.3, 28.3, 26.6, 22.6, 18.2,14.1; IR (ν_max_, solid, cm^–1^): 3299, 2957, 2871, 1691, 1674, 1661, 1632, 1550, 1531, 1451, 1363, 1270, 1248, 1167, 1119, 1030, 903, 761, 671; HRMS (ESI-FT) *m*/*z*: [M + Na]^+^ Calcd for C_17_H_33_N_3_O_5_Na 382.2312; Found 382.2312.

#### Boc-LeuSer-NHHex (**6d**)

Colorless oil (0.13 g, 61%, with >99:1 dr); R_*f*_ = 0.4 (MeOH:DCM = 1:19); ^1^H NMR (300 MHz, CDCl_3_): δ, 7.08–7.10 (m, 1H, -NH), 6.83 (br.s, 1H, -NH), 4.88–4.90 (m, 1H, -NH), 4.38–4.43 (m, 1H), 4.05–4.17 (m, 2H), 3.61–3.65 (m, 1H), 3.13–3.33 (m, 2H), 1.62–1.71 (m, 3H), 1.48–1.55 (m, 3H), 1.44 (s, 9H), 1.28 (br. s, 6H), 0.96 (d, *J* = 3.9 Hz, 3H), 0.94 (d, *J* = 3.7 Hz, 3H), 0.87 (t, *J* = 6.9 Hz, 3H);^13^C{^1^H} NMR (100 MHz, CDCl_3_): δ 173.5, 170.5, 156.1, 80.6, 62.6, 54.4, 53.9, 41.2, 39.8, 31.5, 29.3, 28.3, 26.6, 24.9, 23.1, 22.6, 21.8, 14.1; IR (ν_max_, film, cm^–1^): 3289, 3241, 2957, 2930, 2870, 1689, 1639, 1520, 1468, 1450, 1392, 1366, 1280, 1252, 1165, 1046, 1023, 660; HRMS (ESI-FT) *m*/*z*: [M + Na]^+^ Calcd for C_20_H_39_N_3_O_5_Na 424.2782; Found 424.2782.

#### Boc-ValSer-NHHex (**6e**)

White solid (0.09 g, 44%, with >99:1 dr); R_*f*_ = 0.4 (MeOH:DCM = 1:19); mp = 120–122 °C; ^1^H NMR (300 MHz, CDCl_3_): δ 7.12–7.14 (m, 1H), 6.85 (br.s, 1H), 5.04–5.06 (m, 1H), 4.42–4.44 (m, 1H), 4.13 (dd, *J* = 11.2, 2.8 Hz, 1H), 3.95–3.99 (m, 1H), 3.63 (dd, *J* = 11.5, 4.5 Hz, 1H), 3.12–3.29 (m, 2H), 2.11–2.22 (m, 1H), 1.47–1.52 (m, 2H), 1.44 (s, 9H), 1.28 (br.s, 6H), 0.98 (d, *J* = 6.8 Hz, 3H), 0.92 (d, *J* = 6.9 Hz, 3H), 0.87 (t, *J* = 6.7 Hz, 3H); ^13^C{^1^H} NMR (100 MHz, CDCl_3_): δ 172.4, 170.6, 156.3, 80.6, 62.7, 60.4, 53.9, 39.7, 31.5, 30.6, 29.3, 28.4, 26.6, 22.6, 19.4, 17.7, 14.1; IR (ν_max_, solid, cm^–1^): 3289, 2931, 2961, 2874, 1640, 1520, 1461, 1366, 1295, 1246, 1166, 1046, 657; HRMS (ESI-FT) *m*/*z*: [M + Na]^+^ Calcd for C_19_H_37_N_3_O_5_Na 410.2625; Found 410.2622.

#### Boc-IleSer-NHHex (**6f**)

White solid (0.07 g, 34%, with >99:1 dr); R_*f*_ = 0.4 (MeOH:DCM = 1:19); mp = 86–88 °C; ^1^H NMR (300 MHz, CDCl_3_): δ 7.20–7.22 (m, 1H), 6.92 (br.s, 1H), 5.02 (br.s, 1H), 4.41–4.44 (m, 1H), 4.09–4.13 (m, 2H), 3.64 (dd, *J* = 11.4, 4.8 Hz, 1H), 3.12–3.31 (m, 2H), 1.60–1.74 (m, 2H), 1.46–1.52 (m, 3H), 1.43 (s, 9H), 1.28 (br. s, 6H), 0.93–0.96 (m, 6H), 0.87 (t, *J* = 6.9 Hz, 3H); ^13^C{^1^H} NMR (100 MHz, CDCl_3_): δ 172.4, 170.5, 156.2, 80.7, 62.7, 59.8, 54.2, 39.8, 37.2, 31.6, 29.4, 28.4, 26.7, 24.9, 22.6, 15.8, 14.1, 11.7; IR (ν_max_, solid, cm^–1^): 3297, 3065, 2958, 2929, 2860, 1693, 1651, 1538, 1466, 1450, 1391, 1363, 1296, 1271, 1236, 1194, 1158, 1084, 1023, 879, 756, 736, 671, 621, 595, 547, 461, 426; HRMS (ESI-FT) *m*/*z*: [M + Na]^+^ Calcd for C_20_H_39_N_3_O_5_Na 424.2782; Found 424.2785.

#### Boc-β-AlaSer-NHHex (**6g**)

White solid (0.14 g, 72%); R_*f*_ = 0.3 (MeOH:DCM = 1:19); mp = 102–104 °C; ^1^H NMR (400 MHz, CDCl_3_): δ 6.92 (d, *J* = 7.2 Hz, 1H), 6.86 (t, *J* = 4.8 Hz, 1H), 5.13 (br.s, 1H), 4.39–4.43 (m, 1H), 4.06 (dd, *J* = 11.4, 3.7 Hz, 1H), 3.63 (dd, *J* = 11.2, 5.0 Hz, 1H), 3.42 (td, *J* = 5.9, 5.6 Hz, 2H), 3.20–3.25 (m, 2H), 2.47 (td, *J* = 5.9, 2.1 Hz, 2H), 1.85 (br.s, 1H), 1.46–1.51 (m, 2H), 1.43 (s, 9H), 1.25–1.33 (m, 6H), 0.88 (t, *J* = 6.9 Hz, 3H); ^13^C{^1^H} NMR (75 MHz, CDCl_3_): δ 172.6, 170.7, 156.3, 79.7, 62.9, 54.5, 39.8, 37.0, 36.5, 29.4, 28.5, 26.7, 22.6, 14.1; HRMS (ESI-FT) *m*/*z*: [M + Na]^+^ Calcd for C_17_H_33_N_3_O_5_Na 382.2312; Found 382.2311; IR (ν_max_, solid, cm^–1^): 3336, 3281, 2928, 2856, 1683, 1635, 1527, 1454, 1392, 1367, 1285, 1253, 1235, 1167, 1072, 1034, 976, 868, 722, 651, 551, 461, 433.

#### Boc-(DL-BABA)Ser-NHHex (**6h**)

Isolated as a mixture of 2 inseparable diastereomers. White solid (0.12 g, 61%); R_*f*_ = 0.4 (MeOH:DCM = 1:19); mp = 142–144 °C; ^1^H NMR (400 MHz, CDCl_3_): δ 6.76–6.96 (m, 2H), 5.02–5.18 (m, 1H), 4.38–4.45 (m, 1H), 4.01–4.07 (m, 2H), 3.63 (br.s, 2H), 3.19–3.26 (m, 2H), 2.40–2.45 (m, 2H), 1.47–1.49 (m, 2H), 1.43 (s, 9H), 1.28 (br.s, 6H), 1.22 (d, *J* = 7.1 Hz, 3H), 0.88 (t, *J* = 6.7 Hz, 3H); ^13^C{^1^H} NMR (75 MHz, CDCl_3_): δ 172.0 (*epi*), 171.9, 170.9 (*epi*), 170.8, 155.7, 79.7, 63.0 (*epi*), 62.8, 54.4 (*epi*), 54.2, 43.5 (*epi*), 43.3, 39.8, 31.5, 29.8, 29.4, 28.5, 26.7, 22.6, 21.2, 14.1; HRMS (ESI-FT) *m*/*z*: [M + Na]^+^ Calcd for C_18_H_35_N_3_O_5_Na 396.2469; Found 396.2467; IR (ν_max_, solid, cm^–1^): 3286, 2960, 2929, 2857, 1681, 1632, 1523, 1454, 1391, 1366, 1342, 1313, 1270, 1251, 1162, 1111, 1060, 964, 906, 886, 855, 781, 722, 676, 621, 557, 516, 461, 433.

#### Boc-PheSer-NHHex (**6i**)

White solid (0.18 g, 78%, with >99:1 dr); R_*f*_ = 0.6 (MeOH:DCM = 1:19); mp = 104–106 °C; ^1^H NMR (300 MHz, CDCl_3_): δ 7.18–7.41 (m, 6H), 7.01–7.04 (m, 1H), 6.71–6.75 (m, 1H), 5.06 (br.s, 1H), 4.36–4.42 (m, 2H), 4.07 (dd, *J* = 11.4, 3.0 Hz, 1H), 3.56 (dd, *J* = 11.4, 4.4 Hz, 1H), 2.98–3.29 (m, 4H), 1.42–1.49 (m, 2H), 1.39 (s, 9H), 1.28 (br. s, 6H), 0.88 (t, *J* = 6.9 Hz, 3H); ^13^C{^1^H} NMR (100 MHz, CDCl_3_): δ 174.5, 172.0, 158.1, 138.6, 130.5, 129.6, 127.9, 81.1, 63.0, 58.0, 56.8, 40.7, 38.8, 32.8, 30.4, 28.8, 27.7, 23.7, 14.5; IR (ν_max_, solid, cm^–1^): 3239, 3030, 2928, 2859, 1880, 1692, 1638, 1455, 1366, 1250, 1225, 1166, 1049, 1026, 723, 699; HRMS (ESI-FT) *m*/*z*: [M + Na]^+^ Calcd for C_23_H_37_N_3_O_5_Na 458.2625; Found 458.2624. HPLC (IA-3, 2-propanol/*n*-hexane = 10/90, flow rate = 0.7 mL/min, l = 230.4 nm) t_R_ = 27.553 min (area% = 100), d.r. > 99:1.

#### Boc-MetSer-NHHex (**6j**)

White solid (0.15 g, 68%, with >99:1 dr); R_*f*_ = 0.3 (MeOH:DCM = 1:19); mp = 104–106 °C; ^1^H NMR (300 MHz, CDCl_3_): δ 7.24 (br.s, 1H), 6.31 (br.s, 1H), 5.31–5.33 (m, 1H), 4.40–4.44 (m, 1H), 4.23–4.39 (m, 1H), 4.12 (dd, *J* = 11.7, 3.5 Hz, 1H), 3.65 (dd, *J* = 11.3, 4.4 Hz, 1H), 3.16–3.29 (m, 2H), 2.57 (t, *J* = 6.8 Hz, 2H), 2.07–2.18 (m, 4H), 1.88–2.00 (m, 1H), 1.72 (br.s, 1H), 1.48–1.52 (m, 2H), 1.44 (s, 9H), 1.38 (br.s, 6H), 0.88 (t, *J* = 7.4 Hz, 3H); ^13^C{^1^H} NMR (100 MHz, CDCl_3_): δ 172.6, 170.3, 156.1, 80.5, 62.7, 54.7, 54.4, 39.8, 31.7, 31.5, 30.3, 29.3, 26.6, 22.6, 18.4, 15.4, 14.1; IR (ν_max_, solid, cm^–1^): 3302, 2959, 2927, 2857, 1686, 1641, 1524, 1442, 1392, 1366, 1283, 1253, 1233, 1169, 1044, 1024, 972, 862, 781, 691, 660, 456; HRMS (ESI-FT) *m*/*z*: [M + Na]^+^ Calcd for C_19_H_37_N_3_O_5_SNa 442.2346; Found 442.2344.

#### Boc-ProSer-NHHex (**6k**)

Yellow oil (0.07 g, 33%, with 92:8 dr); R_*f*_ = 0.4 (MeOH:DCM = 1:19); ^1^H NMR (300 MHz, CDCl_3_): δ 7.10–7.17 (m, 1H), 4.42–4.49 (m, 1H), 4.22–4.26 (m, 1H), 4.07–4.16 (m, 1H), 3.56–3.72 (m, 1H), 3.35–3.53 (m, 2H), 3.10–3.34 (m, 2H), 2.00–2.24 (m, 2H), 1.86–1.91 (m, 2H), 1.86–1.91 (m, 2H), 1.48–1.54 (m, 2H), 1.45 (s, 9H), 1.27 (br.s, 6H), 0.86 (t, *J* = 6.9 Hz, 3H); ^13^C{^1^H} NMR (100 MHz, CDCl_3_): δ 172.7, 170.5, 155.9, 81.8, 62.8, 61.1, 54.5, 47.5, 39.9, 31.6, 29.8, 29.3, 28.4, 26.9, 24.7, 22.6, 14.1; IR (ν_max_, film, cm^–1^): 3281, 2956, 2929, 2859, 1641, 1536, 1451, 1393, 1365, 1296, 1240, 1161, 1131, 1077, 1053, 922, 882, 773, 735, 543, 425; HRMS (ESI-FT) *m*/*z*: [M + Na]^+^ Calcd for C_19_H_35_N_3_O_5_Na 408.2469; Found 408.2467.

#### Boc-Cys(^*t*^Bu)Ser-NHHex (**6l**)

Colorless oil (0.17 g, 73%, with >99:1 dr); R_*f*_ = 0.5 (MeOH:DCM = 1:19); ^1^H NMR (300 MHz, CDCl_3_): δ 7.22 (d, *J* = 7.6 Hz, 1H), 6.95 (br.s, 1H), 5.42 (d, *J* = 6.5 Hz, 1H), 4.44–4.47 (m, 1H), 4.23–4.31 (m, 1H), 4.15 (dd, *J* = 11.2, 3.7 Hz, 1H), 3.66 (dd, *J* = 11.2, 4.8 Hz, 1H), 3.14–3.29 (m, 2H), 2.83–3.05 (m, 2H), 1.91 (br.s, 1H), 1.47–1.52 (m, 2H), 1.44 (s, 9H), 1.33 (s, 9H), 1.27 (br.s, 6H), 0.87 (t, *J* = 6.8 Hz, 3H); ^13^C{^1^H} NMR (100 MHz, CDCl_3_): δ 171.2 170.2, 156.9, 81.0, 62.6, 54.9, 54.5, 43.3, 39.7, 31.7, 31.0, 30.6, 29.4, 28.4, 26.5, 22.6, 14.1; IR (ν_max_, solid, cm^–1^): 3301, 2958, 2929, 2860, 1645, 1529, 1460, 1392, 1366, 1250, 1162, 1050, 1023, 868, 588; HRMS (ESI-FT) *m*/*z*: [M + Na]^+^ Calcd for C_21_H_41_N_3_O_5_SNa 470.2659; Found 470.2662.

#### Boc-Gln(Trt)Ser-NHHex (**6m**)

White solid (0.29 g, 83%, with >99:1 dr); R_*f*_ = 0.4 (MeOH:DCM = 1:19); mp = 100–102 °C; ^1^H NMR (300 MHz, CDCl_3_): δ 7.24–7.29 (m, 9H), 7.18–7.21 (m, 6H), 6.96–7.01 (m, 2H), 5.86 (br.s, 1H), 4.36–4.42 (m, 1H), 3.97–4.01 (m, 2H), 3.60 (dd, *J* = 11.1, 4.7 Hz, 1H), 3.30–3.21 (m, 2H), 2.45–2.50 (m, 1H), 1.95–2.11 (m, 3H), 1.32–1.44 (m, 11H), 1.34 (br.s, 6H), 0.86 (t, *J* = 6.7 Hz, 3H); ^13^C{^1^H} NMR (100 MHz, CDCl_3_): δ 172.5, 172.1, 170.2, 156.4, 144.5, 128.7, 128.0, 127.0, 80.4, 70.7, 62.4, 55.1, 54.8, 39.8, 33.3, 31.5, 29.1, 28.4, 27.4, 26.6, 22.6, 14.1; IR (ν_max_, solid, cm^–1^): 3300, 2929, 2858, 1651, 1492, 1392, 1366, 1249, 1163, 1051, 602, 860, 751, 698, 624, 569; HRMS (ESI-FT) *m*/*z*: [M + Na]^+^ Calcd for C_38_H_50_N_4_O_6_Na 681.3623; Found 681.3624.

#### Boc-Asn(Trt)Ser-NHHex (**6n**)

Yellow solid (0.31 g, 90%, with >99:1 dr); R_*f*_ = 0.5 (MeOH:DCM = 1:19); mp = 168–170 °C; ^1^H NMR (300 MHz, CD_3_OD): δ 7.21–7.27 (m, 15H), 4.30–4.37 (m, 2H), 3.85 (dd, *J* = 10.9, 5.1 Hz, 1H), 3.73 (dd, *J* = 11.0, 4.1 Hz, 1H), 2.76–2.89 (m, 3H), 1.45 (s, 9H), 1.18–1.31 (m, 8H), 0.88 (t, *J* = 7.14 Hz, 3H); ^13^C{^1^H} NMR (100 MHz, CD_3_OD): δ 173.8, 171.7, 171.6, 157.5, 145.8, 130.0, 128.7, 127.8, 81.1, 71.6, 62.7, 56.8, 53.0, 40.5, 39.3, 32.6, 30.0, 28.7, 27.5, 23.6, 14.4; IR (ν_max_, solid, cm^–1^): 3301, 3059, 2929, 2857, 1778, 1645, 1520, 1492, 1447, 1367, 1252, 1165, 1049, 752, 698, 636, 624, 564, 507; HRMS (ESI-FT) *m*/*z*: [M + Na]^+^ Calcd for C_37_H_48_N_4_O_6_Na 667.3466; Found 667.3468.

#### Boc-TrpSer-NHHex (**6o**)

Isolated as a mixture of 2 inseparable diastereomers. Yellow solid (0.15 g, 61%, with 76:24 dr); R_*f*_ = 0.4 (MeOH:DCM = 1:19); mp = 76–78 °C; ^1^H NMR (300 MHz, CD_3_OD): δ 7.58 (d, *J* = 7.7 Hz, 1H), 7.34 (d, *J* = 8.0 Hz, 1H), 7.16 (s, 1H), 7.10 (t, *J* = 6.7 Hz, 1H), 7.01 (t, *J* = 4.1 Hz, 1H), 4.31–4.35 (m, 1H), 4.21–4.25 (m, 1H), 3.74 (dd, *J* = 11.0, 4.7 Hz, 1H), 3.46 (dd, *J* = 11.3, 4.8 Hz, 1H), 3.24 (d, *J* = 5.7 Hz, 1H), 3.04–3.18 (m, 3H), 1.43–1.49 (m, 2H), 1.39 (s, 9H), 1.29 (br.s, 6H), 0.90 (t, *J* = 6.8 Hz, 3H); ^13^C{^1^H} NMR (100 MHz, CD_3_OD): δ 174.9, 171.8, 158.2, 138.0, 128.7, 124.8, 122.5, 119.8, 119.3, 112.3, 110.6, 81.0, 62.5, 57.4, 56.7, 40.6, 32.6, 30.7, 30.2, 28.6, 27.5, 23.6, 14.4; IR (ν_max_, solid, cm^–1^): 3301, 3060, 2956, 2927, 2856, 1644, 1505, 1457, 1392, 1366, 1276, 1250, 1162, 1057, 1011, 856, 740, 660, 555, 460, 425; HRMS (ESI-FT) *m*/*z*: [M + Na]^+^ Calcd for C_25_H_38_N_4_O_5_Na 497.2734; Found 497.2737.

#### Boc-SerSer-NHHex (**6p**)

Isolated as a mixture of 2 inseparable diastereomers. White solid (0.08 g, 39%, with 77:23 dr); R_*f*_ = 0.4 (MeOH:DCM = 1:19); mp = 132–134 °C; ^1^H NMR (300 MHz, CD_3_OD): δ 4.37 (d, *J* = 4.5 Hz, 1H), 4.14 (d, *J* = 5.6 Hz, 1H), 3.89 (dd, *J* = 11.1, 4.3 Hz, 1H), 3.83 (dd, *J* = 11.0, 5.7 Hz, 1H), 3.78 (dd, *J* = 11.1, 4.1 Hz, 1H), 3.71 (dd, *J* = 11.3, 6.0 Hz, 1H), 3.10–3.26 (m, 2H), 1.48–1.56 (m, 2H), 1.46 (s, 9H), 1.31 (br.s, 6H), 0.90 (d, *J* = 6.7 Hz, 3H); ^13^C{^1^H} NMR (75 MHz, CD_3_OD): δ 173.4, 171.9, 158.1, 81.1, 63.1, 62.7, 58.3, 57.0, 40.6, 32.6, 30.1, 28.7, 27.5, 23.6, 14.4; HRMS (ESI-FT) *m*/*z*: [M + Na]^+^ Calcd for C_17_H_33_N_3_O_6_Na 398.2262; Found 398.2260; IR (ν_max_, solid, cm^–1^): 3433, 3289, 2929, 2859, 1721, 1702, 1673, 1657, 1634, 1523, 1493, 1388, 1364, 1299, 1235, 1166, 1087, 1058, 1008, 935, 857, 739, 662, 574, 525.

#### Boc-ThrSer-NHHex (**6q**)

Isolated as a mixture of 2 inseparable diastereomers. White solid (0.07 g, 34%, with 88:12 dr); R_*f*_ = 0.4 (MeOH:DCM = 1:19); mp = 132–134 °C; ^1^H NMR (300 MHz, CDCl_3_): δ 7.51 (d, *J* = 8.1 Hz, 1H), 7.04 (br.s, 1H), 5.66 (d, *J* = 7.6 Hz, 1H), 4.51 (dt, *J* = 8.3, 4.4 Hz, 1H), 4.31 (qd, *J* = 10.7, 2.9 Hz, 1H), 4.20 (d, *J* = 8.4 Hz, 1H), 4.02 (dd, *J* = 9.5, 3.5 Hz, 1H), 3.86 (br.s, 1H), 3.72 (dd, *J* = 10.7, 4.3 Hz, 1H), 3.11–3.31 (m, 2H), 1.81 (br.s, 1H), 1.49–1.51 (m, 2H), 1.45 (s, 9H), 1.27 (br.s, 6H), 1.20 (d, *J* = 6.5 Hz, 3H), 0.87 (t, *J* = 6.7 Hz, 3H); ^13^C{^1^H} NMR (75 MHz, CDCl_3_): δ 14.1, 18.6, 22.6, 26.6, 28.4, 29.3, 31.5, 40.0, 55.2, 59.5, 62.6, 67.4, 80.6, 156.4, 170.3, 171.5; HRMS (ESI-FT) *m*/*z*: [M + Na]^+^ Calcd for C_18_H_35_N_3_O_6_Na 412.2418; Found 412.2420; IR (ν_max_, solid, cm^–1^): 3287, 2960, 2931, 2861, 1721, 1691, 1660, 1628, 1543, 1486, 1390, 1365, 1303, 1217, 1166, 1133, 1099, 1064, 881, 730, 678, 616, 597, 513, 460.

#### Boc-TyrSer-NHHex (**6r**)

Yellow solid (0.10 g, 43%, with >99:1 dr); R_*f*_ = 0.3 (MeOH:DCM = 1:19); mp = 136–138 °C; ^1^H NMR (400 MHz, CD_3_OD): δ 7.06 (d, *J* = 11.2 Hz, 2H), 6.71 (d, *J* = 8.4 Hz, 2H), 4.30–4.32 (m, 1H), 4.20–4.24 (m, 1H), 3.80 (dd, *J* = 10.8, 4.8 Hz, 1H), 3.70 (dd, *J* = 10.8, 4.8 Hz, 1H), 3.16 (td, *J* = 4.0, 3.2 Hz, 2H), 3.01 (dd, *J* = 14.4, 6.0 Hz, 1H), 2.79 (dd, *J* = 13.6, 8.8 Hz, 1H), 1.40 (s, 9H), 1.44–1.55(m, 2H), 1.29–1.36 (m, 6H), 0.94 (t, *J* = 6.8 Hz, 3H); ^13^C{^1^H} NMR (100 MHz, CD_3_OD): δ 174.6, 171.8, 158.0, 157.4, 131.3, 128.9, 116.2, 81.0, 62.8, 58.1, 56.6, 40.6, 37.7, 32.5, 30.3, 28.7, 27.6, 23.6, 14.2; IR (ν_max_, solid, cm^–1^): 3318, 2957, 2930, 2859, 1689, 1633, 1515, 1455, 1392, 1366, 1248, 1165, 1049, 930, 883, 826, 666, 593, 543, 514, 491; HRMS (ESI-FT) *m*/*z*: [M + Na]+ Calcd for C_23_H_37_N_3_O_6_Na 474.2575; Found 474.2576.

#### Boc-Ser(^*t*^Bu)Ser-NHHex (**6s**)

Colorless oil (0.13 g, 55%, with >99:1 dr); R_*f*_ = 0.5 (MeOH:DCM = 1:19); ^1^H NMR (300 MHz, CDCl_3_): δ 7.34–7.39 (m, 1H), 6.78–6.86 (m, 1H), 5.38 (br.s, 1H), 4.39–4.44 (m, 1H), 4.13–4.19 (m, 2H), 3.76 (dd, *J* = 8.8, 4.1 Hz, 1H), 3.58–3.66 (m, 1H), 3.45 (dd, *J* = 8.9, 5.9 Hz, 1H), 3.10–3.32 (m, 3H), 1.49–1.51 (m, 2H), 1.45 (s, 9H), 1.28 (br.s, 6H), 1.18 (s, 9H), 0.87 (t, *J* = 6.8 Hz, 3H); ^13^C{^1^H} NMR (75 MHz, CDCl_3_): δ 171.3, 170.5, 155.9, 80.6, 74.2, 62.7, 61.8, 55.4, 54.4, 39.8, 31.5, 29.4, 28.4, 27.5, 26.6, 22.6, 14.1; HRMS (ESI-FT) *m*/*z*: [M + Na]^+^ Calcd for C_21_H_41_N_3_O_6_Na 454.2888; Found 454.2885; IR (ν_max_, solid, cm^–1^): 3300, 2972, 2931, 2872, 1689, 1641, 1524, 1392, 1364, 1239, 1165, 1074, 1055, 1020, 882, 763, 780, 652, 546, 461.

#### Boc-Tyr(^*t*^Bu)Ser-NHHex (**6t**)

Yellow solid (0.21 g, 79%, with >99:1 dr); R_*f*_ = 0.3 (MeOH:DCM = 1:19); mp = 72–74 °C; ^1^H NMR (300 MHz, CDCl_3_): δ 7.08 (d, *J* = 8.1 Hz, 2H), 6.97 (d, *J* = 7.8 Hz, 1H), 6.93 (d, *J* = 8.2 Hz, 2H), 6.74 (t, *J* = 5.6 Hz, 1H), 5.47 (d, *J* = 5.5 Hz, 1H), 4.28–4.41 (m, 2H), 4.07 (dd, *J* = 10.6, 3.5 Hz, 1H), 3.55 (dd, *J* = 11.1, 5.9 Hz, 1H), 2.92–3.30 (m, 4H), 1.82 (br.s, 1H), 1.43–1.53 (m, 2H), 1.40 (s, 9H, (H1)), 1.32 (s, 9H), 1.28 (br.s, 6H), 0.87 (t, *J* = 6.7 Hz, 3H); ^13^C{^1^H} NMR (75 MHz, CDCl_3_): δ 172.3, 170.2, 155.9, 154.6, 131.0, 129.7, 124.4, 80.7, 78.6, 62.7, 56.4, 54.5, 39.8, 37.6, 31.5, 29.4, 28.9, 28.3, 26.6, 22.6, 14.1; HRMS (ESI-FT) *m*/*z*: [M + Na]+ Calcd for C_27_H_45_N_3_O_6_Na 530.3201; Found 530.3202; IR (ν_max_, solid, cm^–1^): 3290, 2930, 2860, 1691, 1639, 1506, 1390, 1365, 1235, 1161, 1051, 1024, 924, 899, 848, 758, 667, 570, 461.

#### Boc-PhgSer-NHHex (**6u**)

Isolated as a mixture of 2 inseparable diastereomers. Colorless solid (0.19 g, 83%, with 52:48 dr); R_*f*_ = 0.5 (MeOH:DCM = 1:19); mp = 90–92 °C; ^1^H NMR (300 MHz, CDCl_3_): δ 7.36 (br.s, 5H), 7.17 (br.s, 1H), 6.60–6.97 (m, 1H), 5.56–5.62 (m, 1H), 5.12–5.19 (m, 1H), 4.42 (d, *J* = 3.4 Hz, 1H), 4.05–4.16 (m, 1H), 3.49–3.66 (m, 1H), 3.32 (br.s, 1H), 3.02–3.24 (m, 2H), 1.49–1.51 (m, 2H), 1.43 (s, 9H), 1.24–1.27 (m, 6H), 0.87 (t, *J* = 6.4 Hz, 3H); ^13^C{^1^H} NMR (75 MHz, CDCl_3_): δ 171.5 (epi), 171.1, 170.4 (epi), 170.2, 155.7, 129.2, 129.1, 128.7, 127.3, 127.2, 80.8, 62.6, 54.6, 54.5, 39.9, 39.8, 31.5, 29.4, 29.2, 28.4, 26.6, 26.5, 22.6, 14.1; HRMS (ESI-FT) *m*/*z*: [M + Na]^+^ Calcd for C_22_H_35_N_3_O_5_Na 444.2469; Found 444.2467; IR (ν_max_, solid, cm^–1^): 3299, 2958, 2930, 2858, 1687, 1641, 1518, 1364, 1248, 1169, 1048, 884, 735, 695; HPLC (IA-3, 2-propanol/*n*-hexane = 10/90, flow rate = 0.7 mL/min, l = 230.4 nm) t_R_ = 26.247 min (major, area% = 50.61), 35.813 min (minor, area% = 49.39) d.r. = 52:48.

#### Boc-HseSer-NHHex (**6v**)

Isolated as a mixture of 2 inseparable diastereomers. Colorless oil (0.07 g, 33%, with 55:45 dr); R_*f*_ = 0.3 (MeOH:DCM = 1:19); ^1^H NMR (300 MHz, CDCl_3_): δ 7.56 (t, *J* = 7.0 Hz, 1H, -NH), 7.02–7.09 (m, 1H, -NH), 5.69–5.82 (m, 1H, -NH), 4.48–4.51 (m, 1H), 4.34 (br.s, 1H), 4.03 (dd, *J* = 10.4, 5.3 Hz, 1H), 3.69–3.76 (m, 3H), 3.13–3.28 (m, 2H), 1.87–2.04 (m, 3H), 1.43 (s, 9H), 1.27 (br.s, 8H), 0.87 (t, *J* = 6.5 Hz, 3H); ^13^C{^1^H} NMR (75 MHz, CDCl_3_): δ 173.5, 173.2 (epi), 170.5, 170.4 (epi), 156.5, 80.5, 62.5, 59.0, 58.6, 55.1, 53.1, 52.6, 39.9, 35.2, 31.5, 29.3, 28.4, 26.6, 22.6, 14.1; HRMS (ESI-FT) *m*/*z*: [M + Na]^+^ Calcd for C_18_H_35_N_3_O_6_Na 412.2418; Found 412.2418; IR (ν_max_, solid, cm^–1^): 3300, 2956, 2930, 2859, 1645, 1525, 1455, 1392, 1366, 1278, 1249, 1165, 1054, 906, 865, 781, 756, 599.

#### (*S*)-2-Acetamido-3-(hexylamino)-3-oxopropyl (*tert*-butoxycarbonyl)glycinate (**6w**)

Colorless solid (0.03 g, 14%); R_*f*_ = 0.5 (MeOH:DCM = 1:19); mp = 62–64 °C; ^1^H NMR (300 MHz, CDCl_3_): δ 5.57–6.62 (m, 2H, −OH, -NH), 5.12 (br.s, 1H, -NH), 4.64–4.70 (m, 1H), 4.40 (d, *J* = 5.4 Hz, 2H), 3.84–3.89 (m, 2H), 3.20–3.27 (m, 2H), 2.04 (s, 3H), 1.49–1.51 (m, 2H), 1.44 (s, 9H), 1.28 (br.s, 6H), 0.88 (t, *J* = 6.7 Hz, 3H); ^13^C{^1^H} NMR (100 MHz, CDCl_3_): δ 170.8, 170.4, 168.7, 156.2, 80.4, 64.4, 52.0, 42.7, 39.9, 31.5, 29.4, 28.4, 26.6, 23.2, 22.6, 14.1; HRMS (ESI-FT) *m*/*z*: [M + Na]^+^ Calcd for C_18_H_33_N_3_O_6_Na 410.2262; Found 410.2263; IR (ν_max_, film, cm^–1^): 3290, 2930, 2861, 1760, 1645, 1526, 1452, 1367, 1293, 1257, 1160, 1056, 952, 865, 729.

#### Boc-AlaGlySer-NHHex (**6ya**)

White solid (0.14 g, 62% from **5u** and **2a**, 0.06 g, 25% from **5a** and **H-GlySer-NHHex**); R_*f*_ = 0.5 (MeOH:DCM = 1:19); mp = 130–132 °C; ^1^H NMR (400 MHz, CDCl_3_): δ 7.44–7.46 (m, 2H, -NH), 6.94–6.97 (m, 1H, -NH), 5.32–5.33 (m, 1H, -NH), 4.48–4.51 (m, 1H), 4.18–4.21 (m, 1H), 4.00–4.01 (m, 2H), 3.73 (dd, *J* = 10.4, 5.2 Hz, 1H), 3.18–3.25 (m, 1H), 1.83 (br.s, 1H, −OH), 1.47–1.52 (m, 2H), 1.43 (s, 9H), 1.38 (d, *J* = 6.8 Hz, 3H), 1.25–1.30 (m, 6H), 0.87 (t, *J* = 7.2 Hz, 3H); ^13^C{^1^H} NMR (100 MHz, CDCl_3_): δ 174.3, 170.4, 169.7, 156.1, 80.6, 62.8, 55.0, 50.1, 43.4, 39.9, 31.8, 29.4, 28.5, 26.7, 22.7, 18.7, 14.1; IR (ν_max_, solid, cm^–1^): 3327, 3291, 3070, 2963, 2932, 2859, 1680, 1657, 1640, 1524, 1446, 1392, 1366, 1321, 1268, 1240, 1216, 1165, 1069, 1048, 856, 639, 565, 549, 424; HRMS (ESI-FT) *m*/*z*: [M + Na]^+^ Calcd for C_19_H_36_N_4_O_6_Na 439.2527; Found 439.2528.

#### Boc-GlySerAla-NHHex (**6yb**)

White solid (0.13 g, 58%); R_*f*_ = 0.5 (MeOH:DCM = 1:19); mp = 166–168 °C; ^1^H NMR (300 MHz, CD_3_OD): δ 4.31–4.39 (m, 2H), 3.88 (dd, *J* = 10.8, 4.9 Hz, 1H), 3.70–3.76 (m, 3H), 3.15 (td, *J* = 13.0, 6.8 Hz, 2H), 1.47–1.52 (m, 2H), 1.45 (s, 9H), 1.39 (d, *J* = 7.2 Hz, 3H), 1.29–1.34 (m, 6H), 0.90 (t, *J* = 6.9 Hz, 3H); ^13^C{^1^H} NMR (100 MHz, CD_3_OD): 174.9, 173.3, 172.4, 158.9, 81.2, 62.9, 57.1, 50.9, 45.1, 40.7, 32.9, 30.4, 28.9, 27.8, 23.8, 18.0, 14.6 ; IR (ν_max_, solid, cm^–1^): 3312, 3275, 3073, 2930, 2872, 2858, 1656, 1626, 1581, 1526, 1502, 1454, 1412, 1393, 1367, 1344, 1234, 1166, 1128, 1070, 1046, 1032, 1004, 991, 848, 704, 660, 555, 462.; HRMS (ESI-FT) *m*/*z*: [M + Na]^+^ Calcd for C_19_H_36_N_4_O_6_Na 439.2527; Found 439.2530.

#### Boc-AlaGlySerAla-NHHex (**6z**)

White solid (0.12 g, 47%); R_*f*_ = 0.5 (MeOH:DCM = 1:9); mp = 184–186 °C; ^1^H NMR (400 MHz, CD_3_OD): δ 4.37–4.40 (m, 1H), 4.31–4.35 (m, 1H), 4.02–4.08 (m, 1H), 3.86–3.94 (m, 3H), 3.78 (dd, *J* = 11.2, 5.6 Hz, 1H), 3.09–3.22 (m, 2H), 1.47–1.53 (m, 2H), 1.45 (s, 9H), 1.39 (d, *J* = 7.3 Hz, 3H), 1.34 (d, J = 3.2 Hz, 3H), 1.31 (br.s, 6H), 0.90 (t, *J* = 6.4 Hz, 3H); ^13^C{^1^H} NMR (100 MHz, CD_3_OD): 176.8, 174.7, 172.2, 158.0, 80.9, 62.8, 57.1, 52.1, 50.8, 43.9, 40.5, 32.6, 30.2, 28.7, 27.6, 23.6, 18.0, 17.8, 14.4 (missing one C=O); IR (ν_max_, solid, cm^–1^): 3280, 3095, 2958, 2929, 2858, 1697, 1626, 1531, 1489, 1391, 1365, 1247, 1164, 1069, 1051, 1028, 862, 777, 697, 624, 563, 499; HRMS (ESI-FT) *m*/*z*: [M + Na]^+^ Calcd for C_22_H_41_N_5_O_7_Na 510.2898; Found 510.2899.

#### (*S*)-*N*-(1-(Hexylamino)-3-hydroxy-1-oxopropan-2-yl)-4-nitrobenzamide (**8a**)

White solid (0.13 g, 75%); R_*f*_ = 0.5 (MeOH:DCM = 1:19); mp = 114–116 °C; ^1^H NMR (300 MHz, CDCl_3_): δ 8.29 (d, *J* = 8.6 Hz, 2H), 8.00 (d, *J* = 14.7 Hz, 2H), 7.65–7.67 (m, 1H, -NH), 6.93 (br.s, 1H, -NH), 4.63–4.65 (m, 1H), 4.21 (dd, *J* = 11.5, 3.0 Hz, 1H), 3.77 (dd, *J* = 11.3, 5.1 Hz, 1H), 3.28 (td, *J* = 6.8, 6.4 Hz, 2H), 1.47–1.54 (m, 2H), 1.27 (br.s, 6H), 0.85 (t, *J* = 6.8 Hz, 3H); ^13^C{^1^H} NMR (100 MHz, CDCl_3_): δ 170.4, 166.1, 150.0, 138.8, 128.5, 124.0, 62.9, 54.5, 31.5, 29.4, 26.6, 22.6, 14.1; IR (ν_max_, solid, cm^–1^): 3690, 3274, 3090, 2932, 2871, 2845, 2051, 1638, 1599, 1524, 1490, 1477, 1464, 1342, 1330, 1243, 1162, 1054, 960, 866, 842, 727, 711, 436; HRMS (ESI-FT) *m*/*z*: [M + Na]^+^ Calcd for C_16_H_23_N_3_O_5_Na 360.1530; Found 360.1531.

#### (*S*)-4-Cyano-*N*-(1-(hexylamino)-3-hydroxy-1-oxopropan-2-yl)benzamide (**8b**)

Yellow solid (0.14 g, 82%); R_*f*_ = 0.5 (MeOH:DCM = 1:19); mp = 128–130 °C; ^1^H NMR (300 MHz, CD_3_OD): δ 8.04 (d, *J* = 8.2 Hz, 2H), 7.85 (d, *J* = 8.9 Hz, 2H), 4.61 (t, *J* = 5.6 Hz, 1H), 3.88 (d, *J* = 5.3 Hz, 2H), 3.22 (t, *J* = 7.0 Hz, 2H), 1.48–1.54 (m, 2H), 1.31 (br.s, 6H), 0.90 (t, *J* = 6.7 Hz, 3H); ^13^C{^1^H} NMR (100 MHz, CDCl_3_): δ 170.4, 166.3, 137.2, 128.0, 117.9, 115.7, 62.9, 54.7, 39.9, 31.5, 29.4, 26.6, 22.6, 14.1; IR (ν_max_, solid, cm^–1^): 3285, 2957, 2927, 2858, 2236, 1643, 1548, 1500, 1435, 1374, 1291, 1251, 1162, 1053, 998, 856, 761, 689, 565, 450; HRMS (ESI-FT) *m*/*z*: [M + Na]^+^ Calcd for C_17_H_23_N_3_O_3_Na 340.1632; Found 340.1632.

#### (*S*)-*N*-(1-(Hexylamino)-3-hydroxy-1-oxopropan-2-yl)-4-iodobenzamide (**8c**)

White solid (0.16 g, 74%); R_*f*_ = 0.6 (MeOH:DCM = 1:19); mp = 152–154 °C; ^1^H NMR (300 MHz, CD_3_OD): δ 7.86 (d, *J* = 8.5 Hz, 2H), 7.65 (d, *J* = 8.5 Hz, 2H), 4.58 (t, *J* = 5.2 Hz, 1H), 3.87 (d, *J* = 5.6 Hz, 2H), 3.21 (t, *J* = 15.6 Hz, 2H), 1.47–1.54 (m, 2H), 1.31 (br.s, 6H), 0.89 (t, *J* = 6.8 Hz, 3H); ^13^C{^1^H} NMR (100 MHz, CDCl_3_): δ 171.0, 167.6, 138.1, 132.7, 128.8, 99.5, 62.9, 54.0, 39.8, 31.5, 29.4, 26.5, 22.7, 14.1; IR (ν_max_, solid, cm^–1^): 3301, 2958, 2957, 2856, 2447, 1728, 1279, 1111, 1008, 749, 706, 685, 629, 626, 537; HRMS (ESI-FT) *m*/*z*: [M + Na]^+^ Calcd for C_16_H_23_IN_2_O_3_Na 441.0646; Found 441.0646.

#### (*S*)-*N*-(1-(Hexylamino)-3-hydroxy-1-oxopropan-2-yl)-3,4,5-trimethoxybenzamide (**8d**)

White solid (0.08 g, 41%); R_*f*_ = 0.6 (MeOH:DCM = 1:19); mp = 156–158 °C; ^1^H NMR (300 MHz, CDCl_3_): δ 7.36–7.38 (m, 1H, -NH), 7.03 (s, 2H), 6.99–7.01 (m, 1H, -NH), 4.57–4.62 (m, 1H), 4.20 (dd, *J* = 11.4, 3.2 Hz, 1H), 3.89 (s, 6H), 3.88 (s, 3H), 3.73 (dd. *J* = 11.5, 5.1 Hz, 1H), 3.22–3.29 (m, 2H), 1.46–1.53 (m, 2H), 1.26 (br.s, 6H), 0.85 (t, *J* = 6.7 Hz, 3H); ^13^C{^1^H} NMR (100 MHz, CDCl_3_): δ 171.0, 167.9, 153.3, 141.4, 128.5, 104.5, 63.0, 60.9, 56.5, 54.6, 39.8, 31.5, 29.4, 26.6, 22.6, 14.0; IR (ν_max_, film, cm^–1^): 3313, 3274, 3004, 2929, 2858, 2526, 1660, 1626, 1582, 1531, 1504, 1456, 1413, 1346, 1233, 1128, 1046, 1004, 848, 761, 705, 661; HRMS (ESI-FT) *m*/*z*: [M + Na]^+^ Calcd for C_19_H_30_N_2_O_6_Na 405.1996; Found 405.1996.

#### (*S*)-*N*-(1-(Hexylamino)-3-hydroxy-1-oxopropan-2-yl)-4-methoxybenzamide (**8e**)

Yellow oil (0.02 g, 14%); R_*f*_ = 0.3 (MeOH:DCM = 1:19); ^1^H NMR (300 MHz, CDCl_3_): δ 7.79 (d, *J* = 8.8 Hz, 1H, -NH), 7.28–7.30 (m, 1H, -NH), 7.03–7.06 (m, 1H, -NH), 6.93 (d, *J* = 8.8 Hz, 2H), 4.56–4.61 (m, 1H), 4.22 (dd, *J* = 11.5, 3.1 Hz, 1H), 3.85 (s, 3H), 3.70 (dd, *J* = 11.5, 4.9 Hz, 1H), 3.25 (td, *J* = 13.0, 6.8 Hz, 2H), 1.45–1.52 (m, 2H), 1.25 (br.s, 6H), 0.85 (t, *J* = 6.7 Hz, 3H); ^13^C{^1^H} NMR (100 MHz, CDCl_3_): δ 171.3, 167.9, 162.8, 129.2,125.5, 114.0, 63.1, 55.6, 54.1, 39.7, 39.5, 31.5, 29.4, 26.6, 22.6, 14.1; IR (ν_max_, film, cm^–1^): 3285, 3087, 2956, 2927, 2856, 1660, 1629, 1535, 1507, 1458, 1367, 1327, 1255, 1173, 1109, 1026, 842, 768, 739, 687, 630, 604, 545, 504, 427; HRMS (ESI-FT) *m*/*z*: [M + Na]^+^ Calcd for C_17_H_26_N_2_O_4_Na 345.1785; Found 345.1782.

#### (*S*)-*N*-(1-(Hexylamino)-3-hydroxy-1-oxopropan-2-yl)benzamide (**8f**)

Yellow oil (0.07 g, 45%); R_*f*_ = 0.6 (MeOH:DCM = 1:19); mp = 72–74 °C; ^1^H NMR (300 MHz, CDCl_3_): δ 7.82 (dd, *J* = 7.3 Hz, 2H), 7.54 (t, *J* = 7.3 Hz, 1H), 7.45 (t, *J* = 7.2 Hz, 2H), 7.36–7.39 (m, 1H, -NH), 6.98 (br s, 1H, -NH), 4.58–4.62 (m, 1H), 4.25 (dd, *J* = 11.8, 2.9 Hz, 1H), 3.72 (dd, *J* = 11.3, 4.5 Hz, 1H), 3.23–3.29 (m, 2H), 1.70 (br.s, 1H, −OH), 1.47–1.53 (m, 2H), 1.26 (br.s, 6H), 0.85 (t, *J* = 6.6 Hz, 3H); ^13^C{^1^H} NMR (100 MHz, CDCl_3_): δ 171.2, 168.3, 133.3, 132.3, 128.9, 127.3, 63.0, 54.0, 39.7, 31.5, 29.4, 22.6, 16.6, 14.1; IR (ν_max_, film, cm^–1^): 3293, 2957, 2928, 2856, 1729, 1633, 1531, 1320, 1248, 1038, 692, 454; HRMS (ESI-FT) *m*/*z*: [M + Na]^+^ Calcd for C_16_H_24_N_2_O_3_Na 315.1679; Found 315.1679.

#### (*S*)-*N*-Hexyl-3-hydroxy-2-(2-phenylacetamido)propanamide (**8g**)

White solid (0.06 g, 40%); R_*f*_ = 0.5 (MeOH:DCM = 1:19); mp = 128–130 °C; ^1^H NMR (300 MHz, CD_3_OD): δ 7.31 (d, *J* = 4.3 Hz, 3H), 7.20–7.27 (m, 2H), 4.37 (t, *J* = 5.4 Hz, 1H), 3.74 (d, *J* = 5.3 Hz, 2H), 3.60 (s, 2H, (H5)), 3.17 (t, *J* = 7.1 Hz, 2H), 1.42–1.49 (m, 2H), 1.30 (br.s, 6H), 0.90 (t, *J* = 6.9 Hz, 3H); ^13^C{^1^H} NMR (100 MHz, CDCl_3_): δ 172.3, 170.9, 134.3, 129.3, 129.2, 127.7, 62.6, 53.7, 43.5, 39.6, 31.5, 29.3, 26.6, 22.7, 15.2; IR (ν_max_, solid, cm^–1^): 3279, 2930, 2859, 1635, 1570, 1536, 1455, 1396, 1360, 1233, 1198, 1048, 948, 693, 569; HRMS (ESI-FT) *m*/*z*: [M + Na]^+^ Calcd for C_17_H_26_N_2_O_3_Na 329.1836; Found 329.1838.

#### (*S*)-2-(2-Cyclohexylacetamido)-*N*-hexyl-3-hydroxypropanamide (**8h**)

White solid (0.02 g, 14%); R_*f*_ = 0.6 (MeOH:DCM = 1:19); mp = 132–134 °C; ^1^H NMR (300 MHz, CD_3_OD): δ 6.89 (br.s, 1H, -NH), 6.63–6.65 (m, 1H, -NH), 4.36–4.41 (m, 1H), 4.14 (dd, *J* = 10.6, 3.1 Hz, 1H), 3.58 (dd, *J* = 11.6, 4.3 Hz, 1H), 3.16–3.28 (m, 2H), 1.66 (dd, *J* = 6.7, 3.4 Hz, 2H), 1.64–1.75 (m, 6H), 1.43–1.53 (m, 2H), 1.28 (br.s, 6H), 1.07–1.21 (m, 4H), 0.94–1.01 (m, 2H), 0.88 (t, *J* = 6.8 Hz, 3H); ^13^C{^1^H} NMR (100 MHz, CDCl_3_): δ 173.8, 171.5, 62.9, 53.3, 44.6, 39.6, 35.6, 33.2, 31.6, 29.5, 26.6, 26.2, 26.1, 22.7, 14.2; IR (ν_max_, solid, cm^–1^): 3282, 2918, 2852, 1633, 1545, 1439, 1392, 1374, 1301, 1295, 1255, 1248, 1193, 1072, 690; HRMS (ESI-FT) *m*/*z*: [M + Na]^+^ Calcd for C_17_H_32_N_2_O_3_Na 335.2305; Found 335.2305.

#### 4-Cyano-*N*-(2-hydroxyethyl)benzamide (**9a**)

White solid (0.09 g, 92%). The spectroscopic data is identical to the reported in the literature.^25^ R_*f*_ = 0.4 (MeOH:DCM = 1:19); ^1^H NMR (300 MHz, CDCl_3_): δ 7.89 (d, *J* = 8.8 Hz, 2H), 7.73 (d, *J* = 8.8 Hz, 2H), 6.71 (br.s, 1H, -NH), 3.85 (t, *J* = 5.0 Hz, 2H), 3.65 (td, *J* = 5.4, 5.3 Hz, 2H), 2.30 (br.s, 1H, −OH).

#### 4-Cyano-*N*-(3-hydroxypropyl)benzamide (**9b**)

White solid (0.09 g, 80%); R_*f*_ = 0.4 (MeOH:DCM = 1:19); mp = 102–104 °C; ^1^H NMR (300 MHz, CDCl_3_): δ 7.87 (d, *J* = 8.3 Hz, 2H), 7.72 (d, *J* = 8.5 Hz, 2H), 7.03 (br.s, 1H, -NH), 3.76–3.81 (m, 2H), 3.64 (td, *J* = 6.0, 5.9 Hz, 2H), 2.72 (br.s, 1H, −OH), 1.71–1.88 (m, 2H); ^13^C{^1^H} NMR (100 MHz, CDCl_3_): δ 166.5, 138.3, 132.5, 127.8, 118.1, 115.0, 60.6, 38.2, 31.5; IR (ν_max_, solid, cm^–1^): 3359, 3321, 3264, 3095, 2953, 2922, 2878, 2227, 1633, 1552, 1500, 1444, 1328, 1315, 1222, 1077, 1048, 999, 954, 940, 856, 763, 682, 658, 639, 564, 545; HRMS (ESI-FT) *m*/*z*: [M + Na]^+^ Calcd for C_11_H_12_N_2_O_2_Na 227.0791; Found 227.0793.

#### 4-Cyano-*N*-(4-hydroxybutyl)benzamide (**9c**)

White solid (0.08 g, 66%); R_*f*_ = 0.5 (MeOH:DCM = 1:19); mp = 109–110 °C; ^1^H NMR (300 MHz, CDCl_3_): δ 7.87 (d, *J* = 8.2 Hz, 2H), 7.71 (d, *J* = 8.8 Hz, 2H), 6.89 (br.s, 1H, -NH), 3.73 (t, *J* = 5.6 Hz, 2H), 3.50 (td, *J* = 6.5, 6.1 Hz, 2H), 1.81 (br.s, 1H, −OH), 1.63–1.78 (m, 4H); ^13^C{^1^H} NMR (100 MHz, CD_3_OD): δ 168.3, 140.0, 133.5, 129.1, 119.1, 115.9, 62.5, 41.0, 31.0, 26.9; IR (ν_max_, solid, cm^–1^): 3401, 3296, 2940, 2867, 2232, 1633, 1539, 1500, 1477, 1343, 1298, 1284, 1057, 1017, 994, 860, 769, 666, 636, 567, 544, 480, 408; HRMS (ESI-FT) *m*/*z*: [M + Na]^+^ Calcd for C_12_H_14_N_2_O_2_Na 241.0948; Found 241.0950.

#### (*S*)-4-(2-(Hydroxymethyl)pyrrolidine-1-carbonyl)benzonitrile (**9d**)

Colorless oil (0.09 g, 73%); R_*f*_ = 0.4 (MeOH:DCM = 1:19); ^1^H NMR (300 MHz, CDCl_3_): δ 7.72 (d, *J* = 8.2 Hz, 2H), 7.60 (d, *J* = 8.2 Hz, 2H), 4.33–4.43 (m, 2H), 3.70–3.85 (m, 2H), 3.40–3.47 (m, 2H), 2.13–2.21 (m, 2H), 1.90–1.98 (m, 1H), 1.64–1.85 (m, 2H); ^13^C{^1^H} NMR (100 MHz, CDCl_3_): δ169.8, 140.9, 132.3, 127.8, 118.1, 113.8, 66.0, 61.4, 51.0, 28.2, 25.0; IR (ν_max_, film, cm^–1^): 3389, 2951, 2879, 2229, 1604, 1557, 1508, 1428, 1281, 1048, 850, 763, 717, 594, 545; HRMS (ESI-FT) *m*/*z*: [M + H]^+^ Calcd for C_13_H_15_N_2_O_2_ 231.1128; Found 231.1130.

#### *tert*-Butyl (2-((2-Hydroxyethyl)amino)-2-oxoethyl)carbamate (**10a**)

Yellow oil (0.05 g, 45%). The spectroscopic data is identical to the reported in the literature.^26^ R_*f*_ = 0.3 (MeOH:DCM = 1:19); ^1^H NMR (300 MHz, CDCl_3_): δ 6.73 (br.s, 1H, -NH), 5.34 (br.s, 1H, -NH), 3.79 (d, *J* = 5.9 Hz, 2H), 3.71 (t, *J* = 5.0 Hz, 2H), 3.40–3.49 (m, 2H), 1.84 (br.s, 1H, −OH). 1.44 (s, 9H).

#### *tert*-Butyl (2-((3-Hydroxypropyl)amino)-2-oxoethyl)carbamate (**10b**)

Yellow oil (0.11 g, 91%). The spectroscopic data is identical to the reported in the literature.^26^ R_*f*_ = 0.5 (MeOH:DCM = 1:19); ^1^H NMR (300 MHz, CDCl_3_): δ 6.76 (br.s, 1H, -NH), 5.32–5.37 (m, 1H, -NH), 3.77 (d, *J* = 5.9 Hz, 2H), 3.64 (t, *J* = 5.6 Hz, 2H), 3.39–3.47 (m, 2H), 1.98 (br.s, 1H, −OH), 1.70 (quint, *J* = 6.1 Hz, 2H), 1.44 (s, 9H).

#### *tert*-butyl (2-((4-hydroxybutyl)amino)-2-oxoethyl)carbamate (**10c**)

Yellow oil (0.11 g, 84%); R_*f*_ = 0.5 (MeOH:DCM = 1:19); ^1^H NMR (300 MHz, CDCl_3_): δ 6.55 (br.s, 1H, -NH), 5.26 (br.s, 1H, -NH), 3.75 (d, *J* = 5.9 Hz, 2H), 3.66 (t, *J* = 6.2 Hz, 2H), 3.30 (td, *J* = 6.2, 5.8 Hz, 2H), 2.20 (br.s, 1H, −OH), 1.59–1.62 (m, 4H), 1.44 (s, 9H); ^13^C{^1^H} NMR (100 MHz, CDCl_3_): δ 170.0, 156.4, 80.2, 61.7, 44.2, 39.3, 29.5, 28.4, 26.0; IR (ν_max_, film, cm^–1^): 3301, 2976, 2933, 2870, 1694, 1657, 1528, 1453, 1392, 1366, 1274, 1248, 1164, 1053, 944, 919, 863, 733, 645, 589; HRMS (ESI-FT) *m*/*z*: [M + Na]^+^ Calcd for C_11_H_22_N_2_O_4_Na 269.1472; Found 269.1475

#### *tert*-Butyl (*S*)-(2-(2-(Hydroxymethyl)pyrrolidin-1-yl)-2-oxoethyl)carbamate (**10d**)

Colorless oil (0.05 g, 37%); R_*f*_ = 0.5 (MeOH:DCM = 1:19); ^1^H NMR (300 MHz, CDCl_3_): δ 5.47 (br.s, 1H, -NH), 4.51 (br.s, 1H, −OH), 4.13–4.20 (m, 1H), 3.89 (d, *J* = 4.1 Hz, 2H), 3.63–3.68 (m, 1H), 3.56 (dd, *J* = 11.6, 7.5 Hz, 1H), 3.38–3.46 (m, 2H), 1.80–2.07 (m, 3H), 1.61–1.70 (m, 1H), 1.42 (s, 9H); ^13^C{^1^H} NMR (100 MHz, CDCl_3_): δ169.4, 157.0, 79.9, 66.3, 61.6, 46.8, 43.4, 28.4, 28.0, 24.4; IR (ν_max_, film, cm^–1^): 3406, 2975, 2934, 2878, 1703, 1634, 1504, 1436, 1392, 1366, 1279, 1248, 1164, 1052, 946, 920, 867, 732, 588; HRMS (ESI-FT) *m*/*z*: [M + Na]^+^ Calcd for C_12_H_22_N_2_O_4_Na 281.1472; Found 281.1474.

### Specific Amidation of Unactivated Esters by Serine in the Presence of Alanine under Cs_2_CO_3_-Promoted Conditions

To a 10 mL flamed-dried, two-necked flask containing a magnetic stir bar were added cesium carbonate (87 mg, 0.27 mmol) and the selected Boc-βAla-OMe (**5e**) (108 mg, 0.53 mmol) which was dissolved with anhydrous acetonitrile (0.2 mL) and anhydrous *N,N*-dimethylformamide (0.4 mL). The mixture was stirred at room temperature for 10 min before adding H-Ser-NHHex (**2a**) (200 mg, 1.06 mmol), H-Ala-NHHex (183 mg, 1.06 mmol), and anhydrous acetonitrile (1.1 mL) sequentially. The reaction was stirred at room temperature for 24 h. After this time, the reaction was transferred to a separatory funnel with ethyl acetate (50 mL) and 1 M HCl_(aq.)_ (50 mL). The reaction was extracted, and the organic layer was collected. The aqueous layer was further extracted with ethyl acetate (50 mL × 2). The combined organic layer was washed sequentially with deionized water (100 mL) and saturated sodium bicarbonate (100 mL). The organic layer was dried over MgSO_4_, filtered, and concentrated under reduced pressure to obtain a residue which was purified by flash column chromatography to yield Boc-βAlaSer-NHHex (**6g**) as a white solid (91 mg, 48%). The spectroscopic data are identical to those reported above.

### Synthesis of Org 26576 (**13**)

#### Methyl 2-Chloronicotinate (**11**)

A 100 mL two-necked flask containing a magnetic stir bar was flushed with nitrogen, and anhydrous methanol (15 mL) was added. At 0 °C, thionyl chloride (1.15 mL, 15.87 mmol) was added dropwise, and the reaction was stirred at 0 °C for 10 min before the addition of 2-chloronicotinic acid (1.00 g, 6.35 mmol). The reaction was returned to room temperature and stirred for 10 h. After this time, the solvent was removed under reduced pressure. The crude mixture was basified with saturated sodium bicarbonate solution (80 mL), transferred to a separatory funnel, and then extracted with ethyl acetate (80 mL × 3). The organic layer was collected, dried over MgSO_4_, filtered, and concentrated under reduced pressure to obtain a residue which was purified by flash column chromatography to yield **11** as a colorless liquid (0.742 g, 68%). R_*f*_ = 0.2 (DCM:Hexane = 1:1); ^1^H NMR (300 MHz, CDCl_3_): δ 8.51 (dd, *J* = 4.7, 1.8 Hz, 1H), 8.16 (dd, *J* = 7.6, 2.3 Hz, 1H), 7.32 (dd, *J* = 8.0, 5.0 Hz, 1H), 3.96 (s, 3H); ^13^C{^1^H} NMR (100 MHz, CDCl_3_): δ 164.4, 151.6, 149.5, 140.0, 126.4, 122.0, 52.5; HRMS (ESI-FT) *m*/*z*: [M + H]^+^ Calcd for C_7_H_7_NO_2_Cl 172.0160; Found 172.0158; IR (ν_max_, film, cm^–1^): 2954, 2917, 2849, 1736, 1579, 1561, 1450, 1434, 1402, 1302, 1275, 1245, 1191, 1141, 1065, 959, 833, 765, 743, 645, 539, 488, 451.

#### (*S)*-(2-Chloropyridin-3-yl)(2-(hydroxymethyl)pyrrolidin-1-yl)methanone (**12**)

A 10 mL flamed-dried, two-necked flask containing a magnetic stir bar was flushed with nitrogen, and cesium carbonate (87 mg, 0.27 mmol) and **11** (82 mg, 0.53 mmol) were added dissolved with anhydrous acetonitrile (0.1 mL) and anhydrous *N,N*-dimethylformamide (0.2 mL). The mixture was stirred at room temperature for 10 min before adding l-prolinol (0.11 mL, 1.06 mmol) and anhydrous acetonitrile (0.55 mL) sequentially. The reaction was stirred for a further 24 h. The reaction was transferred to a separatory funnel with ethyl acetate (80 mL) and saturated sodium bicarbonate solution (80 mL). The organic layer was collected, and the aqueous layer was further extracted with ethyl acetate (80 mL × 2). The combined organic layer was dried over MgSO_4_, filtered, and concentrated under reduced pressure to obtain a residue which was purified by flash column chromatography to yield **12** as a colorless oil (0.054 g, 43%). The spectroscopic data are identical to those reported in the literature.^13^ R_*f*_ = 0.6 (MeOH:DCM = 1:19); ^1^H NMR (300 MHz, CDCl_3_): δ 8.44 (dd, *J* = 4.6, 2.0 Hz, 1H), 7.69 (dd, *J* = 7.6, 1.8 Hz, 1H), 7.32 (dd, *J* = 7.7, 4.7 Hz, 1H), 4.32–4.40 (m, 2H), 3.73–3.85 (m, 2H), 3.26–3.34 (m, 2H), 2.13–2.23 (m, 1H), 1.67–2.07 (m, 3H).

#### (*S*)-8,9,9a,10-Tetrahydro-*5H*,7H-pyrido[3,2-*f*]pyrrolo[2,1-*c*][1,4]oxazepin-5-one (Org 26576, **13**)

A 10 mL two-necked flask containing a magnetic stir bar was flushed with nitrogen, and **12** (80 mg, 0.33 mmol) was added dissolved in anhydrous tetrahydrofuran (4.0 mL). At 0 °C, sodium hydride (53% in mineral oil) (34 mg, 0.50 mmol) was added, and the reaction was stirred for 10 min. Next, the reaction was heated to 70 °C and stirred for 15 h. After this time, the solvent was removed under reduced pressure. The crude mixture was transferred to a separatory funnel with ethyl acetate (50 mL). The organic layer was washed with saturated sodium bicarbonate solution (50 mL × 3). Finally, the organic layer was dried over MgSO_4_, filtered, and concentrated under reduced pressure to obtain a residue which was purified by flash column chromatography to yield **13** as a white solid (0.064 g, 80%). The spectroscopic data are identical to those reported in the literature.^13^ R_*f*_ = 0.6 (MeOH:DCM = 1:19); ^1^H NMR (300 MHz, CDCl_3_): δ 8.62 (dd, *J* = 7.6, 1.7 Hz, 1H), 8.37 (dd, *J* = 4.7, 1.8 Hz, 1H), 7.10 (dd, *J* = 7.6, 4.7 Hz, 1H), 4.62 (d, *J* = 11.1 Hz, 1H), 4.12 (dd, *J* = 12.3, 8.2 Hz, 1H), 3.97–4.05 (m, 1H), 3.68–3.84 (m, 2H), 2.22–2.31 (m, 1H), 1.98–2.08 (m, 1H), 1.84–1.95 (m, 1H), 1.62–1.75 (m, 1H). [α]^25^_D_ = +220.58 (*c* 0.34, CHCl_3_) (Lit.^27^ [α]^25^_D_ = +230.96 (*c* 1.55, CHCl_3_)).

## Data Availability

The data underlying this study are available in the published article and its Supporting Information.

## Supporting Information Available

Detailed experimental procedure for the synthesis of serine and threonine derivatives **2a**–**2g**, glycine derivatives **3b**–**3e**, **4a**–**4d** and methyl esters **3a**, **5a**–**5u**, **7a**, **7c**–**7e**; NMR spectra for all of the synthesized compounds; chiral HPLC chromatograms for UV-active products (PDF)
FAIR data, including the primary NMR FID files, for compounds **1b**, **1c**,**1d**, **2a**–**2g**, **3a**–**3e**, **4a**–**4d**, **5a**–**5u**, **6aa**–**6ae**, **6b**–**6w**, **6ya**, **6yb**, **6z**, **7a**, **7c**, **7d**, **7e**, **8a**–**8h**, **9a**–**9d**, **10a**–**10d**, **11**, **12**, **13** (ZIP)
