# Supplementary material for: Cesium Carbonate Promoted Direct Amidation of Unactivated Esters with Amino Alcohol Derivatives
Source: J Org Chem. 2024 Mar 25;89(7):4958–70. doi: 10.1021/acs.joc.4c00162 (PMC11002823; doi:10.1021/acs.joc.4c00162)
Supplement: Supplementary file 1 — jo4c00162_si_001.pdf [file jo4c00162_si_001.pdf]

# Cesium Carbonate-Promoted Direct Amidation of Unactivated Esters with Amino Alcohol Derivatives

Chih-Hung Kuo,† Wen-Tsai Hsieh,† Ya-Hsu Yang,† Teng-Li Hwang,† Yu-Shan Cheng,† Yuya A. Lin*,†,‡

†*Department of Chemistry, National Sun Yat-sen University, Kaohsiung 804, Taiwan*
‡*Department of Medicinal and Applied Chemistry, Kaohsiung Medical University, Kaohsiung 807, Taiwan*

## Contents

**Synthesis of Serine or Threonine Derivatives 2a-2g** **S2**

**Synthesis of *N*-Boc-Protected Glycine Ester Derivatives 3b-3e** **S10**

**Synthesis of *N*-Protected Glycine Methyl Ester Derivatives 4a-4d** **S12**

**Synthesis of Methyl Ester Derivatives** **S14**

**Attempted direct amidation with other N-terminal amino acids** **S22**

**NMR Spectra** **S23**

**Chiral HPLC Chromatograms** **S168**

**References** **S174**

**Synthesis of Serine or Threonine Derivatives 2a-2g:**

**H-Ser-NHHex (2a).** To a 100 mL round-bottomed flask containing a magnetic stir bar was added L-serine (1.00 g, 9.52 mmol) and di-*tert*-butyl dicarbonate ($Boc_2O$, 2.49 g, 11.42 mmol) dissolved in tetrahydrofuran (5.00 mL). Sodium hydroxide (0.46 g, 11.42 mmol) dissolved in distilled water (5.00 mL) was further added at 0 °C. The reaction mixture was returned to room temperature and stirred for 18 hours. After this time, the solvent was removed under reduced pressure to obtain a residue. The residue was transferred to a separatory funnel with distilled water (30 mL). The aqueous layer was washed with *n*-hexane (30 mL × 2) then acidified at 0 °C with 12 M $HCl_{(aq.)}$ until pH value is 1. The acidified aqueous layer was extracted with ethyl acetate (30 mL × 3). The combined organic layer was dried over $MgSO_4$, filtered, and concentrated under reduced pressure to give the crude **Boc-Ser-OH** which was used directly in the next step without any purification. To a 250 mL round-bottomed flask containing a magnetic stir bar was added the crude **Boc-Ser-OH** and HOBt hydrate (1.29 g, 9.52 mmol). The flask was then dried on high vacuum for 1 hour. After this time, this flask was flushed with nitrogen then added anhydrous tetrahydrofuran (50 mL) and 4-methylmorphiline (40 mL) at 0 °C. The mixture was stirred for 5 mins before adding *N,N'*-dicyclohexylcarbodiimide (DCC, 2.16 g, 10.47 mmol) in tetrahydrofuran (10 mL). Finally, *n*-hexylamine (1.33 mL, 9.52 mmol) was added. The reaction was stirred for 2 hours at 0 °C and kept stirring for further 24 hours at room temperature. After this time, the reaction was filtered to remove the urea-byproduct and the filtrate was concentrated under reduced pressure to obtain a residue. The residue was transferred to a separatory funnel with ethyl acetate (60 mL). The organic layer was washed sequentially with saturated sodium bicarbonate solution (60 mL × 2), 5 % potassium hydrogen sulfite solution (60 mL × 2), and brine (60 mL). The organic layer was dried over $MgSO_4$, filtered, and concentrated under reduced pressure to give the crude **Boc-Ser-NHHex (1a)** which was used directly in the next step without any purification. To a 250 mL round-bottomed flask containing a magnetic stir bar was added the crude **1a** was added methanol (30 mL) and 12 M $HCl_{(aq.)}$ (60 mL). The reaction was stirred until TLC analysis revealed complete consumption of **1a** (around 1 hour). Methanol was removed under

reduced pressure and the resulting solution was basified at 0 °C with 4 M $NaOH_{(aq.)}$ until the pH value is greater than 9 then transferred to a separatory funnel. The aqueous layer was extracted with ethyl acetate (60 mL × 3). The combined organic layer was dried over $MgSO_4$, filtered, and concentrated under reduced pressure to obtain a residue which was purified by flash column chromatography to yield **2a** as a yellow solid (1.40 g, 78%). $R_f$ = 0.3 (MeOH:DCM = 1:19 + 1% $NH_4OH$); m.p. = 76-78 °C; $^1$H NMR (300 MHz, $CDCl_3$): δ 7.42 (br.s, 1H, -NH), 3.85 (dd, *J* = 10.6, 5.1 Hz, 1H), 3.69 (dd, *J* = 10.7, 5.8 Hz, 1H), 3.42 (t, *J* = 5.5 Hz, 1H), 3.21-3.28 (m, 2H), 2.06 (br.s, 3H, -OH, $-NH_2$), 1.47-1.53 (m, 2H), 1.29 (br.s, 6H), 0.88 (t, *J*=6.5 Hz, 3H); $^{13}C\{^1H\}$ NMR (75 MHz, $CDCl_3$): δ 173.8, 65.4, 56.1, 39.3, 31.6, 29.6, 26.7, 22.6, 14.1; IR ($\nu_{max}$, solid): 3331, 3277, 3094, 2957, 2924, 2856, 1636, 1557, 1465, 1376, 1295, 1241, 1226, 1143, 1061, 1013, 941, 891, 759, 724, 693, 561, 439; HRMS (ESI-FT) m/z: $[M + Na]^+$ Calcd for $C_9H_{20}N_2O_2Na$ 211.1417; Found 211.1415.

**Boc-Thr-NHHex (1b).** To a 100 mL round-bottomed flask containing a magnetic stir bar was added L-threonine (0.50 g, 4.20 mmol) and di-*tert*-butyl dicarbonate ($Boc_2O$, 1.10 g, 5.04 mmol) dissolved in tetrahydrofuran (5 mL). Next, sodium hydroxide (0.20 g, 5.04 mmol) dissolved in deionized water (5 mL) was added to the reaction. The reaction was stirred for 18 hours. After this time, the tetrahydrofuran was removed under reduced pressure to obtain a residue. The residue was transferred to a separatory funnel with deionized water (30 mL). The aqueous layer was washed with *n*-hexane (30 mL) then acidified at 0 °C with 10 M $HCl_{(aq.)}$ until pH value is 1. The aqueous layer was extracted with ethyl acetate (50 mL × 3). The combined organic layer was dried over $MgSO_4$, filtered, and concentrated under reduced pressure to obtain the crude **Boc-Thr-OH** which was used directly in the next step without any purification. To a 250 mL round-bottomed flask containing a magnetic stir bar was added the crude **Boc-Thr-OH** and benzotriazol-1-yloxytripyrrolidinophosphonium hexafluorophosphate (PyBOP, 2.44 g, 4.69 mmol) and dried on high vacuum for 30 mins. After this time, the flask was flushed with nitrogen. Next, anhydrous dichloromethane (65 mL) and *N,N*-Diisopropylethylamine (DIEA, 1.60 mL, 9.10 mmol) were added subsequently at 0 °C. The reaction

was stirred for 10 mins before *n*-hexylamine (0.46 mL, 3.50 mmol) was added. The reaction was returned to room temperature and stirred for 24 hours. After this time, the solvent was removed under reduced pressure to obtain a residue. The residue was transferred to a separatory funnel with ethyl acetate (100 mL). The organic layer was washed sequentially with 5 % potassium hydrogen sulfite solution (100 × 3), 5 % sodium bicarbonate solution (100 mL), and brine (100 mL). The organic layer was then dried over $MgSO_4$, filtered, and concentrated under reduced pressure to obtain a residue which was purified by flash column chromatography to yield **1b** as a colorless oil (0.94 g, 74 %). $R_f$ = 0.4 (EtOAc:Hexane = 1:1); $^1$H NMR (300 MHz, $CDCl_3$): δ 6.65 (br.s, 1H, -NH), 5.51 (d, *J* = 7.8 Hz, 1H, -NH), 4.33 (qd, *J* = 10.7, 2.4 Hz, 1H), 3.96 (dd, *J* = 8.2, 1.8 Hz, 1H), 3.59 (br.s, 1H, -OH), 3.14-3.32 (m, 2H), 1.49-1.51 (m, 2H), 1.45 (s, 9H), 1.24-1.37 (m, 6H), 1.17 (d, *J* = 6.5 Hz, 3H), 0.87 (t, *J* = 6.7 Hz, 3H). $^{13}C\{^1H\}$ NMR (75 MHz, $CDCl_3$): δ 171.6, 156.8, 80.5, 68.8, 58.1, 39.6, 31.5, 29.5, 28.4, 26.6, 22.6, 18.3, 14.1; HRMS (ESI-FT) m/z: $[M + Na]^+$ Calcd for $C_{15}H_{30}N_2O_4Na$ 325.2098; Found 325.2099; IR ($\nu_{max}$, film): 3320, 2958, 2931, 2860, 1692, 1649, 1598, 1511, 1403, 1366, 1304, 1218, 1155, 1093, 1016, 949, 884, 831, 812, 736, 676, 524, 502, 427.

**H-Thr-NHHex (2b).** To a 250 mL round-bottomed flask containing a magnetic stir bar was added **1b** (0.713 g, 2.36 mmol) was added methanol (7.40 mL) and 12 M $HCl_{(aq.)}$ (15 mL). The reaction was stirred until TLC analysis revealed complete consumption of **1b** (around 1 hour**)**. Methanol was removed under reduced pressure and the resulting solution was basified at 0 °C with 4 M $NaOH_{(aq.)}$ until pH value is greater than 9 then transferred to a separatory funnel with ethyl acetate (30 mL). The aqueous layer was extracted with ethyl acetate (30 mL × 3). The combined organic layer was dried over $MgSO_4$, filtered, and concentrated under reduced pressure to yield **2b** as a white solid (0.52 g, 83 %). $R_f$ = 0.2 (MeOH:DCM = 1:19 + 1% $NH_4OH$); m.p. = 77-78 °C; $^1$H NMR (300 MHz, $CDCl_3$): δ 7.40 (br.s, 2H, $-NH_2$), 4.26 (qd, *J* = 10.8, 3.5 Hz, 1H), 3.21-3.29 (m, 3H), 1.98 (br.s, 1H, -OH), 1.46-1.53 (m, 2H), 1.28-1.34 (m, 6H), 1.19 (d, *J* = 6.5 Hz, 3H), 0.88 (t, *J* = 6.7 Hz, 3H); $^{13}C\{^1H\}$ NMR (75 MHz, $CDCl_3$): δ 173.7, 67.8, 59.7, 39.3, 31.5, 29.5, 26.7, 22.6, 19.0, 14.0; HRMS (ESI-FT)

m/z: $[M + H]^+$ Calcd for $C_{10}H_{23}N_2O_2$ 203.1754; Found 203.1756; IR ($\nu_{max}$, solid): 3326, 3274, 3095, 2953, 2925, 2855, 1607, 1646, 1554, 1459, 1435, 1374, 1264, 1217, 1203, 1153, 1119, 1108, 1086, 1016, 923, 880, 813, 706, 525, 504, 449, 402.

**Ac-Ser-NHHex (2c).** To a 100 mL round-bottomed flask containing a magnetic stir bar was added **2a** (0.50 g, 2.70 mmol). The flask was then dried on high vacuum for 1 hour. After this time, this flask was flushed with nitrogen and added anhydrous dichloromethane (8 mL). Triethylamine (0.44 mL, 3.20 mmol) and acetyl chloride (0.23 mL, 3.20 mmol) were added subsequently at 0 °C and the reaction was stirred for 4 hours at room temperature. After 4 hours, the solvent was removed under reduced pressure to obtain a residue. The residue was transferred to a separatory funnel with ethyl acetate (50 mL). The organic layer was washed sequentially with deionized water (50 mL × 3) and brine (50 mL). The organic layer was then dried over $MgSO_4$, filtered, and concentrated under reduced pressure to obtain a residue which was purified by flash column chromatography to yield **2c** as a white solid (0.13 g, 20 %). $R_f$ = 0.2 (EtOAc:Hexane = 4:1); m.p. = 94-96 °C; $^1$H NMR (300 MHz, $CDCl_3$): δ 6.99 (br.s, 1H, -NH), 6.89 (d, $J$ = 6.9 Hz, 1H, -NH), 4.38-4.44 (m, 1H), 4.05 (dd, $J$ = 11.1, 3.5 Hz, 1H), 3.61 (dd, $J$ = 11.1, 5.3 Hz, 1H), 3.18-3.25 (m, 2H), 2.04 (s, 3H), 1.41-1.51 (m, 2H), 1.28 (br.s, 6H,), 0.87 (t, $J$ = 6.8 Hz, 3H); $^{13}C\{^1H\}$ NMR (100 MHz, $CDCl_3$): δ 171.4, 170.7, 63.1, 54.6, 39.7, 31.5, 29.3, 26.6, 23.1, 22.6, 14.0; HRMS (ESI-FT) m/z: $[M + Na]^+$ Calcd for $C_{11}H_{22}N_2O_3Na$ 253.1523; Found 253.1523; IR ($\nu_{max}$, solid): 3290, 2928, 2856, 1646, 1558, 1377, 1281, 1251, 1151, 1051, 726, 684, 594.

**Fmoc-Ser(O*^t*Bu)-NHHex (1c).** To a 100 mL round-bottom flask with a magnetic stir bar was added **Fmoc-Ser(O*^t*Bu)-OH** (1.00g, 2.61 mmol), TBTU (1.00 g, 3.10 mmol) and HOBt hydrate (0.42 g, 3.10 mmol) then dried on high vacuum for 1 hour. The flask was flushed with nitrogen and anhydrous dichloromethane (15 mL) was added to obtain a suspension. The suspension was stirred and cooled to 0 °C. After 10 mins, *N,N*-diisopropylethylamine (DIEA, 0.54 mL, 3.12 mmol) and *n*-hexylamine (0.52 mL, 3.90 mmol) were added sequentially to the reaction mixture. The reaction was returned to

room temperature and stirred for 24 hours. After 24 hours, the solvent was removed under reduced pressure. The residue was transferred to a separatory funnel with ethyl acetate (100 mL) then washed sequentially with 1 M HCl $_{(aq.)}$ (100 mL × 3), saturated sodium bicarbonate solution (100 mL) and brine (100 mL). Then the organic layer was dried over $MgSO_4$, filtered, and concentrated under reduced pressure to obtain a residue which was purified by flash column chromatography to yield **1c** as a white solid (1.20 g, 99 %). $R_f$ = 0.5 (EtOAc:Hexane = 3:7); m.p. = 96-98 °C; $^1$H NMR (300 MHz, $CDCl_3$): δ 7.76 (d, *J* = 7.5 Hz, 2H), 7.60 (d, *J* = 7.4 Hz, 2H), 7.40 (t, *J* = 7.1 Hz, 2H), 7.31 (t, *J* = 7.1 Hz, 2H), 6.58 (br.s, 1H, -NH), 5.77 (br.s, 1H, -NH), 4.41 (d, *J* = 6.8 Hz, 2H), 4.23 (t, *J* = 7.3 Hz, 1H), 4.13-4.18 (m, 1H), 3.81 (dd, *J* = 8.6, 3.8 Hz, 1H), 3.33-3.38 (m, 1H), 3.24-3.31 (m, 2H), 1.45-1.52 (m, 2H), 1.29 (br.s, 6H), 1.20 (s, 9H), 0.89 (t, *J* = 6.9 Hz, 3H); $^{13}C\{^1H\}$ NMR (100 MHz, $CDCl_3$): δ 170.2, 156.2, 144.0, 141.4, 127.9, 127.2, 125.2, 120.1, 74.3, 67.1, 62.0, 54.3, 47.3, 39.7, 31.6, 29.6, 27.6, 26.7, 22.7, 14.2; IR ($\nu_{max}$, solid): 3296, 3065, 2957, 2928, 2859, 1693, 1650, 1538, 1466, 1450, 1363, 1296, 1236, 1194, 1084, 1023, 879, 756, 736, 671, 621, 595, 547, 531, 426; HRMS (ESI-FT) m/z: $[M + Na]^+$ Calcd for $C_{28}H_{38}N_2O_4Na$ 489.2724; Found 489.2721.

**H- Ser(O*t*Bu)-NHHex (2d).** To a 100 mL round-bottom flask with a magnetic stir bar was added **1c** (1.00 g, 2.14 mmol) and dichloromethane (3.0 mL). Piperidine (0.62 mL, 6.28 mmol) was added after the mixture was totally dissolved. The reaction was stirred for 2 hours until TLC analysis revealed complete consumption of the starting material and the solvent was removed under reduced pressure to obtain a crude. The crude was purified by flash column chromatography to yield **2d** as a yellow liquid (0.52 g, 100 %). The spectroscopic data is identical to those reported in the literature.[1] $R_f$ = 0.3 (MeOH:DCM = 1:19 + 1 % $NH_4OH$); $^1$H NMR (300 MHz, $CDCl_3$): δ 7.32 (br.s, 1H, -NH), 3.61 (dd, *J* = 7.6, 3.6 Hz, 1H), 3.39-3.49 (m, 2H), 3.23 (td, *J* = 7.0, 6.2 Hz, 2H), 1.45-1.52 (m, 2H), 1.29-1.36 (m, 6H), 1.18 (s, 9H), 0.88 (t, *J* = 6.8 Hz, 3H).

**Boc-SerAla-NHHex (1d).** To a 250 mL round-bottomed flask containing a magnetic stir bar was added **Boc-Ala-NHHex**[2] (1.58 g, 5.80 mmol) and methanol (20 mL). Then 12 M HCl $_{(aq.)}$ (20 mL)

was added dropwise to the stirred mixture. The mixture was stirred for 1 hour until TLC analysis revealed complete consumption of **Boc-Ala-NHHex** and methanol was removed under reduced pressure. The resulting solution was basified with 4 M NaOH $_{(aq.)}$ at 0 °C until pH value is 14 then transferred to a separatory funnel. The aqueous layer was extracted with ethyl acetate (100 mL × 3). The combined organic layer was dried over $MgSO_4$, filtered, and concentrated under reduced pressure to obtain **H-Ala-NHHex** in quantitative yield. To a 250 mL round-bottomed flask containing a magnetic stir bar was added **Boc-Ser-OH** (1.43 g, 6.96 mmol) and benzotriazol-1-yloxytripyrrolidinophosphonium hexafluorophosphate (PyBOP, 4.04 g, 7.77 mmol) then dried on high vacuum for 30 mins before it was flushed with nitrogen. Next, anhydrous dichloromethane (100 mL) and *N,N*-Diisopropylethylamine (DIEA, 2.61 mL, 15.08 mmol,) were added subsequently at 0 °C and the reaction was stirred for 10 mins before **H-Ala-NHHex** was added. The reaction was returned to room temperature and stirred for 24 hours. After this time, the solvent was removed under reduced pressure to obtain a residue. The residue was transferred to a separatory funnel with ethyl acetate (60 mL). The organic layer was washed sequentially with 5 % potassium hydrogen sulfate solution (60 mL × 3), 5 % sodium bicarbonate solution (60 mL), and brine (60 mL). The collected organic layer was dried over $MgSO_4$, filtered, and concentrated under reduced pressure to obtain a residue which was purified by flash column chromatography to yield **1d** as a white solid (1.00 g, 48%). $R_f$ = 0.3 (EtOAc:Hexane = 7:3); m.p. = 106-108 °C; $^1$H NMR (300 MHz, $CDCl_3$): δ 7.15-7.17 (m, 1H, -NH), 6.62 (br.s, 1H, -NH), 5.63-5.65 (m, 1H, -NH), 4.40-4.49 (m, 1H), 4.21 (br.s, 1H), 3.96 (dd, *J* = 10.8, 4.3 Hz, 1H), 3.10-3.29 (dd, *J* = 10.9, 6.3 Hz, 1H), 1.94 (br.s, 1H, -OH), 1.47-1.49 (m, 2H), 1.44 (s, 9H), 1.39 (d, *J* = 7.1 Hz, 3H), 1.27 (br.s, 6H), 0.87 (t, *J* = 6.8 Hz, 3H); $^{13}C\{^1H\}$ NMR (100 MHz, $CDCl_3$): δ 172.4, 171.3, 156.2, 80.7, 63.1, 55.8, 49.5, 39.9, 31.6, 28.4, 26.6, 22.7, 18.2, 14.1; IR ($\nu_{max}$, solid): 3300, 3074, 2961, 2931, 2872, 2859, 1675, 1625, 1532, 1450, 1364, 1304, 1236, 1172, 1058, 1008, 932, 907, 873, 853, 659; HRMS (ESI-FT) m/z: $[M + Na]^+$ Calcd for $C_{17}H_{33}N_3O_5Na$ 382.2312; Found 382.2315.

**H-SerAla-NHHex (2e).** To a 250 mL round-bottomed flask containing a magnetic stir bar was added the crude of **1d** (1.26 g, 3.5 mmol) was added methanol (20 mL) and 12 M HCl $_{(aq.)}$ (44 mL). The reaction was sutured for 1 hour until TLC analysis revealed complete consumption of **1d**. Methanol was removed under reduced pressure and the resulting solution was basified with 4 M NaOH$_{(aq.)}$ at 0 °C until pH value is greater than 9 then transferred to a separatory funnel. The aqueous layer was extracted with ethyl acetate (150 mL × 3). Combined organic layer was dried over $MgSO_4$, filtered, and concentrated under reduced pressure to yield **2e** as a white solid; $R_f$ = 0.4 (MeOH:DCM = 1:19 + 1% $NH_4OH$); $^1$H NMR (400 MHz, $CD_3OD$): δ 4.35 (q, $J$ = 7.3 Hz, 1H), 3.72 (dd, $J$ = 10.0, 5.6 Hz, 1H), 3.60 (dd, $J$ = 10.1, 6.4 Hz, 1H), 3.43 (t, $J$ = 6.2 Hz, 1H), 3.15 (t, $J$ = 7.1 Hz, 2H), 1.45-1.50 (m, 2H), 1.36 (d, $J$ = 7.2 Hz, 3H), 1.26-1.34 (m, 6H), 0.91 (t, $J$ = 6.4 Hz, 3H); $^{13}$C{$^1$H} NMR (100 MHz, $CD_3OD$): δ 175.6, 174.8, 65.8, 57.4, 50.4, 40.4, 32.6, 30.2, 27.6, 23.6, 18.2, 14.4; IR ($\nu_{max}$, solid): 3271, 2926, 2856, 1637, 1545, 1444, 1374, 1295, 1247, 1226, 1152, 1063, 1021, 951, 890, 693, 570, 530; HRMS (ESI-FT) m/z: $[M + H]^+$ Calcd for $C_{12}H_{26}N_3O_3$ 260.1969; Found 260.1971.

**H-Ser-NH$_2$ (2f).** To a 250 mL two-necked flask containing a magnetic stir bar was dried on high vacuum for 30 mins, flushed with nitrogen and then added anhydrous methanol (20 mL). At 0 °C, thionyl chloride (1.75 mL, 24.0 mmol) was added slowly and the mixture was stirred for 10 mins. L-serine (1.26 g, 12.0 mmol) was added then the mixture was returned to room temperature and continued to stir for 16 hours. The solvent was removed under reduced pressure to result the crude **H-Ser-OMe**, which was used in the next step without further purification. At 0 °C, ammonia solution (6 mL) was added slowly to the 250 mL two-necked flask containing the crude **H-Ser-OMe**. The mixture was returned to room temperature and stirred for 4 hours. Toluene was added to the flask and solvents were evaporated under reduced pressure and product **2f** was obtained as a yellow solid (1.224 g, 97 %). The spectroscopic data is identical to those reported in the literature.[3] $^1$H NMR (300 MHz, $D_2O$): δ 4.7 (t, $J$ = 4.5 Hz,1H), 4.08−3.96 (m, 2H).

**H-Ser-NHBn (2g).** To a 100 mL round-bottomed flask containing a magnetic stir bar was added L-serine (1.00 g, 9.52 mmol) and di-*tert*-butyl dicarbonate ($Boc_2O$, 2.49 g, 11.42 mmol) dissolved in tetrahydrofuran (5.00 mL). Sodium hydroxide (0.46 g, 11.42 mmol) dissolved in distilled water (5.00 mL) was further added at 0 °C. The reaction mixture was returned to room temperature and stirred for 18 hours. After this time, the solvent was removed under reduced pressure to obtain a residue. The residue was transferred to a separatory funnel with distilled water (30 mL). The aqueous layer was washed with *n*-hexane (30 mL × 2) then acidified at 0 °C with 12 M $HCl_{(aq.)}$ until pH value is 1. The acidified aqueous layer was extracted with ethyl acetate (30 mL × 3). The combined organic layer was dried over $MgSO_4$, filtered, and concentrated under reduced pressure to give the crude **Boc-Ser-OH** which was used directly in the next step without any purification. To a 250 mL round-bottomed flask containing a magnetic stir bar was added the crude **Boc-Ser-OH** and HOBt hydrate (1.29 g, 9.52 mmol). The flask was then dried on high vacuum for 1 hour. After this time, this flask was flushed with nitrogen then added anhydrous tetrahydrofuran (50 mL) and 4-methylmorphiline (40 mL) at 0 °C. The mixture was stirred for 5 mins before adding *N,N'*-dicyclohexylcarbodiimide (DCC, 2.16 g, 10.47 mmol) in tetrahydrofuran (10 mL). Finally, benzylamine (1.12 mL, 10.3 mmol) was added. The reaction was stirred for 2 hours at 0 °C and kept stirring for further 24 hours at room temperature. After this time, the reaction was filtered to remove the urea-byproduct and the filtrate was concentrated under reduced pressure to obtain a residue. The residue was transferred to a separatory funnel with ethyl acetate (60 mL). The organic layer was washed sequentially with saturated sodium bicarbonate solution (60 mL × 2), 5 % potassium hydrogen sulfite solution (60 mL × 2), and brine (60 mL). The organic layer was dried over $MgSO_4$, filtered, and concentrated under reduced pressure to give the crude **Boc-Ser-NHBn** which was used directly in the next step without any purification. To a 250 mL round-bottomed flask containing a magnetic stir bar was added the crude **Boc-Ser-NHBn** was added methanol (30 mL) and 12 M $HCl_{(aq.)}$ (60 mL). The reaction was stirred until TLC analysis revealed complete consumption of **Boc-Ser-NHBn** (around 1 hour). Methanol was removed under reduced pressure and the resulting solution was basified at 0 °C with 4

M $NaOH_{(aq.)}$ until the pH value is greater than 9 then transferred to a separatory funnel. The aqueous layer was extracted with ethyl acetate (60 mL × 3). The combined organic layer was dried over $MgSO_4$, filtered, and concentrated under reduced pressure to obtain a residue which was purified by flash column chromatography to yield **2g** as a yellow solid (405 mg, 22%). The spectroscopic data is identical to the reported in the literature.[4] $R_f$ = 0.25 (MeOH:DCM = 1:9); $^1$H NMR (300 MHz, $CDCl_3$): δ 7.79 (br.s, 1H), 7.26–7.33 (m, 5H), 4.45 (d, *J* = 5.6Hz, 2H), 3.91 (dd, *J* = 11.9, 3.5 Hz, 1H), 3.73 (dd, *J* = 9.6, 6.7 Hz, 1H), 3.44-3.54 (m, 1H), 1.84 (br.s, 3H).

**Synthesis of *N*-Boc-Protected Glycine Ester Derivatives 3b-3e:**

**Boc-Gly-OEt (3b).** To a 100 mL two-necked flask containing a magnetic stir bar was added *N*-Boc-protected glycine derivative (1.00 g, 5.71 mmol) and dried on high vacuum. After 20 mins, the flask was flushed with nitrogen and added anhydrous DMF (20 mL). Next, potassium carbonate (0.98 g, 7.05 mmol) and ethyl iodide (1.82 mL, 11.75 mmol) were added at 0 °C. The reaction was returned to room temperature and stirred for 18 hours. After 18 hours, the mixture was transferred to a separatory funnel with diethyl ether (350 mL) and distilled water (350 mL). The organic layer was collected, and the aqueous layer was further extracted with diethyl ether (350 mL × 2). The combined organic layer was washed sequentially with saturated sodium bicarbonate (350 mL) and brine (350 mL). The organic layer was dried over $MgSO_4$, filtered, and concentrated under reduced pressure to yield **3b** as a colorless liquid (1.15 g, 99 %). The spectroscopic data is identical to the reported in the literature.[5] $R_f$ = 0.3 (EtOAc:Hexane = 1:9); $^1$H NMR (300 MHz, $CDCl_3$): δ 5.00 (br.s, 1H, -NH), 4.20 (q, *J* = 7.2 Hz, 2H), 3.89 (d, *J* = 5.4 Hz, 2H), 1.45 (s, 9H), 1.27 (t, *J* = 7.1 Hz, 3H).

**Boc-Gly-OBn (3c).** To a 25 mL two-necked flask containing a magnetic stir bar was added *N*-Boc-protected glycine derivative (0.50 g, 2.85 mmol) and potassium carbonate (0.24 g, 1.71 mmol) then dried on high vacuum for 20 mins. Anhydrous DMF (5 mL) was added and stirred for 15 mins before adding benzyl bromide (0.38 mL, 3.14 mmol) dropwise at 0 °C. The reaction was returned to room

temperature and stirred for 18 hours. After 18 hours, the mixture was transferred to a separatory funnel with diethyl ether (100 mL) and distilled water (100 mL). The organic layer was collected, and the aqueous layer was further extracted with diethyl ether (100 mL × 2). The combined organic layer was washed with brine (100 mL) then dried over $MgSO_4$, filtered, and concentrated under reduced pressure to obtain a residue which was purified by flash column chromatography to yield **3c** as a white solid (0.708 g, 91 %). The spectroscopic data is identical to those reported in the literature.[5] $R_f$ = 0.5 (EtOAc:Hexane = 1:4); $^1$H NMR (300 MHz, $CDCl_3$): δ 7.35 (br.s, 5H), 5.18 (s, 2H), 5.00 (br.s, 1H, -NH), 3.95 (d, $J$ = 5.3 Hz, 2H), 1.44 (s, 9H).

**Boc-Gly-O$^t$Bu (3d).** To a 250 mL round-bottomed flask containing a magnetic stir bar and glycine (0.50 g, 6.66 mmol) was dried on high vacuum for 10 mins. After this time, the flask was flushed with nitrogen and *tert*-butyl acetate (16 mL) was added. Next, 70 % perchloric acid (0.80 mL, 13.32 mmol) was added slowly at 0 °C. The reaction was returned to room temperature and stirred for 2 hours. The mixture was then transferred to a separatory funnel and extracted sequentially with distilled water (35 mL) and 1 M HCl $_{(aq.)}$ (20 mL). The combined aqueous layer was basified with 10 M NaOH $_{(aq.)}$ until the pH value is greater than 9 and then extracted with dichloromethane (25 mL × 3). The combined organic layer was dried over $MgSO_4$ and filtered to yield a solution containing **H-Gly-O$^t$Bu** which is used directly in the next step. To the DCM solution containing **H-Gly-O$^t$Bu** was added triethylamine (1.11 mL, 7.99 mmol) at 0 °C then stirred for 10 mins before adding di-*tert*-butyl dicarbonate ($Boc_2O$, 1.74 g, 7.99 mmol). The reaction was returned to room temperature and stirred for 24 hours. After this time, the solvent was removed under reduced pressure to obtain a residue. The residue was transferred with to a separatory funnel ethyl acetate (30 mL). The organic layer was washed sequentially with 10 % citric acid $_{(aq.)}$ (25 mL × 2), saturated sodium bicarbonate solution (25 mL × 2), and brine (25 mL × 2). The organic layer was dried over $MgSO_4$, filtered, and concentrated under reduced pressure to obtain a crude which was purified by flash column chromatography to yield **3d** as a white solid (0.95 g, 62 %). The spectroscopic data is identical to the reported in the literature.[6]

$R_f$ = 0.4 (EtOAc:Hexane = 1:9); $^1$H NMR (300 MHz, $CDCl_3$): δ 4.96 (br.s, 1H, -NH), 3.79 (d, $J$ = 5.1 Hz, 2H), 1.47 (s, 9H), 1.45 (s, 9H).

**Boc-Gly-OPh (3e).** To a dried, 50 mL two-necked flask containing a magnetic stir bar under nitrogen was added **Boc-Gly-OH** (1.00 g, 5.71 mmol) in anhydrous THF/ACN (1:1, 6.0 mL) and thiophenol (0.553 g, 5.71 mmol) in anhydrous THF/ACN (1:1, 2.0 mL) were added sequentially. Next, a solution of *N,N'*-dicyclohexylcarbodiimide (DCC, 1.33 g, 6.28 mmol) and 4-dimethylaminopyridine (DMAP, 0.03 g, 0.23 mmol) in anhydrous THF/ACN (1:1) were added sequentially at -10 °C. The mixture was returned to room temperature and stirred for 24 hours. After this time, the solvent was removed under reduced pressure to obtain a residue. Diethyl ether (150 mL) was added to the residue and the mixture was filtered. The filtrate was transferred to a separatory funnel and washed sequentially with 1 M HCl $_{(aq.)}$ (100 mL × 2), saturated sodium bicarbonate solution (100 mL × 2), and brine (100 mL). The organic layer was dried over $MgSO_4$, filtered, and concentrated under reduced pressure to obtain a crude which was purified by flash column chromatography to yield **3e** as a white solid (1.13 g, 79 %). $R_f$ = 0.5 (EtOAc:Hexane = 1:4); m.p. = 84-86 °C; $^1$H NMR (300 MHz, $CDCl_3$): δ 7.36-7.40 (m, 2H), 7.23 (d, $J$ = 7.6 Hz, 1H), 7.11 (d, $J$ = 7.6 Hz, 2H), 5.07 (br.s, 1H, -NH), 4.17 (d, $J$ = 5.5 Hz, 2H), 1.47 (s, 9H); $^{13}C\{^1H\}$ NMR (75 MHz, $CDCl_3$): δ 69.2, 155.9, 150.5, 129.6, 126.2, 121.4, 80.4, 42.8, 28.4; HRMS (ESI-FT) m/z: $[M + H]^+$ Calcd for $C_{13}H_{18}NO_4$ 252.1230; Found 252.1231; IR ($\nu_{max}$, solid): 3323, 2978, 2937, 1772, 1764, 1705, 1684, 1593, 1539, 1493, 1457, 1414, 1394, 1367, 1278, 1252, 1203, 1145, 1054, 955, 864, 818, 790, 770, 713, 689, 642, 549, 497.

**Synthesis of *N*-Protected Glycine Methyl Ester Derivatives 4a-4d:**

**Cbz-Gly-OMe (4a).** To a 100 mL round-bottomed flask with a magnetic stir bar was added sodium bicarbonate powder (2.00 g, 23.90 mmol) which was dissolved with distilled water (10 mL). Next, glycine methyl ester hydrochloride (1.50 g, 11.95 mmol) in distilled water (5 mL) and benzyl chloroformate (CbzCl, 2.3 mL, 15.53 mmol) were added subsequently at 0 °C. The reaction was

returned to room temperature and stirred for 12 hours. After this time, the mixture was transferred to a separatory funnel then extracted with ethyl acetate (30 mL × 3). The combined organic layer was dried over $MgSO_4$, filtered, and concentrated under reduced pressure to obtain a residue which was purified by flash column chromatography to yield **4a** as a colorless liquid (2.75 g, 97 %). The spectroscopic data is identical to the reported in the literature.[7] $R_f = 0.2$ (EtOAc:Hexane = 1:4); $^1$H NMR (300 MHz, $CDCl_3$): δ 7.31-7.35 (m, 5H), 5.26 (br.s, 1H, -NH), 5.13 (s, 2H), 3.99 (d, $J$ = 5.4 Hz, 2H), 3.76 (s, 3H).

**Fmoc-Gly-OMe (4b).** To a 500 mL round-bottomed flask with a magnetic stir bar was added glycine methyl ester hydrochloride (1.00 g, 7.96 mmol) which was dissolved with acetonitrile (80 mL) and distilled water (80 mL). Fmoc-OSu (2.96 g, 8.76 mmol) and potassium carbonate (4.40 g, 31.86 mmol) were added subsequently. The mixture was stirred for 2 hours. After 2 hours, the organic solvent was removed under reduced pressure, and the aqueous solution was transferred to a separatory funnel then extracted with diethyl ether (80 mL × 3). The combined organic layer was washed sequentially with 1 M $HCl_{(aq.)}$ (200 mL) and brine (200 mL) then dried over $MgSO_4$, filtered, and concentrated under reduced pressure to obtain a residue which was purified by flash column chromatography to yield **4b** as a white solid (2.10 g, 85 %). The spectroscopic data is identical to the reported in the literature.[8] $Rf = 0.2$ (EtOAc:Hexane = 1:4); $^1$H NMR (300 MHz, $CDCl_3$): δ 7.77 (d, $J$ = 7.8 Hz, 2H), 7.60 (d, $J$ = 7.8 Hz, 2H), 7.41 (t, $J$ = 7.3 Hz, 2H), 7.32 (td, $J$ = 7.5, 0.9 Hz, 2H), 5.30 (br.s, 1H, -NH), 4.42 (d, $J$ = 6.9 Hz, 2H), 4.24 (t, $J$ = 7.1 Hz, 1H), 4.01 (d, $J$ = 5.5 Hz, 2H), 3.77 (s, 3H).

**Ac-Gly-OMe (4c).** To a 100 mL round-bottomed flask with a magnetic stir bar was added **Ac-Gly-OH** (1.00 g, 8.54 mmol) and dried on high vacuum. After 20 mins, the flask was flushed with nitrogen and added anhydrous methanol (10 mL) followed by thionyl chloride (0.75 mL, 10.25 mmol) dropwise at 0 °C. The reaction was returned to room temperature and stirred for 6 hours until TLC analysis revealed complete consumption of the starting material. The solvent was removed under reduced pressure and transferred to a separatory funnel with ethyl acetate (50 mL) and distilled water

(50 mL). The organic layer was collected and the aqueous layer was further extracted with ethyl acetate (50 mL). The combined organic layer was dried over $MgSO_4$, filtered, and concentrated under reduced pressure to obtain a residue which was purified by flash column chromatography to yield **4c** as a yellow liquid (0.43 g, 38 %). The spectroscopic data is identical to the reported in the literature.[9] $R_f$ = 0.3 (MeOH:DCM = 1:19); $^1$H NMR (300 MHz, $CDCl_3$): δ 6.13 (br.s, 1H, -NH), 4.03 (d, $J$ = 4.1 Hz, 2H), 3.75 (s, 3H), 2.03 (s, 3H).

**$(Bn)_2$-Gly-OMe (4d).** To a 100 mL two-necked flask containing a magnetic stir bar was added glycine methyl ester hydrochloride (1.00 g, 7.96 mmol) and dried on high vacuum for 10 mins. After this time, the flask was flushed with nitrogen and added anhydrous acetonitrile (20 mL) followed by potassium carbonate (2.75 g, 20.00 mmol) and the reaction was stirred for 12 hours. After 12 hours, the solvent was removed under reduced pressure. The crude was dissolved with ethyl acetate (50 mL) and transferred to a separatory funnel. The organic layer was washed sequentially with 1 M HCl $_{(aq.)}$ (50 mL × 3), saturated sodium bicarbonate solution (50 mL), and brine (50 mL). The organic layer was dried over $MgSO_4$, filtered, and concentrated under reduced pressure to obtain a residue which was purified by flash column chromatography to yield **4d**. as a colorless liquid (1.78 g, 83 %); $R_f$ = 0.7 (EtOAc:Hexane = 1:4); $^1$H NMR (300 MHz, $CDCl_3$): δ 7.38 (t, $J$ = 8.5 Hz, 4H), 7.33 (t, $J$ = 7.0 Hz, 4H), 7.26 (d, $J$ = 7.0 Hz, 2H), 3.82 (s, 4H), 3.69 (s, 3H), 3.31 (s, 2H); $^{13}$C{$^1$H} NMR (75 MHz, $CDCl_3$): δ 172.0, 139.1, 129.0, 128.4, 127.3, 57.9, 53.5, 51.3; HRMS (ESI-FT) m/z: $[M + H]^+$ Calcd for $C_{17}H_{20}NO_2$ 270.1489; Found 270.1490; IR ($\nu_{max}$, film): 3506, 3375, 3325, 3273, 3229, 3060, 2932, 2857, 1739, 1692, 1653, 1632, 1547, 1457, 1365, 1343, 1284, 1257, 1202, 1152, 1056, 942, 899, 868, 825, 780, 738, 698, 613, 593, 575, 534, 478, 457, 434.

**Synthesis of Methyl Ester Derivatives**

Methyl ester **7b**, **7f**, **7g**, 7h were purchased from commercial source and were used directly in reaction.

**General Procedure for the Synthesis of *N*-Boc Protected Amino Acid Methyl Ester 3a, 5a-5i, 5m-5p, 5s:** To a 100 mL two-necked flask containing a magnetic stir bar was dried on high vacuum for 30 mins then flushed with nitrogen and added anhydrous methanol (15 mL). Thionyl chloride (2.5 equiv.) was added slowly at 0 °C and the mixture was stirred for 10 mins before adding the corresponding amino acid (1.00 g, 1.0 equiv.). The reaction was returned to room temperature and stirred for 16 hours, after which the solvent was removed under reduced pressure to obtain a crude solid. The solid was washed with cold diethyl ether to obtain the amino acid methyl ester hydrochloride in quantitative yield. This compound (**H-Xaa-OMe · HCl**) was used directly in the next step without further purification. To another 100 mL round-bottomed flask containing a magnetic stir bar was added the corresponding amino acid methyl ester hydrochloride, **H-Xaa-OMe · HCl** (1.00 g, 1.0 equiv.) and dissolved in distilled water (5.00 mL). Di-*tert*-butyl dicarbonate ($Boc_2O$, 1.2 equiv.) in tetrahydrofuran (5.00 mL) was added to the reaction followed by slow addition of triethylamine (2.5 equiv.) at 0 °C. The reaction was stirred at room temperature for 24 hours, after which the solvent was removed under reduced pressure to obtain a crude. The crude was transferred to a separatory funnel with ethyl acetate (50 mL) and washed sequentially with 1 M HCl $_{(aq.)}$ (50 mL × 3), saturated sodium bicarbonate (50 mL), and brine (50 mL). The organic layer was dried over $MgSO_4$, filtered, and concentrated under reduced pressure to obtain a residue which was purified by flash column chromatography to yield *N*-Boc-protected amino acid methyl esters. The yields reported below are based on 1.0 g of **H-Xaa-OMe · HCl** as the limiting reagent. All spectroscopic data of the known compounds was identical to those reported in the literature.

**Boc-Gly-OMe (3a).**[10] Colorless liquid (1.30 g, 86 % from **H-Gly-OMe · HCl**); $R_f$ = 0.3 (EtOAc:Hexane = 1:9); $^1H$ NMR (300 MHz, $CDCl_3$): δ 4.99 (br.s, 1H, -NH), 3.92 (d, $J$ = 5.3 Hz, 2H), 3.75 (s, 3H,), 1.45 (br. s, 9H).

**Boc-Ala-OMe (5a).**[11] Colorless solid (1.35 g, 93 %); $R_f$ = 0.4 (EtOAc:Hexane = 1:9); $^1$H NMR (300 MHz, $CDCl_3$): δ 5.03 (br.s, 1H, -NH), 4.29-4.31 (m, 1H), 3.74 (s, 3H,), 1.44 (s, 9H), 1.38 (d, $J$ = 7.3 Hz, 3H).

**Boc-Val-OMe (5b).**[12] Yellow liquid (1.62 g, 82 %); $R_f$ = 0.5 (EtOAc:Hexane = 1:9); $^1$H NMR (300 MHz, $CDCl_3$): δ 5.02 (br.s, 1H, -NH), 4.23 (br.s, 1H), 3.74 (s, 3H), 2.04-2.20 (m, 1H), 1.45 (s, 9H), 0.96 (d, $J$ = 6.9 Hz, 3H), 0.89 (d, $J$ = 6.9 Hz, 3H).

**Boc-Leu-OMe (5c).**[13] Yellow liquid (1.68 g, 90 %); $R_f$ = 0.5 (EtOAc:Hexane = 1:9); $^1$H NMR (300 MHz, $CDCl_3$): δ 4.86-4.90 (m,1H, -NH), 4.30-4.32 (m, 1H), 3.73 (s, 3H), 1.65-1.74 (m, 2H), 1.47-1.52 (m, 1H), 1.44 (s, 9H), 0.94 (d, $J$ = 6.4 Hz, 3H), 0.93 (d, $J$ = 6.5 Hz, 3H).

**Boc-Ile-OMe (5d).**[13] Yellow liquid (1.70 g, 91 %); $R_f$ = 0.6 (EtOAc:Hexane = 1:9); $^1$H NMR (300 MHz, $CDCl_3$): δ 5.01-5.03 (m, 1H, -NH), 4.24-4.28 (m, 1H), 3.72 (s, 3H), 1.84 (br.s, 1H), 1.44 (s, 9H), 1.35-1.41 (m, 1H), 1.11-1.25 (m, 1H), 0.91 (d, $J$ = 6.8 Hz, 3H), 0.90 (t, $J$ = 7.3 Hz, 3H).

**Boc-βAla-OMe (5e).**[7] Colorless oil (1.93 g, 94 %); $R_f$ = 0.2 (EtOAc:Hexane = 1:9); $^1$H NMR (300 MHz, $CDCl_3$): δ 5.02 (br.s, 1H, -NH), 3.69 (s, 3H), 3.38 (td, $J$ = 6.2, 6.0 Hz, 2H), 2.52 (d, $J$ = 5.5 Hz), 1.42 (s, 9H).

**Boc-HomoAla-OMe (5f).**[14] Colorless oil (1.98 g, 94 %); $R_f$ = 0.5 (EtOAc:Hexane = 3:7); $^1$H NMR (300 MHz, $CDCl_3$): δ 4.90 (br.s, 1H, -NH), 4.01-4.05 (m, 1H), 3.68 (s, 3H), 2.43-2.56 (m, 2H), 1.43 (s, 9H), 1.20 (d, $J$ = 7.0 Hz, 3H).

**Boc-Phe-OMe (5g).**[15] Colorless liquid (1.64 g, 97 %); $R_f$ = 0.4 (EtOAc:Hexane = 1:9); $^1$H NMR (300 MHz, $CDCl_3$): δ 7.24-7.32 (m, 3H), 7.12 (d, $J$ = 6.5 Hz, 2H), 4.95-4.98 (m, 1H, -NH), 4.55-4.62 (m, 1H), 3.71 (s, 3H), 3.03-3.15 (m, 2H), 1.41(s, 9H).

**Boc-Met-OMe (5h).**[16] Yellow liquid (1.14 g, 86 %); $R_f$ = 0.3 (EtOAc:Hexane = 1:9); $^1$H NMR (300 MHz, $CDCl_3$): δ 5.10 (br.s, 1H, -NH), 4.40 (br.s, 1H), 3.75 (s, 3H), 2.53 (t, *J*=7.7 Hz, 2H), 2.14-2.18 (m, 1H), 2.09 (s, 3H) , 1.86-1.98 (m, 1H), 1.44 (s, 9H).

**Boc-Pro-OMe (5i).**[17] Colorless liquid (1.37 g, 99 %); $R_f$ = 0.3 (EtOAc:Hexane = 1:9); $^1$H NMR (300 MHz, $CDCl_3$): (mixture of rotamers) δ 4.32 (dd, *J*=8.9, 6.2 Hz, 0.4H), 4.22 (dd, *J*=9.2, 5.0 Hz, 0.6H), 3.72 (s, 3H), 3.37-3.59 (m, 2H), 2.16-2.28 (m, 1H), 1.80-1.98 (m, 3H), 1.46 (s, 4H), 1.41 (s, 5H).

**Boc-Trp-OMe (5m).**[18] White solid (1.48 g, 95 %); $R_f$ = 0.3 (EtOAc:Hexane = 3:7); $^1$H NMR (300 MHz, $CDCl_3$): δ 8.13 (br.s, 1H, -NH), 7.56 (d, *J* = 7.8 Hz, 1H), 7.35 (d, *J* = 8.1 Hz, 1H), 7.19 (ddd, *J* = 8.2, 8.0, 1.1 Hz, 1H), 7.00 (d, *J* = 3.0 Hz, 1H), 7.12 (ddd, *J* = 7.1, 7.1, 0.8 Hz, 1H), 5.07-5.09 (m, 1H, -NH), 4.62-4.68 (m, 1H), 3.68 (s, 3H), 3.29 (d, *J* = 4.9 Hz, 2H), 1.43 (s, 9H).

**Boc-Ser-OMe (5n).**[19] Colorless oil (0.82 g, 58 %); $R_f$ = 0.4 (EtOAc:Hexane = 2:3); $^1$H NMR (300 MHz, $CDCl_3$): δ 5.45 (br.s, 1H, -NH), 4.38 (br.s, 1H), 3.85-3.99 (m, 2H), 3.78 (s, 3H), 2.46 (t, *J* = 5.9 Hz, 1H, -OH), 1.45 (s, 9H).

**Boc-Thr-OMe (5o).**[20] Colorless oil (1.82 g, 88 %); $R_f$ = 0.2 (EtOAc:Hexane = 1:4); $^1$H NMR (300 MHz, $CDCl_3$): δ 5.32 (d, *J* = 7.3 Hz, 1H, -NH), 4.25-4.29 (m, 2H), 3.77 (s, 3H), 2.12 (d, *J* = 4.6 Hz, 1H, -OH), 1.45 (s, 9H), 1.25 (d, *J* = 6.4 Hz, 3H).

**Boc-Tyr-OMe (5p).**[21] Colorless oil (1.12 g, 88 %); $R_f$ = 0.5 (EtOAc:Hexane = 2:3); $^1$H NMR (300 MHz, $CDCl_3$): δ 6.93 (d, *J* = 8.9 Hz, 2H), 6.73 (d, *J* = 8.2 Hz, 2H), 5.04 (br.s, 1H, -NH), 4.47 (br.s, 1H), 3.68 (s, 3H), 2.93-2.97 (m, 2H), 2.13 (br.s, 1H, -OH), 1.38 (s, 9H).

**Boc-Phg-OMe (5s).**[22] Colorless oil (1.276 g, 97 %); $R_f$ = 0.7 (EtOAc:Hexane = 1:4); $^1$H NMR (300 MHz, $CDCl_3$): δ 7.31-7.37 (m, 5H), 5.53 (br.s, 1H, -NH), 5.32 (d, *J* = 7.0 Hz, 1H), 3.72 (s, 3H), 1.43 (s, 9H). Phg stands for phenylglycine.

**General Procedure for synthesizing the methyl ester from Boc-Xaa-OH or benzoic acid derivatives:** To a round-bottom flask containing a magnetic stir bar was added the corresponding *N*-Boc-protected amino acid derivative, **Boc-Xaa-OH** or benzoic acid derivative (1.00 g, 1.0 equiv.) and potassium carbonate (2.0 equiv.) which were dissolved in anhydrous DMF (10 mL). The mixture was stirred at 0 °C for 10 mins before adding methyl iodide (1.5 equiv.) dropwise. The reaction mixture was returned to room temperature and stirred for 16 hours. After this time, the reaction was transferred to a separatory funnel with saturated sodium bicarbonate solution (30 mL) and extracted with ethyl acetate (30 mL × 3). The combined organic layer was washed sequentially with distilled water (100 mL × 3), saturated sodium thiosulfate solution (100 mL) and brine (100 mL). The combined organic layer was dried over $MgSO_4$, filtered, and concentrated under reduced pressure to obtain a residue which was purified by flash column chromatography to yield the desired **5j**, **5k**, **5l**, **7a**, or **7c**-**7e**. The spectroscopic data of the known compounds is identical to those reported in the literature.

**Boc-Cys($^t$Bu)-OMe (5j).**[23] White solid (0.82 g, 68 %); $R_f$ = 0.6 (EtOAc:Hexane = 1:4); $^1$H NMR (300 MHz, $CDCl_3$): δ 5.32 (br.s, 1H, -NH), 4.55-4.57 (m, 1H), 3.75 (s, 3H), 2.96 (d, *J* = 4.7 Hz, 2H)), 1.44 (s, 9H),1.30 (s, 9H).

**Boc-Gln(Trt)-OMe (5k).**[24] White solid (1.00g, 97 %); $R_f$ = 0.2 (EtOAc:Hexane = 3:7); $^1$H NMR (300 MHz, $CD_3OD$): δ 7.18-7.29 (m, 15H), 4.13 (td, *J* = 5.0, 4.4 Hz, 1H), 3.70 (s, 3H), 2.36-2.50 (m, 2H), 1.98-2.10 (m, 1H), 1.74-1.87 (m, 1H), 1.44 (s, 9H).

**Boc-Asn(Trt)-OMe (5l).** White solid (1.01 g, 98 %); $R_f$= 0.2 (EtOAc:Hexane = 3:7); m.p. = 174-176 °C; $^1$H NMR (300 MHz, $CDCl_3$): δ 7.26 (t, *J* = 6.2 Hz, 9H), 7.15 (d, *J* = 6.6 Hz, 6H), 6.69 (br.s, 1H, -NH), 5.73-5.76 (m, 1H, -NH), 4.45-4.51 (m, 1H), 3.67 (s, 3H), 3.02 (dd, *J* = 15.7, 4.3 Hz, 1H), 2.79 (dd, *J* = 15.5, 4.4 Hz, 1H), 1.42 (s, 9H, (H1)); $^{13}$C{$^1$H} NMR (100 MHz, $CDCl_3$): δ 171.9, 169.4, 156.7, 144.4, 128.7, 128.0, 127.1, 79.8, 70.8, 52.6, 50.6, 38.7, 28.3; IR ($\nu_{max}$, solid): 3354, 3269, 2977,

1748, 1699, 1643, 1516, 1446, 1392, 1367, 1353, 1295, 1254, 1166, 1034, 993, 767, 751, 698, 625, 572; HRMS (ESI-FT) m/z: $[M+Na]^+$ Calcd for $C_{29}H_{32}N_2O_5Na$ 511.2203; Found 511.2202.

**Methyl 4-nitrobenzoate (7a):**[25] White solid (1.03 g, 95 %); $R_f$ = 0.6 (EtOAc:Hexane = 1:9); $^1$H NMR (300 MHz, $CDCl_3$): δ 8.29 (d, *J* = 8.8 Hz, 2H), 8.20 (d, *J* = 8.9 Hz, 2H), 3.98 (s, 3H).

**Methyl 4-iodobenzoate (7c):**[26] White solid (0.94 g, 89 %); $R_f$ = 0.7 (EtOAc:Hexane = 1:9); $^1$H NMR (300 MHz, $CDCl_3$): δ 7.80 (d, *J* = 8.6 Hz, 2H), 7.74 (d, *J* = 8.5 Hz, 2H), 3.91 (s, 3H).

**Methyl 3,4,5-trimethoxybenzoate (7d).**[25] White solid (1.04 g, 98 %); $R_f$ = 0.3 (EtOAc:Hexane = 1:9); $^1$H NMR (300 MHz, $CDCl_3$): δ 7.30 (s, 2H), 3.90 (s, 12H).

**Methyl 4-methoxybenzoate (7e).**[25] White solid (0.97 g, 89 %); $R_f$ = 0.6 (EtOAc:Hexane = 1:9); $^1$H NMR (300 MHz, $CDCl_3$): δ 7.99 (d, *J* = 8.8 Hz, 2H), 6.91 (d, *J* = 8.8 Hz, 2H), 3.88 (s, 3H), 3.85 (s, 3H).

**Synthesis of Boc-Ser(tBu)-OMe (5q) and Boc-Tyr(tBu)-OMe (5r):** To a 100 mL round-bottomed flask with a magnetic stir bar was added *O*-*tert*-butyl-L-serine or *O*-*tert*-butyl-L-tyrosine (0.50 g, 1.0 equiv.) and dissolved with distilled water (5 mL). Next, di-*tert*-butyl dicarbonate ($Boc_2O$, 1.2 equiv.) in tetrahydrofuran (5 mL) followed by triethylamine (1.5 equiv.) were added to the reaction and then stirred for 16 hours. After this time, the solvent was removed under reduced pressure to obtain the crude of *N*-Boc-protected *O*-*tert*-butyl-L-serine or *O*-*tert*-butyl-L-tyrosine. The crude was used directly in the next transformation without any purification. To the 100 mL round-bottomed flask containing a magnetic stir bar and the crude of *N*-Boc-protected *O*-*tert*-butyl-L-serine or *O*-*tert*-butyl-L-tyrosine was added *N,N*-dimethylformamide (DMF, 10 mL). Potassium carbonate (2.0 equiv.) and methyl iodide (1.6 equiv.) were added subsequently at 0 °C. The reaction was returned to room temperature and stirred for 18 hours. After this time, the reaction was transferred to a separatory funnel with ethyl acetate (150 mL) and distilled water (150 mL). The organic layer was collected and the aqueous layer was further extracted with ethyl acetate (150 mL × 2). The combined organic layer

was washed sequentially with saturated sodium bicarbonate (400 mL) and brine (400 mL). The organic layer was dried over $MgSO_4$, filtered, and concentrated under reduced pressure to obtain a residue which was purified by flash column chromatography to yield **5q** or **5r**. All spectroscopic data is identical to those reported in the literature.

**Boc-Ser(tBu)-OMe (5q):**[27] Colorless oil (0.95 g, 94 %); $R_f$ = 0.2 (EtOAc:Hexane = 1:9); $^1$H NMR (300 MHz, $CDCl_3$): δ 5.36 (d, *J* = 8.1 Hz, 1H, -NH), 4.36-4.39 (m, 1H), 3.79 (dd, *J* = 9.4, 2.9 Hz, 1H), 3.73 (s, 3H), 3.56 (dd, *J* = 8.8, 2.9 Hz, 1H), 1.45 (s, 9H), 1.13 (s, 9H).

**Boc-Tyr(tBu)-OMe (5r):**[28] White solid (0.66 g, 89 %); $R_f$ = 0.2 (EtOAc:Hexane = 1:9); $^1$H NMR (300 MHz, $CDCl_3$): δ 7.01 (d, *J* = 8.2 Hz, 2H), 6.90 (d, *J* = 8.6 Hz, 2H), 4.96 (d, *J* = 7.5 Hz, 1H, -NH), 4.49-4.60 (m, 1H), 3.68 (s, 3H), 2.95-3.09 (m, , 2H), 1.41 (s, 9H), 1.32 (s, 9H).

**Boc-Hse-OMe (5t).** To a 100 mL round-bottomed flask containing a magnetic stir bar was added L-homoserine (0.50 g, 4.20 mmol) dissolved in tetrahydrofuran (5 mL). Next, di-*tert*-butyl dicarbonate ($Boc_2O$, 1.10 g, 5.04 mmol) and sodium hydroxide solution (0.20 g, 5.04 mmol, dissolved in 5 mL of distilled water) were added. The reaction was stirred for 16 hours. After this time, the solvent was removed under reduced pressure to obtain a crude. The crude was transferred to a separatory funnel with distilled water (30 mL). The aqueous layer was washed with *n*-hexane (30 mL × 2) and then acidified with 12 M HCl $_{(aq.)}$ at 0 °C until pH value is 1-2. The acidified aqueous layer was extracted with ethyl acetate (50 mL × 3). The combined organic layer was dried over $MgSO_4$, filtered, and concentrated under reduced pressure to give the crude of *N*-Boc-protected homoserine derivative. This crude was used directly in the next reaction without any purification. To the 100 mL round-bottomed flask containing a magnetic stir bar and the crude of *N*-Boc-protected homoserine derivative was added *N, N*-dimethylformamide (DMF, 15 mL) and potassium carbonate (1.16 g, 8.39 mmol). The reaction was stirred for 10 mins before adding methyl iodide (0.42 mL, 6.72 mmol) dropwise at 0 °C. The reaction was returned to room temperature and stirred for 18 hours. After 18 hours, the

reaction was transferred to a separatory funnel with ethyl acetate (150 mL) and distilled water (150 mL). The organic layer was collected and the aqueous layer was further extracted with ethyl acetate (150 mL × 2). The combined organic layer was washed sequentially with saturated sodium bicarbonate solution (400 mL), saturated sodium thiosulfate (400 mL), and brine (400 mL). The organic layer was dried over $MgSO_4$, filtered, and concentrated under reduced pressure to obtain a residue which was purified by flash column chromatography to yield **5t** as a colorless oil (0.507 g, 52 %). The spectroscopic data is identical to those reported in the literature.[29]. $R_f$ = 0.3 (EtOAc:Hexane = 1:1); $^1$H NMR (300 MHz, $CDCl_3$): δ 5.37 (d, *J* = 5.8 Hz, 1H, -NH), 4.49 (td, *J* = 8.6, 3.3 Hz, 1H), 3.76 (s, 3H), 3.62-3.69 (m, 2H), 3.10 (br.s, 1H, -OH), 2.09-2.18 (m, 1H), 1.65 (br.s, 1H), 1.45 (s, 9H).

**Boc-AlaGly-OMe (5u).** To a 100 mL round-bottomed flask with a magnetic stir bar, L-alanine (1.00 g, 11.22 mmol) was added in distilled water (5 mL) and stirred at 0 °C. After 10 minutes, di-*tert*-butyl dicarbonate ($Boc_2O$, 2.94 g, 13.47 mmol) was dissolved in tetrahydrofuran (5 mL) and transferred to the stirred mixture at 0 °C. Then, triethylamine (1.88 mL, 13.47 mmol) was added slowly at 0 °C and then the stirred mixture was stirred at room temperature. After 16 hours, the solvent was removed under reduced pressure. The crude mixture was diluted with saturated sodium bicarbonate solution (30 mL) and washed with diethyl ether (30 mL × 2). The collected aqueous solution was added with 10 M $HCl_{(aq.)}$ until pH is 1 at 0 °C. The aqueous solution was extracted with ethyl acetate (3 × 50 mL). The collected organic layer was dried over $MgSO_4$, filtered, and concentrated under reduced pressure to give the crude *N*-Boc-protected alanine derivative used directly in the next reaction without any purification. To another two-neck flask containing a magnetic stir bar and the crude of *N*-Boc-protected alanine derivative, HOBt hydrate (1.82 g, 13.47 mmol) and TBTU (4.32 g, 13.47 mmol) were added and dried on high vacuum. After 1 hour, the round-bottomed flask was flushed with nitrogen and anhydrous dichloromethane (15 mL) was added to obtain the suspension. The suspension was cooled to 0 °C and keep stirred. After 10 mins, triethylamine (3.91 mL, 28.06 mmol)

and glycine methyl ester hydrochloride (2.11 g, 16.84 mmol) were added sequentially to the reaction mixture. The reaction mixture was returned at room temperature and stirred for 24 hours. After 24 hours, the solvent was removed under reduced pressure. The residue was dissolved with ethyl acetate (100 mL) then transferred to a separatory funnel and extracted with and 1 M HCl $_{(aq.)}$ (100 mL × 3). The organic layer was collected and washed with saturated sodium bicarbonate solution (100 mL) and brine (100 mL) sequentially. Then the organic layer was dried over $MgSO_4$, filtered, and concentrated under reduced pressure to obtain a residue which was purified by flash column chromatography to yield **5u** as a colorless liquid (2.16 g, 74 %). The spectroscopic data was identical to those reported in the literature.[30] $R_f$ = 0.3 (EtOAc:Hexane = 1:1); $^1H$ NMR (300 MHz, $CDCl_3$): δ 6.65 (br.s, 1H, -NH), 4.96 (br.s, 1H, -NH), 4.19-4.23 (m, 1H), 4.03-4.06 (m, 2H), 3.76 (s, 3H), 1.45 (s, 9H), 1.38 (d, *J* = 7.1 Hz, 3H).

**Table S1. Attempted direct amidation with *O*-protected Serine and other N-terminal amino acids that do not contain hydroxyl side chain.**

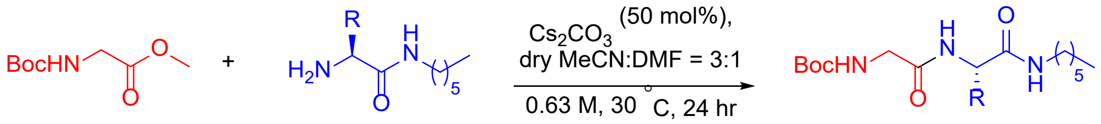


| Entry | Nucleophile | Expected Product | % Yield |
|---|---|---|---|
| 1 | H-Ser($^t$Bu)-NHHex | Boc-GlySer($^t$Bu)-NHHex | n.r |
| 2 | H-Ala-NHHex | Boc-GlyAla-NHHex | n.r. |
| 3 | H-βAla-NHHex | Boc-GlyβAla-NHHex | n.r. |
| 4 | H-Gly-NHHex | Boc-GlyGly-NHHex | n.r. |
| 5 | H-HomoAla-NHHex | Boc-GlyHomoAla-NHHex | n.r. |

n.r. = no reaction

$^{1}$H NMR Spectrum of **1b** (300 MHz, $CDCl_3$)

```
Current Data Parameters
NAME      20210308 Boc-Thr-Hex
EXPNO                    1
PROCNO                   1

F2 - Acquisition Parameters
Date_            20210308
Time                16.53 h
INSTRUM             spect
PROBHD   Z104275_0120 (
PULPROG              zg30
TD                  16384
SOLVENT             CDCl3
NS                     12
DS                      0
SWH              4807.692 Hz
FIDRES           0.586877 Hz
AQ              1.7039360 sec
RG                    128
DW                104.000 usec
DE                   6.50 usec
TE                  300.0 K
D1             2.00000000 sec
TD0                     1
SFO1          300.1321009 MHz
NUC1                   1H
P1                  15.00 usec
PLW1           5.69999981 W

F2 - Processing parameters
SI                   8192
SF            300.1300065 MHz
WDW                    EM
SSB      0
LB       0 Hz
GB       0
PC                   1.00
```

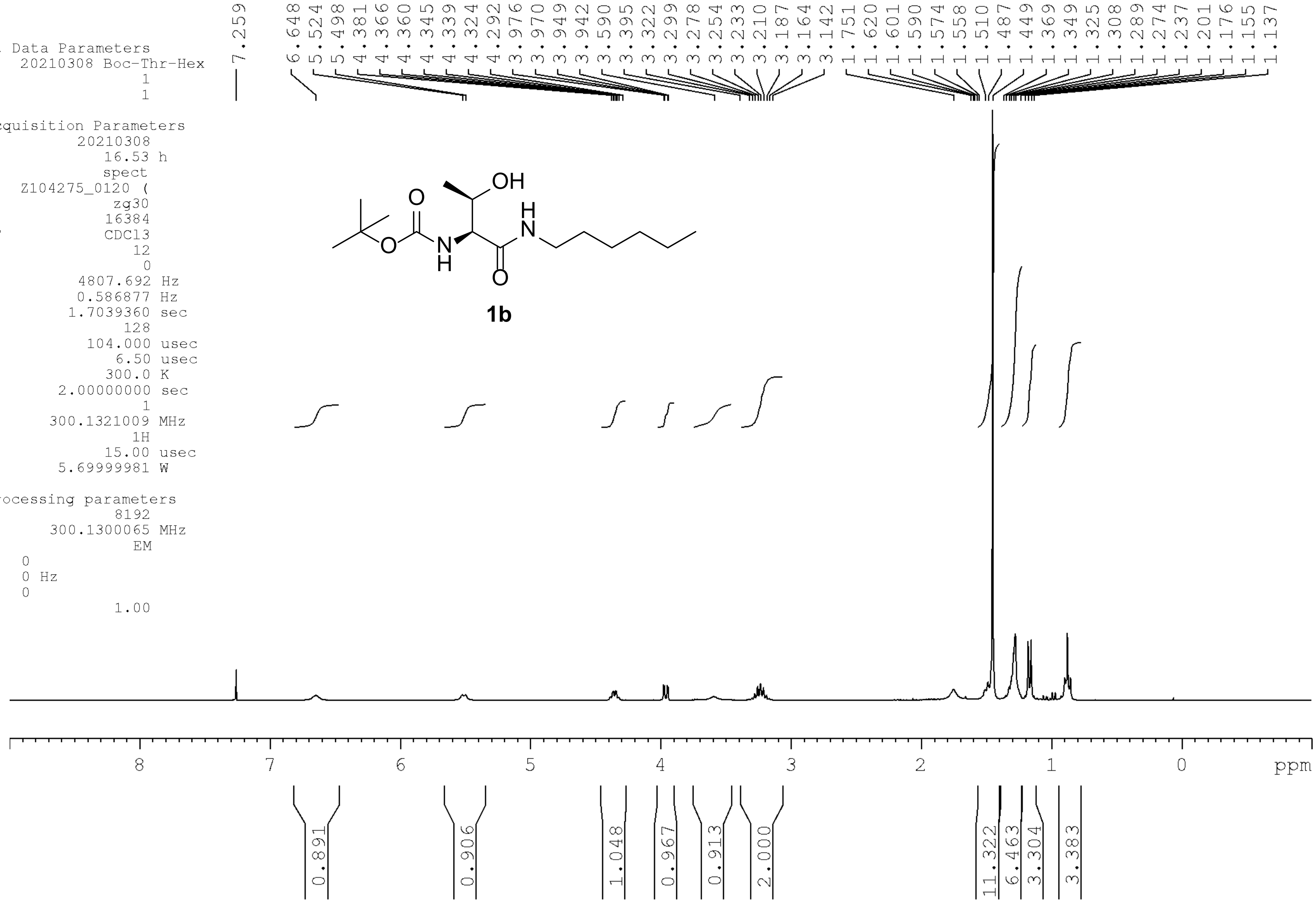

$^{13}C\{^{1}H\}$ NMR Spectrum of **1b** (75 MHz, $CDCl_3$)

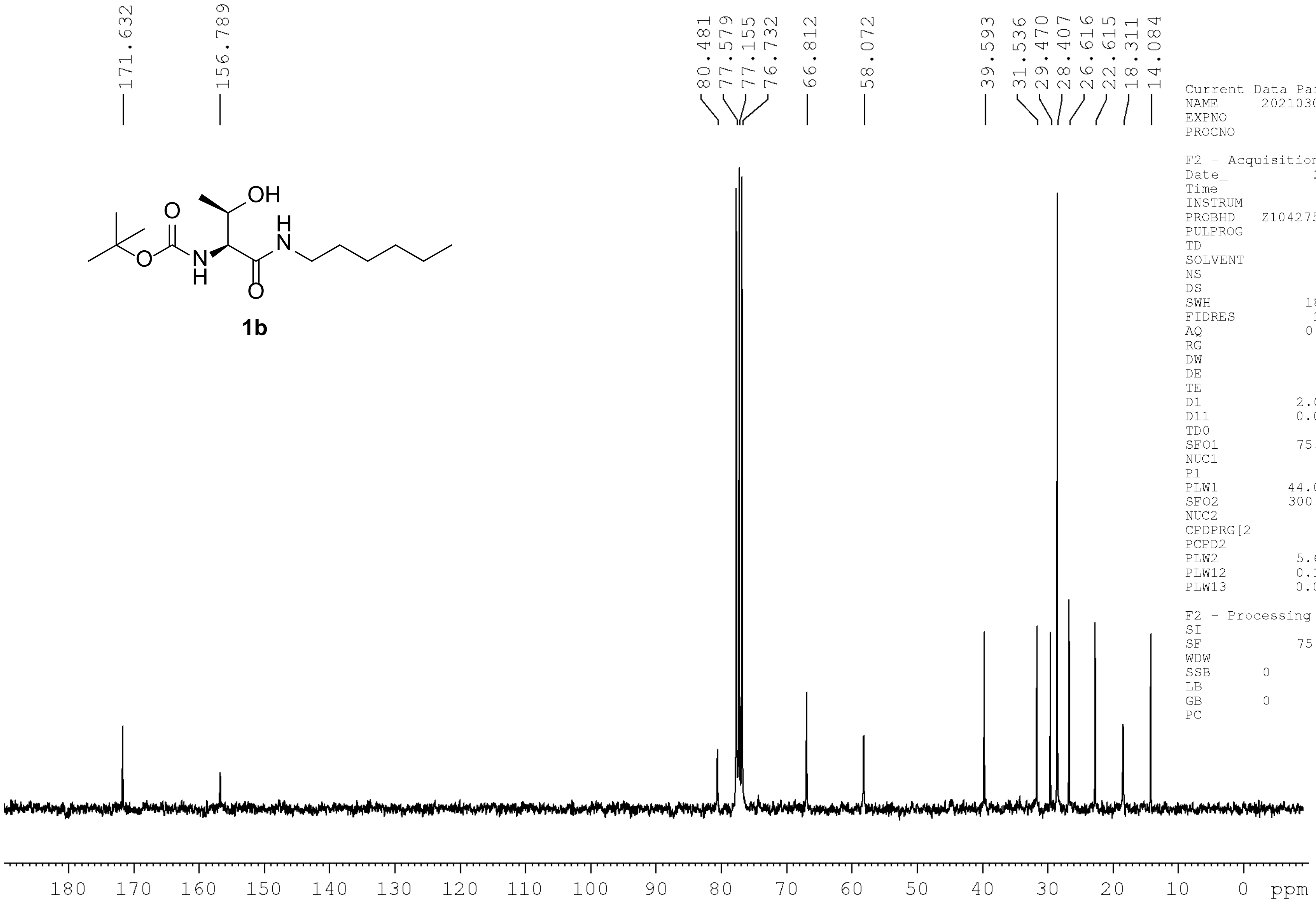


Current Data Parameters
NAME 20210309 Boc-Thr-Hex-C
EXPNO 1
PROCNO 1

F2 - Acquisition Parameters
Date_ 20210309
Time 9.25 h
INSTRUM spect
PROBHD Z104275_0120 (
PULPROG zgpg30
TD 32768
SOLVENT CDCl3
NS 460
DS 0
SWH 18028.846 Hz
FIDRES 1.100393 Hz
AQ 0.9087659 sec
RG 2050
DW 27.733 usec
DE 6.50 usec
TE 300.0 K
D1 2.00000000 sec
D11 0.03000000 sec
TD0 1
SFO1 75.4760505 MHz
NUC1 13C
P1 10.00 usec
PLW1 44.00000000 W
SFO2 300.1312005 MHz
NUC2 1H
CPDPRG[2 waltz16
PCPD2 90.00 usec
PLW2 5.69999981 W
PLW12 0.15832999 W
PLW13 0.07964100 W

F2 - Processing parameters
SI 16384
SF 75.4677383 MHz
WDW EM
SSB 0
LB 3.00 Hz
GB 0
PC 1.00

$^{1}$H NMR Spectrum of **1c** (300 MHz, $CDCl_3$)

```
Current Data Parameters
NAME     20210412 fmoc-ser(tbu)-hex 1
EXPNO                  1
PROCNO                 1

F2 - Acquisition Parameters
Date_          20210412
Time              12.46 h
INSTRUM           spect
PROBHD   Z104275_0120 (
PULPROG            zg30
TD                16384
SOLVENT           CDCl3
NS                   16
DS                    0
SWH            4807.692 Hz
FIDRES         0.586877 Hz
AQ            1.7039360 sec
RG                  144
DW              104.000 usec
DE                 6.50 usec
TE                300.0 K
D1           2.00000000 sec
TD0                   1
SFO1        300.1321009 MHz
NUC1                 1H
P1                15.00 usec
PLW1         5.69999981 W

F2 - Processing parameters
SI                 8192
SF          300.1300064 MHz
WDW                  EM
SSB      0
LB       0 Hz
GB       0
PC                 1.00
```

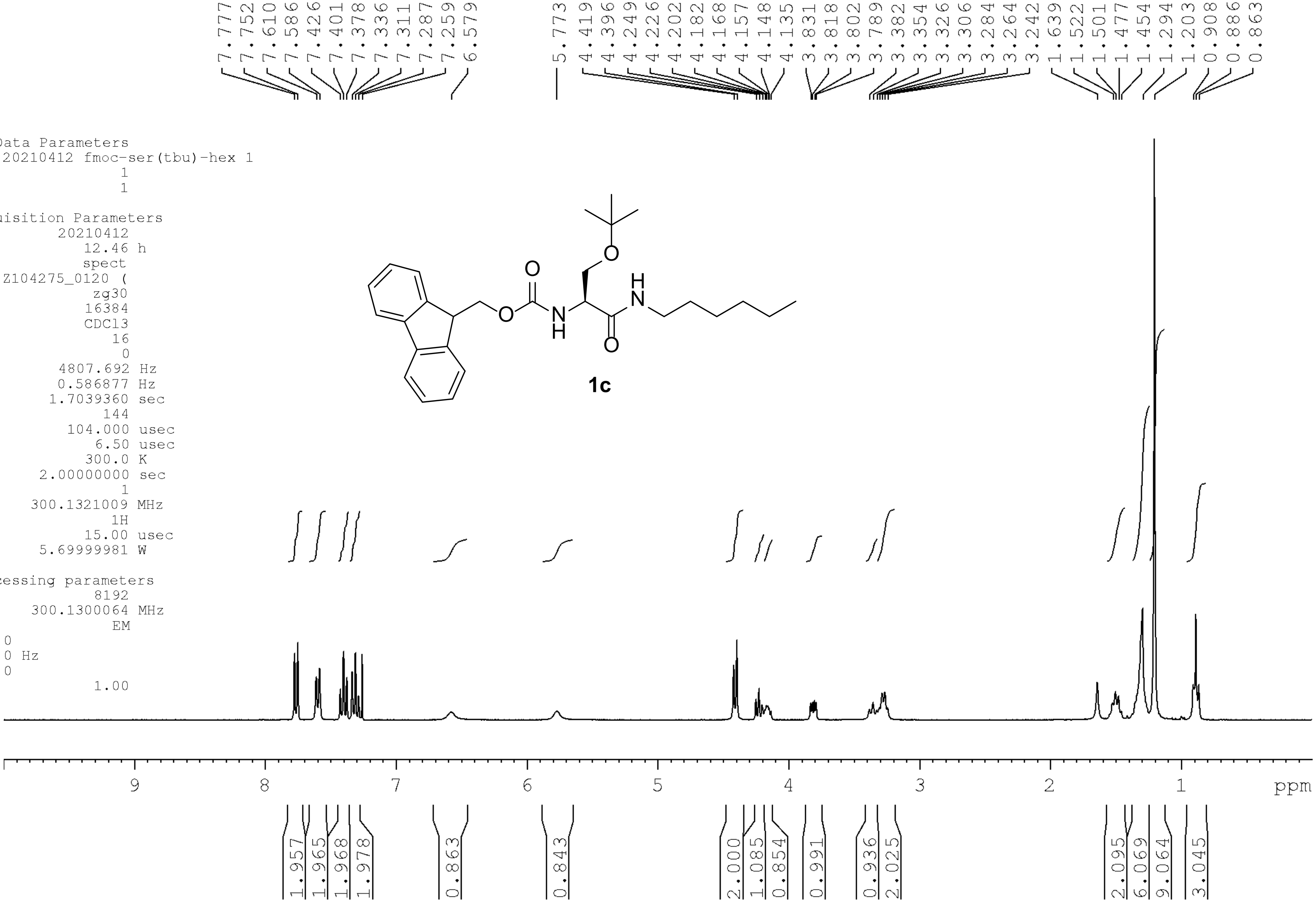

## $^{13}C\{^1H\}$ NMR Spectrum of **1c** (100 MHz, $CDCl_3$)

| | |
|---|---|
| Filename | = fmoc-ser(tbu)-hex. |
| Author | = YYL |
| Experiment | = carbon.jxp |
| Sample_Id | = fmoc-ser(tbu)-hex |
| Solvent | = CHLOROFORM-D |
| Actual_Start_Time | = 16-APR-2021 11:42: |
| Revision_Time | = 1-MAY-2021 22:43: |
| Comment | = single pulse decou |
| Data_Format | = 1D COMPLEX |
| Dim_Size | = 26214 |
| X_Domain | = Carbon13 |
| Dim_Title | = Carbon13 |
| Dim_Units | = [ppm] |
| Dimensions | = X |
| Spectrometer | = JNM-ECZ400S/L1 |
| Field_Strength | = 9.389766[T] (400[M |
| X_Acq_Duration | = 1.03809024[s] |
| X_Domain | = 13C |
| X_Freq | = 100.52530333[MHz] |
| X_Offset | = 100[ppm] |
| X_Points | = 32768 |
| X_Prescans | = 4 |
| X_Resolution | = 0.96330739[Hz] |
| X_Sweep | = 31.56565657[kHz] |
| X_Sweep_Clipped | = 25.25252525[kHz] |
| Irr_Domain | = Proton |
| Irr_Freq | = 399.78219838[MHz] |
| Irr_Offset | = 5[ppm] |
| Clipped | = FALSE |
| Scans | = 1024 |
| Total_Scans | = 1024 |
| Relaxation_Delay | = 2[s] |
| Recvr_Gain | = 50 |
| Temp_Get | = 22.4[dC] |
| X_90_Width | = 10.3[us] |
| X_Acq_Time | = 1.03809024[s] |
| X_Angle | = 30[deg] |
| X_Atn | = 7[dB] |
| X_Pulse | = 3.43333333[us] |
| Irr_Atn_Dec | = 26.116[dB] |
| Irr_Atn_Dec_Calc | = 26.116[dB] |
| Irr_Atn_Dec_Default_Calc | = 26.116[dB] |
| Irr_Atn_Noe | = 26.116[dB] |
| Irr_Dec_Bandwidth_Hz | = 4.7826087[kHz] |
| Irr_Dec_Bandwidth_Ppm | = 11.96303566[ppm] |
| Irr_Dec_Freq | = 399.78219838[MHz] |
| Irr_Dec_Merit_Factor | = 2.2 |
| Irr_Decoupling | = TRUE |
| Irr_Noe | = TRUE |
| Irr_Noise | = WALTZ |
| Irr_Offset_Default | = 5[ppm] |
| Irr_Pwidth | = 0.115[ms] |
| Irr_Pwidth_Default | = 0.115[ms] |
| Irr_Pwidth_Default_Calc | = 0.115[ms] |
| Irr_Pwidth_Temp1 | = 0.115[ms] |
| Irr_Wurst | = FALSE |
| Decimation_Rate | = 0 |
| Initial_Wait | = 1[s] |
| Noe_Time | = 2[s] |
| Noe_Time_Flag | = FALSE |
| Relaxation_Delay_Calc | = 0[s] |
| Relaxation_Delay_Temp | = 2[s] |
| Repetition_Time | = 3.03809024[s] |

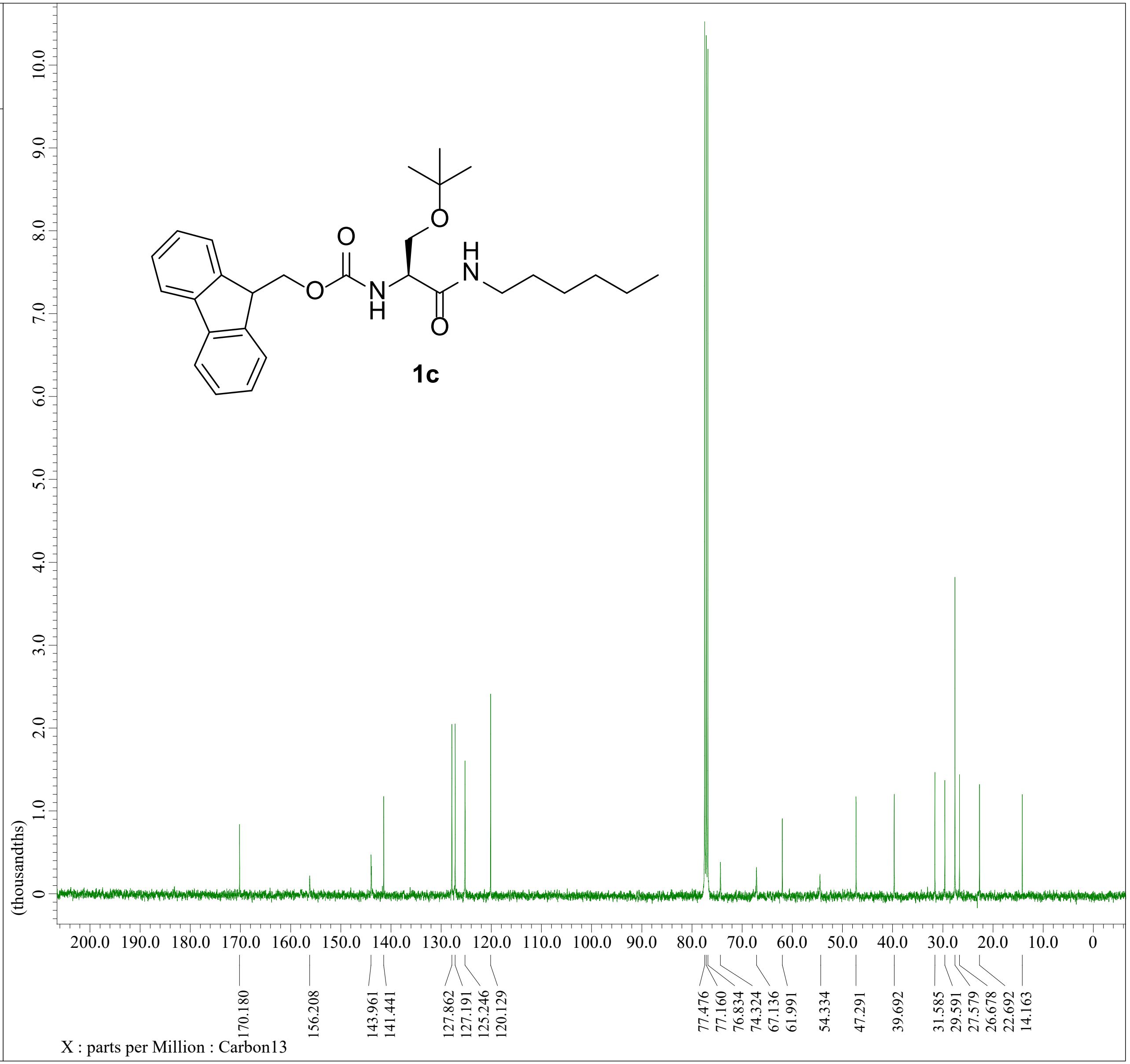

$^{1}$H NMR Spectrum of **1d** (300 MHz, $CDCl_3$)

```
Current Data Parameters
NAME     20201117 boc-ser-ala-hex m
EXPNO                  1
PROCNO                 1

F2 - Acquisition Parameters
Date_           20201117
Time               14.04
INSTRUM            spect
PROBHD   5 mm PABBO BB-
PULPROG             zg30
TD                 16384
SOLVENT            CDCl3
NS                    16
DS                     0
SWH             4807.692 Hz
FIDRES          0.293438 Hz
AQ             1.7039360 sec
RG                   144
DW               104.000 usec
DE                  6.50 usec
TE                 300.0 K
D1            2.00000000 sec
TD0                    1

======== CHANNEL f1 ========
NUC1                  1H
P1                 10.80 usec
PL1                -1.00 dB
PL1W         10.11928844 W
SFO1         300.1321009 MHz

F2 - Processing parameters
SI                  8192
SF           300.1300062 MHz
WDW                   EM
SSB                    0
LB                     0 Hz
GB                     0
PC                  1.00
```

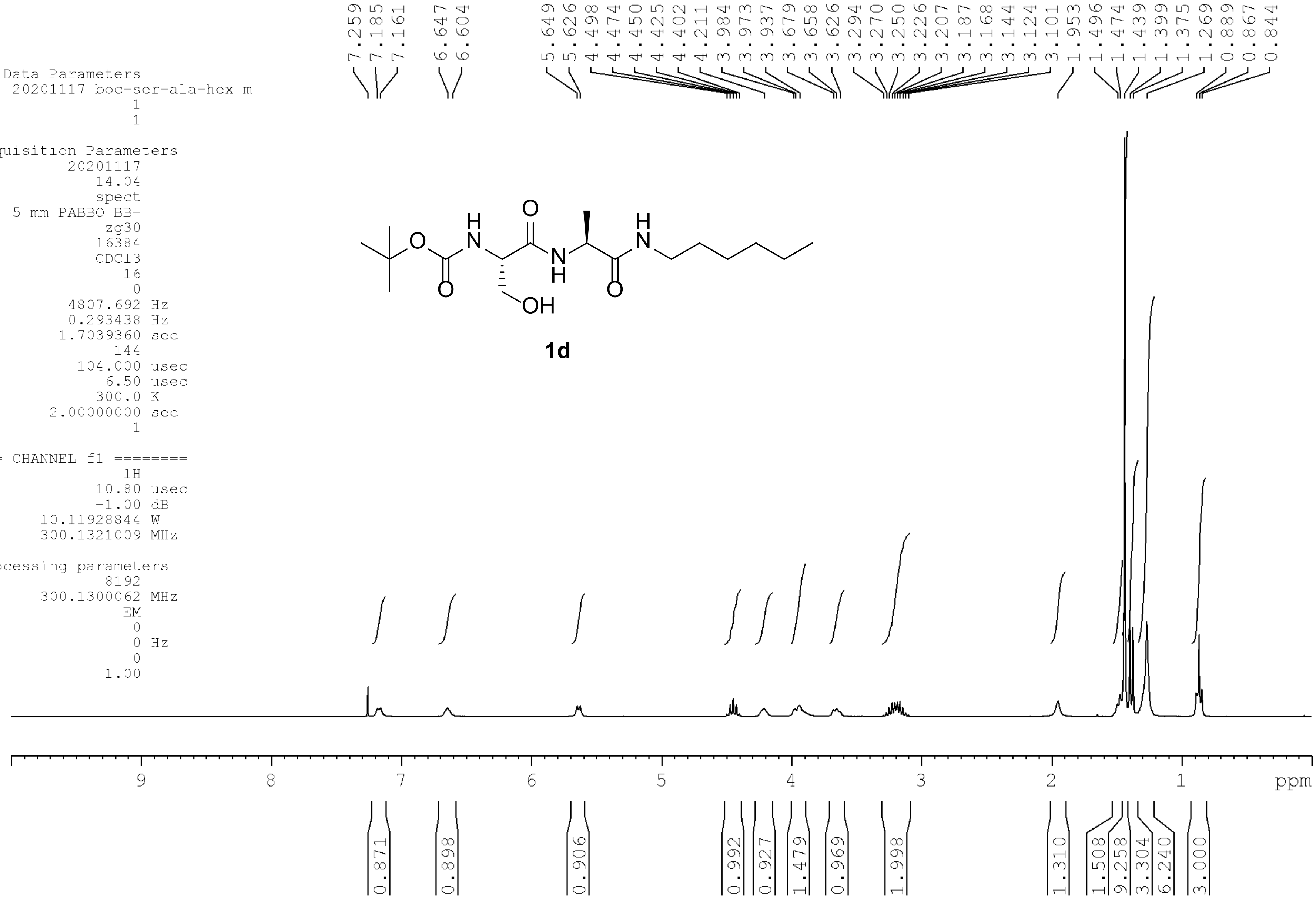

## $^{13}C\{^{1}H\}$ NMR Spectrum of **1d** (100 MHz, $CDCl_3$)

```
Filename                   = boc-ser-ala-hex.jd
Author                     = YYL
Experiment                 = carbon.jxp
Sample_Id                  = BOC-ser-ala-hex
Solvent                    = CHLOROFORM-D
Actual_Start_Time          = 16-APR-2021 10:45:
Revision_Time              =  1-MAY-2021 21:39:

Comment                    = single pulse decou
Data_Format                = 1D COMPLEX
Dim_Size                   = 26214
X_Domain                   = Carbon13
Dim_Title                  = Carbon13
Dim_Units                  = [ppm]
Dimensions                 = X
Spectrometer               = JNM-ECZ400S/L1

Field_Strength             = 9.389766[T] (400[M
X_Acq_Duration             = 1.03809024[s]
X_Domain                   = 13C
X_Freq                     = 100.52530333[MHz]
X_Offset                   = 100[ppm]
X_Points                   = 32768
X_Prescans                 = 4
X_Resolution               = 0.96330739[Hz]
X_Sweep                    = 31.56565657[kHz]
X_Sweep_Clipped            = 25.25252525[kHz]
Irr_Domain                 = Proton
Irr_Freq                   = 399.78219838[MHz]
Irr_Offset                 = 5[ppm]
Clipped                    = FALSE
Scans                      = 1024
Total_Scans                = 1024

Relaxation_Delay           = 2[s]
Recvr_Gain                 = 50
Temp_Get                   = 22.5[dC]
X_90_Width                 = 10.3[us]
X_Acq_Time                 = 1.03809024[s]
X_Angle                    = 30[deg]
X_Atn                      = 7[dB]
X_Pulse                    = 3.43333333[us]
Irr_Atn_Dec                = 26.116[dB]
Irr_Atn_Dec_Calc           = 26.116[dB]
Irr_Atn_Dec_Default_Calc   = 26.116[dB]
Irr_Atn_Noe                = 26.116[dB]
Irr_Dec_Bandwidth_Hz       = 4.7826087[kHz]
Irr_Dec_Bandwidth_Ppm      = 11.96303566[ppm]
Irr_Dec_Freq               = 399.78219838[MHz]
Irr_Dec_Merit_Factor       = 2.2
Irr_Decoupling             = TRUE
Irr_Noe                    = TRUE
Irr_Noise                  = WALTZ
Irr_Offset_Default         = 5[ppm]
Irr_Pwidth                 = 0.115[ms]
Irr_Pwidth_Default         = 0.115[ms]
Irr_Pwidth_Default_Calc    = 0.115[ms]
Irr_Pwidth_Temp1           = 0.115[ms]
Irr_Wurst                  = FALSE
Decimation_Rate            = 0
Initial_Wait               = 1[s]
Noe_Time                   = 2[s]
Noe_Time_Flag              = FALSE
Relaxation_Delay_Calc      = 0[s]
Relaxation_Delay_Temp      = 2[s]
Repetition_Time            = 3.03809024[s]
```

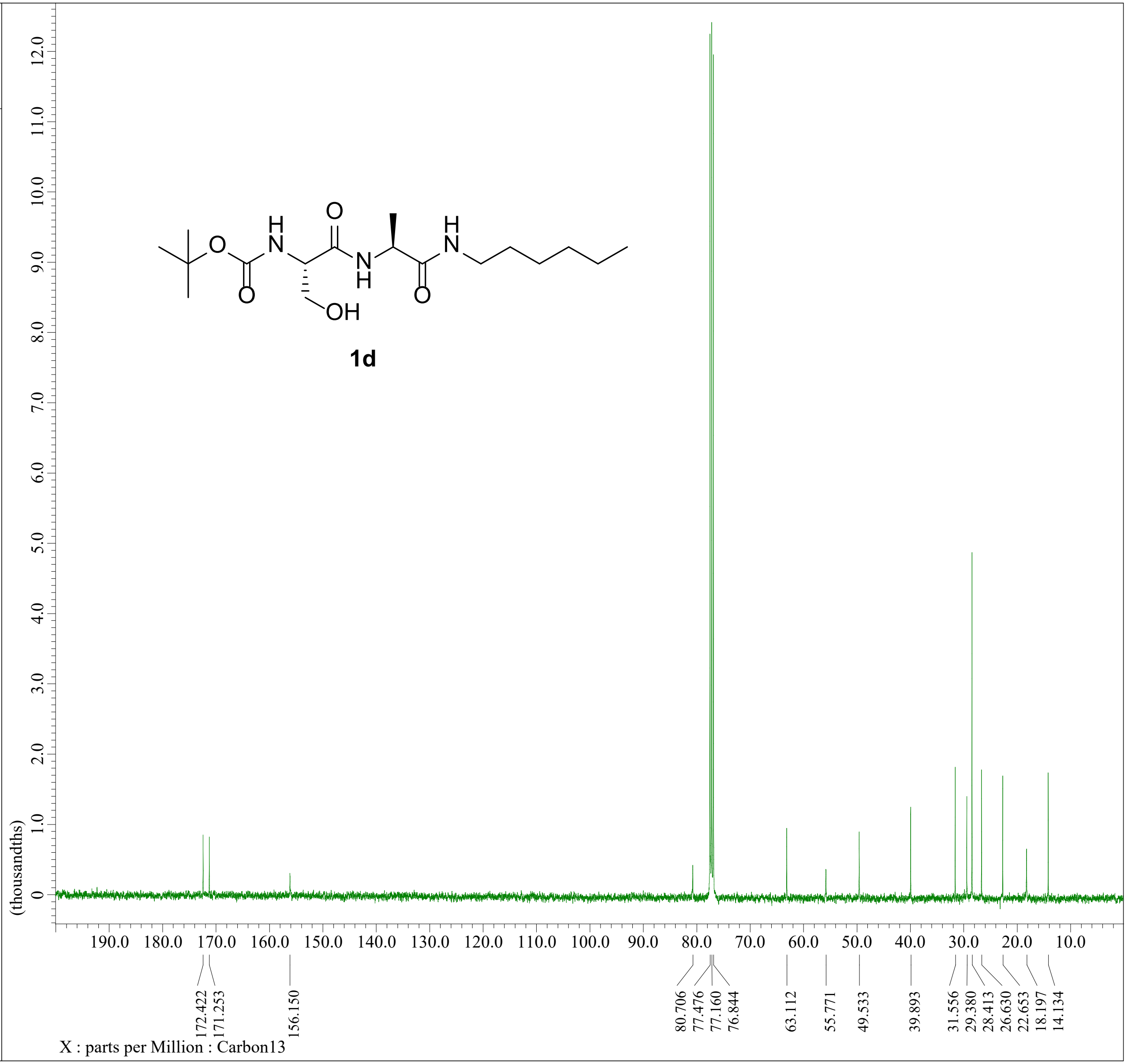

$^{1}$H NMR Spectrum of **2a** (300 MHz, $CDCl_3$)

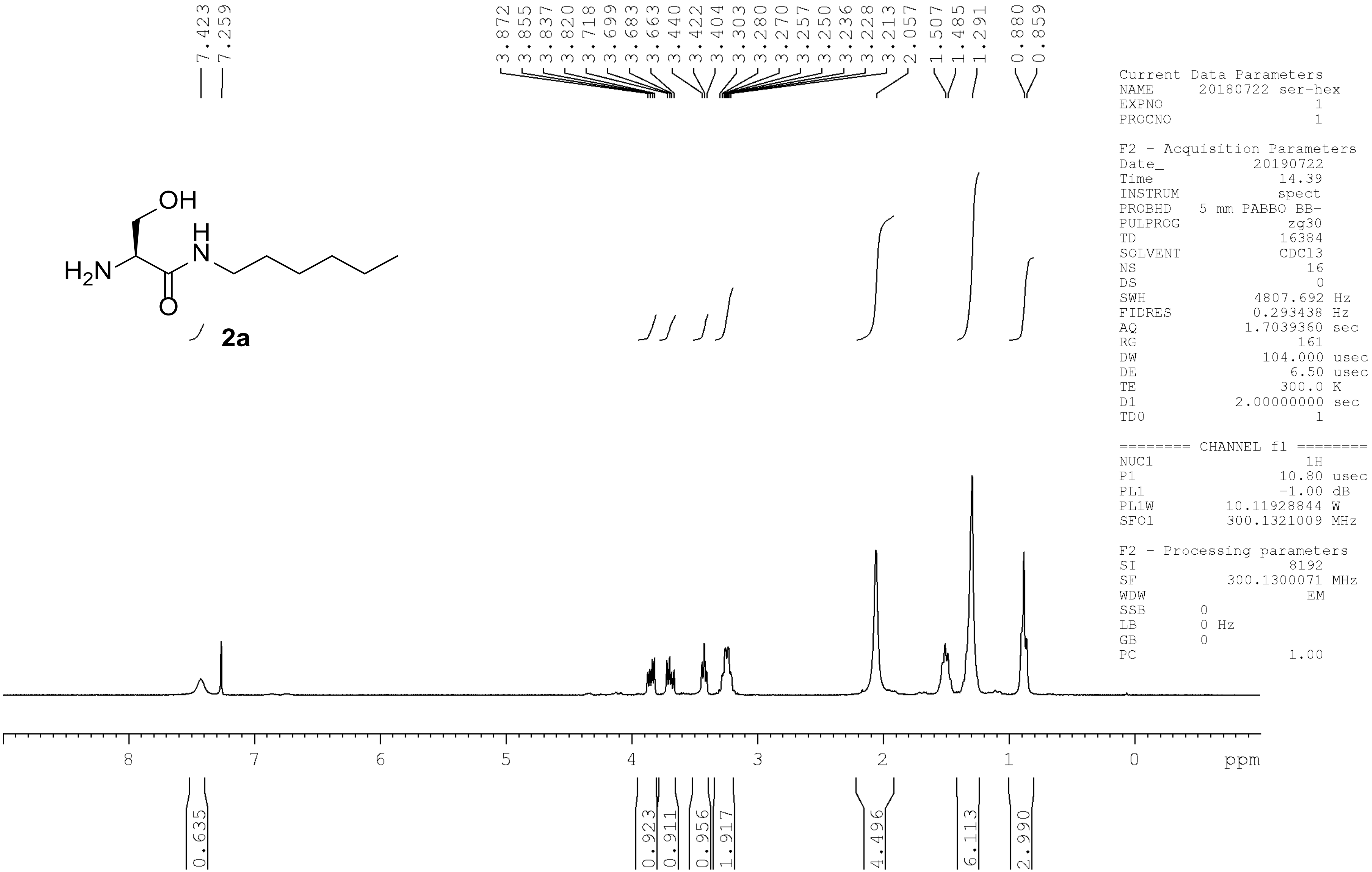

$^{13}C\{^{1}H\}$ NMR Spectrum of **2a** (75 MHz, $CDCl_3$)

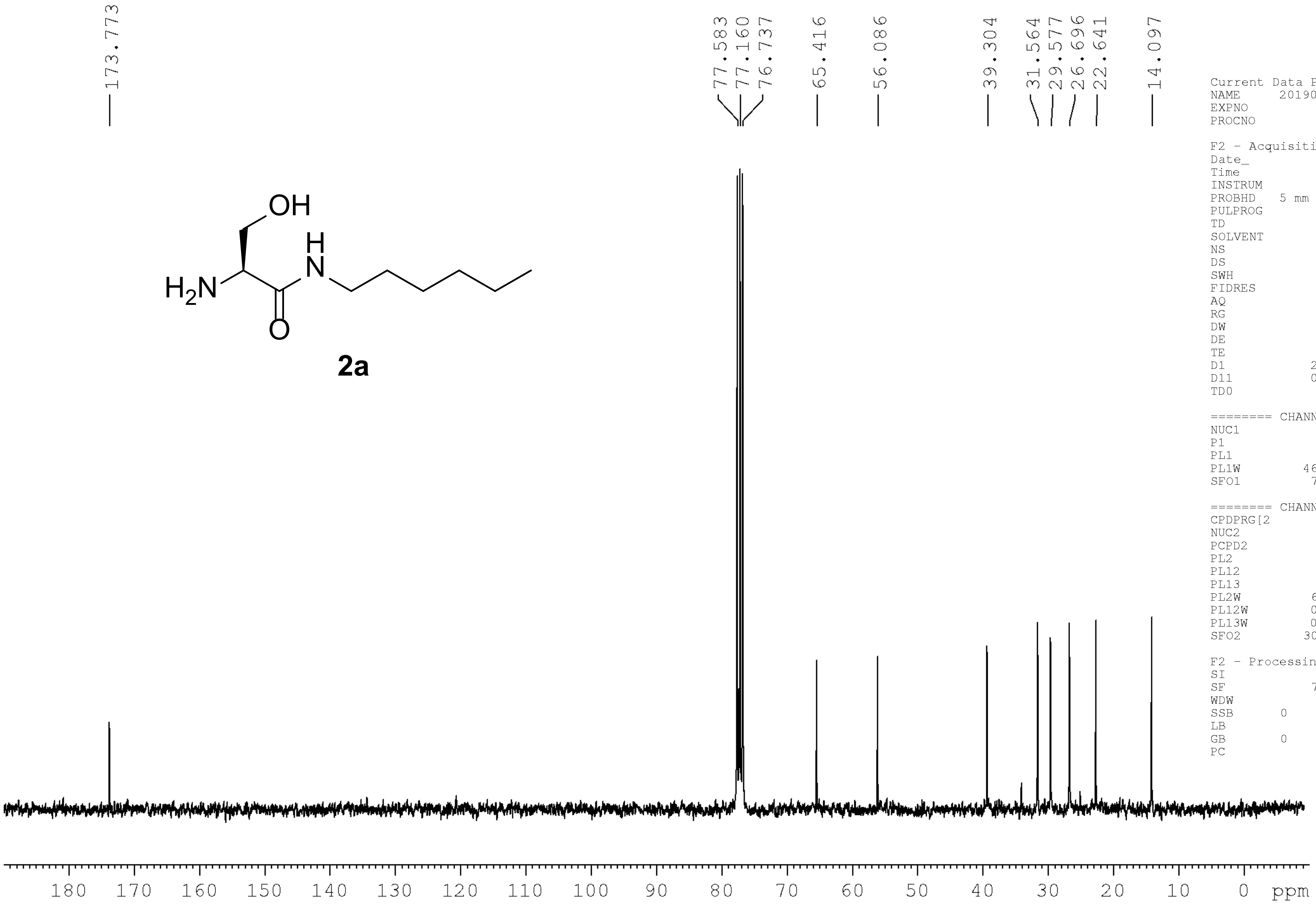


Current Data Parameters
NAME 20190917ser-hex-C
EXPNO 1
PROCNO 1

F2 - Acquisition Parameters
Date_ 20190917
Time 17.58
INSTRUM spect
PROBHD 5 mm PABBO BB-
PULPROG zgpg30
TD 32768
SOLVENT CDCl3
NS 346
DS 0
SWH 18028.846 Hz
FIDRES 0.550197 Hz
AQ 0.9087659 sec
RG 2050
DW 27.733 usec
DE 6.50 usec
TE 300.0 K
D1 2.00000000 sec
D11 0.03000000 sec
TD0 1

======== CHANNEL f1 ========
NUC1 13C
P1 9.50 usec
PL1 -1.00 dB
PL1W 46.16925430 W
SFO1 75.4760505 MHz

======== CHANNEL f2 ========
CPDPRG[2 waltz16
NUC2 1H
PCPD2 90.00 usec
PL2 1.00 dB
PL12 17.29 dB
PL13 22.00 dB
PL2W 6.38483953 W
PL12W 0.15002026 W
PL13W 0.05071658 W
SFO2 300.1312005 MHz

F2 - Processing parameters
SI 16384
SF 75.4677389 MHz
WDW EM
SSB 0
LB 3.00 Hz
GB 0
PC 1.00

$^{1}$H NMR Spectrum of **2b** (300 MHz, $CDCl_3$)

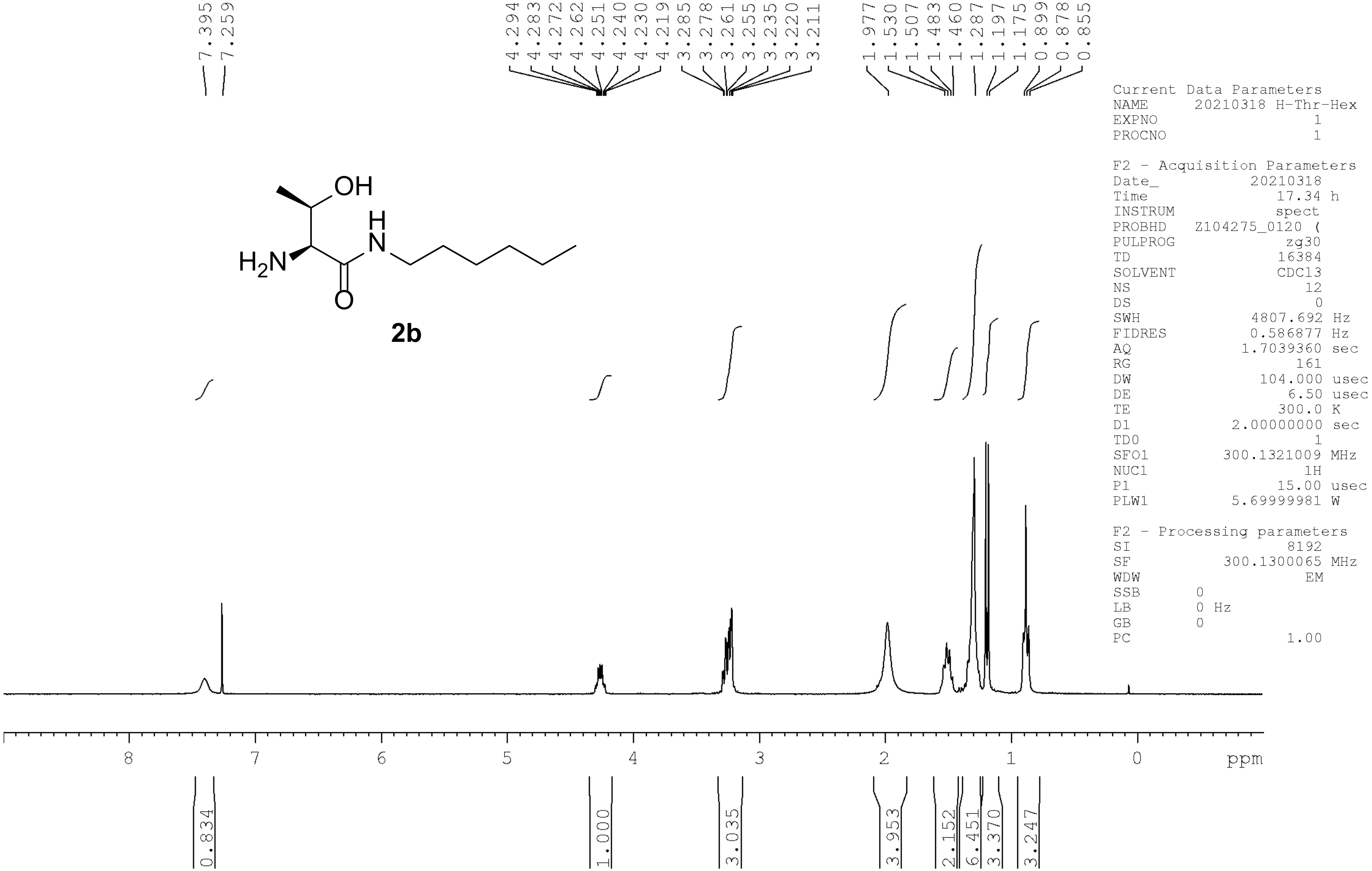

$^{13}C\{^{1}H\}$ NMR Spectrum of **2b** (75 MHz, $CDCl_3$)

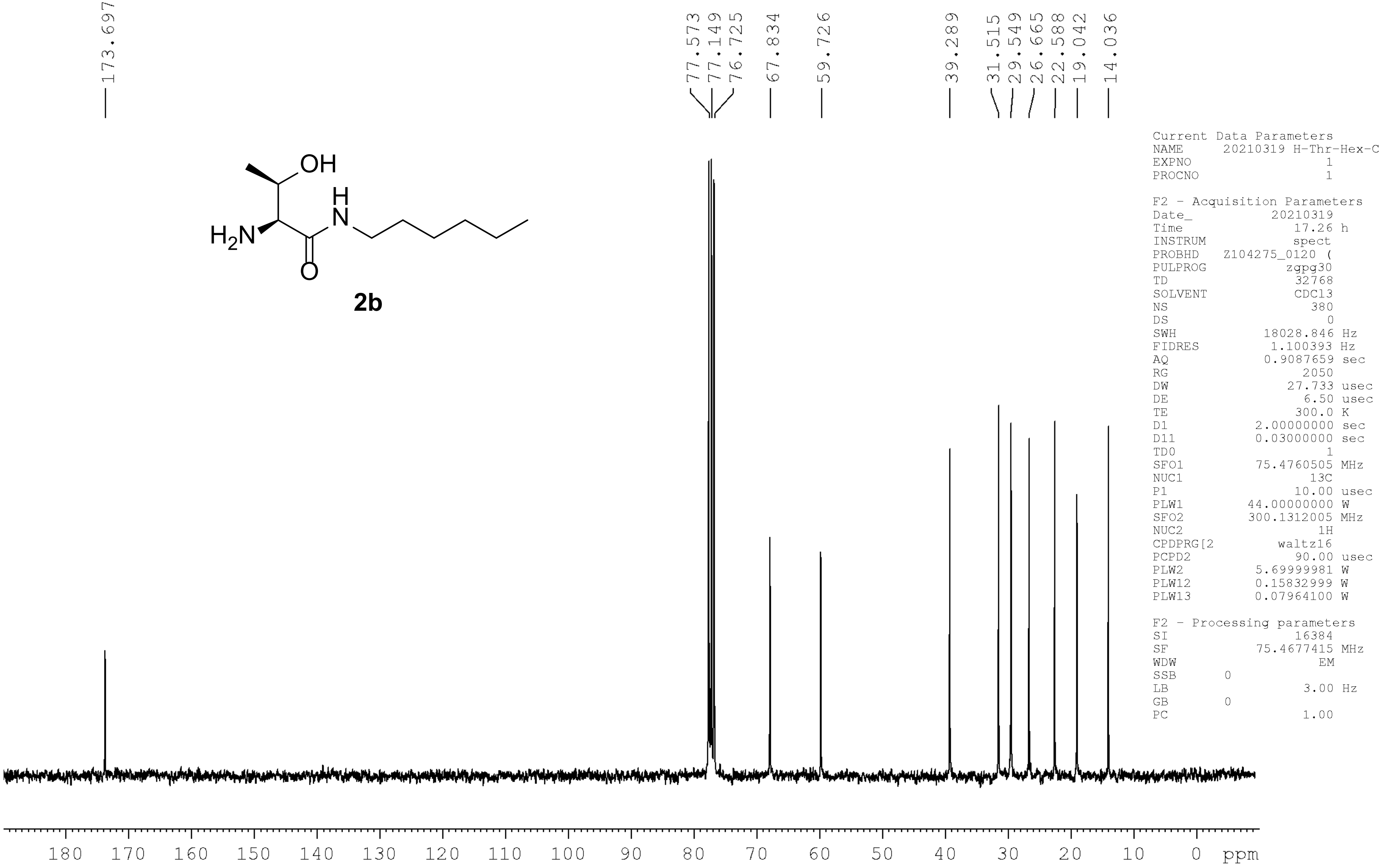


**2b**

$^{1}$H NMR Spectrum of **2c** (300 MHz, $CDCl_3$)

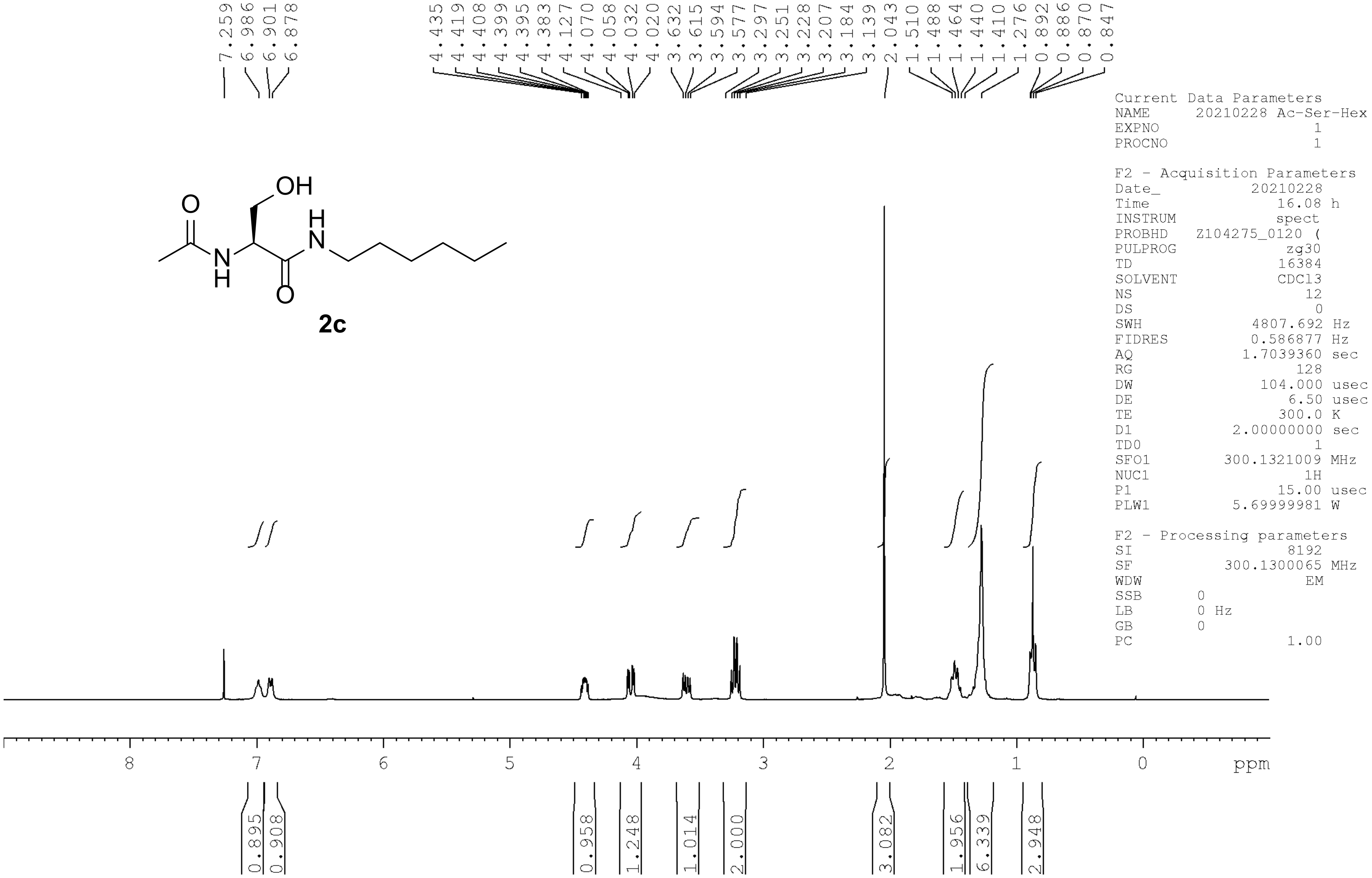

$^{13}C\{^{1}H\}$ NMR Spectrum of **2c** (100 MHz, $CDCl_3$)

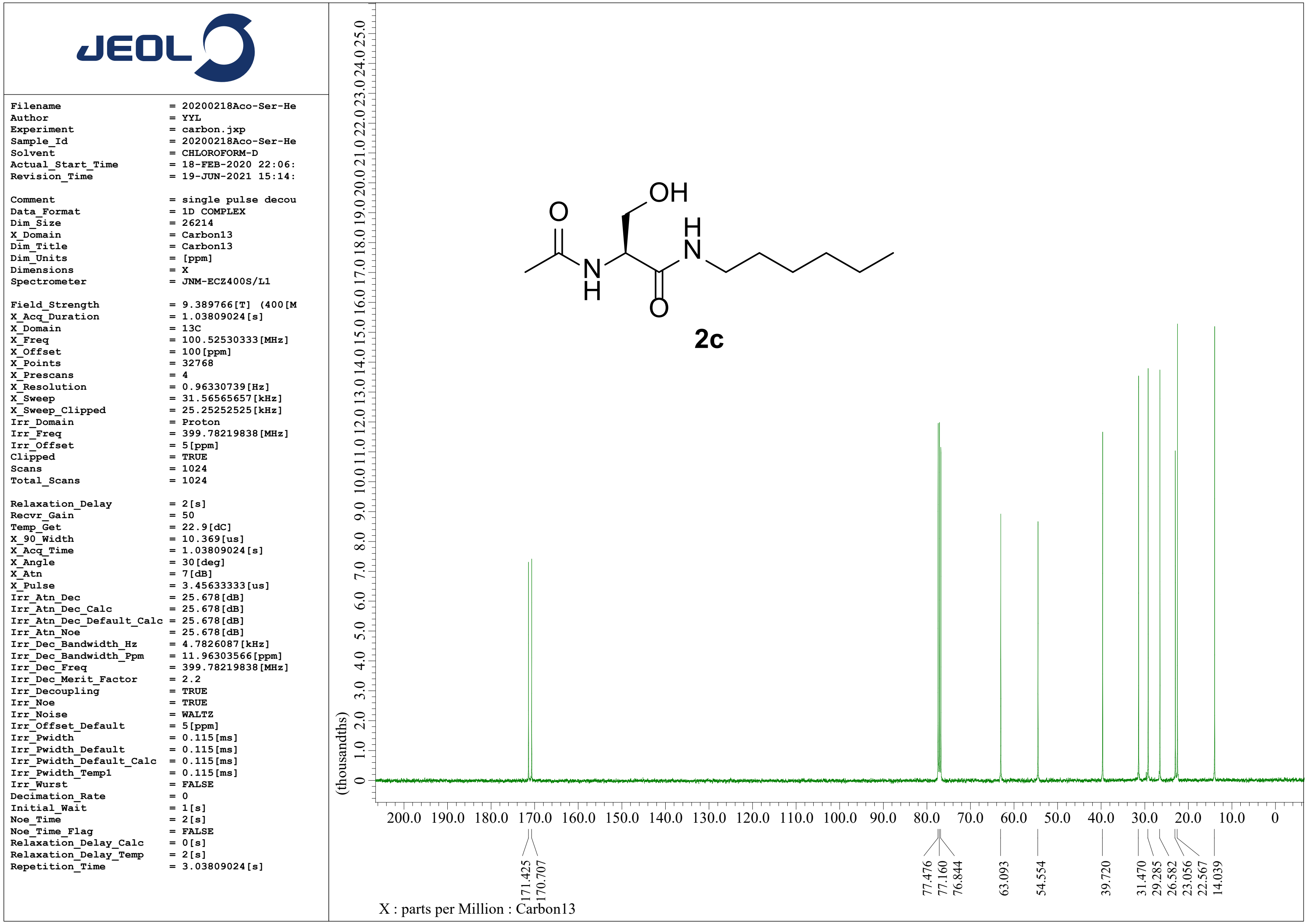

$^{1}$H NMR Spectrum of **2d** (300 MHz, $CDCl_3$)

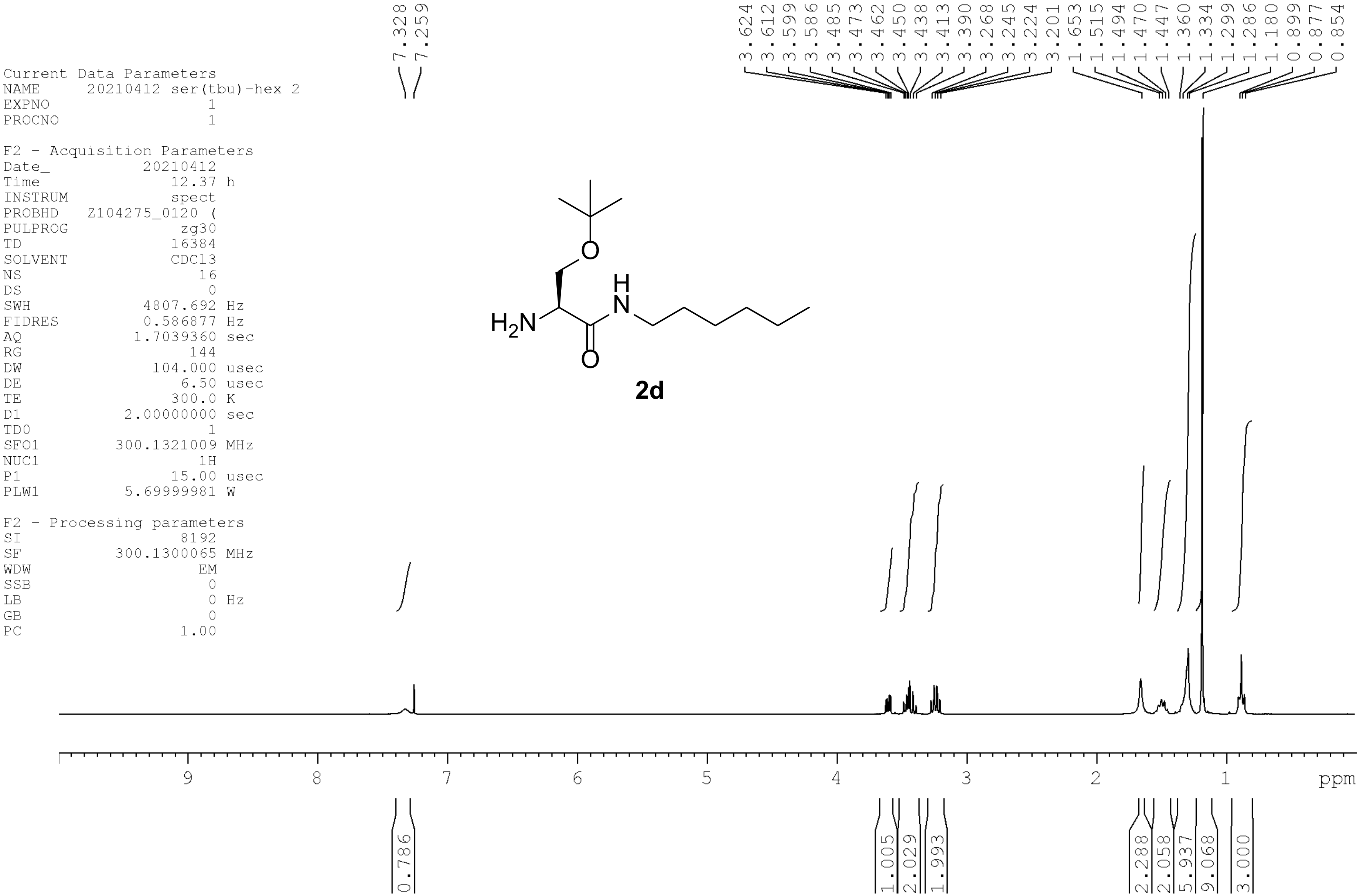

## $^{1}$H NMR Spectrum of **2e** (400 MHz, $CD_3OD$)

| | |
|---|---|
| Filename | = 20210818 H-Ser-Ala-He |
| Author | = YYL |
| Experiment | = proton.jxp |
| Sample_Id | = 20210818 H-Ser-Ala-He |
| Solvent | = METHANOL-D4 |
| Actual_Start_Time | = 18-AUG-2021 10:17:20 |
| Revision_Time | = 22-AUG-2021 15:22:23 |
| Comment | = single_pulse |
| Data_Format | = 1D COMPLEX |
| Dim_Size | = 13107 |
| X_Domain | = Proton |
| Dim_Title | = Proton |
| Dim_Units | = [ppm] |
| Dimensions | = X |
| Spectrometer | = JNM-ECZ400S/L1 |
| Field_Strength | = 9.389766[T] (400[MHz] |
| X_Acq_Duration | = 2.18628096[s] |
| X_Domain | = 1H |
| X_Freq | = 399.78219838[MHz] |
| X_Offset | = 5[ppm] |
| X_Points | = 16384 |
| X_Prescans | = 1 |
| X_Resolution | = 0.45739775[Hz] |
| X_Sweep | = 7.4940048[kHz] |
| X_Sweep_Clipped | = 5.99520384[kHz] |
| Irr_Domain | = Proton |
| Irr_Freq | = 399.78219838[MHz] |
| Irr_Offset | = 5[ppm] |
| Tri_Domain | = Proton |
| Tri_Freq | = 399.78219838[MHz] |
| Tri_Offset | = 5[ppm] |
| Clipped | = FALSE |
| Scans | = 8 |
| Total_Scans | = 8 |
| Relaxation_Delay | = 5[s] |
| Recvr_Gain | = 46 |
| Temp_Get | = 22.9[dC] |
| X_90_Width | = 7.7[us] |
| X_Acq_Time | = 2.18628096[s] |
| X_Angle | = 45[deg] |
| X_Atn | = 2[dB] |
| X_Pulse | = 3.85[us] |
| Irr_Mode | = Off |
| Tri_Mode | = Off |
| Dante_Loop | = 500 |
| Dante_Presat | = FALSE |
| Decimation_Rate | = 0 |
| Initial_Wait | = 1[s] |
| Phase | = {0, 90, 270, 180, 180 |
| Presat_Time | = 5[s] |
| Presat_Time_Flag | = FALSE |
| Relaxation_Delay_Calc | = 0[s] |
| Relaxation_Delay_Temp | = 5[s] |
| Repetition_Time | = 7.18628096[s] |

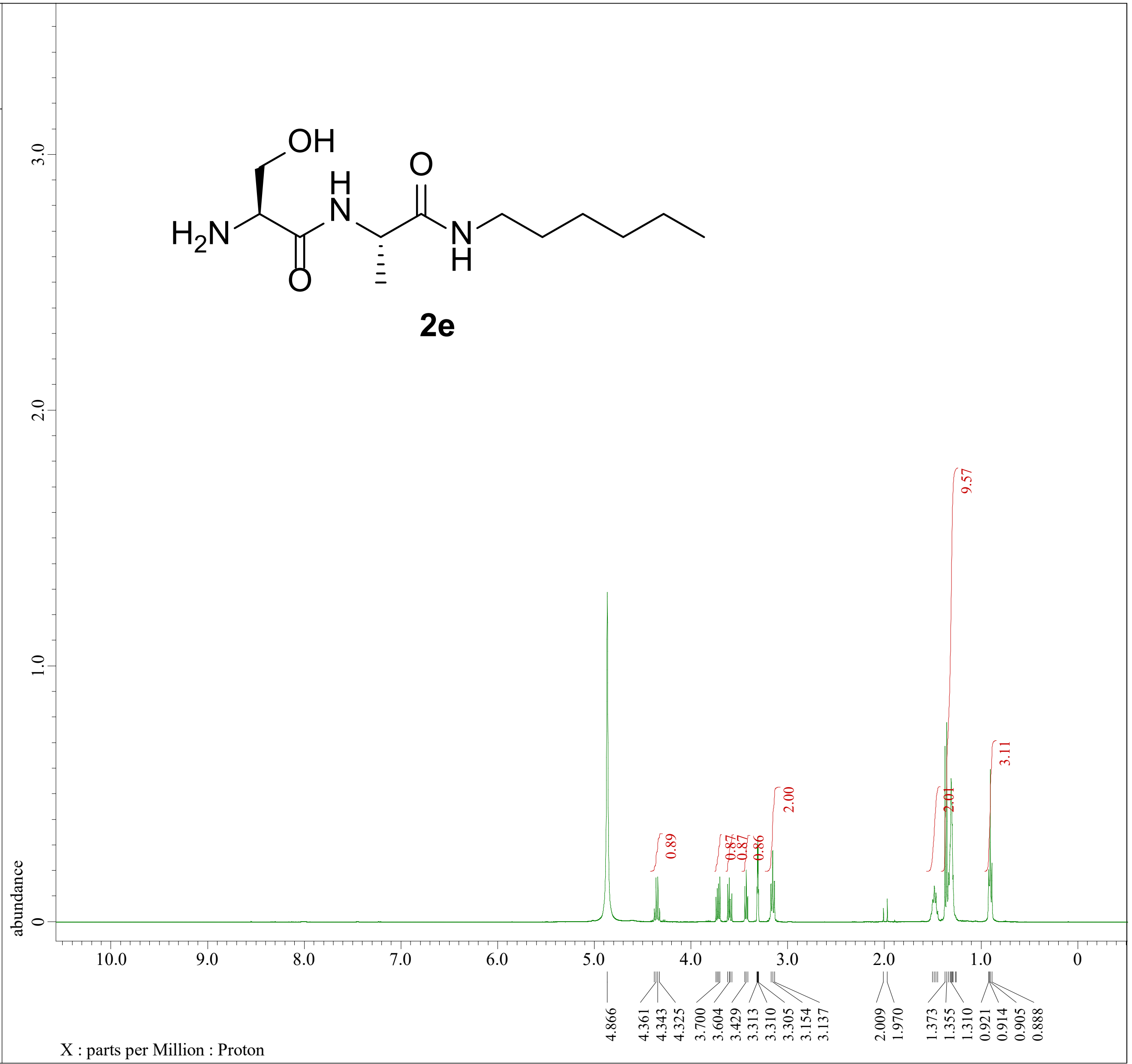

$^{13}C\{^{1}H\}$ NMR Spectrum of **2e** (100 MHz, $CD_3OD$)

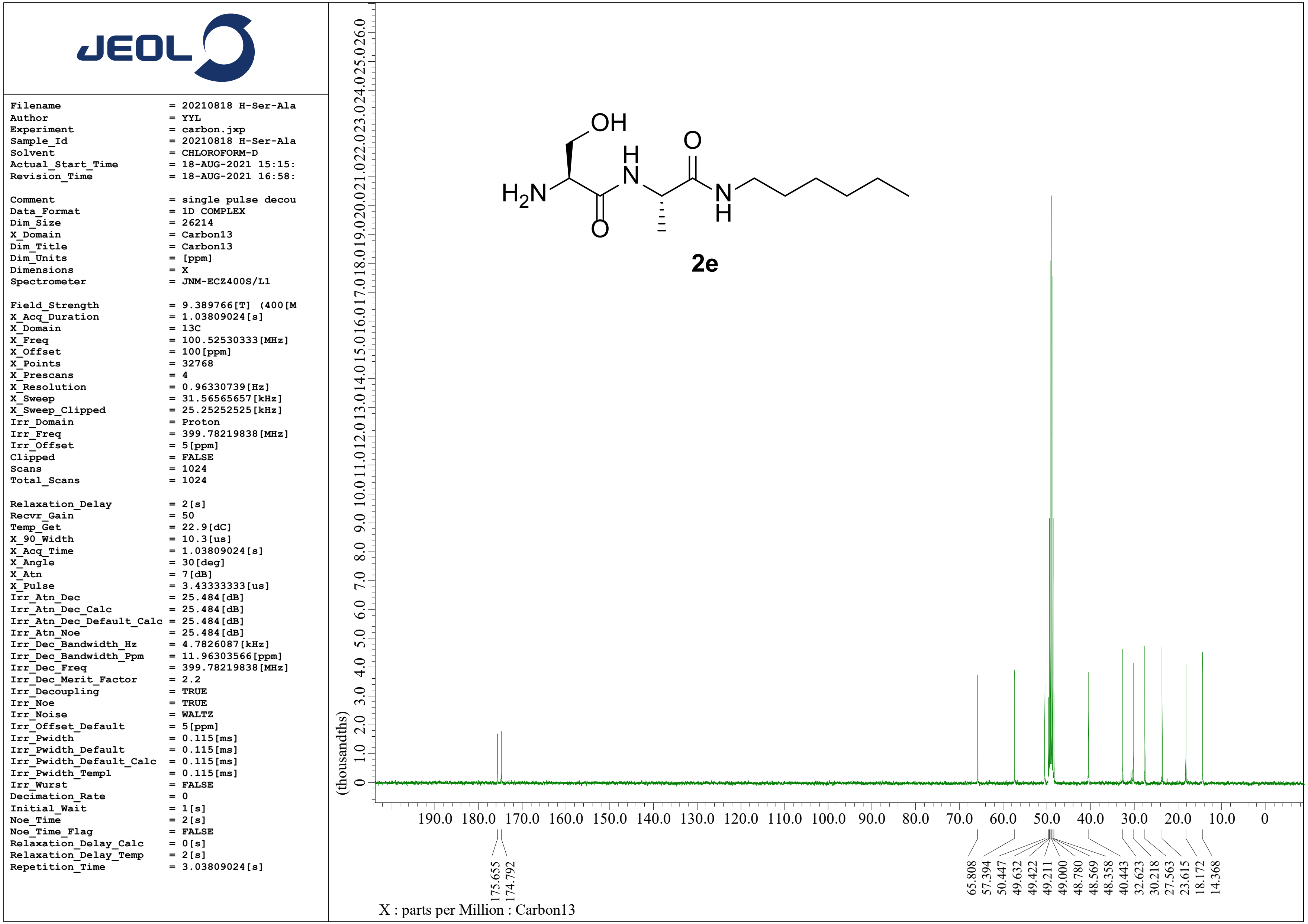

$^{1}$H NMR Spectrum of **2f** (300 MHz, $D_2O$)

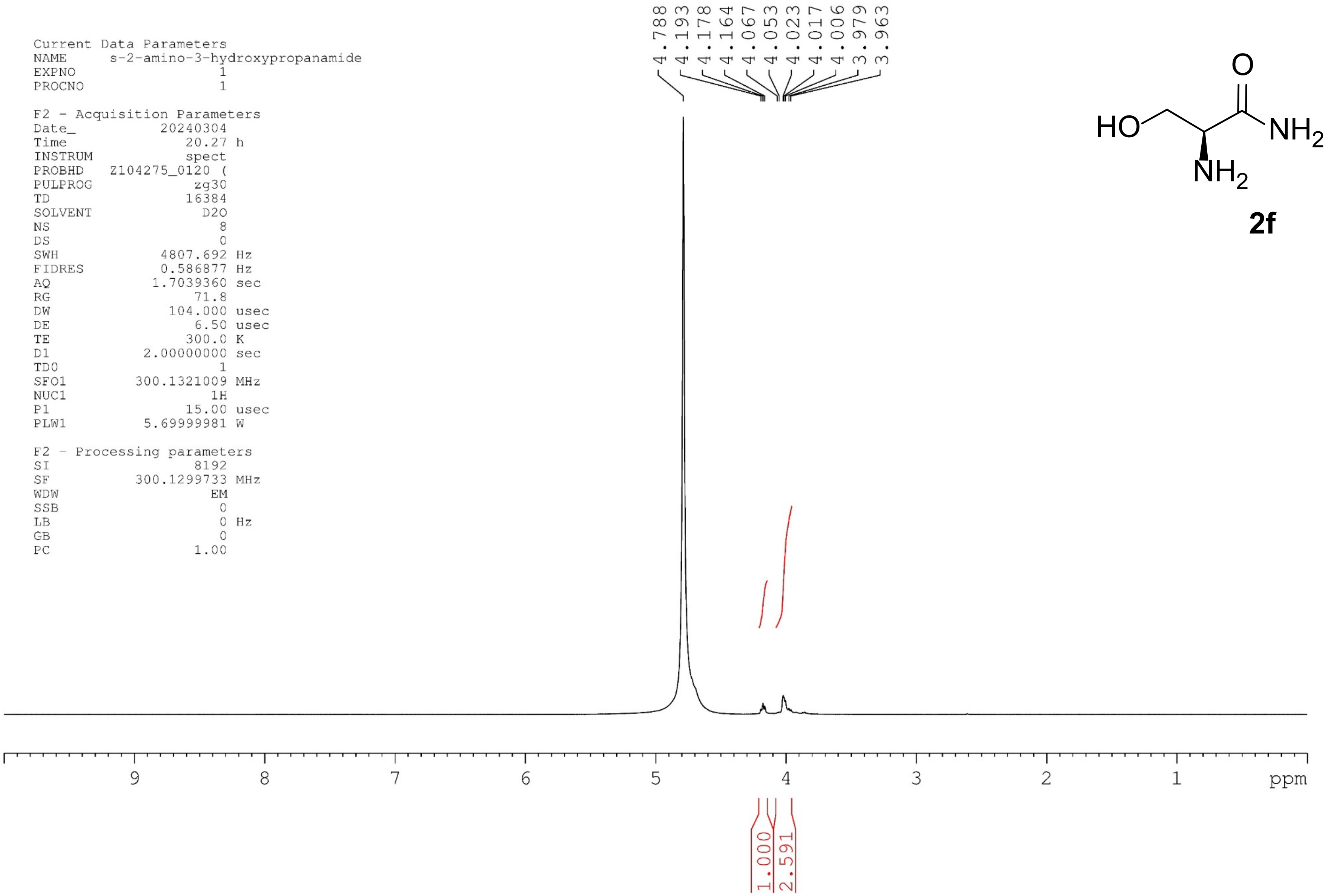


**2f**

$^{1}$H NMR Spectrum of **2g** (300 MHz, $CDCl_3$)

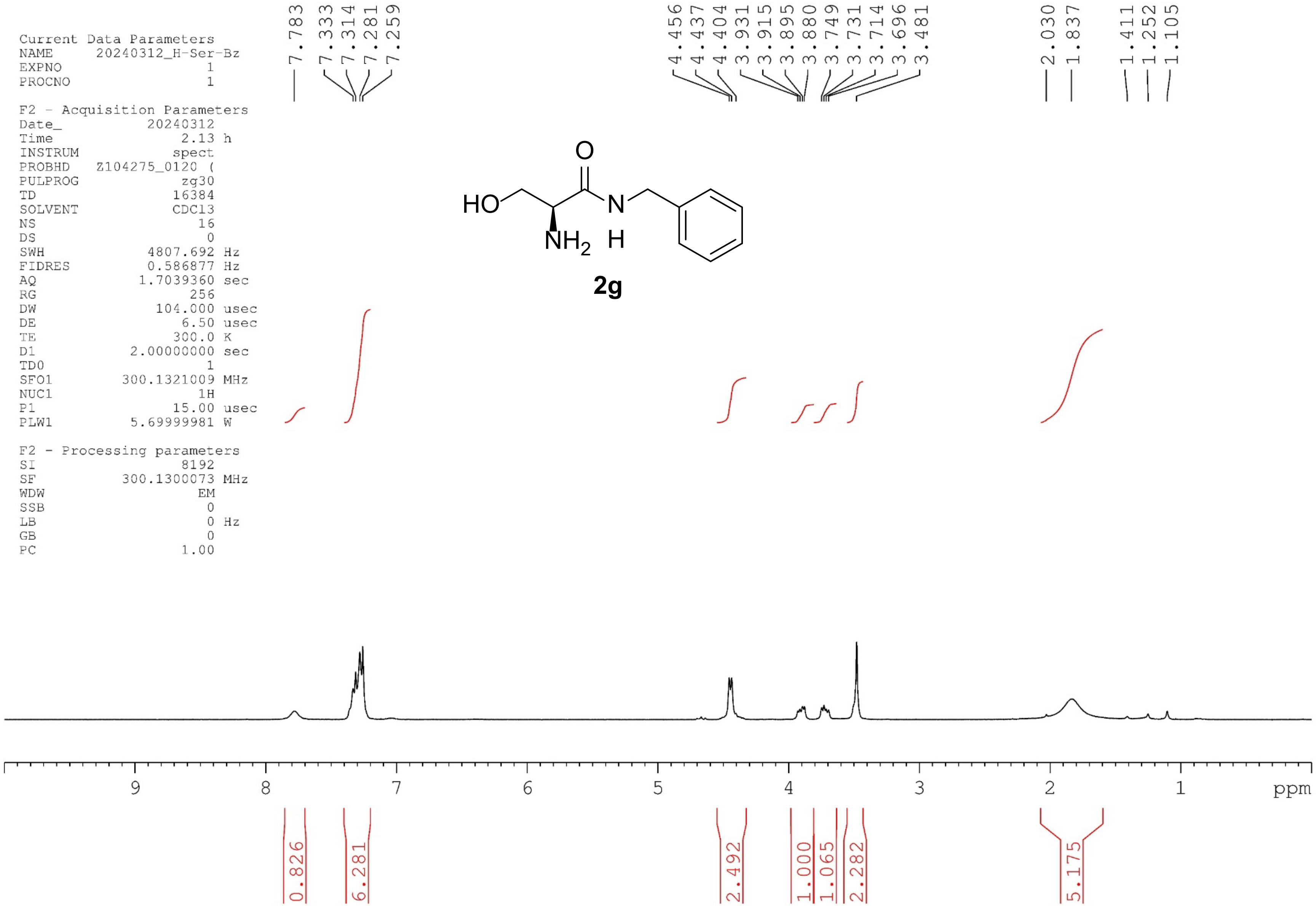

$^{1}$H NMR Spectrum of **3a** (300 MHz, $CDCl_3$)

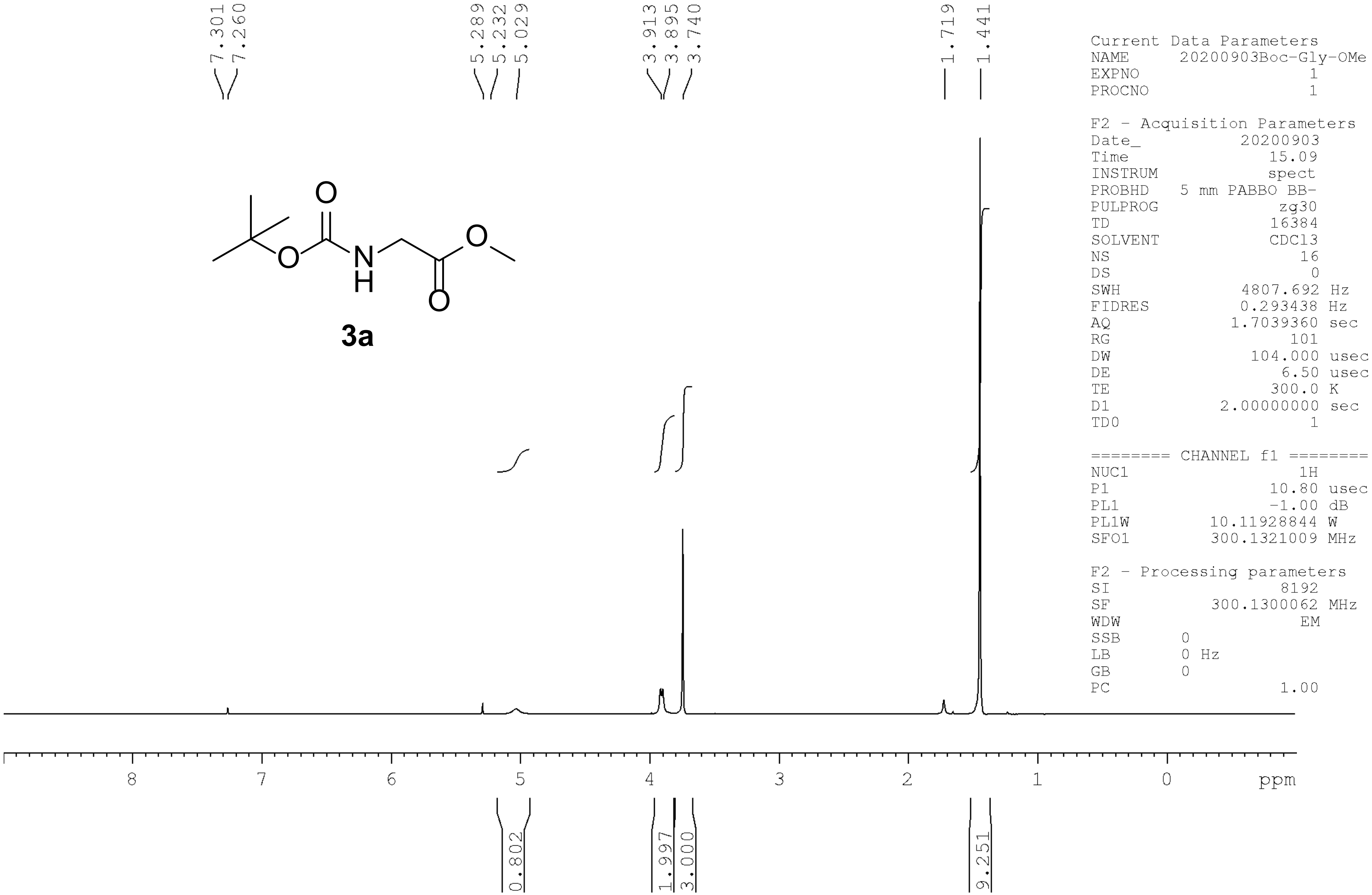

**3a**



```
Current Data Parameters
NAME      20200903Boc-Gly-OMe
EXPNO                       1
PROCNO                      1

F2 - Acquisition Parameters
Date_              20200903
Time                  15.09
INSTRUM               spect
PROBHD    5 mm PABBO BB-
PULPROG                zg30
TD                    16384
SOLVENT               CDCl3
NS                       16
DS                        0
SWH                4807.692 Hz
FIDRES             0.293438 Hz
AQ                1.7039360 sec
RG                      101
DW                  104.000 usec
DE                     6.50 usec
TE                    300.0 K
D1               2.00000000 sec
TD0                       1

======== CHANNEL f1 ========
NUC1                     1H
P1                    10.80 usec
PL1                   -1.00 dB
PL1W            10.11928844 W
SFO1            300.1321009 MHz

F2 - Processing parameters
SI                     8192
SF              300.1300062 MHz
WDW                      EM
SSB       0
LB        0 Hz
GB        0
PC                     1.00
```

$^{1}$H NMR Spectrum of **3b** (300 MHz, $CDCl_3$)

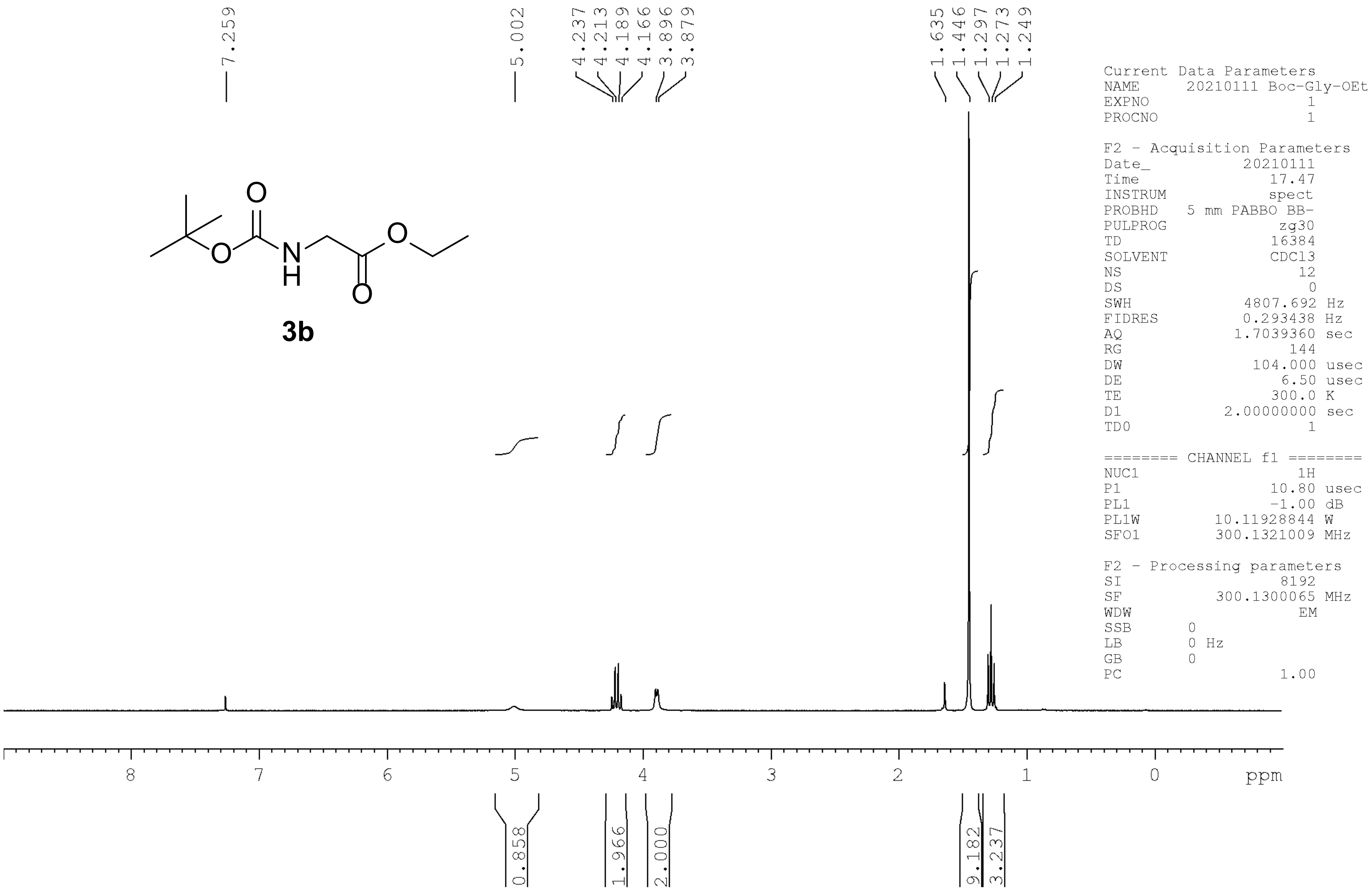

$^{1}$H NMR Spectrum of **3c** (300 MHz, $CDCl_3$)

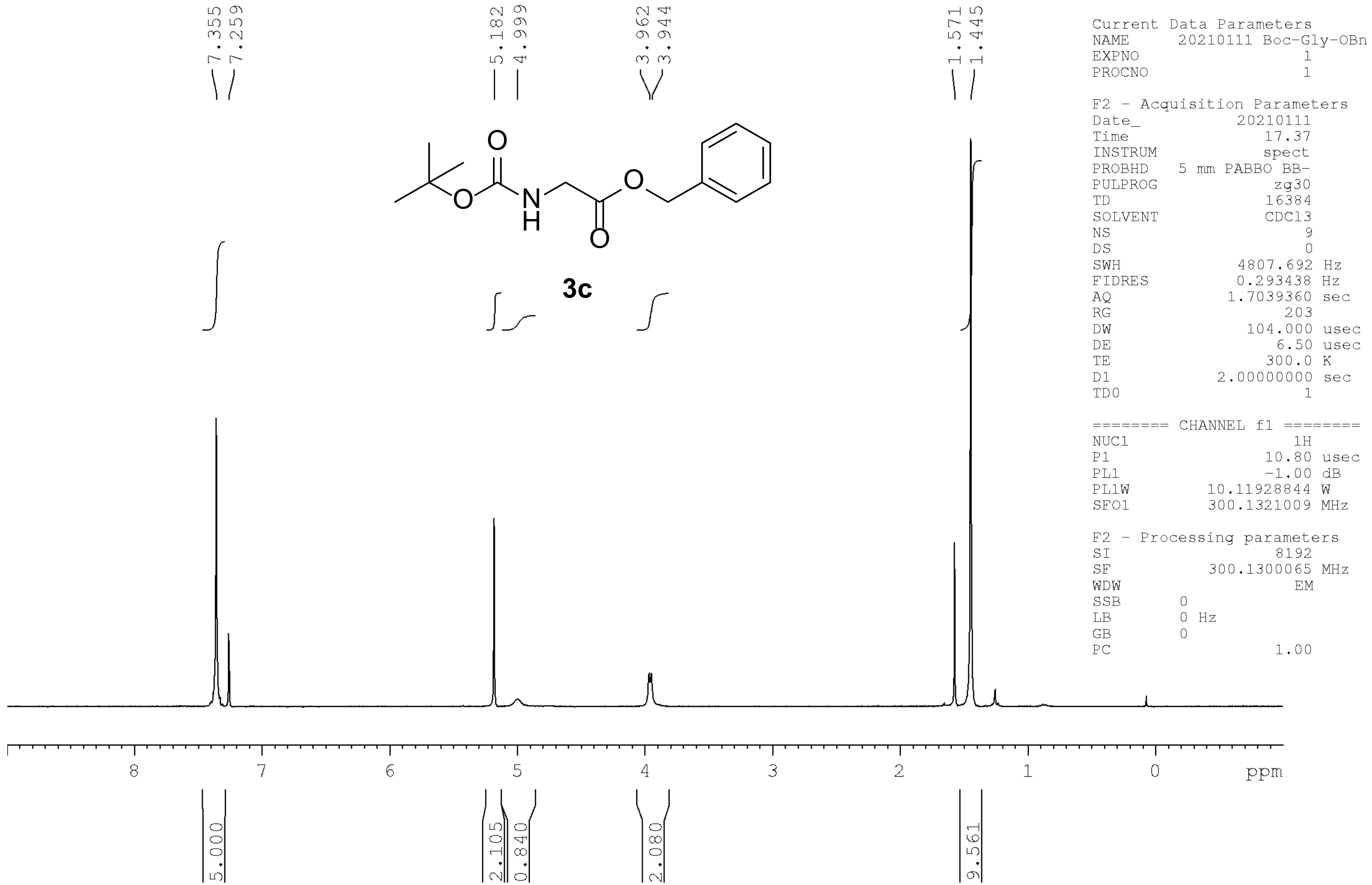

$^{1}$H NMR Spectrum of **3d** (300 MHz, $CDCl_3$)

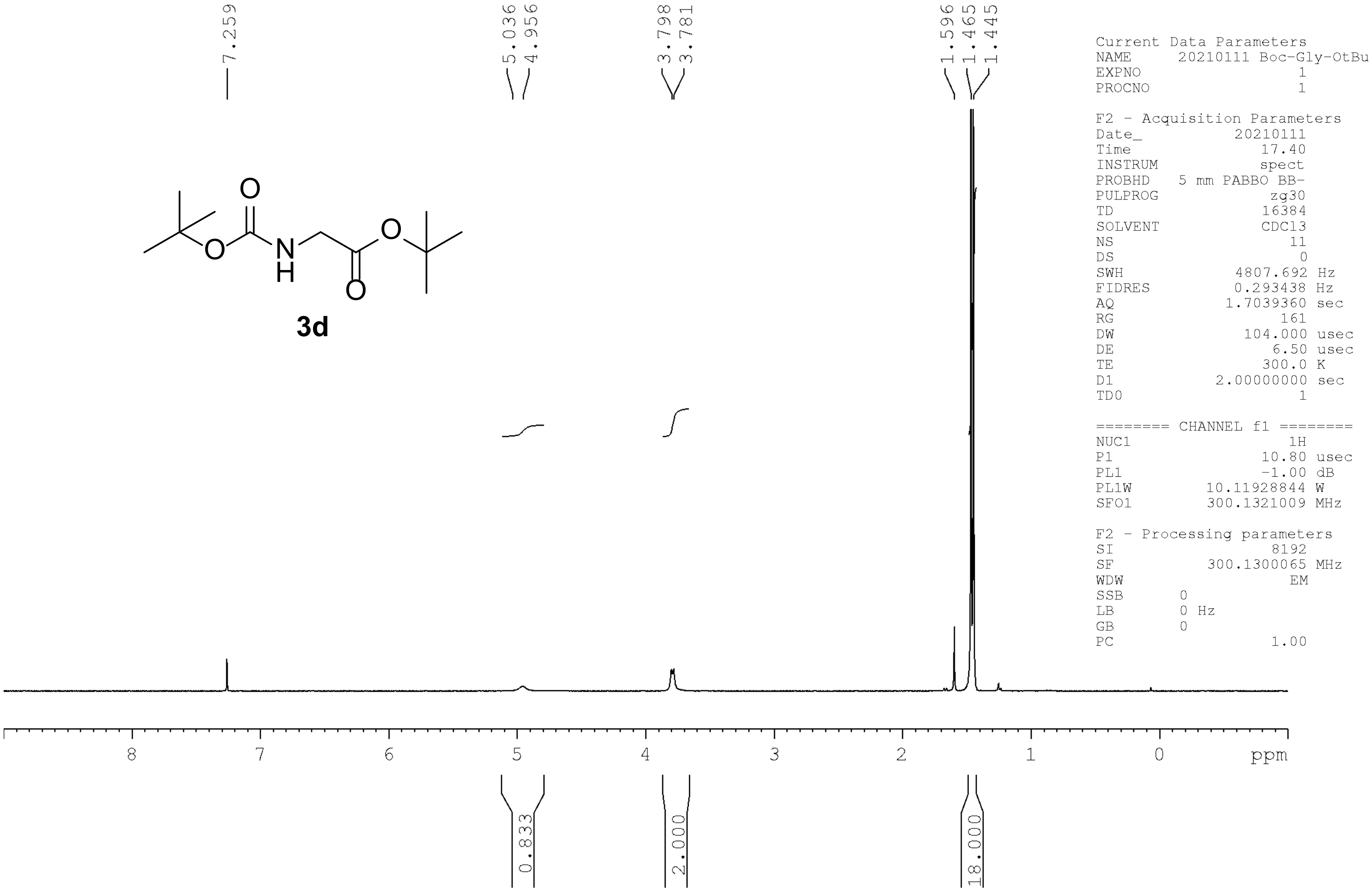


| | |
|---|---|
| Current Data Parameters | |
| NAME | 20210111 Boc-Gly-OtBu |
| EXPNO | 1 |
| PROCNO | 1 |
| F2 - Acquisition Parameters | |
| Date_ | 20210111 |
| Time | 17.40 |
| INSTRUM | spect |
| PROBHD | 5 mm PABBO BB- |
| PULPROG | zg30 |
| TD | 16384 |
| SOLVENT | CDCl3 |
| NS | 11 |
| DS | 0 |
| SWH | 4807.692 Hz |
| FIDRES | 0.293438 Hz |
| AQ | 1.7039360 sec |
| RG | 161 |
| DW | 104.000 usec |
| DE | 6.50 usec |
| TE | 300.0 K |
| D1 | 2.00000000 sec |
| TD0 | 1 |
| ======== CHANNEL f1 ======== | |
| NUC1 | 1H |
| P1 | 10.80 usec |
| PL1 | -1.00 dB |
| PL1W | 10.11928844 W |
| SFO1 | 300.1321009 MHz |
| F2 - Processing parameters | |
| SI | 8192 |
| SF | 300.1300065 MHz |
| WDW | EM |
| SSB | 0 |
| LB | 0 Hz |
| GB | 0 |
| PC | 1.00 |

$^{1}$H NMR Spectrum of **3e** (300 MHz, $CDCl_3$)

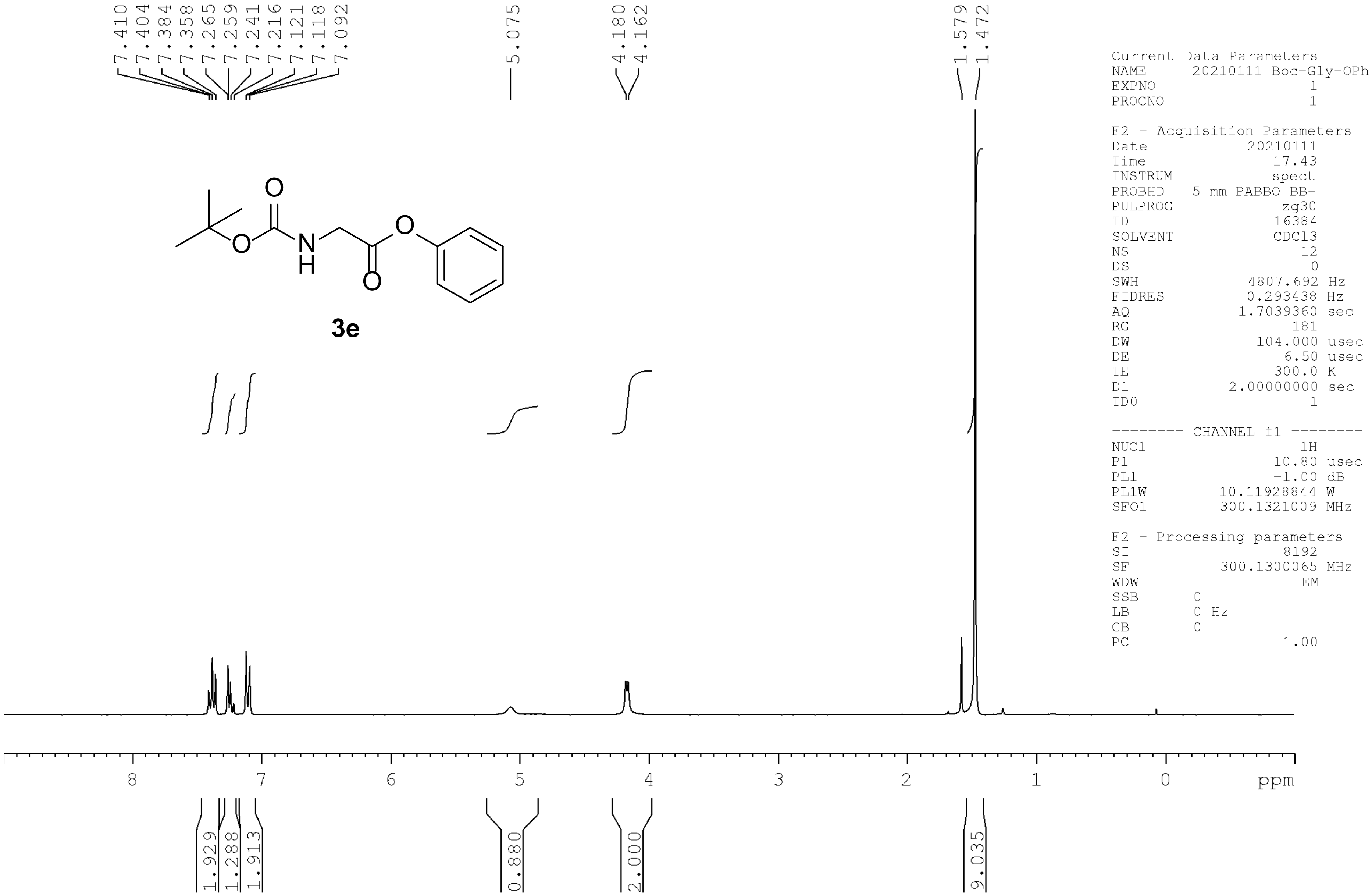

$^{13}C\{^{1}H\}$ NMR Spectrum of **3e** (75 MHz, $CDCl_3$)

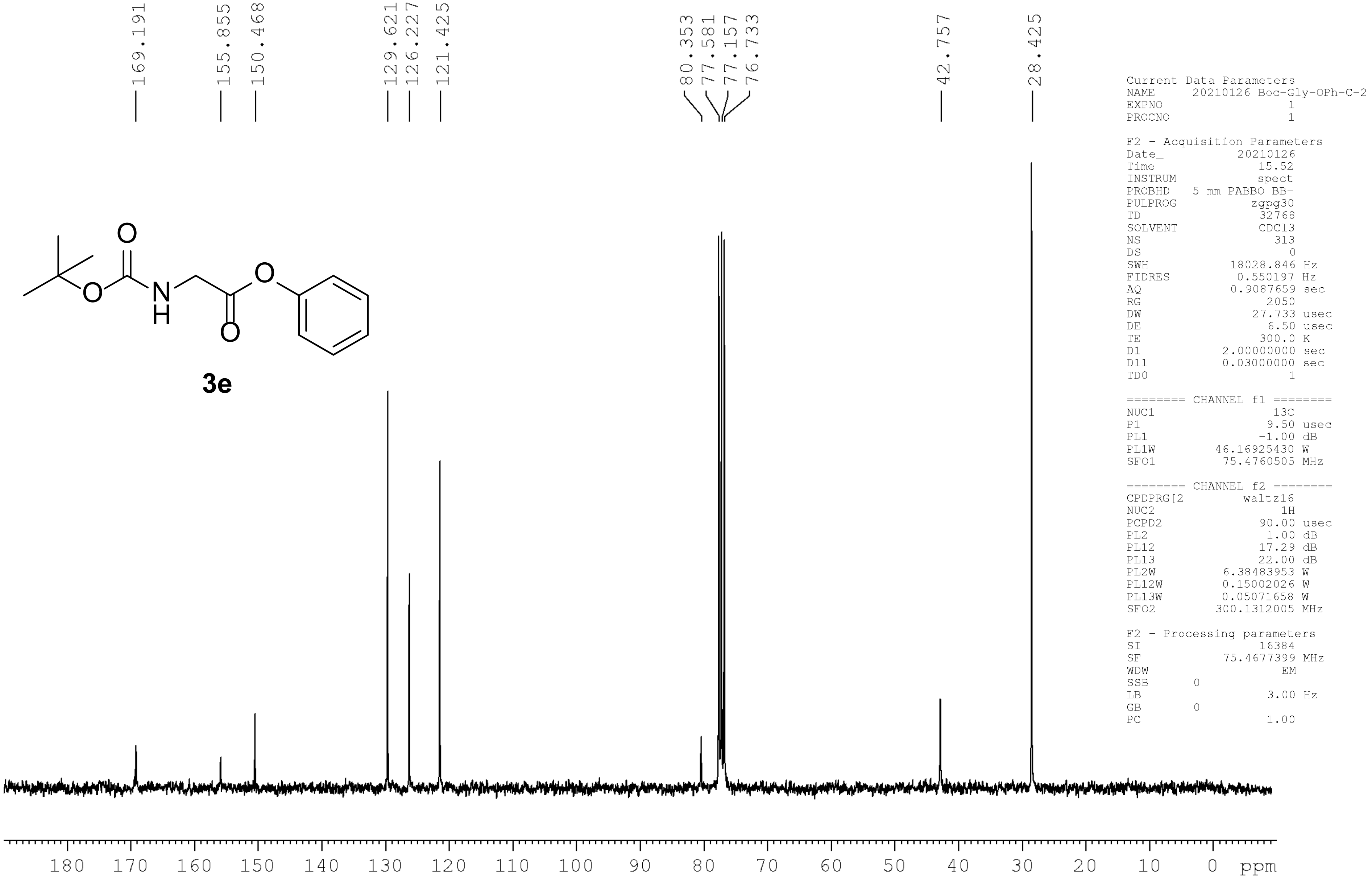

$^{1}$H NMR Spectrum of **4a** (300 MHz, $CDCl_3$)

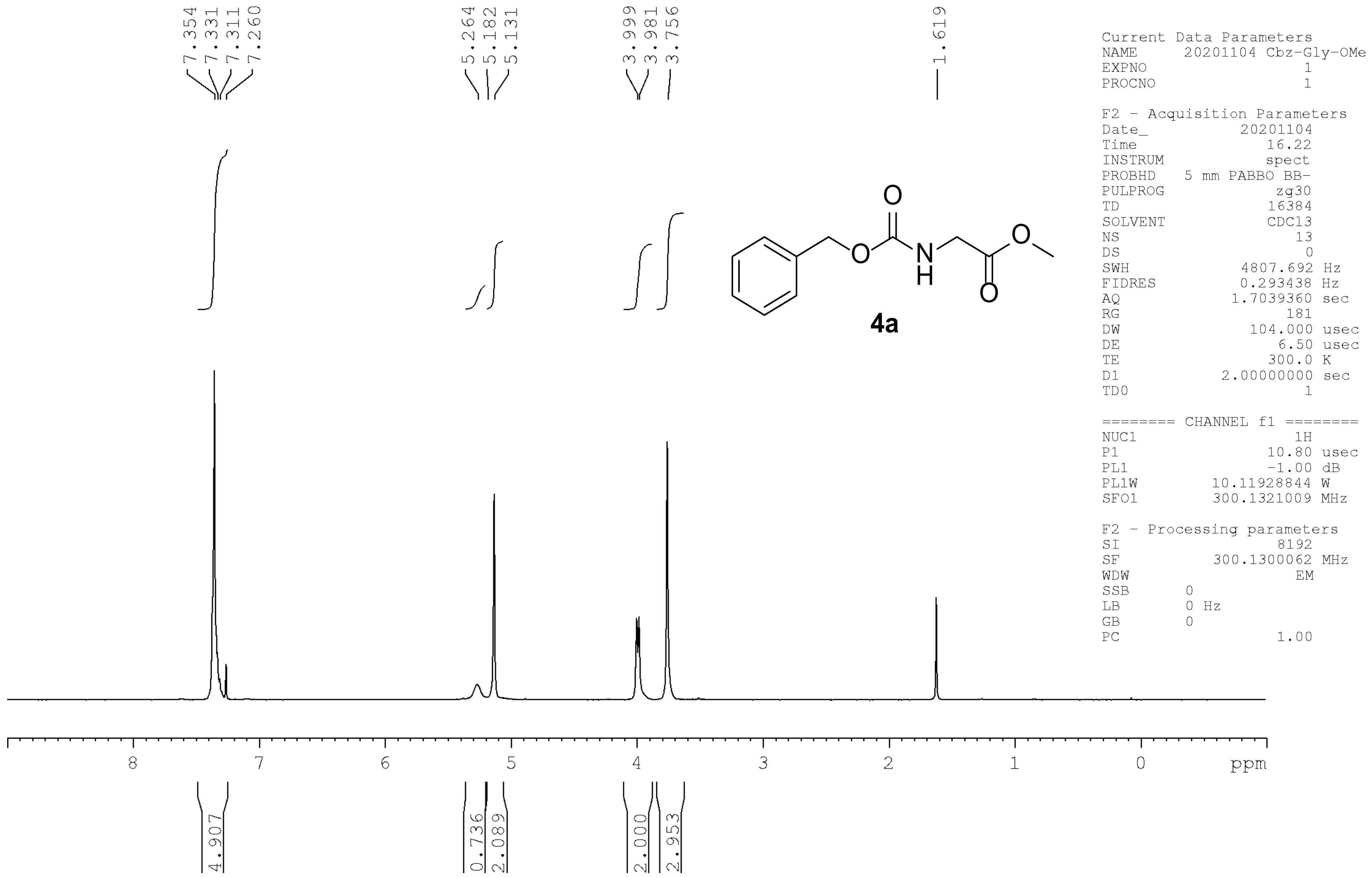

$^{1}$H NMR Spectrum of **4b** (400 MHz, $CDCl_3$)

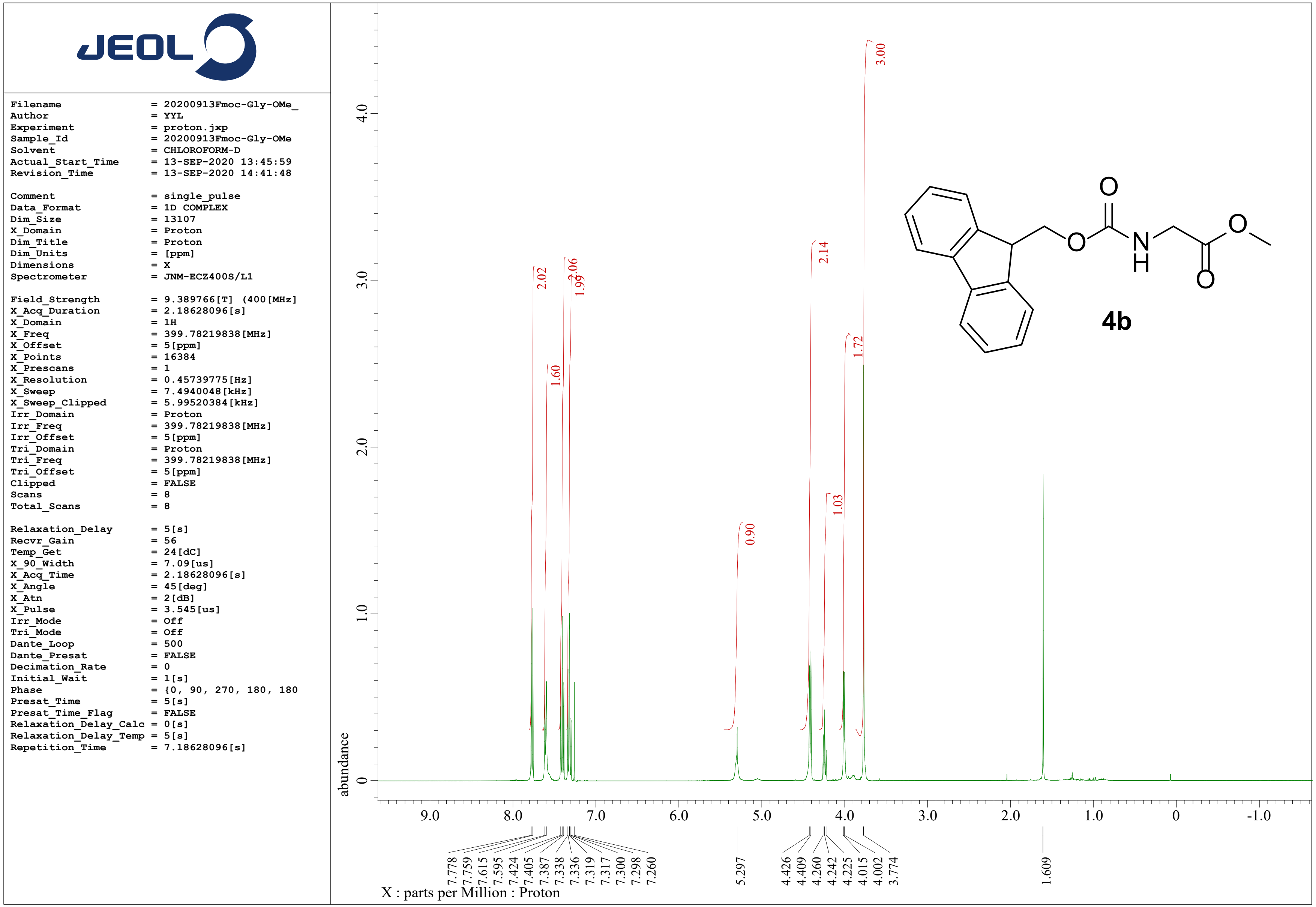

$^{1}$H NMR Spectrum of **4c** (300 MHz, $CDCl_3$)

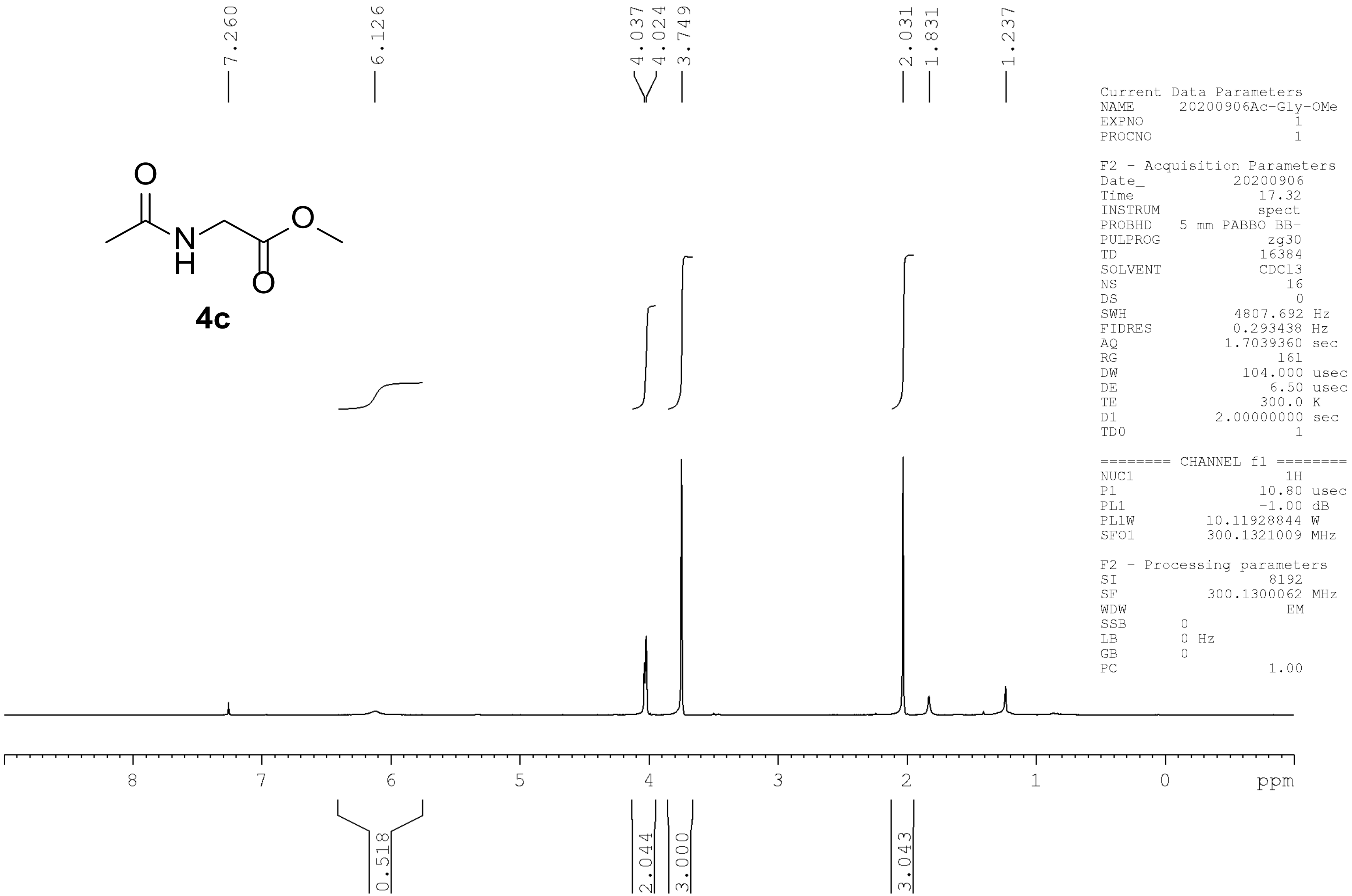

$^{1}$H NMR Spectrum of **4d** (300 MHz, $CDCl_3$)

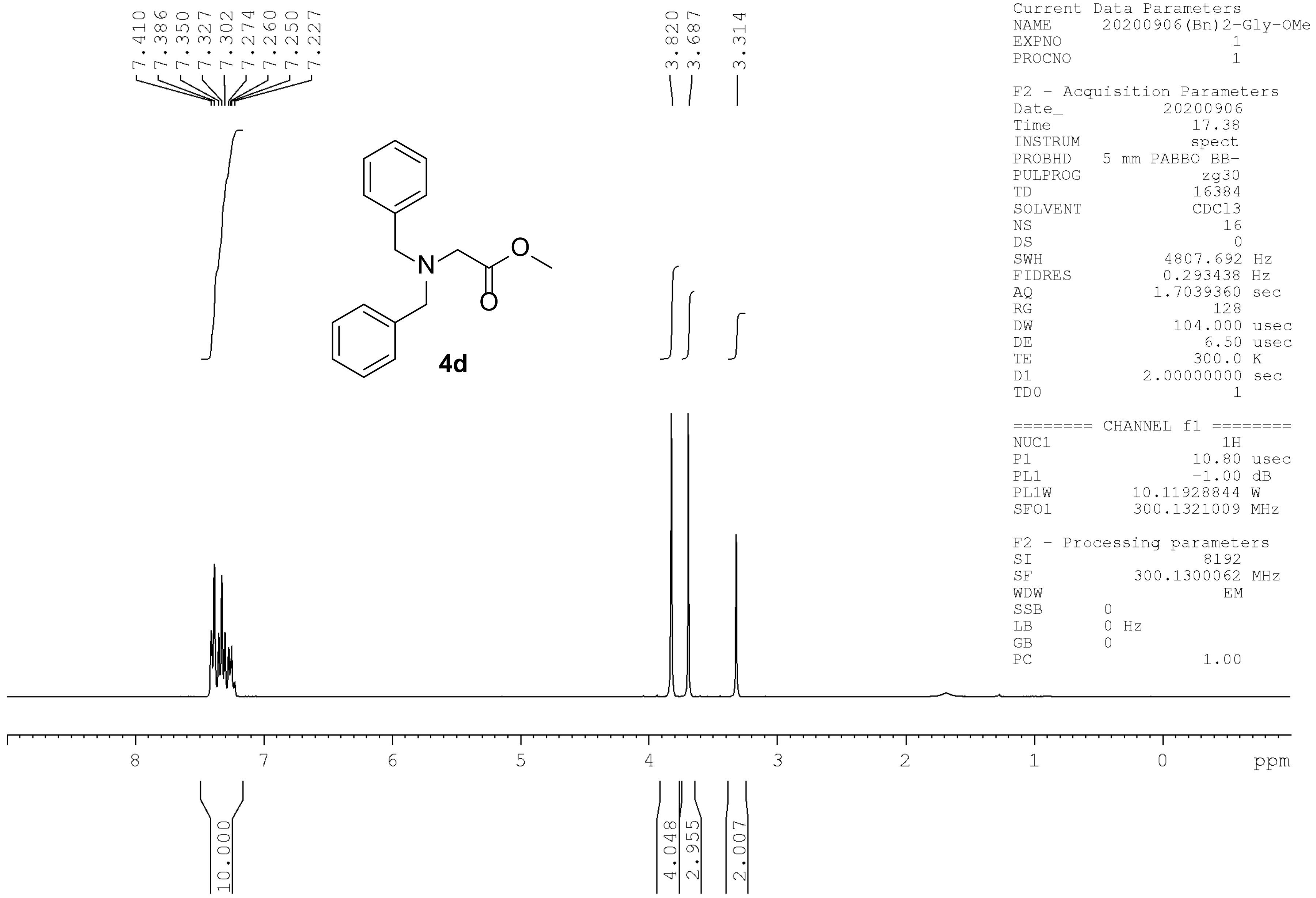

$^{13}C\{^{1}H\}$ NMR Spectrum of **4d** (75 MHz, $CDCl_3$)

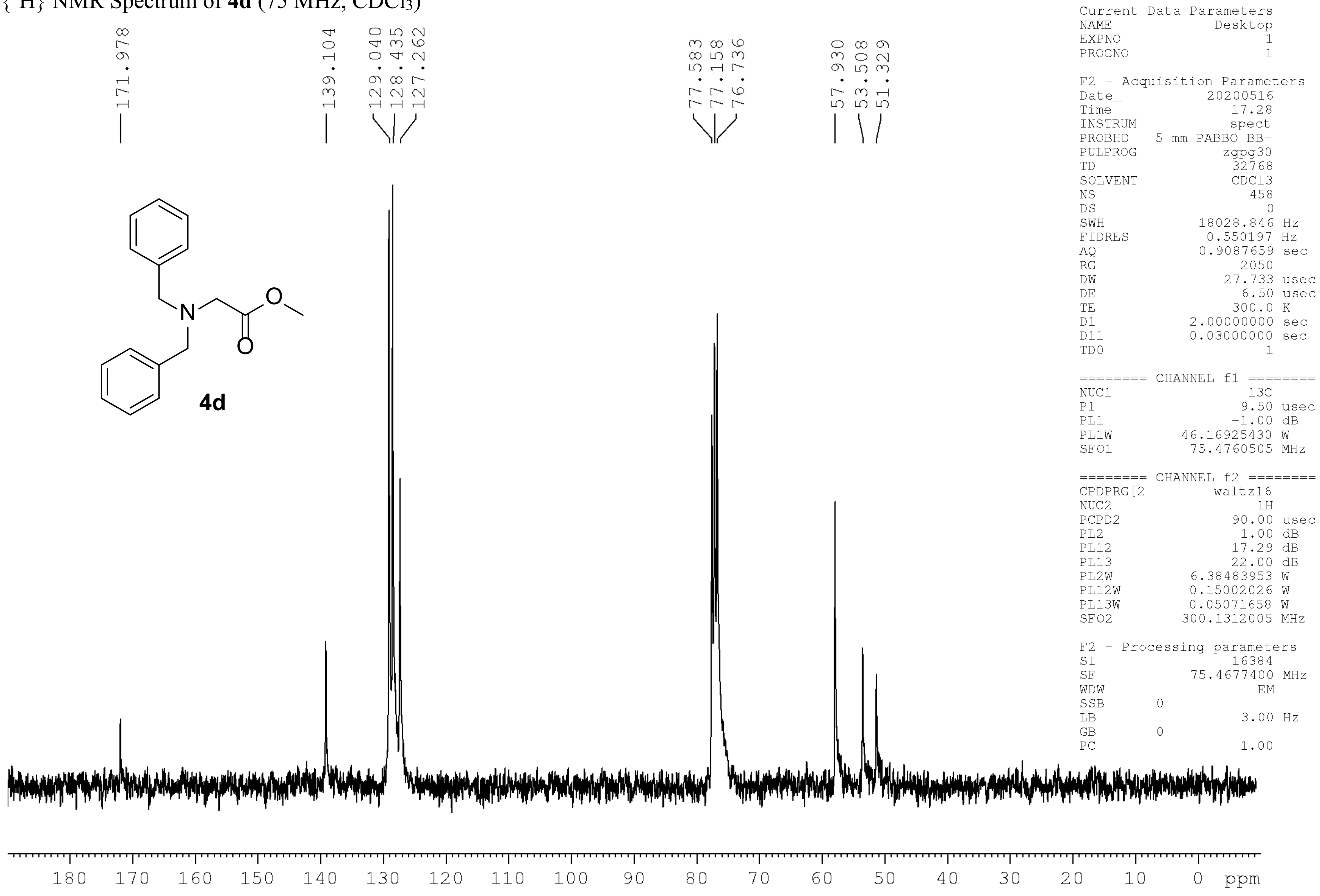

$^{1}$H NMR Spectrum of **5a** (300 MHz, $CDCl_3$)

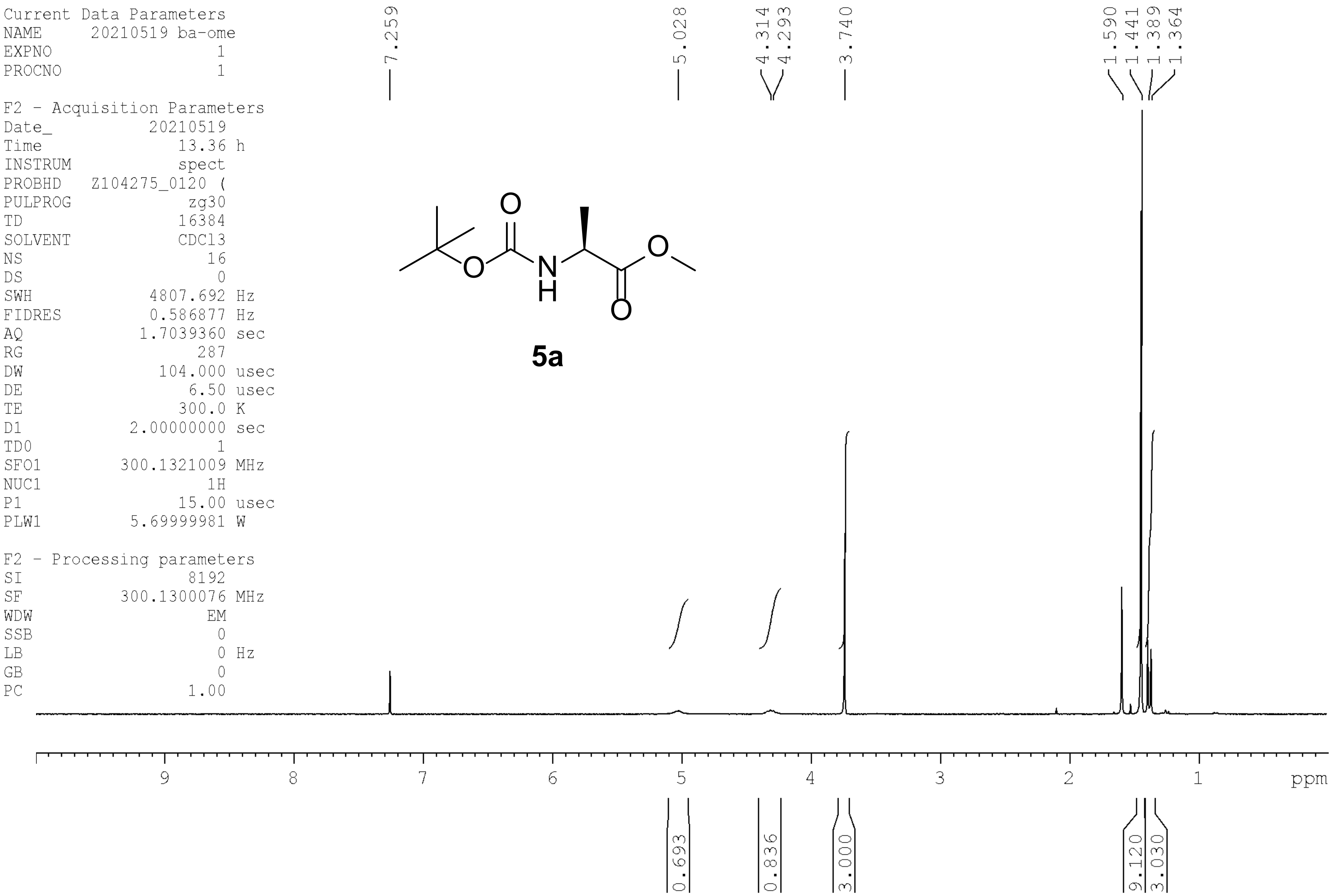

$^{1}$H NMR Spectrum of **5b** (300 MHz, $CDCl_3$)

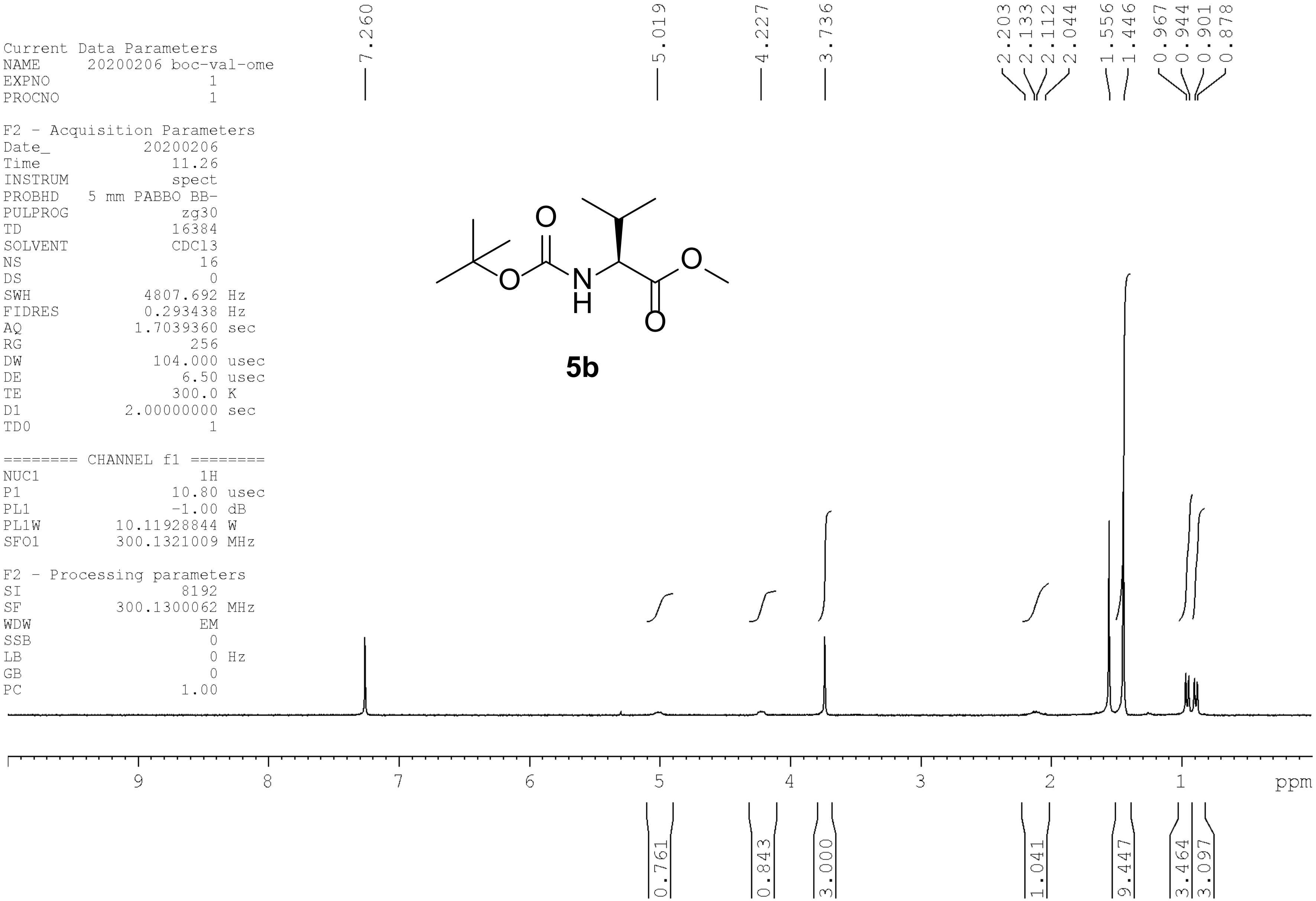

$^{1}$H NMR Spectrum of **5c** (300 MHz, $CDCl_3$)

```
Current Data Parameters
NAME      20210519 bl-ome
EXPNO                   1
PROCNO                  1

F2 - Acquisition Parameters
Date_          20210519
Time              13.32 h
INSTRUM           spect
PROBHD   Z104275_0120 (
PULPROG            zg30
TD                16384
SOLVENT           CDCl3
NS                   16
DS                    0
SWH            4807.692 Hz
FIDRES         0.586877 Hz
AQ            1.7039360 sec
RG                  256
DW              104.000 usec
DE                 6.50 usec
TE                300.0 K
D1           2.00000000 sec
TD0                   1
SFO1        300.1321009 MHz
NUC1                 1H
P1                15.00 usec
PLW1         5.69999981 W

F2 - Processing parameters
SI                 8192
SF          300.1300077 MHz
WDW                  EM
SSB                   0
LB                    0 Hz
GB                    0
PC                 1.00
```

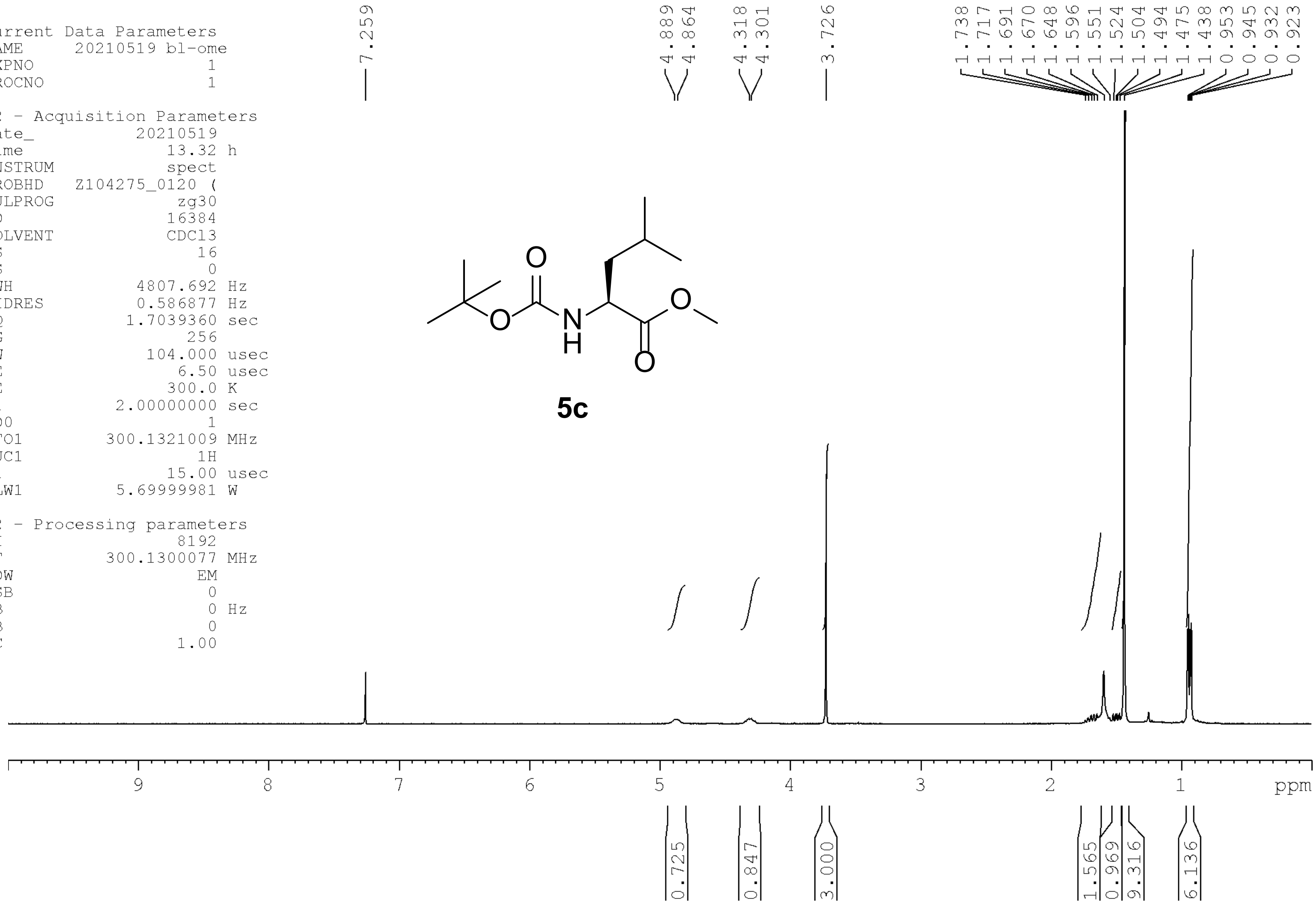


**5c**

$^{1}$H NMR Spectrum of **5d** (300 MHz, $CDCl_3$)

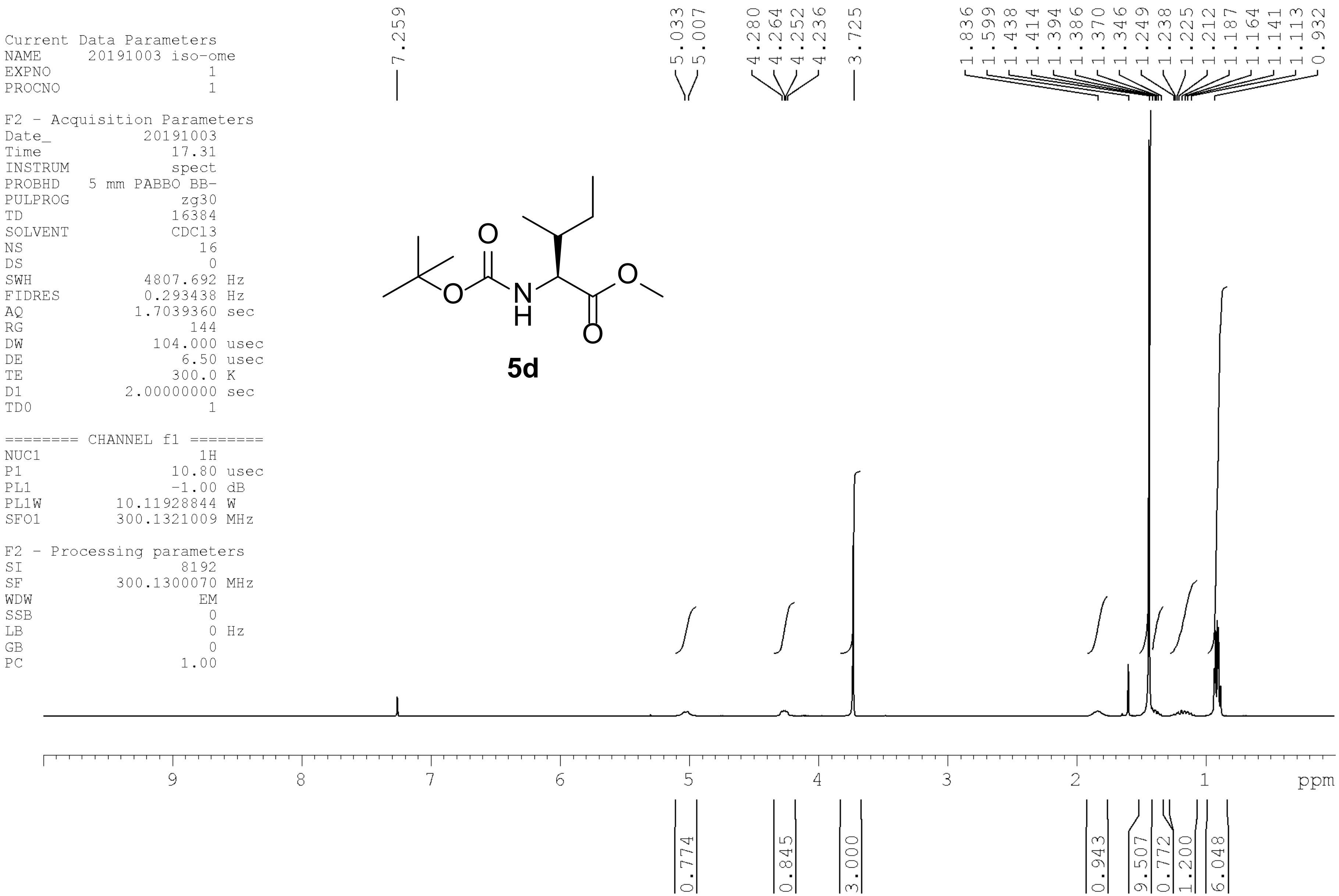


**5d**

$^{1}$H NMR Spectrum of **5e** (300 MHz, $CDCl_3$)

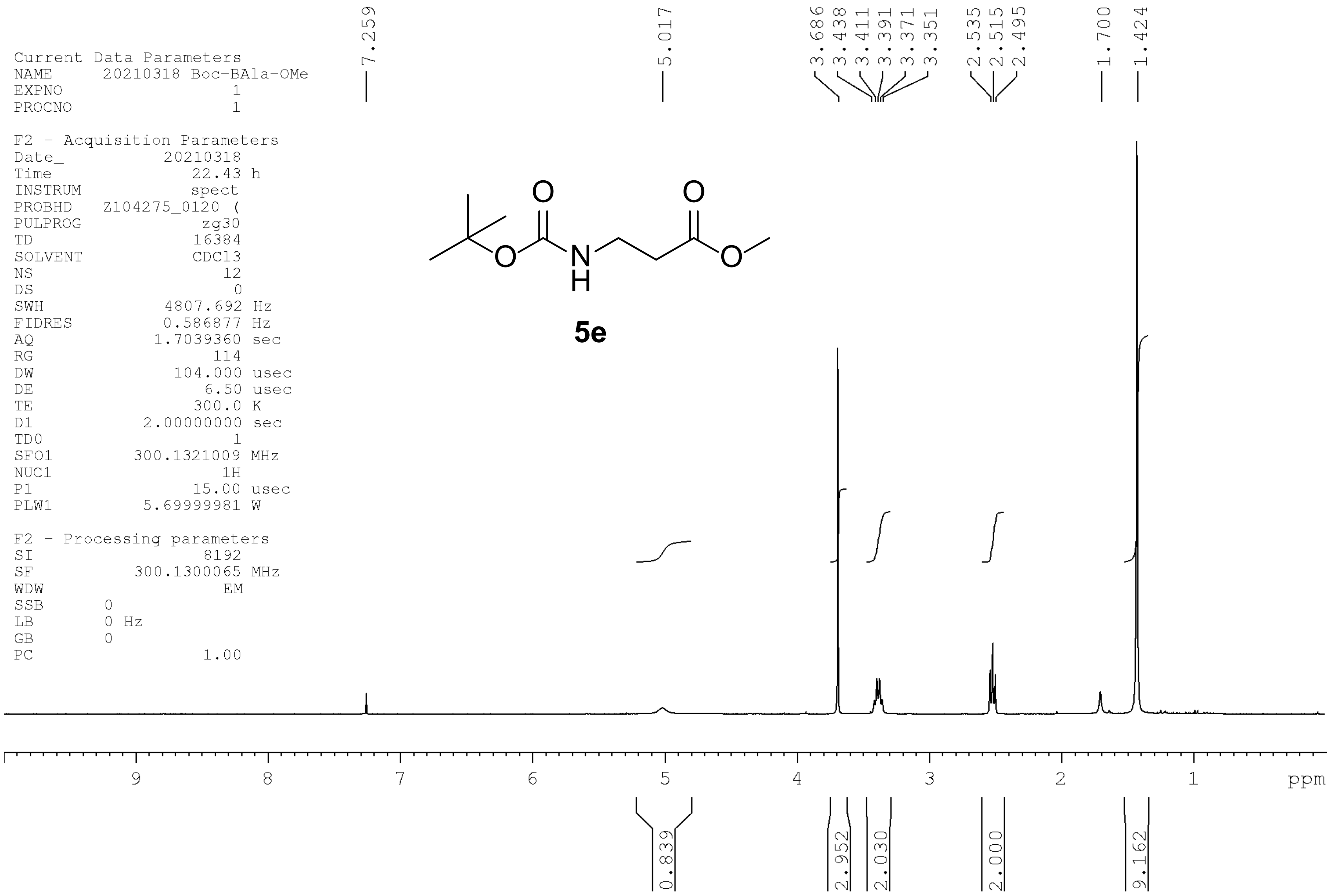

## $^{1}$H NMR Spectrum of **5f** (300 MHz, $CDCl_3$)

```
Current Data Parameters
NAME      20210409 Boc-HomoAla-OMe
EXPNO                    1
PROCNO                   1

F2 - Acquisition Parameters
Date_            20210409
Time                22.20 h
INSTRUM             spect
PROBHD   Z104275_0120 (
PULPROG              zg30
TD                  16384
SOLVENT             CDCl3
NS                     12
DS                      0
SWH              4807.692 Hz
FIDRES           0.586877 Hz
AQ              1.7039360 sec
RG                    144
DW                104.000 usec
DE                   6.50 usec
TE                  300.0 K
D1             2.00000000 sec
TD0                     1
SFO1          300.1321009 MHz
NUC1                   1H
P1                  15.00 usec
PLW1           5.69999981 W

F2 - Processing parameters
SI                   8192
SF            300.1300065 MHz
WDW                    EM
SSB       0
LB        0 Hz
GB        0
PC                   1.00
```

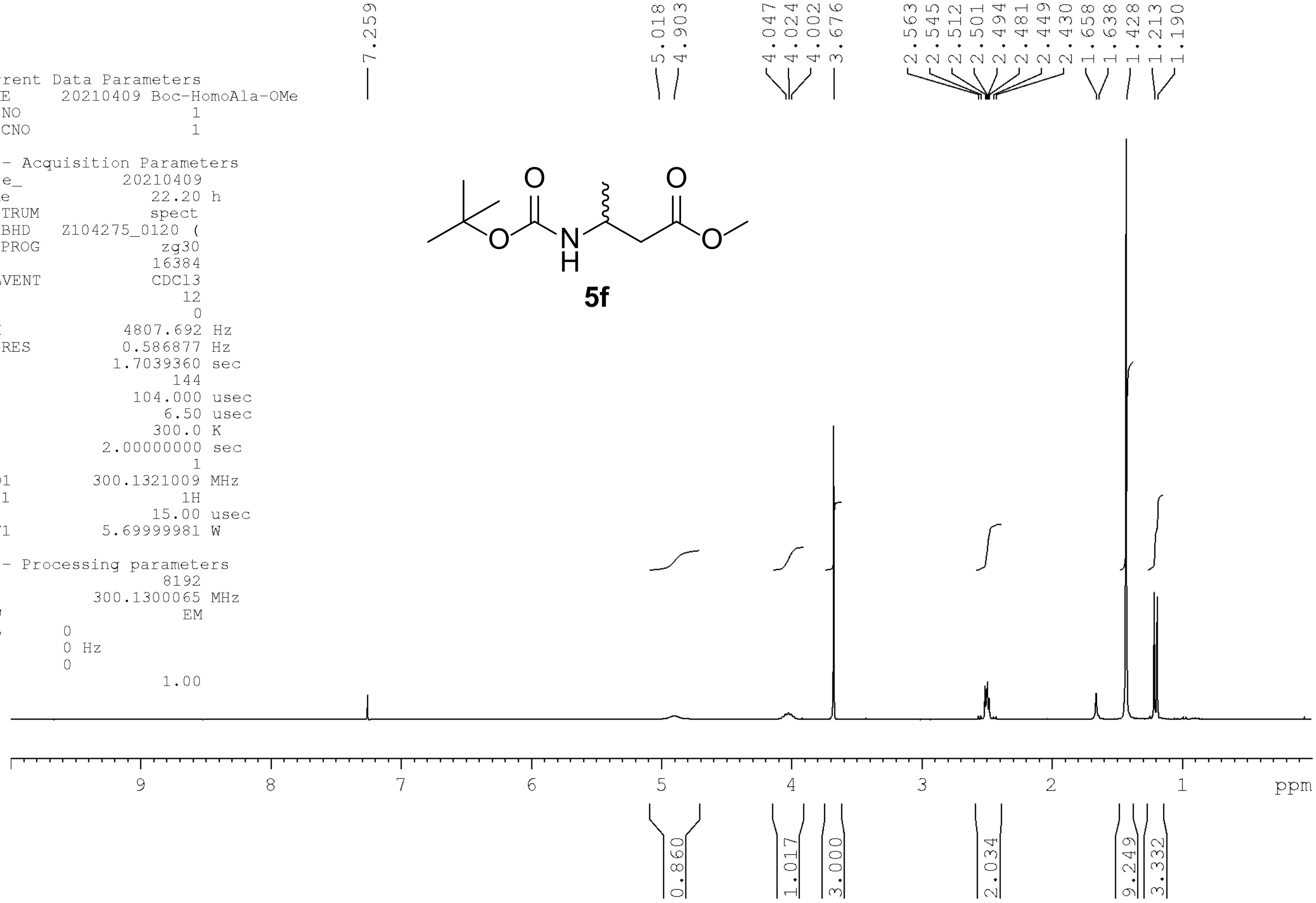

$^{1}$H NMR Spectrum of **5g** (300 MHz, $CDCl_3$)

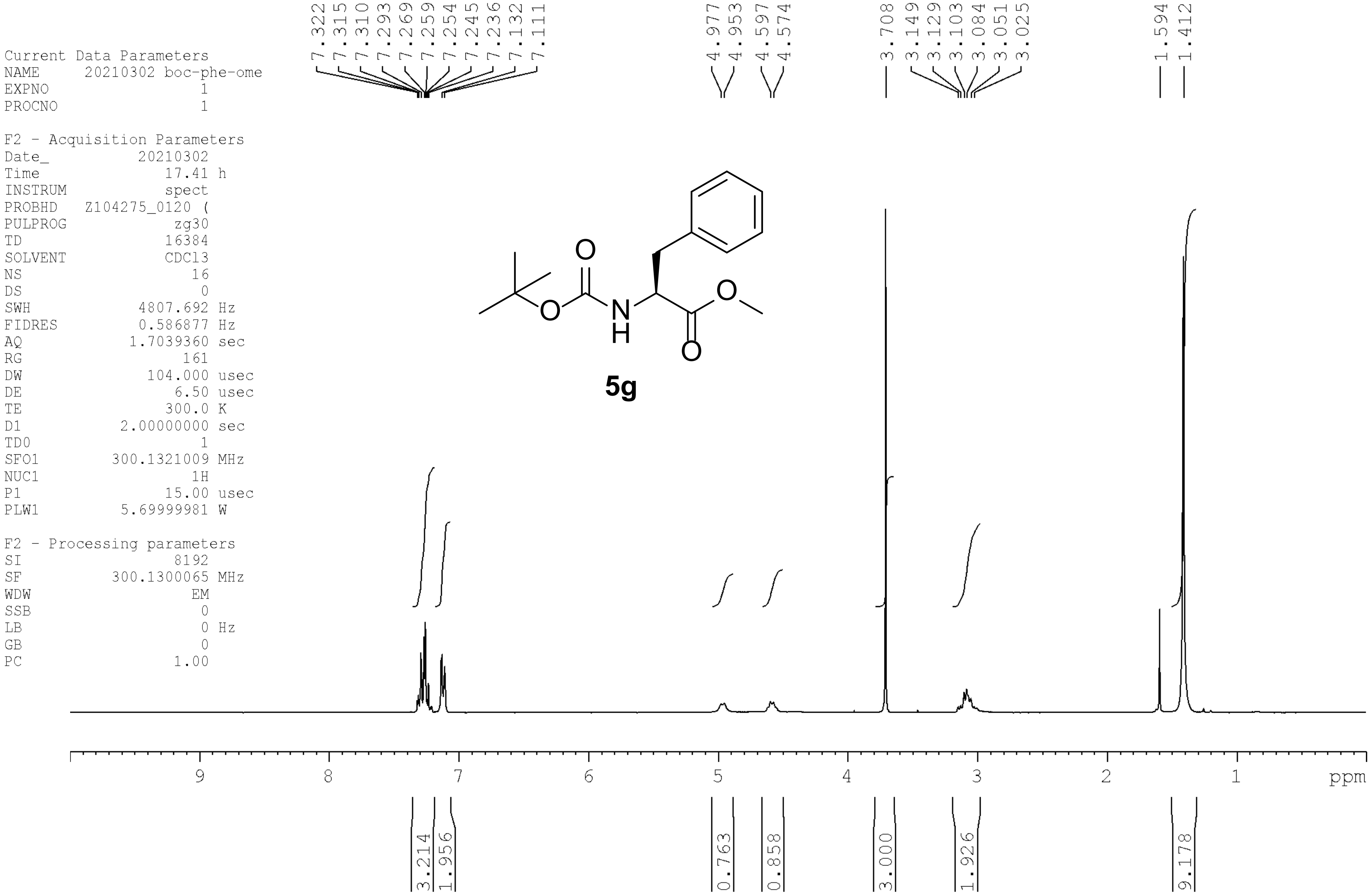

$^{1}$H NMR Spectrum of **5h** (300 MHz, $CDCl_3$)

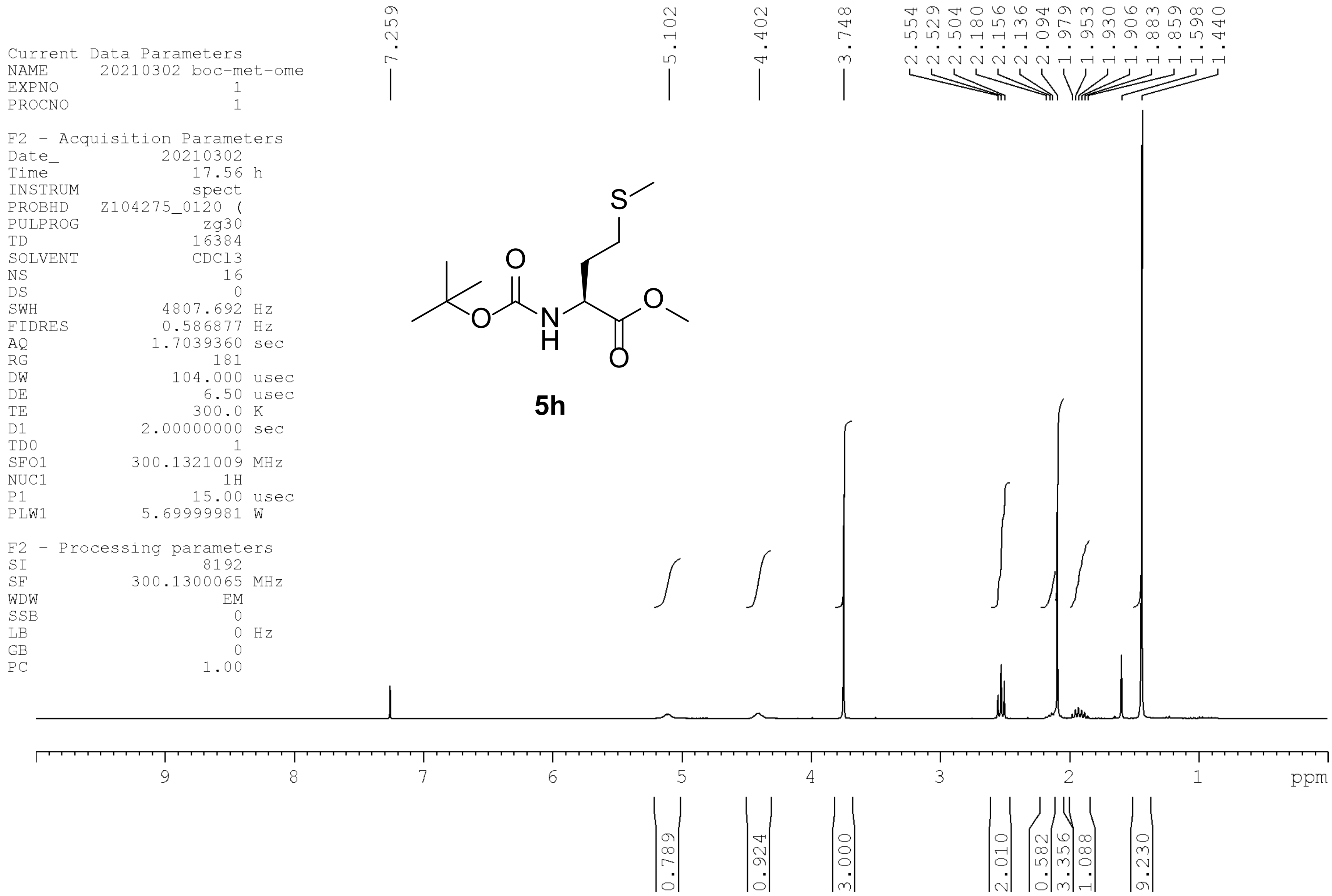

$^{1}$H NMR Spectrum of **5i** (300 MHz, $CDCl_3$)

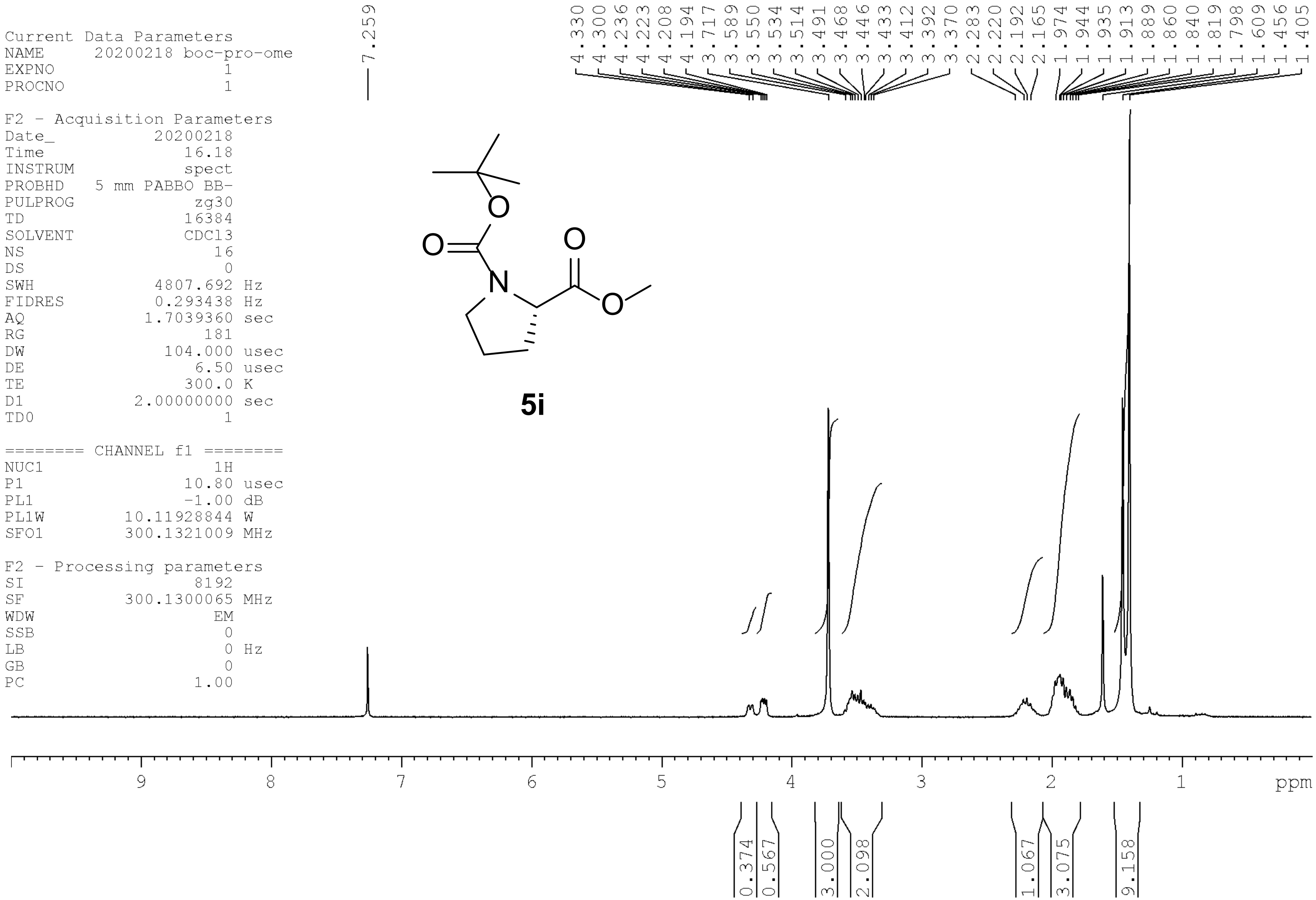

$^{1}$H NMR Spectrum of **5j** (300 MHz, $CDCl_3$)

```
Current Data Parameters
NAME     20210519  boc(tbu)-cys-ome
EXPNO                    1
PROCNO                   1

F2 - Acquisition Parameters
Date_          20210519
Time              17.40 h
INSTRUM           spect
PROBHD   Z104275_0120 (
PULPROG            zg30
TD                16384
SOLVENT           CDCl3
NS                   16
DS                    0
SWH            4807.692 Hz
FIDRES         0.586877 Hz
AQ            1.7039360 sec
RG                  181
DW              104.000 usec
DE                 6.50 usec
TE                300.0 K
D1           2.00000000 sec
TD0                   1
SFO1        300.1321009 MHz
NUC1                 1H
P1                15.00 usec
PLW1         5.69999981 W

F2 - Processing parameters
SI                 8192
SF          300.1300076 MHz
WDW                  EM
SSB                   0
LB                    0 Hz
GB                    0
PC                 1.00
```

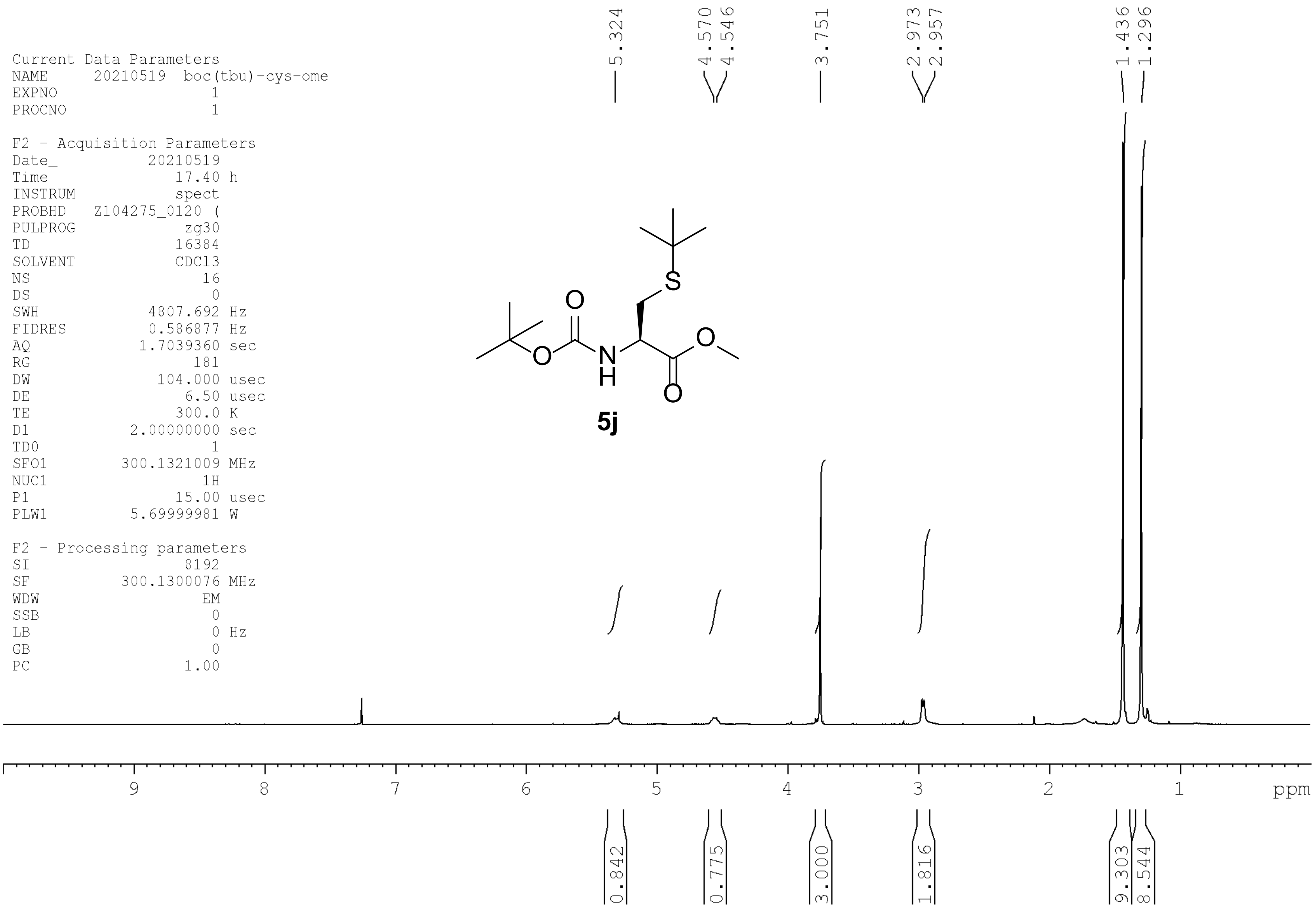

**5j**

$^{1}$H NMR Spectrum of **5k** (300 MHz, $CD_3OD$)

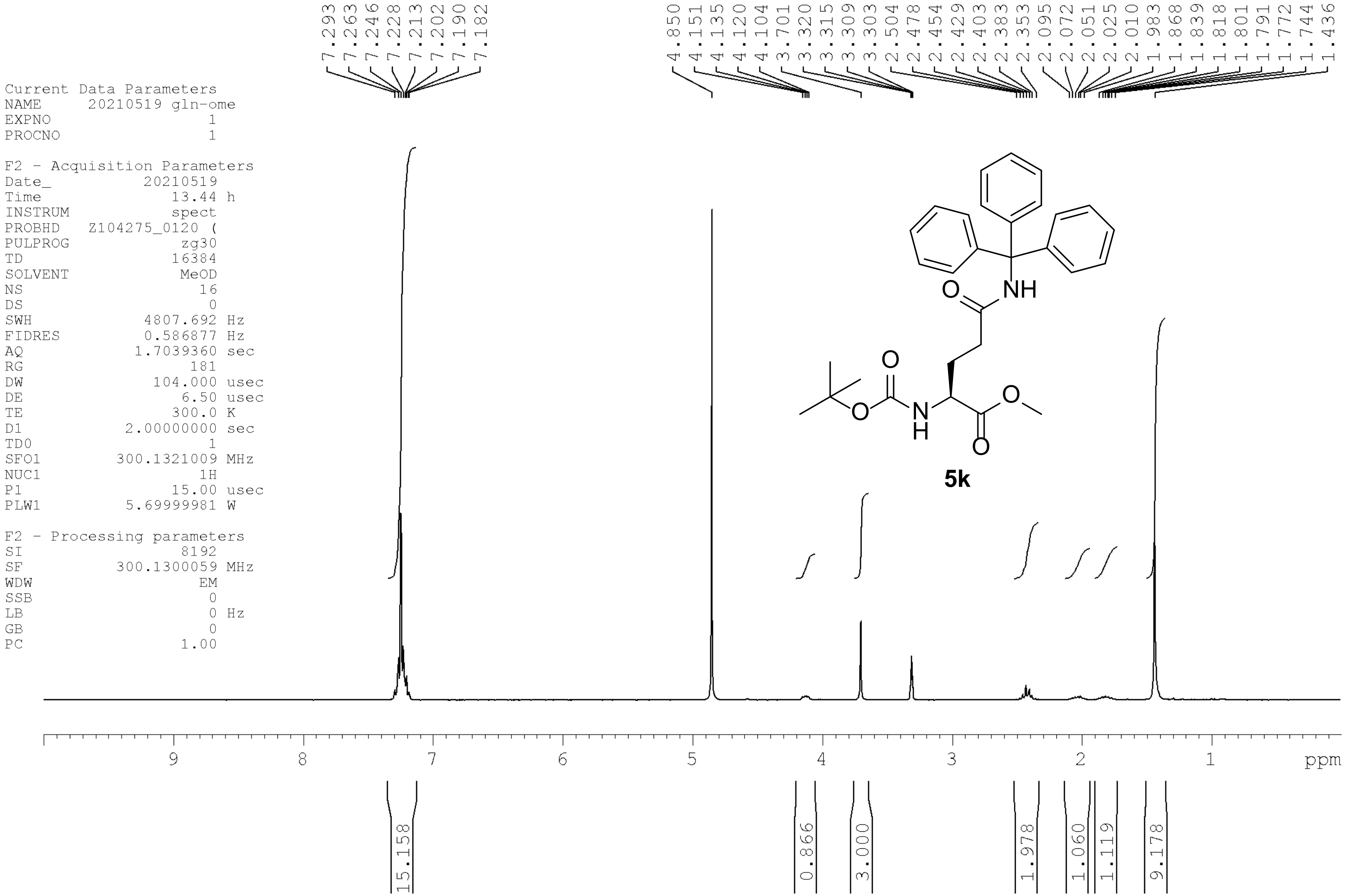

## $^{1}H$ NMR Spectrum of **5l** (300 MHz, $CDCl_3$)

```
Current Data Parameters
NAME     20200903 boc-trt-asn-ome
EXPNO                   1
PROCNO                  1

F2 - Acquisition Parameters
Date_            20200903
Time                17.35
INSTRUM             spect
PROBHD   5 mm PABBO BB-
PULPROG              zg30
TD                  16384
SOLVENT             CDCl3
NS                     16
DS                      0
SWH              4807.692 Hz
FIDRES           0.293438 Hz
AQ              1.7039360 sec
RG                    181
DW                104.000 usec
DE                   6.50 usec
TE                  300.0 K
D1             2.00000000 sec
TD0                     1

======== CHANNEL f1 ========
NUC1                   1H
P1                  10.80 usec
PL1                 -1.00 dB
PL1W          10.11928844 W
SFO1          300.1321009 MHz

F2 - Processing parameters
SI                   8192
SF            300.1300083 MHz
WDW                    EM
SSB                     0
LB                      0 Hz
GB                      0
PC                   1.00
```

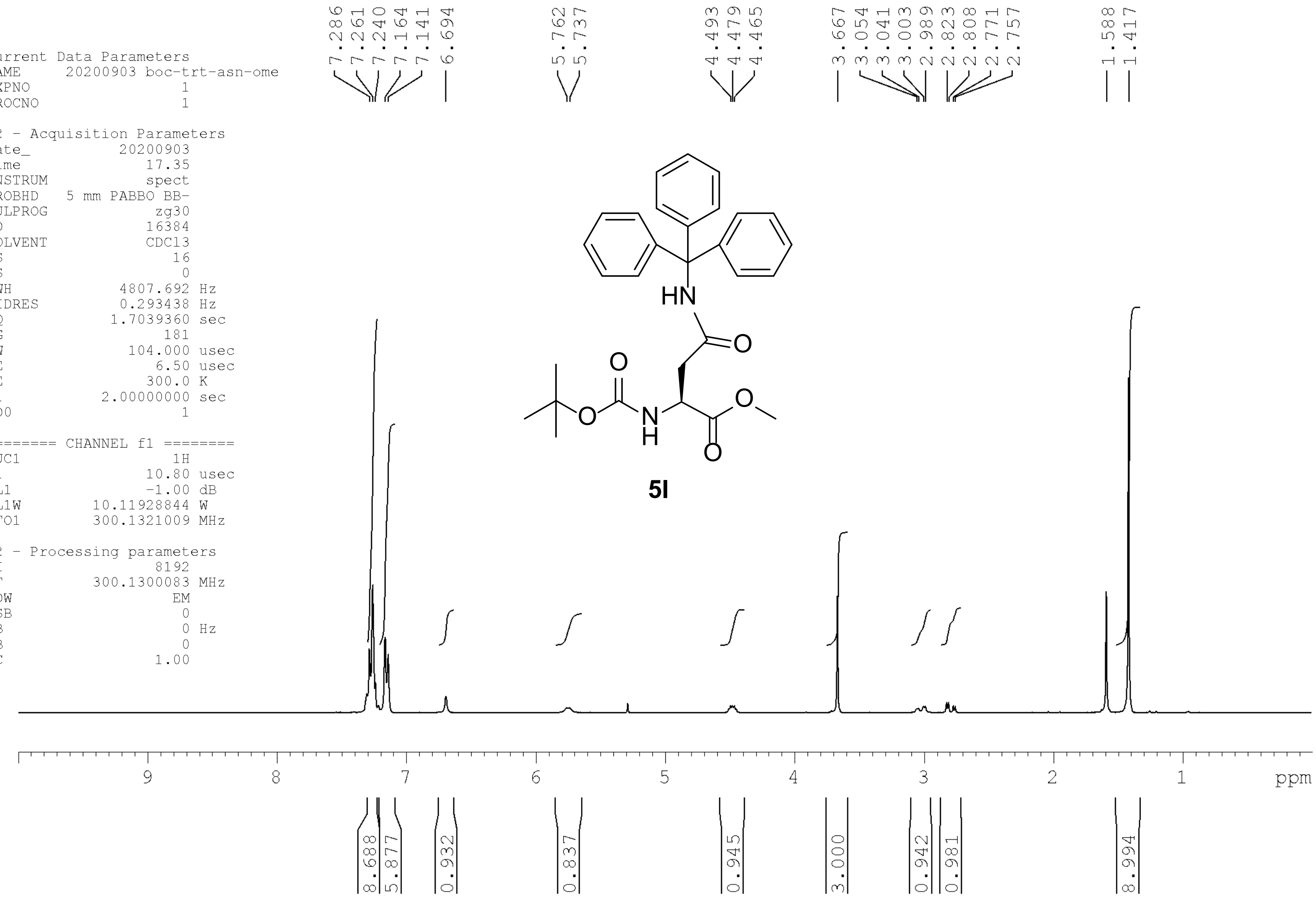

$^{13}C\{^{1}H\}$ NMR Spectrum of **5l** (100 MHz, $CDCl_3$)

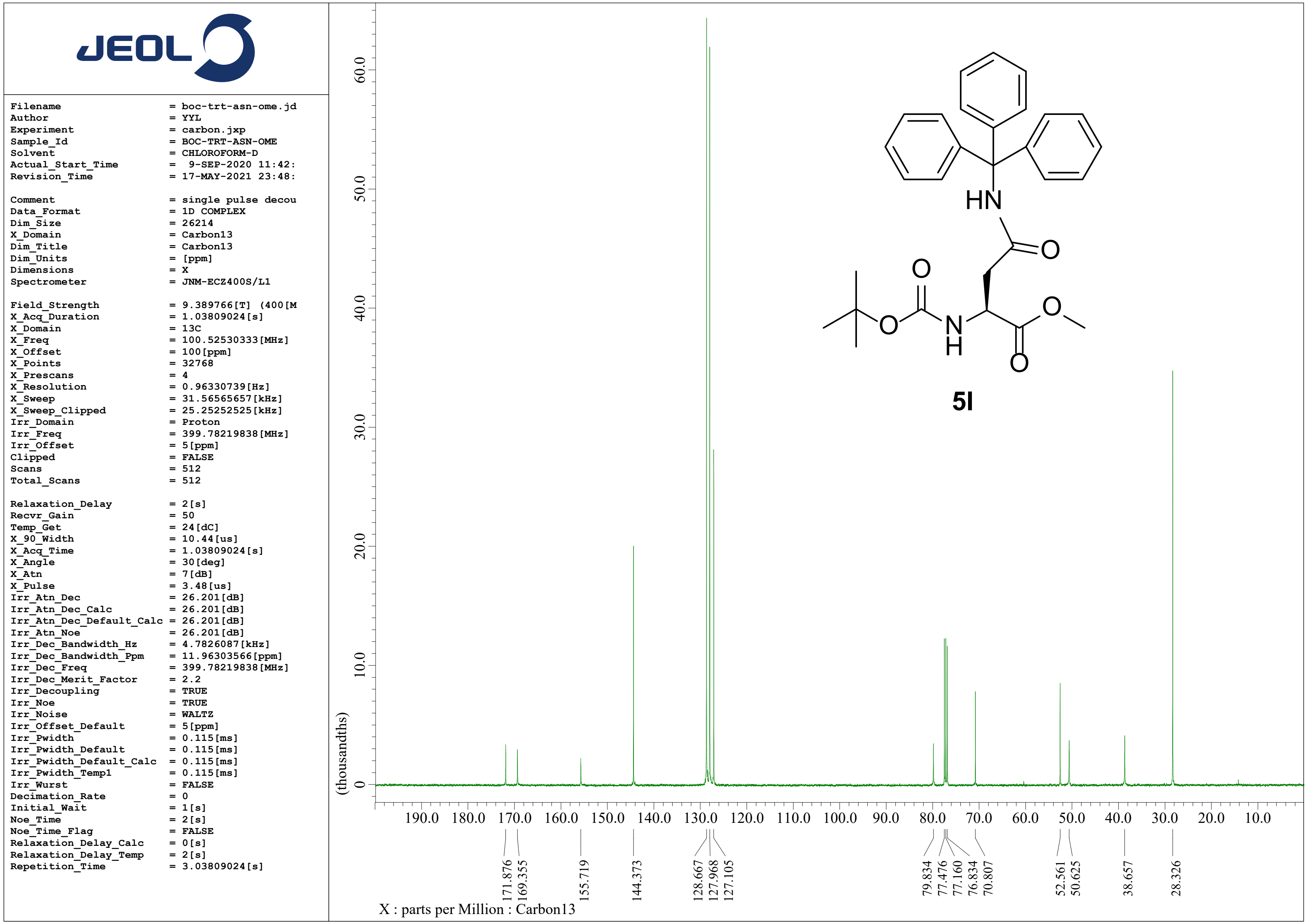

$^{1}$H NMR Spectrum of **5m** (300 MHz, $CDCl_3$)

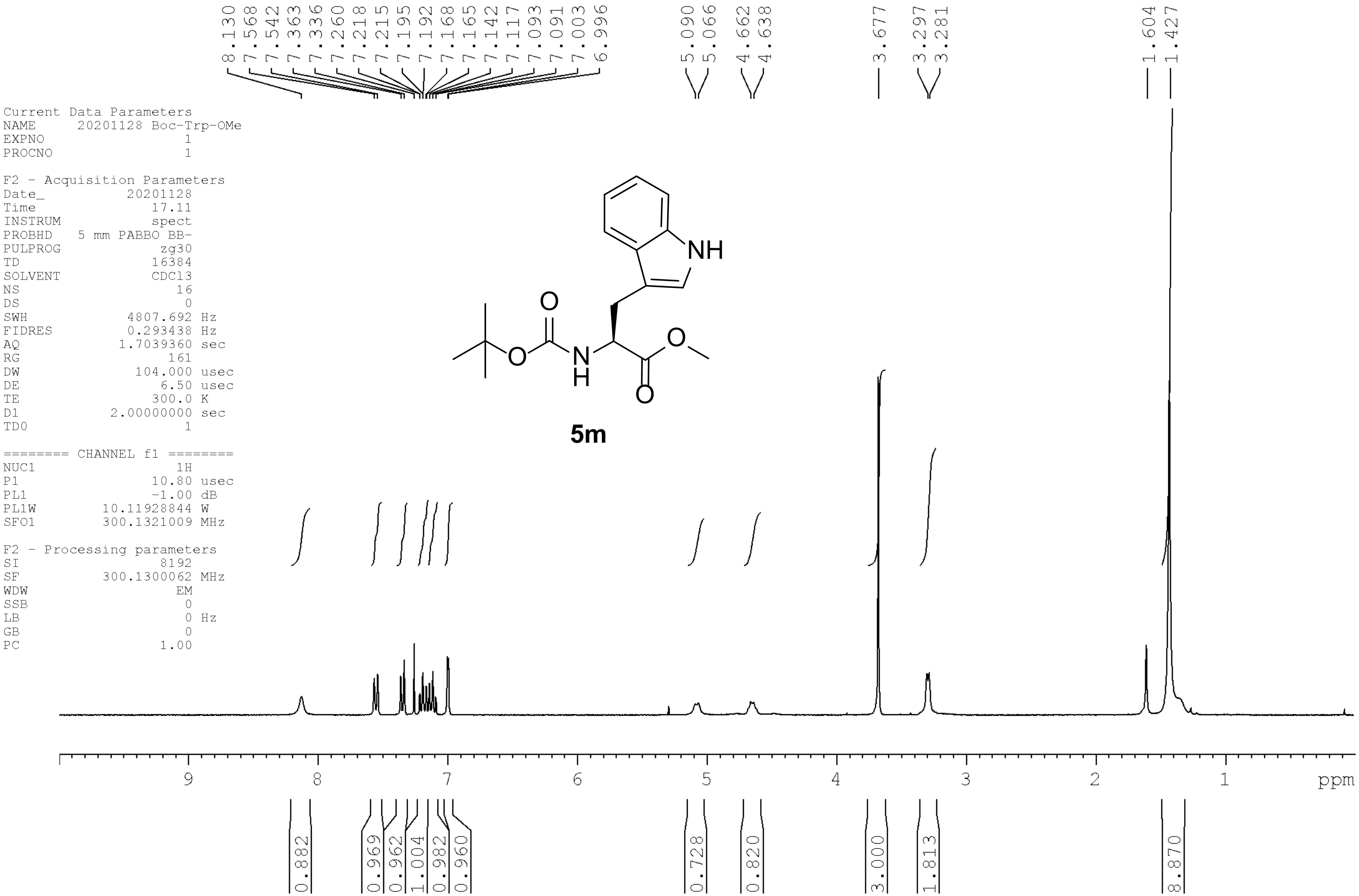

$^{1}$H NMR Spectrum of **5n** (300 MHz, $CDCl_3$)

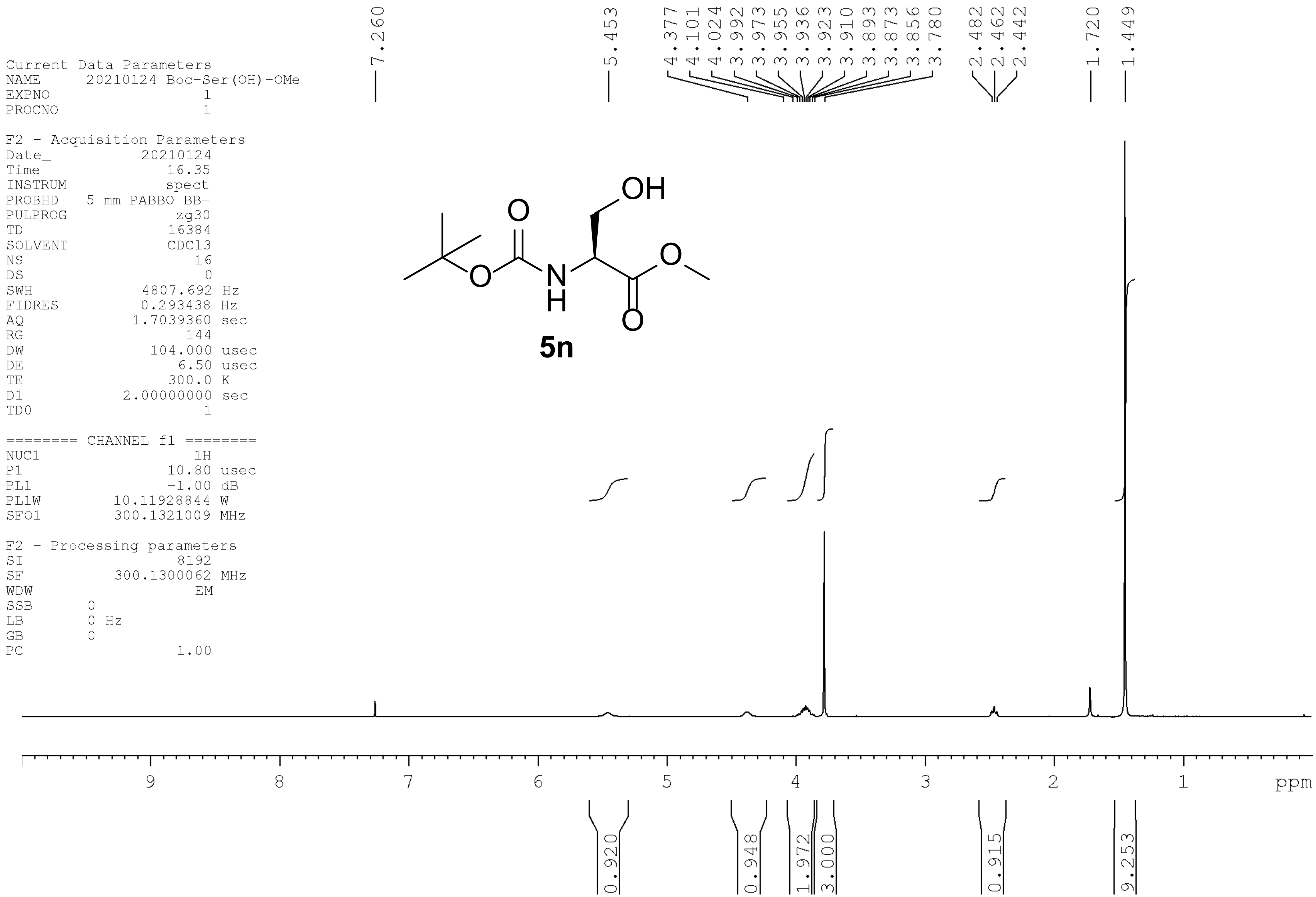

$^{1}$H NMR Spectrum of **5o** (300 MHz, $CDCl_3$)

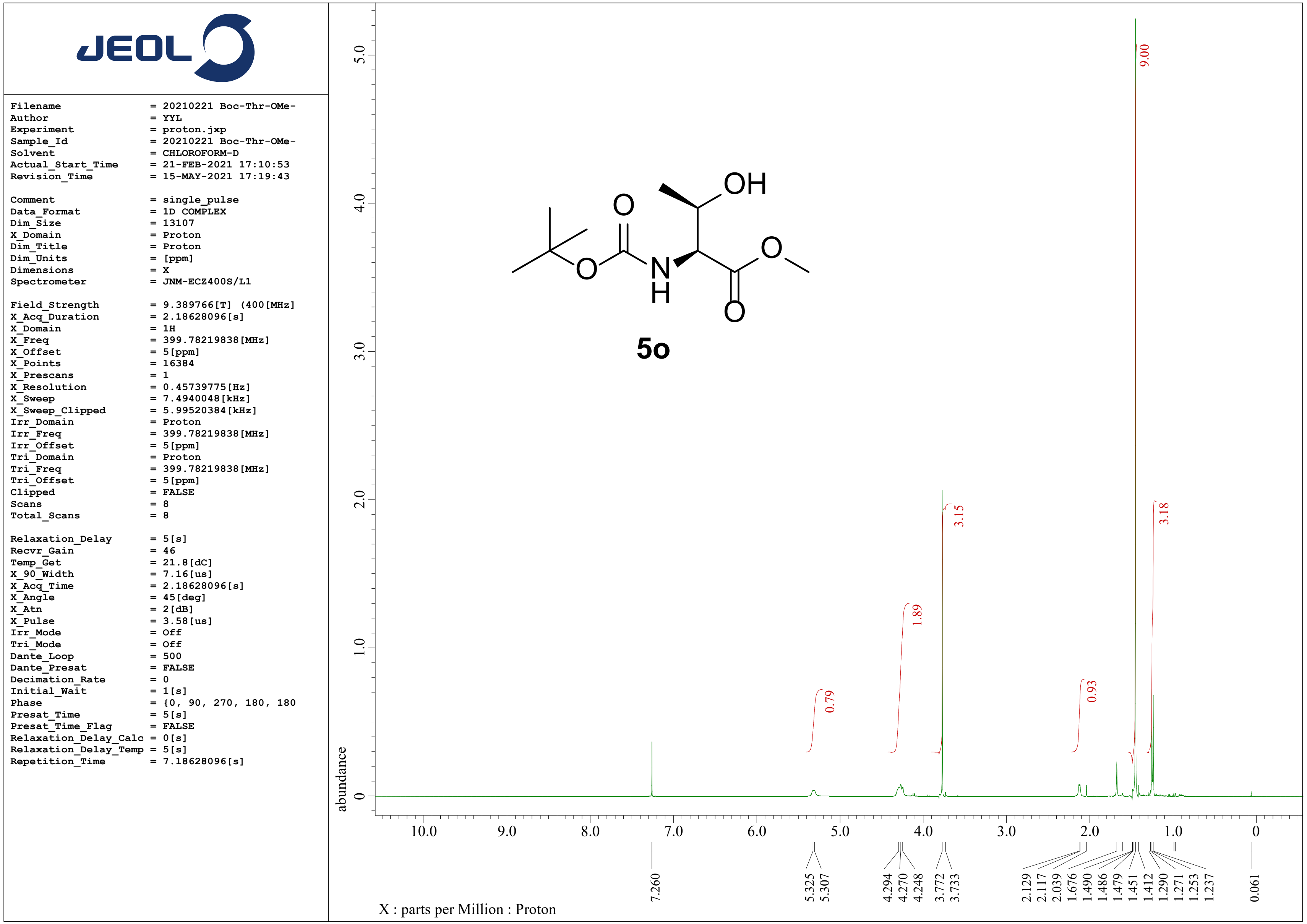

$^{1}$H NMR Spectrum of **5p** (300 MHz, $CDCl_3$)

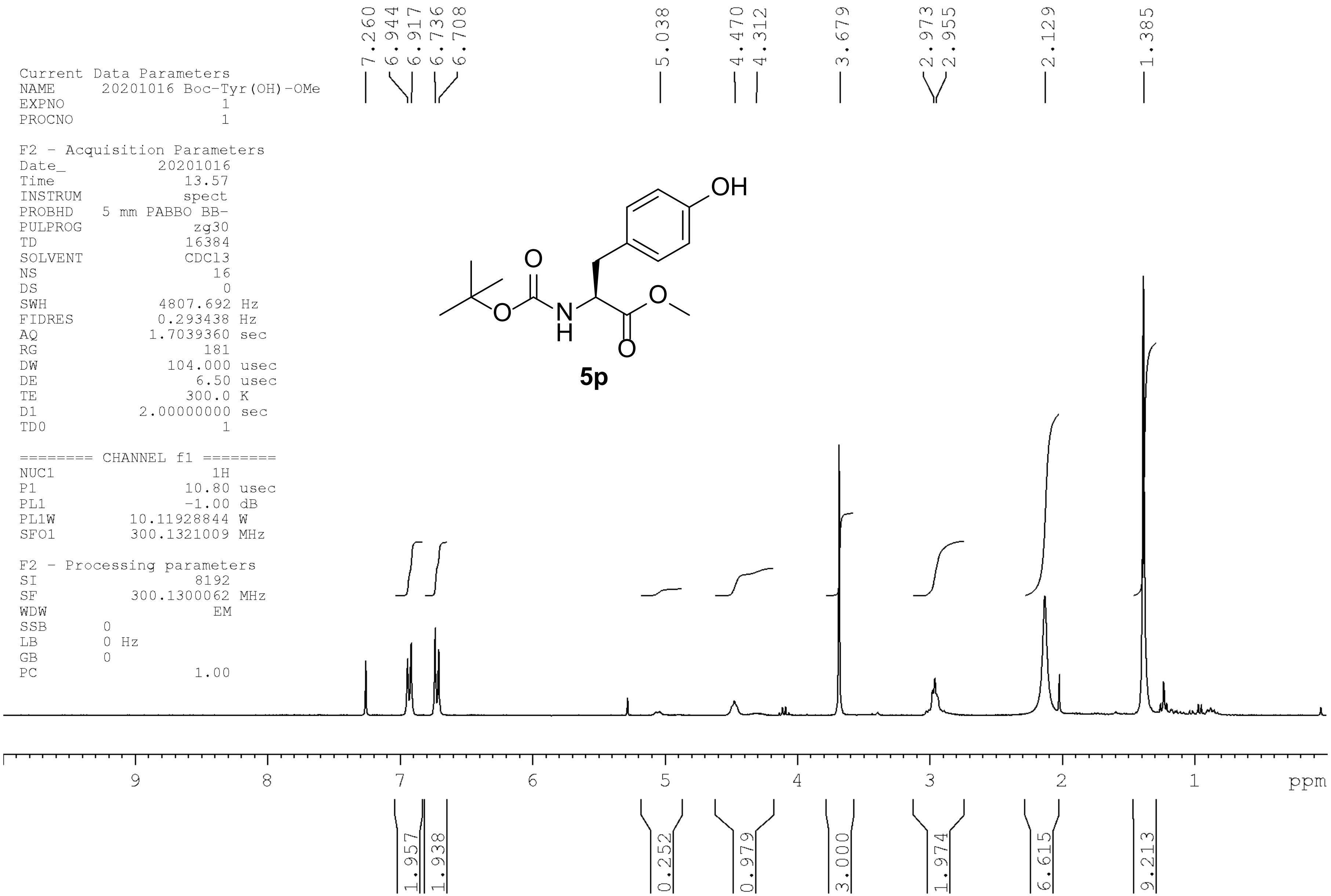

$^{1}$H NMR Spectrum of **5q** (300 MHz, $CDCl_3$)

```
Current Data Parameters
NAME      20210319 Boc-Ser(OtBu)-OMe
EXPNO                  1
PROCNO                 1

F2 - Acquisition Parameters
Date_          20210319
Time              22.35 h
INSTRUM           spect
PROBHD   Z104275_0120 (
PULPROG            zg30
TD                16384
SOLVENT           CDCl3
NS                   12
DS                    0
SWH            4807.692 Hz
FIDRES         0.586877 Hz
AQ            1.7039360 sec
RG                  161
DW              104.000 usec
DE                 6.50 usec
TE                300.0 K
D1           2.00000000 sec
TD0                   1
SFO1        300.1321009 MHz
NUC1                 1H
P1                15.00 usec
PLW1         5.69999981 W

F2 - Processing parameters
SI                 8192
SF          300.1300065 MHz
WDW                  EM
SSB      0
LB       0 Hz
GB       0
PC                 1.00
```

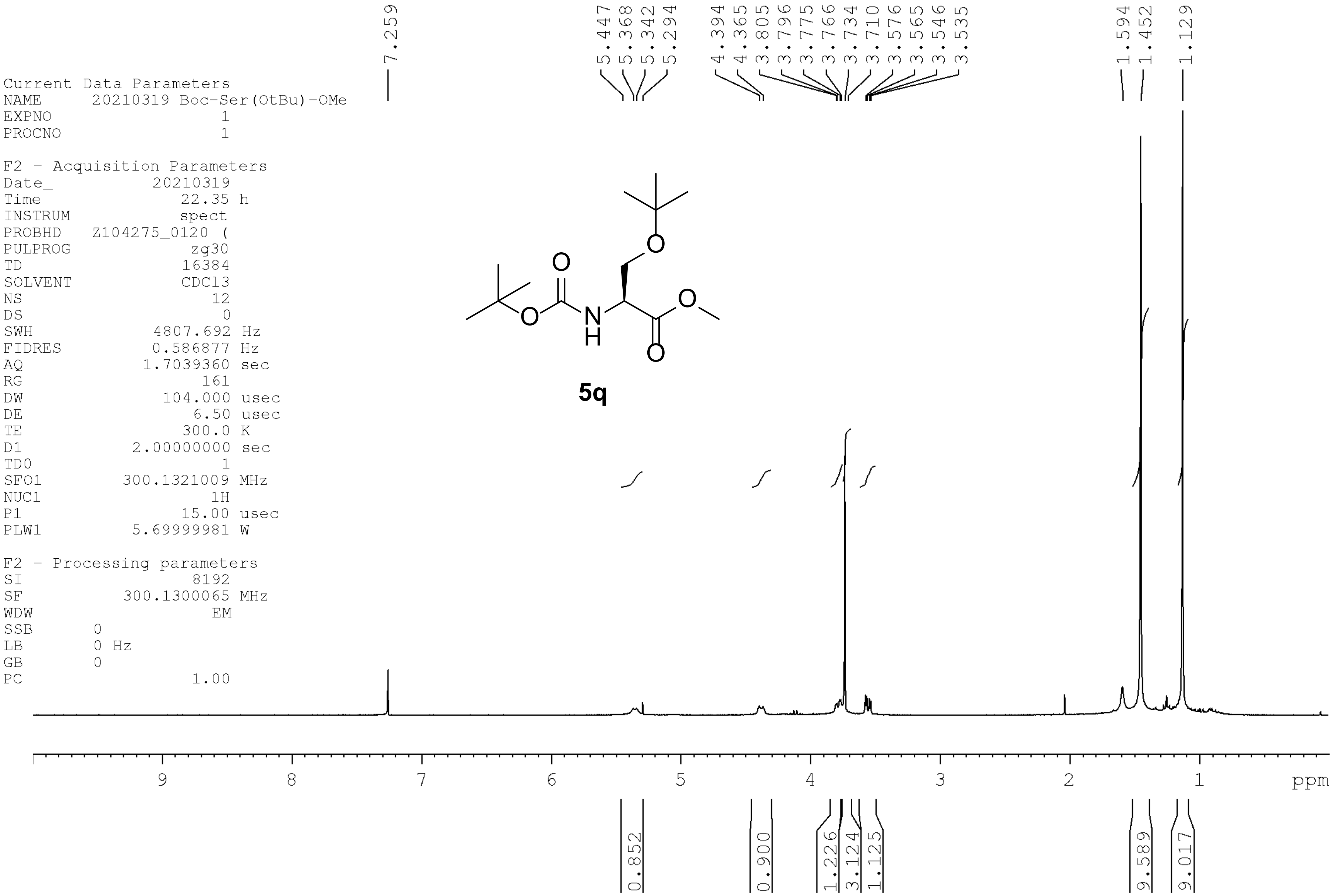

$^{1}$H NMR Spectrum of **5r** (300 MHz, $CDCl_3$)

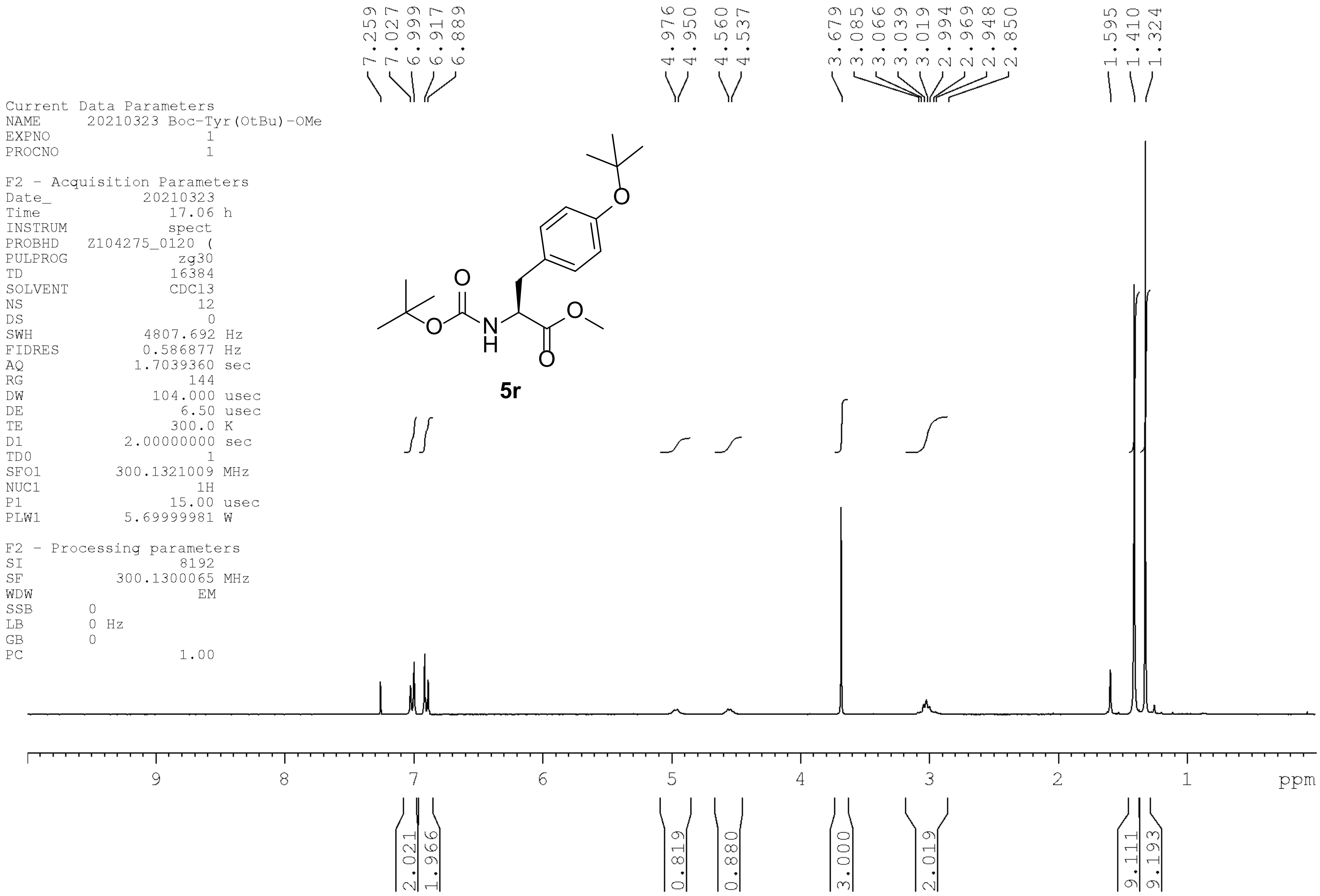

$^{1}$H NMR Spectrum of **5s** (300 MHz, $CDCl_3$)

```
Current Data Parameters
NAME      20201218 Boc-Phg-OMe
EXPNO                    1
PROCNO                   1

F2 - Acquisition Parameters
Date_          20201218
Time              16.29
INSTRUM           spect
PROBHD   5 mm PABBO BB-
PULPROG            zg30
TD                16384
SOLVENT           CDCl3
NS                   16
DS                    0
SWH            4807.692 Hz
FIDRES         0.293438 Hz
AQ            1.7039360 sec
RG                  161
DW              104.000 usec
DE                 6.50 usec
TE                300.0 K
D1           2.00000000 sec
TD0                   1

======== CHANNEL f1 ========
NUC1                 1H
P1                10.80 usec
PL1               -1.00 dB
PL1W        10.11928844 W
SFO1        300.1321009 MHz

F2 - Processing parameters
SI                 8192
SF          300.1300062 MHz
WDW                  EM
SSB       0
LB        0 Hz
GB        0
PC                 1.00
```

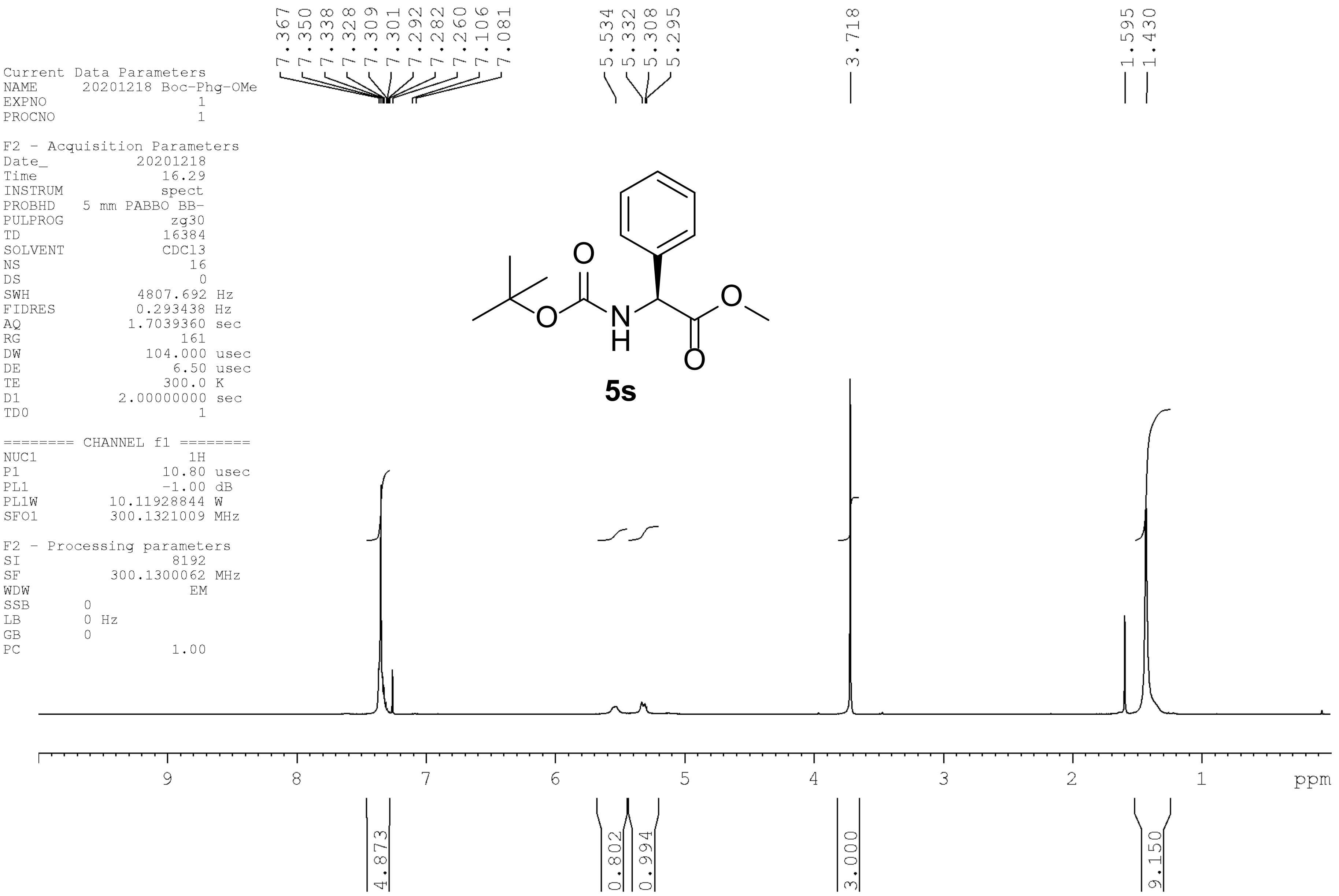

$^{1}$H NMR Spectrum of **5t** (300 MHz, $CDCl_3$)

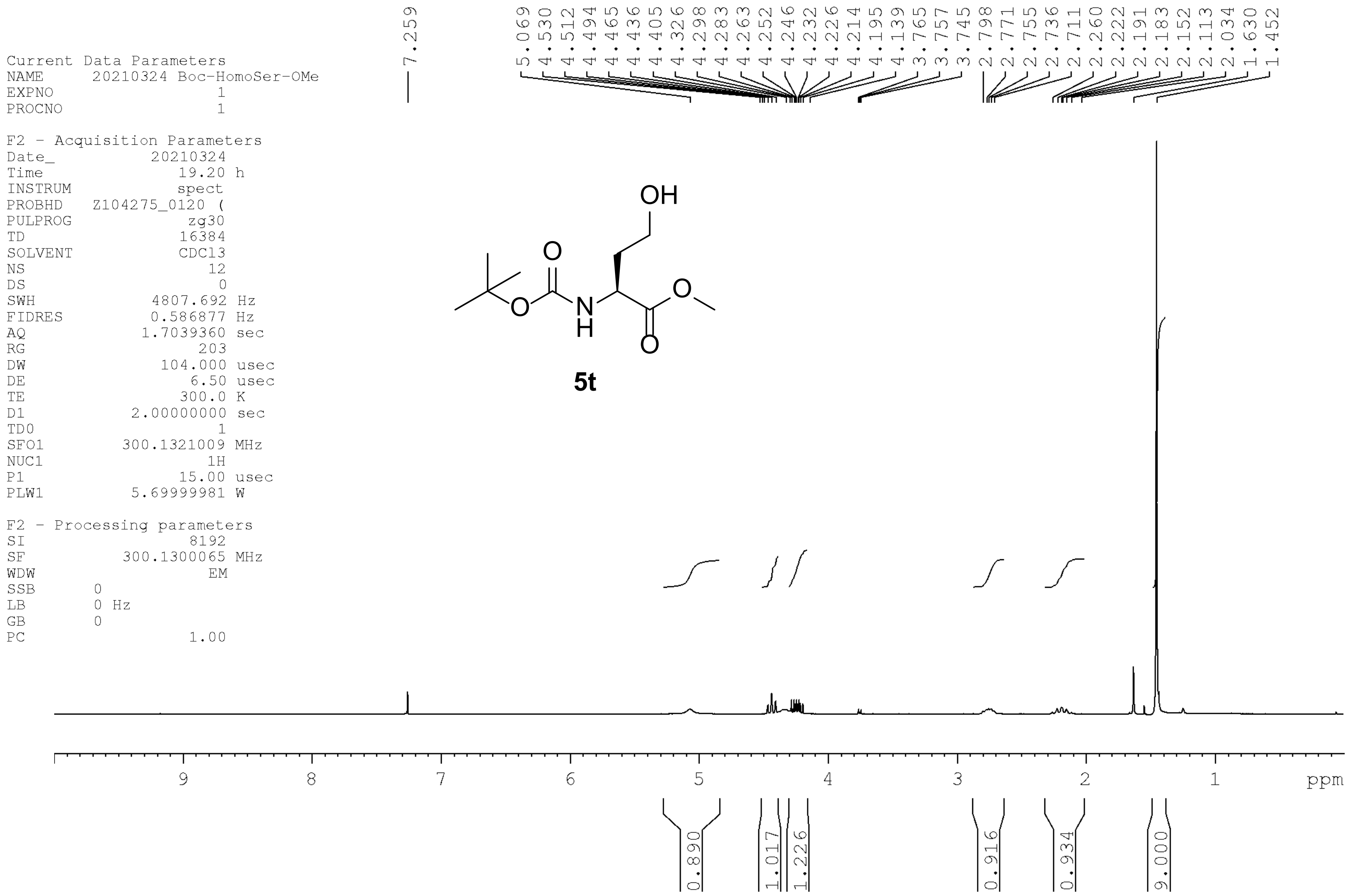

$^{1}$H NMR Spectrum of **5u** (300 MHz, $CDCl_3$)

```
Current Data Parameters
NAME      20190912boc-ala-gly-ome-1.1
EXPNO                  1
PROCNO                 1

F2 - Acquisition Parameters
Date_           20190912
Time               14.02
INSTRUM            spect
PROBHD   5 mm PABBO BB-
PULPROG             zg30
TD                 16384
SOLVENT            CDCl3
NS                    16
DS                     0
SWH             4807.692 Hz
FIDRES          0.293438 Hz
AQ             1.7039360 sec
RG                   181
DW               104.000 usec
DE                  6.50 usec
TE                 300.0 K
D1            2.00000000 sec
TD0                    1

======== CHANNEL f1 ========
NUC1                  1H
P1                 10.80 usec
PL1                -1.00 dB
PL1W         10.11928844 W
SFO1         300.1321009 MHz

F2 - Processing parameters
SI                  8192
SF           300.1300067 MHz
WDW                   EM
SSB                    0
LB                     0 Hz
GB                     0
PC                  1.00
```

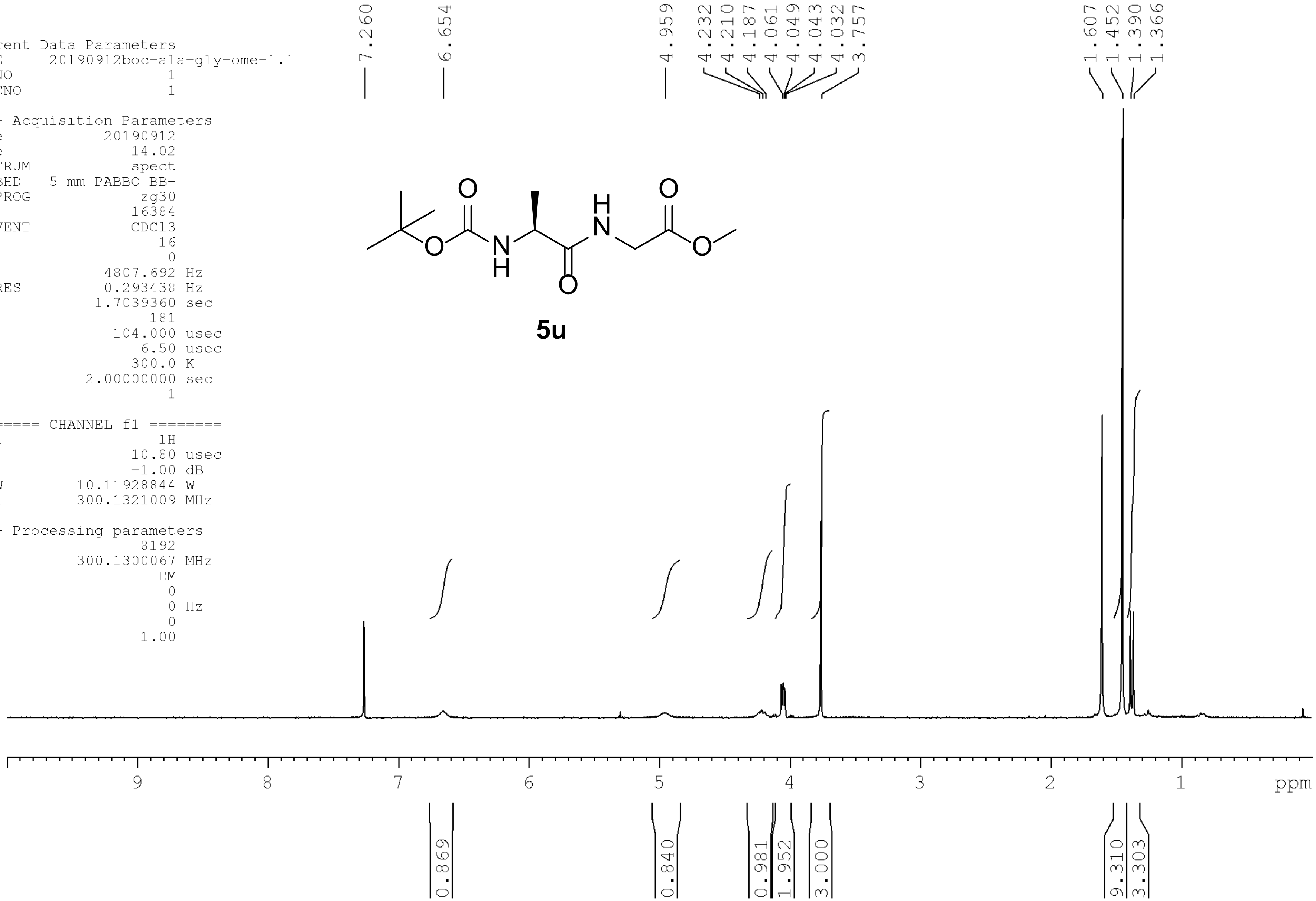


**5u**

$^{1}$H NMR Spectrum of **6aa** (300 MHz, $CDCl_3$)

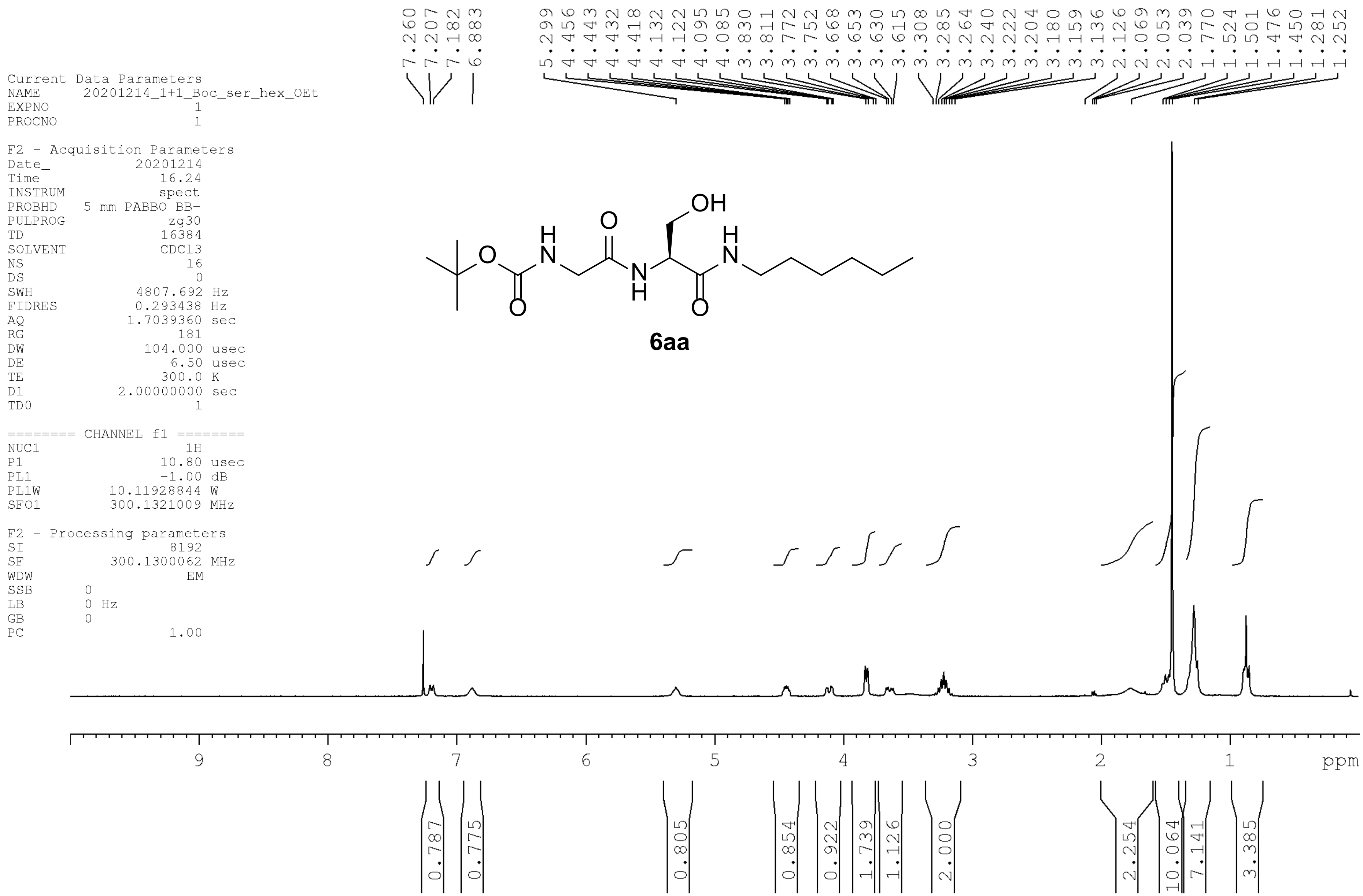

## $^{13}C\{^{1}H\}$ NMR Spectrum of **6aa** (100 MHz, $CDCl_3$)

JEOL

| | |
|---|---|
| Filename | = (9a)ser-hex+boc-gl |
| Author | = YYL |
| Experiment | = carbon.jxp |
| Sample_Id | = 20181128 2-2 C |
| Solvent | = CHLOROFORM-D |
| Actual_Start_Time | = 11-DEC-2018 12:51: |
| Revision_Time | = 22-JUL-2019 12:23: |
| Comment | = single pulse decou |
| Data_Format | = 1D COMPLEX |
| Dim_Size | = 26214 |
| X_Domain | = Carbon13 |
| Dim_Title | = Carbon13 |
| Dim_Units | = [ppm] |
| Dimensions | = X |
| Spectrometer | = JNM-ECZ400S/L1 |
| Field_Strength | = 9.389766[T] (400[M |
| X_Acq_Duration | = 1.03809024[s] |
| X_Domain | = 13C |
| X_Freq | = 100.52530333[MHz] |
| X_Offset | = 100[ppm] |
| X_Points | = 32768 |
| X_Prescans | = 4 |
| X_Resolution | = 0.96330739[Hz] |
| X_Sweep | = 31.56565657[kHz] |
| X_Sweep_Clipped | = 25.25252525[kHz] |
| Irr_Domain | = Proton |
| Irr_Freq | = 399.78219838[MHz] |
| Irr_Offset | = 5[ppm] |
| Clipped | = FALSE |
| Scans | = 1024 |
| Total_Scans | = 1024 |
| Relaxation_Delay | = 2[s] |
| Recvr_Gain | = 50 |
| Temp_Get | = 24.7[dC] |
| X_90_Width | = 10.6[us] |
| X_Acq_Time | = 1.03809024[s] |
| X_Angle | = 30[deg] |
| X_Atn | = 7[dB] |
| X_Pulse | = 3.53333333[us] |
| Irr_Atn_Dec | = 26.437[dB] |
| Irr_Atn_Dec_Calc | = 26.437[dB] |
| Irr_Atn_Dec_Default_Calc | = 26.437[dB] |
| Irr_Atn_Noe | = 26.437[dB] |
| Irr_Dec_Bandwidth_Hz | = 4.7826087[kHz] |
| Irr_Dec_Bandwidth_Ppm | = 11.96303566[ppm] |
| Irr_Dec_Freq | = 399.78219838[MHz] |
| Irr_Dec_Merit_Factor | = 2.2 |
| Irr_Decoupling | = TRUE |
| Irr_Noe | = TRUE |
| Irr_Noise | = WALTZ |
| Irr_Offset_Default | = 5[ppm] |
| Irr_Pwidth | = 0.115[ms] |
| Irr_Pwidth_Default | = 0.115[ms] |
| Irr_Pwidth_Default_Calc | = 0.115[ms] |
| Irr_Pwidth_Temp1 | = 0.115[ms] |
| Irr_Wurst | = FALSE |
| Decimation_Rate | = 0 |
| Initial_Wait | = 1[s] |
| Noe_Time | = 2[s] |
| Noe_Time_Flag | = FALSE |
| Relaxation_Delay_Calc | = 0[s] |
| Relaxation_Delay_Temp | = 2[s] |
| Repetition_Time | = 3.03809024[s] |

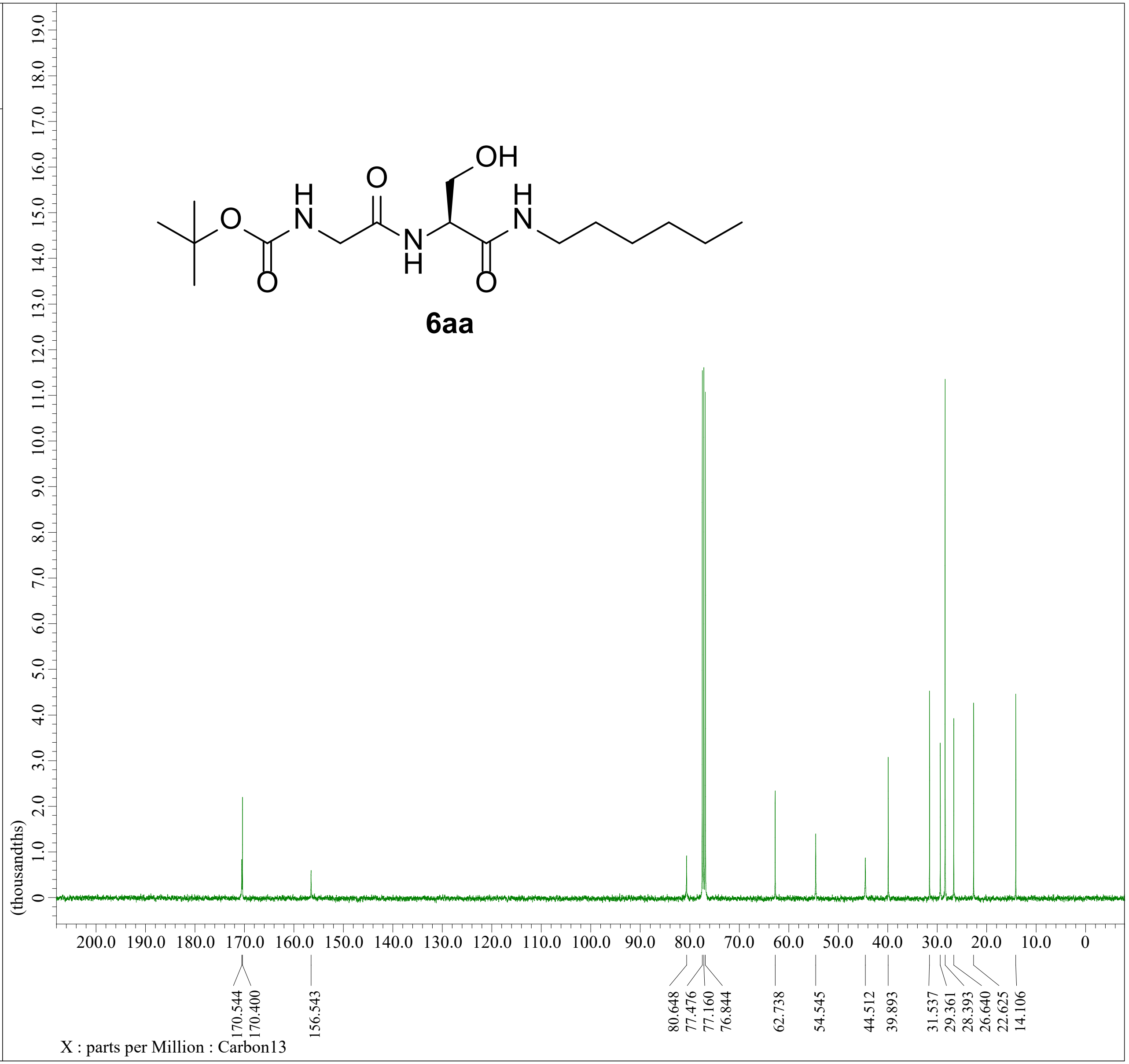

$^{1}$H NMR Spectrum of **6ab** (300 MHz, $CD_3OD$)

```
Current Data Parameters
NAME      20210421 Cbz-Gly-Ser-Hex
EXPNO                    1
PROCNO                   1

F2 - Acquisition Parameters
Date_            20210421
Time                20.50 h
INSTRUM             spect
PROBHD    Z104275_0120 (
PULPROG              zg30
TD                  16384
SOLVENT              MeOD
NS                     12
DS                      0
SWH              4807.692 Hz
FIDRES           0.586877 Hz
AQ              1.7039360 sec
RG                    144
DW                104.000 usec
DE                   6.50 usec
TE                  300.0 K
D1             2.00000000 sec
TD0                     1
SFO1          300.1321009 MHz
NUC1                   1H
P1                  15.00 usec
PLW1           5.69999981 W

F2 - Processing parameters
SI                   8192
SF            300.1300059 MHz
WDW                    EM
SSB       0
LB        0 Hz
GB        0
PC                   1.00
```

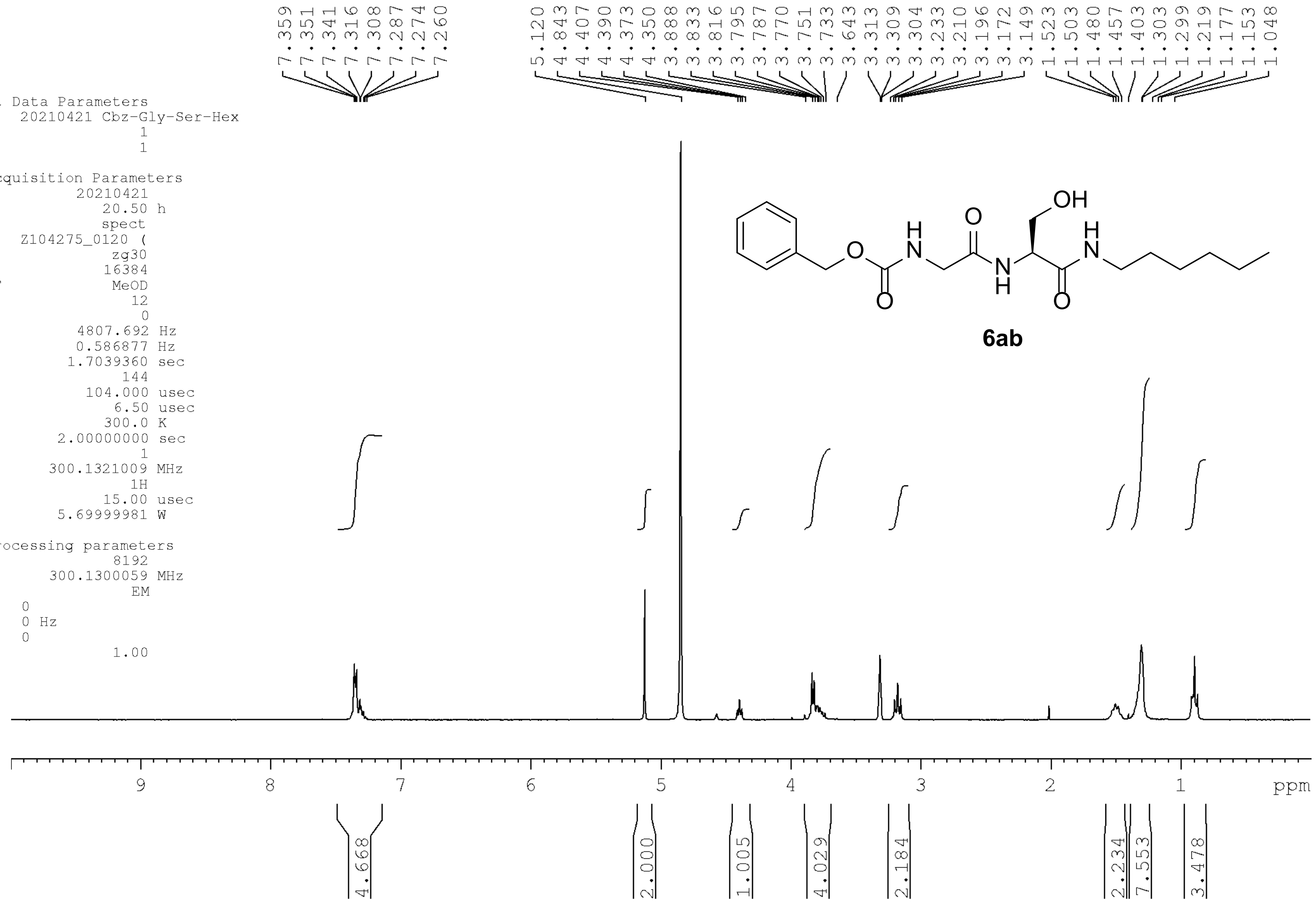

$^{13}C\{^1H\}$ NMR Spectrum of **6ab** (75 MHz, DMSO-$d_6$)

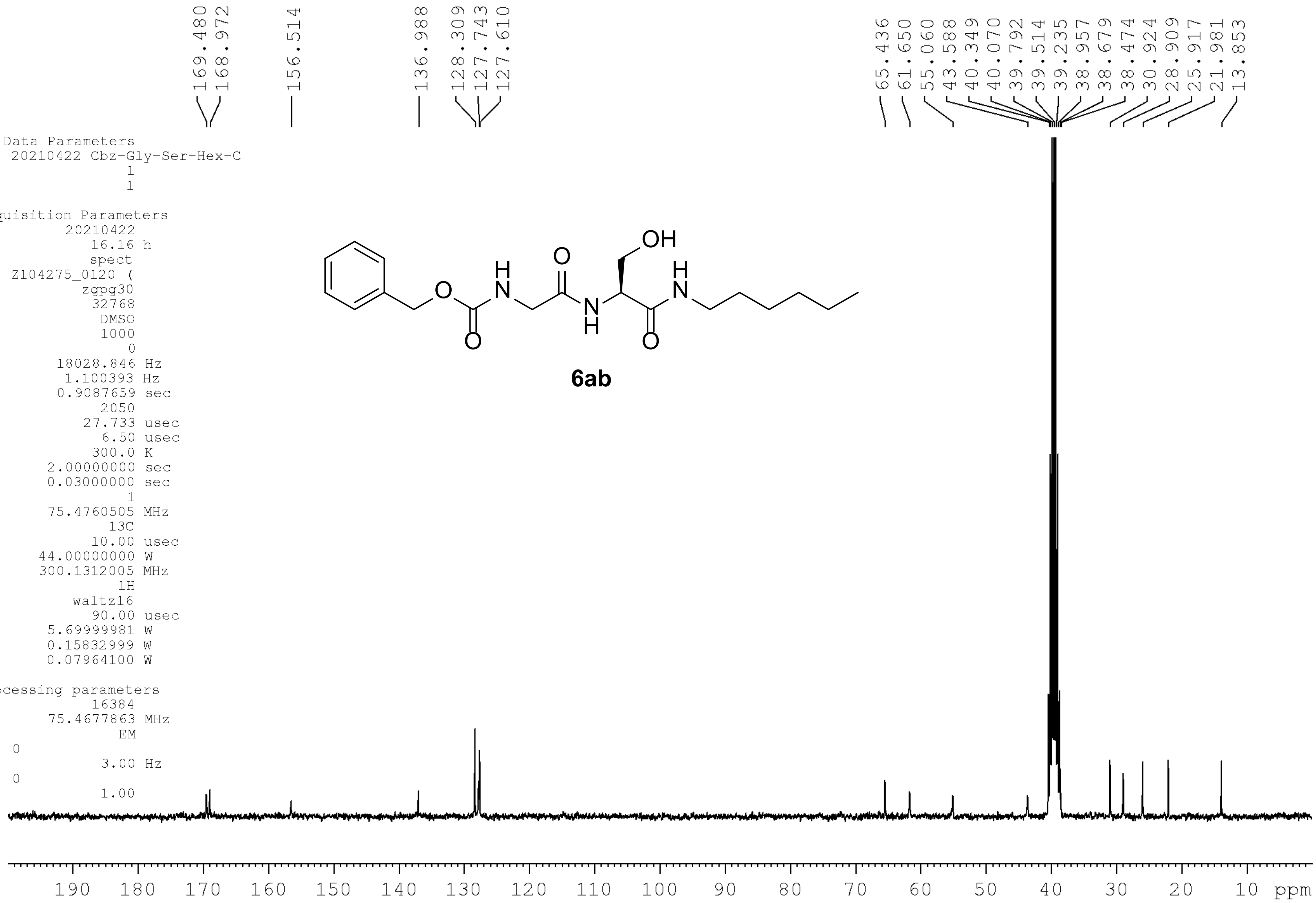


**6ab**

```
Current Data Parameters
NAME       20210422 Cbz-Gly-Ser-Hex-C
EXPNO                     1
PROCNO                    1

F2 - Acquisition Parameters
Date_            20210422
Time                16.16 h
INSTRUM             spect
PROBHD    Z104275_0120 (
PULPROG           zgpg30
TD                 32768
SOLVENT             DMSO
NS                  1000
DS                     0
SWH            18028.846 Hz
FIDRES          1.100393 Hz
AQ             0.9087659 sec
RG                  2050
DW                27.733 usec
DE                  6.50 usec
TE                 300.0 K
D1            2.00000000 sec
D11           0.03000000 sec
TD0                    1
SFO1          75.4760505 MHz
NUC1                 13C
P1                 10.00 usec
PLW1         44.00000000 W
SFO2         300.1312005 MHz
NUC2                  1H
CPDPRG[2         waltz16
PCPD2              90.00 usec
PLW2          5.69999981 W
PLW12         0.15832999 W
PLW13         0.07964100 W

F2 - Processing parameters
SI                 16384
SF            75.4677863 MHz
WDW                   EM
SSB       0
LB                  3.00 Hz
GB        0
PC                  1.00
```

$^{1}$H NMR Spectrum of **6ad** (300 MHz, DMSO-d$_6$)

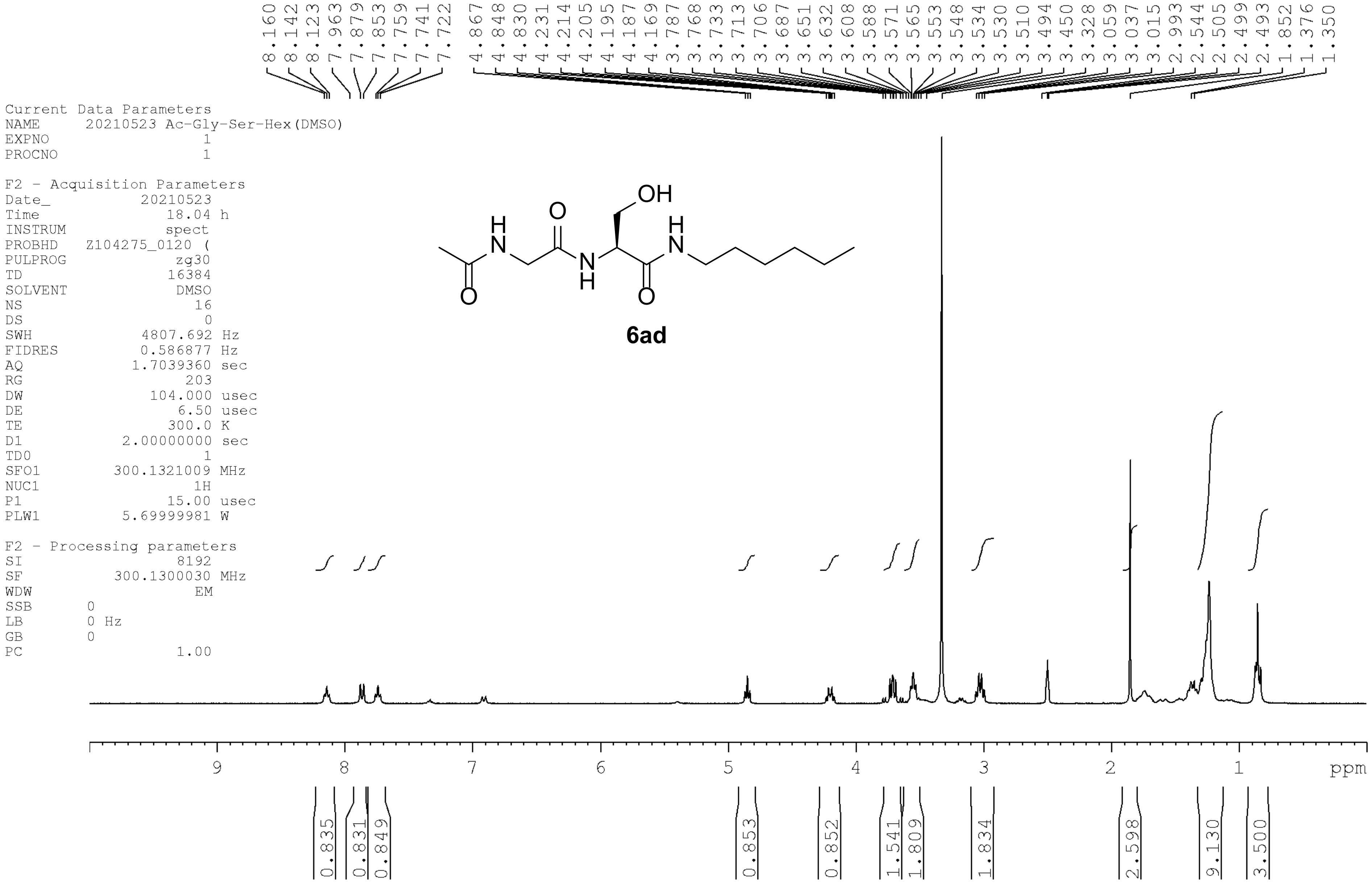

## $^{13}C\{^1H\}$ NMR Spectrum of **6ad** (100 MHz, DMSO-d$_6$)

```
Filename                     = 20210525 Ac-Gly-Se
Author                       = YYL
Experiment                   = carbon.jxp
Sample_Id                    = 20210525 Ac-Gly-Se
Solvent                      = DMSO-D6
Actual_Start_Time            = 25-MAY-2021 11:16:
Revision_Time                =  5-JUN-2021 13:28:

Comment                      = single pulse decou
Data_Format                  = 1D COMPLEX
Dim_Size                     = 26214
X_Domain                     = Carbon13
Dim_Title                    = Carbon13
Dim_Units                    = [ppm]
Dimensions                   = X
Spectrometer                 = JNM-ECZ400S/L1

Field_Strength               = 9.389766[T] (400[M
X_Acq_Duration               = 1.03809024[s]
X_Domain                     = 13C
X_Freq                       = 100.52530333[MHz]
X_Offset                     = 100[ppm]
X_Points                     = 32768
X_Prescans                   = 4
X_Resolution                 = 0.96330739[Hz]
X_Sweep                      = 31.56565657[kHz]
X_Sweep_Clipped              = 25.25252525[kHz]
Irr_Domain                   = Proton
Irr_Freq                     = 399.78219838[MHz]
Irr_Offset                   = 5[ppm]
Clipped                      = TRUE
Scans                        = 2400
Total_Scans                  = 2400

Relaxation_Delay             = 2[s]
Recvr_Gain                   = 50
Temp_Get                     = 25.3[dC]
X_90_Width                   = 10.3[us]
X_Acq_Time                   = 1.03809024[s]
X_Angle                      = 30[deg]
X_Atn                        = 7[dB]
X_Pulse                      = 3.43333333[us]
Irr_Atn_Dec                  = 26.116[dB]
Irr_Atn_Dec_Calc             = 26.116[dB]
Irr_Atn_Dec_Default_Calc     = 26.116[dB]
Irr_Atn_Noe                  = 26.116[dB]
Irr_Dec_Bandwidth_Hz         = 4.7826087[kHz]
Irr_Dec_Bandwidth_Ppm        = 11.96303566[ppm]
Irr_Dec_Freq                 = 399.78219838[MHz]
Irr_Dec_Merit_Factor         = 2.2
Irr_Decoupling               = TRUE
Irr_Noe                      = TRUE
Irr_Noise                    = WALTZ
Irr_Offset_Default           = 5[ppm]
Irr_Pwidth                   = 0.115[ms]
Irr_Pwidth_Default           = 0.115[ms]
Irr_Pwidth_Default_Calc      = 0.115[ms]
Irr_Pwidth_Temp1             = 0.115[ms]
Irr_Wurst                    = FALSE
Decimation_Rate              = 0
Initial_Wait                 = 1[s]
Noe_Time                     = 2[s]
Noe_Time_Flag                = FALSE
Relaxation_Delay_Calc        = 0[s]
Relaxation_Delay_Temp        = 2[s]
Repetition_Time              = 3.03809024[s]
```

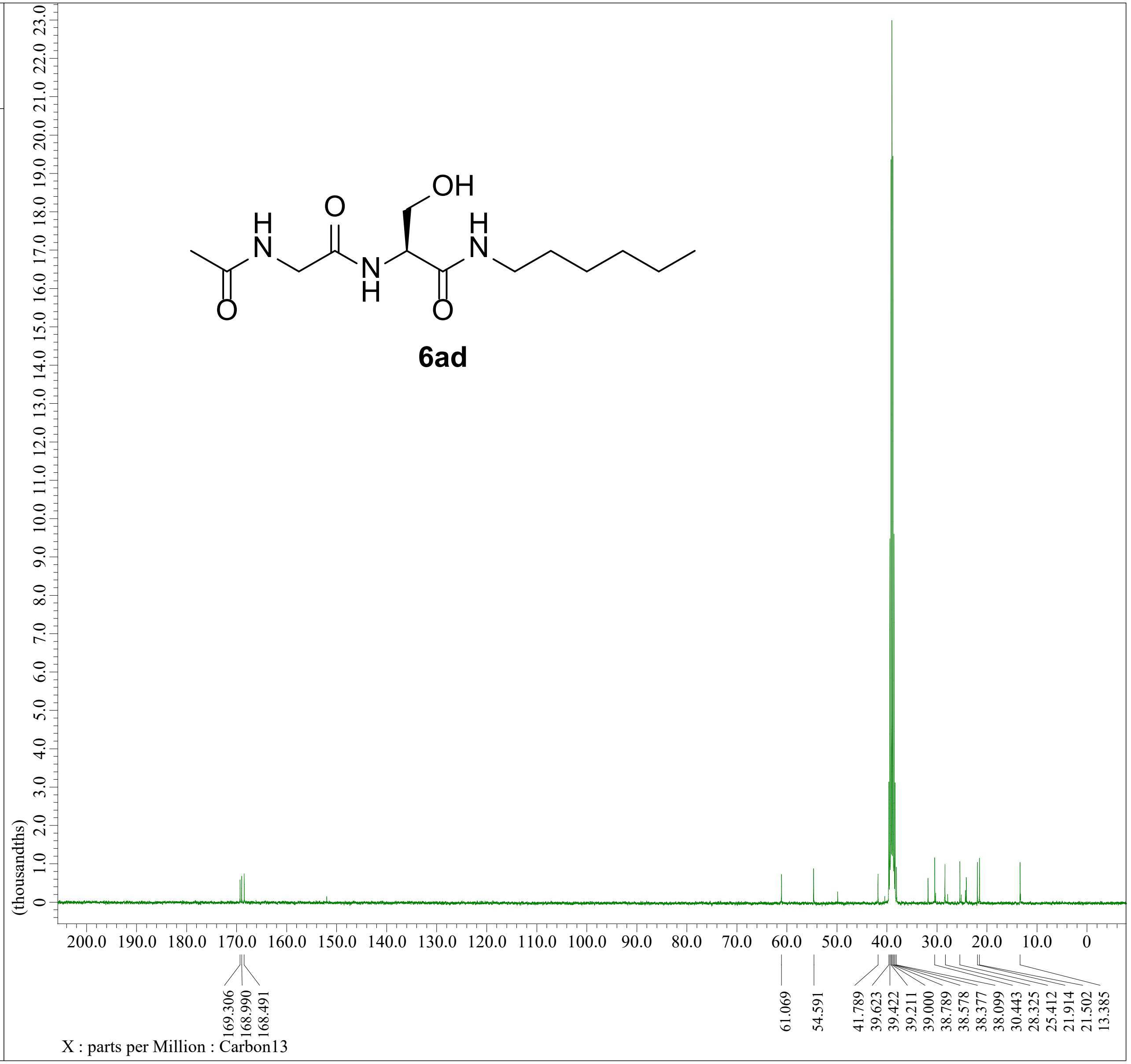

$^{1}$H NMR Spectrum of **6ae** (300 MHz, $CDCl_3$)

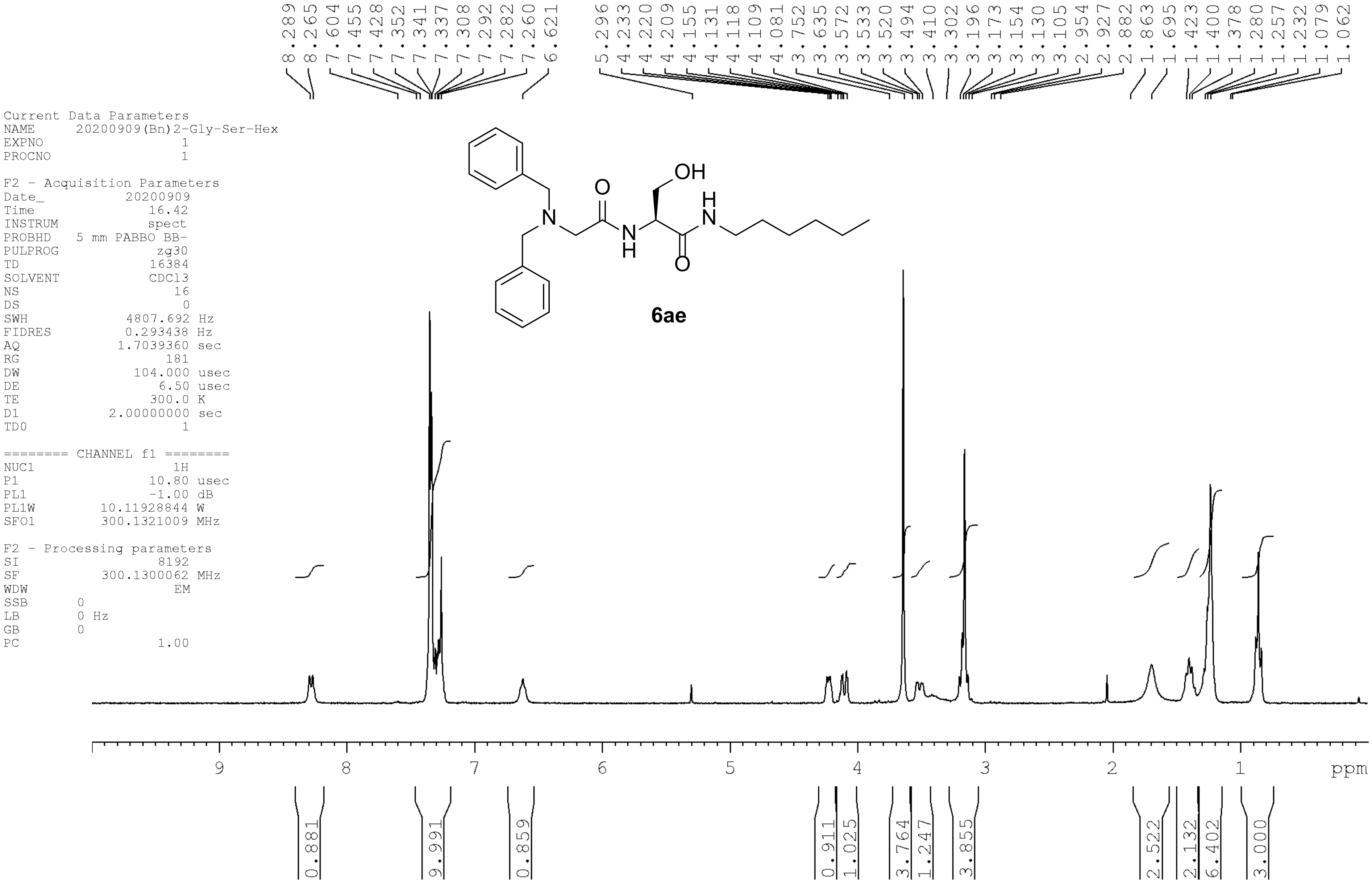

$^{13}C\{^1H\}$ NMR Spectrum of **6ae** (75 MHz, $CDCl_3$)

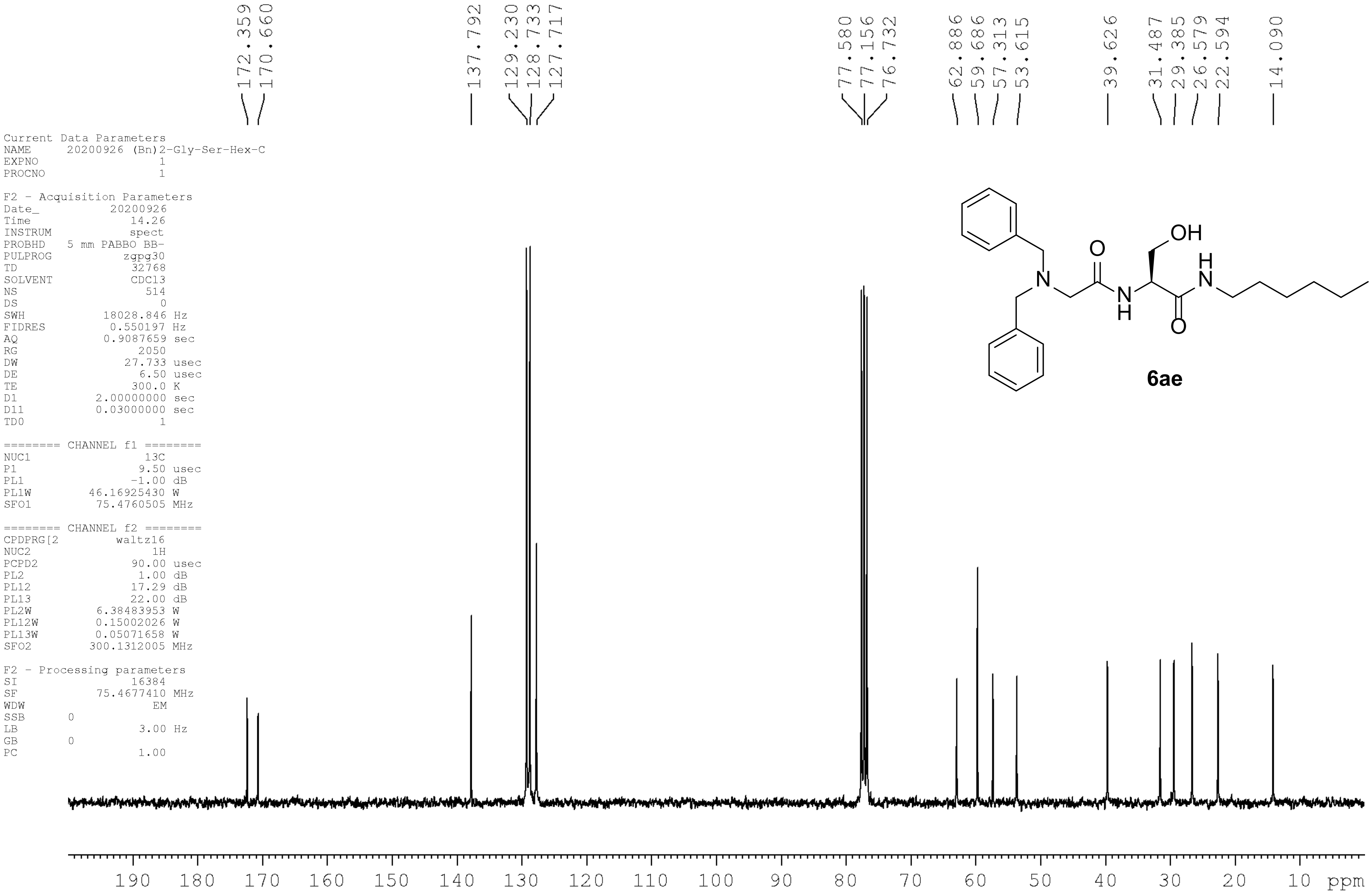


**6ae**

## $^{1}$H NMR Spectrum of **6b** (300 MHz, $CDCl_3$)

```
Current Data Parameters
NAME                H
EXPNO               1
PROCNO              1

F2 - Acquisition Parameters
Date_          20200217
Time              14.31
INSTRUM           spect
PROBHD   5 mm PABBO BB-
PULPROG            zg30
TD                16384
SOLVENT           CDCl3
NS                   16
DS                    0
SWH            4807.692 Hz
FIDRES         0.293438 Hz
AQ            1.7039360 sec
RG                  203
DW              104.000 usec
DE                 6.50 usec
TE                300.0 K
D1           2.00000000 sec
TD0                   1

======== CHANNEL f1 ========
NUC1                 1H
P1                10.80 usec
PL1               -1.00 dB
PL1W        10.11928844 W
SFO1        300.1321009 MHz

F2 - Processing parameters
SI                 8192
SF          300.1300062 MHz
WDW                  EM
SSB      0
LB       0 Hz
GB       0
PC                 1.00
```

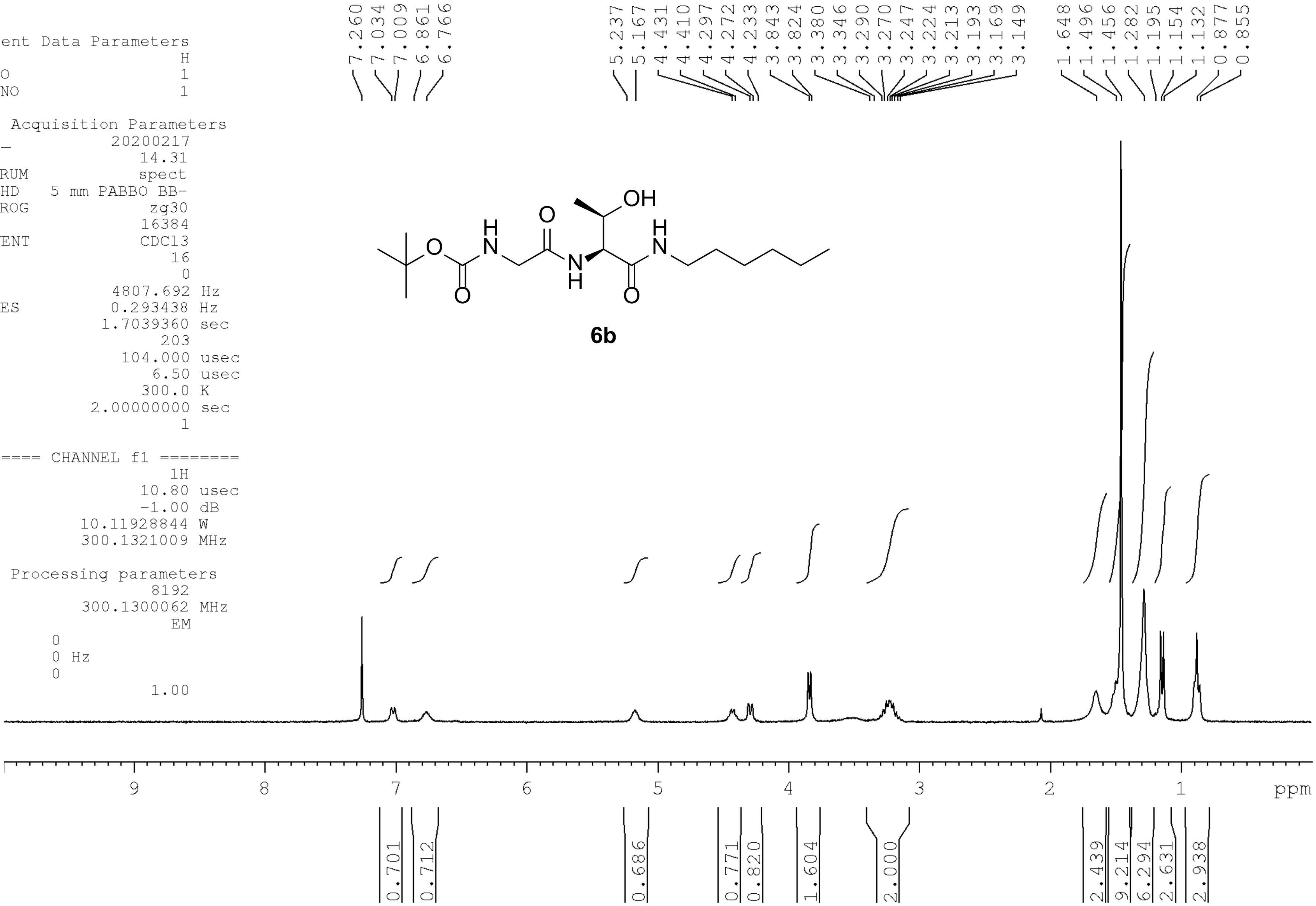

## $^{13}C\{^{1}H\}$ NMR Spectrum of **6b** (100 MHz, $CDCl_3$)

| | |
|---|---|
| Filename | = BOC-GLY-THR-HEX 20 |
| Author | = YYL |
| Experiment | = carbon.jxp |
| Sample_Id | = BOC-GLY-THR-HEX 20 |
| Solvent | = CHLOROFORM-D |
| Actual_Start_Time | = 19-FEB-2020 11:07: |
| Revision_Time | = 22-FEB-2020 16:09: |
| Comment | = single pulse decou |
| Data_Format | = 1D COMPLEX |
| Dim_Size | = 26214 |
| X_Domain | = Carbon13 |
| Dim_Title | = Carbon13 |
| Dim_Units | = [ppm] |
| Dimensions | = X |
| Spectrometer | = JNM-ECZ400S/L1 |
| Field_Strength | = 9.389766[T] (400[M |
| X_Acq_Duration | = 1.03809024[s] |
| X_Domain | = 13C |
| X_Freq | = 100.52530333[MHz] |
| X_Offset | = 100[ppm] |
| X_Points | = 32768 |
| X_Prescans | = 4 |
| X_Resolution | = 0.96330739[Hz] |
| X_Sweep | = 31.56565657[kHz] |
| X_Sweep_Clipped | = 25.25252525[kHz] |
| Irr_Domain | = Proton |
| Irr_Freq | = 399.78219838[MHz] |
| Irr_Offset | = 5[ppm] |
| Clipped | = FALSE |
| Scans | = 1024 |
| Total_Scans | = 1024 |
| Relaxation_Delay | = 2[s] |
| Recvr_Gain | = 50 |
| Temp_Get | = 22.1[dC] |
| X_90_Width | = 10.369[us] |
| X_Acq_Time | = 1.03809024[s] |
| X_Angle | = 30[deg] |
| X_Atn | = 7[dB] |
| X_Pulse | = 3.45633333[us] |
| Irr_Atn_Dec | = 25.678[dB] |
| Irr_Atn_Dec_Calc | = 25.678[dB] |
| Irr_Atn_Dec_Default_Calc | = 25.678[dB] |
| Irr_Atn_Noe | = 25.678[dB] |
| Irr_Dec_Bandwidth_Hz | = 4.7826087[kHz] |
| Irr_Dec_Bandwidth_Ppm | = 11.96303566[ppm] |
| Irr_Dec_Freq | = 399.78219838[MHz] |
| Irr_Dec_Merit_Factor | = 2.2 |
| Irr_Decoupling | = TRUE |
| Irr_Noe | = TRUE |
| Irr_Noise | = WALTZ |
| Irr_Offset_Default | = 5[ppm] |
| Irr_Pwidth | = 0.115[ms] |
| Irr_Pwidth_Default | = 0.115[ms] |
| Irr_Pwidth_Default_Calc | = 0.115[ms] |
| Irr_Pwidth_Templ | = 0.115[ms] |
| Irr_Wurst | = FALSE |
| Decimation_Rate | = 0 |
| Initial_Wait | = 1[s] |
| Noe_Time | = 2[s] |
| Noe_Time_Flag | = FALSE |
| Relaxation_Delay_Calc | = 0[s] |
| Relaxation_Delay_Temp | = 2[s] |
| Repetition_Time | = 3.03809024[s] |

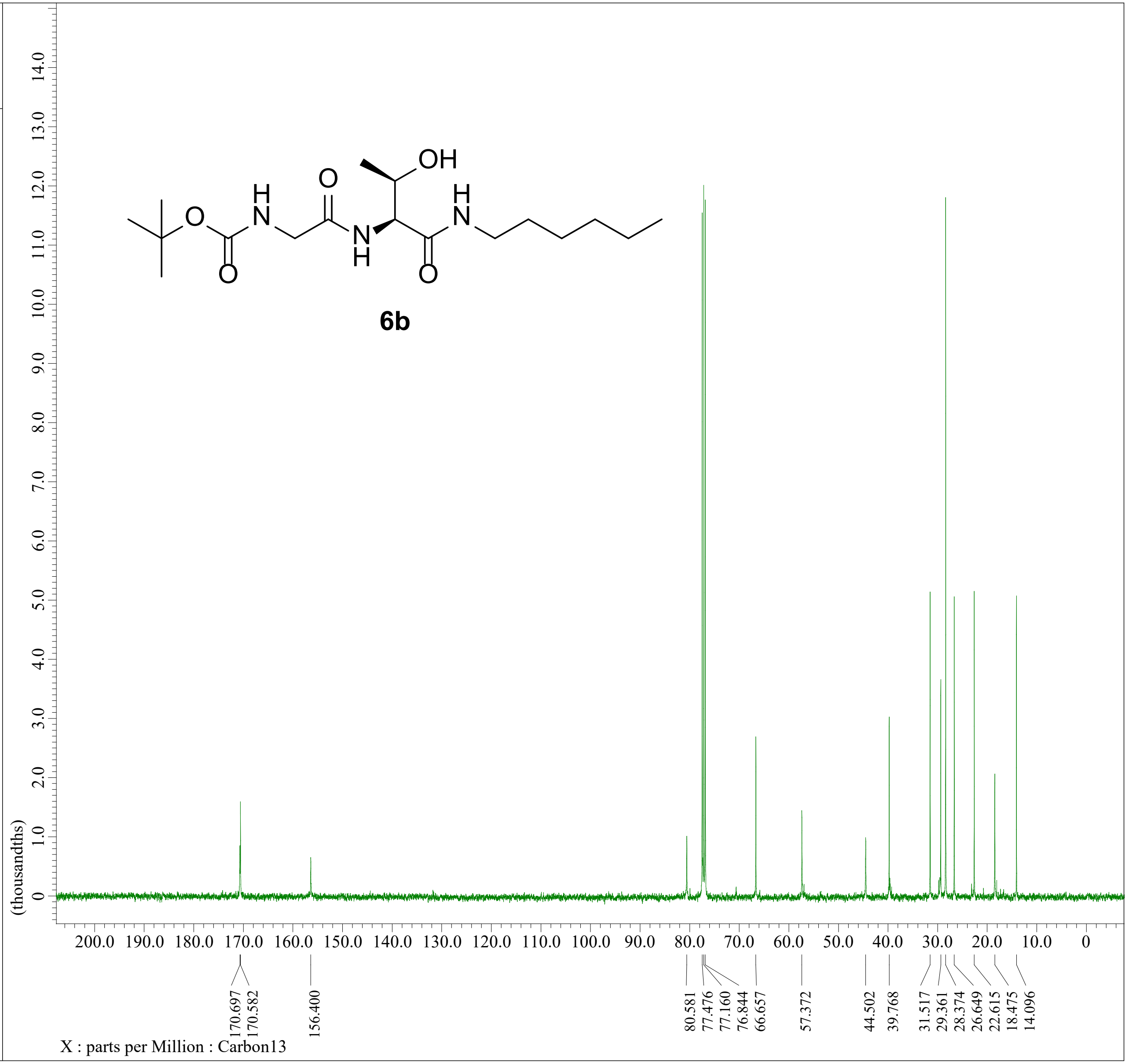

$^{1}$H NMR Spectrum of **6c** (300 MHz, $CDCl_3$)

```
Current Data Parameters
NAME     20200515 ala-ser
EXPNO                   1
PROCNO                  1

F2 - Acquisition Parameters
Date_           20200515
Time               16.25
INSTRUM            spect
PROBHD   5 mm PABBO BB-
PULPROG             zg30
TD                 16384
SOLVENT            CDCl3
NS                    14
DS                     0
SWH             4807.692 Hz
FIDRES          0.293438 Hz
AQ             1.7039360 sec
RG                   161
DW               104.000 usec
DE                  6.50 usec
TE                 300.0 K
D1            2.00000000 sec
TD0                    1

======== CHANNEL f1 ========
NUC1                  1H
P1                 10.80 usec
PL1                -1.00 dB
PL1W         10.11928844 W
SFO1         300.1321009 MHz

F2 - Processing parameters
SI                  8192
SF           300.1300062 MHz
WDW                   EM
SSB                    0
LB                     0 Hz
GB                     0
PC                  1.00
```

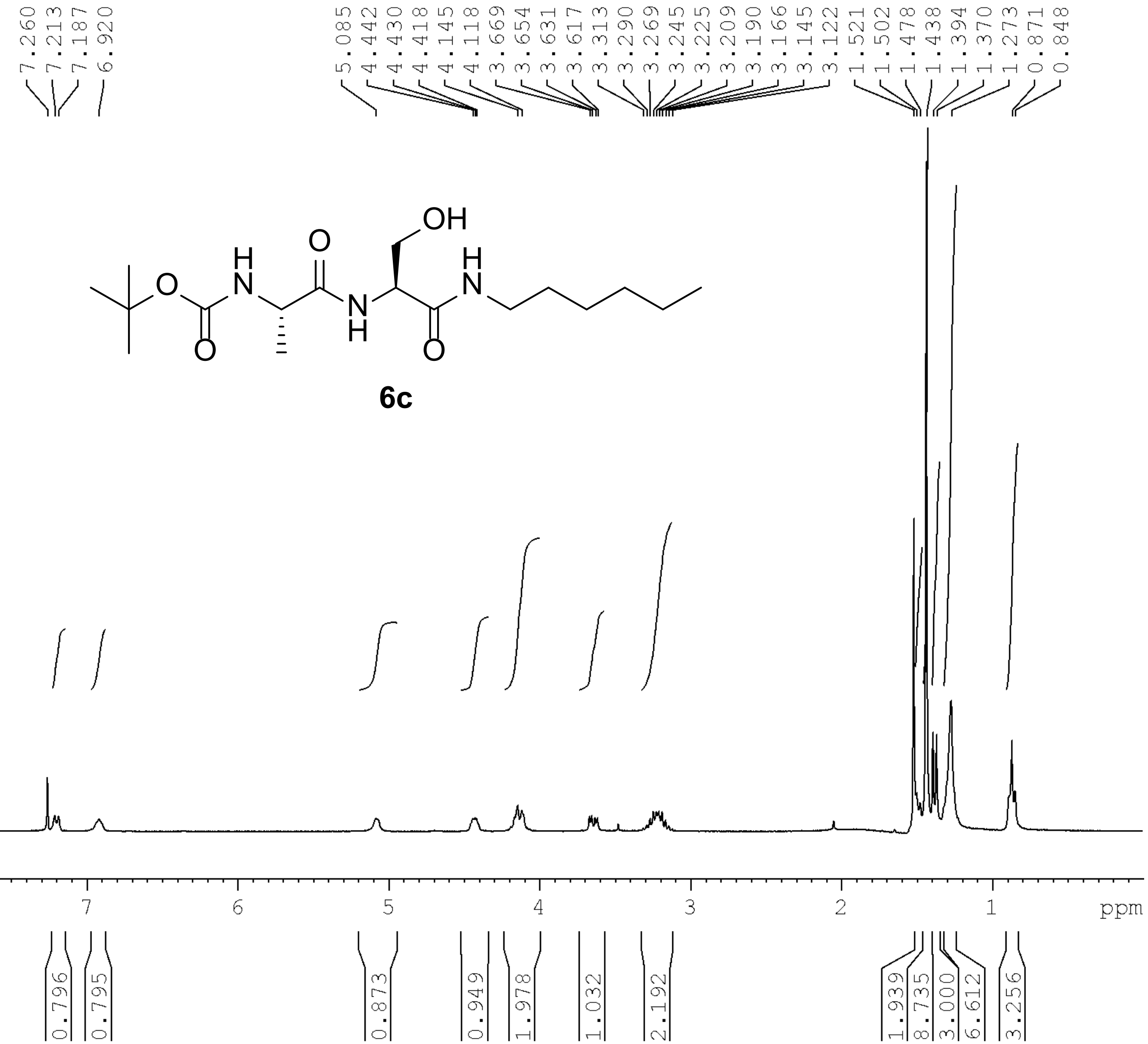

## $^{13}C\{^1H\}$ NMR Spectrum of **6c** (100 MHz, $CDCl_3$)

```
Filename                      = boc-ala-ser-hex.jd
Author                        = yyl
Experiment                    = carbon.jxp
Sample_Id                     = 20190618 val+ser c
Solvent                       = CHLOROFORM-D
Actual_Start_Time             = 18-JUN-2019 23:33:
Revision_Time                 = 22-JUL-2019 11:12:

Comment                       = single pulse decou
Data_Format                   = 1D COMPLEX
Dim_Size                      = 26214
X_Domain                      = Carbon13
Dim_Title                     = Carbon13
Dim_Units                     = [ppm]
Dimensions                    = X
Spectrometer                  = JNM-ECZ400S/L1

Field_Strength                = 9.389766[T] (400[M
X_Acq_Duration                = 1.03809024[s]
X_Domain                      = 13C
X_Freq                        = 100.52530333[MHz]
X_Offset                      = 100[ppm]
X_Points                      = 32768
X_Prescans                    = 4
X_Resolution                  = 0.96330739[Hz]
X_Sweep                       = 31.56565657[kHz]
X_Sweep_Clipped               = 25.25252525[kHz]
Irr_Domain                    = Proton
Irr_Freq                      = 399.78219838[MHz]
Irr_Offset                    = 5[ppm]
Clipped                       = FALSE
Scans                         = 1024
Total_Scans                   = 1024

Relaxation_Delay              = 2[s]
Recvr_Gain                    = 50
Temp_Get                      = 21.6[dC]
X_90_Width                    = 10.27[us]
X_Acq_Time                    = 1.03809024[s]
X_Angle                       = 30[deg]
X_Atn                         = 7[dB]
X_Pulse                       = 3.42333333[us]
Irr_Atn_Dec                   = 24.372[dB]
Irr_Atn_Dec_Calc              = 24.372[dB]
Irr_Atn_Dec_Default_Calc      = 24.372[dB]
Irr_Atn_Noe                   = 24.372[dB]
Irr_Dec_Bandwidth_Hz          = 4.7826087[kHz]
Irr_Dec_Bandwidth_Ppm         = 11.96303566[ppm]
Irr_Dec_Freq                  = 399.78219838[MHz]
Irr_Dec_Merit_Factor          = 2.2
Irr_Decoupling                = TRUE
Irr_Noe                       = TRUE
Irr_Noise                     = WALTZ
Irr_Offset_Default            = 5[ppm]
Irr_Pwidth                    = 0.115[ms]
Irr_Pwidth_Default            = 0.115[ms]
Irr_Pwidth_Default_Calc       = 0.115[ms]
Irr_Pwidth_Templ              = 0.115[ms]
Irr_Wurst                     = FALSE
Decimation_Rate               = 0
Initial_Wait                  = 1[s]
Noe_Time                      = 2[s]
Noe_Time_Flag                 = FALSE
Relaxation_Delay_Calc         = 0[s]
Relaxation_Delay_Temp         = 2[s]
Repetition_Time               = 3.03809024[s]
```

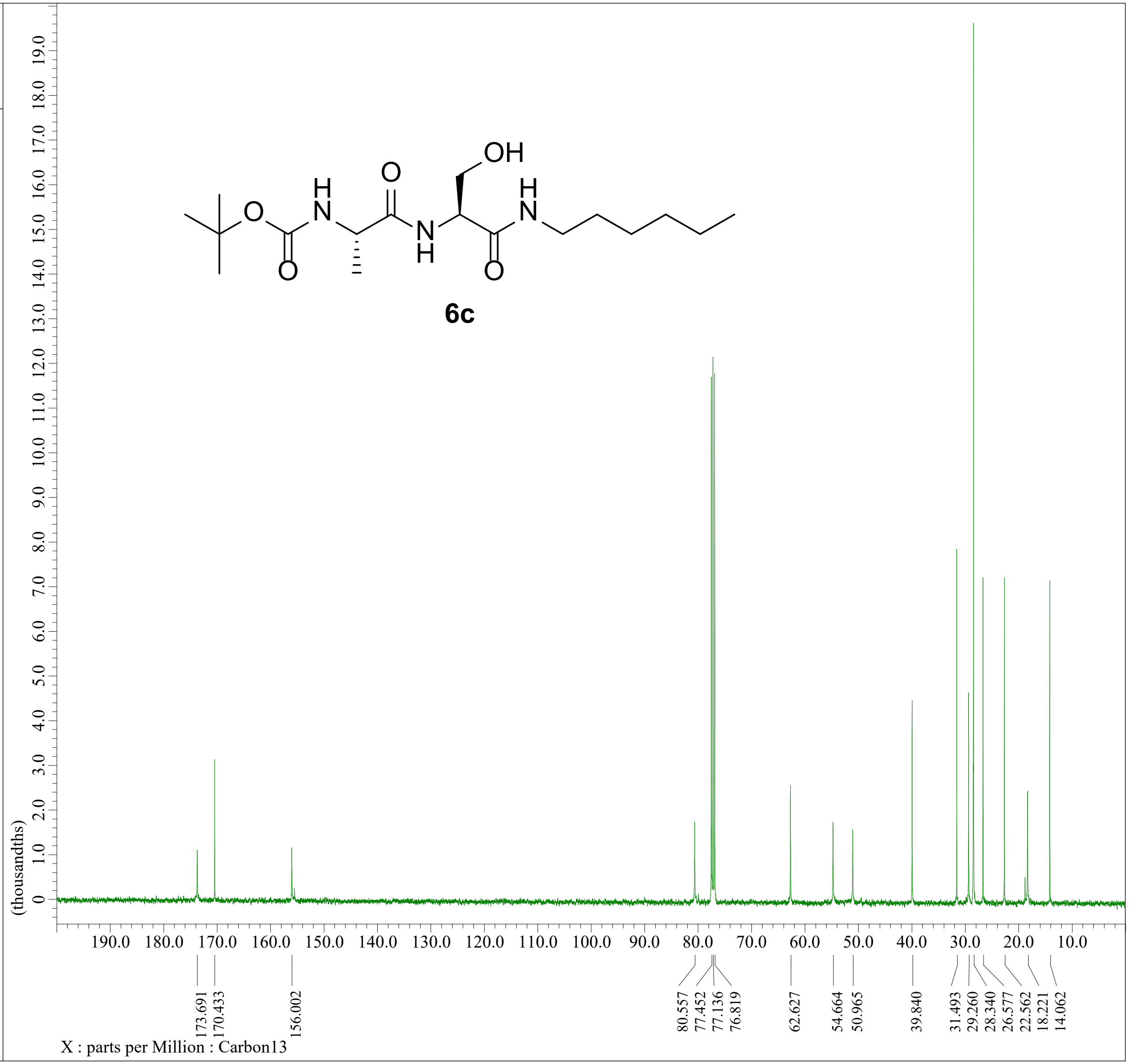

$^{1}$H NMR Spectrum of **6d** (300 MHz, $CDCl_3$)

```
Current Data Parameters
NAME      20191007 9d clean
EXPNO                   1
PROCNO                  1

F2 - Acquisition Parameters
Date_          20191007
Time              17.18
INSTRUM           spect
PROBHD   5 mm PABBO BB-
PULPROG            zg30
TD                16384
SOLVENT           CDCl3
NS                   14
DS                    0
SWH            4807.692 Hz
FIDRES         0.293438 Hz
AQ            1.7039360 sec
RG                  228
DW              104.000 usec
DE                 6.50 usec
TE                300.0 K
D1           2.00000000 sec
TD0                   1

======== CHANNEL f1 ========
NUC1                 1H
P1                10.80 usec
PL1               -1.00 dB
PL1W        10.11928844 W
SFO1        300.1321009 MHz

F2 - Processing parameters
SI                 8192
SF          300.1300065 MHz
WDW                  EM
SSB                   0
LB                    0 Hz
GB                    0
PC                 1.00
```

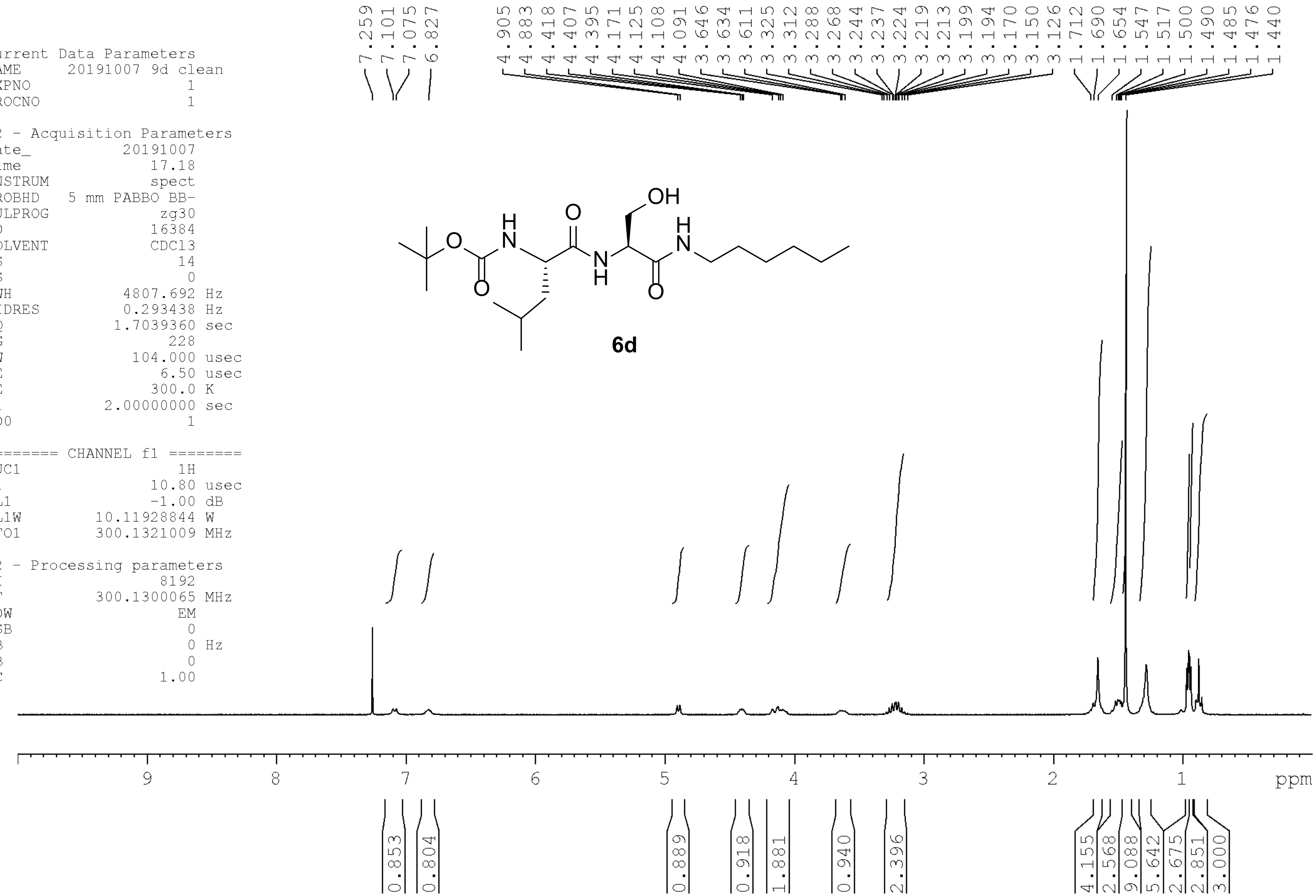

## $^{13}C\{^1H\}$ NMR Spectrum of **6d** (100 MHz, $CDCl_3$)

| | |
|---|---|
| Filename | = boc-leu-ser-hex.jd |
| Author | = YYL |
| Experiment | = carbon.jxp |
| Sample_Id | = 20190411 leu+ser |
| Solvent | = CHLOROFORM-D |
| Actual_Start_Time | = 11-APR-2019 23:33: |
| Revision_Time | = 22-JUL-2019 12:27: |
| Comment | = single pulse decou |
| Data_Format | = 1D COMPLEX |
| Dim_Size | = 26214 |
| X_Domain | = Carbon13 |
| Dim_Title | = Carbon13 |
| Dim_Units | = [ppm] |
| Dimensions | = X |
| Spectrometer | = JNM-ECZ400S/L1 |
| Field_Strength | = 9.389766[T] (400[M |
| X_Acq_Duration | = 1.03809024[s] |
| X_Domain | = 13C |
| X_Freq | = 100.52530333[MHz] |
| X_Offset | = 100[ppm] |
| X_Points | = 32768 |
| X_Prescans | = 4 |
| X_Resolution | = 0.96330739[Hz] |
| X_Sweep | = 31.56565657[kHz] |
| X_Sweep_Clipped | = 25.25252525[kHz] |
| Irr_Domain | = Proton |
| Irr_Freq | = 399.78219838[MHz] |
| Irr_Offset | = 5[ppm] |
| Clipped | = FALSE |
| Scans | = 2048 |
| Total_Scans | = 2048 |
| Relaxation_Delay | = 2[s] |
| Recvr_Gain | = 50 |
| Temp_Get | = 21.6[dC] |
| X_90_Width | = 10.6[us] |
| X_Acq_Time | = 1.03809024[s] |
| X_Angle | = 30[deg] |
| X_Atn | = 7[dB] |
| X_Pulse | = 3.53333333[us] |
| Irr_Atn_Dec | = 26.437[dB] |
| Irr_Atn_Dec_Calc | = 26.437[dB] |
| Irr_Atn_Dec_Default_Calc | = 26.437[dB] |
| Irr_Atn_Noe | = 26.437[dB] |
| Irr_Dec_Bandwidth_Hz | = 4.7826087[kHz] |
| Irr_Dec_Bandwidth_Ppm | = 11.96303566[ppm] |
| Irr_Dec_Freq | = 399.78219838[MHz] |
| Irr_Dec_Merit_Factor | = 2.2 |
| Irr_Decoupling | = TRUE |
| Irr_Noe | = TRUE |
| Irr_Noise | = WALTZ |
| Irr_Offset_Default | = 5[ppm] |
| Irr_Pwidth | = 0.115[ms] |
| Irr_Pwidth_Default | = 0.115[ms] |
| Irr_Pwidth_Default_Calc | = 0.115[ms] |
| Irr_Pwidth_Temp1 | = 0.115[ms] |
| Irr_Wurst | = FALSE |
| Decimation_Rate | = 0 |
| Initial_Wait | = 1[s] |
| Noe_Time | = 2[s] |
| Noe_Time_Flag | = FALSE |
| Relaxation_Delay_Calc | = 0[s] |
| Relaxation_Delay_Temp | = 2[s] |
| Repetition_Time | = 3.03809024[s] |

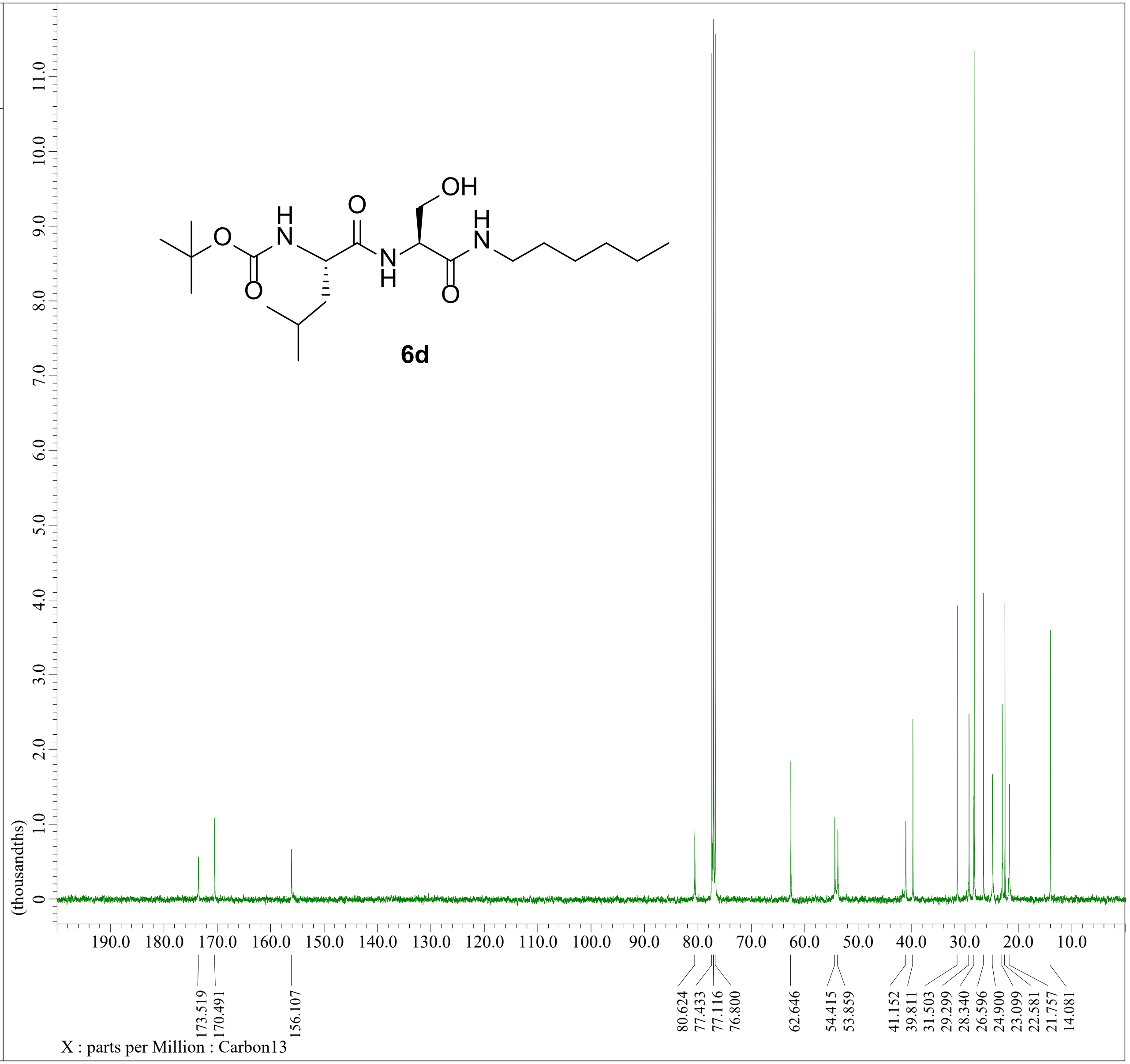

## $^{1}$H NMR Spectrum of **6e** (300 MHz, $CDCl_3$)

Current Data Parameters
NAME 20201029 boc-val-ser-hex
EXPNO 1
PROCNO 1

F2 - Acquisition Parameters
Date_ 20201029
Time 11.21
INSTRUM spect
PROBHD 5 mm PABBO BB-
PULPROG zg30
TD 16384
SOLVENT CDCl3
NS 16
DS 0
SWH 4807.692 Hz
FIDRES 0.293438 Hz
AQ 1.7039360 sec
RG 181
DW 104.000 usec
DE 6.50 usec
TE 300.0 K
D1 2.00000000 sec
TD0 1

======== CHANNEL f1 ========
NUC1 1H
P1 10.80 usec
PL1 -1.00 dB
PL1W 10.11928844 W
SFO1 300.1321009 MHz

F2 - Processing parameters
SI 8192
SF 300.1300065 MHz
WDW EM
SSB 0
LB 0 Hz
GB 0
PC 1.00

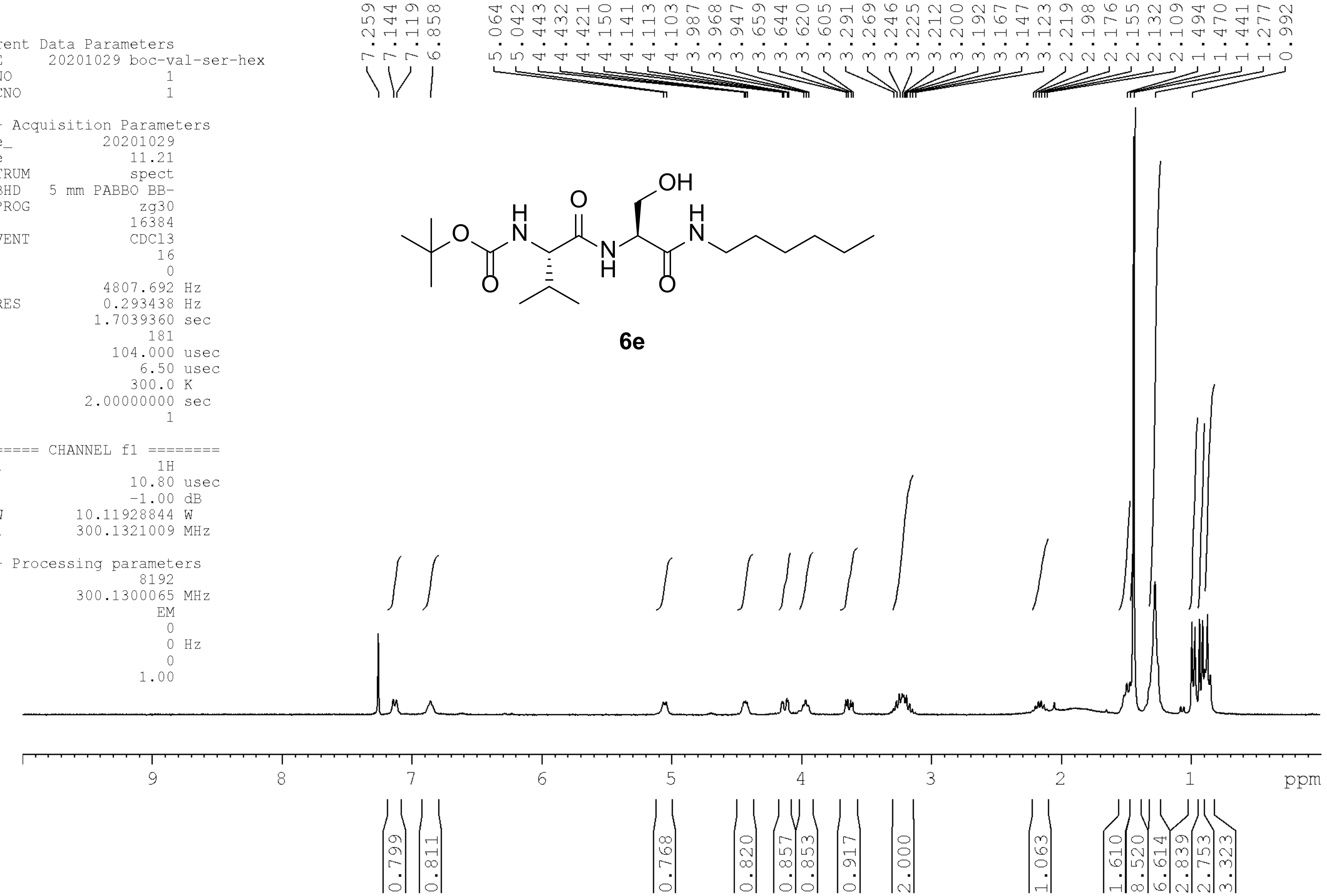

## $^{13}C\{^{1}H\}$ NMR Spectrum of **6e** (100 MHz, $CDCl_3$)

```
Filename                    = boc-val-ser-hex.jd
Author                      = yyl
Experiment                  = carbon.jxp
Sample_Id                   = 20190618 ala+ser 1
Solvent                     = CHLOROFORM-D
Actual_Start_Time           = 19-JUN-2019 00:34:
Revision_Time               = 19-JUN-2019 15:08:

Comment                     = single pulse decou
Data_Format                 = 1D COMPLEX
Dim_Size                    = 26214
X_Domain                    = Carbon13
Dim_Title                   = Carbon13
Dim_Units                   = [ppm]
Dimensions                  = X
Spectrometer                = JNM-ECZ400S/L1

Field_Strength              = 9.389766[T] (400[M
X_Acq_Duration              = 1.03809024[s]
X_Domain                    = 13C
X_Freq                      = 100.52530333[MHz]
X_Offset                    = 100[ppm]
X_Points                    = 32768
X_Prescans                  = 4
X_Resolution                = 0.96330739[Hz]
X_Sweep                     = 31.56565657[kHz]
X_Sweep_Clipped             = 25.25252525[kHz]
Irr_Domain                  = Proton
Irr_Freq                    = 399.78219838[MHz]
Irr_Offset                  = 5[ppm]
Clipped                     = FALSE
Scans                       = 4096
Total_Scans                 = 4096

Relaxation_Delay            = 2[s]
Recvr_Gain                  = 50
Temp_Get                    = 21.7[dC]
X_90_Width                  = 10.27[us]
X_Acq_Time                  = 1.03809024[s]
X_Angle                     = 30[deg]
X_Atn                       = 7[dB]
X_Pulse                     = 3.42333333[us]
Irr_Atn_Dec                 = 24.372[dB]
Irr_Atn_Dec_Calc            = 24.372[dB]
Irr_Atn_Dec_Default_Calc    = 24.372[dB]
Irr_Atn_Noe                 = 24.372[dB]
Irr_Dec_Bandwidth_Hz        = 4.7826087[kHz]
Irr_Dec_Bandwidth_Ppm       = 11.96303566[ppm]
Irr_Dec_Freq                = 399.78219838[MHz]
Irr_Dec_Merit_Factor        = 2.2
Irr_Decoupling              = TRUE
Irr_Noe                     = TRUE
Irr_Noise                   = WALTZ
Irr_Offset_Default          = 5[ppm]
Irr_Pwidth                  = 0.115[ms]
Irr_Pwidth_Default          = 0.115[ms]
Irr_Pwidth_Default_Calc     = 0.115[ms]
Irr_Pwidth_Templ            = 0.115[ms]
Irr_Wurst                   = FALSE
Decimation_Rate             = 0
Initial_Wait                = 1[s]
Noe_Time                    = 2[s]
Noe_Time_Flag               = FALSE
Relaxation_Delay_Calc       = 0[s]
Relaxation_Delay_Temp       = 2[s]
Repetition_Time             = 3.03809024[s]
```

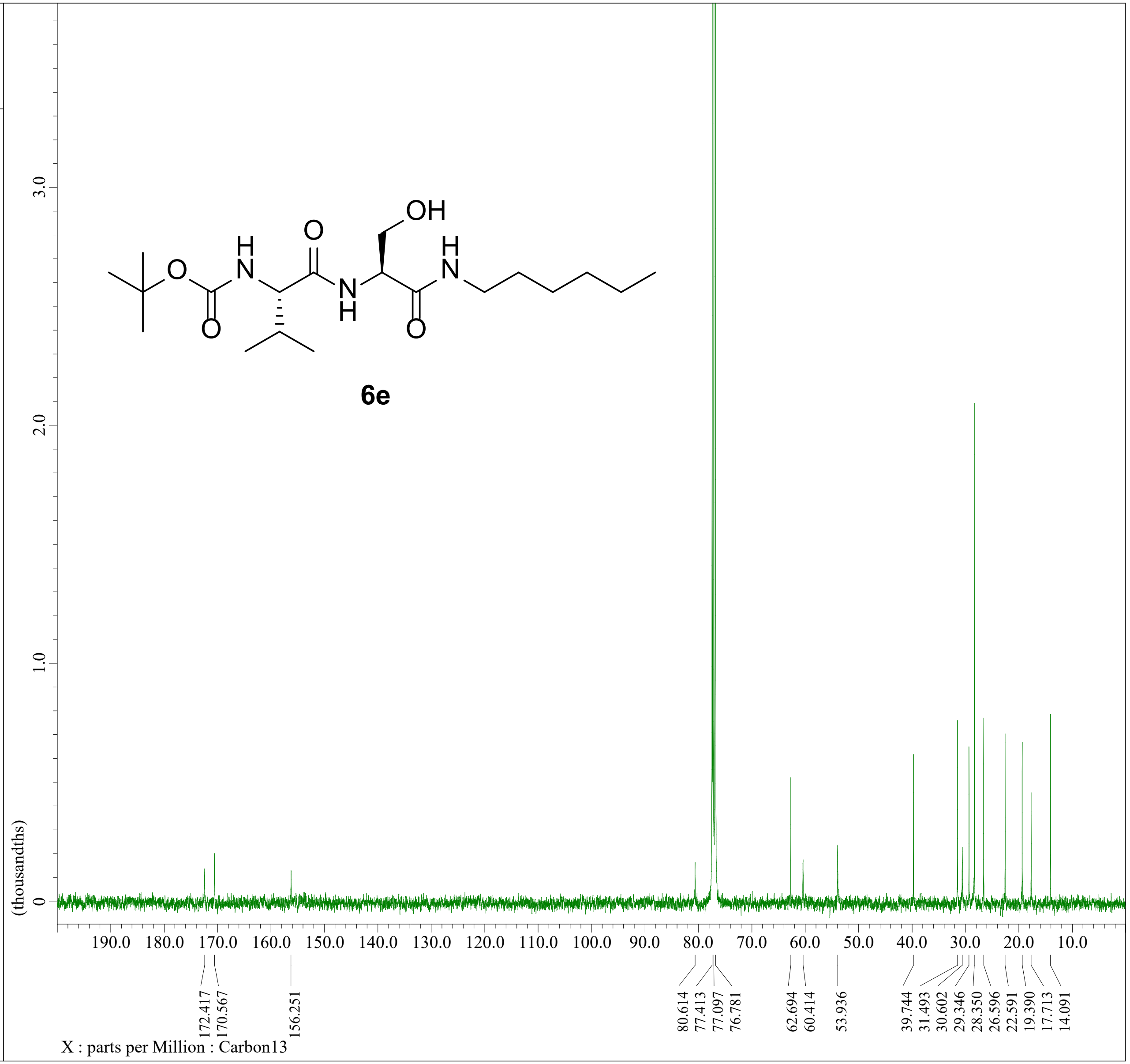

$^{1}$H NMR Spectrum of **6f** (300 MHz, $CDCl_3$)

```
Current Data Parameters
NAME      20191003 iso 1+1
EXPNO                    1
PROCNO                   1

F2 - Acquisition Parameters
Date_           20191003
Time               17.26
INSTRUM            spect
PROBHD   5 mm PABBO BB-
PULPROG             zg30
TD                 16384
SOLVENT            CDCl3
NS                    13
DS                     0
SWH             4807.692 Hz
FIDRES          0.293438 Hz
AQ             1.7039360 sec
RG                   128
DW               104.000 usec
DE                  6.50 usec
TE                 300.0 K
D1            2.00000000 sec
TD0                    1

======== CHANNEL f1 ========
NUC1                  1H
P1                 10.80 usec
PL1                -1.00 dB
PL1W         10.11928844 W
SFO1        300.1321009 MHz

F2 - Processing parameters
SI                  8192
SF          300.1300071 MHz
WDW                   EM
SSB                    0
LB                     0 Hz
GB                     0
PC                  1.00
```

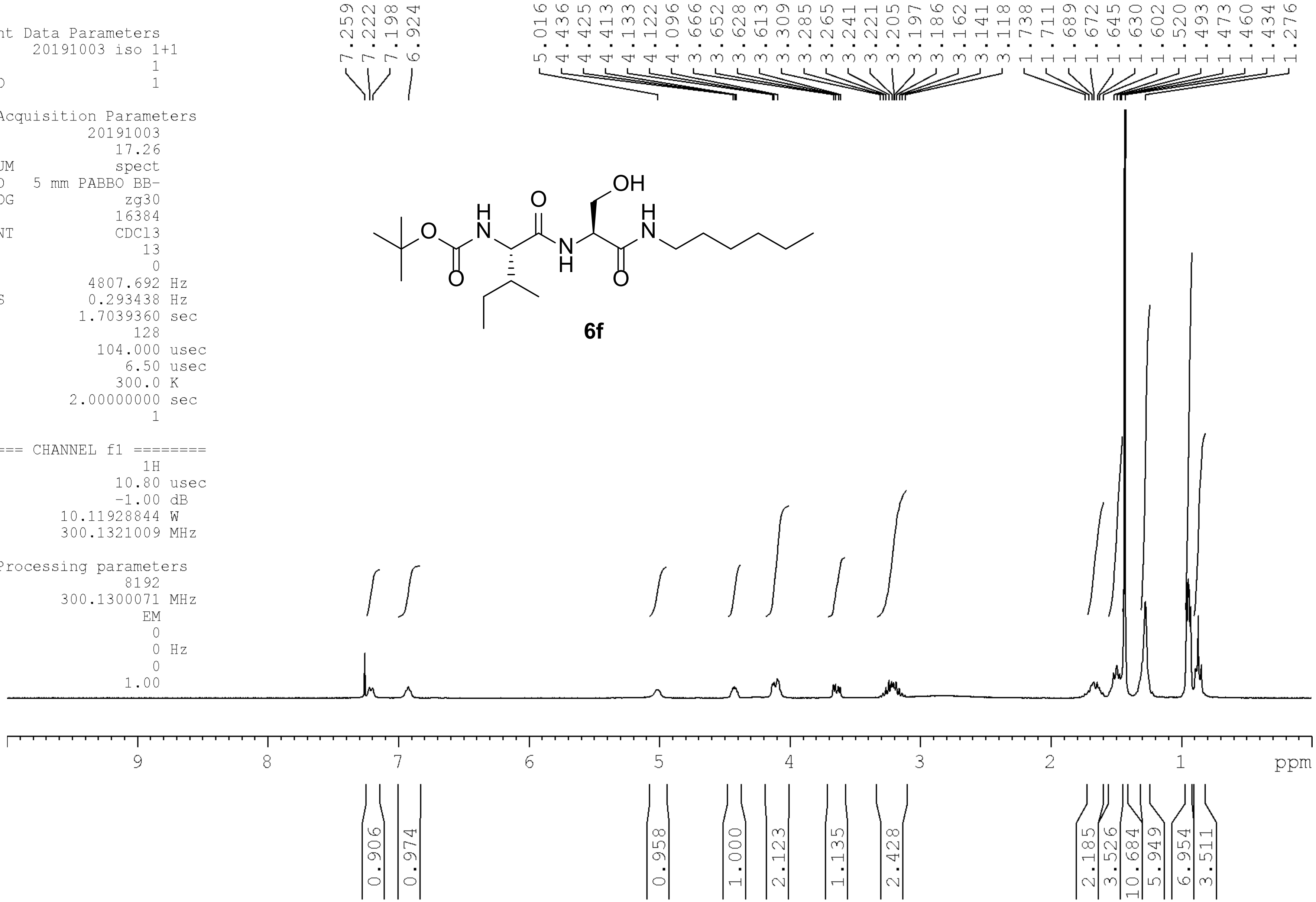

$^{13}C\{^{1}H\}$ NMR Spectrum of **6f** (100 MHz, $CDCl_3$)

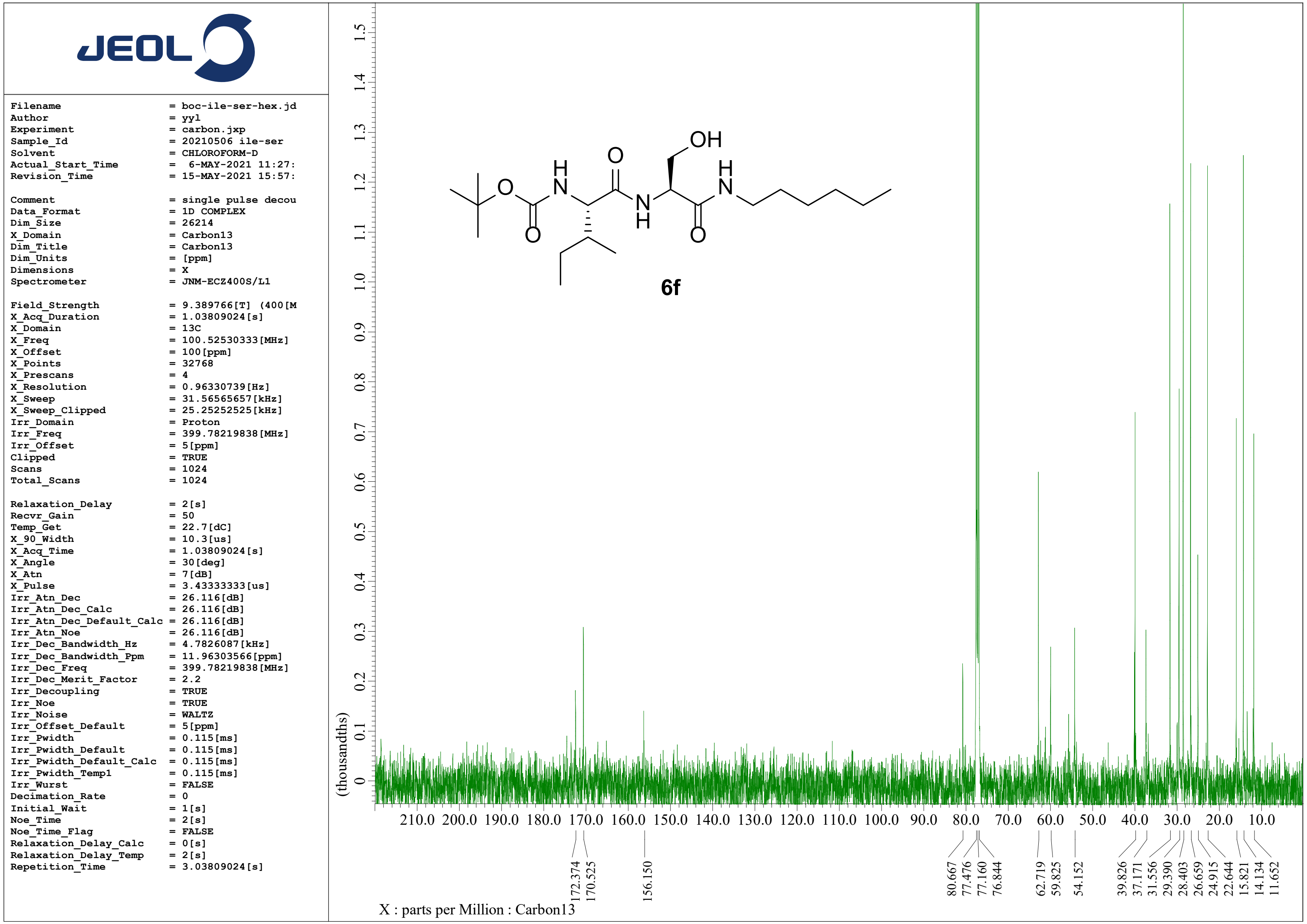

$^1$H NMR Spectrum of **6g** (400 MHz, $CDCl_3$)

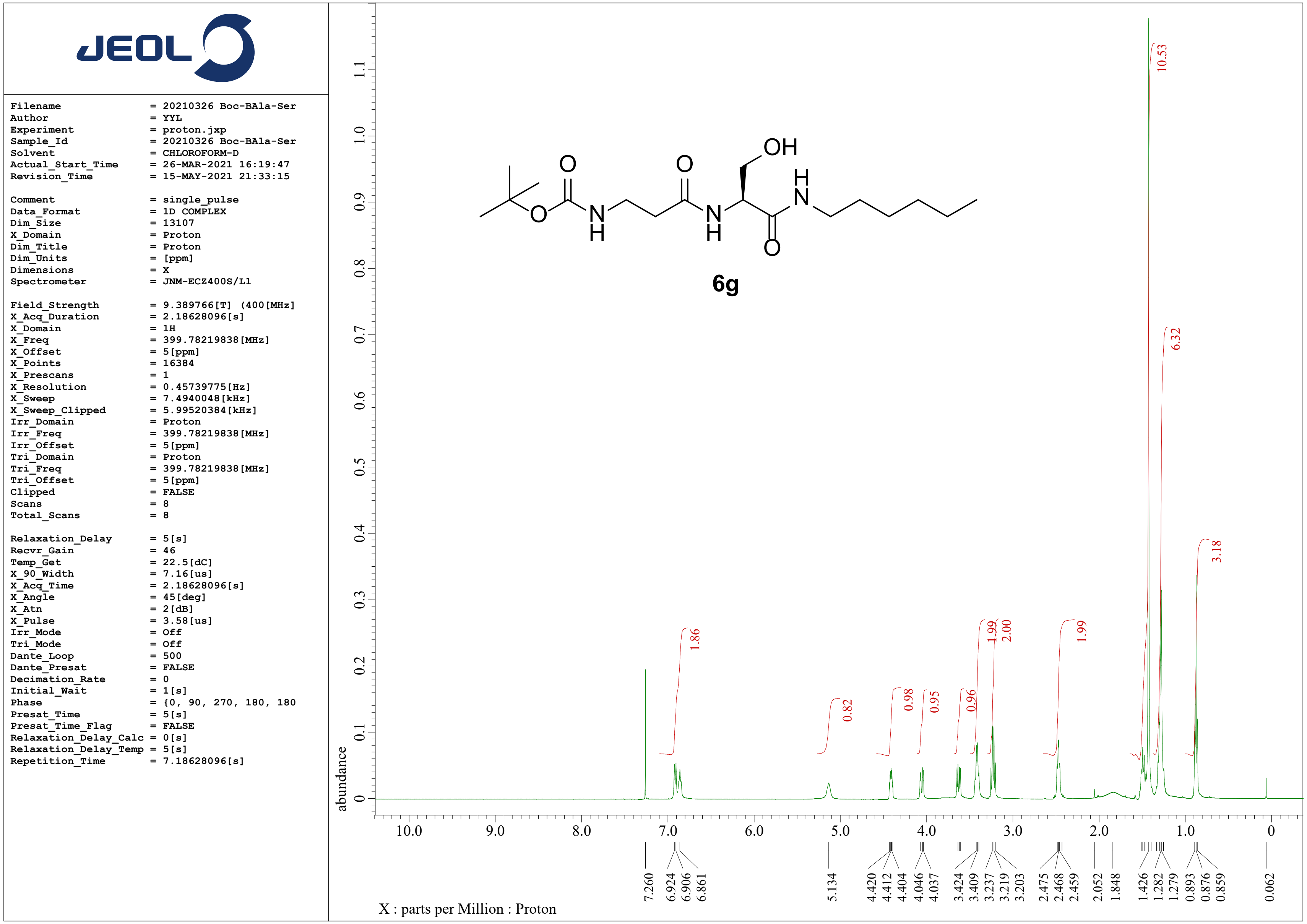

## $^{13}C\{^1H\}$ NMR Spectrum of **6g** (75 MHz, $CDCl_3$)

```
Current Data Parameters
NAME     20210329 Boc-BAla-Ser-Hex-C
EXPNO                  1
PROCNO                 1

F2 - Acquisition Parameters
Date_          20210329
Time              11.25 h
INSTRUM           spect
PROBHD   Z104275_0120 (
PULPROG          zgpg30
TD                32768
SOLVENT           CDCl3
NS                  440
DS                    0
SWH           18028.846 Hz
FIDRES         1.100393 Hz
AQ            0.9087659 sec
RG                 2050
DW               27.733 usec
DE                 6.50 usec
TE                300.0 K
D1           2.00000000 sec
D11          0.03000000 sec
TD0                   1
SFO1         75.4760505 MHz
NUC1                13C
P1                10.00 usec
PLW1        44.00000000 W
SFO2        300.1312005 MHz
NUC2                 1H
CPDPRG[2        waltz16
PCPD2             90.00 usec
PLW2         5.69999981 W
PLW12        0.15832999 W
PLW13        0.07964100 W

F2 - Processing parameters
SI                16384
SF           75.4677394 MHz
WDW                  EM
SSB      0
LB                 3.00 Hz
GB       0
PC                 1.00
```

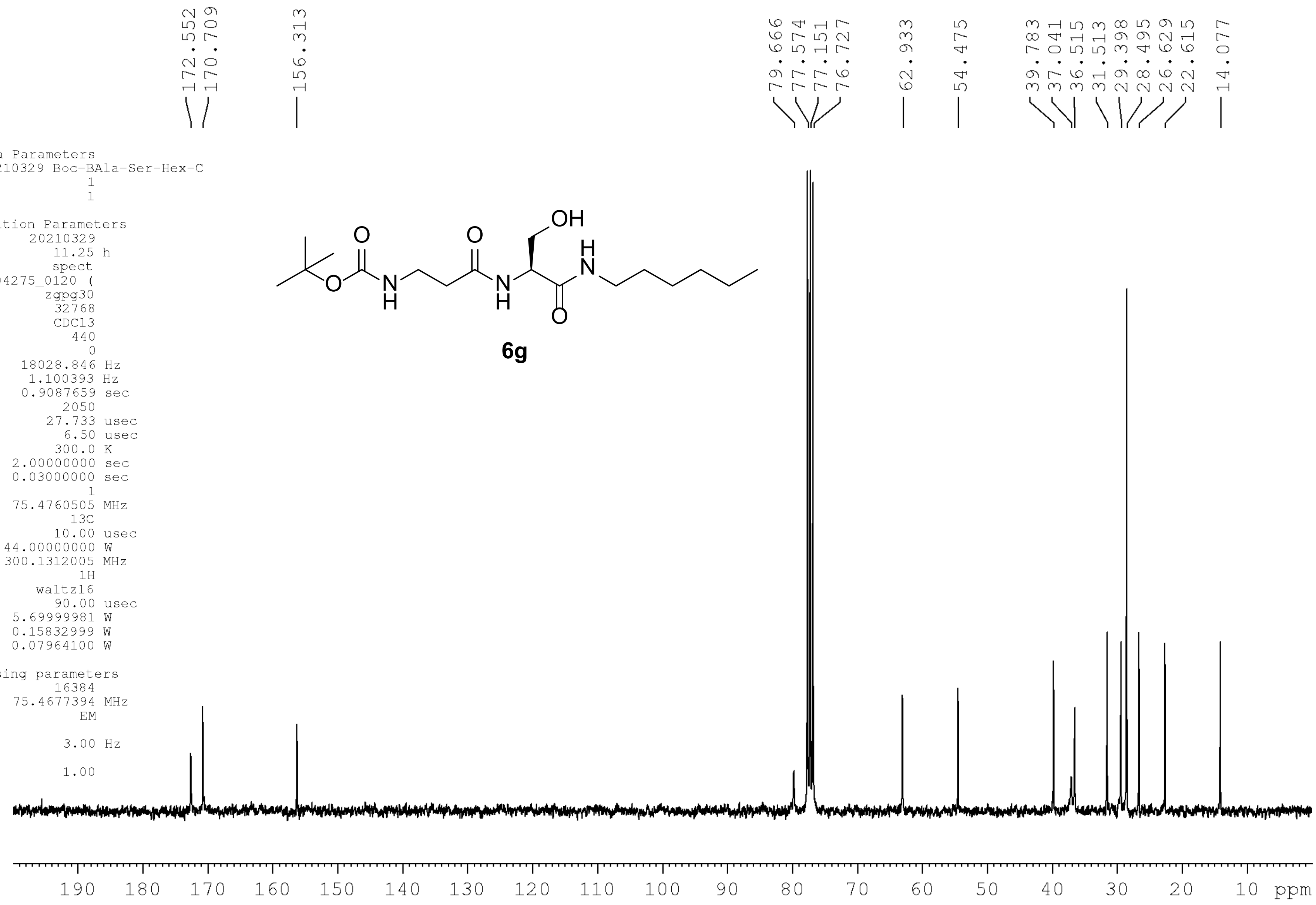

$^{1}$H NMR Spectrum of **6h** (300 MHz, $CDCl_3$)

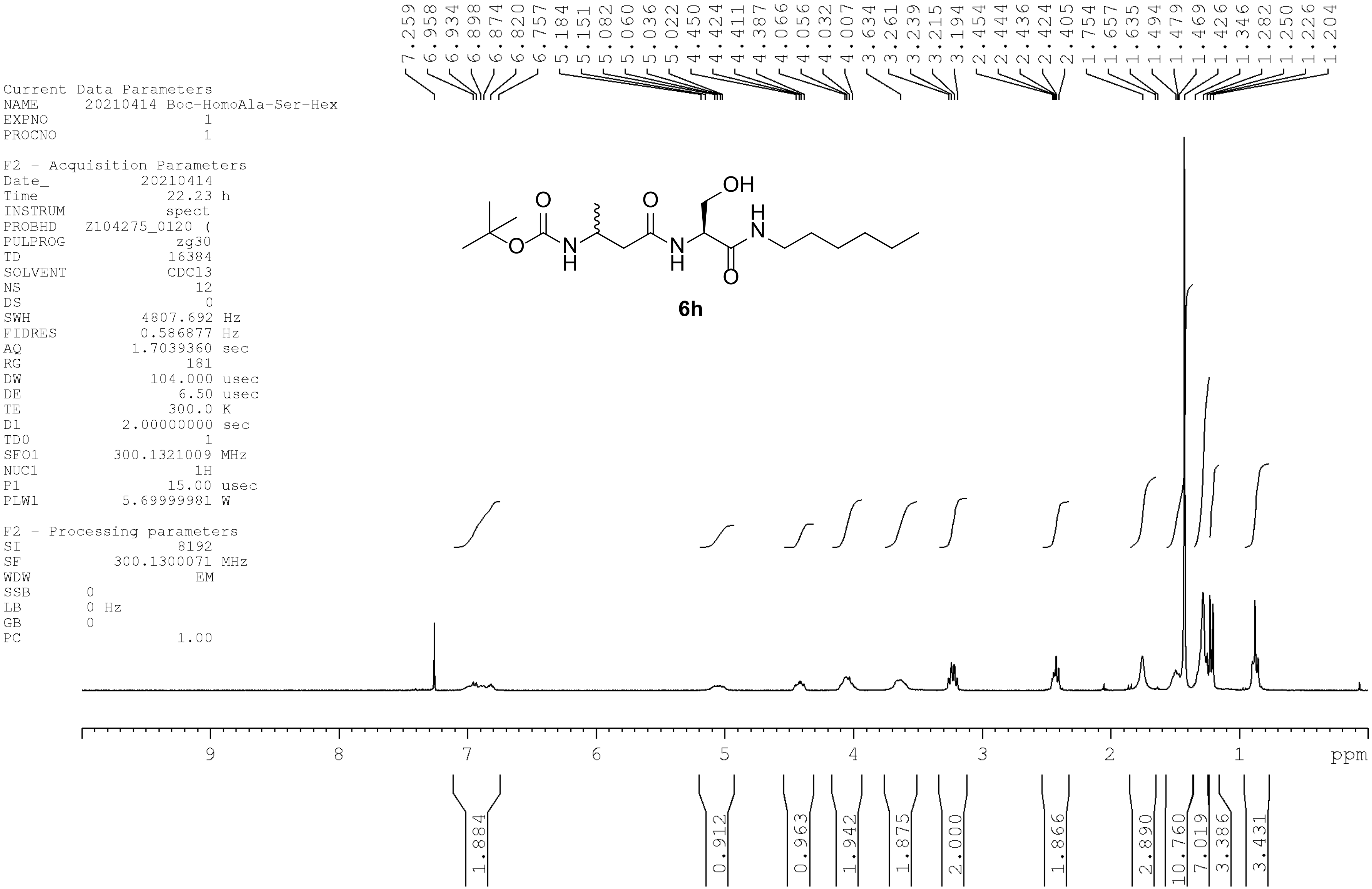

$^{13}C\{^{1}H\}$ NMR Spectrum of **6h** (75 MHz, $CDCl_3$)

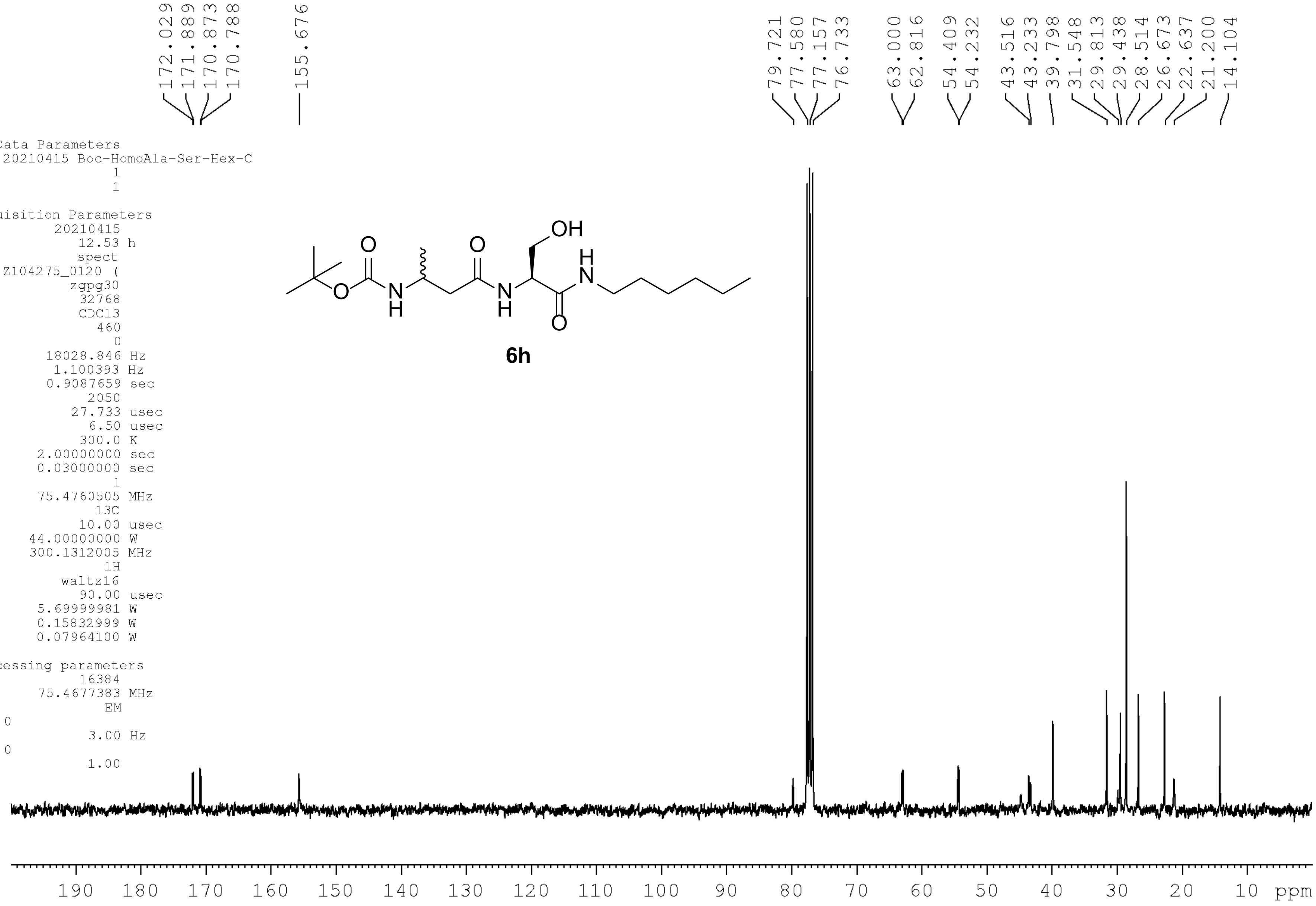


```
Current Data Parameters
NAME      20210415 Boc-HomoAla-Ser-Hex-C
EXPNO                  1
PROCNO                 1

F2 - Acquisition Parameters
Date_            20210415
Time               12.53 h
INSTRUM            spect
PROBHD   Z104275_0120 (
PULPROG           zgpg30
TD                 32768
SOLVENT            CDCl3
NS                   460
DS                     0
SWH            18028.846 Hz
FIDRES          1.100393 Hz
AQ             0.9087659 sec
RG                  2050
DW                27.733 usec
DE                  6.50 usec
TE                 300.0 K
D1            2.00000000 sec
D11           0.03000000 sec
TD0                    1
SFO1          75.4760505 MHz
NUC1                 13C
P1                 10.00 usec
PLW1         44.00000000 W
SFO2         300.1312005 MHz
NUC2                  1H
CPDPRG[2         waltz16
PCPD2              90.00 usec
PLW2          5.69999981 W
PLW12         0.15832999 W
PLW13         0.07964100 W

F2 - Processing parameters
SI                 16384
SF            75.4677383 MHz
WDW                   EM
SSB      0
LB                  3.00 Hz
GB       0
PC                  1.00
```

## $^{1}H$ NMR Spectrum of **6i** (300 MHz, $CDCl_3$)

```
Current Data Parameters
NAME     20191125 phe1+1 2
EXPNO                  1
PROCNO                 1

F2 - Acquisition Parameters
Date_           20191125
Time               18.22
INSTRUM            spect
PROBHD   5 mm PABBO BB-
PULPROG             zg30
TD                 16384
SOLVENT            CDC13
NS                    16
DS                     0
SWH             4807.692 Hz
FIDRES          0.293438 Hz
AQ             1.7039360 sec
RG                   144
DW               104.000 usec
DE                  6.50 usec
TE                 300.0 K
D1            2.00000000 sec
TD0                    1

======== CHANNEL f1 ========
NUC1                  1H
P1                 10.80 usec
PL1                -1.00 dB
PL1W         10.11928844 W
SFO1         300.1321009 MHz

F2 - Processing parameters
SI                  8192
SF           300.1300062 MHz
WDW                   EM
SSB                    0
LB                     0 Hz
GB                     0
PC                  1.00
```

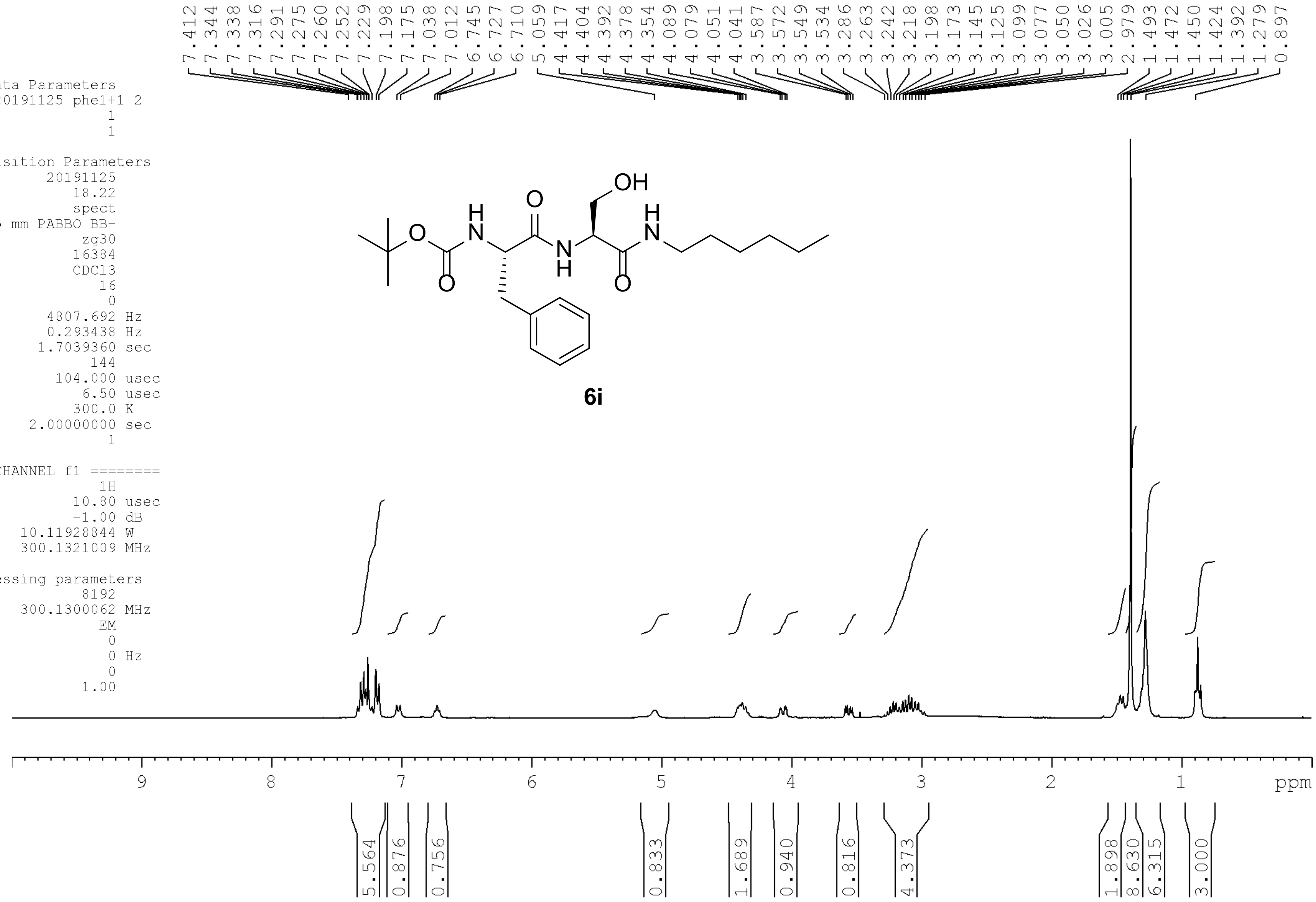

$^{13}C\{^1H\}$ NMR Spectrum of **6i** (100 MHz, $CDCl_3$)

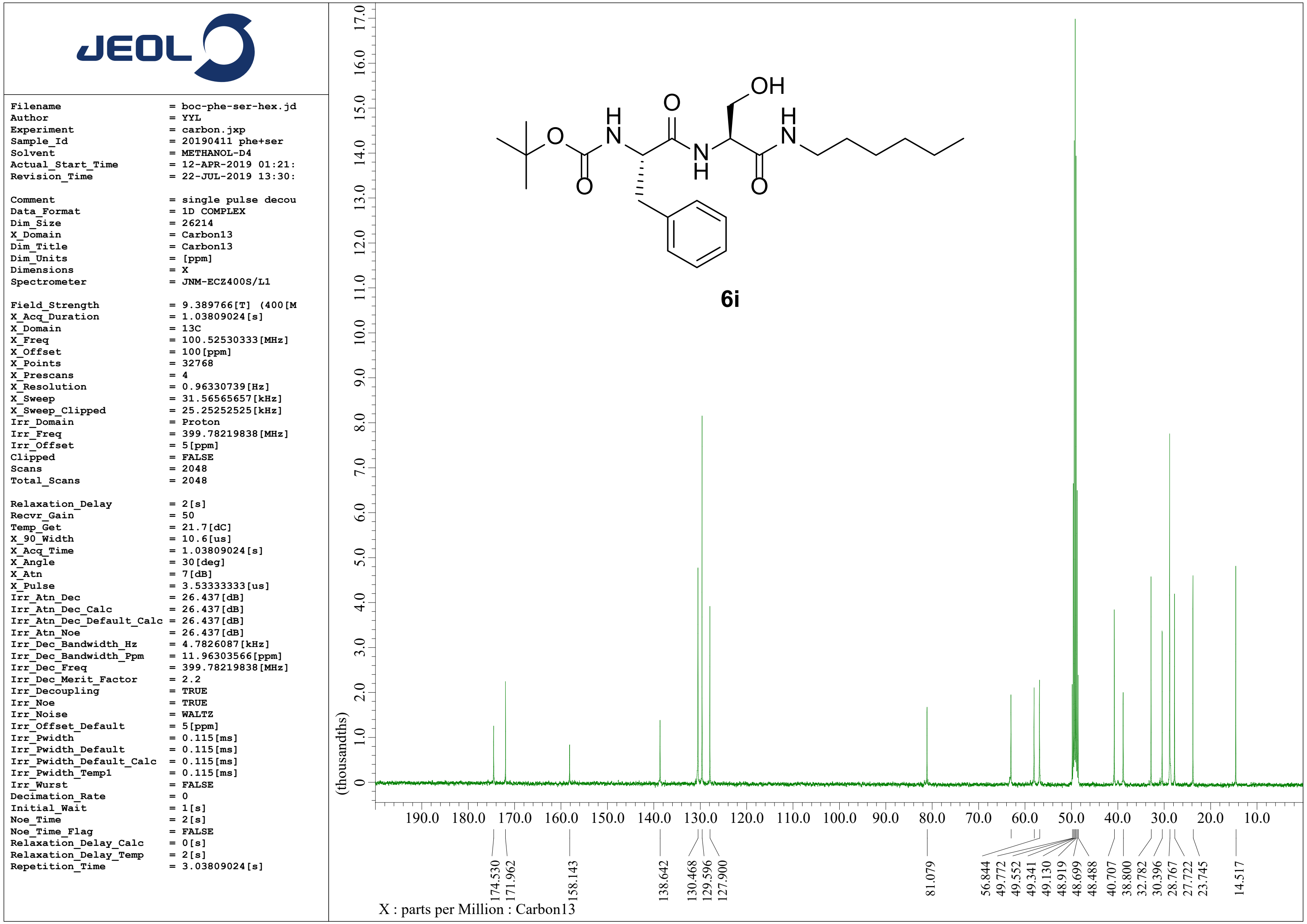

$^{1}$H NMR Spectrum of **6j** (300 MHz, $CDCl_3$)

```
Current Data Parameters
NAME      20200831 met-ser cp
EXPNO                    1
PROCNO                   1

F2 - Acquisition Parameters
Date_            20200831
Time                14.23
INSTRUM             spect
PROBHD    5 mm PABBO BB-
PULPROG              zg30
TD                  16384
SOLVENT             CDCl3
NS                     16
DS                      0
SWH              4807.692 Hz
FIDRES           0.293438 Hz
AQ              1.7039360 sec
RG                    203
DW                104.000 usec
DE                   6.50 usec
TE                  300.0 K
D1             2.00000000 sec
TD0                     1

======== CHANNEL f1 ========
NUC1                   1H
P1                  10.80 usec
PL1                 -1.00 dB
PL1W          10.11928844 W
SFO1          300.1321009 MHz

F2 - Processing parameters
SI                   8192
SF            300.1300062 MHz
WDW                    EM
SSB                     0
LB                      0 Hz
GB                      0
PC                   1.00
```

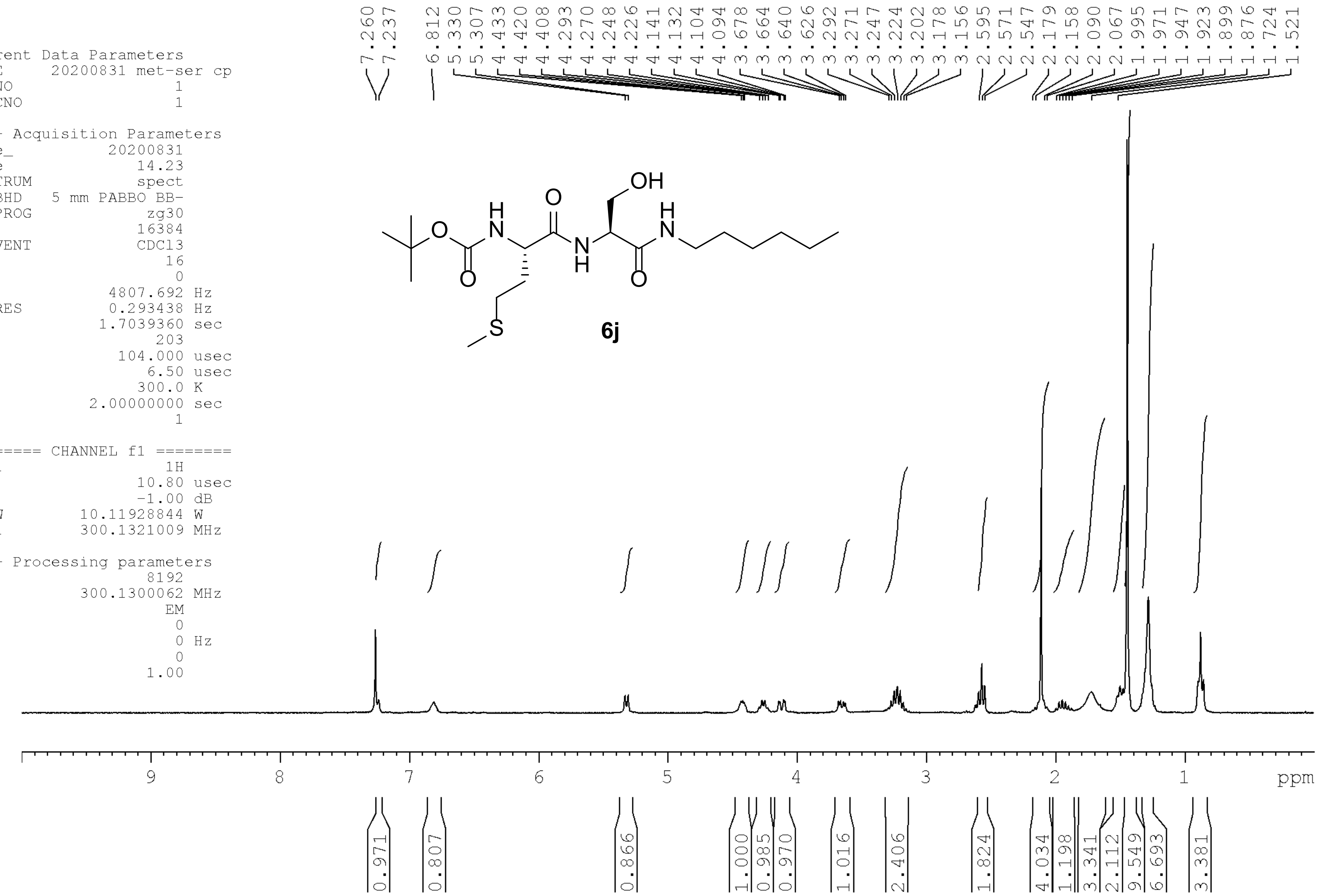

$^{13}C\{^{1}H\}$ NMR Spectrum of **6j** (100 MHz, $CDCl_3$)

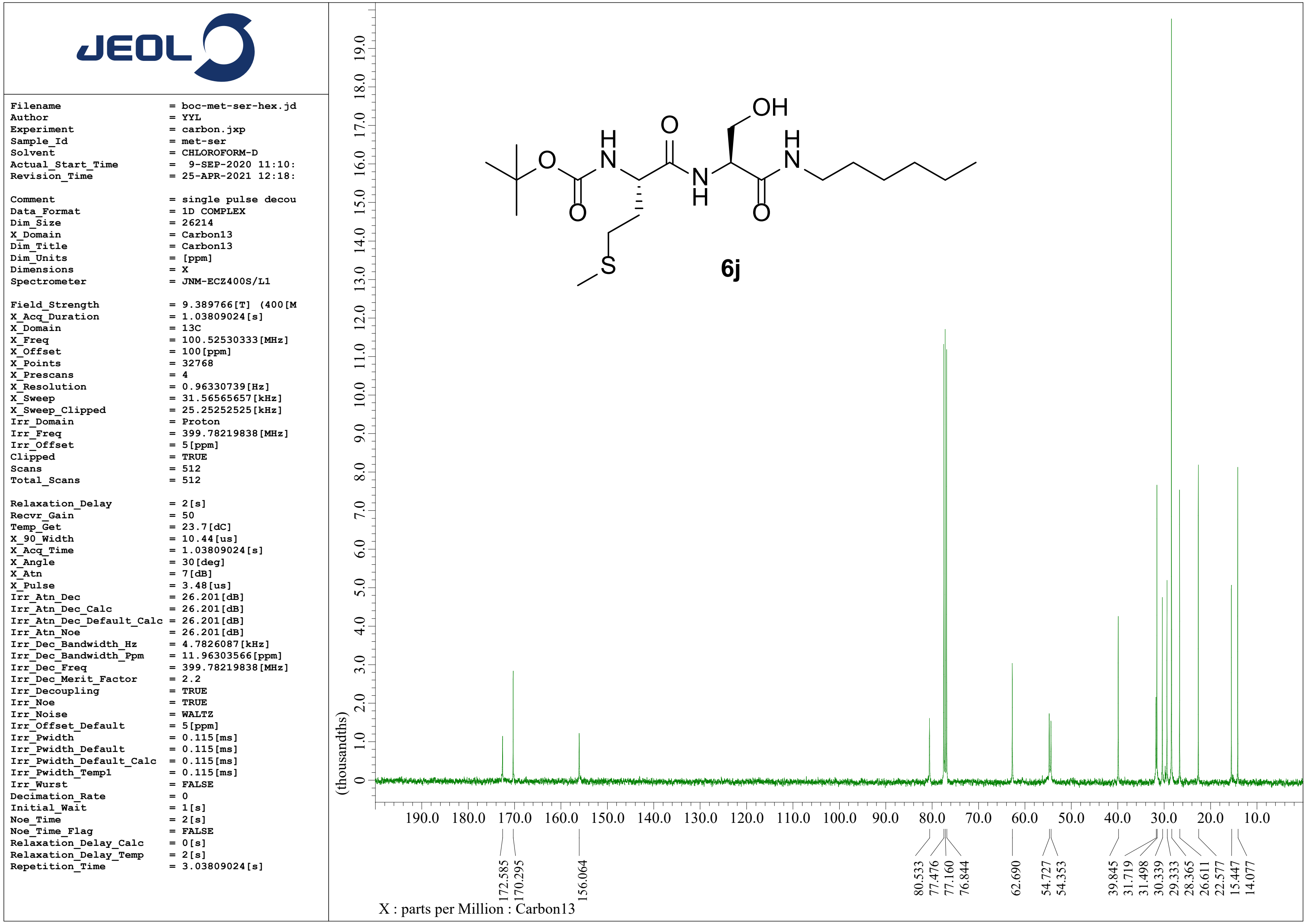

## $^1$H NMR Spectrum of **6k** (300 MHz, $CDCl_3$)

```
Current Data Parameters
NAME     20191013 pro 1+1 2
EXPNO                   1
PROCNO                  1

F2 - Acquisition Parameters
Date_          20191013
Time              22.21
INSTRUM           spect
PROBHD   5 mm PABBO BB-
PULPROG            zg30
TD                16384
SOLVENT           CDCl3
NS                   16
DS                    0
SWH            4807.692 Hz
FIDRES         0.293438 Hz
AQ            1.7039360 sec
RG                  101
DW              104.000 usec
DE                 6.50 usec
TE                300.0 K
D1           2.00000000 sec
TD0                   1

======== CHANNEL f1 ========
NUC1                 1H
P1                10.80 usec
PL1               -1.00 dB
PL1W        10.11928844 W
SFO1        300.1321009 MHz

F2 - Processing parameters
SI                 8192
SF          300.1300062 MHz
WDW                  EM
SSB                   0
LB                    0 Hz
GB                    0
PC                 1.00
```

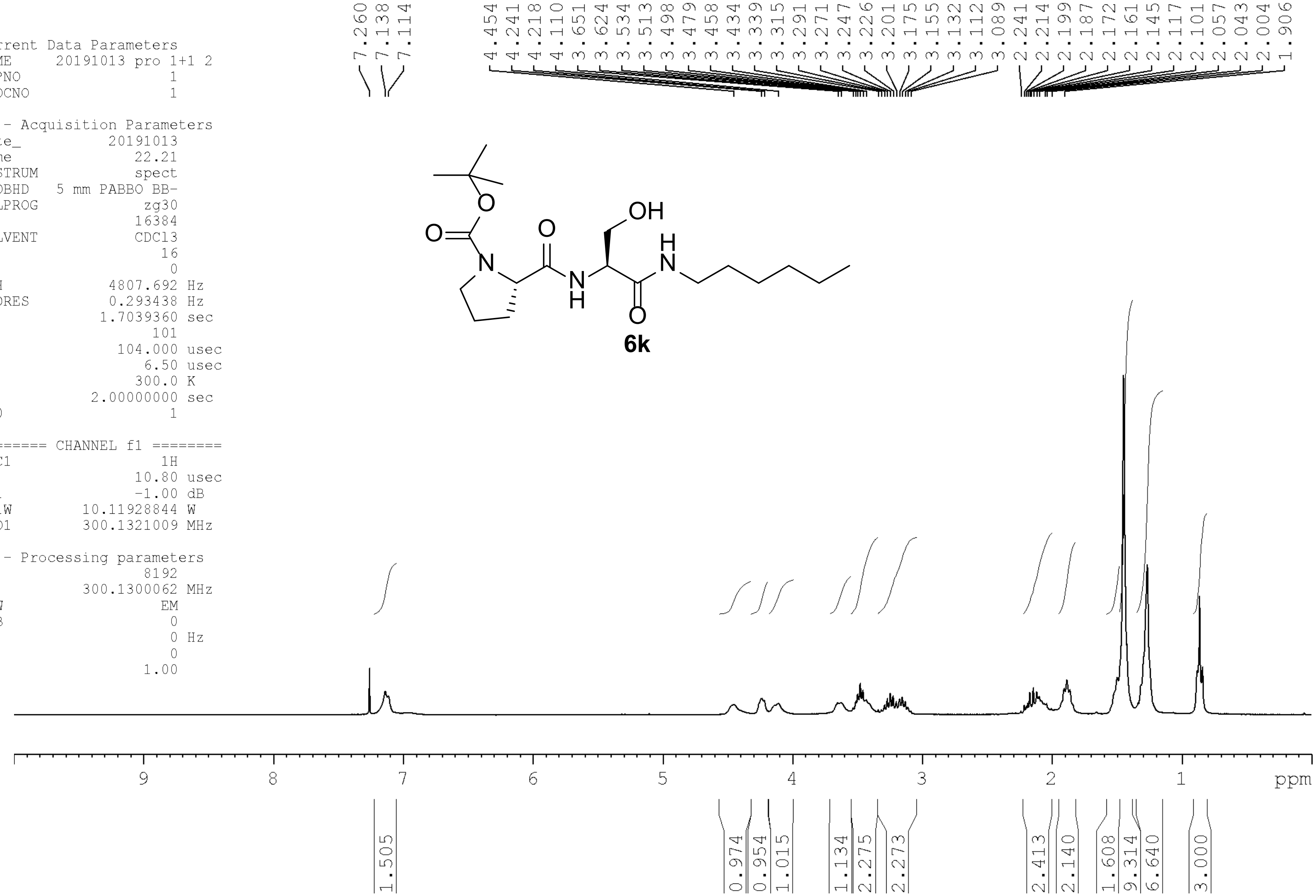


**6k**

$^{13}C\{^{1}H\}$ NMR Spectrum of **6k** (100 MHz, $CDCl_3$)

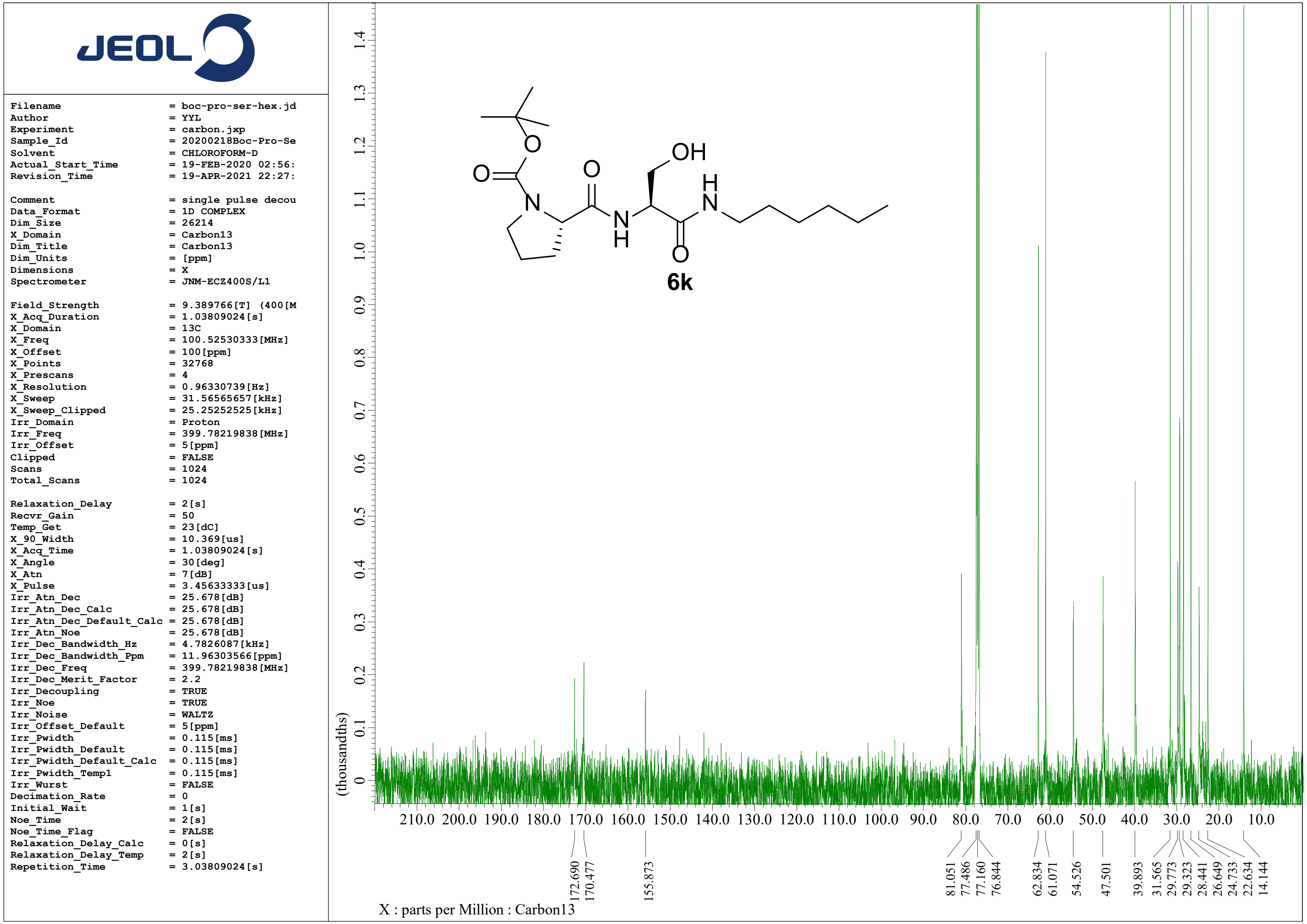

$^{1}$H NMR Spectrum of **6l** (300 MHz, $CDCl_3$)

```
Current Data Parameters
NAME      20210713 cys-ser 1
EXPNO                    1
PROCNO                   1

F2 - Acquisition Parameters
Date_          20210713
Time              16.07 h
INSTRUM           spect
PROBHD   Z104275_0120 (
PULPROG            zg30
TD                16384
SOLVENT           CDCl3
NS                   16
DS                    0
SWH            4807.692 Hz
FIDRES         0.586877 Hz
AQ            1.7039360 sec
RG                  161
DW              104.000 usec
DE                 6.50 usec
TE                300.0 K
D1           2.00000000 sec
TD0                   1
SFO1        300.1321009 MHz
NUC1                 1H
P1                15.00 usec
PLW1         5.69999981 W

F2 - Processing parameters
SI                 8192
SF          300.1300076 MHz
WDW                  EM
SSB      0
LB       0 Hz
GB       0
PC                 1.00
```

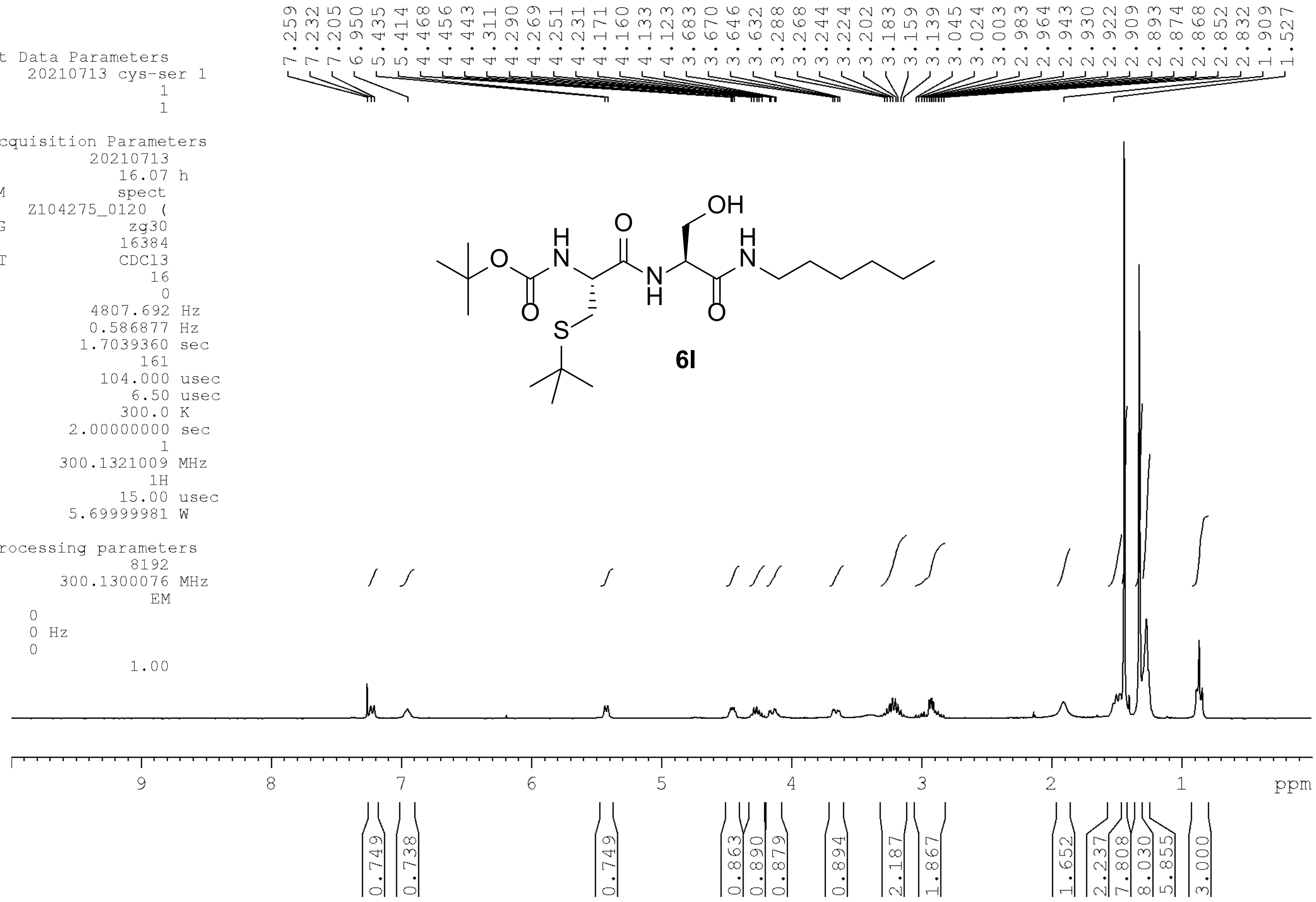

## $^{13}C\{^{1}H\}$ NMR Spectrum of **6l** (100 MHz, $CDCl_3$)

| | |
|---|---|
| Filename | = cys-ser_Carbon-1-2 |
| Author | = YYL |
| Experiment | = carbon.jxp |
| Sample_Id | = cys-ser |
| Solvent | = CHLOROFORM-D |
| Actual_Start_Time | = 3-AUG-2021 12:10: |
| Revision_Time | = 5-AUG-2021 22:40: |
| Comment | = single pulse decou |
| Data_Format | = 1D COMPLEX |
| Dim_Size | = 26214 |
| X_Domain | = Carbon13 |
| Dim_Title | = Carbon13 |
| Dim_Units | = [ppm] |
| Dimensions | = X |
| Spectrometer | = JNM-ECZ400S/L1 |
| Field_Strength | = 9.389766[T] (400[M |
| X_Acq_Duration | = 1.03809024[s] |
| X_Domain | = 13C |
| X_Freq | = 100.52530333[MHz] |
| X_Offset | = 100[ppm] |
| X_Points | = 32768 |
| X_Prescans | = 4 |
| X_Resolution | = 0.96330739[Hz] |
| X_Sweep | = 31.56565657[kHz] |
| X_Sweep_Clipped | = 25.25252525[kHz] |
| Irr_Domain | = Proton |
| Irr_Freq | = 399.78219838[MHz] |
| Irr_Offset | = 5[ppm] |
| Clipped | = FALSE |
| Scans | = 2048 |
| Total_Scans | = 2048 |
| Relaxation_Delay | = 2[s] |
| Recvr_Gain | = 50 |
| Temp_Get | = 22.9[dC] |
| X_90_Width | = 10.3[us] |
| X_Acq_Time | = 1.03809024[s] |
| X_Angle | = 30[deg] |
| X_Atn | = 7[dB] |
| X_Pulse | = 3.43333333[us] |
| Irr_Atn_Dec | = 25.484[dB] |
| Irr_Atn_Dec_Calc | = 25.484[dB] |
| Irr_Atn_Dec_Default_Calc | = 25.484[dB] |
| Irr_Atn_Noe | = 25.484[dB] |
| Irr_Dec_Bandwidth_Hz | = 4.7826087[kHz] |
| Irr_Dec_Bandwidth_Ppm | = 11.96303566[ppm] |
| Irr_Dec_Freq | = 399.78219838[MHz] |
| Irr_Dec_Merit_Factor | = 2.2 |
| Irr_Decoupling | = TRUE |
| Irr_Noe | = TRUE |
| Irr_Noise | = WALTZ |
| Irr_Offset_Default | = 5[ppm] |
| Irr_Pwidth | = 0.115[ms] |
| Irr_Pwidth_Default | = 0.115[ms] |
| Irr_Pwidth_Default_Calc | = 0.115[ms] |
| Irr_Pwidth_Templ | = 0.115[ms] |
| Irr_Wurst | = FALSE |
| Decimation_Rate | = 0 |
| Initial_Wait | = 1[s] |
| Noe_Time | = 2[s] |
| Noe_Time_Flag | = FALSE |
| Relaxation_Delay_Calc | = 0[s] |
| Relaxation_Delay_Temp | = 2[s] |
| Repetition_Time | = 3.03809024[s] |

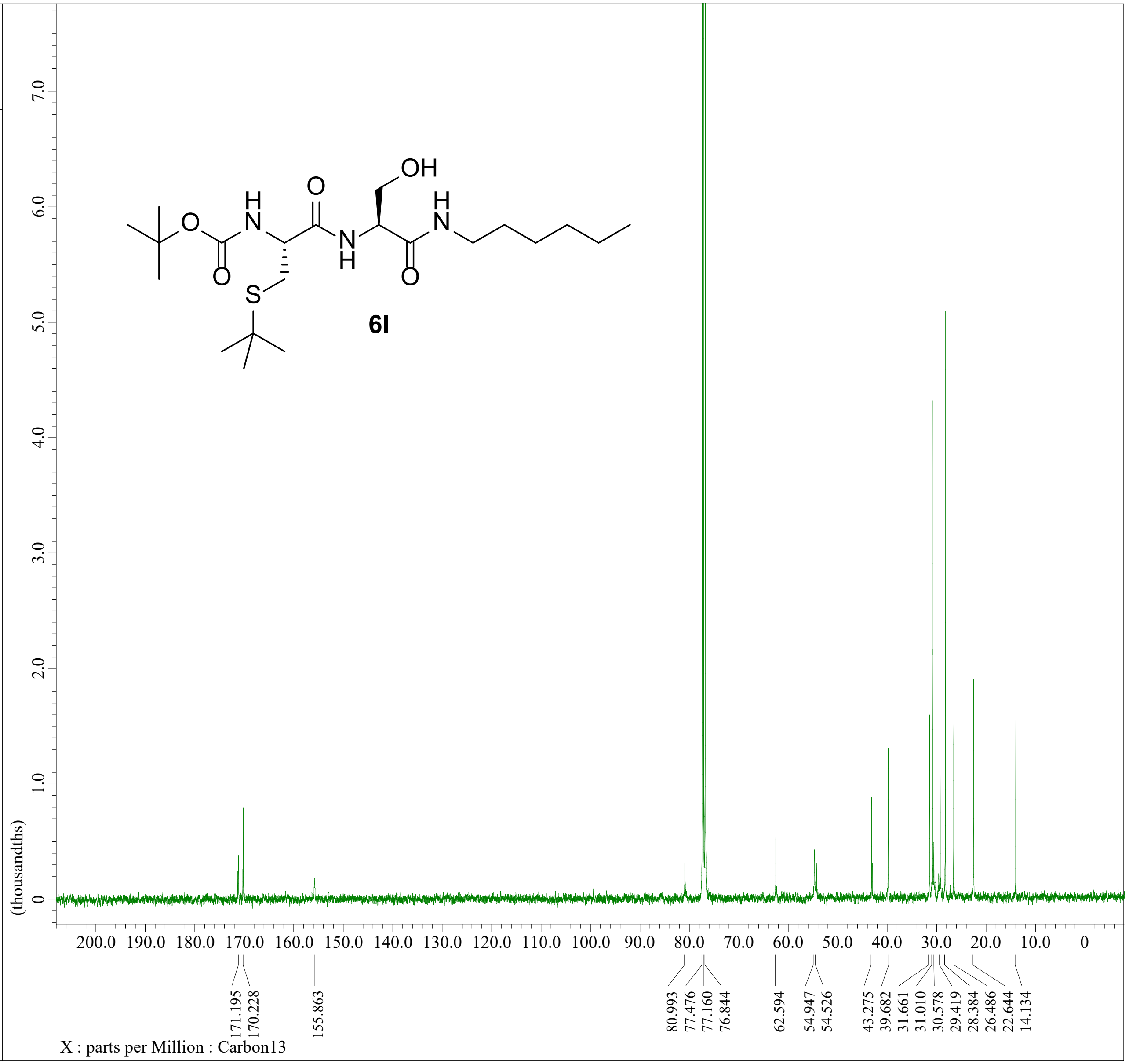

## $^1$H NMR Spectrum of **6m** (300 MHz, $CDCl_3$)

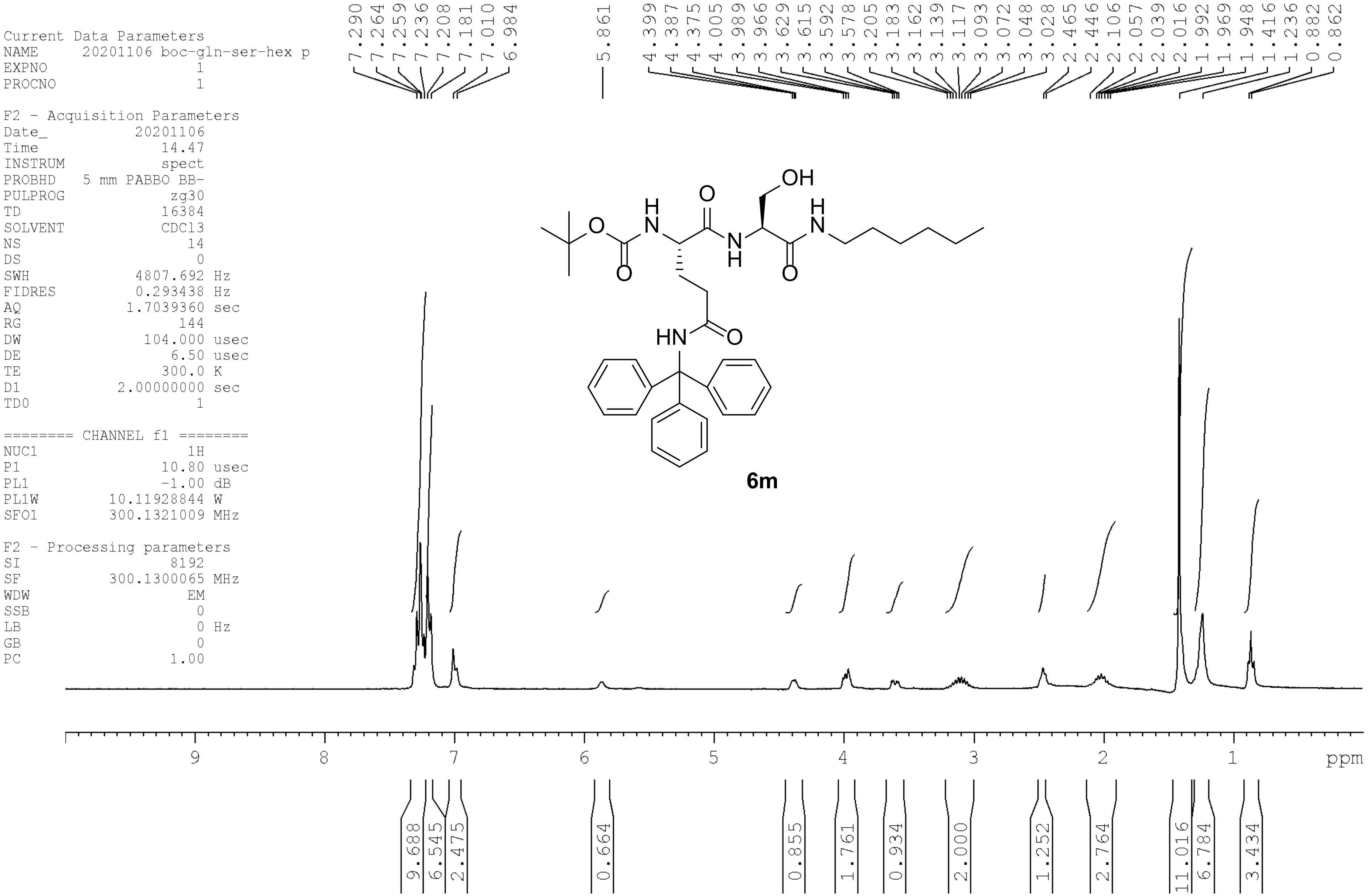

$^{13}C\{^{1}H\}$ NMR Spectrum of **6m** (100 MHz, $CDCl_3$)

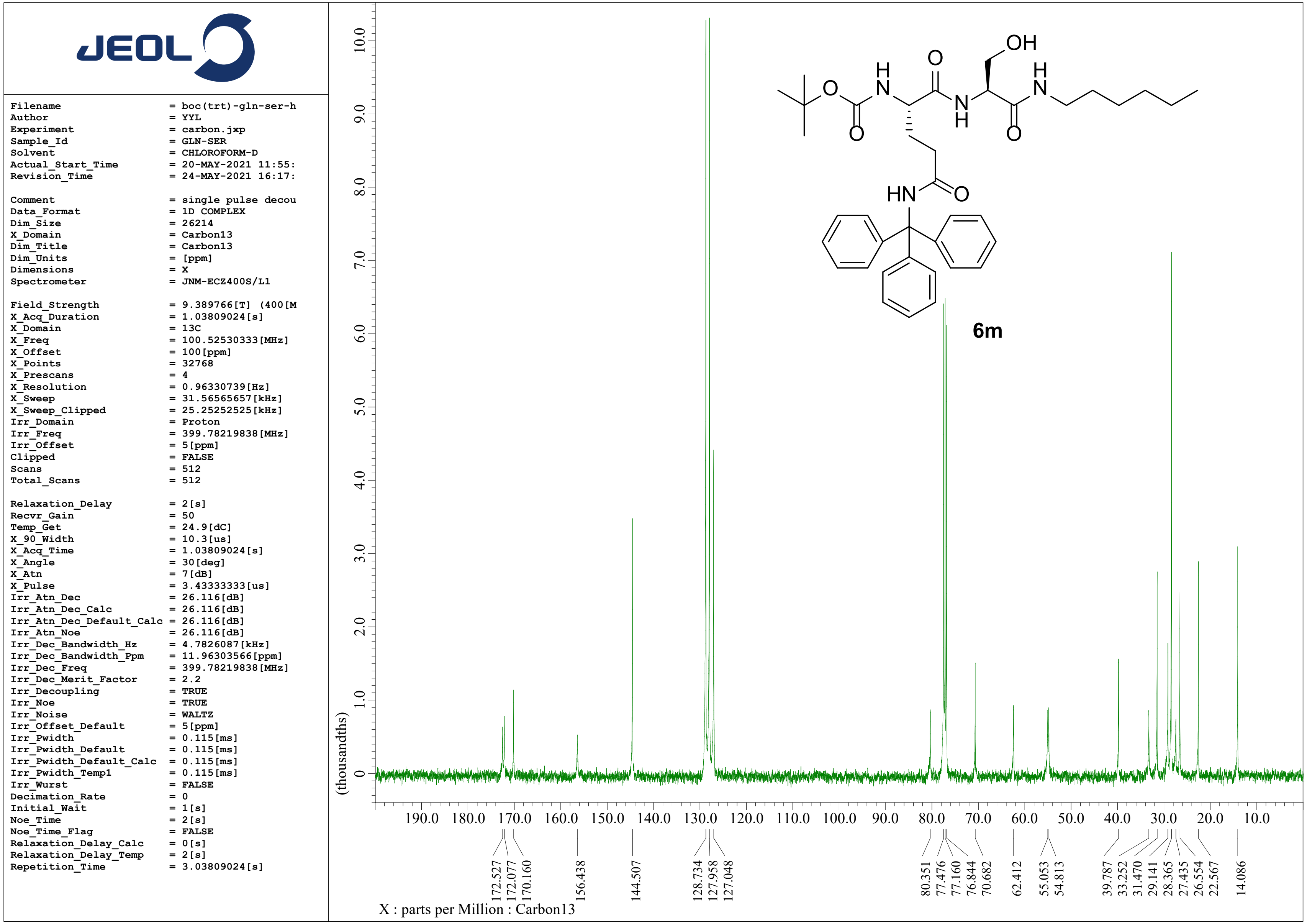

$^{1}$H NMR Spectrum of **6n** (300 MHz, $CD_3OD$)

```
Current Data Parameters
NAME      20200820 boc-trt-asn-ser cd3od
EXPNO                    1
PROCNO                   1

F2 - Acquisition Parameters
Date_             20200820
Time                 12.56
INSTRUM              spect
PROBHD   5 mm PABBO BB-
PULPROG               zg30
TD                   16384
SOLVENT              CDCl3
NS                      16
DS                       0
SWH               4807.692 Hz
FIDRES            0.293438 Hz
AQ               1.7039360 sec
RG                     161
DW                 104.000 usec
DE                    6.50 usec
TE                   300.0 K
D1              2.00000000 sec
TD0                      1

======== CHANNEL f1 ========
NUC1                    1H
P1                   10.80 usec
PL1                  -1.00 dB
PL1W           10.11928844 W
SFO1           300.1321009 MHz

F2 - Processing parameters
SI                    8192
SF             300.1311870 MHz
WDW                     EM
SSB                      0
LB                       0 Hz
GB                       0
PC                    1.00
```

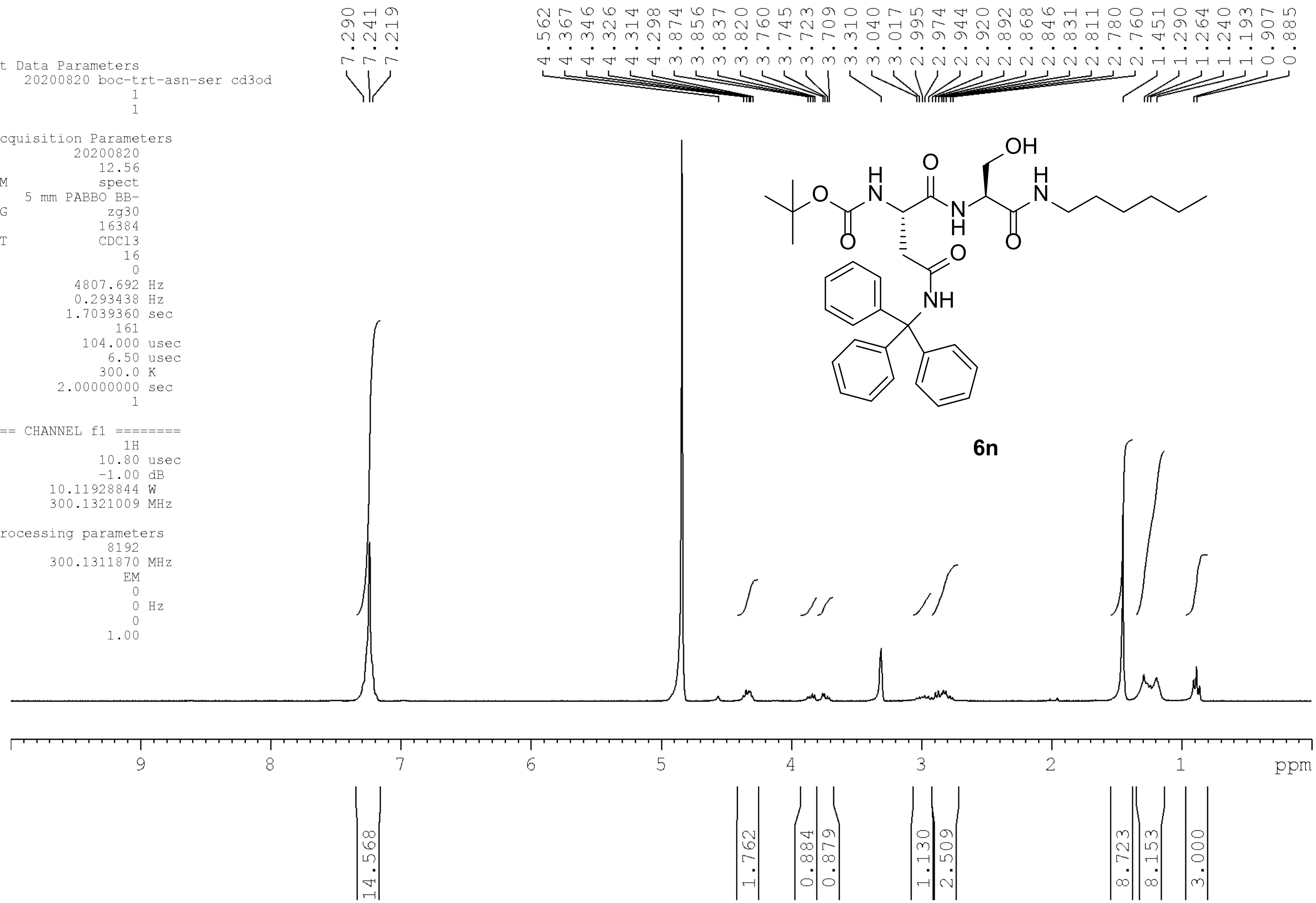

## $^{13}C\{^1H\}$ NMR Spectrum of **6n** (100 MHz, $CD_3OD$)

```
Filename                 = boc-trt-asn-ser-he
Author                   = YYL
Experiment               = carbon.jxp
Sample_Id                = BOC-TRT-ASN-SER-HE
Solvent                  = METHANOL-D4
Actual_Start_Time        =  9-SEP-2020 12:14:
Revision_Time            = 26-APR-2021 22:46:

Comment                  = single pulse decou
Data_Format              = 1D COMPLEX
Dim_Size                 = 26214
X_Domain                 = Carbon13
Dim_Title                = Carbon13
Dim_Units                = [ppm]
Dimensions               = X
Spectrometer             = JNM-ECZ400S/L1

Field_Strength           = 9.389766[T] (400[M
X_Acq_Duration           = 1.03809024[s]
X_Domain                 = 13C
X_Freq                   = 100.52530333[MHz]
X_Offset                 = 100[ppm]
X_Points                 = 32768
X_Prescans               = 4
X_Resolution             = 0.96330739[Hz]
X_Sweep                  = 31.56565657[kHz]
X_Sweep_Clipped          = 25.25252525[kHz]
Irr_Domain               = Proton
Irr_Freq                 = 399.78219838[MHz]
Irr_Offset               = 5[ppm]
Clipped                  = TRUE
Scans                    = 512
Total_Scans              = 512

Relaxation_Delay         = 2[s]
Recvr_Gain               = 50
Temp_Get                 = 23.6[dC]
X_90_Width               = 10.44[us]
X_Acq_Time               = 1.03809024[s]
X_Angle                  = 30[deg]
X_Atn                    = 7[dB]
X_Pulse                  = 3.48[us]
Irr_Atn_Dec              = 26.201[dB]
Irr_Atn_Dec_Calc         = 26.201[dB]
Irr_Atn_Dec_Default_Calc = 26.201[dB]
Irr_Atn_Noe              = 26.201[dB]
Irr_Dec_Bandwidth_Hz     = 4.7826087[kHz]
Irr_Dec_Bandwidth_Ppm    = 11.96303566[ppm]
Irr_Dec_Freq             = 399.78219838[MHz]
Irr_Dec_Merit_Factor     = 2.2
Irr_Decoupling           = TRUE
Irr_Noe                  = TRUE
Irr_Noise                = WALTZ
Irr_Offset_Default       = 5[ppm]
Irr_Pwidth               = 0.115[ms]
Irr_Pwidth_Default       = 0.115[ms]
Irr_Pwidth_Default_Calc  = 0.115[ms]
Irr_Pwidth_Templ         = 0.115[ms]
Irr_Wurst                = FALSE
Decimation_Rate          = 0
Initial_Wait             = 1[s]
Noe_Time                 = 2[s]
Noe_Time_Flag            = FALSE
Relaxation_Delay_Calc    = 0[s]
Relaxation_Delay_Temp    = 2[s]
Repetition_Time          = 3.03809024[s]
```

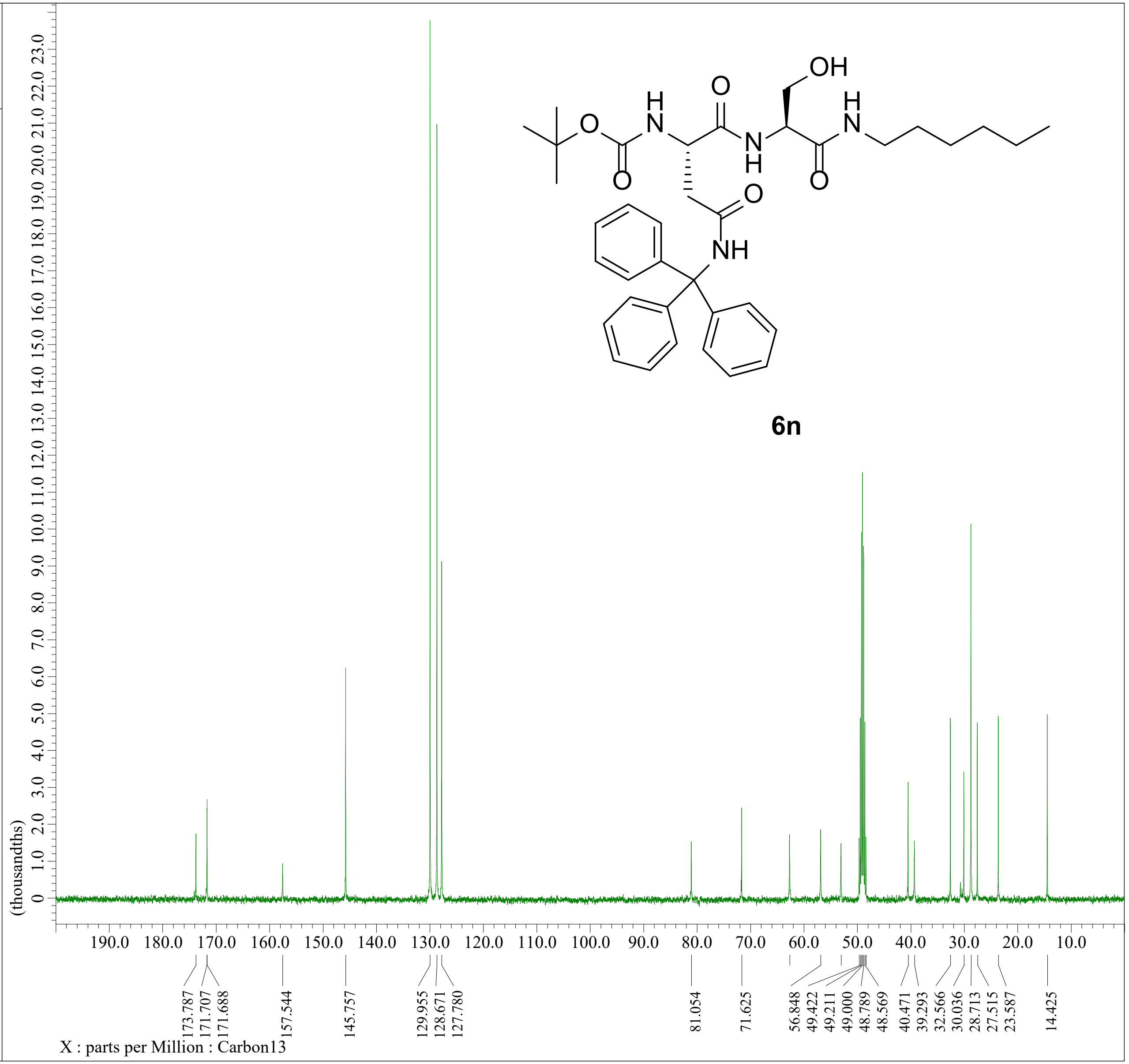

$^{1}$H NMR Spectrum of **6o** (300 MHz, $CD_3OD$)

```
Current Data Parameters
NAME     20201217 boc-trp-ser-hex p new
EXPNO                 1
PROCNO                1

F2 - Acquisition Parameters
Date_          20201217
Time              11.39
INSTRUM           spect
PROBHD   5 mm PABBO BB-
PULPROG            zg30
TD                16384
SOLVENT            MeOD
NS                   16
DS                    0
SWH            4807.692 Hz
FIDRES         0.293438 Hz
AQ            1.7039360 sec
RG                  128
DW              104.000 usec
DE                 6.50 usec
TE                300.0 K
D1           2.00000000 sec
TD0                   1

======== CHANNEL f1 ========
NUC1                 1H
P1                10.80 usec
PL1               -1.00 dB
PL1W        10.11928844 W
SFO1        300.1321009 MHz

F2 - Processing parameters
SI                 8192
SF          300.1300047 MHz
WDW                  EM
SSB                   0
LB                    0 Hz
GB                    0
PC                 1.00
```

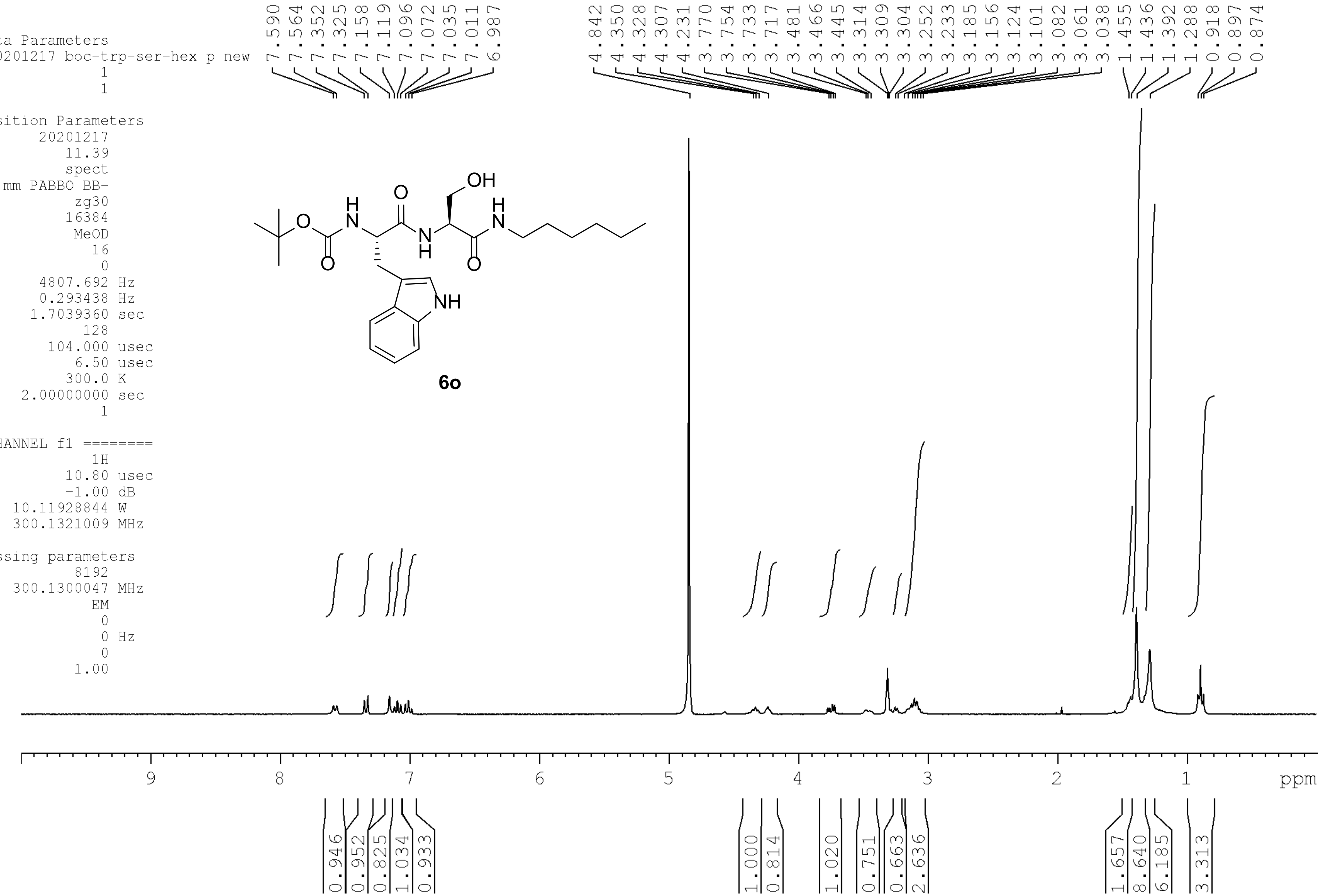

## $^{13}C\{^1H\}$ NMR Spectrum of **6o** (100 MHz, $CD_3OD$)

| | |
|---|---|
| Filename | = boc-trp-ser-hex.jd |
| Author | = yyl |
| Experiment | = carbon.jxp |
| Sample_Id | = 20200225 trp 1+1 |
| Solvent | = METHANOL-D4 |
| Actual_Start_Time | = 25-FEB-2021 12:23: |
| Revision_Time | = 20-APR-2021 13:44: |
| Comment | = single pulse decou |
| Data_Format | = 1D COMPLEX |
| Dim_Size | = 26214 |
| X_Domain | = Carbon13 |
| Dim_Title | = Carbon13 |
| Dim_Units | = [ppm] |
| Dimensions | = X |
| Spectrometer | = JNM-ECZ400S/L1 |
| Field_Strength | = 9.389766[T] (400[M |
| X_Acq_Duration | = 1.03809024[s] |
| X_Domain | = 13C |
| X_Freq | = 100.52530333[MHz] |
| X_Offset | = 100[ppm] |
| X_Points | = 32768 |
| X_Prescans | = 4 |
| X_Resolution | = 0.96330739[Hz] |
| X_Sweep | = 31.56565657[kHz] |
| X_Sweep_Clipped | = 25.25252525[kHz] |
| Irr_Domain | = Proton |
| Irr_Freq | = 399.78219838[MHz] |
| Irr_Offset | = 5[ppm] |
| Clipped | = FALSE |
| Scans | = 1024 |
| Total_Scans | = 1024 |
| Relaxation_Delay | = 2[s] |
| Recvr_Gain | = 50 |
| Temp_Get | = 22.1[dC] |
| X_90_Width | = 10.3[us] |
| X_Acq_Time | = 1.03809024[s] |
| X_Angle | = 30[deg] |
| X_Atn | = 7[dB] |
| X_Pulse | = 3.43333333[us] |
| Irr_Atn_Dec | = 26.116[dB] |
| Irr_Atn_Dec_Calc | = 26.116[dB] |
| Irr_Atn_Dec_Default_Calc | = 26.116[dB] |
| Irr_Atn_Noe | = 26.116[dB] |
| Irr_Dec_Bandwidth_Hz | = 4.7826087[kHz] |
| Irr_Dec_Bandwidth_Ppm | = 11.96303566[ppm] |
| Irr_Dec_Freq | = 399.78219838[MHz] |
| Irr_Dec_Merit_Factor | = 2.2 |
| Irr_Decoupling | = TRUE |
| Irr_Noe | = TRUE |
| Irr_Noise | = WALTZ |
| Irr_Offset_Default | = 5[ppm] |
| Irr_Pwidth | = 0.115[ms] |
| Irr_Pwidth_Default | = 0.115[ms] |
| Irr_Pwidth_Default_Calc | = 0.115[ms] |
| Irr_Pwidth_Templ | = 0.115[ms] |
| Irr_Wurst | = FALSE |
| Decimation_Rate | = 0 |
| Initial_Wait | = 1[s] |
| Noe_Time | = 2[s] |
| Noe_Time_Flag | = FALSE |
| Relaxation_Delay_Calc | = 0[s] |
| Relaxation_Delay_Temp | = 2[s] |
| Repetition_Time | = 3.03809024[s] |

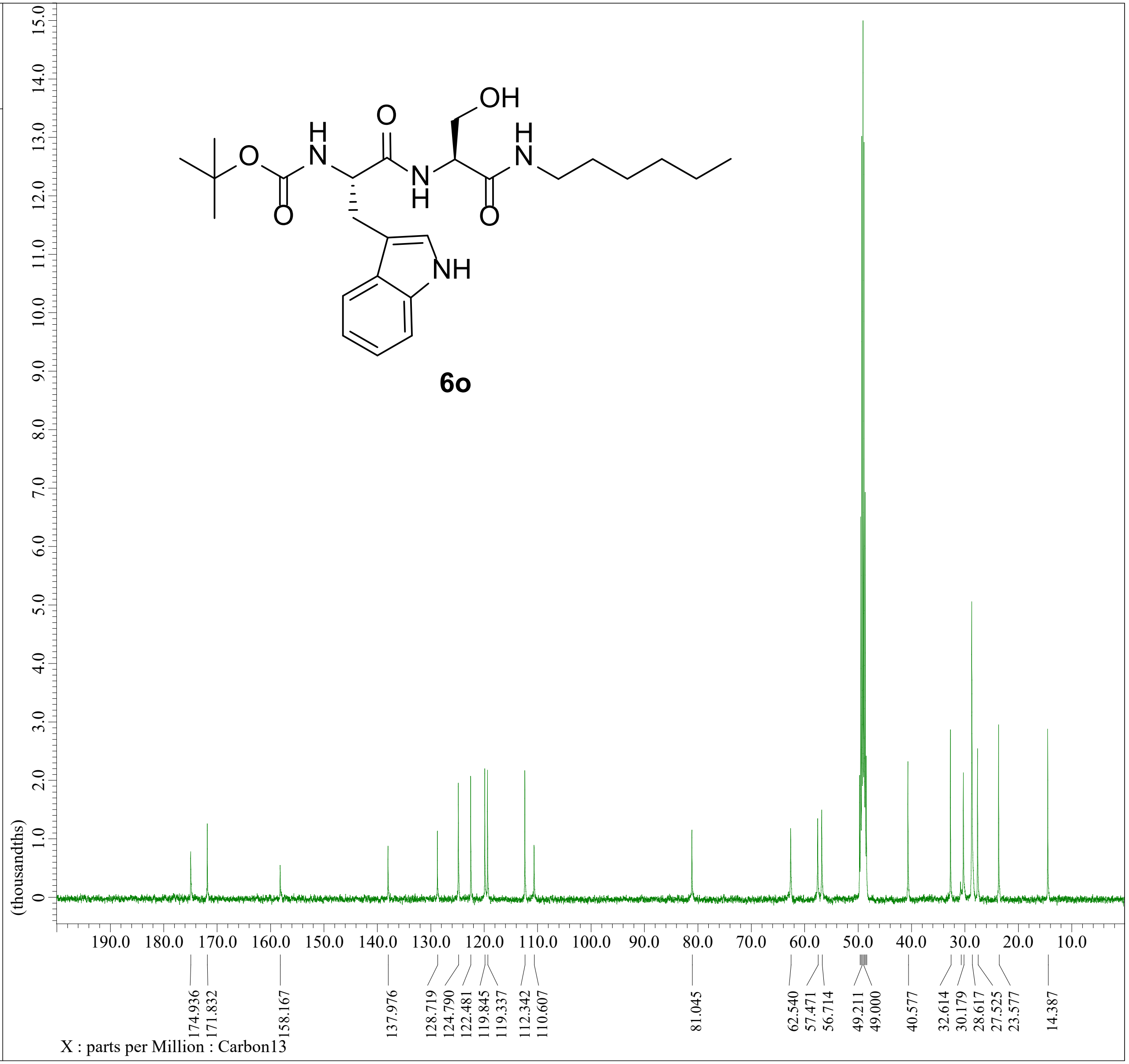

## $^{1}$H NMR Spectrum of **6p** (300 MHz, $CD_3OD$)

```
Current Data Parameters
NAME     20201125 Boc-Ser-Ser-Hex-CD3OD
EXPNO                  1
PROCNO                 1

F2 - Acquisition Parameters
Date_          20201125
Time              17.07
INSTRUM           spect
PROBHD   5 mm PABBO BB-
PULPROG            zg30
TD                16384
SOLVENT            MeOD
NS                   16
DS                    0
SWH            4807.692 Hz
FIDRES         0.293438 Hz
AQ            1.7039360 sec
RG                  128
DW              104.000 usec
DE                 6.50 usec
TE                300.0 K
D1           2.00000000 sec
TD0                   1

======== CHANNEL f1 ========
NUC1                 1H
P1                10.80 usec
PL1               -1.00 dB
PL1W        10.11928844 W
SFO1        300.1321009 MHz

F2 - Processing parameters
SI                 8192
SF          300.1300044 MHz
WDW                  EM
SSB      0
LB       0 Hz
GB       0
PC                 1.00
```

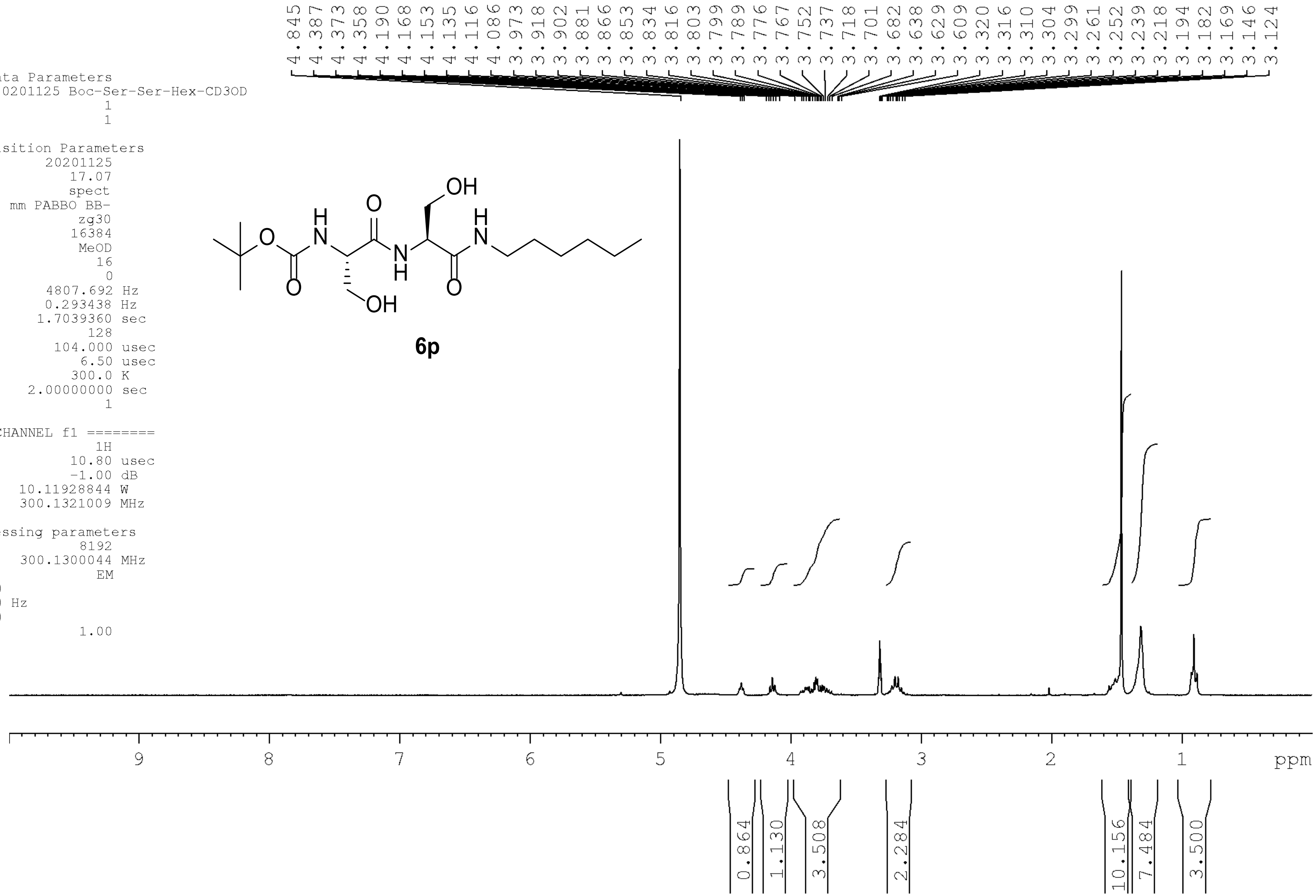

$^{13}C\{^{1}H\}$ NMR Spectrum of **6p** (75 MHz, $CD_3OD$)

```
Current Data Parameters
NAME     20201125 Boc-Ser-Ser-Hex-C
EXPNO                 1
PROCNO                1

F2 - Acquisition Parameters
Date_           20201125
Time               19.25
INSTRUM            spect
PROBHD   5 mm PABBO BB-
PULPROG          zgpg30
TD                32768
SOLVENT            MeOD
NS                  537
DS                    0
SWH           18028.846 Hz
FIDRES         0.550197 Hz
AQ            0.9087659 sec
RG                 2050
DW               27.733 usec
DE                 6.50 usec
TE                300.0 K
D1           2.00000000 sec
D11          0.03000000 sec
TD0                   1

======== CHANNEL f1 ========
NUC1                13C
P1                 9.50 usec
PL1               -1.00 dB
PL1W        46.16925430 W
SFO1         75.4760505 MHz

======== CHANNEL f2 ========
CPDPRG[2        waltz16
NUC2                 1H
PCPD2             90.00 usec
PL2                1.00 dB
PL12              17.29 dB
PL13              22.00 dB
PL2W         6.38483953 W
PL12W        0.15002026 W
PL13W        0.05071658 W
SFO2        300.1312005 MHz

F2 - Processing parameters
SI                16384
SF           75.4676467 MHz
WDW                  EM
SSB      0
LB                 3.00 Hz
GB       0
PC                 1.00
```

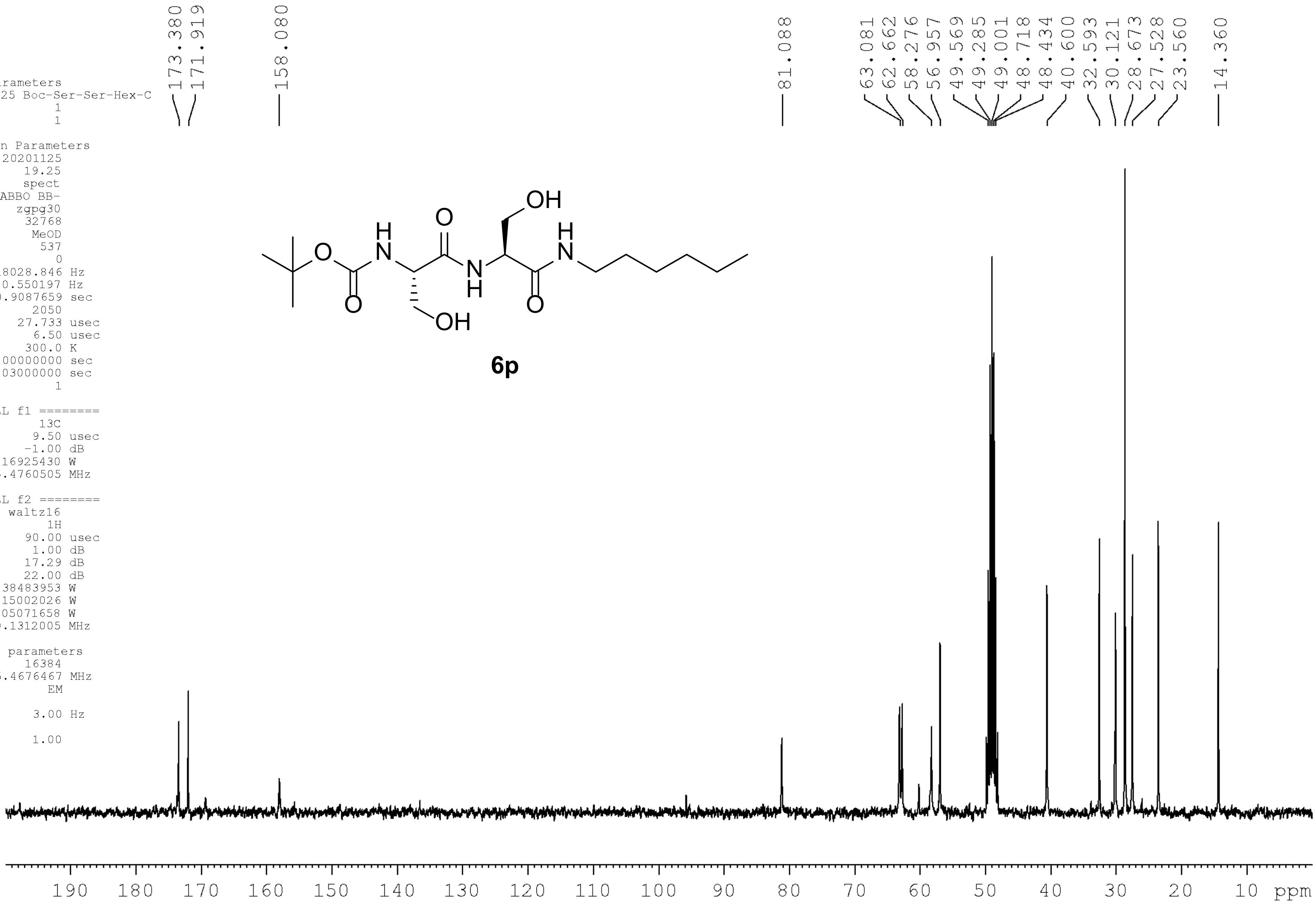

**6p**

$^{1}$H NMR Spectrum of **6q** (300 MHz, $CDCl_3$)

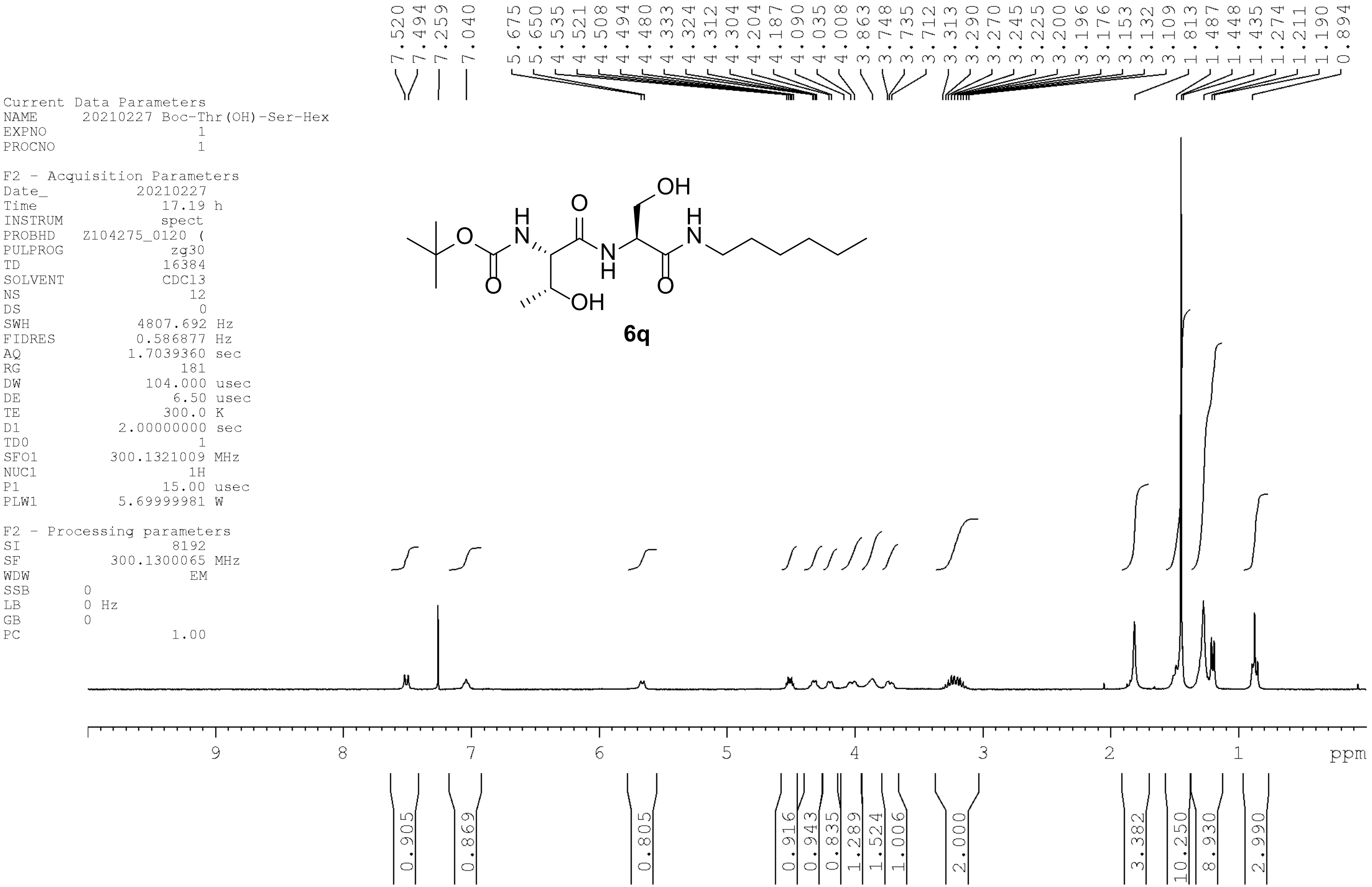

## $^{13}C\{^{1}H\}$ NMR Spectrum of **6q** (75 MHz, $CDCl_3$)

```
Current Data Parameters
NAME     20210227 Boc-Thr(OH)-Ser-Hex-C
EXPNO              1
PROCNO             1

F2 - Acquisition Parameters
Date_          20210227
Time              17.48 h
INSTRUM           spect
PROBHD   Z104275_0120 (
PULPROG          zgpg30
TD                32768
SOLVENT           CDCl3
NS                  440
DS                    0
SWH           18028.846 Hz
FIDRES         1.100393 Hz
AQ            0.9087659 sec
RG                 2050
DW               27.733 usec
DE                 6.50 usec
TE                300.0 K
D1           2.00000000 sec
D11          0.03000000 sec
TD0                   1
SFO1         75.4760505 MHz
NUC1                13C
P1                10.00 usec
PLW1        44.00000000 W
SFO2        300.1312005 MHz
NUC2                 1H
CPDPRG[2        waltz16
PCPD2             90.00 usec
PLW2         5.69999981 W
PLW12        0.15832999 W
PLW13        0.07964100 W

F2 - Processing parameters
SI                16384
SF           75.4677394 MHz
WDW                  EM
SSB      0
LB                 3.00 Hz
GB       0
PC                 1.00
```

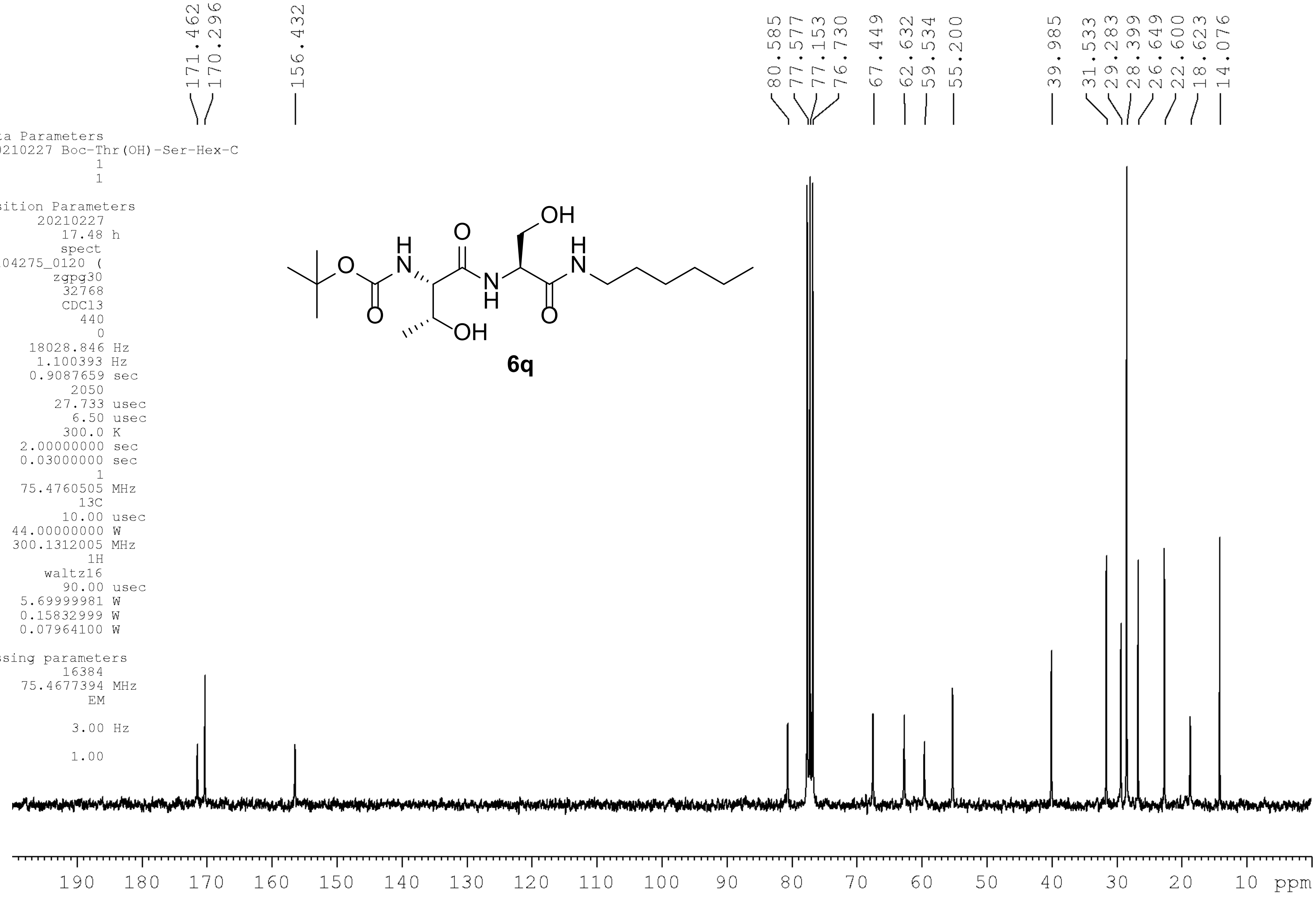


**6q**

$^{1}$H NMR Spectrum of **6r** (400 MHz, $CD_3OD$)

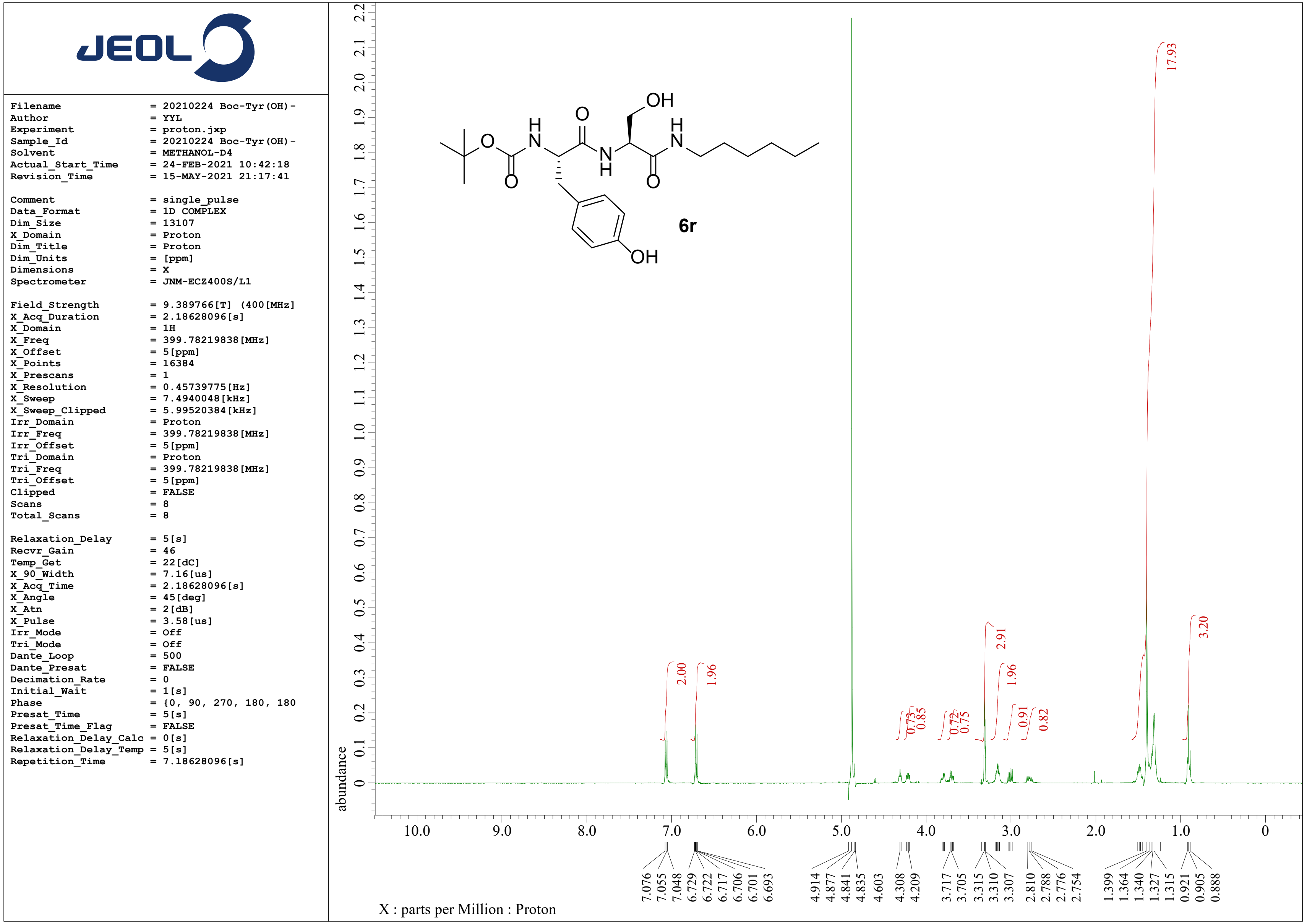

## $^{13}C\{^{1}H\}$ NMR Spectrum of **6r** (100 MHz, $CD_3OD$)

```
Filename                  = 20210225 Boc-Tyr(O
Author                    = yyl
Experiment                = carbon.jxp
Sample_Id                 = 20210225 Boc-Tyr(O
Solvent                   = METHANOL-D4
Actual_Start_Time         = 25-FEB-2021 11:26:
Revision_Time             = 15-MAY-2021 21:19:

Comment                   = single pulse decou
Data_Format               = 1D COMPLEX
Dim_Size                  = 26214
X_Domain                  = Carbon13
Dim_Title                 = Carbon13
Dim_Units                 = [ppm]
Dimensions                = X
Spectrometer              = JNM-ECZ400S/L1

Field_Strength            = 9.389766[T] (400[M
X_Acq_Duration            = 1.03809024[s]
X_Domain                  = 13C
X_Freq                    = 100.52530333[MHz]
X_Offset                  = 100[ppm]
X_Points                  = 32768
X_Prescans                = 4
X_Resolution              = 0.96330739[Hz]
X_Sweep                   = 31.56565657[kHz]
X_Sweep_Clipped           = 25.25252525[kHz]
Irr_Domain                = Proton
Irr_Freq                  = 399.78219838[MHz]
Irr_Offset                = 5[ppm]
Clipped                   = FALSE
Scans                     = 1024
Total_Scans               = 1024

Relaxation_Delay          = 2[s]
Recvr_Gain                = 50
Temp_Get                  = 21.9[dC]
X_90_Width                = 10.3[us]
X_Acq_Time                = 1.03809024[s]
X_Angle                   = 30[deg]
X_Atn                     = 7[dB]
X_Pulse                   = 3.43333333[us]
Irr_Atn_Dec               = 26.116[dB]
Irr_Atn_Dec_Calc          = 26.116[dB]
Irr_Atn_Dec_Default_Calc  = 26.116[dB]
Irr_Atn_Noe               = 26.116[dB]
Irr_Dec_Bandwidth_Hz      = 4.7826087[kHz]
Irr_Dec_Bandwidth_Ppm     = 11.96303566[ppm]
Irr_Dec_Freq              = 399.78219838[MHz]
Irr_Dec_Merit_Factor      = 2.2
Irr_Decoupling            = TRUE
Irr_Noe                   = TRUE
Irr_Noise                 = WALTZ
Irr_Offset_Default        = 5[ppm]
Irr_Pwidth                = 0.115[ms]
Irr_Pwidth_Default        = 0.115[ms]
Irr_Pwidth_Default_Calc   = 0.115[ms]
Irr_Pwidth_Temp1          = 0.115[ms]
Irr_Wurst                 = FALSE
Decimation_Rate           = 0
Initial_Wait              = 1[s]
Noe_Time                  = 2[s]
Noe_Time_Flag             = FALSE
Relaxation_Delay_Calc     = 0[s]
Relaxation_Delay_Temp     = 2[s]
Repetition_Time           = 3.03809024[s]
```

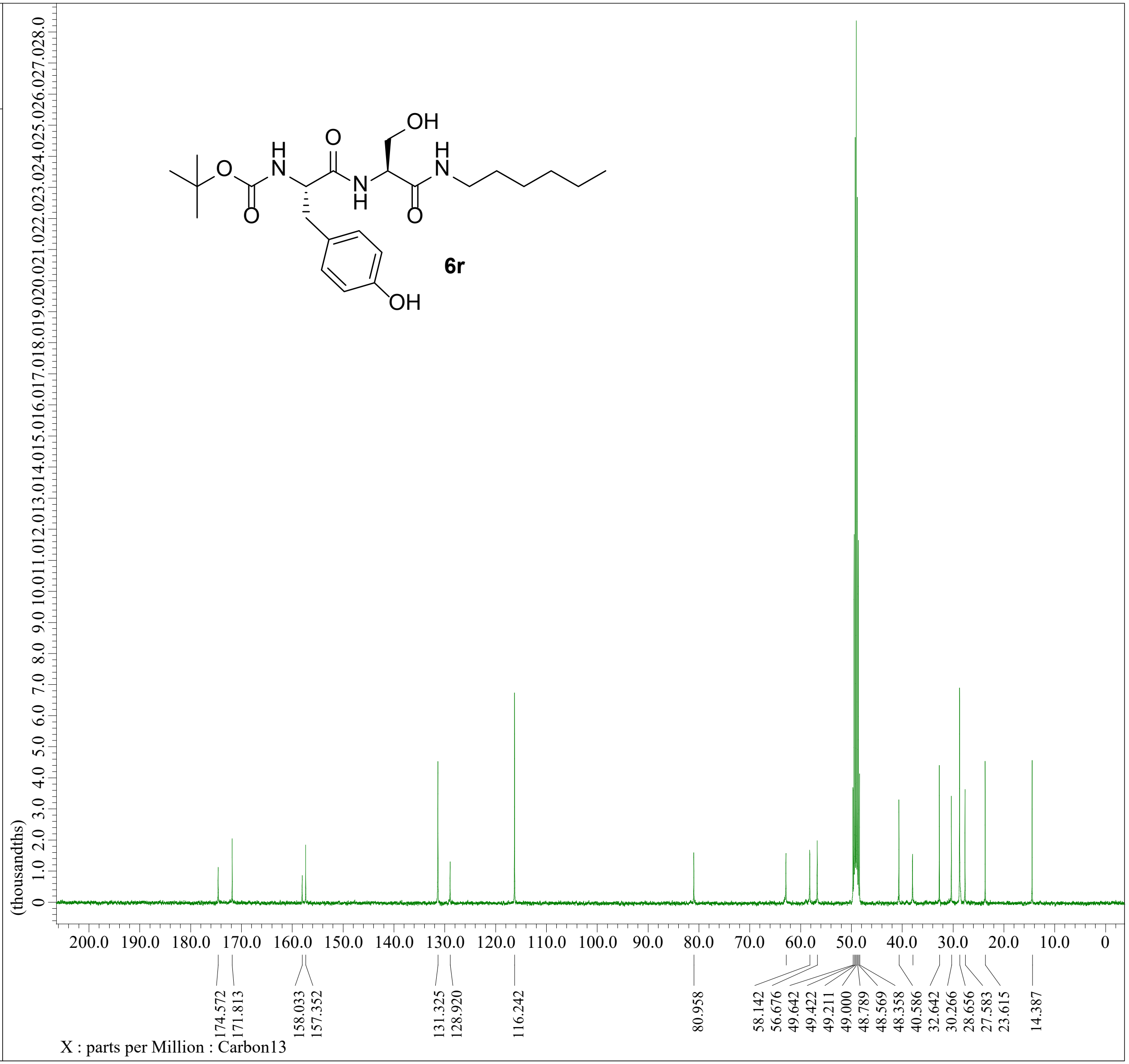

$^{1}$H NMR Spectrum of **6s** (300 MHz, $CDCl_3$)

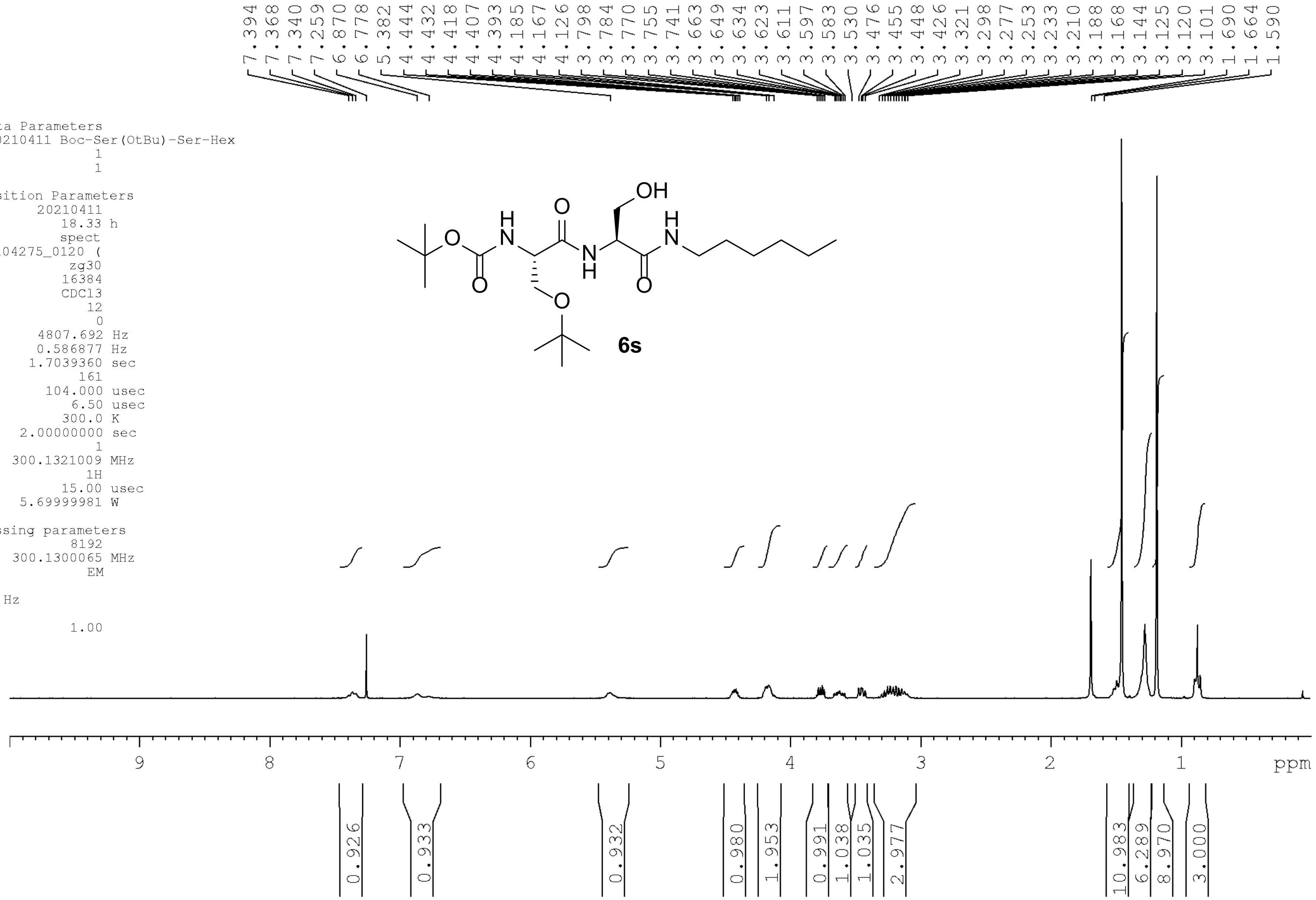


```
Current Data Parameters
NAME     20210411 Boc-Ser(OtBu)-Ser-Hex
EXPNO                   1
PROCNO                  1

F2 - Acquisition Parameters
Date_          20210411
Time              18.33 h
INSTRUM           spect
PROBHD   Z104275_0120 (
PULPROG            zg30
TD                16384
SOLVENT           CDCl3
NS                   12
DS                    0
SWH            4807.692 Hz
FIDRES         0.586877 Hz
AQ            1.7039360 sec
RG                  161
DW              104.000 usec
DE                 6.50 usec
TE                300.0 K
D1           2.00000000 sec
TD0                   1
SFO1        300.1321009 MHz
NUC1                 1H
P1                15.00 usec
PLW1         5.69999981 W

F2 - Processing parameters
SI                 8192
SF          300.1300065 MHz
WDW                  EM
SSB      0
LB       0 Hz
GB       0
PC                 1.00
```

**6s**

$^{13}C\{^{1}H\}$ NMR Spectrum of **6s** (75 MHz, $CDCl_3$)

```
Current Data Parameters
NAME      20210411 Boc-Ser(OtBu)-Ser-Hex-C
EXPNO                 1
PROCNO                1

F2 - Acquisition Parameters
Date_           20210411
Time               20.39 h
INSTRUM            spect
PROBHD   Z104275_0120 (
PULPROG          zgpg30
TD                32768
SOLVENT           CDCl3
NS                  480
DS                    0
SWH           18028.846 Hz
FIDRES         1.100393 Hz
AQ            0.9087659 sec
RG                 2050
DW               27.733 usec
DE                 6.50 usec
TE                300.0 K
D1           2.00000000 sec
D11          0.03000000 sec
TD0                   1
SFO1         75.4760505 MHz
NUC1                13C
P1                10.00 usec
PLW1        44.00000000 W
SFO2        300.1312005 MHz
NUC2                 1H
CPDPRG[2        waltz16
PCPD2             90.00 usec
PLW2         5.69999981 W
PLW12        0.15832999 W
PLW13        0.07964100 W

F2 - Processing parameters
SI                16384
SF           75.4677393 MHz
WDW                  EM
SSB      0
LB                 3.00 Hz
GB       0
PC                 1.00
```

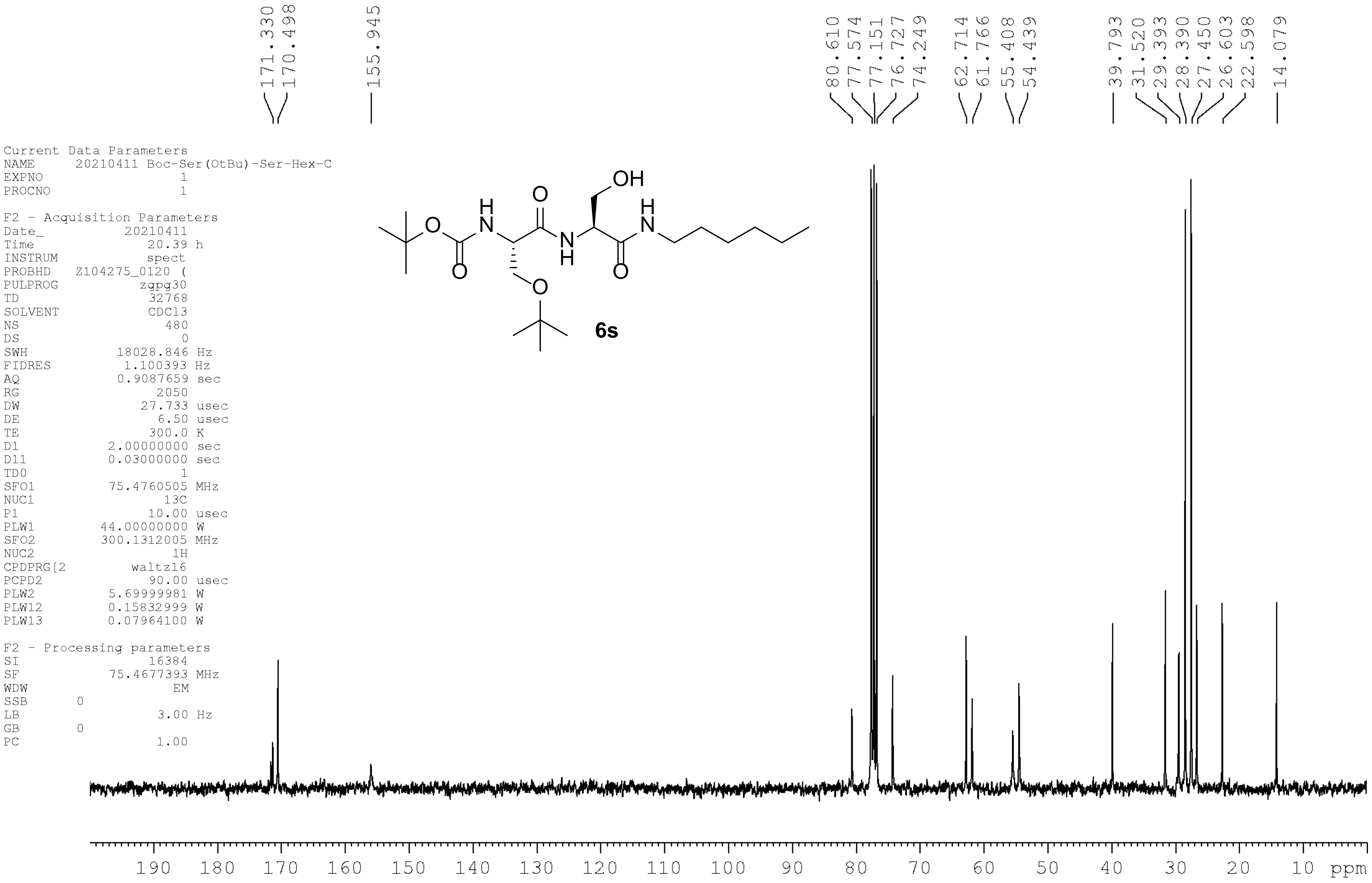

$^{1}$H NMR Spectrum of **6t** (300 MHz, $CDCl_3$)

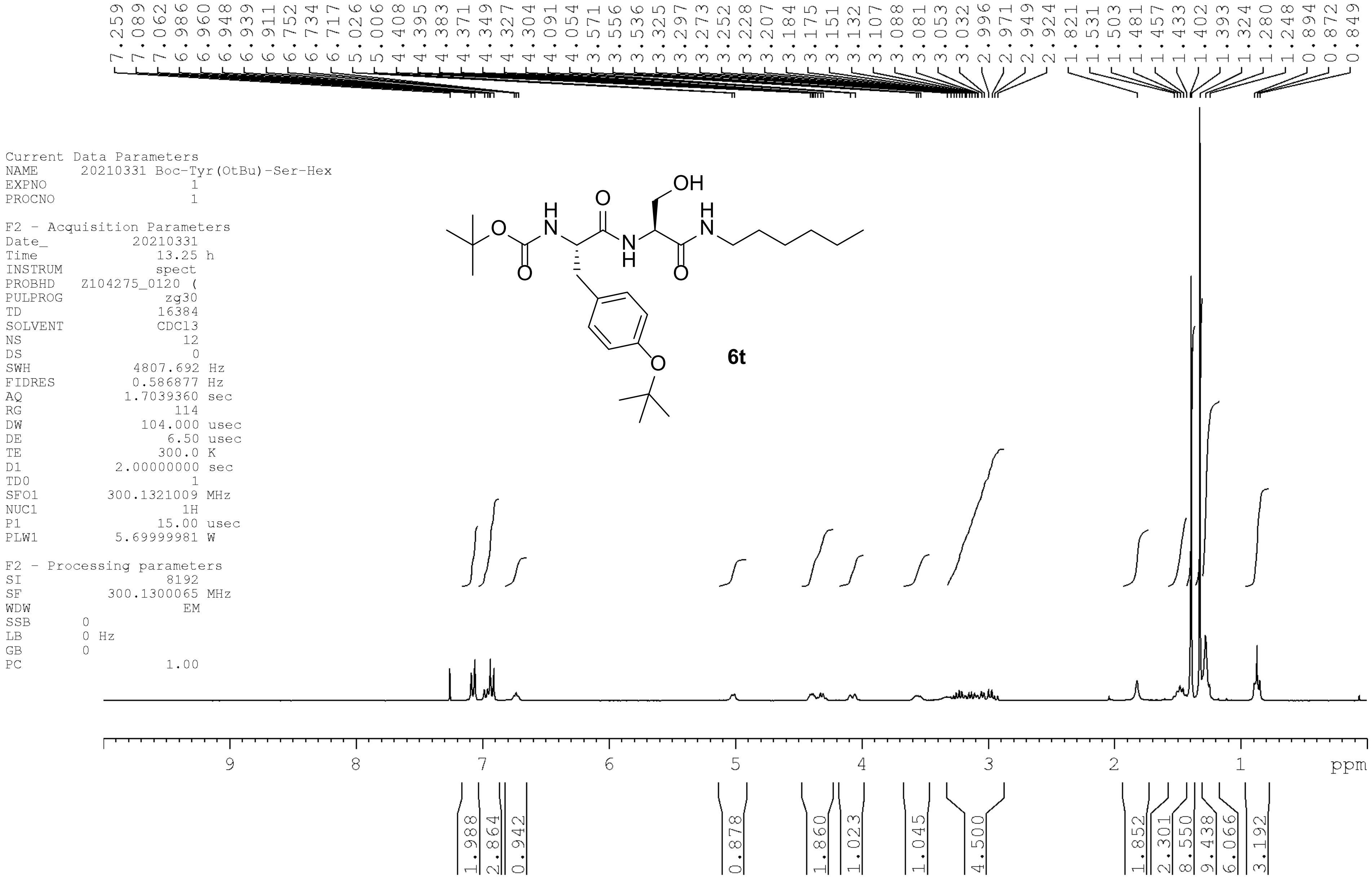

$^{13}C\{^{1}H\}$ NMR Spectrum of **6t** (75 MHz, $CDCl_3$)

```
Current Data Parameters
NAME     20210331 Boc-Try(OtBu)-Ser-Hex-C
EXPNO                 1
PROCNO                1

F2 - Acquisition Parameters
Date_          20210331
Time              17.05 h
INSTRUM           spect
PROBHD   Z104275_0120 (
PULPROG          zgpg30
TD                32768
SOLVENT           CDCl3
NS                  475
DS                    0
SWH           18028.846 Hz
FIDRES         1.100393 Hz
AQ            0.9087659 sec
RG                 2050
DW               27.733 usec
DE                 6.50 usec
TE                300.0 K
D1           2.00000000 sec
D11          0.03000000 sec
TD0                   1
SFO1         75.4760505 MHz
NUC1                13C
P1                10.00 usec
PLW1        44.00000000 W
SFO2        300.1312005 MHz
NUC2                 1H
CPDPRG[2        waltz16
PCPD2             90.00 usec
PLW2         5.69999981 W
PLW12        0.15832999 W
PLW13        0.07964100 W

F2 - Processing parameters
SI                16384
SF           75.4677405 MHz
WDW                  EM
SSB      0
LB                 3.00 Hz
GB       0
PC                 1.00
```

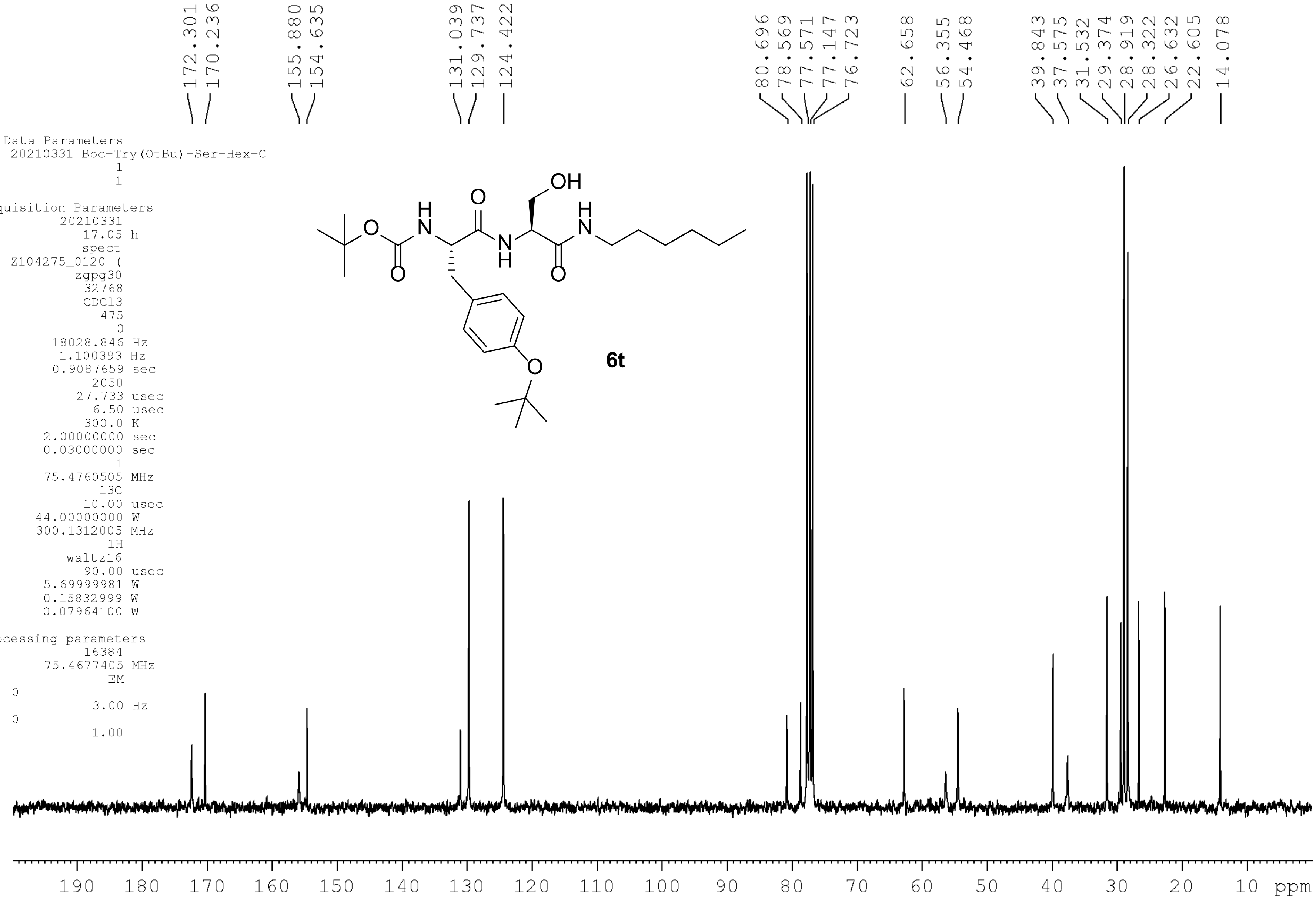


**6t**

$^{1}$H NMR Spectrum of **6u** (300 MHz, $CDCl_3$)

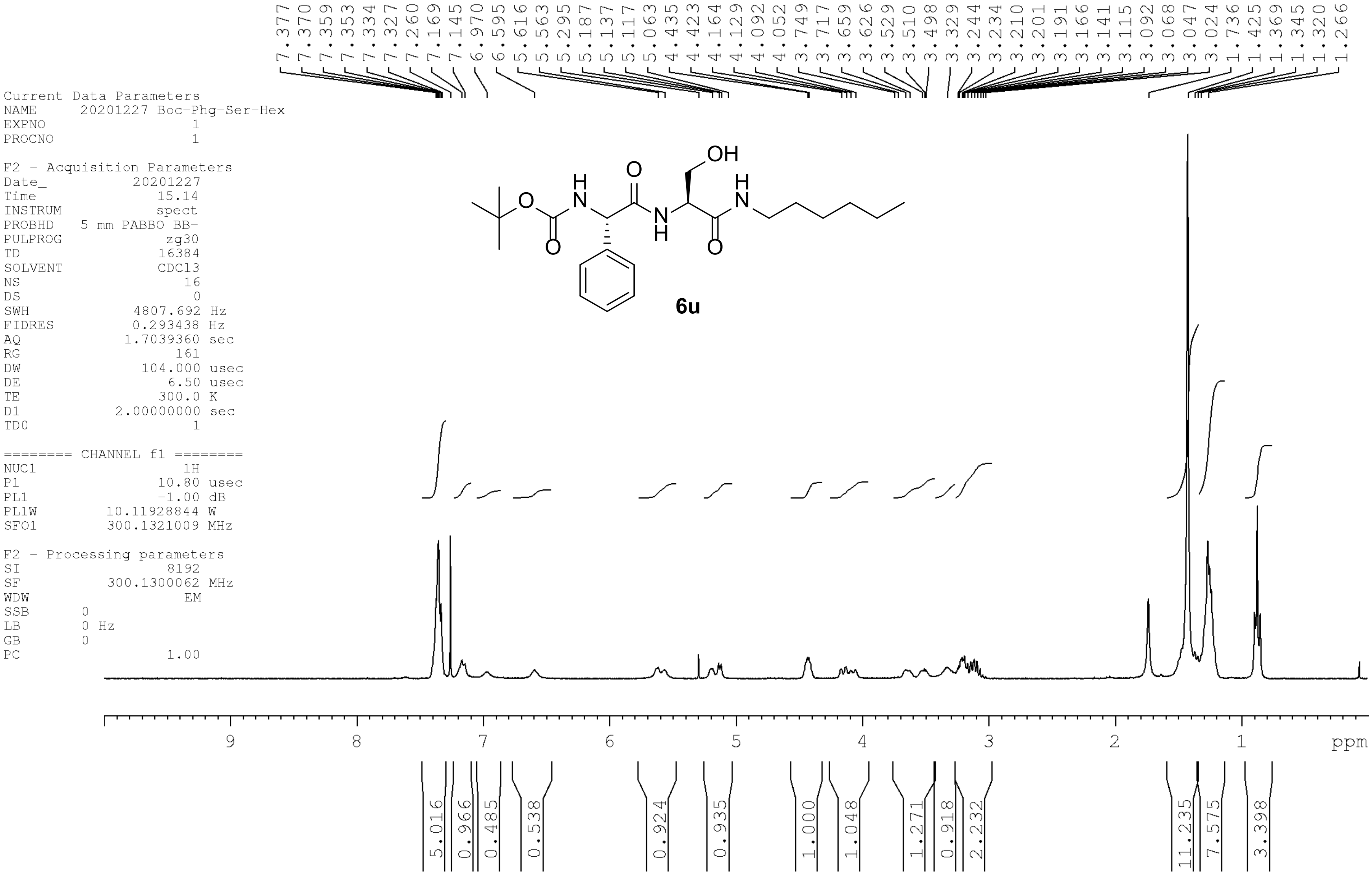

## $^{13}C\{^{1}H\}$ NMR Spectrum of **6u** (75 MHz, $CDCl_3$)

```
Current Data Parameters
NAME     20201226 Boc-Phg-Ser-Hex-C
EXPNO                     1
PROCNO                    1

F2 - Acquisition Parameters
Date_              20201226
Time                  17.25
INSTRUM               spect
PROBHD   5 mm PABBO BB-
PULPROG             zgpg30
TD                   32768
SOLVENT              CDCl3
NS                     401
DS                       0
SWH            18028.846 Hz
FIDRES          0.550197 Hz
AQ             0.9087659 sec
RG                    2050
DW                  27.733 usec
DE                    6.50 usec
TE                   300.0 K
D1              2.00000000 sec
D11             0.03000000 sec
TD0                      1

======== CHANNEL f1 ========
NUC1                   13C
P1                    9.50 usec
PL1                  -1.00 dB
PL1W           46.16925430 W
SFO1           75.4760505 MHz

======== CHANNEL f2 ========
CPDPRG[2           waltz16
NUC2                    1H
PCPD2                90.00 usec
PL2                   1.00 dB
PL12                 17.29 dB
PL13                 22.00 dB
PL2W            6.38483953 W
PL12W           0.15002026 W
PL13W           0.05071658 W
SFO2           300.1312005 MHz

F2 - Processing parameters
SI                   16384
SF              75.4677390 MHz
WDW                     EM
SSB       0
LB                    3.00 Hz
GB        0
PC                    1.00
```

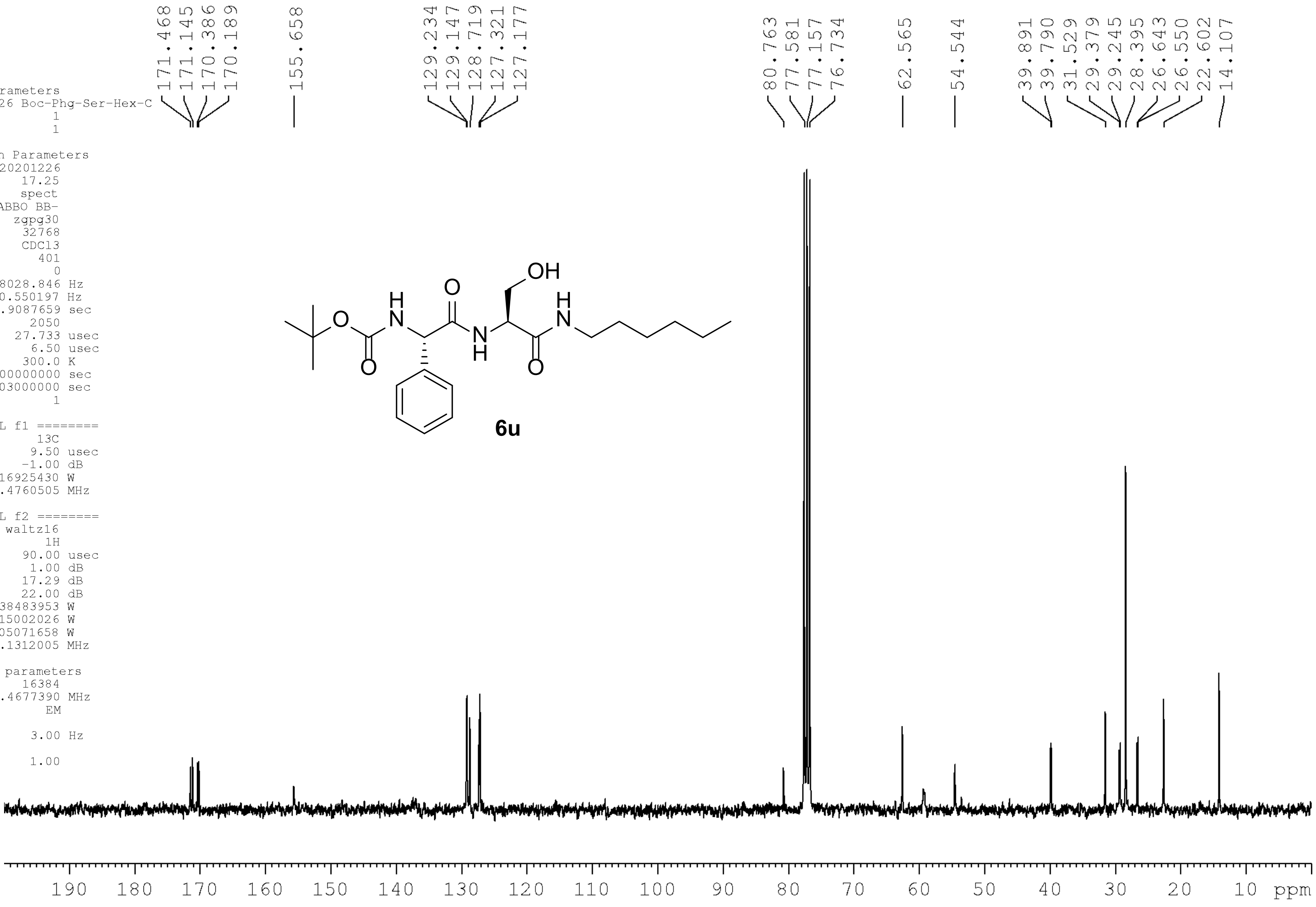

$^{1}$H NMR Spectrum of **6v** (300 MHz, $CDCl_3$)

```
Current Data Parameters
NAME       20210410 Boc-HomoSer-Ser-Hex
EXPNO                    2
PROCNO                   1

F2 - Acquisition Parameters
Date_           20210410
Time               16.07 h
INSTRUM            spect
PROBHD   Z104275_0120 (
PULPROG            zg30
TD                 16384
SOLVENT            CDCl3
NS                    12
DS                     0
SWH             4807.692 Hz
FIDRES          0.586877 Hz
AQ             1.7039360 sec
RG                   144
DW               104.000 usec
DE                  6.50 usec
TE                 300.0 K
D1            2.00000000 sec
TD0                    1
SFO1         300.1321009 MHz
NUC1                  1H
P1                 15.00 usec
PLW1          5.69999981 W

F2 - Processing parameters
SI                  8192
SF           300.1300065 MHz
WDW                   EM
SSB       0
LB        0 Hz
GB        0
PC                  1.00
```

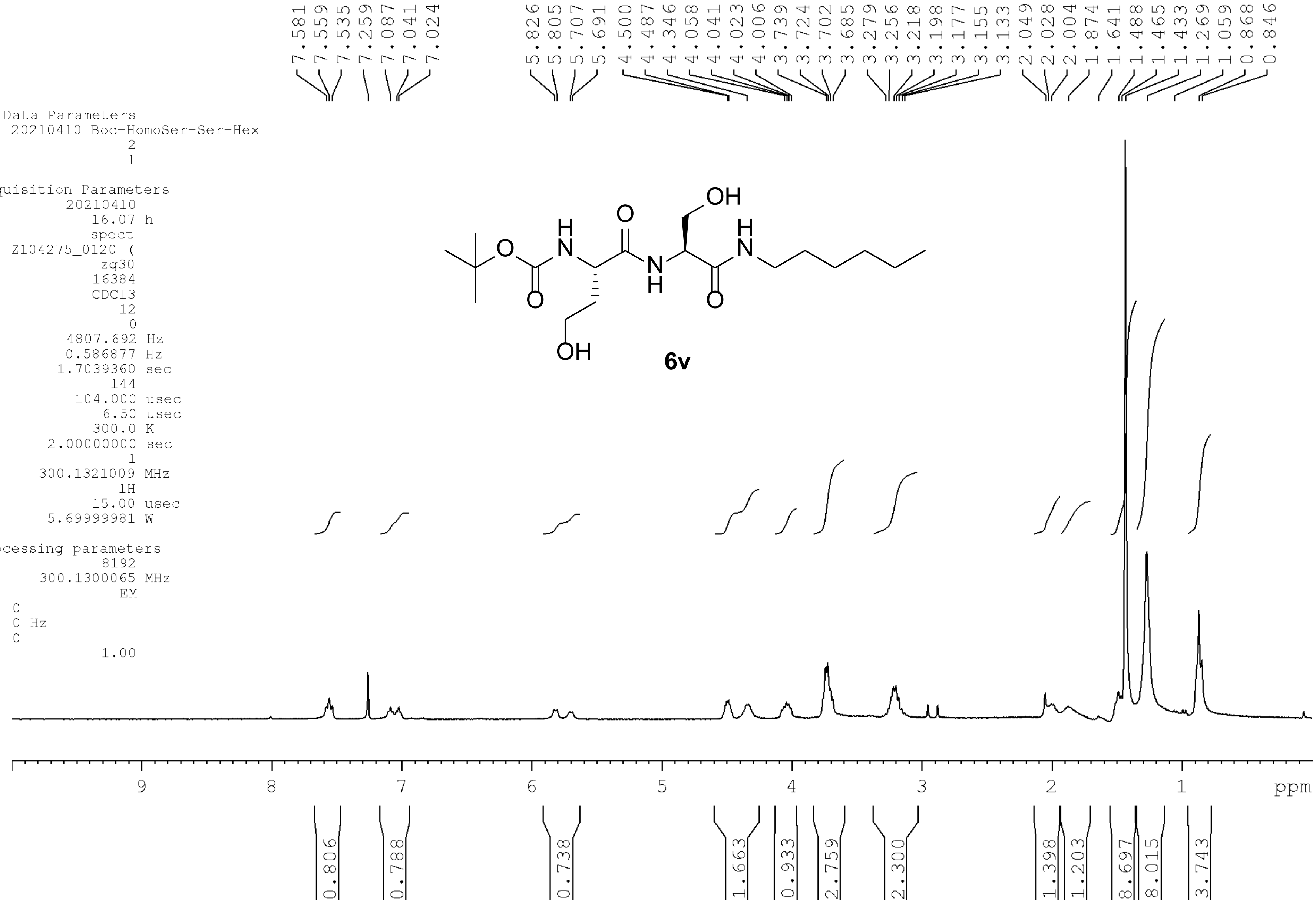

$^{13}C\{^1H\}$ NMR Spectrum of **6v** (75 MHz, $CDCl_3$)

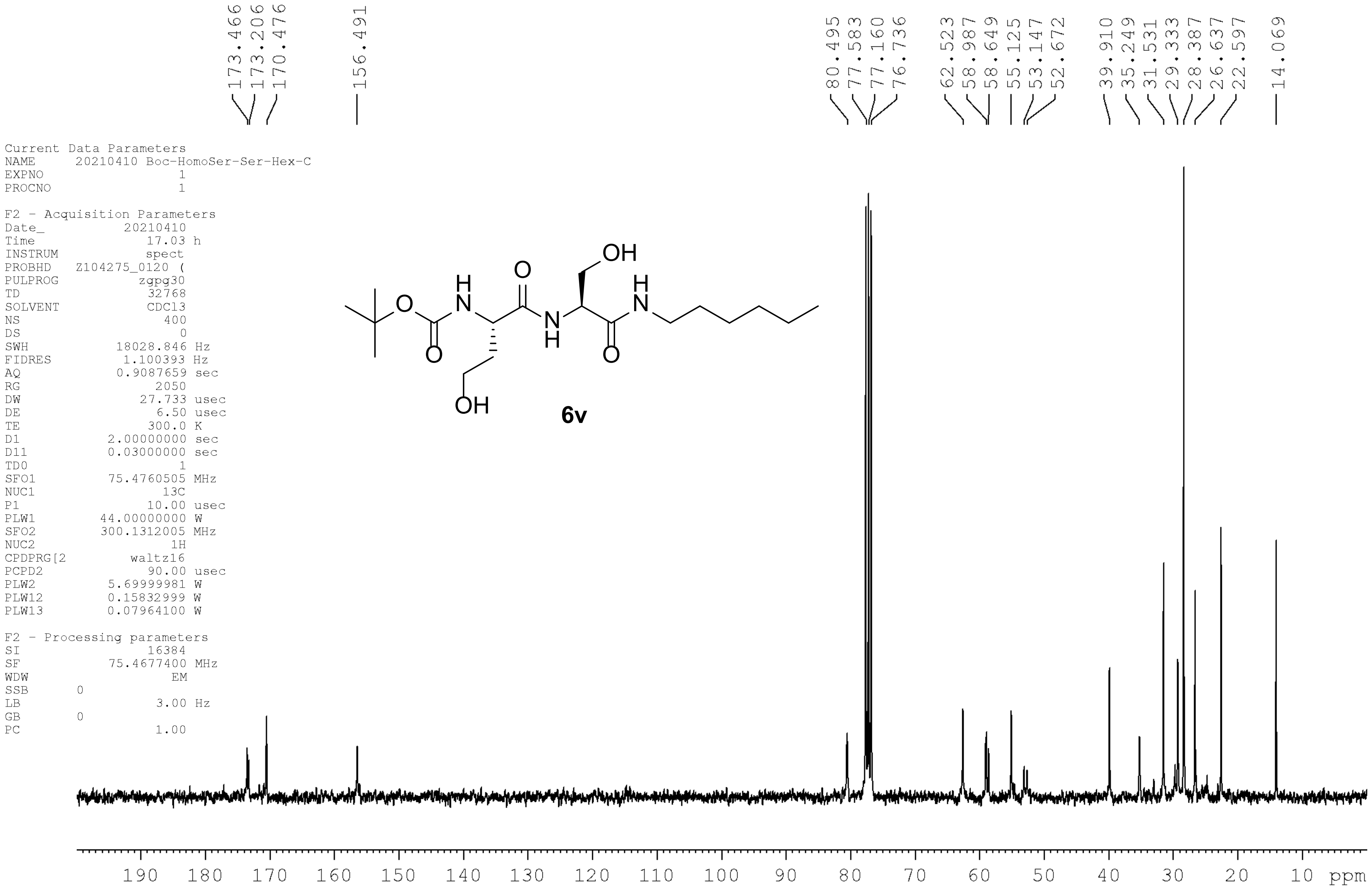

$^{1}$H NMR Spectrum of **6w** (300 MHz, $CDCl_3$)

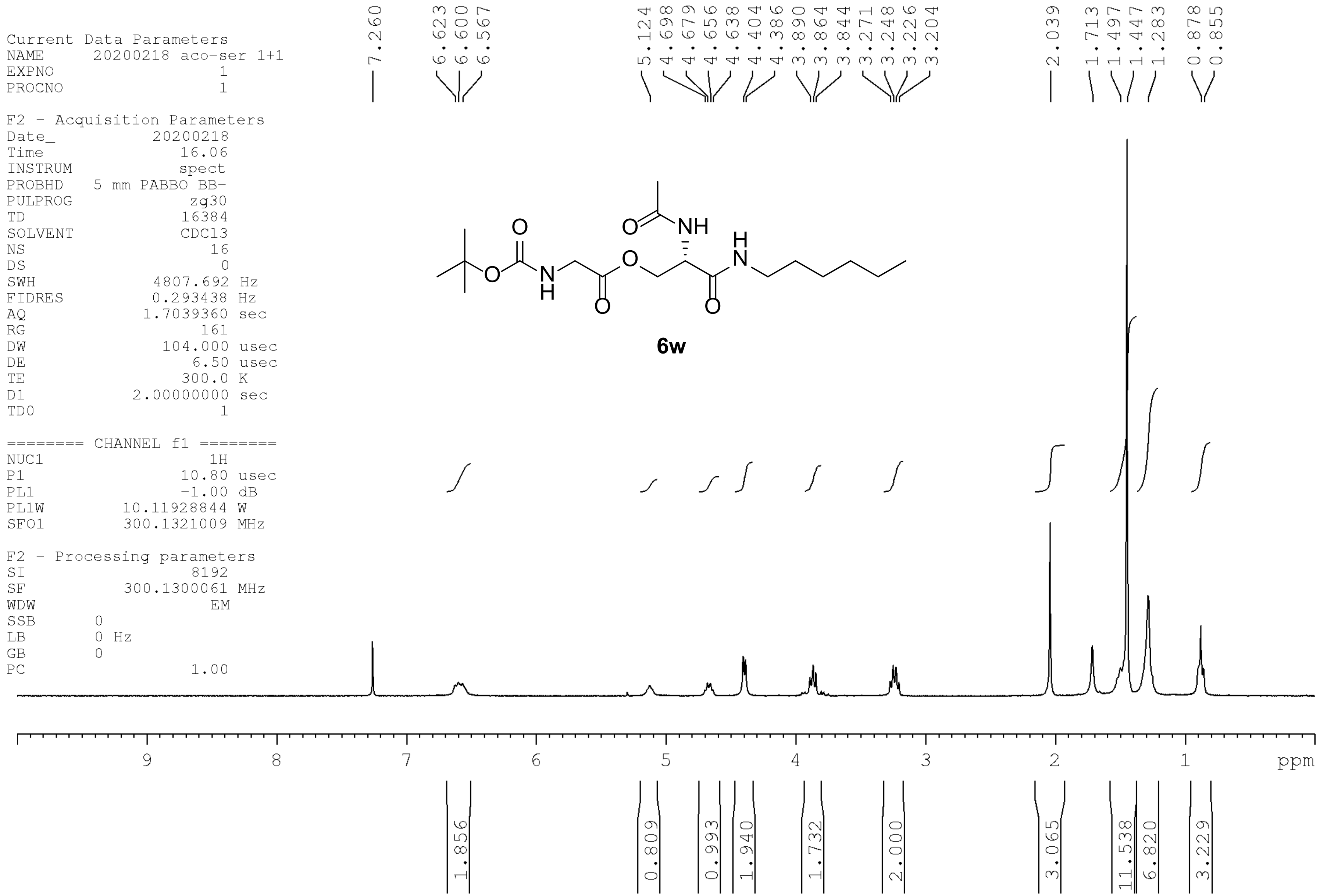

$^{13}C\{^{1}H\}$ NMR Spectrum of **6w** (100 MHz, $CDCl_3$)

```
Filename                  = 20200218Boc-Gly-Ac
Author                    = YYL
Experiment                = carbon.jxp
Sample_Id                 = 20200218Boc-Gly-Ac
Solvent                   = CHLOROFORM-D
Actual_Start_Time         = 19-FEB-2020 01:02:
Revision_Time             = 20-JUN-2021 19:47:

Comment                   = single pulse decou
Data_Format               = 1D COMPLEX
Dim_Size                  = 26214
X_Domain                  = Carbon13
Dim_Title                 = Carbon13
Dim_Units                 = [ppm]
Dimensions                = X
Spectrometer              = JNM-ECZ400S/L1

Field_Strength            = 9.389766[T] (400[M
X_Acq_Duration            = 1.03809024[s]
X_Domain                  = 13C
X_Freq                    = 100.52530333[MHz]
X_Offset                  = 100[ppm]
X_Points                  = 32768
X_Prescans                = 4
X_Resolution              = 0.96330739[Hz]
X_Sweep                   = 31.56565657[kHz]
X_Sweep_Clipped           = 25.25252525[kHz]
Irr_Domain                = Proton
Irr_Freq                  = 399.78219838[MHz]
Irr_Offset                = 5[ppm]
Clipped                   = TRUE
Scans                     = 1024
Total_Scans               = 1024

Relaxation_Delay          = 2[s]
Recvr_Gain                = 50
Temp_Get                  = 22.7[dC]
X_90_Width                = 10.369[us]
X_Acq_Time                = 1.03809024[s]
X_Angle                   = 30[deg]
X_Atn                     = 7[dB]
X_Pulse                   = 3.45633333[us]
Irr_Atn_Dec               = 25.678[dB]
Irr_Atn_Dec_Calc          = 25.678[dB]
Irr_Atn_Dec_Default_Calc  = 25.678[dB]
Irr_Atn_Noe               = 25.678[dB]
Irr_Dec_Bandwidth_Hz      = 4.7826087[kHz]
Irr_Dec_Bandwidth_Ppm     = 11.96303566[ppm]
Irr_Dec_Freq              = 399.78219838[MHz]
Irr_Dec_Merit_Factor      = 2.2
Irr_Decoupling            = TRUE
Irr_Noe                   = TRUE
Irr_Noise                 = WALTZ
Irr_Offset_Default        = 5[ppm]
Irr_Pwidth                = 0.115[ms]
Irr_Pwidth_Default        = 0.115[ms]
Irr_Pwidth_Default_Calc   = 0.115[ms]
Irr_Pwidth_Templ          = 0.115[ms]
Irr_Wurst                 = FALSE
Decimation_Rate           = 0
Initial_Wait              = 1[s]
Noe_Time                  = 2[s]
Noe_Time_Flag             = FALSE
Relaxation_Delay_Calc     = 0[s]
Relaxation_Delay_Temp     = 2[s]
Repetition_Time           = 3.03809024[s]
```

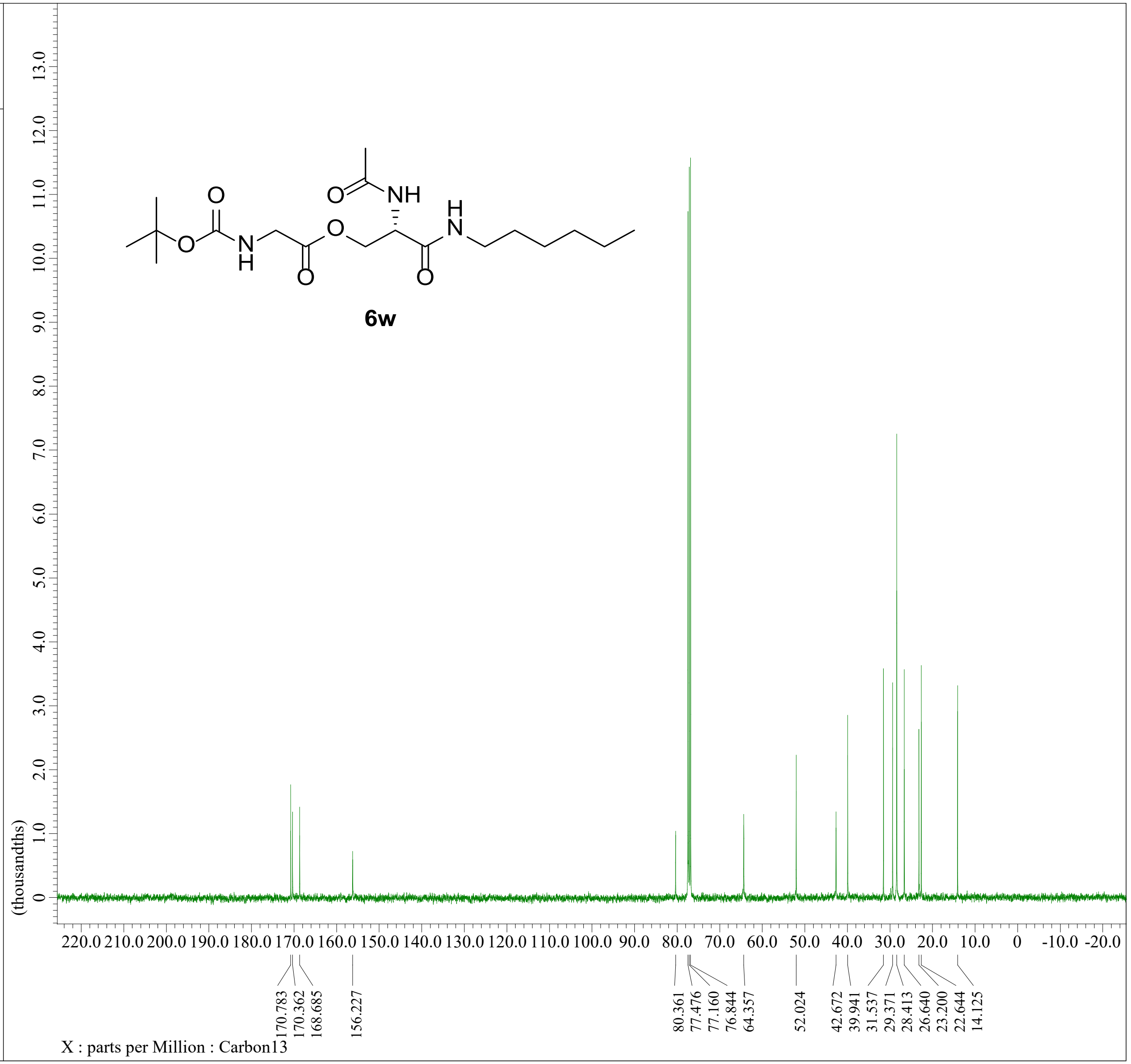

## $^1$H NMR Spectrum of **6ya, 6yb** (400 MHz, $CDCl_3$)

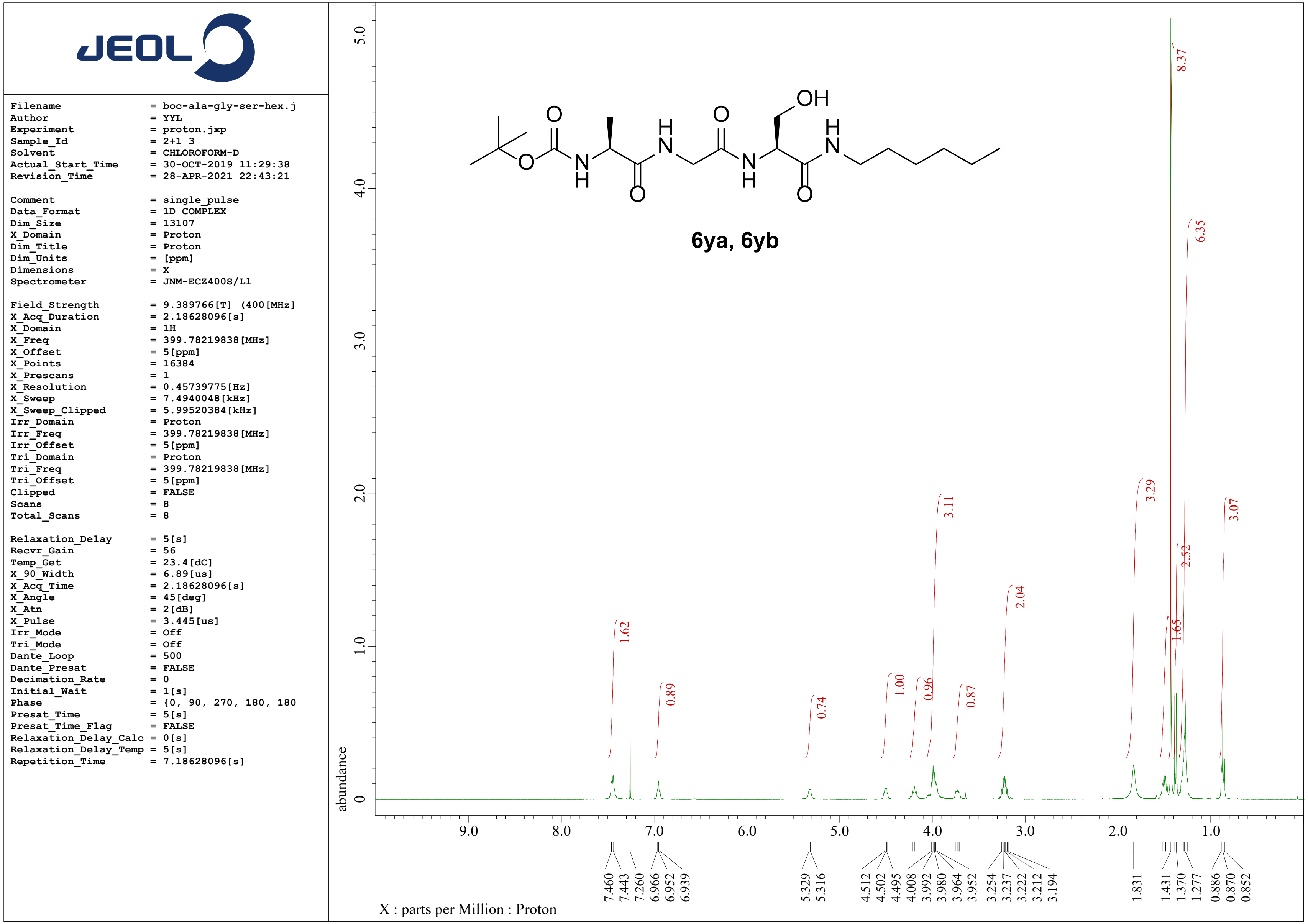

## $^{13}C\{^{1}H\}$ NMR Spectrum of **6ya, 6yb** (100 MHz, $CDCl_3$)

```
Filename                    = boc-ala-gly-ser-he
Author                      = YYL
Experiment                  = carbon.jxp
Sample_Id                   = 2+1
Solvent                     = CHLOROFORM-D
Actual_Start_Time           = 20-MAY-2021 12:27:
Revision_Time               = 24-MAY-2021 11:47:

Comment                     = single pulse decou
Data_Format                 = 1D COMPLEX
Dim_Size                    = 26214
X_Domain                    = Carbon13
Dim_Title                   = Carbon13
Dim_Units                   = [ppm]
Dimensions                  = X
Spectrometer                = JNM-ECZ400S/L1

Field_Strength              = 9.389766[T] (400[M
X_Acq_Duration              = 1.03809024[s]
X_Domain                    = 13C
X_Freq                      = 100.52530333[MHz]
X_Offset                    = 100[ppm]
X_Points                    = 32768
X_Prescans                  = 4
X_Resolution                = 0.96330739[Hz]
X_Sweep                     = 31.56565657[kHz]
X_Sweep_Clipped             = 25.25252525[kHz]
Irr_Domain                  = Proton
Irr_Freq                    = 399.78219838[MHz]
Irr_Offset                  = 5[ppm]
Clipped                     = FALSE
Scans                       = 512
Total_Scans                 = 512

Relaxation_Delay            = 2[s]
Recvr_Gain                  = 50
Temp_Get                    = 24.4[dC]
X_90_Width                  = 10.3[us]
X_Acq_Time                  = 1.03809024[s]
X_Angle                     = 30[deg]
X_Atn                       = 7[dB]
X_Pulse                     = 3.43333333[us]
Irr_Atn_Dec                 = 26.116[dB]
Irr_Atn_Dec_Calc            = 26.116[dB]
Irr_Atn_Dec_Default_Calc    = 26.116[dB]
Irr_Atn_Noe                 = 26.116[dB]
Irr_Dec_Bandwidth_Hz        = 4.7826087[kHz]
Irr_Dec_Bandwidth_Ppm       = 11.96303566[ppm]
Irr_Dec_Freq                = 399.78219838[MHz]
Irr_Dec_Merit_Factor        = 2.2
Irr_Decoupling              = TRUE
Irr_Noe                     = TRUE
Irr_Noise                   = WALTZ
Irr_Offset_Default          = 5[ppm]
Irr_Pwidth                  = 0.115[ms]
Irr_Pwidth_Default          = 0.115[ms]
Irr_Pwidth_Default_Calc     = 0.115[ms]
Irr_Pwidth_Temp1            = 0.115[ms]
Irr_Wurst                   = FALSE
Decimation_Rate             = 0
Initial_Wait                = 1[s]
Noe_Time                    = 2[s]
Noe_Time_Flag               = FALSE
Relaxation_Delay_Calc       = 0[s]
Relaxation_Delay_Temp       = 2[s]
Repetition_Time             = 3.03809024[s]
```

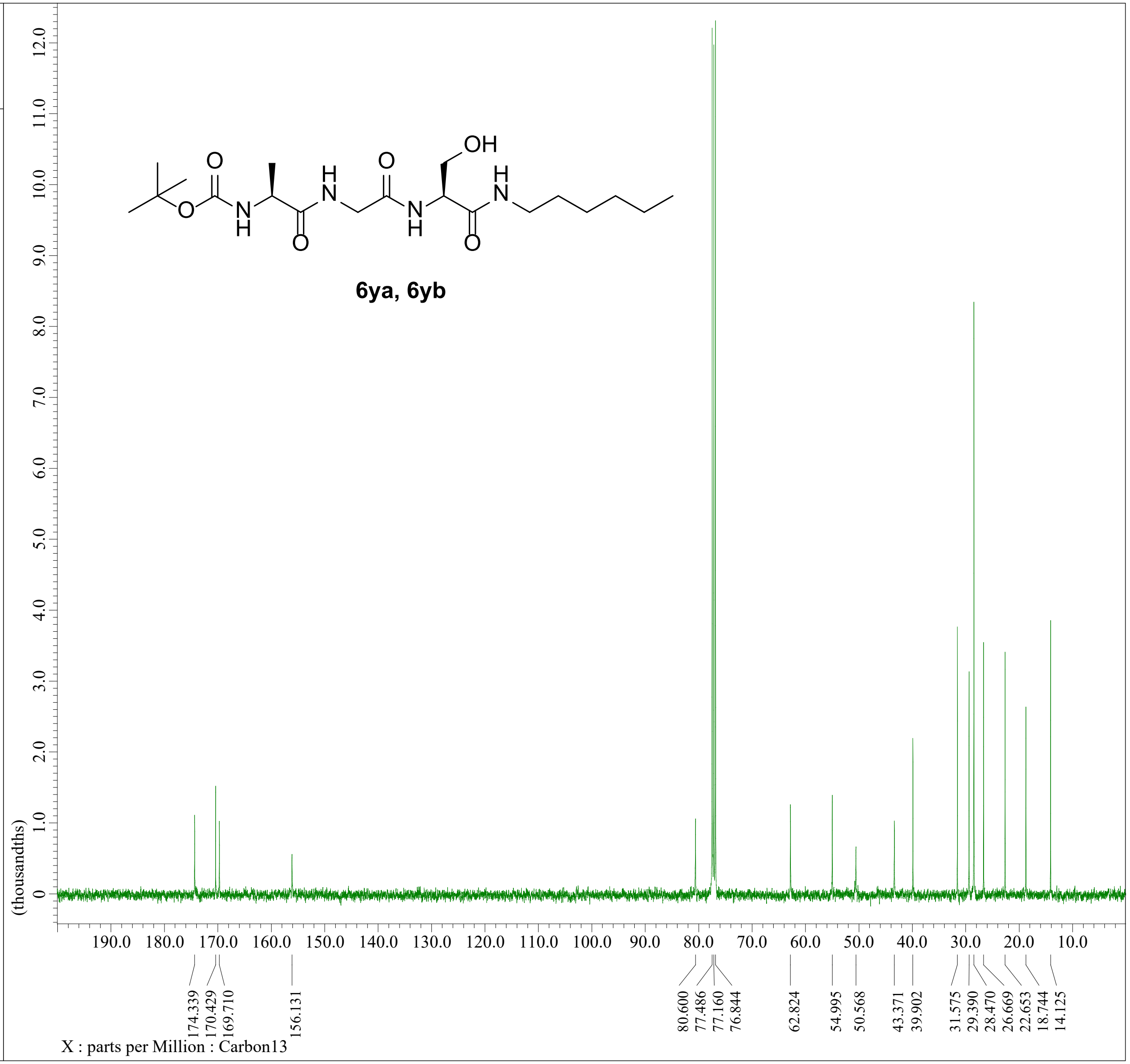

$^{1}$H NMR Spectrum of **6yc** (300 MHz, $CD_3OD$)

```
Current Data Parameters
NAME     20201120 3petide
EXPNO                   1
PROCNO                  1

F2 - Acquisition Parameters
Date_          20201120
Time              15.59
INSTRUM           spect
PROBHD   5 mm PABBO BB-
PULPROG            zg30
TD                16384
SOLVENT            MeOD
NS                   16
DS                    0
SWH            4807.692 Hz
FIDRES         0.293438 Hz
AQ            1.7039360 sec
RG                  101
DW              104.000 usec
DE                 6.50 usec
TE                300.0 K
D1           2.00000000 sec
TD0                   1

======== CHANNEL f1 ========
NUC1                 1H
P1                10.80 usec
PL1               -1.00 dB
PL1W        10.11928844 W
SFO1        300.1321009 MHz

F2 - Processing parameters
SI                 8192
SF          300.1300048 MHz
WDW                  EM
SSB                   0
LB                    0 Hz
GB                    0
PC                 1.00
```

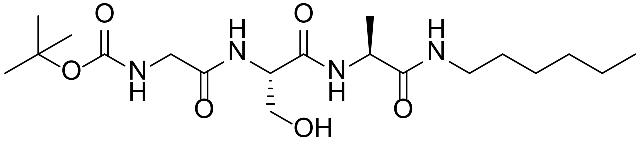

**6yc**

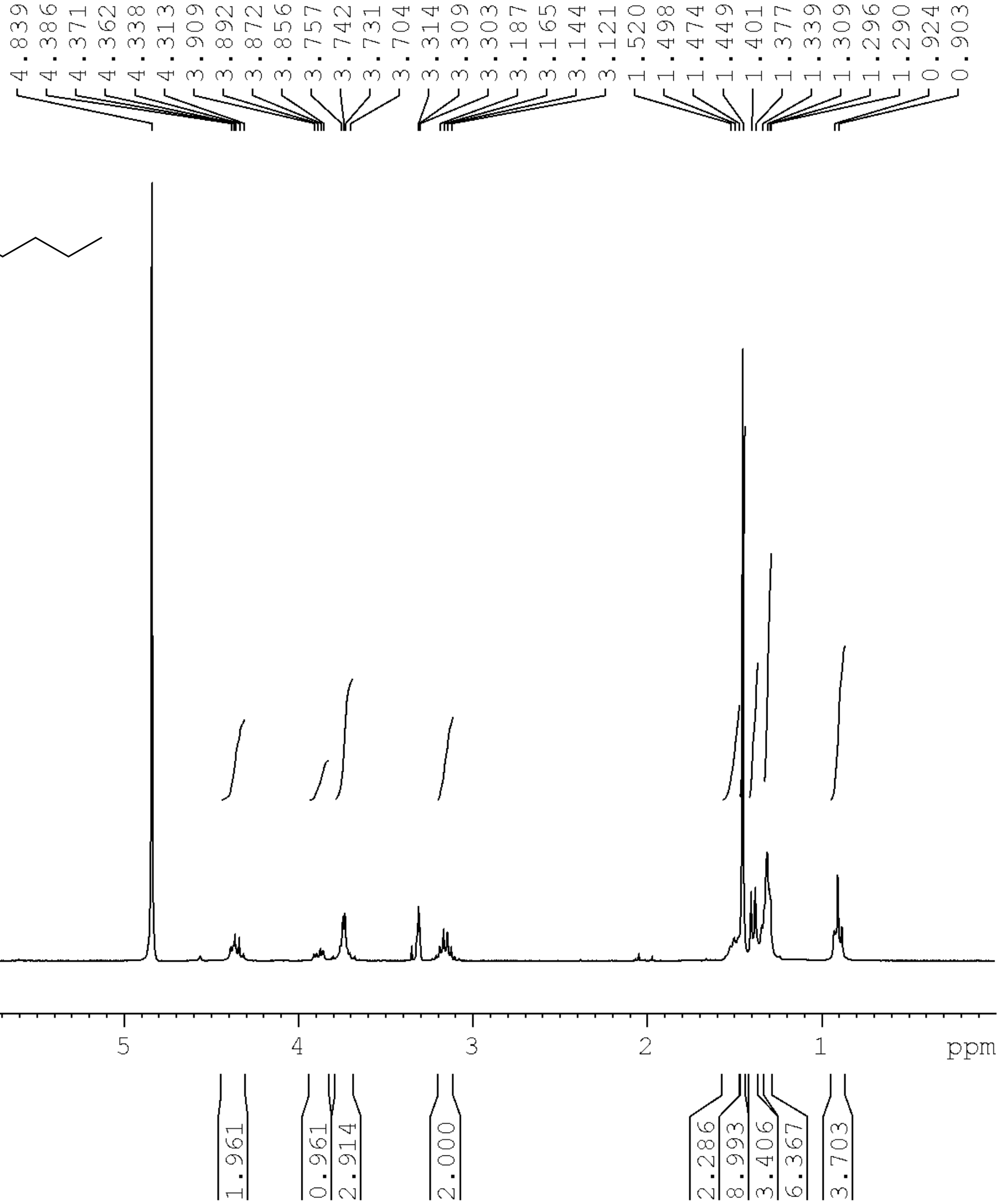

$^{13}C\{^{1}H\}$ NMR Spectrum of **6yc** (100 MHz, $CD_3OD$)

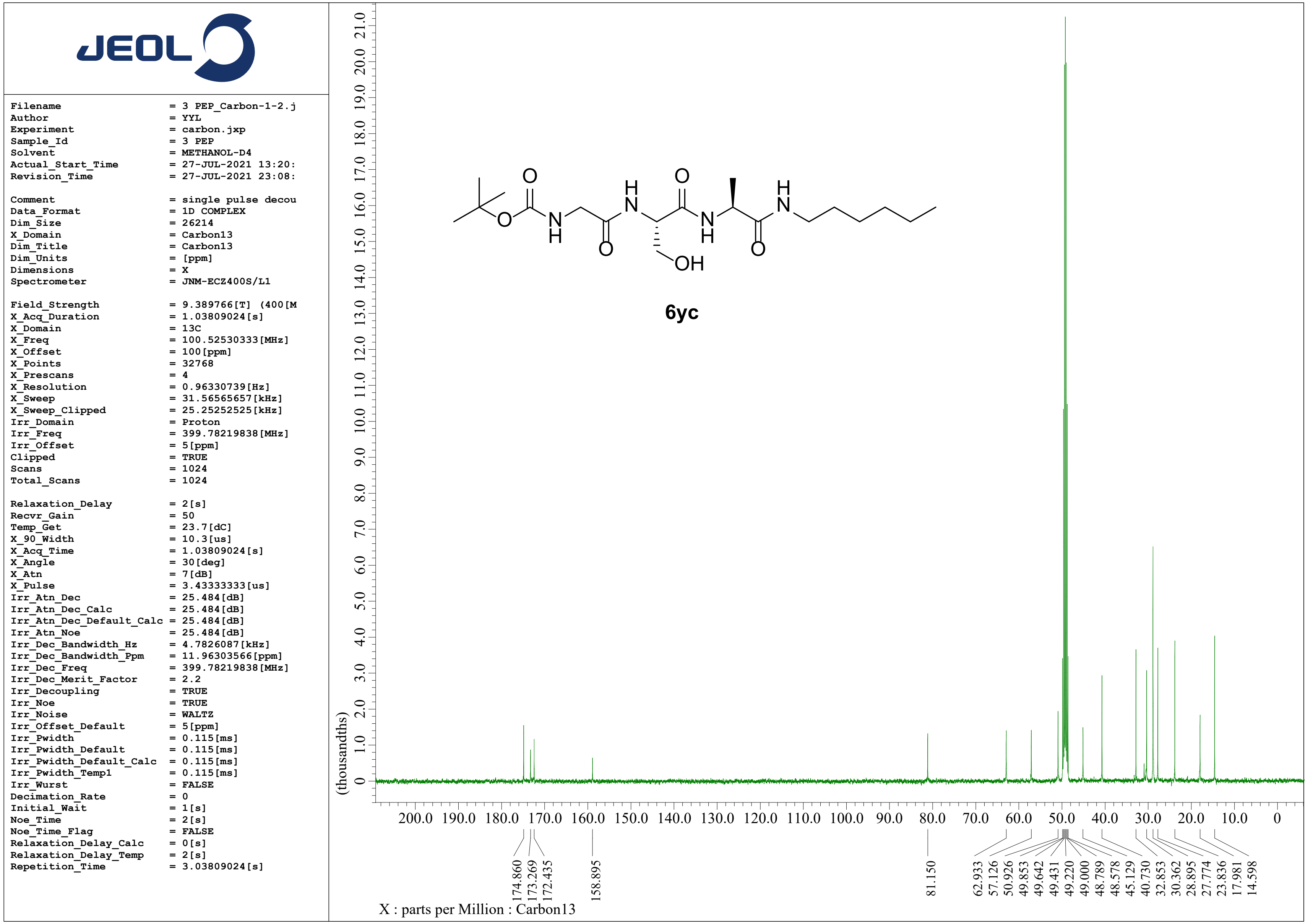

$^{1}$H NMR Spectrum of **6z** (400 MHz, $CD_3OD$)

| | |
|---|---|
| Filename | = 4 PEP_Proton-2-3.jdf |
| Author | = YYL |
| Experiment | = proton.jxp |
| Sample_Id | = 4 PEP |
| Solvent | = METHANOL-D4 |
| Actual_Start_Time | = 18-JUL-2021 16:34:03 |
| Revision_Time | = 29-JUL-2021 14:43:49 |
| Comment | = single_pulse |
| Data_Format | = 1D COMPLEX |
| Dim_Size | = 13107 |
| X_Domain | = Proton |
| Dim_Title | = Proton |
| Dim_Units | = [ppm] |
| Dimensions | = X |
| Spectrometer | = JNM-ECZ400S/L1 |
| Field_Strength | = 9.389766[T] (400[MHz] |
| X_Acq_Duration | = 2.18628096[s] |
| X_Domain | = 1H |
| X_Freq | = 399.78219838[MHz] |
| X_Offset | = 5[ppm] |
| X_Points | = 16384 |
| X_Prescans | = 1 |
| X_Resolution | = 0.45739775[Hz] |
| X_Sweep | = 7.4940048[kHz] |
| X_Sweep_Clipped | = 5.99520384[kHz] |
| Irr_Domain | = Proton |
| Irr_Freq | = 399.78219838[MHz] |
| Irr_Offset | = 5[ppm] |
| Tri_Domain | = Proton |
| Tri_Freq | = 399.78219838[MHz] |
| Tri_Offset | = 5[ppm] |
| Clipped | = FALSE |
| Scans | = 8 |
| Total_Scans | = 8 |
| Relaxation_Delay | = 5[s] |
| Recvr_Gain | = 36 |
| Temp_Get | = 24[dC] |
| X_90_Width | = 7.7[us] |
| X_Acq_Time | = 2.18628096[s] |
| X_Angle | = 45[deg] |
| X_Atn | = 2[dB] |
| X_Pulse | = 3.85[us] |
| Irr_Mode | = Off |
| Tri_Mode | = Off |
| Dante_Loop | = 500 |
| Dante_Presat | = FALSE |
| Decimation_Rate | = 0 |
| Initial_Wait | = 1[s] |
| Phase | = {0, 90, 270, 180, 180 |
| Presat_Time | = 5[s] |
| Presat_Time_Flag | = FALSE |
| Relaxation_Delay_Calc | = 0[s] |
| Relaxation_Delay_Temp | = 5[s] |
| Repetition_Time | = 7.18628096[s] |

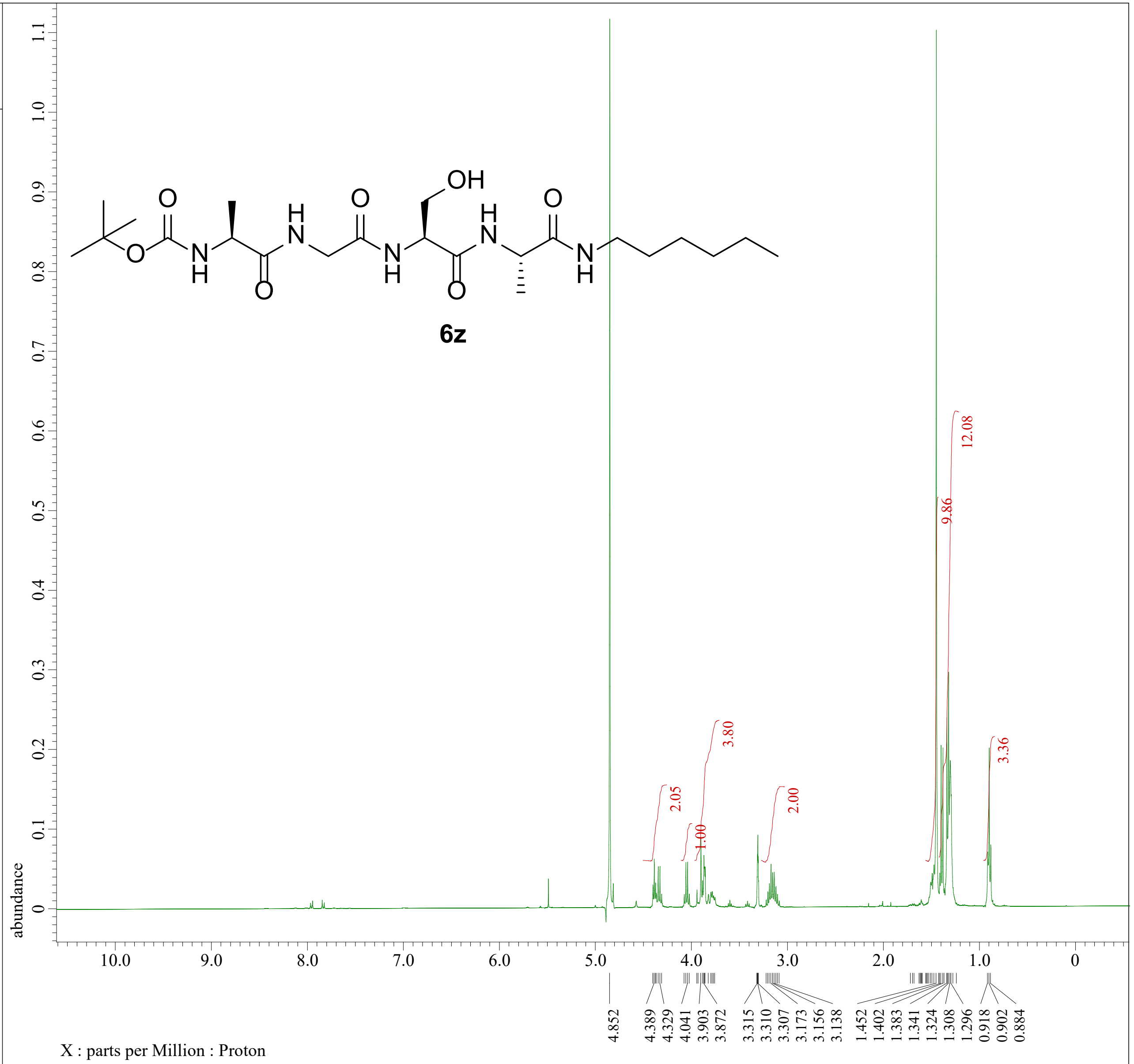

$^{13}C\{^{1}H\}$ NMR Spectrum of **6z** (100 MHz, $CD_3OD$)

JEOL

```
Filename                     = 20210731 4peptide-
Author                       = YYL
Experiment                   = carbon.jxp
Sample_Id                    = 20210731 4peptide-
Solvent                      = METHANOL-D4
Actual_Start_Time            = 31-JUL-2021 11:10:
Revision_Time                =  6-AUG-2021 13:53:

Comment                      = single pulse decou
Data_Format                  = 1D COMPLEX
Dim_Size                     = 26214
X_Domain                     = Carbon13
Dim_Title                    = Carbon13
Dim_Units                    = [ppm]
Dimensions                   = X
Spectrometer                 = JNM-ECZ400S/L1

Field_Strength               = 9.389766[T] (400[M
X_Acq_Duration               = 1.03809024[s]
X_Domain                     = 13C
X_Freq                       = 100.52530333[MHz]
X_Offset                     = 100[ppm]
X_Points                     = 32768
X_Prescans                   = 4
X_Resolution                 = 0.96330739[Hz]
X_Sweep                      = 31.56565657[kHz]
X_Sweep_Clipped              = 25.25252525[kHz]
Irr_Domain                   = Proton
Irr_Freq                     = 399.78219838[MHz]
Irr_Offset                   = 5[ppm]
Clipped                      = FALSE
Scans                        = 2048
Total_Scans                  = 2048

Relaxation_Delay             = 2[s]
Recvr_Gain                   = 50
Temp_Get                     = 23.4[dC]
X_90_Width                   = 10.3[us]
X_Acq_Time                   = 1.03809024[s]
X_Angle                      = 30[deg]
X_Atn                        = 7[dB]
X_Pulse                      = 3.43333333[us]
Irr_Atn_Dec                  = 25.484[dB]
Irr_Atn_Dec_Calc             = 25.484[dB]
Irr_Atn_Dec_Default_Calc     = 25.484[dB]
Irr_Atn_Noe                  = 25.484[dB]
Irr_Dec_Bandwidth_Hz         = 4.7826087[kHz]
Irr_Dec_Bandwidth_Ppm        = 11.96303566[ppm]
Irr_Dec_Freq                 = 399.78219838[MHz]
Irr_Dec_Merit_Factor         = 2.2
Irr_Decoupling               = TRUE
Irr_Noe                      = TRUE
Irr_Noise                    = WALTZ
Irr_Offset_Default           = 5[ppm]
Irr_Pwidth                   = 0.115[ms]
Irr_Pwidth_Default           = 0.115[ms]
Irr_Pwidth_Default_Calc      = 0.115[ms]
Irr_Pwidth_Templ             = 0.115[ms]
Irr_Wurst                    = FALSE
Decimation_Rate              = 0
Initial_Wait                 = 1[s]
Noe_Time                     = 2[s]
Noe_Time_Flag                = FALSE
Relaxation_Delay_Calc        = 0[s]
Relaxation_Delay_Temp        = 2[s]
Repetition_Time              = 3.03809024[s]
```

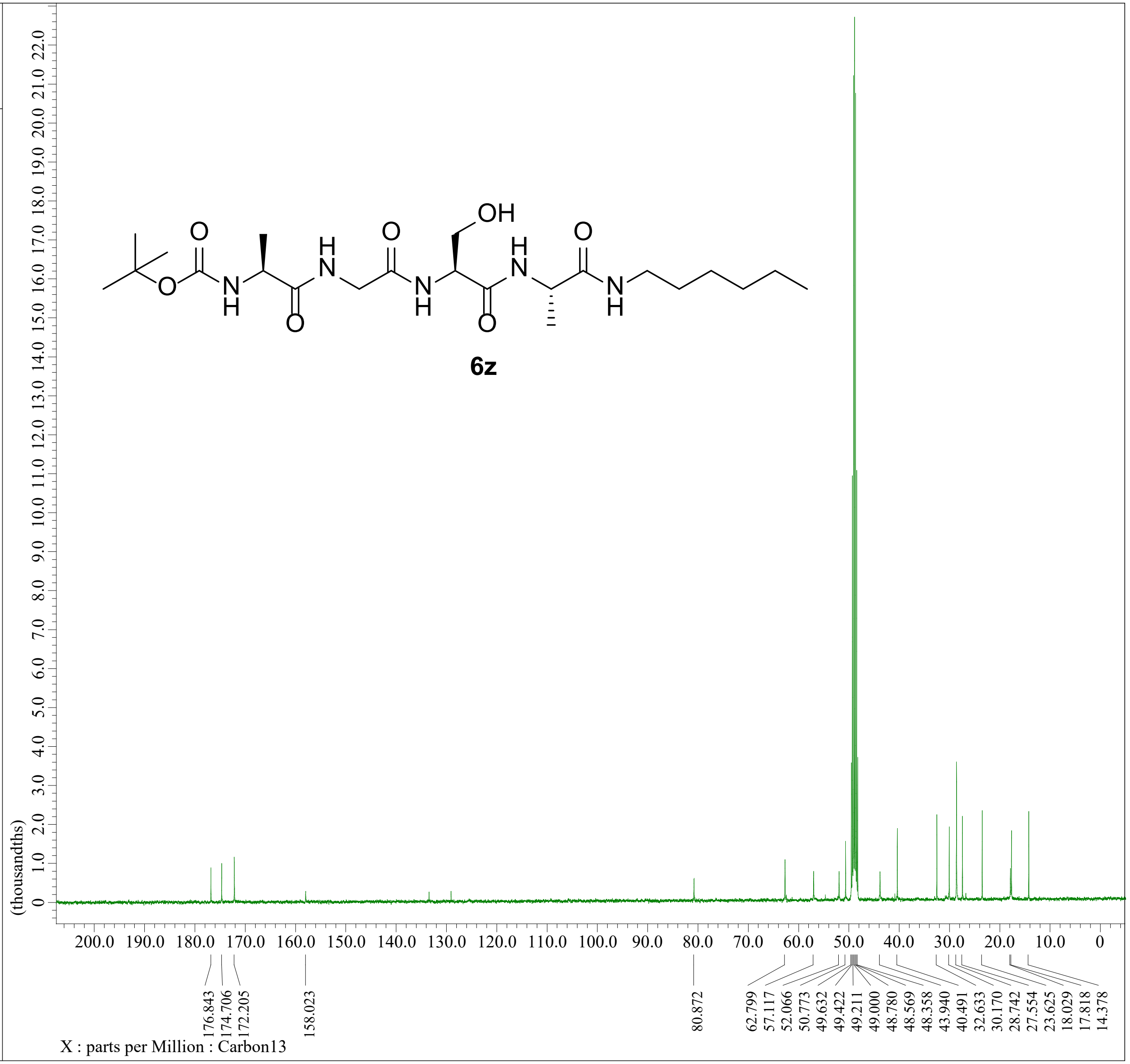

$^{1}$H NMR Spectrum of **7a** (300 MHz, $CDCl_3$)

```
Current Data Parameters
NAME     20210519 no2-ome
EXPNO                   1
PROCNO                  1

F2 - Acquisition Parameters
Date_          20210519
Time              13.28 h
INSTRUM           spect
PROBHD   Z104275_0120 (
PULPROG            zg30
TD                16384
SOLVENT           CDCl3
NS                   16
DS                    0
SWH            4807.692 Hz
FIDRES         0.586877 Hz
AQ            1.7039360 sec
RG                  256
DW              104.000 usec
DE                 6.50 usec
TE                300.0 K
D1           2.00000000 sec
TD0                   1
SFO1        300.1321009 MHz
NUC1                 1H
P1                15.00 usec
PLW1         5.69999981 W

F2 - Processing parameters
SI                 8192
SF          300.1300077 MHz
WDW                  EM
SSB                   0
LB                    0 Hz
GB                    0
PC                 1.00
```

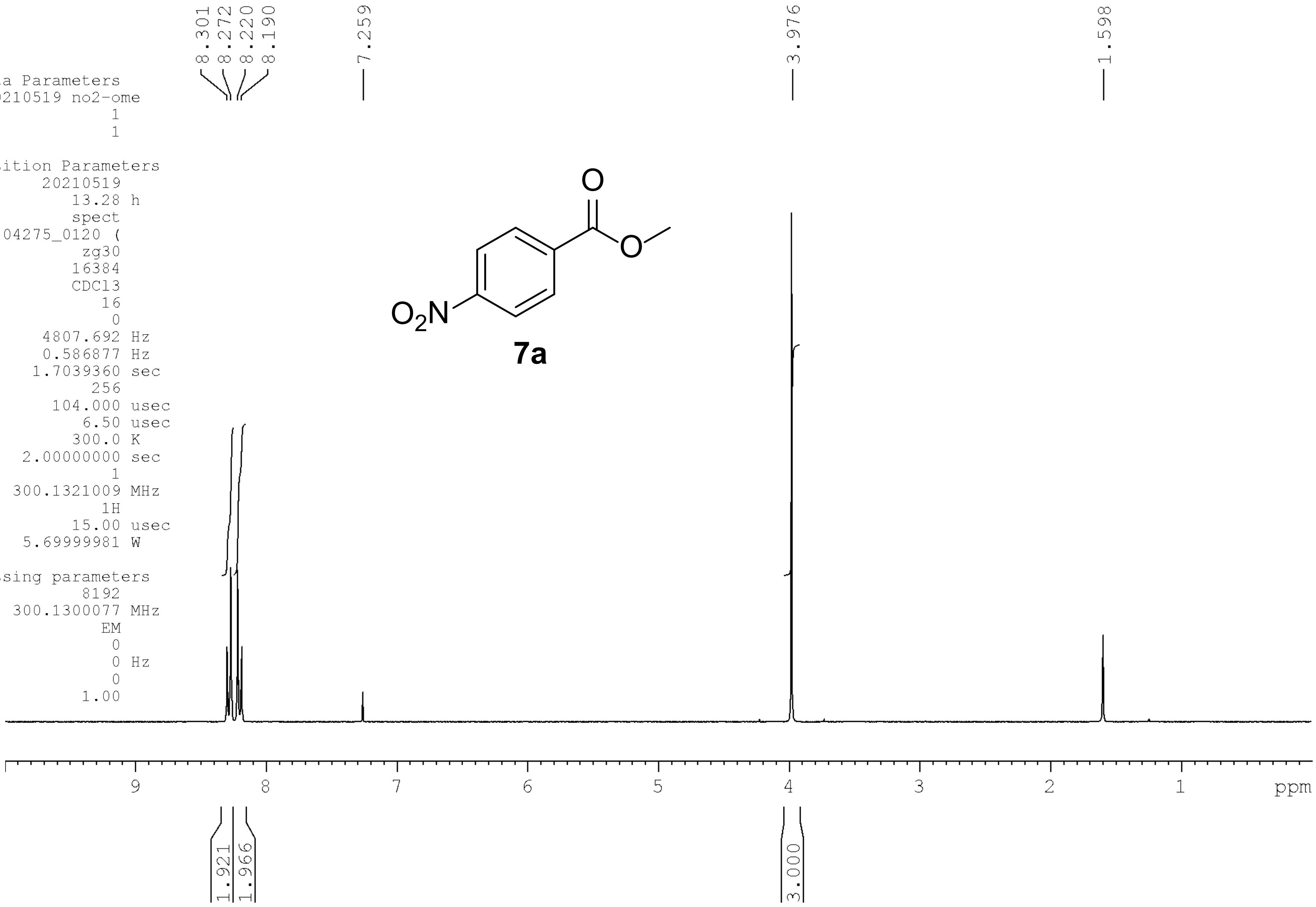

$^{1}$H NMR Spectrum of **7c** (300 MHz, $CDCl_3$)

```
Current Data Parameters
NAME      20200827  i-ome
EXPNO                 1
PROCNO                1

F2 - Acquisition Parameters
Date_          20200827
Time              11.11
INSTRUM           spect
PROBHD   5 mm PABBO BB-
PULPROG            zg30
TD                16384
SOLVENT           CDCl3
NS                   16
DS                    0
SWH            4807.692 Hz
FIDRES         0.293438 Hz
AQ            1.7039360 sec
RG                  228
DW              104.000 usec
DE                 6.50 usec
TE                300.0 K
D1           2.00000000 sec
TD0                   1

======== CHANNEL f1 ========
NUC1                 1H
P1                10.80 usec
PL1               -1.00 dB
PL1W        10.11928844 W
SFO1        300.1321009 MHz

F2 - Processing parameters
SI                 8192
SF          300.1300065 MHz
WDW                  EM
SSB                   0
LB                    0 Hz
GB                    0
PC                 1.00
```

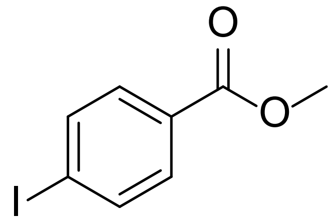


**7c**

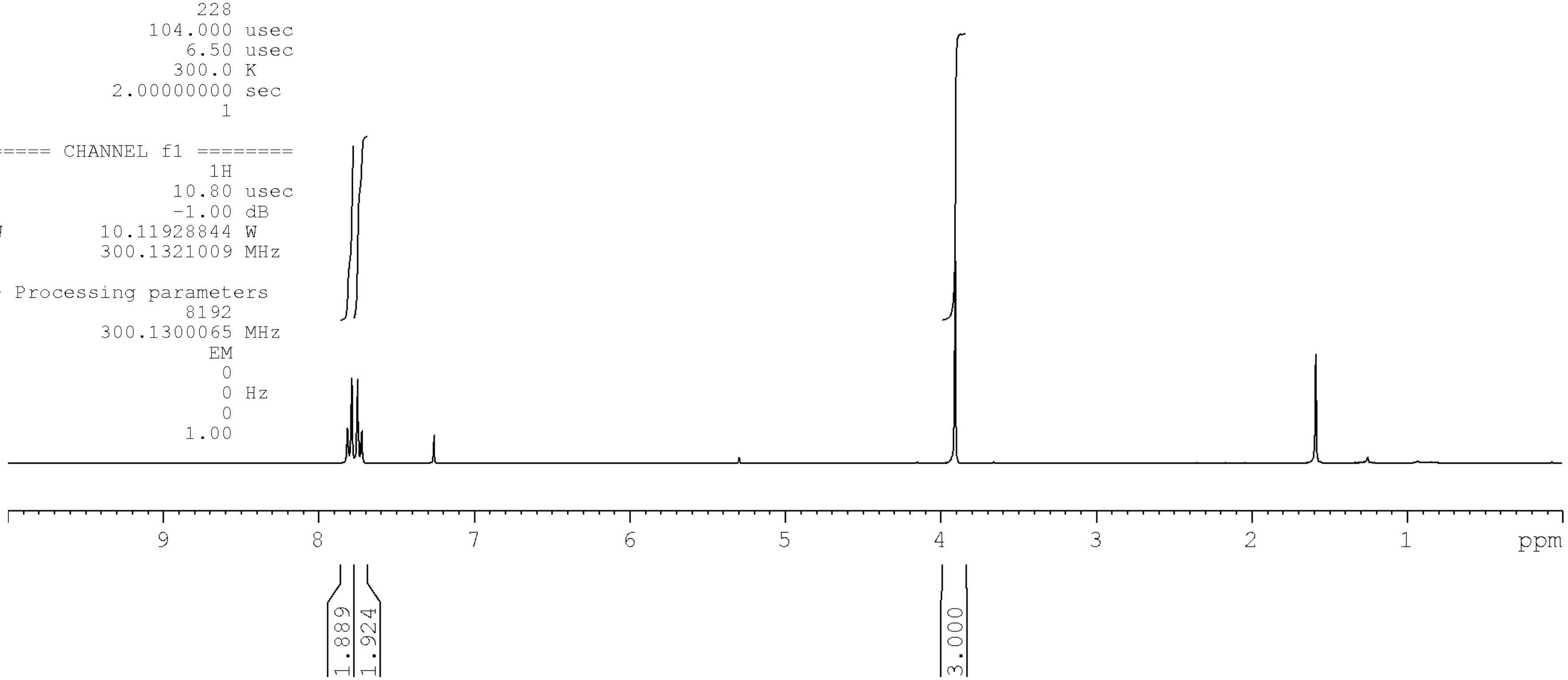

$^1H$ NMR Spectrum of **7d** (300 MHz, $CDCl_3$)

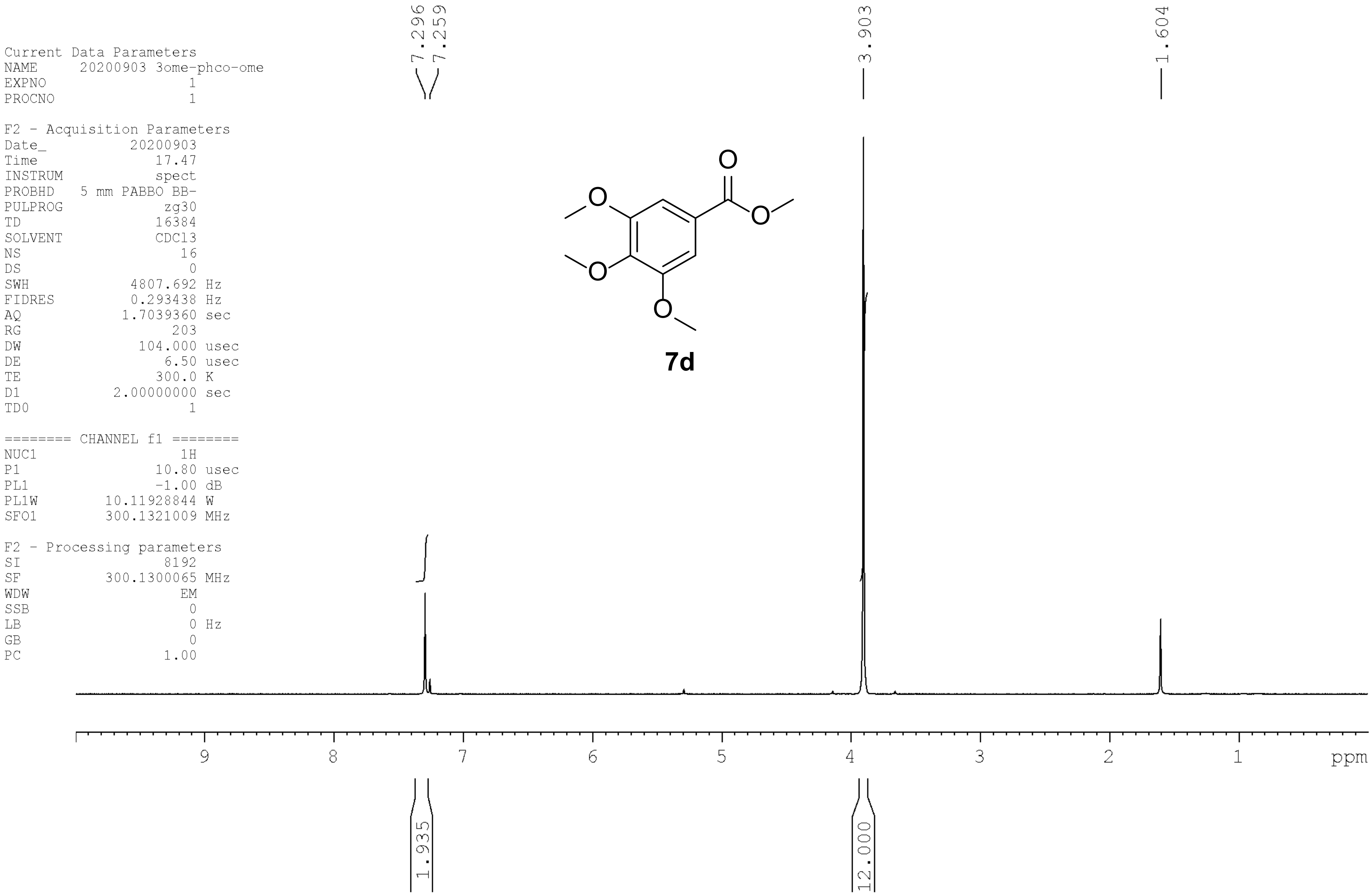

$^{1}$H NMR Spectrum of **7e** (300 MHz, $CDCl_3$)

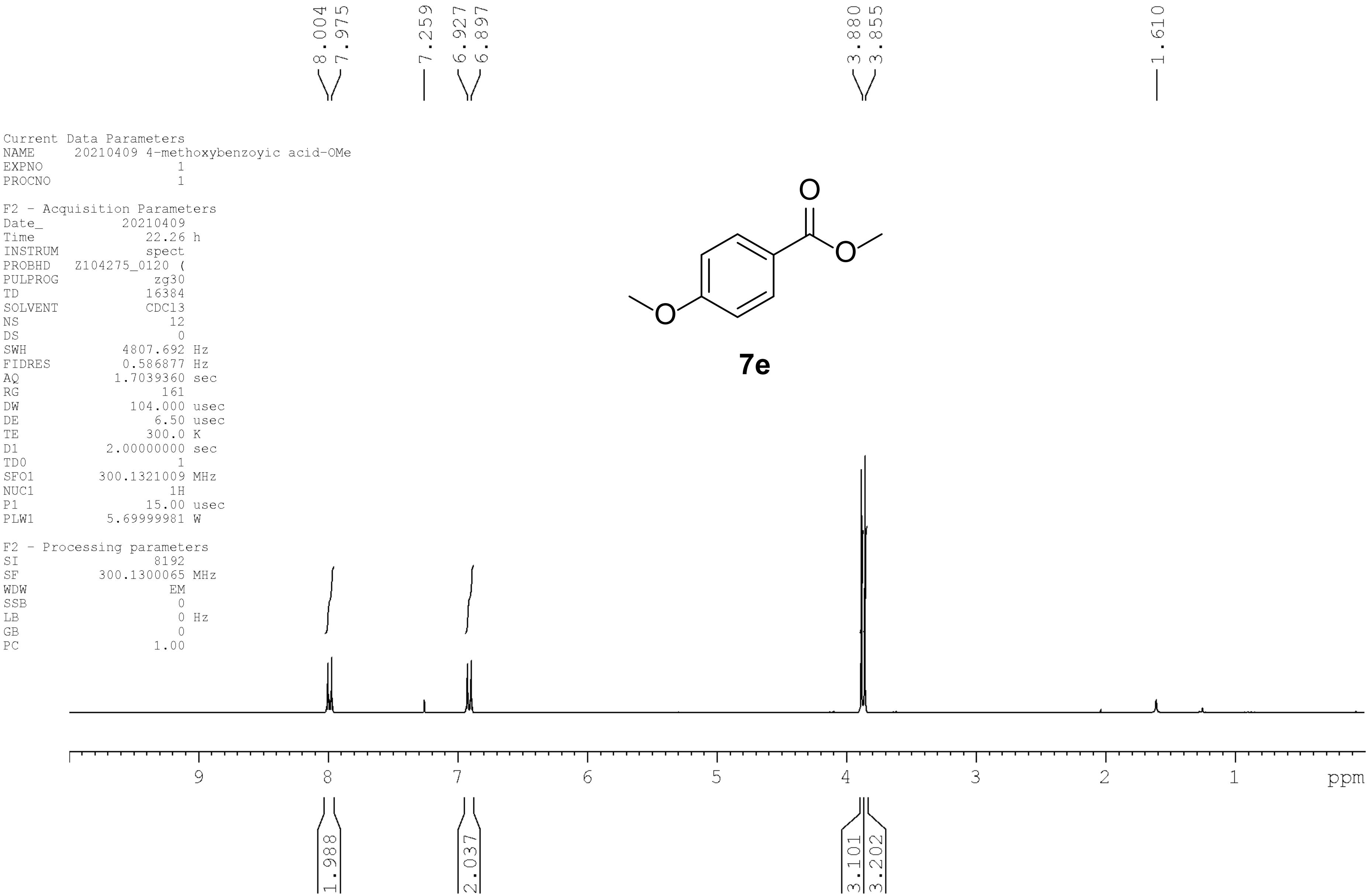

## $^{1}$H NMR Spectrum of **8a** (300 MHz, $CDCl_3$)

```
Current Data Parameters
NAME     20210418 no2-ser
EXPNO                   1
PROCNO                  1

F2 - Acquisition Parameters
Date_          20210518
Time              14.46 h
INSTRUM           spect
PROBHD   Z104275_0120 (
PULPROG            zg30
TD                16384
SOLVENT           CDCl3
NS                   16
DS                    0
SWH            4807.692 Hz
FIDRES         0.586877 Hz
AQ            1.7039360 sec
RG                  203
DW              104.000 usec
DE                 6.50 usec
TE                300.0 K
D1           2.00000000 sec
TD0                   1
SFO1        300.1321009 MHz
NUC1                 1H
P1                15.00 usec
PLW1         5.69999981 W

F2 - Processing parameters
SI                 8192
SF          300.1300076 MHz
WDW                  EM
SSB                   0
LB                    0 Hz
GB                    0
PC                 1.00
```

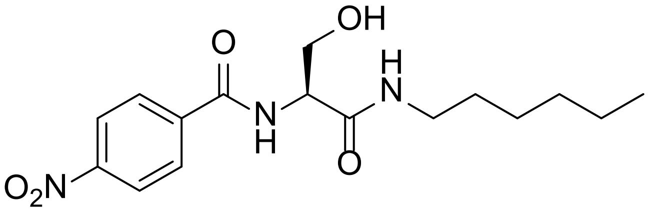

**8a**

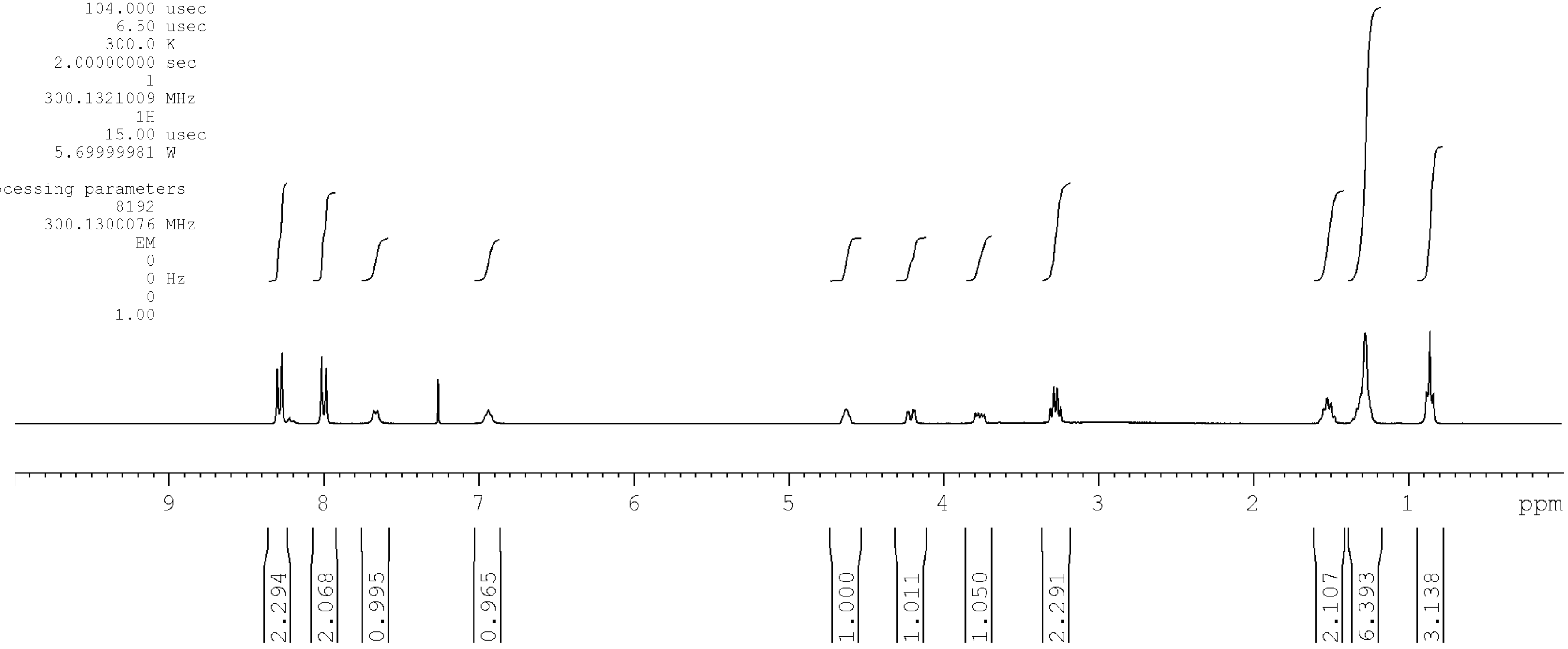

$^{13}C\{^{1}H\}$ NMR Spectrum of **8a** (100 MHz, $CDCl_3$)

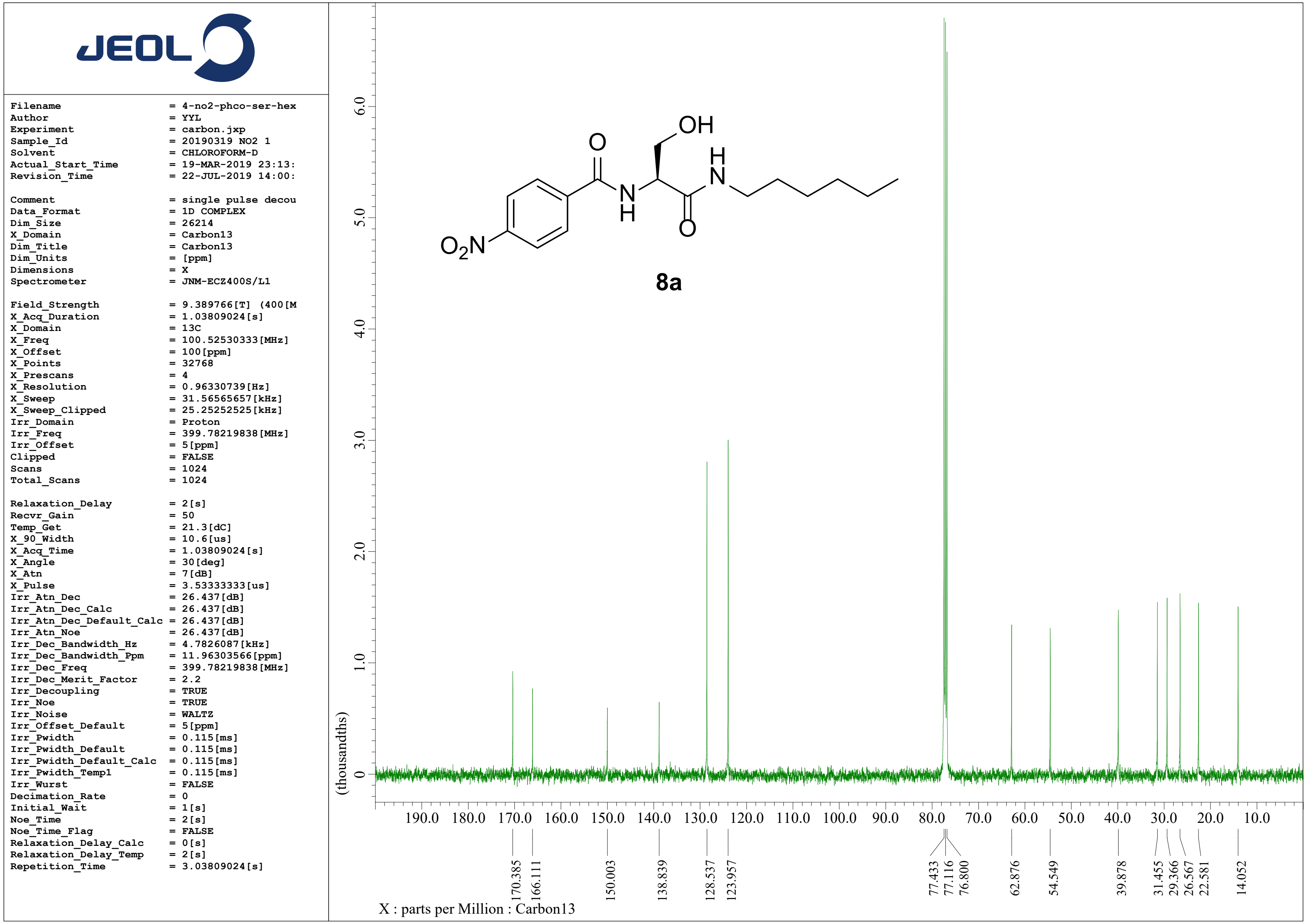

$^{1}$H NMR Spectrum of **8b** (300 MHz, $CD_3OD$)

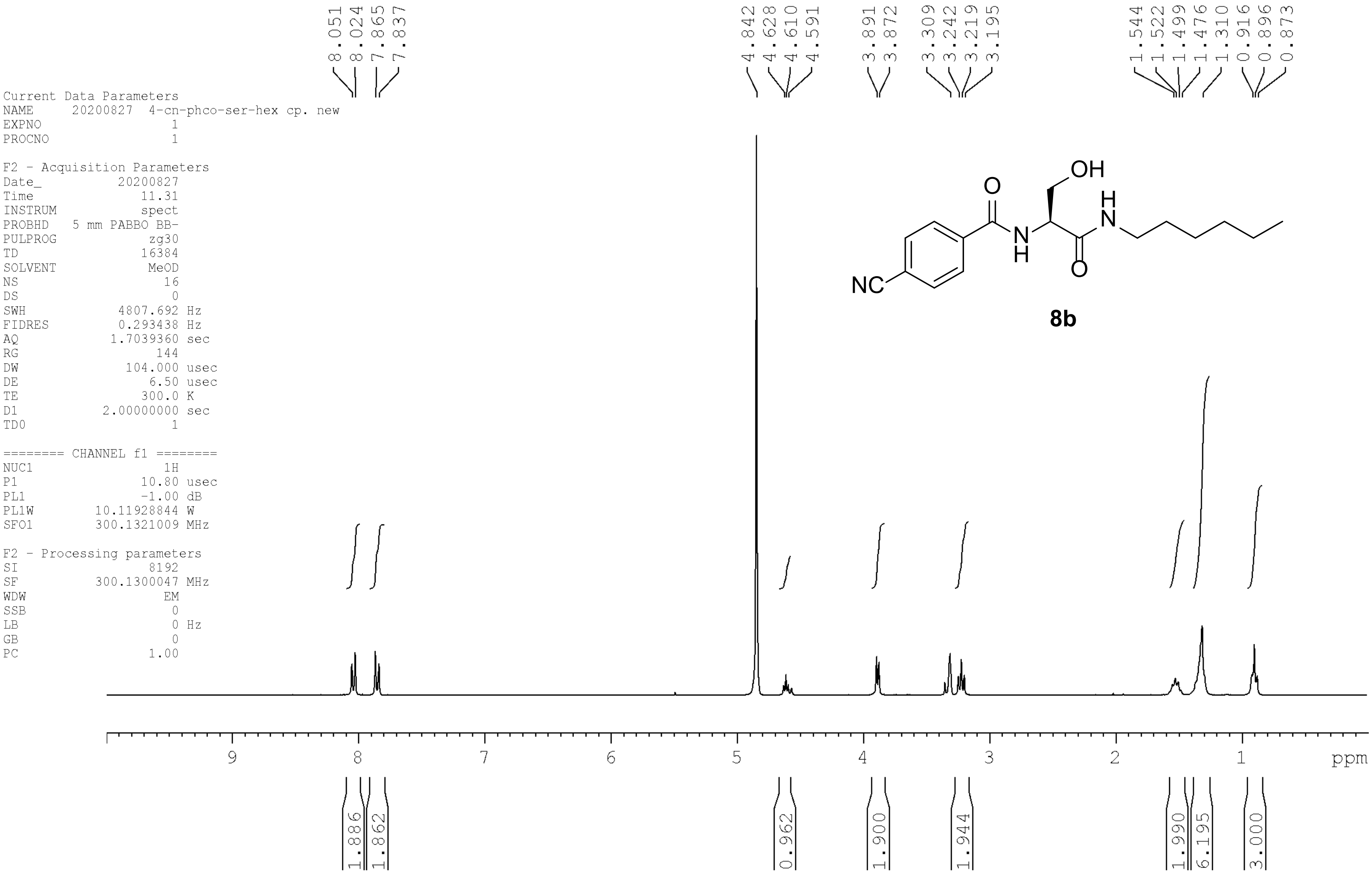

$^{13}C\{^{1}H\}$ NMR Spectrum of **8b** (100 MHz, $CDCl_3$)

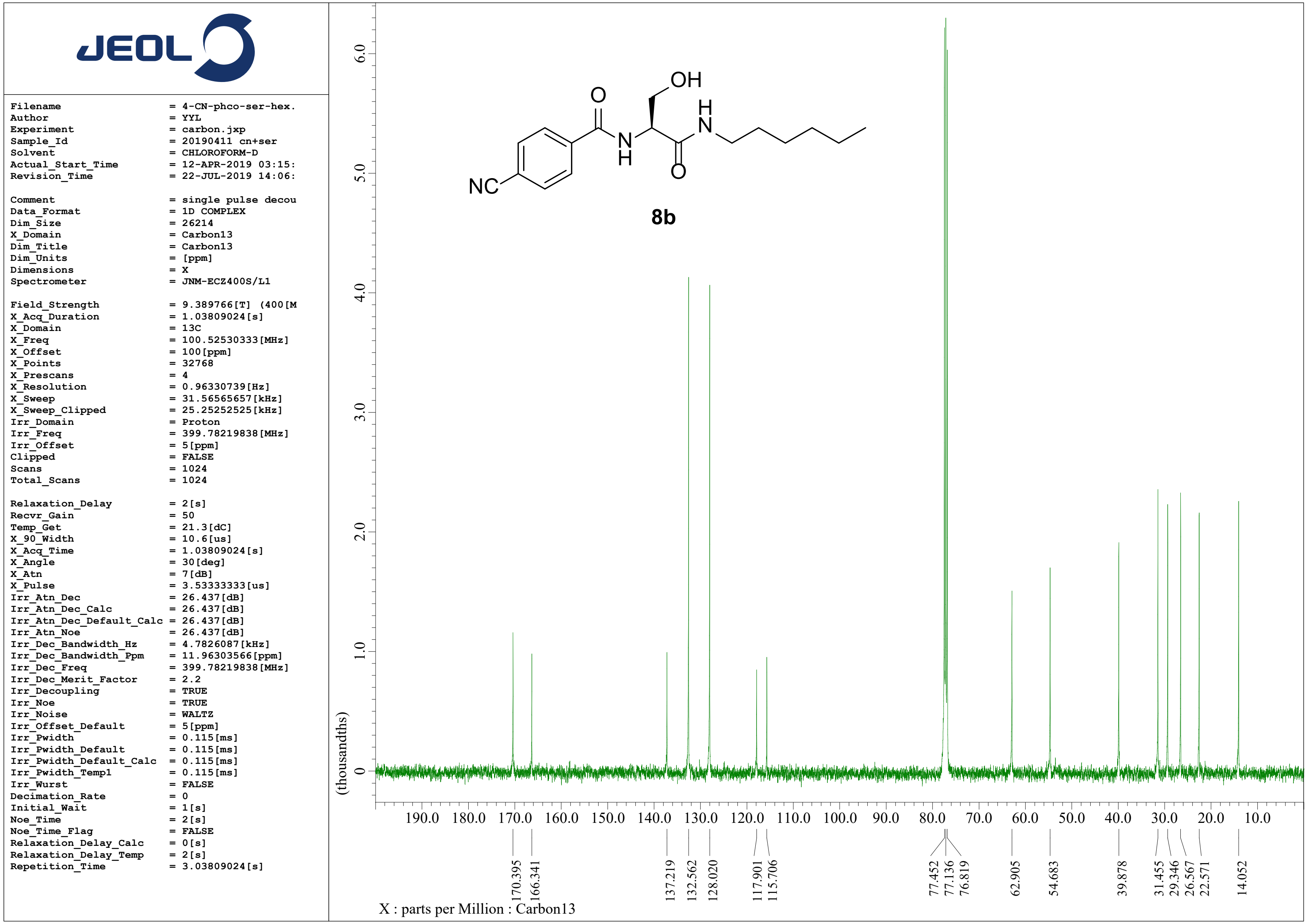

$^{1}$H NMR Spectrum of **8c** (300 MHz, $CD_3OD$)

```
Current Data Parameters
NAME     20200810 i-ser cp NEW
EXPNO                1
PROCNO               1

F2 - Acquisition Parameters
Date_          20200810
Time              17.16
INSTRUM           spect
PROBHD   5 mm PABBO BB-
PULPROG            zg30
TD                16384
SOLVENT            MeOD
NS                   16
DS                    0
SWH            4807.692 Hz
FIDRES         0.293438 Hz
AQ            1.7039360 sec
RG                  181
DW              104.000 usec
DE                 6.50 usec
TE                300.0 K
D1           2.00000000 sec
TD0                   1

======== CHANNEL f1 ========
NUC1                 1H
P1                10.80 usec
PL1               -1.00 dB
PL1W        10.11928844 W
SFO1        300.1321009 MHz

F2 - Processing parameters
SI                 8192
SF          300.1300047 MHz
WDW                  EM
SSB                   0
LB                    0 Hz
GB                    0
PC                 1.00
```

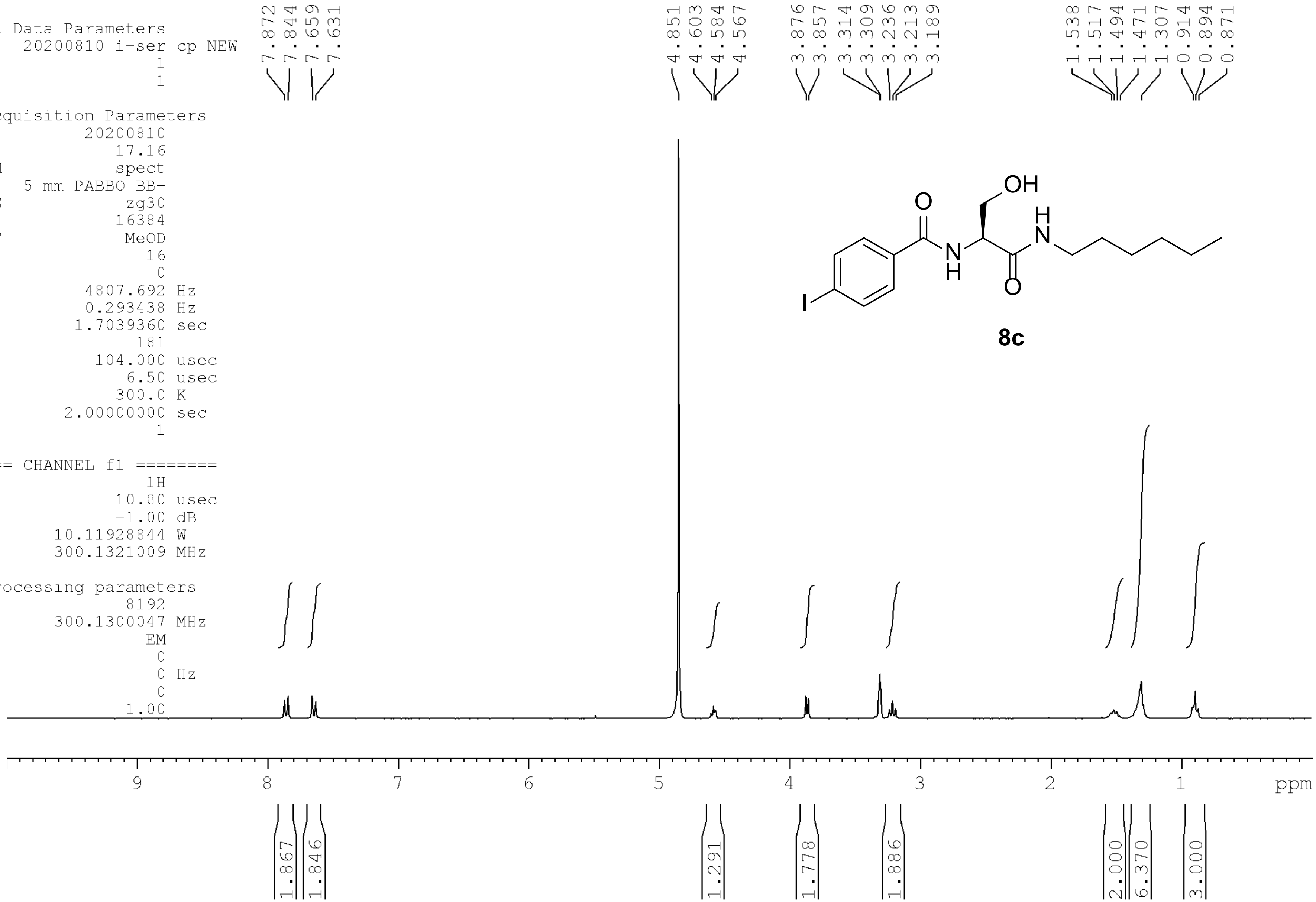

$^{13}C\{^{1}H\}$ NMR Spectrum of **8c** (100 MHz, $CDCl_3$)

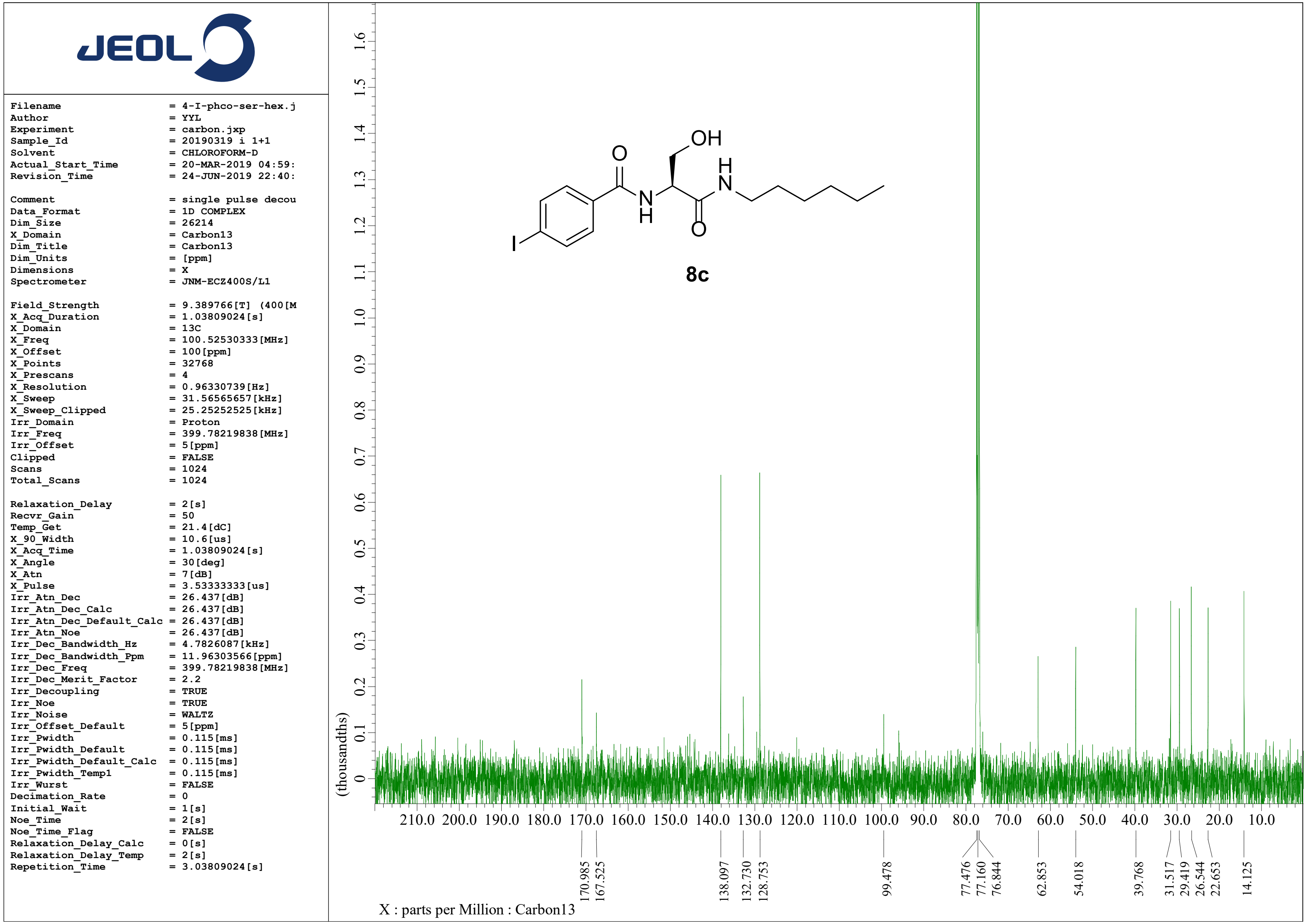

$^{1}$H NMR Spectrum of **8d** (300 MHz, $CDCl_3$)

```
Current Data Parameters
NAME       20210519 3ome-ser
EXPNO                      1
PROCNO                     1

F2 - Acquisition Parameters
Date_           20210519
Time               13.24 h
INSTRUM            spect
PROBHD    Z104275_0120 (
PULPROG             zg30
TD                 16384
SOLVENT            CDCl3
NS                    16
DS                     0
SWH             4807.692 Hz
FIDRES          0.586877 Hz
AQ             1.7039360 sec
RG                   181
DW               104.000 usec
DE                  6.50 usec
TE                 300.0 K
D1            2.00000000 sec
TD0                    1
SFO1        300.1321009 MHz
NUC1                  1H
P1                 15.00 usec
PLW1          5.69999981 W

F2 - Processing parameters
SI                  8192
SF           300.1300076 MHz
WDW                   EM
SSB                    0
LB                     0 Hz
GB                     0
PC                  1.00
```

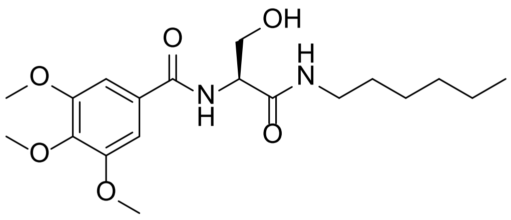


**8d**

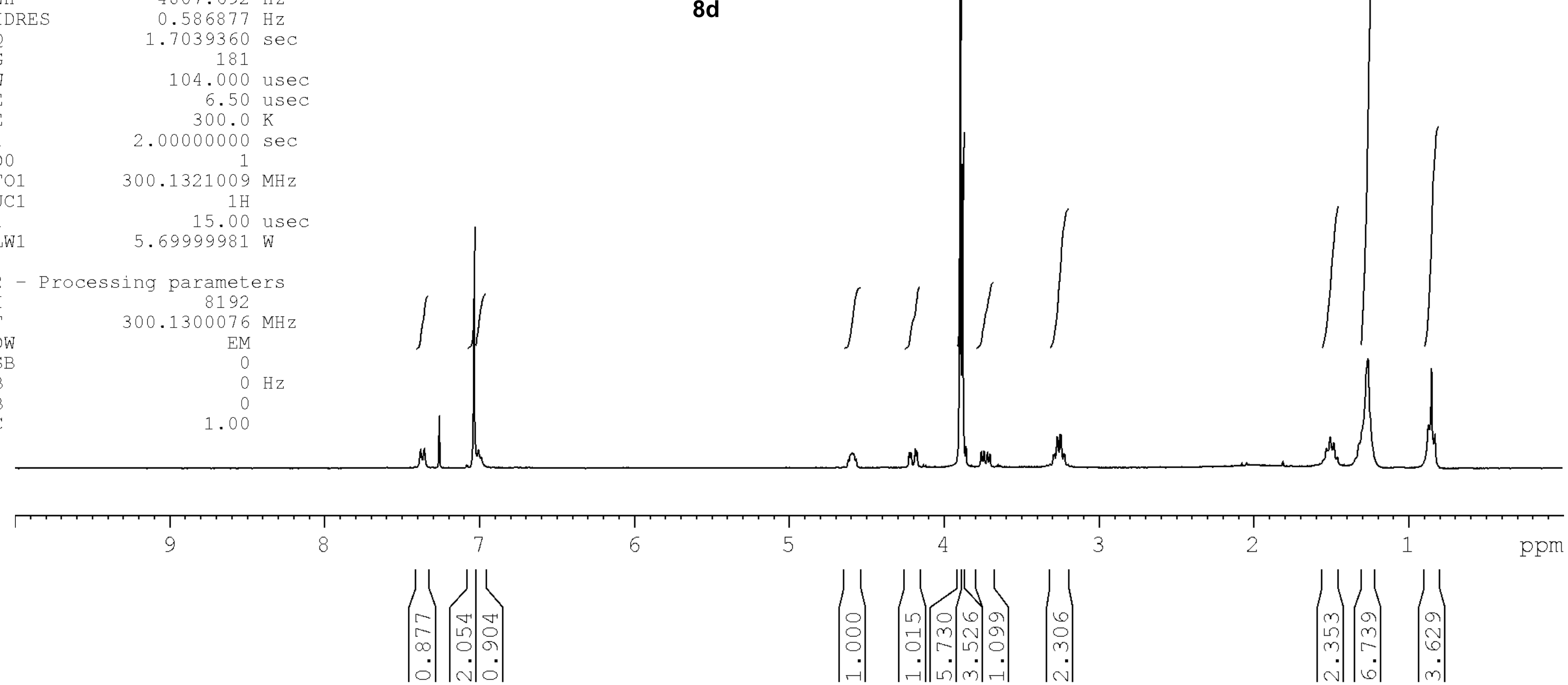

## $^{13}C\{^{1}H\}$ NMR Spectrum of **8d** (100 MHz, $CDCl_3$)

JEOL

```
Filename                    = 3,4,5-OMe-phco-ser
Author                      = YYL
Experiment                  = carbon.jxp
Sample_Id                   = 20190402 ome+ser
Solvent                     = CHLOROFORM-D
Actual_Start_Time           =  2-APR-2019 21:22:
Revision_Time               = 21-APR-2021 17:29:

Comment                     = single pulse decou
Data_Format                 = 1D COMPLEX
Dim_Size                    = 26214
X_Domain                    = Carbon13
Dim_Title                   = Carbon13
Dim_Units                   = [ppm]
Dimensions                  = X
Spectrometer                = JNM-ECZ400S/L1

Field_Strength              = 9.389766[T] (400[M
X_Acq_Duration              = 1.03809024[s]
X_Domain                    = 13C
X_Freq                      = 100.52530333[MHz]
X_Offset                    = 100[ppm]
X_Points                    = 32768
X_Prescans                  = 4
X_Resolution                = 0.96330739[Hz]
X_Sweep                     = 31.56565657[kHz]
X_Sweep_Clipped             = 25.25252525[kHz]
Irr_Domain                  = Proton
Irr_Freq                    = 399.78219838[MHz]
Irr_Offset                  = 5[ppm]
Clipped                     = FALSE
Scans                       = 2048
Total_Scans                 = 2048

Relaxation_Delay            = 2[s]
Recvr_Gain                  = 50
Temp_Get                    = 22.2[dC]
X_90_Width                  = 10.6[us]
X_Acq_Time                  = 1.03809024[s]
X_Angle                     = 30[deg]
X_Atn                       = 7[dB]
X_Pulse                     = 3.53333333[us]
Irr_Atn_Dec                 = 26.437[dB]
Irr_Atn_Dec_Calc            = 26.437[dB]
Irr_Atn_Dec_Default_Calc    = 26.437[dB]
Irr_Atn_Noe                 = 26.437[dB]
Irr_Dec_Bandwidth_Hz        = 4.7826087[kHz]
Irr_Dec_Bandwidth_Ppm       = 11.96303566[ppm]
Irr_Dec_Freq                = 399.78219838[MHz]
Irr_Dec_Merit_Factor        = 2.2
Irr_Decoupling              = TRUE
Irr_Noe                     = TRUE
Irr_Noise                   = WALTZ
Irr_Offset_Default          = 5[ppm]
Irr_Pwidth                  = 0.115[ms]
Irr_Pwidth_Default          = 0.115[ms]
Irr_Pwidth_Default_Calc     = 0.115[ms]
Irr_Pwidth_Templ            = 0.115[ms]
Irr_Wurst                   = FALSE
Decimation_Rate             = 0
Initial_Wait                = 1[s]
Noe_Time                    = 2[s]
Noe_Time_Flag               = FALSE
Relaxation_Delay_Calc       = 0[s]
Relaxation_Delay_Temp       = 2[s]
Repetition_Time             = 3.03809024[s]
```

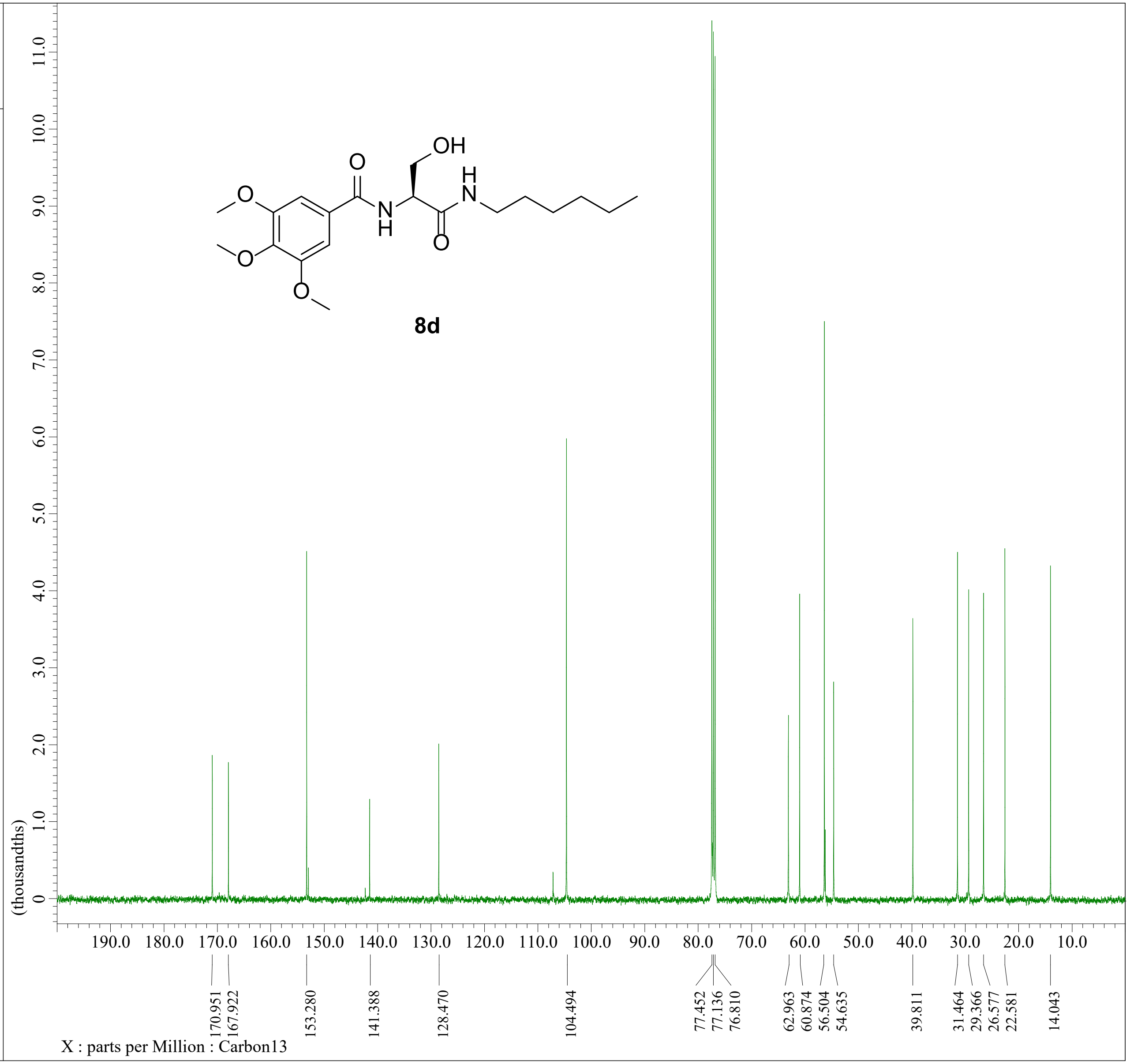

$^{1}$H NMR Spectrum of **8e** (300 MHz, $CDCl_3$)

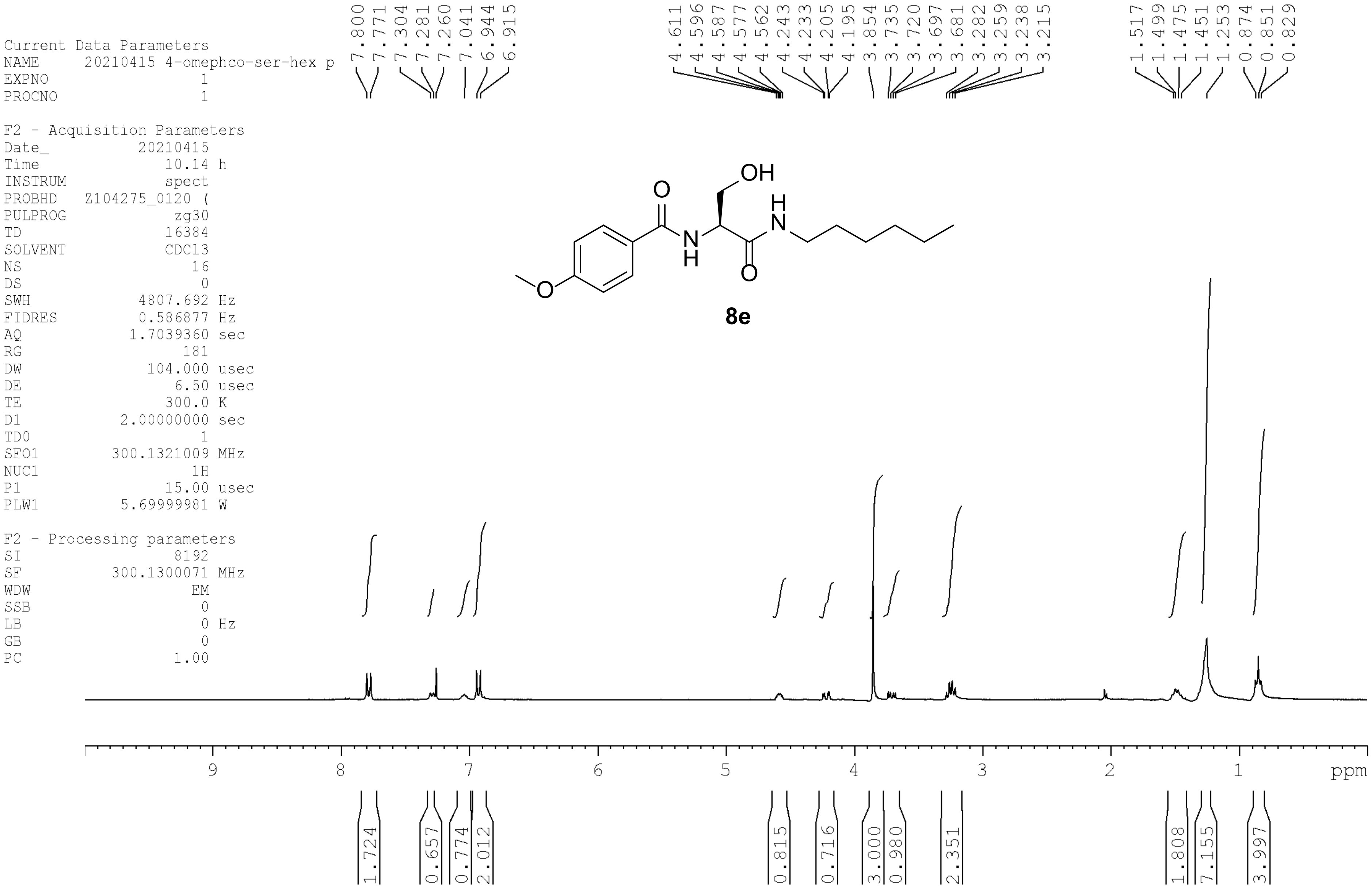

## $^{13}C\{^{1}H\}$ NMR Spectrum of **8e** (100 MHz, $CDCl_3$)

JEOL

```
Filename                  = 4-ome-phco-ser-hex
Author                    = YYL
Experiment                = carbon.jxp
Sample_Id                 = 4-ome-phco-ser-hex
Solvent                   = CHLOROFORM-D
Actual_Start_Time         = 16-APR-2021 12:39:
Revision_Time             = 26-APR-2021 23:03:

Comment                   = single pulse decou
Data_Format               = 1D COMPLEX
Dim_Size                  = 26214
X_Domain                  = Carbon13
Dim_Title                 = Carbon13
Dim_Units                 = [ppm]
Dimensions                = X
Spectrometer              = JNM-ECZ400S/L1

Field_Strength            = 9.389766[T] (400[M
X_Acq_Duration            = 1.03809024[s]
X_Domain                  = 13C
X_Freq                    = 100.52530333[MHz]
X_Offset                  = 100[ppm]
X_Points                  = 32768
X_Prescans                = 4
X_Resolution              = 0.96330739[Hz]
X_Sweep                   = 31.56565657[kHz]
X_Sweep_Clipped           = 25.25252525[kHz]
Irr_Domain                = Proton
Irr_Freq                  = 399.78219838[MHz]
Irr_Offset                = 5[ppm]
Clipped                   = FALSE
Scans                     = 1024
Total_Scans               = 1024

Relaxation_Delay          = 2[s]
Recvr_Gain                = 50
Temp_Get                  = 22.1[dC]
X_90_Width                = 10.3[us]
X_Acq_Time                = 1.03809024[s]
X_Angle                   = 30[deg]
X_Atn                     = 7[dB]
X_Pulse                   = 3.43333333[us]
Irr_Atn_Dec               = 26.116[dB]
Irr_Atn_Dec_Calc          = 26.116[dB]
Irr_Atn_Dec_Default_Calc  = 26.116[dB]
Irr_Atn_Noe               = 26.116[dB]
Irr_Dec_Bandwidth_Hz      = 4.7826087[kHz]
Irr_Dec_Bandwidth_Ppm     = 11.96303566[ppm]
Irr_Dec_Freq              = 399.78219838[MHz]
Irr_Dec_Merit_Factor      = 2.2
Irr_Decoupling            = TRUE
Irr_Noe                   = TRUE
Irr_Noise                 = WALTZ
Irr_Offset_Default        = 5[ppm]
Irr_Pwidth                = 0.115[ms]
Irr_Pwidth_Default        = 0.115[ms]
Irr_Pwidth_Default_Calc   = 0.115[ms]
Irr_Pwidth_Templ          = 0.115[ms]
Irr_Wurst                 = FALSE
Decimation_Rate           = 0
Initial_Wait              = 1[s]
Noe_Time                  = 2[s]
Noe_Time_Flag             = FALSE
Relaxation_Delay_Calc     = 0[s]
Relaxation_Delay_Temp     = 2[s]
Repetition_Time           = 3.03809024[s]
```

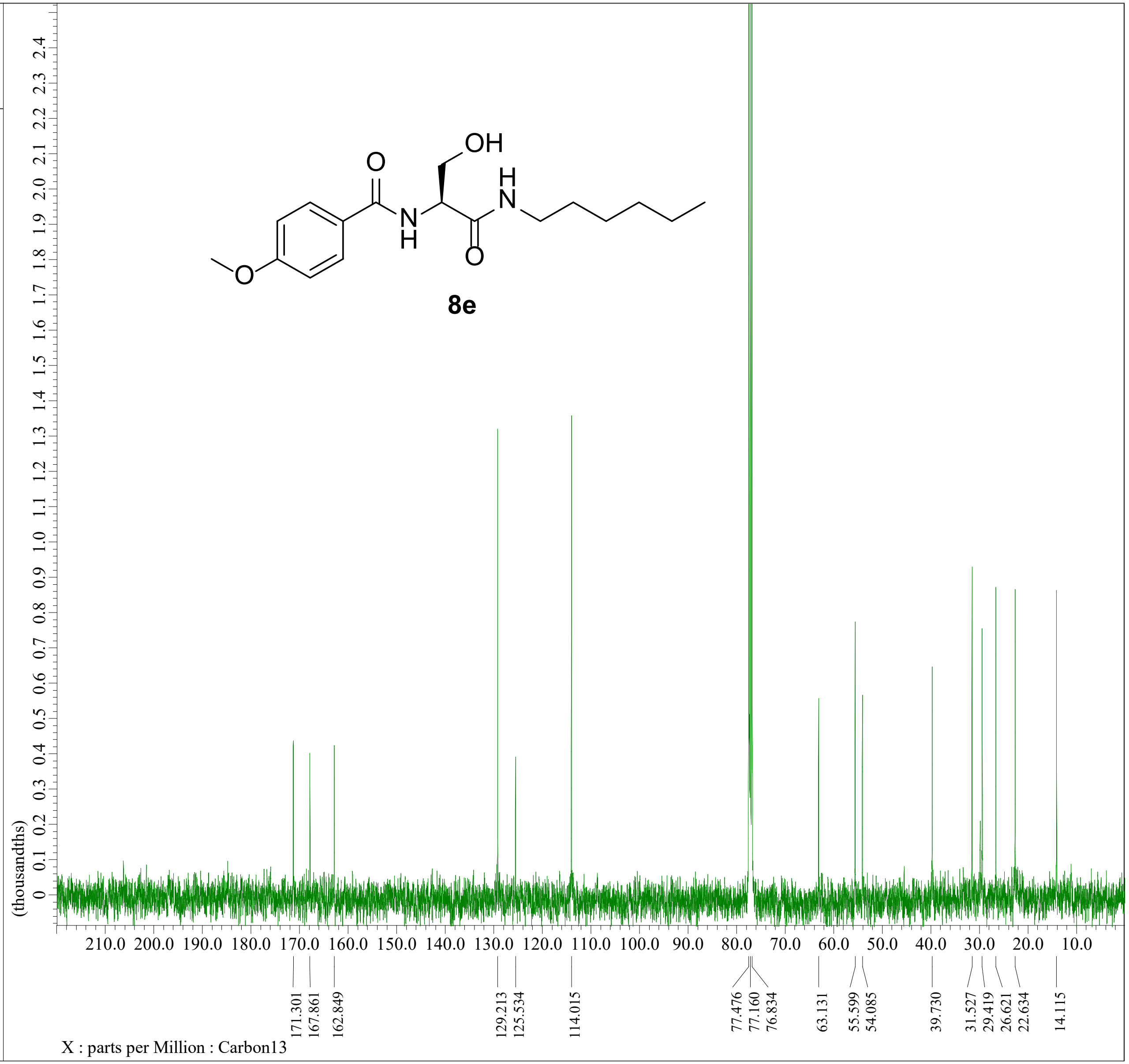

$^{1}$H NMR Spectrum of **8f** (300 MHz, $CDCl_3$)

```
Current Data Parameters
NAME     20200831 phco-ser cp
EXPNO                   1
PROCNO                  1

F2 - Acquisition Parameters
Date_          20200831
Time              14.18
INSTRUM           spect
PROBHD   5 mm PABBO BB-
PULPROG            zg30
TD                16384
SOLVENT           CDCl3
NS                   16
DS                    0
SWH            4807.692 Hz
FIDRES         0.293438 Hz
AQ            1.7039360 sec
RG                  228
DW              104.000 usec
DE                 6.50 usec
TE                300.0 K
D1           2.00000000 sec
TD0                   1

======== CHANNEL f1 ========
NUC1                 1H
P1                10.80 usec
PL1               -1.00 dB
PL1W        10.11928844 W
SFO1        300.1321009 MHz

F2 - Processing parameters
SI                 8192
SF          300.1300061 MHz
WDW                  EM
SSB                   0
LB                    0 Hz
GB                    0
PC                 1.00
```

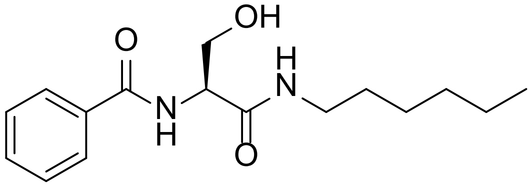

**8f**

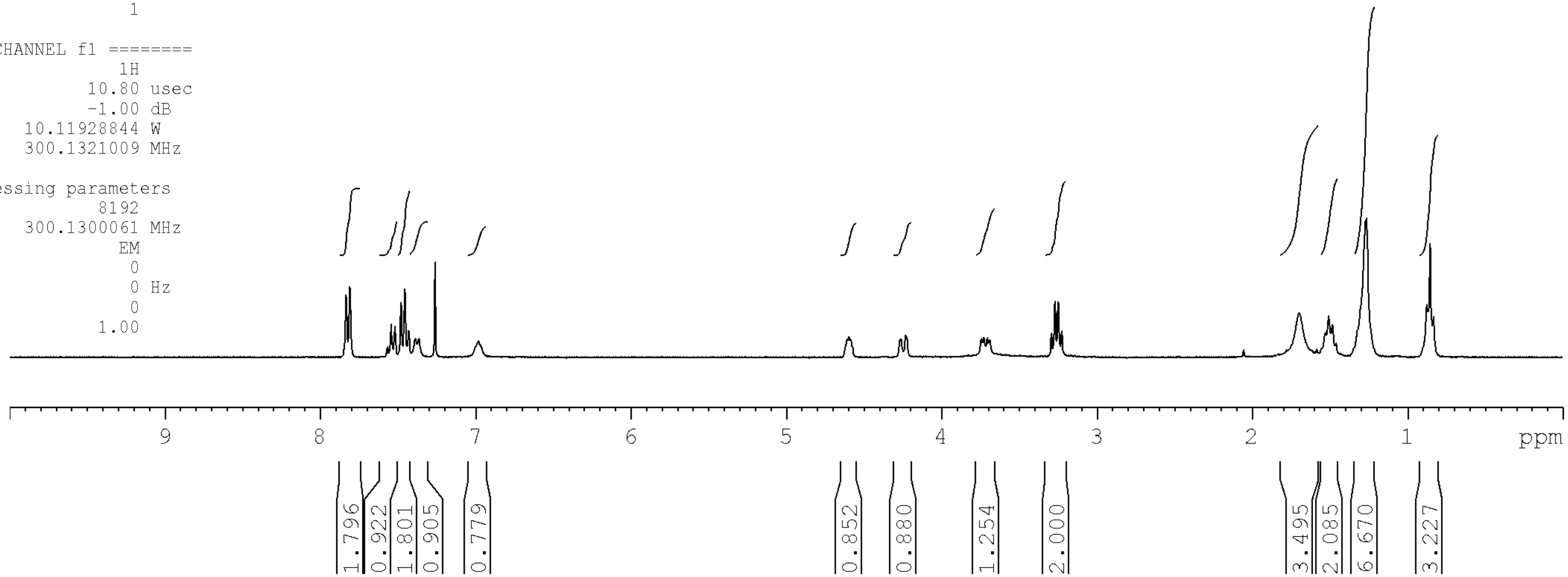

$^{13}C\{^{1}H\}$ NMR Spectrum of **8f** (100 MHz, $CDCl_3$)

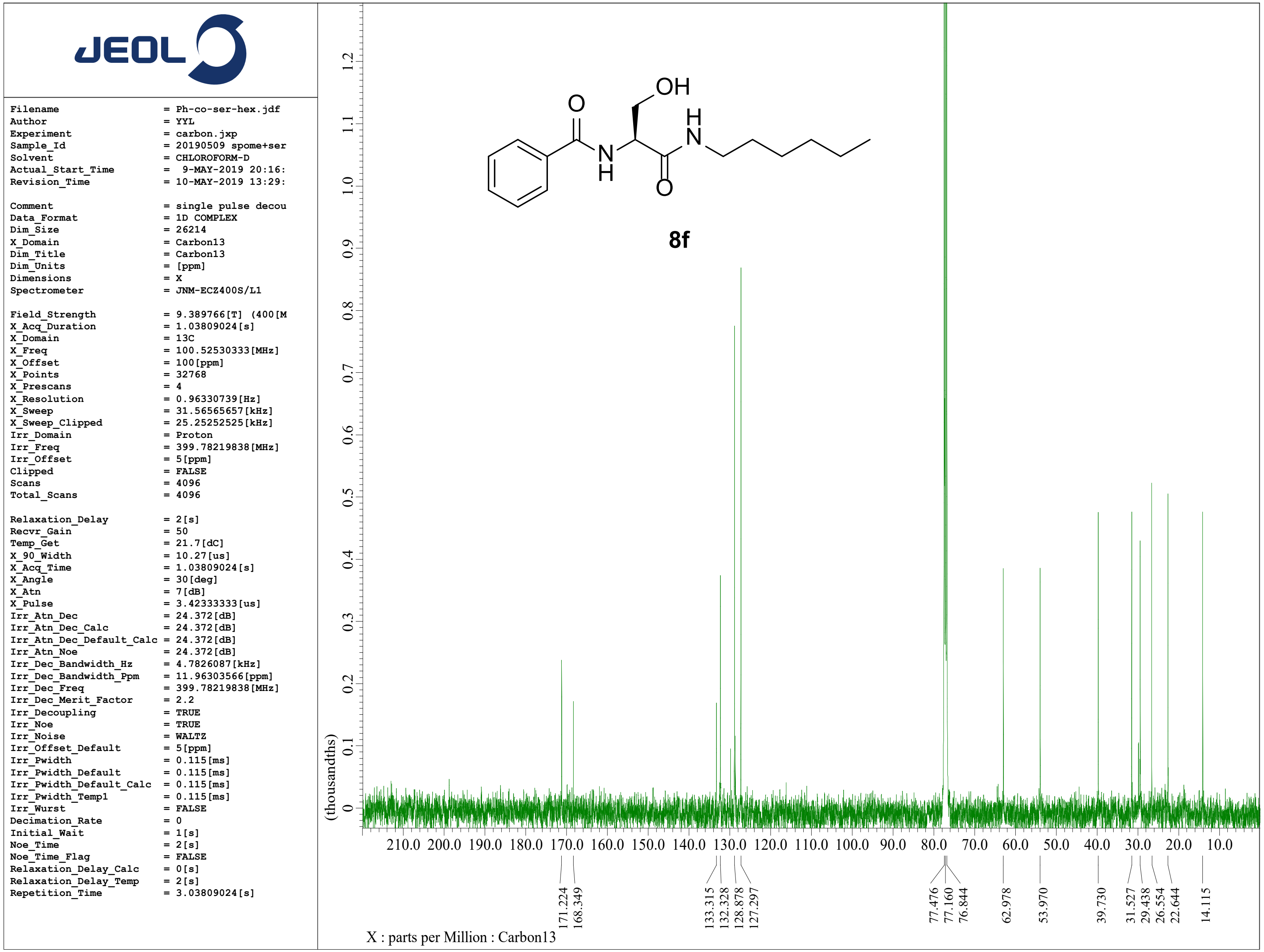

## $^{1}$H NMR Spectrum of **8g** (300 MHz, $CD_3OD$)

```
Current Data Parameters
NAME      20200827  bnco-ser-hex cp
EXPNO                    1
PROCNO                   1

F2 - Acquisition Parameters
Date_          20200827
Time              11.15
INSTRUM           spect
PROBHD   5 mm PABBO BB-
PULPROG            zg30
TD                16384
SOLVENT            MeOD
NS                   16
DS                    0
SWH            4807.692 Hz
FIDRES         0.293438 Hz
AQ            1.7039360 sec
RG                  181
DW              104.000 usec
DE                 6.50 usec
TE                300.0 K
D1           2.00000000 sec
TD0                   1

======== CHANNEL f1 ========
NUC1                 1H
P1                10.80 usec
PL1               -1.00 dB
PL1W        10.11928844 W
SFO1        300.1321009 MHz

F2 - Processing parameters
SI                 8192
SF          300.1300044 MHz
WDW                  EM
SSB                   0
LB                    0 Hz
GB                    0
PC                 1.00
```

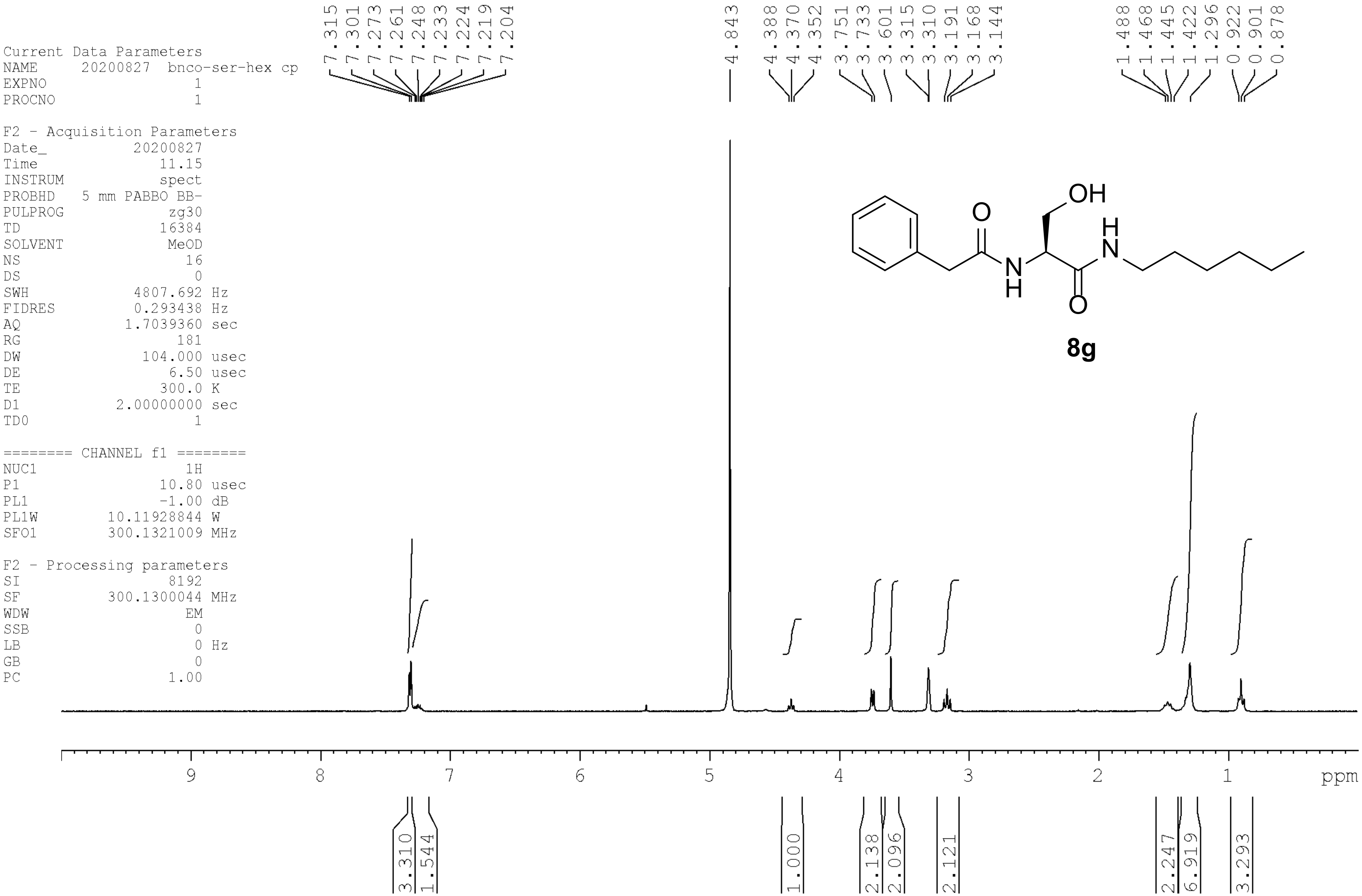


**8g**

$^{13}C\{^{1}H\}$ NMR Spectrum of **8g** (100 MHz, $CDCl_3$)

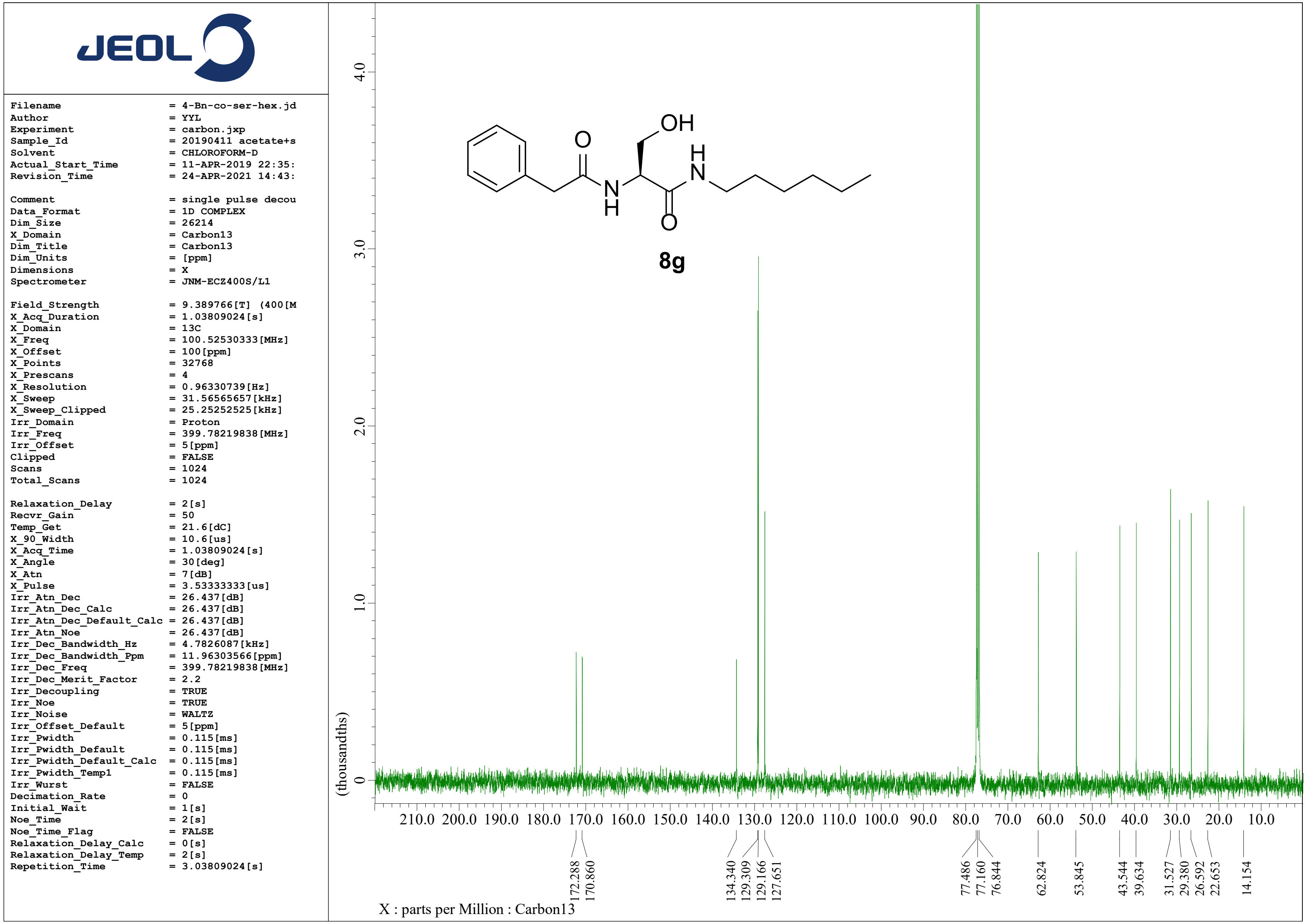

$^{1}$H NMR Spectrum of **8h** (300 MHz, $CDCl_3$)

```
Current Data Parameters
NAME     20200904 cyclo-ser-hex
EXPNO                 1
PROCNO                1

F2 - Acquisition Parameters
Date_          20200904
Time              10.51
INSTRUM           spect
PROBHD   5 mm PABBO BB-
PULPROG            zg30
TD                16384
SOLVENT           CDCl3
NS                   16
DS                    0
SWH            4807.692 Hz
FIDRES         0.293438 Hz
AQ            1.7039360 sec
RG                  203
DW              104.000 usec
DE                 6.50 usec
TE                300.0 K
D1           2.00000000 sec
TD0                   1

======== CHANNEL f1 ========
NUC1                 1H
P1                10.80 usec
PL1               -1.00 dB
PL1W        10.11928844 W
SFO1        300.1321009 MHz

F2 - Processing parameters
SI                 8192
SF          300.1300062 MHz
WDW                  EM
SSB                   0
LB                    0 Hz
GB                    0
PC                 1.00
```

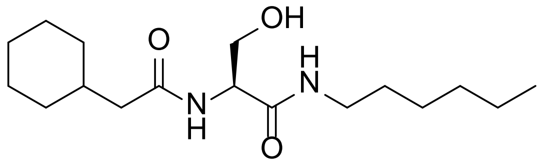


**8h**

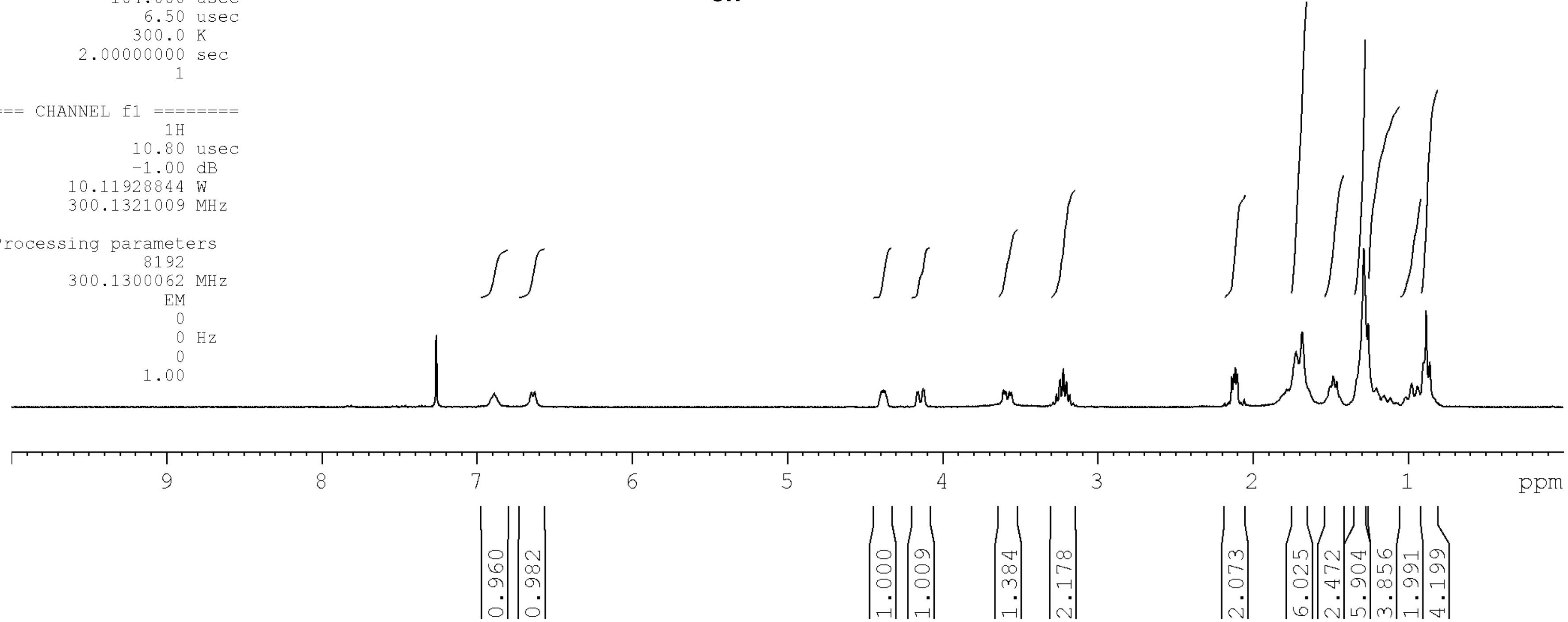

## $^{13}C\{^{1}H\}$ NMR Spectrum of **8h** (100 MHz, $CDCl_3$)

| | |
|---|---|
| Filename | = cyclo-hex-co-ser-h |
| Author | = YYL |
| Experiment | = carbon.jxp |
| Sample_Id | = 20190509 six+ser d |
| Solvent | = CHLOROFORM-D |
| Actual_Start_Time | = 9-MAY-2019 23:50: |
| Revision_Time | = 24-JUN-2019 22:58: |
| Comment | = single pulse decou |
| Data_Format | = 1D COMPLEX |
| Dim_Size | = 26214 |
| X_Domain | = Carbon13 |
| Dim_Title | = Carbon13 |
| Dim_Units | = [ppm] |
| Dimensions | = X |
| Spectrometer | = JNM-ECZ400S/L1 |
| Field_Strength | = 9.389766[T] (400[M |
| X_Acq_Duration | = 1.03809024[s] |
| X_Domain | = 13C |
| X_Freq | = 100.52530333[MHz] |
| X_Offset | = 100[ppm] |
| X_Points | = 32768 |
| X_Prescans | = 4 |
| X_Resolution | = 0.96330739[Hz] |
| X_Sweep | = 31.56565657[kHz] |
| X_Sweep_Clipped | = 25.25252525[kHz] |
| Irr_Domain | = Proton |
| Irr_Freq | = 399.78219838[MHz] |
| Irr_Offset | = 5[ppm] |
| Clipped | = FALSE |
| Scans | = 4096 |
| Total_Scans | = 4096 |
| Relaxation_Delay | = 2[s] |
| Recvr_Gain | = 50 |
| Temp_Get | = 21.8[dC] |
| X_90_Width | = 10.27[us] |
| X_Acq_Time | = 1.03809024[s] |
| X_Angle | = 30[deg] |
| X_Atn | = 7[dB] |
| X_Pulse | = 3.42333333[us] |
| Irr_Atn_Dec | = 24.372[dB] |
| Irr_Atn_Dec_Calc | = 24.372[dB] |
| Irr_Atn_Dec_Default_Calc | = 24.372[dB] |
| Irr_Atn_Noe | = 24.372[dB] |
| Irr_Dec_Bandwidth_Hz | = 4.7826087[kHz] |
| Irr_Dec_Bandwidth_Ppm | = 11.96303566[ppm] |
| Irr_Dec_Freq | = 399.78219838[MHz] |
| Irr_Dec_Merit_Factor | = 2.2 |
| Irr_Decoupling | = TRUE |
| Irr_Noe | = TRUE |
| Irr_Noise | = WALTZ |
| Irr_Offset_Default | = 5[ppm] |
| Irr_Pwidth | = 0.115[ms] |
| Irr_Pwidth_Default | = 0.115[ms] |
| Irr_Pwidth_Default_Calc | = 0.115[ms] |
| Irr_Pwidth_Temp1 | = 0.115[ms] |
| Irr_Wurst | = FALSE |
| Decimation_Rate | = 0 |
| Initial_Wait | = 1[s] |
| Noe_Time | = 2[s] |
| Noe_Time_Flag | = FALSE |
| Relaxation_Delay_Calc | = 0[s] |
| Relaxation_Delay_Temp | = 2[s] |
| Repetition_Time | = 3.03809024[s] |

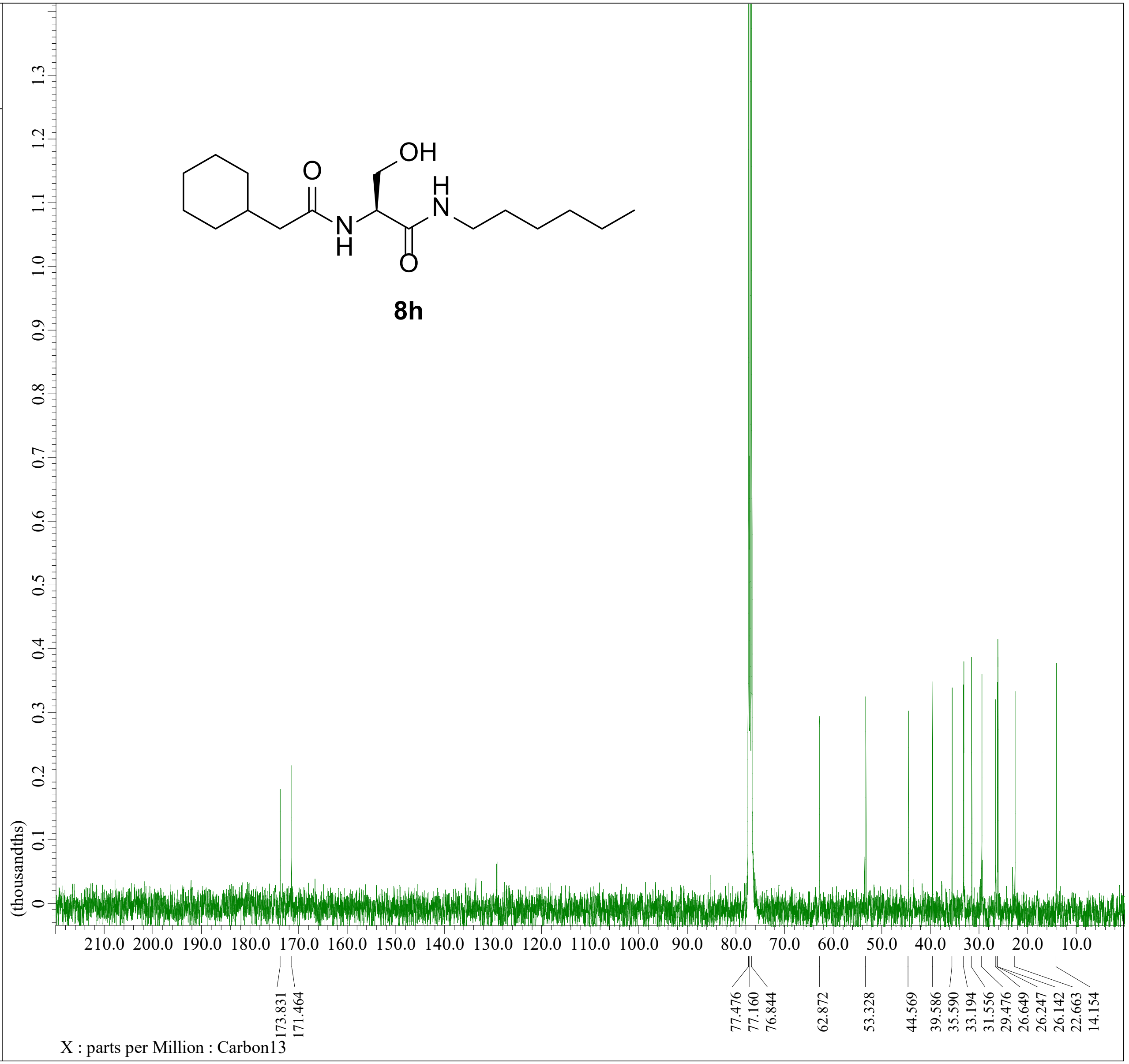

$^{1}$H NMR Spectrum of **9a** (300 MHz, $CDCl_3$)

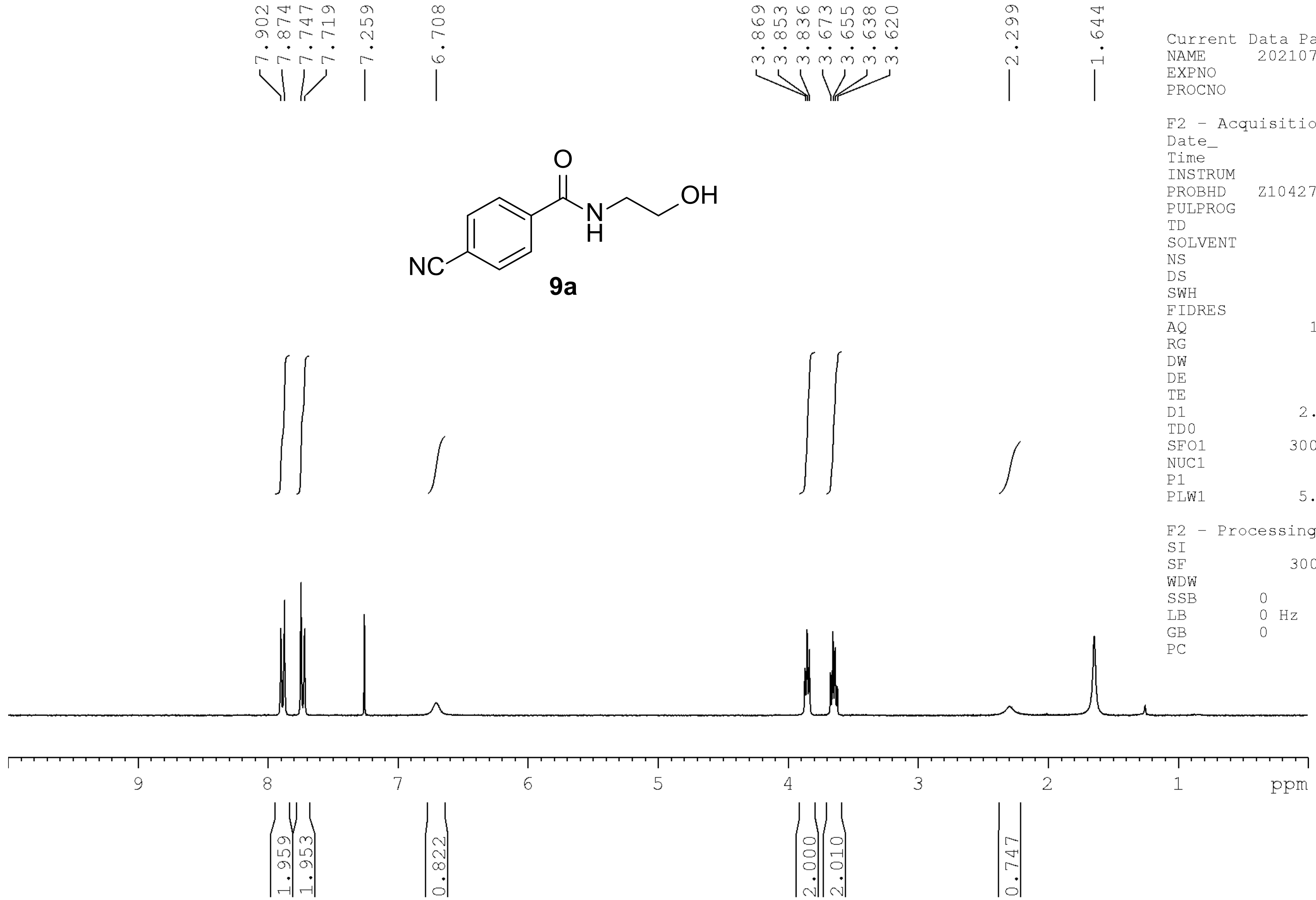


```
Current Data Parameters
NAME       20210705 4-cn-sm(2)
EXPNO                    1
PROCNO                   1

F2 - Acquisition Parameters
Date_            20210705
Time                15.36 h
INSTRUM             spect
PROBHD     Z104275_0120 (
PULPROG              zg30
TD                  16384
SOLVENT             CDCl3
NS                     16
DS                      0
SWH              4807.692 Hz
FIDRES           0.586877 Hz
AQ              1.7039360 sec
RG                    322
DW                104.000 usec
DE                   6.50 usec
TE                  300.0 K
D1            2.00000000 sec
TD0                     1
SFO1          300.1321009 MHz
NUC1                   1H
P1                  15.00 usec
PLW1           5.69999981 W

F2 - Processing parameters
SI                   8192
SF            300.1300076 MHz
WDW                    EM
SSB        0
LB         0 Hz
GB         0
PC                   1.00
```

$^{1}$H NMR Spectrum of **9b** (300 MHz, $CDCl_3$)

```
Current Data Parameters
NAME     20210705 4-cn-sm(3)
EXPNO                    1
PROCNO                   1

F2 - Acquisition Parameters
Date_          20210705
Time              15.40 h
INSTRUM           spect
PROBHD   Z104275_0120 (
PULPROG            zg30
TD                16384
SOLVENT           CDCl3
NS                   16
DS                    0
SWH            4807.692 Hz
FIDRES         0.586877 Hz
AQ            1.7039360 sec
RG                  256
DW              104.000 usec
DE                 6.50 usec
TE                300.0 K
D1           2.00000000 sec
TD0                   1
SFO1        300.1321009 MHz
NUC1                 1H
P1                15.00 usec
PLW1         5.69999981 W

F2 - Processing parameters
SI                 8192
SF          300.1300076 MHz
WDW                  EM
SSB      0
LB       0 Hz
GB       0
PC                 1.00
```

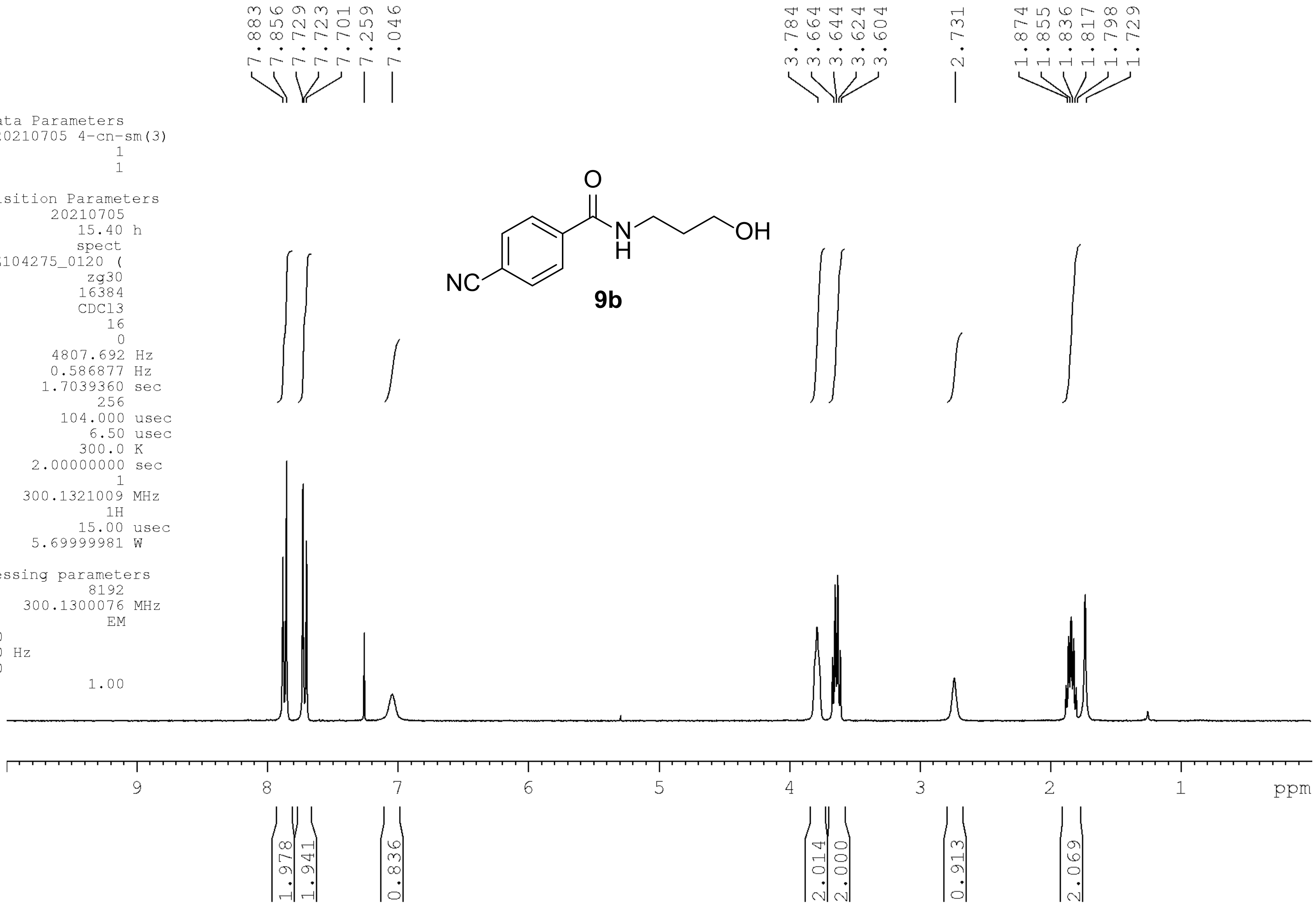

$^{13}C\{^{1}H\}$ NMR Spectrum of **9b** (100 MHz, $CDCl_3$)

| | |
|---|---|
| Filename | = 4-cn-nh(ch2)3oh.jd |
| Author | = YYL |
| Experiment | = carbon.jxp |
| Sample_Id | = 4-cn-nh(ch2)3oh -c |
| Solvent | = CHLOROFORM-D |
| Actual_Start_Time | = 23-MAY-2021 12:42: |
| Revision_Time | = 24-MAY-2021 23:20: |
| Comment | = single pulse decou |
| Data_Format | = 1D COMPLEX |
| Dim_Size | = 26214 |
| X_Domain | = Carbon13 |
| Dim_Title | = Carbon13 |
| Dim_Units | = [ppm] |
| Dimensions | = X |
| Spectrometer | = JNM-ECZ400S/L1 |
| Field_Strength | = 9.389766[T] (400[M |
| X_Acq_Duration | = 1.03809024[s] |
| X_Domain | = 13C |
| X_Freq | = 100.52530333[MHz] |
| X_Offset | = 100[ppm] |
| X_Points | = 32768 |
| X_Prescans | = 4 |
| X_Resolution | = 0.96330739[Hz] |
| X_Sweep | = 31.56565657[kHz] |
| X_Sweep_Clipped | = 25.25252525[kHz] |
| Irr_Domain | = Proton |
| Irr_Freq | = 399.78219838[MHz] |
| Irr_Offset | = 5[ppm] |
| Clipped | = FALSE |
| Scans | = 512 |
| Total_Scans | = 512 |
| Relaxation_Delay | = 2[s] |
| Recvr_Gain | = 50 |
| Temp_Get | = 24.8[dC] |
| X_90_Width | = 10.3[us] |
| X_Acq_Time | = 1.03809024[s] |
| X_Angle | = 30[deg] |
| X_Atn | = 7[dB] |
| X_Pulse | = 3.43333333[us] |
| Irr_Atn_Dec | = 26.116[dB] |
| Irr_Atn_Dec_Calc | = 26.116[dB] |
| Irr_Atn_Dec_Default_Calc | = 26.116[dB] |
| Irr_Atn_Noe | = 26.116[dB] |
| Irr_Dec_Bandwidth_Hz | = 4.7826087[kHz] |
| Irr_Dec_Bandwidth_Ppm | = 11.96303566[ppm] |
| Irr_Dec_Freq | = 399.78219838[MHz] |
| Irr_Dec_Merit_Factor | = 2.2 |
| Irr_Decoupling | = TRUE |
| Irr_Noe | = TRUE |
| Irr_Noise | = WALTZ |
| Irr_Offset_Default | = 5[ppm] |
| Irr_Pwidth | = 0.115[ms] |
| Irr_Pwidth_Default | = 0.115[ms] |
| Irr_Pwidth_Default_Calc | = 0.115[ms] |
| Irr_Pwidth_Temp1 | = 0.115[ms] |
| Irr_Wurst | = FALSE |
| Decimation_Rate | = 0 |
| Initial_Wait | = 1[s] |
| Noe_Time | = 2[s] |
| Noe_Time_Flag | = FALSE |
| Relaxation_Delay_Calc | = 0[s] |
| Relaxation_Delay_Temp | = 2[s] |
| Repetition_Time | = 3.03809024[s] |

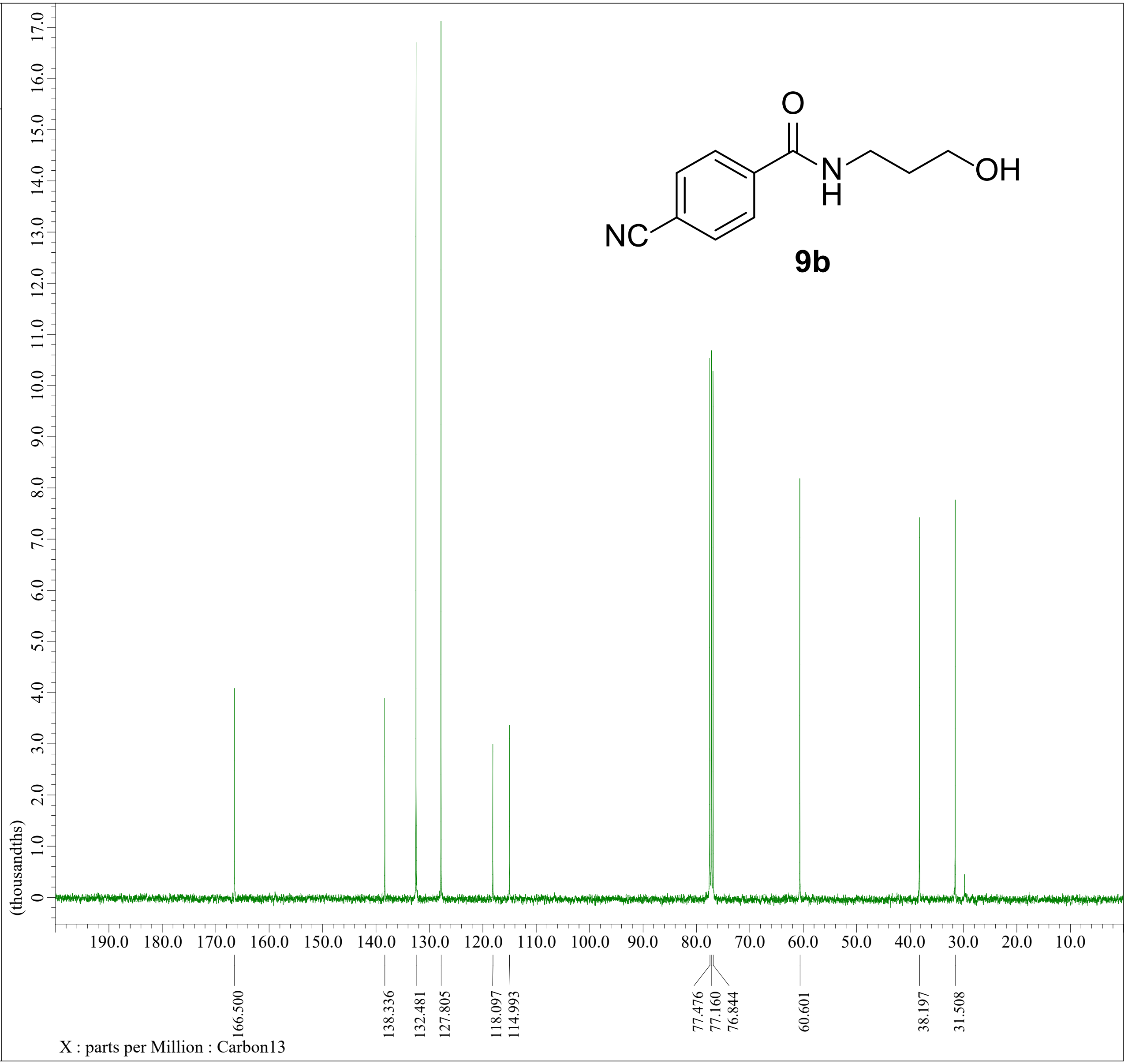

$^{1}$H NMR Spectrum of **9c** (300 MHz, $CDCl_3$)

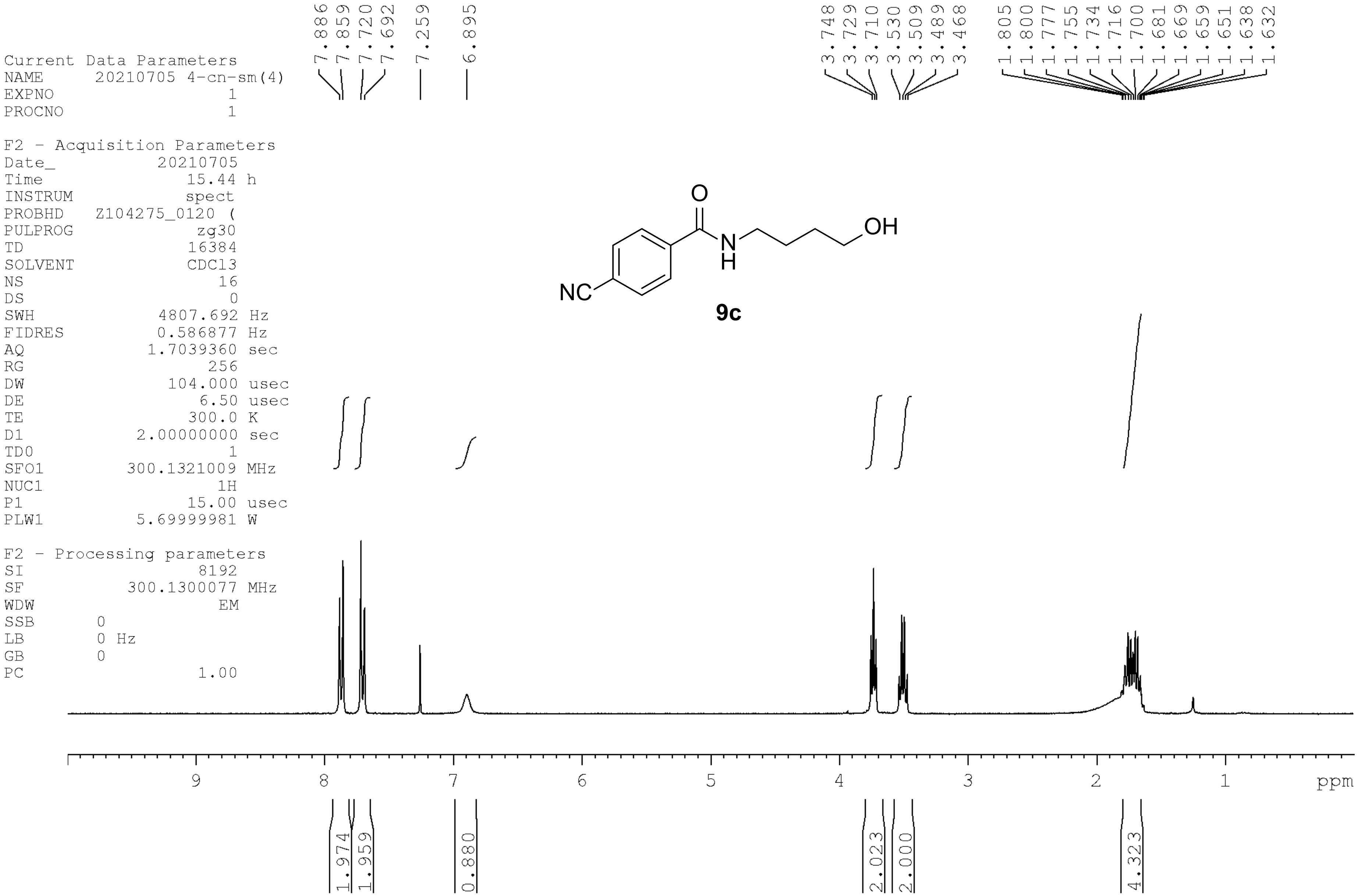

$^{13}C\{^{1}H\}$ NMR Spectrum of **9c** (100 MHz, $CDCl_3$)

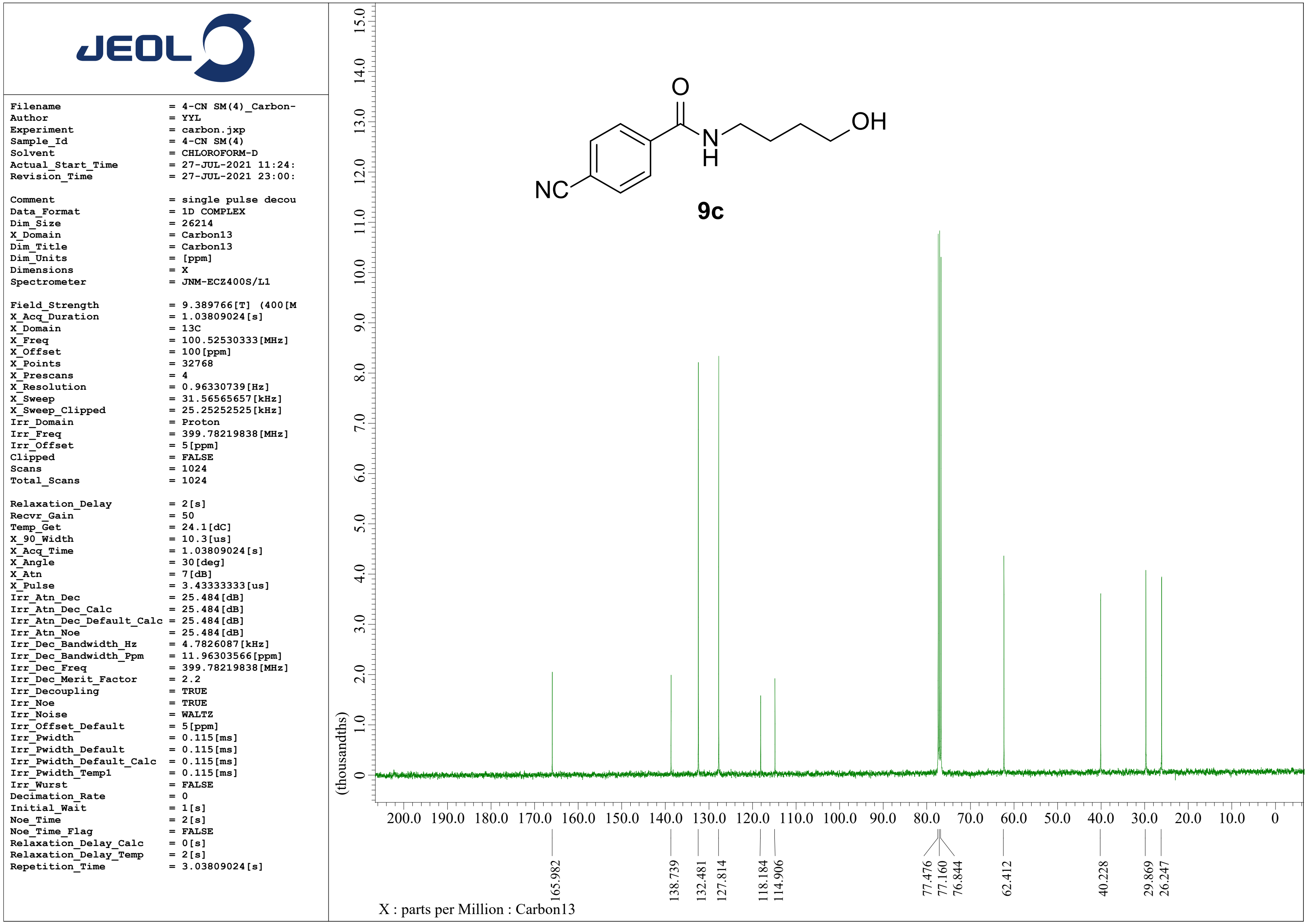

$^{1}$H NMR Spectrum of **9d** (300 MHz, $CDCl_3$)

```
Current Data Parameters
NAME      20210727 4-CN-PHCO-2nd amine
EXPNO                 1
PROCNO                1

F2 - Acquisition Parameters
Date_          20210727
Time              21.29 h
INSTRUM           spect
PROBHD   Z104275_0120 (
PULPROG            zg30
TD                16384
SOLVENT           CDCl3
NS                   12
DS                    0
SWH            4807.692 Hz
FIDRES         0.586877 Hz
AQ            1.7039360 sec
RG                  256
DW              104.000 usec
DE                 6.50 usec
TE                300.0 K
D1           2.00000000 sec
TD0                   1
SFO1        300.1321009 MHz
NUC1                 1H
P1                15.00 usec
PLW1         5.69999981 W

F2 - Processing parameters
SI                 8192
SF          300.1300076 MHz
WDW                  EM
SSB      0
LB       0 Hz
GB       0
PC                 1.00
```

**9d**

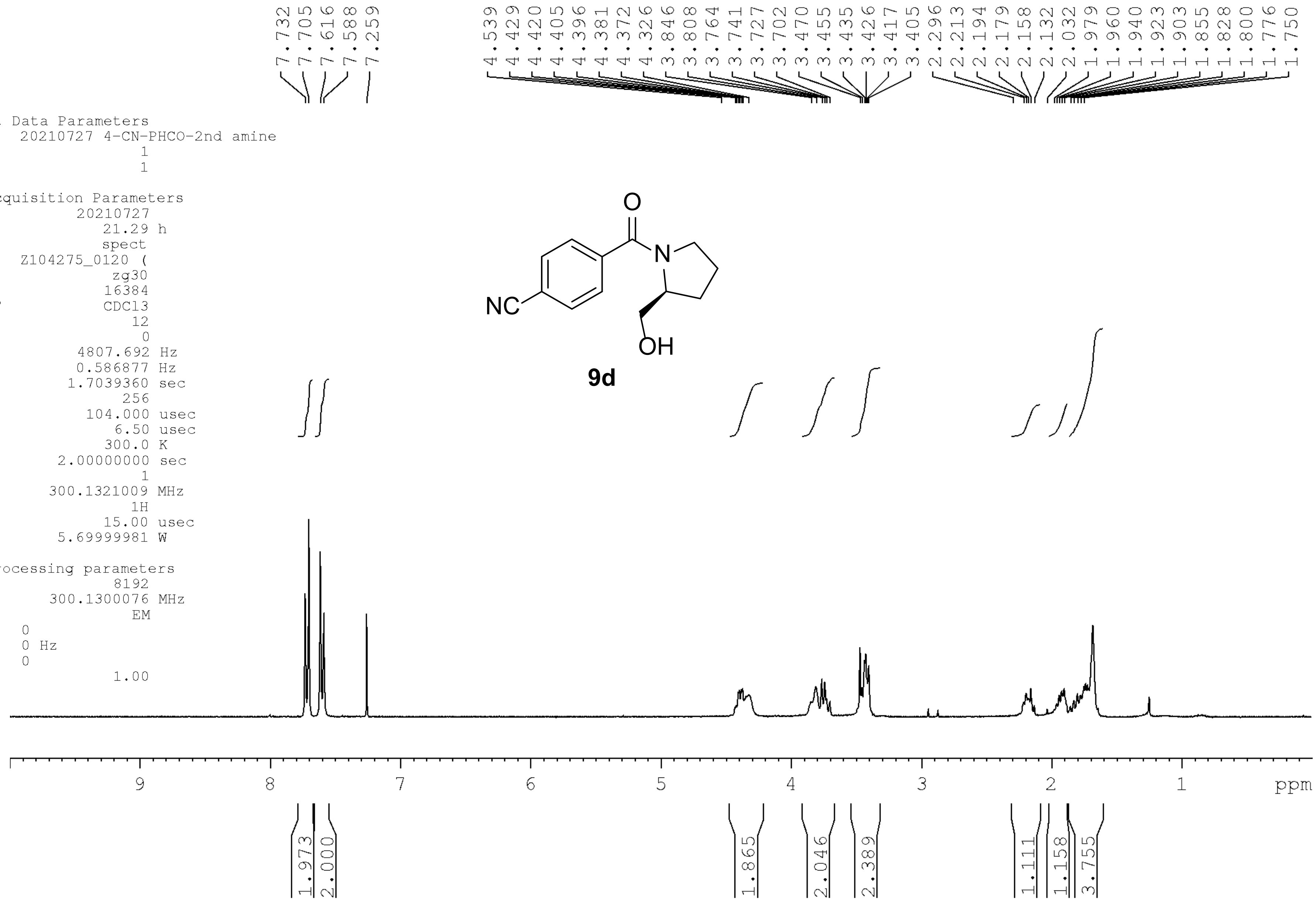

$^{13}C\{^{1}H\}$ NMR Spectrum of **9d** (100 MHz, $CDCl_3$)

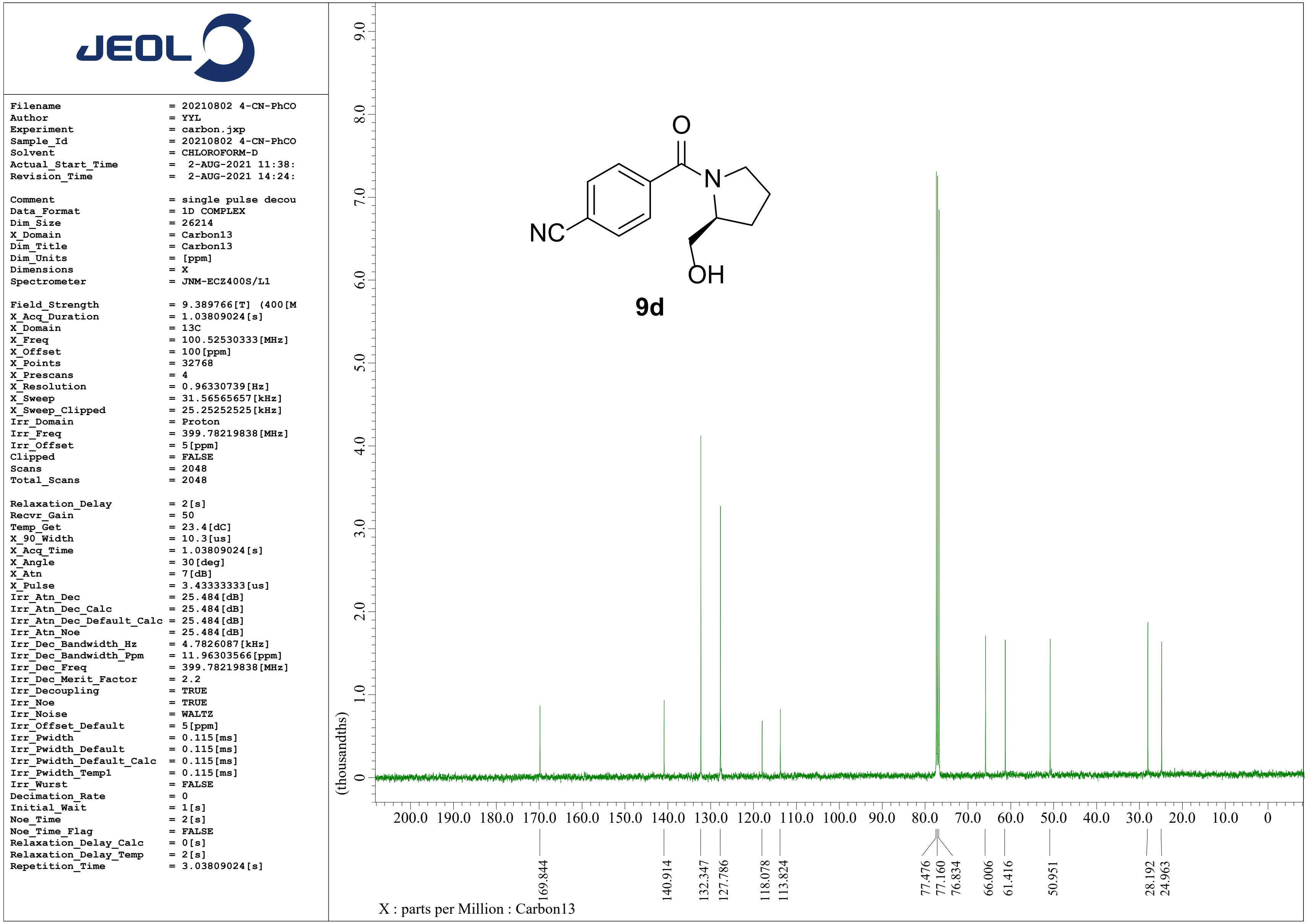

$^{1}$H NMR Spectrum of **10a** (300 MHz, $CDCl_3$)

Current Data Parameters
NAME 20210705 bg-sm(2)
EXPNO 1
PROCNO 1

F2 - Acquisition Parameters
Date_ 20210705
Time 16.43 h
INSTRUM spect
PROBHD Z104275_0120 (
PULPROG zg30
TD 16384
SOLVENT CDCl3
NS 16
DS 0
SWH 4807.692 Hz
FIDRES 0.586877 Hz
AQ 1.7039360 sec
RG 256
DW 104.000 usec
DE 6.50 usec
TE 300.0 K
D1 2.00000000 sec
TD0 1
SFO1 300.1321009 MHz
NUC1 1H
P1 15.00 usec
PLW1 5.69999981 W

F2 - Processing parameters
SI 8192
SF 300.1300077 MHz
WDW EM
SSB 0
LB 0 Hz
GB 0
PC 1.00

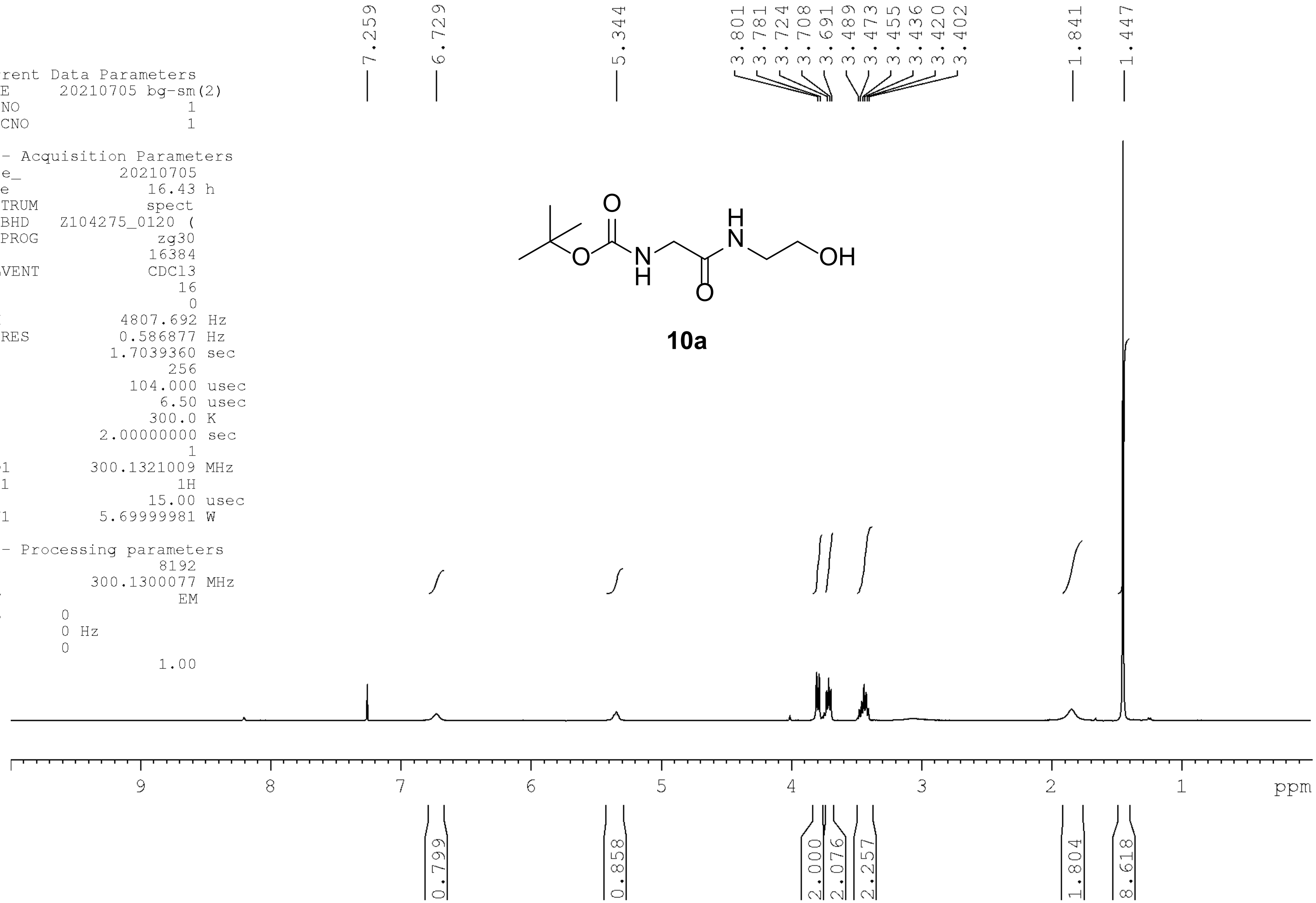

$^{1}$H NMR Spectrum of **10b** (300 MHz, $CDCl_3$)

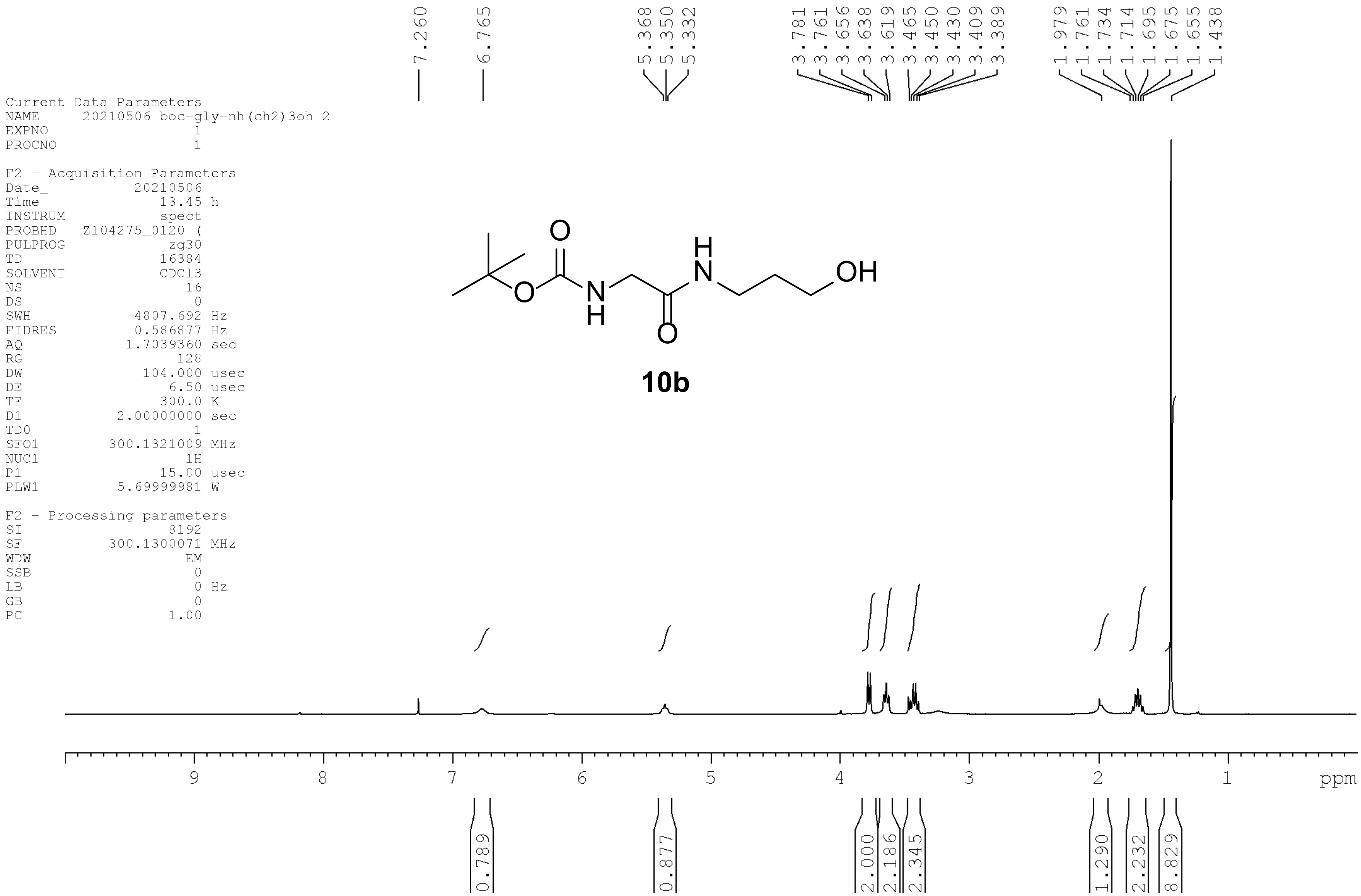

$^{1}$H NMR Spectrum of **10c** (300 MHz, $CDCl_3$)

```
Current Data Parameters
NAME      20210705 bg-sm(4-2)
EXPNO                    1
PROCNO                   1

F2 - Acquisition Parameters
Date_            20210705
Time                16.59 h
INSTRUM             spect
PROBHD   Z104275_0120 (
PULPROG              zg30
TD                  16384
SOLVENT             CDCl3
NS                     16
DS                      0
SWH              4807.692 Hz
FIDRES           0.586877 Hz
AQ              1.7039360 sec
RG                    228
DW                104.000 usec
DE                   6.50 usec
TE                  300.0 K
D1             2.00000000 sec
TD0                     1
SFO1          300.1321009 MHz
NUC1                   1H
P1                  15.00 usec
PLW1           5.69999981 W

F2 - Processing parameters
SI                   8192
SF            300.1300076 MHz
WDW                    EM
SSB      0
LB       0 Hz
GB       0
PC                   1.00
```

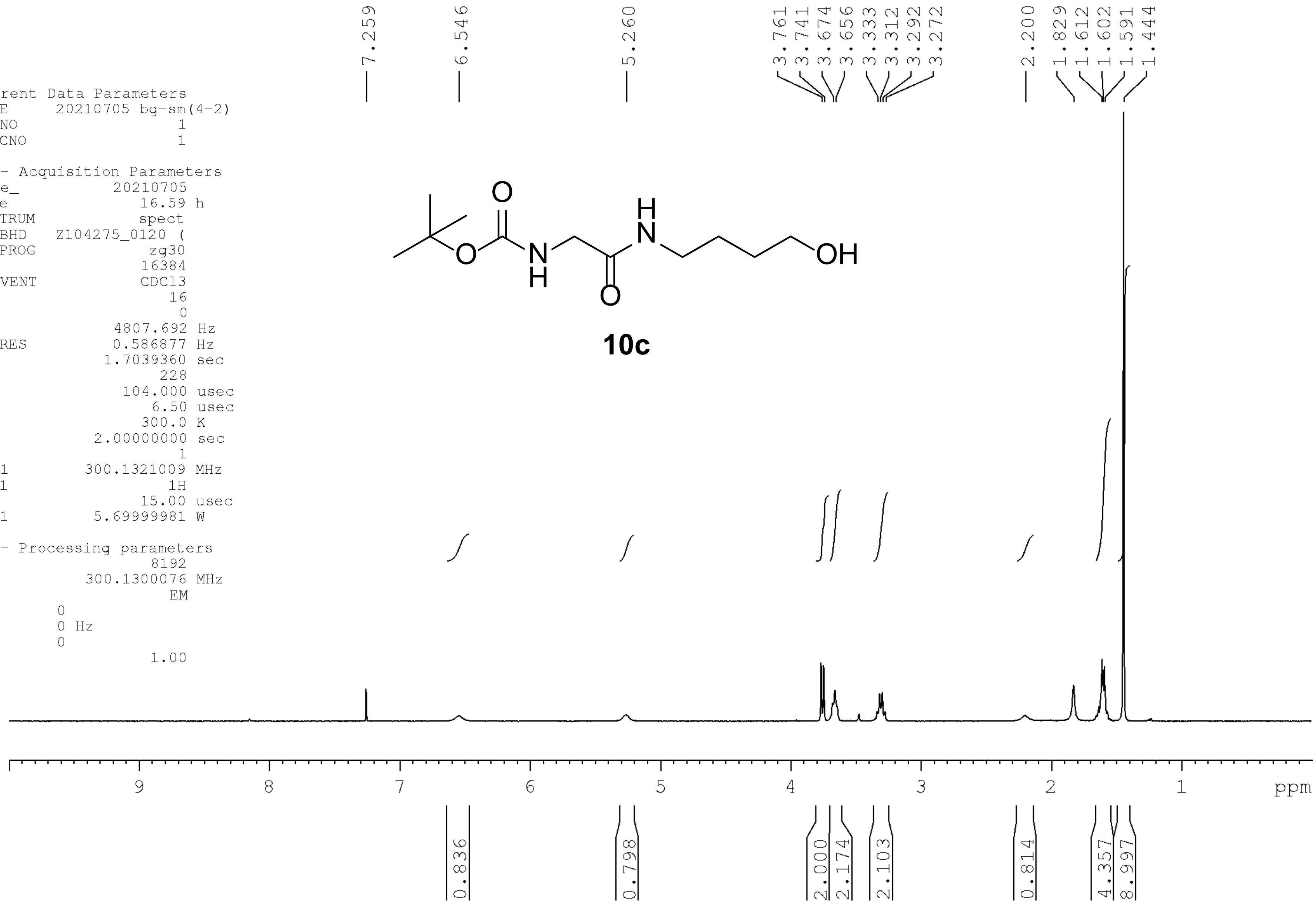

$^{13}C\{^{1}H\}$ NMR Spectrum of **10c** (100 MHz, $CDCl_3$)

| | |
|---|---|
| Filename | = BG-SM4_Carbon-1-2. |
| Author | = YYL |
| Experiment | = carbon.jxp |
| Sample_Id | = BG-SM4 |
| Solvent | = CHLOROFORM-D |
| Actual_Start_Time | = 9-AUG-2021 11:09: |
| Revision_Time | = 10-AUG-2021 11:50: |
| Comment | = single pulse decou |
| Data_Format | = 1D COMPLEX |
| Dim_Size | = 26214 |
| X_Domain | = Carbon13 |
| Dim_Title | = Carbon13 |
| Dim_Units | = [ppm] |
| Dimensions | = X |
| Spectrometer | = JNM-ECZ400S/L1 |
| Field_Strength | = 9.389766[T] (400[M |
| X_Acq_Duration | = 1.03809024[s] |
| X_Domain | = 13C |
| X_Freq | = 100.52530333[MHz] |
| X_Offset | = 100[ppm] |
| X_Points | = 32768 |
| X_Prescans | = 4 |
| X_Resolution | = 0.96330739[Hz] |
| X_Sweep | = 31.56565657[kHz] |
| X_Sweep_Clipped | = 25.25252525[kHz] |
| Irr_Domain | = Proton |
| Irr_Freq | = 399.78219838[MHz] |
| Irr_Offset | = 5[ppm] |
| Clipped | = FALSE |
| Scans | = 2048 |
| Total_Scans | = 2048 |
| Relaxation_Delay | = 2[s] |
| Recvr_Gain | = 50 |
| Temp_Get | = 23[dC] |
| X_90_Width | = 10.3[us] |
| X_Acq_Time | = 1.03809024[s] |
| X_Angle | = 30[deg] |
| X_Atn | = 7[dB] |
| X_Pulse | = 3.43333333[us] |
| Irr_Atn_Dec | = 25.484[dB] |
| Irr_Atn_Dec_Calc | = 25.484[dB] |
| Irr_Atn_Dec_Default_Calc | = 25.484[dB] |
| Irr_Atn_Noe | = 25.484[dB] |
| Irr_Dec_Bandwidth_Hz | = 4.7826087[kHz] |
| Irr_Dec_Bandwidth_Ppm | = 11.96303566[ppm] |
| Irr_Dec_Freq | = 399.78219838[MHz] |
| Irr_Dec_Merit_Factor | = 2.2 |
| Irr_Decoupling | = TRUE |
| Irr_Noe | = TRUE |
| Irr_Noise | = WALTZ |
| Irr_Offset_Default | = 5[ppm] |
| Irr_Pwidth | = 0.115[ms] |
| Irr_Pwidth_Default | = 0.115[ms] |
| Irr_Pwidth_Default_Calc | = 0.115[ms] |
| Irr_Pwidth_Temp1 | = 0.115[ms] |
| Irr_Wurst | = FALSE |
| Decimation_Rate | = 0 |
| Initial_Wait | = 1[s] |
| Noe_Time | = 2[s] |
| Noe_Time_Flag | = FALSE |
| Relaxation_Delay_Calc | = 0[s] |
| Relaxation_Delay_Temp | = 2[s] |
| Repetition_Time | = 3.03809024[s] |

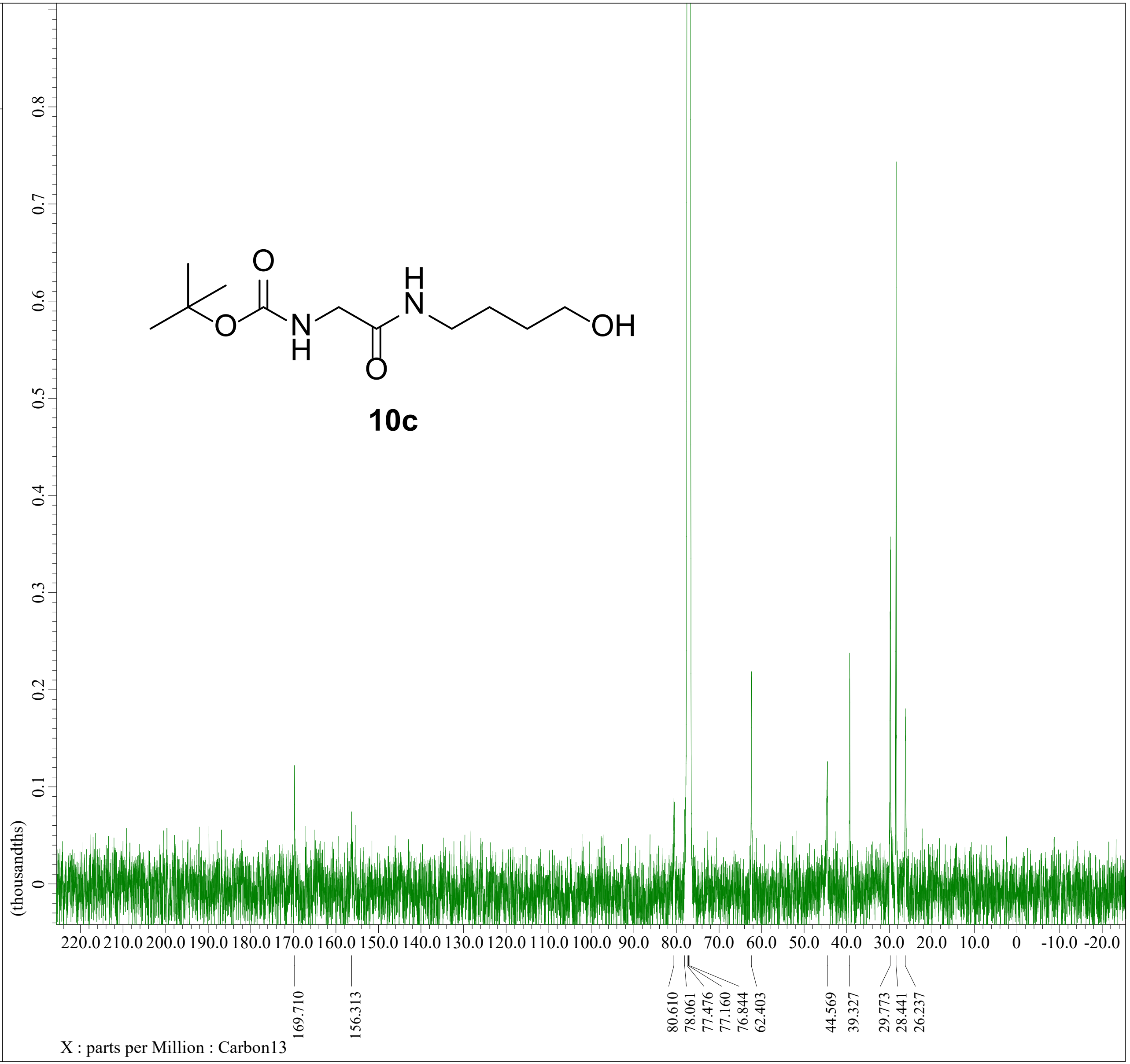

$^{1}$H NMR Spectrum of **10d** (300 MHz, $CDCl_3$)

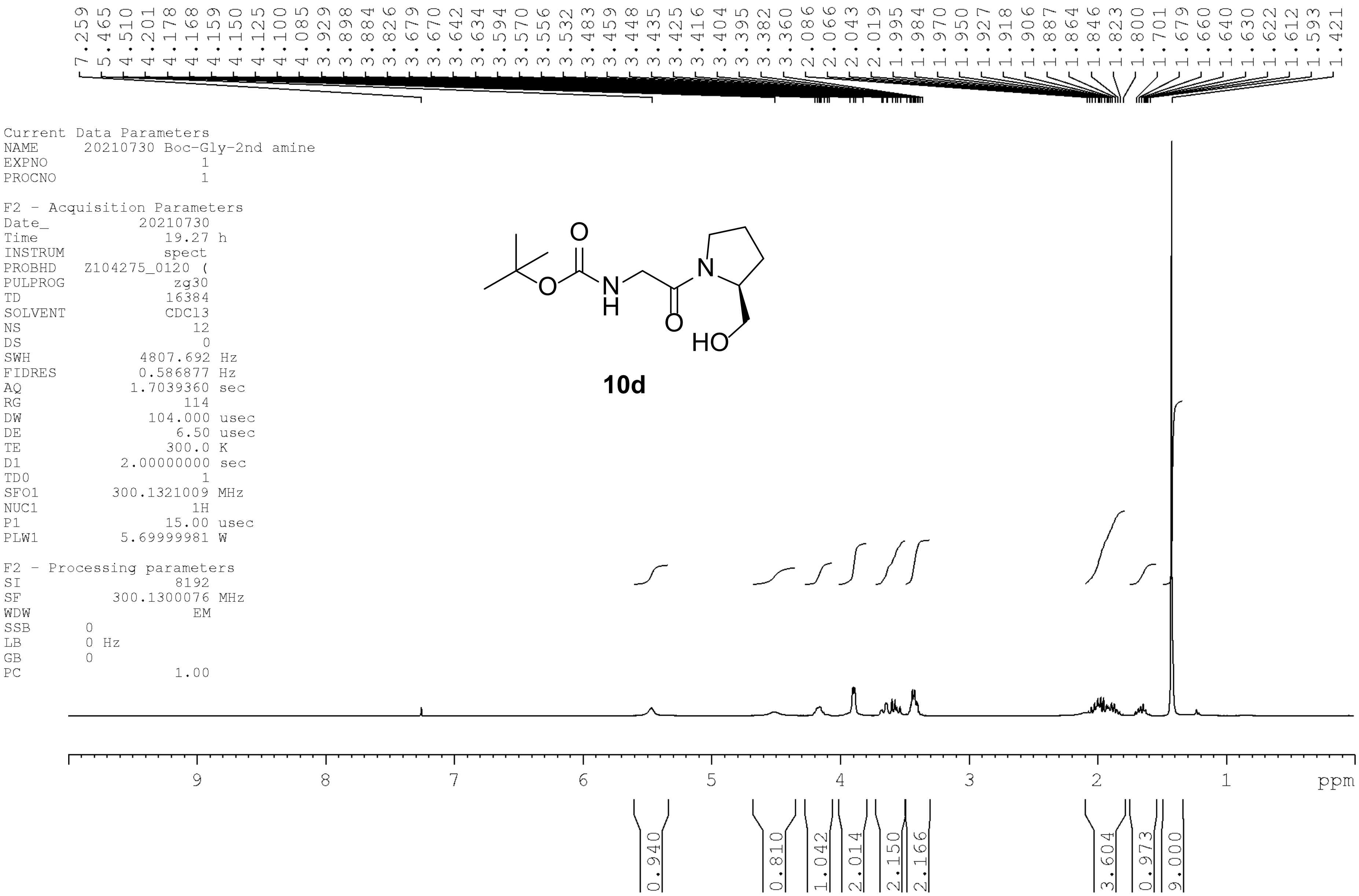

$^{13}C\{^{1}H\}$ NMR Spectrum of **10d** (100 MHz, $CDCl_3$)

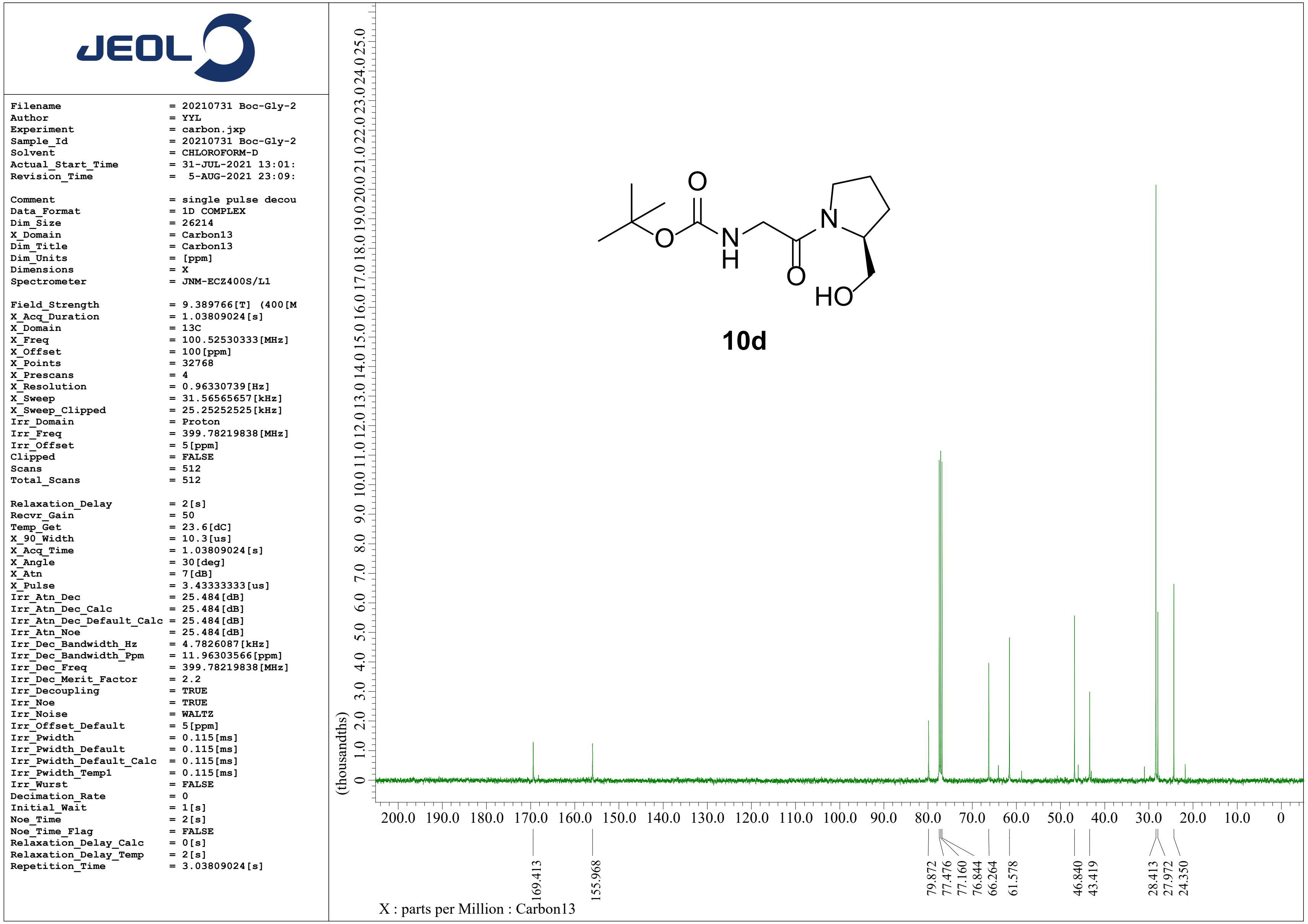

$^{1}$H NMR Spectrum of **11** (300 MHz, $CDCl_3$)

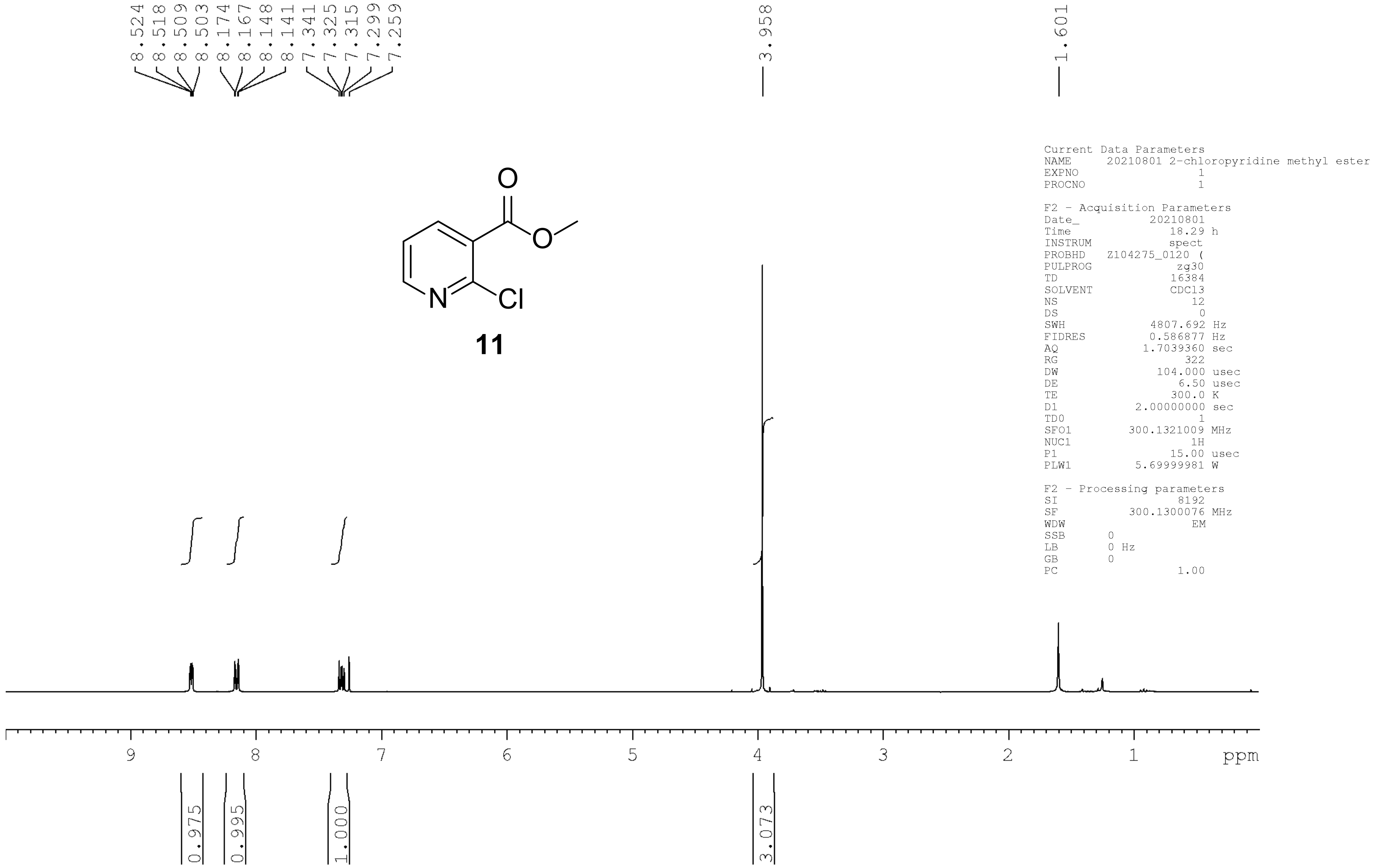

$^{13}C\{^1H\}$ NMR Spectrum of **11** (100 MHz, $CDCl_3$)

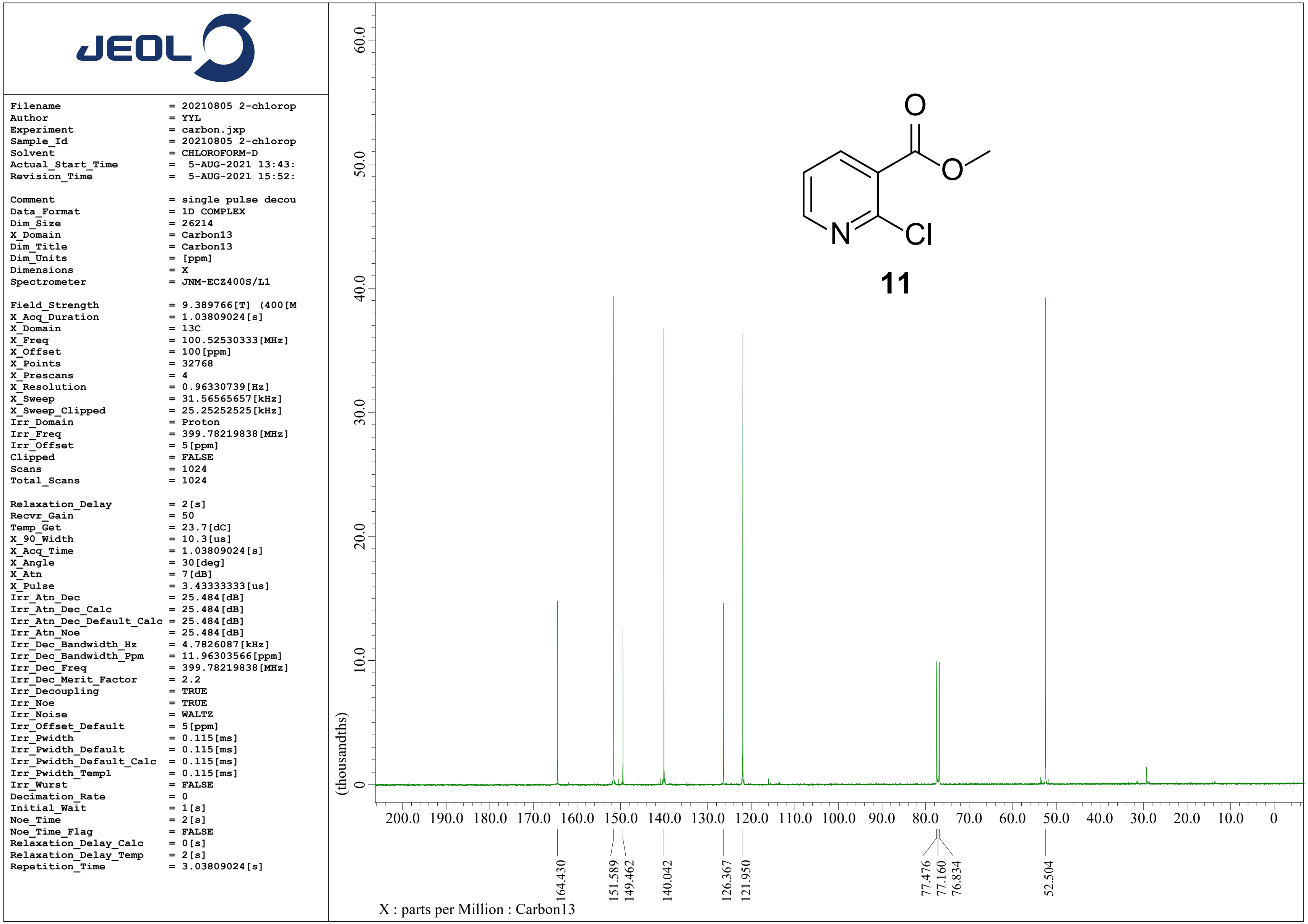

## $^1$H NMR Spectrum of **12** (300 MHz, $CDCl_3$)

```
Current Data Parameters
NAME     20210727 medicine-before
EXPNO                   1
PROCNO                  1

F2 - Acquisition Parameters
Date_           20210727
Time               17.58 h
INSTRUM            spect
PROBHD   Z104275_0120 (
PULPROG             zg30
TD                 16384
SOLVENT            CDCl3
NS                    12
DS                     0
SWH             4807.692 Hz
FIDRES          0.586877 Hz
AQ             1.7039360 sec
RG                   228
DW               104.000 usec
DE                  6.50 usec
TE                 300.0 K
D1            2.00000000 sec
TD0                    1
SFO1         300.1321009 MHz
NUC1                  1H
P1                 15.00 usec
PLW1          5.69999981 W

F2 - Processing parameters
SI                  8192
SF           300.1300076 MHz
WDW                   EM
SSB      0
LB       0 Hz
GB       0
PC                  1.00
```

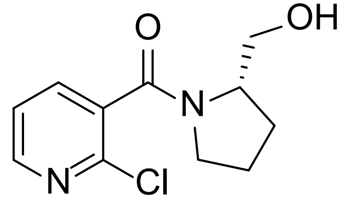


**12**

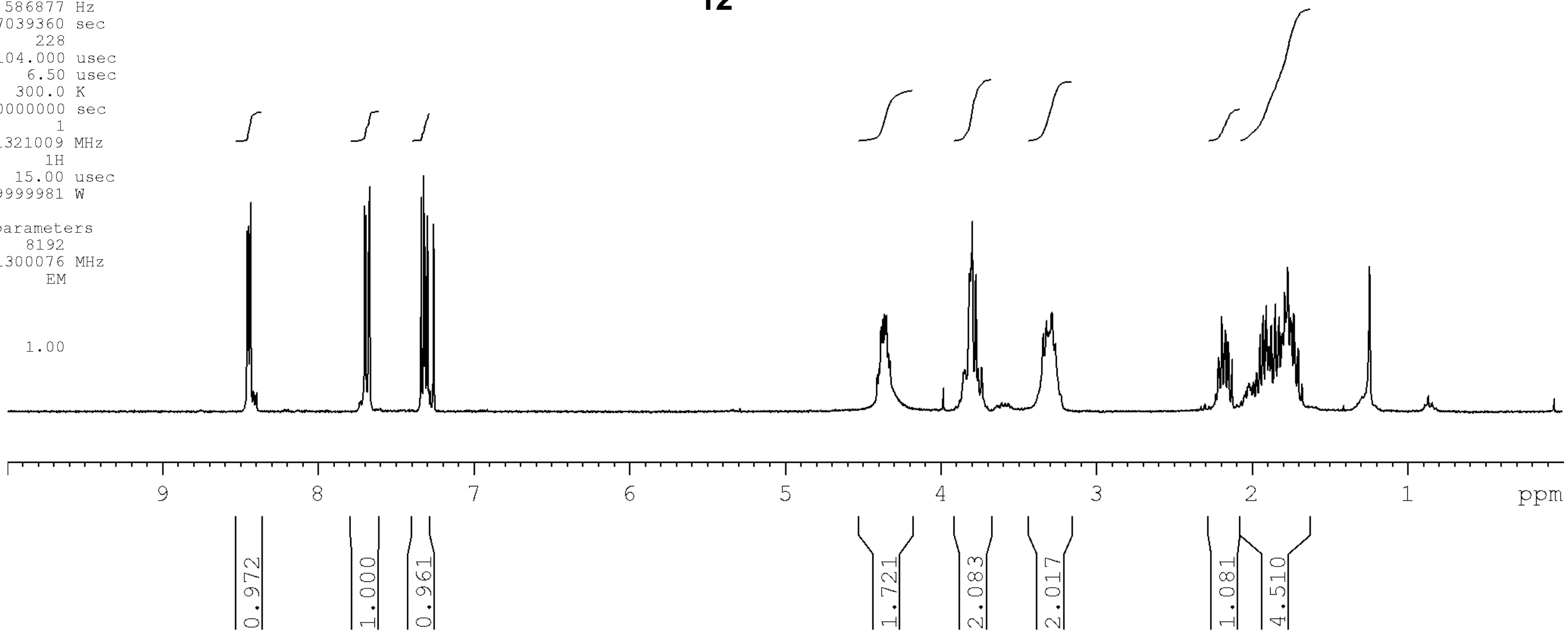

## $^1$H NMR Spectrum of **13** (300 MHz, $CDCl_3$)

```
Current Data Parameters
NAME      20210730 medicine-after-1-wash
EXPNO                    1
PROCNO                   1

F2 - Acquisition Parameters
Date_            20210730
Time                16.02 h
INSTRUM             spect
PROBHD   Z104275_0120 (
PULPROG              zg30
TD                  16384
SOLVENT             CDCl3
NS                     12
DS                      0
SWH              4807.692 Hz
FIDRES           0.586877 Hz
AQ              1.7039360 sec
RG                    256
DW                104.000 usec
DE                   6.50 usec
TE                  300.0 K
D1             2.00000000 sec
TD0                     1
SFO1          300.1321009 MHz
NUC1                   1H
P1                  15.00 usec
PLW1           5.69999981 W

F2 - Processing parameters
SI                   8192
SF            300.1300076 MHz
WDW                    EM
SSB      0
LB       0 Hz
GB       0
PC                   1.00
```

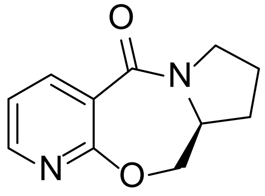

**13**

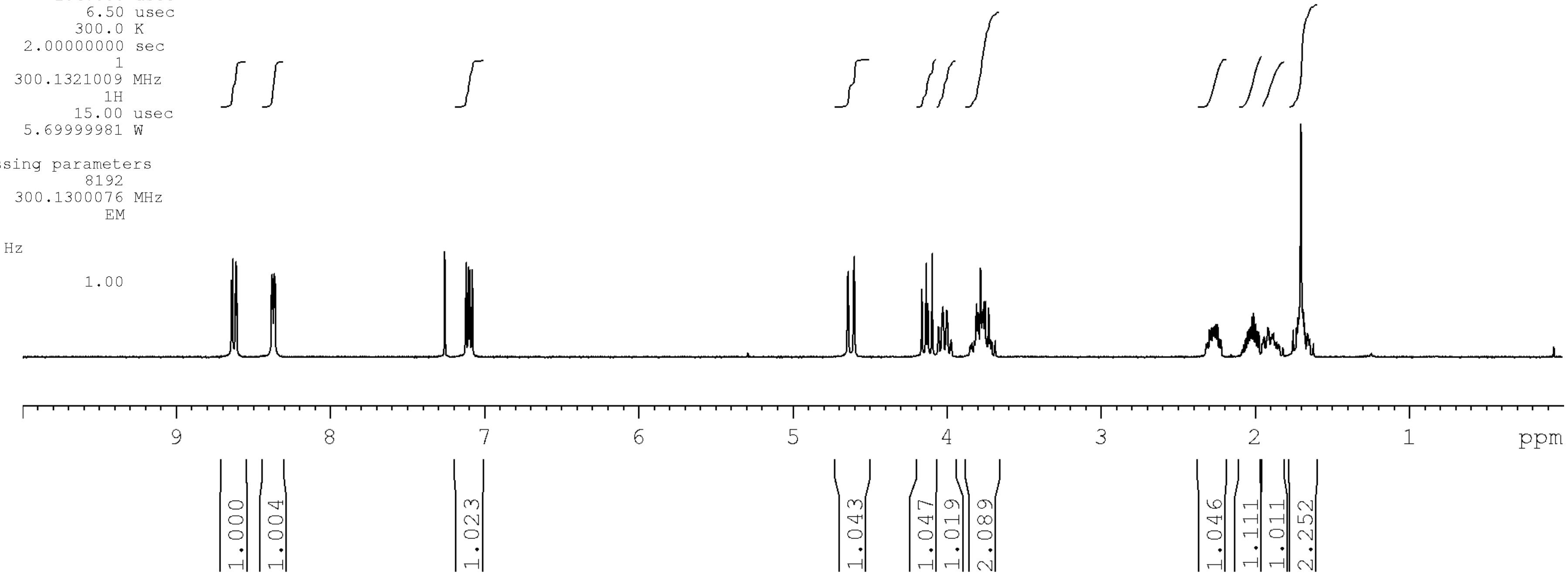

## Chiral HPLC Spectrum of Cbz-Gly-DL-Ser-NHHex

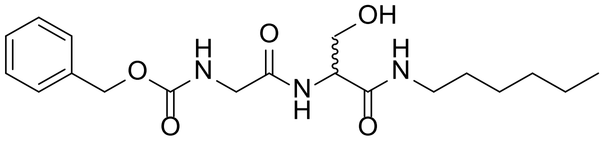


| | | | |
|---|---|---|---|
| **Data file:** | 2024-03-12 21-00-55+08-00-01.dx | | |
| **Sequence Name:** | 20240313 Cbz-Gly-Ser-Hex-2 IA 9010 0.7 mL (230 nm, 60 min) | **Project Name:** | LC1260 |
| **WEI-016-PA** | | **Operator:** | SYSTEM |
| **Instrument:** | LC1260 | **Injection date:** | 2024-03-12 21:02:03+08:00 |
| **Inj. volume:** | 25.000 | **Location:** | P2-D1 |
| **Acq. method:** | flow rate 0.7 mL per min(230nm, 60min).amx | **Type:** | Sample |
| **Processing method:** | GC_LC Quantitative_DefaultMethod.pmx | **Sample amount:** | 0.00 |
| **Manually modified:** | Manual Integration | | |

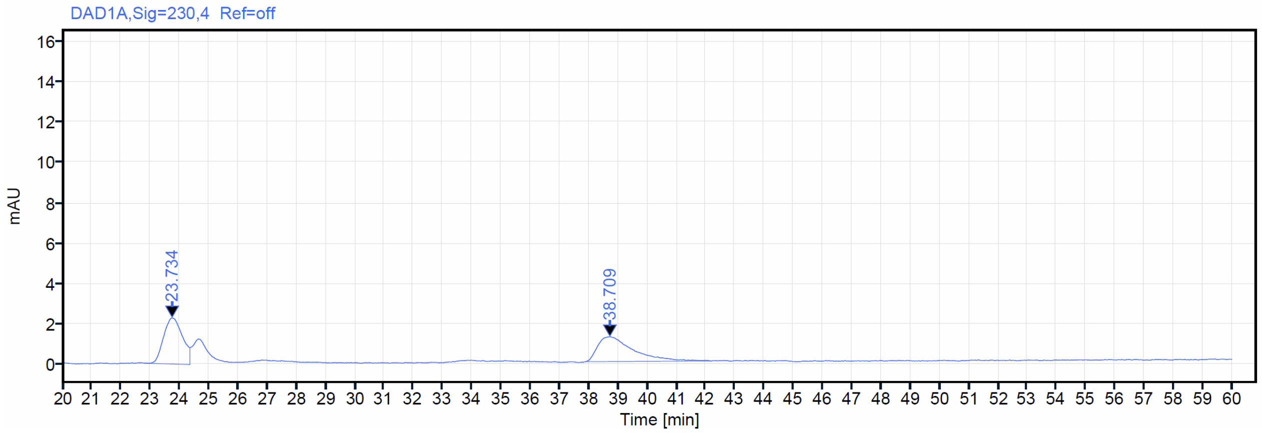


**Signal:** DAD1A,Sig=230,4 Ref=off

| RT [min] | Type | Width [min] | Area | Height | Area% | Name |
|---|---|---|---|---|---|---|
| 23.734 | MM m | 0.54 | 102.51 | 2.27 | 49.66 | |
| 38.709 | MM m | 1.00 | 103.92 | 1.22 | 50.34 | |
| | | Sum | 206.43 | | | |

Retention time:

23.734 min = Cbz-Gly-D-Ser-NHHex; 38.709 min = Cbz-Gly-L-Ser-NHHex

Conditions: 2-propanol/hexane = 1:9, v = 0.7 mL/min, λ = 230.4 nm

## Chiral HPLC Spectrum of Cbz-Gly-L-Ser-NHHex (**6ab**)

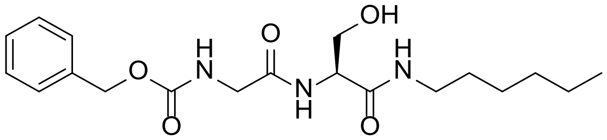


**6ab**

| | | | |
|---|---|---|---|
| **Data file:** | 2021-05-07 20-49-56+08-00-01.dx | | |
| **Sequence Name:** | 20210507 Cbz-Gly-Ser-Hex IA3 0.7 1090 | **Project Name:** | LC1260 |
| **WEI-016-PA** | | **Operator:** | SYSTEM |
| **Instrument:** | LC1260 | **Injection date:** | 2021-05-07 20:50:51+08:00 |
| **Inj. volume:** | 8.000 | **Location:** | P2-F3 |
| **Acq. method:** | 20200729_VS-PA TEST.amx | **Type:** | Sample |
| **Processing method:** | GC_LC Quantitative_DefaultMethod.pmx | **Sample amount:** | 0.00 |
| **Manually modified:** | Manual Integration | | |

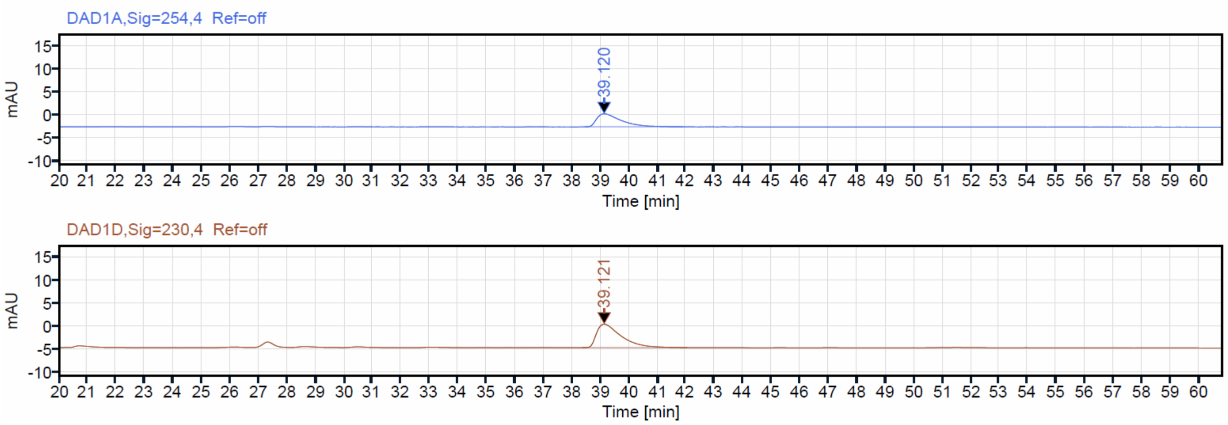


**Signal:** DAD1A,Sig=254,4 Ref=off

| RT [min] | Type | Width [min] | Area | Height | Area% | Name |
|---|---|---|---|---|---|---|
| 39.120 | BB | 3.44 | 168.11 | 2.81 | 100.00 | |
| | | Sum | 168.11 | | | |

**Signal:** DAD1D,Sig=230,4 Ref=off

| RT [min] | Type | Width [min] | Area | Height | Area% | Name |
|---|---|---|---|---|---|---|
| 39.121 | BB | 3.69 | 306.33 | 5.09 | 100.00 | |
| | | Sum | 306.33 | | | |

Retention time: 39.121 min

Conditions: 2-propanol/hexane = 1:9, v = 0.7 mL/min, λ = 230.4 nm

Chiral HPLC Spectrum of Boc-DL-Phe-DL-Ser-NHHex

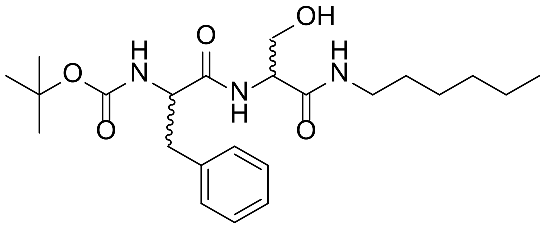

| | | | |
|---|---|---|---|
| **Data file:** | 2024-03-11 14-36-31+08-00-01.dx | | |
| **Sequence Name:** | 20240311 Boc-DL-Phe-Ser-Hex(IA 9010 0.7 ml, 230nm)-1 | **Project Name:** | LC1260 |
| **WEI-016-PA** | | **Operator:** | SYSTEM |
| **Instrument:** | LC1260 | **Injection date:** | 2024-03-11 14:37:24+08:00 |
| **Inj. volume:** | 5.000 | **Location:** | P2-B2 |
| **Acq. method:** | flow rate 0.7 mL per min(230nm, 60min).amx | **Type:** | Sample |
| **Processing method:** | GC_LC Quantitative_DefaultMethod.pmx | **Sample amount:** | 0.00 |
| **Manually modified:** | Manual Integration | | |

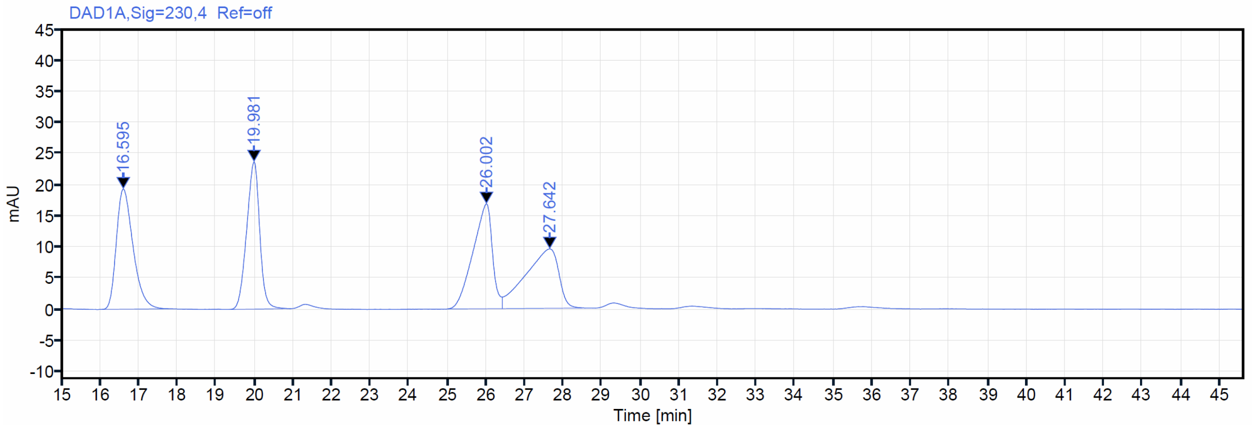


**Signal:** DAD1A,Sig=230,4 Ref=off

| RT [min] | Type | Width [min] | Area | Height | Area% | Name |
|---|---|---|---|---|---|---|
| 16.595 | BB | 1.74 | 580.92 | 19.30 | 25.27 | |
| 19.981 | BB | 1.60 | 554.47 | 23.65 | 24.12 | |
| 26.002 | BV | 1.47 | 590.60 | 16.84 | 25.69 | |
| 27.642 | VB | 2.16 | 572.53 | 9.54 | 24.91 | |
| | | Sum | 2298.52 | | | |

Retention time: 16.595 min; 19.981 min; 26.002 min; 27.642 min

Conditions: 2-propanol/hexane = 1:9, v = 0.7 mL/min, λ = 230.4 nm

Chiral HPLC Spectrum of Boc-L-Phe-L-Ser-NHHex (**6i**)

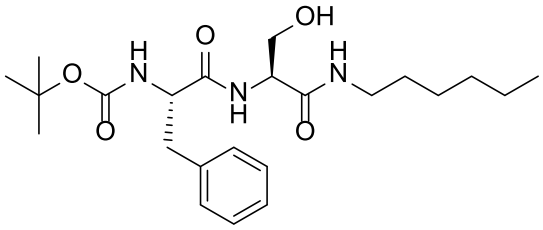

**6i**

| | | | |
|---|---|---|---|
| **Data file:** | 2021-03-15 16-09-34+08-00-01.dx | | |
| **Sequence Name:** | 20210315 Boc-Phe-Ser-Hex(IA-3 0.7 1090) | **Project Name:** | LC1260 |
| **WEI-016-PA** | | **Operator:** | SYSTEM |
| **Instrument:** | LC1260 | **Injection date:** | 2021-03-15 16:10:35+08:00 |
| **Inj. volume:** | 8.000 | **Location:** | P2-A1 |
| **Acq. method:** | test.amx | **Type:** | Sample |
| **Processing method:** | GC_LC Quantitative_DefaultMethod.pmx | **Sample amount:** | 0.00 |
| **Manually modified:** | Manual Integration | | |

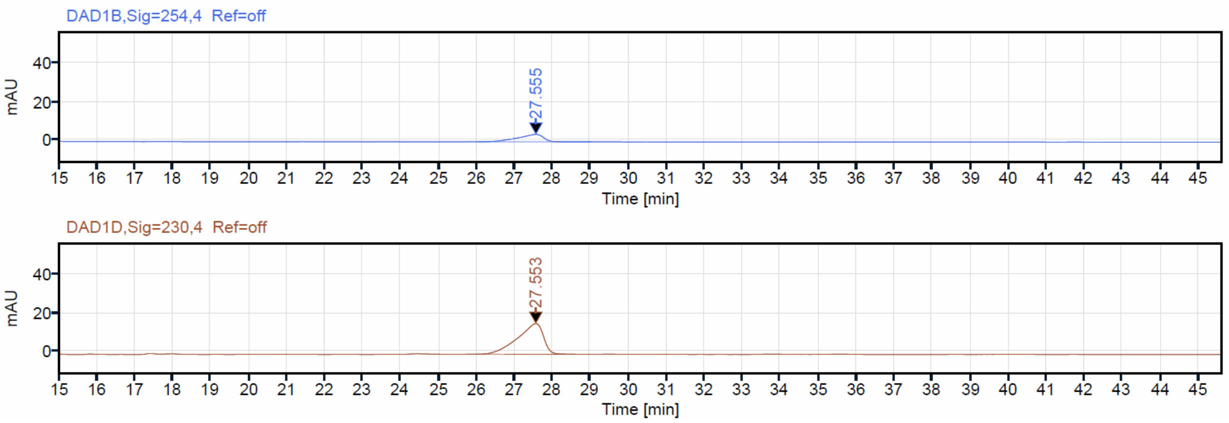


**Signal:** DAD1B,Sig=254,4 Ref=off

| RT [min] | Type | Width [min] | Area | Height | Area% | Name |
|---|---|---|---|---|---|---|
| 27.555 | BB | 2.97 | 181.43 | 3.83 | 100.00 | |
| | | Sum | 181.43 | | | |

**Signal:** DAD1D,Sig=230,4 Ref=off

| RT [min] | Type | Width [min] | Area | Height | Area% | Name |
|---|---|---|---|---|---|---|
| 27.553 | BB | 2.88 | 756.90 | 15.89 | 100.00 | |
| | | Sum | 756.90 | | | |

Retention time: 27.553 min

Conditions: 2-propanol/hexane = 1:9, v = 0.7 mL/min, λ = 230.4 nm

Chiral HPLC Spectrum of Boc-DL-Phg-DL-Ser-NHHex

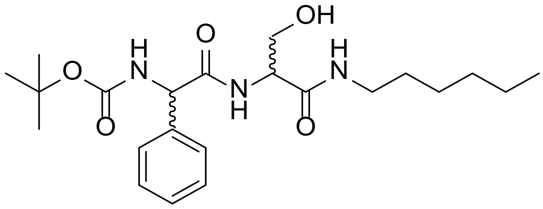

| | | | |
|---|---|---|---|
| **Data file:** | 2024-03-11 13-35-27+08-00-01.dx | | |
| **Sequence Name:** | 20240311 Boc-DL-Phg-Ser-Hex(IA 9010 0.7 ml, 230nm)-1 | **Project Name:** | LC1260 |
| **WEI-016-PA** | | **Operator:** | SYSTEM |
| **Instrument:** | LC1260 | **Injection date:** | 2024-03-11 13:36:18+08:00 |
| **Inj. volume:** | 5.000 | **Location:** | P2-A2 |
| **Acq. method:** | flow rate 0.7 mL per min(230nm, 60min).amx | **Type:** | Sample |
| **Processing method:** | GC_LC Quantitative_DefaultMethod.pmx | **Sample amount:** | 0.00 |
| **Manually modified:** | Manual Integration | | |

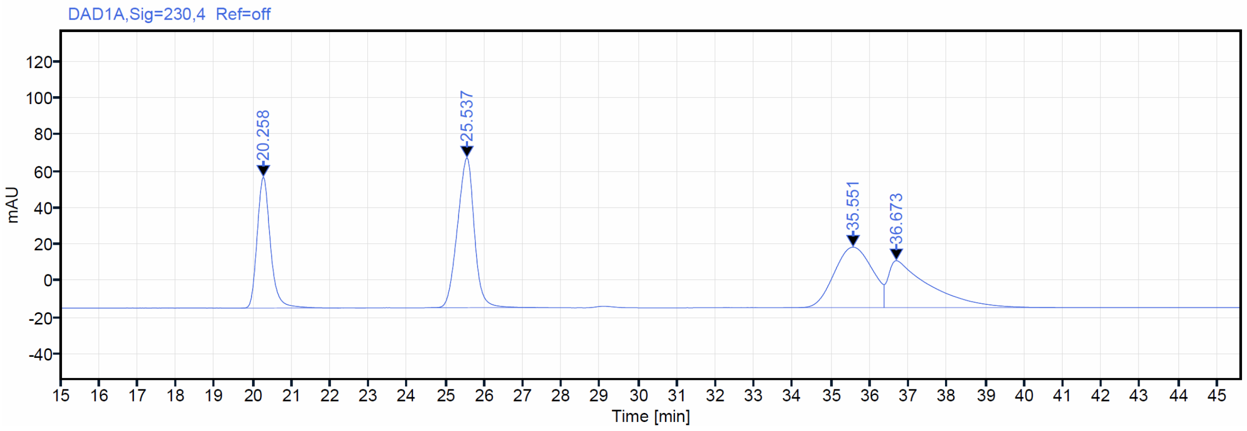


**Signal:** DAD1A,Sig=230,4 Ref=off

| RT [min] | Type | Width [min] | Area | Height | Area% | Name |
|---|---|---|---|---|---|---|
| 20.258 | BB | 2.53 | 1708.50 | 71.55 | 20.42 | |
| 25.537 | BB | 2.81 | 2484.69 | 81.89 | 29.70 | |
| 35.551 | BV | 2.21 | 2253.12 | 33.03 | 26.93 | |
| 36.673 | VB | 4.44 | 1919.48 | 25.64 | 22.94 | |
| | | Sum | 8365.79 | | | |

Retention time: 20.258 min; 25.537 min; 35.551 min; 36.673 min

Conditions: 2-propanol/hexane = 1:9, v = 0.7 mL/min, λ = 230.4 nm

Chiral HPLC Spectrum of Boc-L-Phg-L-Ser-NHHex (**6u**)

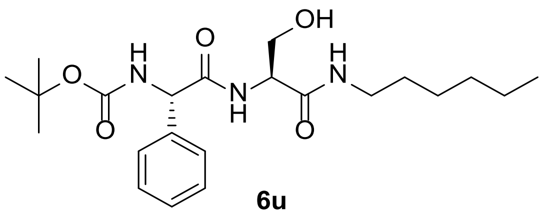

**6u**

| | | | |
|---|---|---|---|
| **Data file:** | 2021-03-11 10-21-29+08-00-01.dx | | |
| **Sequence Name:** | 20210311 Boc-Phg-Ser-Hex (IA-0.7-10-90, 230nm) | **Project Name:** | LC1260 |
| **WEI-016-PA** | | **Operator:** | SYSTEM |
| **Instrument:** | LC1260 | **Injection date:** | 2021-03-11 10:22:31+08:00 |
| **Inj. volume:** | 8.000 | **Location:** | P2-A2 |
| **Acq. method:** | test.amx | **Type:** | Sample |
| **Processing method:** | GC_LC Quantitative_DefaultMethod.pmx | **Sample amount:** | 0.00 |
| **Manually modified:** | Manual Integration | | |

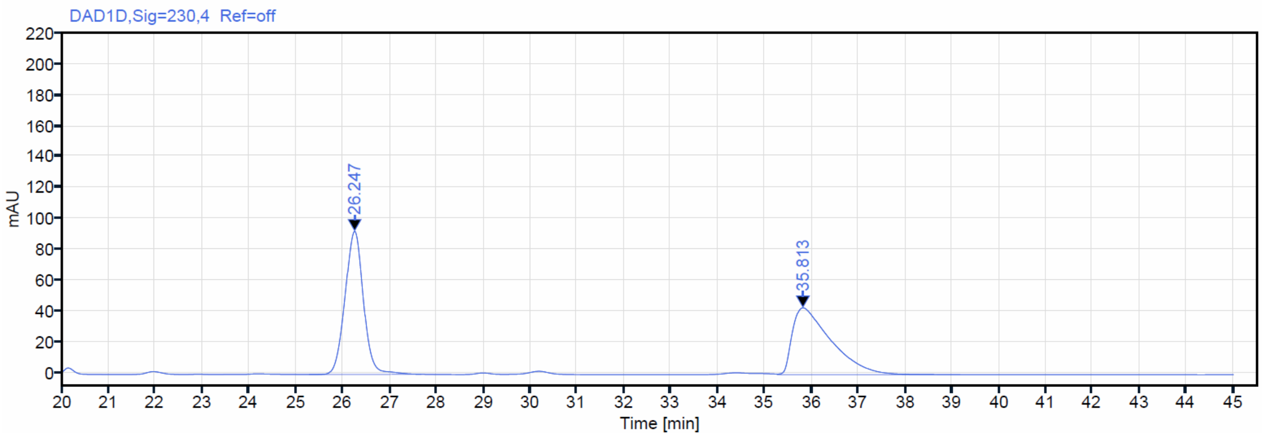


**Signal:** DAD1D,Sig=230,4 Ref=off

| RT [min] | Type | Width [min] | Area | Height | Area% | Name |
|---|---|---|---|---|---|---|
| 26.247 | BM m | 0.42 | 2544.87 | 92.73 | 50.61 | |
| 35.813 | VB | 3.90 | 2483.05 | 43.29 | 49.39 | |
| | | Sum | 5027.92 | | | |

Retention time: 26.247 & 35.813 min, Area% ~1:1

Conditions: 2-propanol/hexane = 1:9, v = 0.7 mL/min, λ = 230.4 nm

**References:**


1. Chan, W.-K.; Ho, C.-M.; Wong, M.-K.; Che, C.-M., *J. Am. Chem. Soc.* **2006,** *128*, 14796-14797.
2. Jiang, S.-Y.; Hsieh, W.-T.; Chen, W.-S.; Liao, J.-S.; Chiang, P.-Y.; Lin, Y. A., *Asian J. Org. Chem.* **2020,** *9*, 1638-1649.
3. Purushottam, L.; Adusumalli, S. R.; Singh, U.; Unnikrishnan, V. B.; Rawale, D. G.; Gujrati, M.; Mishra, R. K.; Rai, V., *Nat. Commun.* **2019,** *10*, 2539.
4. Sabatini, M. T.; Karaluka, V.; Lanigan, R. M.; Boulton, L. T.; Badland, M.; Sheppard, T. D., *Chem. Eur. J.* **2018,** *24*, 7033-7043.
5. Maheswara Rao, B. L.; Nowshuddin, S.; Jha, A.; Divi, M. K.; Rao, M. N. A., *Synth. Commun.* **2017,** *47*, 2127-2132.
6. Horikawa, R.; Fujimoto, C.; Yazaki, R.; Ohshima, T., *Chem. Eur. J.* **2016,** *22*, 12278-12281.
7. Konnert, L.; Lamaty, F.; Martinez, J.; Colacino, E., *J. Org. Chem.* **2014,** *79*, 4008-4017.
8. Di Gioia, M. L.; Gagliardi, A.; Leggio, A.; Leotta, V.; Romio, E.; Liguori, A., *RSC Adv.* **2015,** *5*, 63407-63420.
9. Nathanael, J. G.; Wille, U., *J. Org. Chem.* **2019,** *84*, 3405-3418.
10. Ji, Y.; Sweeney, J.; Zoglio, J.; Gorin, D. J., *J. Org. Chem.* **2013,** *78*, 11606-11611.
11. Jahns, C.; Hoffmann, T.; Müller, S.; Gerth, K.; Washausen, P.; Höfle, G.; Reichenbach, H.; Kalesse, M.; Müller, R., *Angew. Chem. Int. Ed.* **2012,** *51*, 5239-5243.
12. Sudhakar, G.; Bayya, S.; Reddy, K. J.; Sridhar, B.; Sharma, K.; Bathula, S. R., *Eur. J. Org. Chem.* **2014,** *2014*, 1253-1265.
13. Di Gioia, M.; Barattucci, A.; Bonaccorsi, P.; Leggio, A.; Minuti, L.; Romio, E.; Temperini, A.; Siciliano, C., *RSC Adv.* **2014,** *4*, 2678-2686.
14. Fisher, B. F.; Hong, S. H.; Gellman, S. H., *J. Am. Chem. Soc.* **2018,** *140*, 9396-9399.
15. Xu, J.; Lin, B.; Jiang, X.; Jia, Z.; Wu, J.; Dai, W.-M., *Org. Lett.* **2019,** *21*, 830-834.
16. Bhushan, B.; Lin, Y. A.; Bak, M.; Phanumartwiwath, A.; Yang, N.; Bilyard, M. K.; Tanaka, T.; Hudson, K. L.; Lercher, L.; Stegmann, M., *J. Am. Chem. Soc.* **2018,** *140*, 14599-14603.
17. Barraclough, P.; Dieterich, P.; Spray, C. A.; Young, D. W., *Org. Biomol. Chem.* **2006,** *4*, 1483-1491.
18. Reay, A. J.; Williams, T. J.; Fairlamb, I. J. S., *Org. Biomol. Chem.* **2015,** *13*, 8298-8309.
19. Brandstätter, M.; Roth, F.; Luedtke, N. W., *J. Org. Chem.* **2015,** *80*, 40-51.
20. Tsuji, H.; Yamamoto, H., *J. Am. Chem. Soc.* **2016,** *138*, 14218-14221.
21. Maurits, E.; van de Graaff, M. J.; Maiorana, S.; Wander, D. P. A.; Dekker, P. M.; van der Zanden, S. Y.; Florea, B. I.; Neefjes, J. J. C.; Overkleeft, H. S.; van Kasteren, S. I., *J. Am. Chem. Soc.* **2020,** *142*, 7250-7253.
22. Wei, Y.; He, W.; Liu, Y.; Liu, P.; Zhang, S., *Org. Lett.* **2012,** *14*, 704-707.
23. Muramatsu, W.; Hattori, T.; Yamamoto, H., *J. Am. Chem. Soc.* **2019,** *141*, 12288-12295.
24. Tossi, A.; Benedetti, F.; Norbedo, S.; Skrbec, D.; Berti, F.; Romeo, D., *Bioorg. Med. Chem.* **2003,**

*11*, 4719-4727.

25. Zhu, Y.; Yan, H.; Lu, L.; Liu, D.; Rong, G.; Mao, J., *J. Org. Chem.* **2013,** *78*, 9898-9905.
26. Jiang, Y.; Pan, S.; Zhang, Y.; Yu, J.; Liu, H., *Eur. J. Org. Chem.* **2014,** *2014*, 2027-2031.
27. Yamada, K.; Hayakawa, N.; Fujita, H.; Kitamura, M.; Kunishima, M., *Eur. J. Org. Chem.* **2016,** *2016*, 4093-4098.
28. Procopio, A.; Costanzo, P.; Curini, M.; Nardi, M.; Oliverio, M.; Paonessa, R., *Synthesis* **2011,** *2011*, 73-78.
29. Buer, B. C.; Levin, B. J.; Marsh, E. N. G., *J. Pept. Sci.* **2013,** *19*, 308-314.
30. Kaur, B.; Kaur, M.; Kaur, N.; Garg, S.; Bhatti, R.; Singh, P., *J. Med. Chem.* **2019,** *62*, 6363-6376.
