# Supplementary figures and images for: Cesium Carbonate Promoted Direct Amidation of Unactivated Esters with Amino Alcohol Derivatives
Source: J Org Chem. 2024 Mar 25;89(7):4958–70. doi: 10.1021/acs.joc.4c00162 (PMC11002823; doi:10.1021/acs.joc.4c00162)

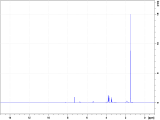

Supplement: Supplementary file 2 — jo4c00162_si_002.zip [file jo4c00162_si_002.zip › FID for publication/10a-Boc-Gly-NH(CH)2OH/H/20210705 bg-sm(2)/1/pdata/1/thumb.png]

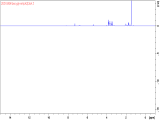

Supplement: Supplementary file 2 — jo4c00162_si_002.zip [file jo4c00162_si_002.zip › FID for publication/10b-Boc-Gly-NH(CH)3OH/H/20210506 boc-gly-nh(ch2)3oh 2/1/pdata/1/thumb.png]

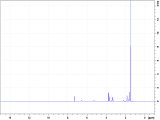

Supplement: Supplementary file 2 — jo4c00162_si_002.zip [file jo4c00162_si_002.zip › FID for publication/10c-Boc-Gly-NH(CH)4OH/H/20210705 bg-sm(4-2)/1/pdata/1/thumb.png]

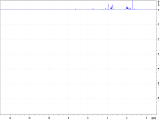

Supplement: Supplementary file 2 — jo4c00162_si_002.zip [file jo4c00162_si_002.zip › FID for publication/10d-Boc-Gly-2 nd amine/H/20210730 Boc-Gly-2nd amine/1/pdata/1/thumb.png]

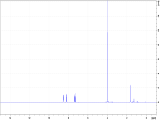

Supplement: Supplementary file 2 — jo4c00162_si_002.zip [file jo4c00162_si_002.zip › FID for publication/11/H/20210801 2-chloropyridine methyl ester/1/pdata/1/thumb.png]

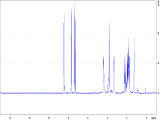

Supplement: Supplementary file 2 — jo4c00162_si_002.zip [file jo4c00162_si_002.zip › FID for publication/12/H/20210727 medicine-before/1/pdata/1/thumb.png]
